# The circadian rhythm: A key variable in aging?

**DOI:** 10.1111/acel.14268

**Published:** 2024-07-30

**Authors:** Patrick R. Winterhalter, Adrian‐Iustin Georgevici, Nitin J. Gharpure, Gábor Szabó, Andreas Simm

**Affiliations:** ^1^ Clinic for Heart Surgery (UMH) Martin‐Luther‐University Halle‐Wittenberg Halle (Saale) Germany; ^2^ Department of Anaesthesiology and Intensive Care Medicine St. Josef‐Hospital Ruhr‐University Bochum Bochum Germany; ^3^ Department of Cardiac Surgery University of Heidelberg Heidelberg Germany

**Keywords:** aging hallmarks, Bhlhe40 (Dec1), circadian rhythms, interaction network, machine learning, mouse cohort, organ strain sex, sex hormones

## Abstract

The determination of age‐related transcriptional changes may contribute to the understanding of health and life expectancy. The broad application of results from age cohorts may have limitations. Altering sample sizes per time point or sex, using a single mouse strain or tissue, a limited number of replicates, or omitting the middle of life can bias the surveys. To achieve higher general validity and to identify less distinctive players, bulk RNA sequencing of a mouse cohort, including seven organs of two strains from both sexes of 5 ages, was performed. Machine learning by bootstrapped variable importance and selection methodology (Boruta) was used to identify common aging features where the circadian rhythms (CiR) transcripts appear as promising age markers in an unsupervised analysis. Pathways of 11 numerically analyzed local network clusters were affected and classified into four major gene expression profiles, whereby CiR and proteostasis candidates were particularly conspicuous with partially opposing changes. In a data‐based interaction association network, the CiR‐proteostasis axis occupies an exposed central position, highlighting its relevance. The computation of 11,830 individual transcript associations provides potential superordinate contributors, such as hormones, to age‐related changes, as in CiR. In hormone‐sensitive LNCaP cells, short‐term supraphysiologic levels of the sex hormones dihydrotestosterone or estradiol increase the expression of the CiR transcript Bhlhe40 and the associated senescence regulator Cdkn2b (p15). According to these findings, the bilateral dysregulation of CiR appears as a fundamental protagonist of aging, whose transcripts could serve as a biological marker and its restoration as a therapeutic opportunity.

## Graphical abstract

In this study, general age‐related transcriptional trajectories in seven organs were investigated over the lifespan of mice. Since both sexes and two strains were used equally, biases in this regard are diminished. Twenty replicas per time provide a deeper insight than usual. We discovered that the circadian rhythm has a more substantial impact than previously assumed. The CiR reveals the most pronounced age associations and opposing expression profiles, suggesting the deregulation of its negative feedback loop. The CiR and proteostasis appear to be the central hub in a self‐created aging network. Computation predicts hormonal effects as a potential CiR modulator.

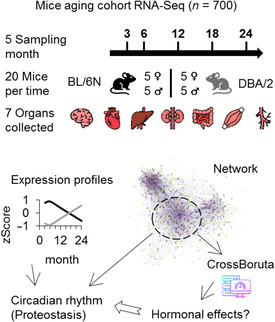

**Abbreviations**
0610009B22Rik: RIKEN cDNA 0610009B22 gene
AAS: Asymmetric Association Strength
AGE‐RAGE: Advanced Glycation Endproducts‐Receptor for Advanced Glycation Endproducts
Arntl (Bmal1): Basic helix–loop–helix ARNT‐like protein 1 or aryl hydrocarbon receptor nuclear, translocator‐like protein 1
Bhlhe40 (Dec1): Class E basic helix–loop–helix protein 40
Bhlhe41 (Dec2): Class E basic helix–loop–helix protein 41
Ca4: Carbonic anhydrase 4
Cdkn2b (p15): Cyclin Dependent Kinase Inhibitor 2B
CiR: Circadian Rhythms
Cirbp: Cold‐Inducible RNA‐Binding Protein
Clock: Circadian locomoter output cycles protein kaput
Cor: Hydrocortisone
CUB: C1r/C1s, Uegf, Bmp1
CVA: Categorical dependent Variable Analysis
Dbp: D site of albumin promoter (albumin D‐box) binding protein
DEG: Differentially Expressed Gene
DHT: Dihydrotestosterone
DMSO: Dimethyl Sulfoxide
E2: Estradiol
ECM: Extracellular Matrix
ErbB: Receptor tyrosine‐protein kinase erbB
FDR: False Discovery Rate
Fkbp5: Peptidyl‐prolyl cis‐trans isomerase FKBP5
FPKM: Fragments Per Kilobase of transcript per Million mapped reads
Foxo3: Forkhead‐Box‐Protein O3
GH: Growth Hormone
GnRH: Gonadotropin‐Releasing Hormone
HC: Hierarchical Cluster
iF3: insert of Figure 3
Igf2bp2: Insulin‐like growth factor 2 mRNA‐binding protein 2
Klf9: Krueppel‐like factor 9
Klhl8: Kelch‐like protein 8
mTOR: mechanistic Target of Rapamycin
LNCaP: Lymph Node Carcinoma of the Prostate
LNS: Local Network STRING cluster
MAPK: Mitogen‐Activated Protein Kinase
ME: Module Eigengene
meanImp: mean Importance
Ncor1: Nuclear receptor corepressor 1
NF κB: Nuclear Factor ‘kappa‐light‐chain‐enhancer’ of activated B‐cells
Nfil3: Nuclear Factor Interleukin‐3‐regulated protein
Npas2: Neuronal PAS domain‐containing protein 2
NVA: Numerical dependent Variable Analysis
p53: Cellular tumor antigen p53
PCA: Principal Component Analysis
Per3: Period circadian protein homolog 3
PVCA: Principal Variance Component Analysis
Ric3: Resistance to Inhibitors of Cholinesterase 3
Sfpq: Splicing factor, proline‐ and glutamine‐rich
sH: small hub
Slc39a10: Zinc transporter ZIP10
Slc41a1: Solute carrier family 41 member 1
*t*‐SNE: *t*‐distributed Stochastic Neighbor Embedding
TSH: Thyroid‐Stimulating Hormone
TNF‐α: Tumor Necrosis Factor‐alpha
Top1: DNA topoisomerase 1
WGCNA: Weighted Gene Co‐expression Network Analysis

## 1 INTRODUCTION

Aging is controversially classified as a nondisease (Bulterijs et al., 2015). During the 11th International Classification of Diseases, the World Health Organization proposed including the term “old age” as a diagnosis but withdrew it. Nevertheless, aging remains undisputed as a leading risk for degenerative diseases and death (Harman, 1991). A broad spectrum of age‐related disorders can be alleviated by targeting altered signaling pathways, including putative physiological rejuvenation (Rutledge et al., 2022). Consequently, even the US Food and Drug Administration addressed aging as a drug target through the Treating Aging with Metformin study. Recognizing universal age‐related changes is essential for identifying relevant mechanisms for potential interventions. The straightforward interpretation and great sensitivity of transcriptome analysis provide an opportunity for novel findings.

Comprehensive transcriptome analyses of various aging tissues have been realized both at the level of single cells (Tabula Muris, 2020) and whole structures (Schaum et al., 2020), revealing clusters of gene expression pathways for the extracellular matrix, mitochondrion, protein folding, stress response, signaling, and immune response by a statistical method (DESeq2). Each study has its dynamics and peculiarities, varying the number of samples between time points and sexes. The results might be biased, as gender affects aging (Wang et al., 2018). In part, there are no sampling intervals in midlife. Additionally, comparatively few biological replicates are used per sampling. The analyses are based on Black/6 mice, whose genetic background contributes to aging, demonstrated by considering caloric restriction, the first known method of extending life expectancy in mammals (McCay & Crowell, 1934). Forty‐one strains of mice extend or shorten their lifespan with almost equal frequency (Liao et al., 2010), with Black/6 extending its lifespan in contrast to DBA/2 with no positive effect of caloric restriction. Besides raising doubts about the general feasibility of extending lifespan through caloric restriction, this will cause biases in omics‐based age analyses that will likely overestimate biological changes or mask more relevant ones. Equality in the number of sexes, sampling events, and the inclusion of an additional mouse strain should diminish bias in an age analysis and promote more valid discoveries. A high data density can provide a more in‐depth analysis, uncovering previously undiscovered or less addressed biological functions of aging.

In recent years, machine learning has been used alongside statistical methods to identify biomarkers and predict biological age in complex data sets (Rutledge et al., 2022). Using iterative ensembles of decision trees, the variable selection method Boruta counts the instances in which multivariate trees result in a target variable prediction improvement compared to the same set of randomly sampled covariates (Degenhardt et al., 2019). Irrelevant features are thereby eliminated effectively, which in most filter‐based methods depends on the selected threshold value, partly set softly to obtain larger data quantities. Boruta considers multivariate nonlinear interactions, which might benefit omics data, as nonlinear interactions are more abundant than simple bivariate and independent associations (Olecka et al., 2024). The sole use of bivariate linear association tests could neglect the characterization of trajectories. It might also contravene the complexity of homeostasis, in which auto‐regulation emerges from integrating intricate pathways and feedback mechanisms.

Today, at the transcriptional level, inflammation, the immune system, energy metabolism (likely due to mitochondrial dysfunction), proteostasis, nutrient sensing (insulin, mTOR), and cellular senescence are frequently arising pathways interacting with lifespan (Rutledge et al., 2022), alongside other hallmarks of aging as genomic instability, telomere attrition, and epigenetic alterations (Lopez‐Otin et al., 2023). The best chronological marker remains the methylation pattern of the DNA (Olecka et al., 2024), which usually lacks specific biological processes regarding aging, so approaches such as those of the chromatin accessibility aging clock (Morandini et al., 2023) may be more useful.

It is hypothesized that studying different strains of mice with balanced sex ratios in multiple organs using a more comprehensive set of biological replicates will reveal more profound insights into the mechanisms of general aging.

## 2 RESULTS

### 2.1 Variations and commonalities among organ aging

**FIGURE 1:**
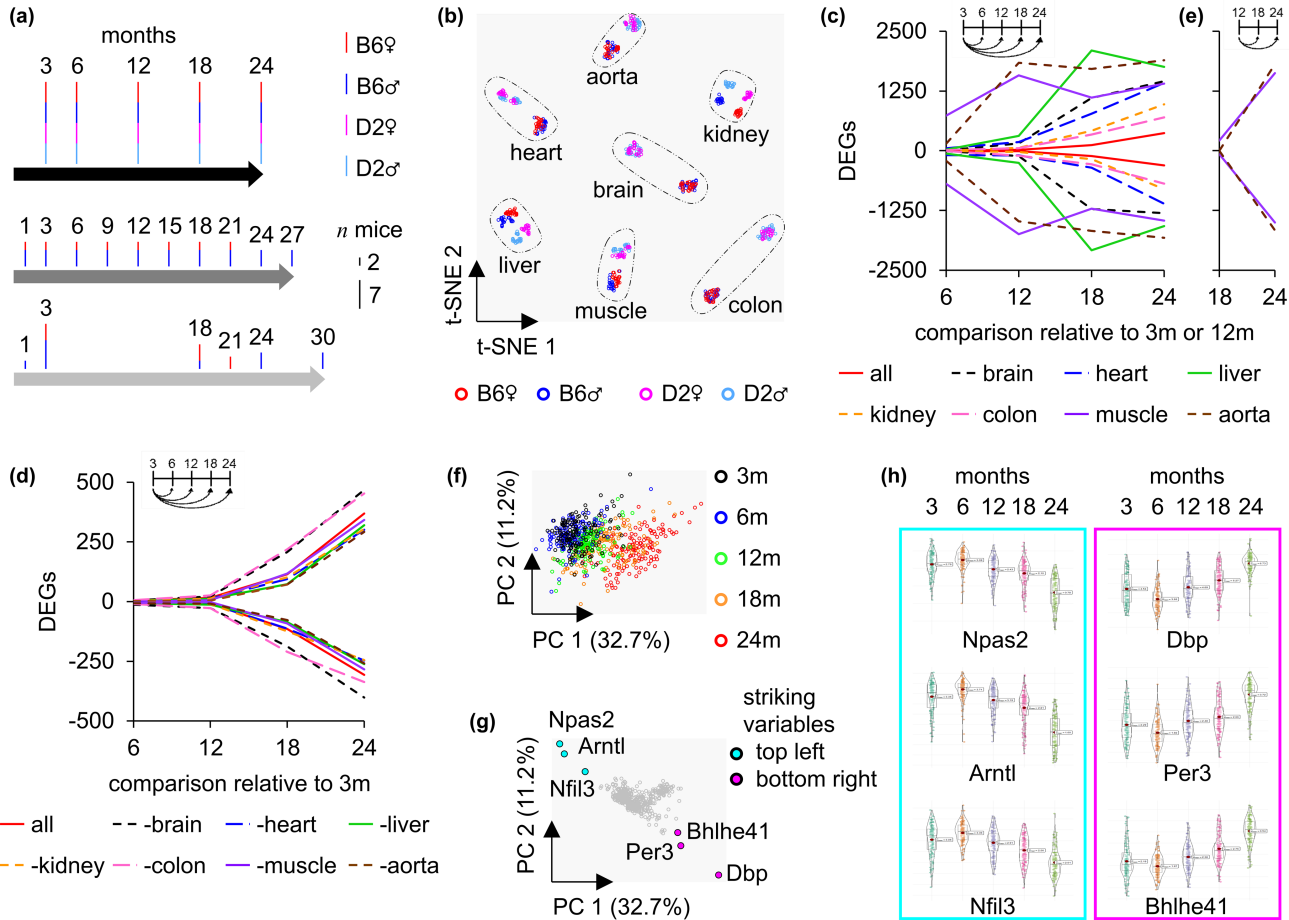
Aging alterations in and across organs. (a) The study design and the number of biological replications in black were compared to two existing approaches in dark (Schaum et al., 2020) and light gray (Tabula Muris, 2020). Black/6‐based (B6) or DBA/2 (D2) strains, female (♀) or male (♂), and the number of mice represented as the length of the line from the lowest (2) to the highest (7) distinct group. (b) *t*‐SNE for time‐independent characteristics. (c, e) Pairwise comparisons depicting the organ‐specific number of DEGs during aging in a line plot referring to 3 (3 m) as solid or 12 months (12 m) as dashed lines compared to later times. Inset: Scheme for pairwise comparisons for 3 m or 12 m. (d) Line plot displays the number of DEGs during common and distinct aging with respective organ exception (e.g., ‐brain: Brain is excluded from the joint analysis). (f) Unsupervised cluster for the 489 nonrejected features of the variable age using PCA across all samples (*n* = 700) with color‐coded timing. (g) The corresponding loading plot highlights six variables associated with aging. (h) Age‐dependent violin plots are used for all samples for Npas2, Arntl, Nfil3 and Dbp, Per3, and Bhlhe41. DEG, differentially expressed genes; t‐SNE, *t*‐distributed stochastic neighbor embedding.

To describe age changes during the lifespan of mice, the time points of 3, 6, 12, 18, and 24 months (m) of seven organs [brain (B), heart (H), liver (L), kidney (K), colon (C), muscle (M), and aorta (A)], in two mouse strains [C57BL/6N_Rj_ (B6) and DBA/2J_Rj_ (D2)], from both sexes [female (♀), male (♂)] are examined with five replicates each (20 samples per organ per time), resulting in a total of 700 samples. A comparison of our study with existing ones demonstrates the relatively extensive quantity of biological replicates used (Figure 1a). Cross‐tissue comparison is achieved on 11,830 transcripts (Table S1). The *t*‐distributed stochastic neighbor embedding (*t*‐SNE) characterizes similarities among samples, indicating separations strength between organs and mouse strains, while sex‐specific differences appear in the kidneys and liver but not in the brain and colon (Figure 1b). The variable for aging in the *t*‐SNE and principal component analysis (PCA) appears to exhibit predominantly continuous trajectories (Figure S1A), suggesting a substantial number of partly linear combinations to describe aging. Consequently, an assessment using principal variance component analysis (PVCA) (Li et al., 2009) can reveal organ‐specific diversity in the contribution of variance, indicating nonuniform aging processes (Figure S1B). It also shows a diverse spectrum in which the influence further varies by strain and sex, depending on the organ. In the kidney, the effect of sex is similarly pronounced as that of the strain. In contrast, the brain exhibits no distinct sex difference. For the specific insides of the individual organ aging progression, DESeq2 exposes differentially expressed genes (DEGs), attempting to reduce strain and sex variables. Unlike the other organs, the aging effects in the aorta and muscle seem more distinct in the early stages of life (Figure 1c). One tissue per test was excluded from the analysis of combined DEGs to assess the relatedness of organ aging. The more similar the process is, the less variance is included in the collective analysis, increasing the DEGs (positive and negative). More DEGs over time could indicate a more specific aging of organs, such as in the brain and colon (Figure 1d).

In comparison with the 3‐month‐old animals, it is noticeable that the amount of DEGs increases over time in most tissues and the combined analysis, except the aorta, muscle, and liver (Figure 1c). It might mislead to the conclusion that the aging process is finished sooner in these organs since the DEGs at later time points could be predominantly identical. The increase in DEGs in a 12‐month comparison proves a transformation of the transcriptome while aging (Figure 1e). The extent to which a two‐point analysis is suitable to determine candidates for the progression of aging is controversial, especially as the trajectory of this process remains unclear. Nevertheless, various comparative strategies for a two‐point analysis are feasible, although the results differ (Figure S1C).

Approaches beyond DESeq2 often require a correction to reduce nongeneral age effects due to unequal variance power. This is particularly evident as the aging variable exerts the most negligible impact, organ > residual > strain > sex > age (Figure S1D).

There is a tendency for irrelevant but pronounced factors such as organ and strain to overshadow the age effects; to elaborate on these as much as possible, an optimal correction order (Figure S1E) was determined using a zScore or ComBat (Johnson et al., 2007) an empirical Bayes method adjustment. It reveals the ComBat‐corrected data from consecutively fitted subgroups with the strongest (4.87%) age‐specific variance in PVCA (Figure S1F) and is hence used below (Table S2).

Boruta, a machine learning algorithm based on the random forest method that is robust to overfitting, is applied to identify predictive features for aging. A numerical‐dependent variable analysis (NVA) revealed 489 nonrejected features (4.13% of 11,830) in at least one of 30 process iterations (Table S3, Data S1). The chaperone Ric3 exhibits the highest association strength, the so‐called mean importance (meanImp), establishing it as the most predictive value for age. Ric3 promotes the efficient folding of nicotinic acetylcholine receptors and influences the maturation of a serotonin (5‐HT) receptor. However, the number of hits matched with their meanImp shows a separation between rejected and tentative features (Figure S1G), suggesting tentative attributes as relevant. Based on the aligned data (Table S2), the PVCA age variance of 489 hits is 21.3%. Forty ANOVA analyses with different filter parameters were used to compare the results with the strength of the Boruta selection. The top PVCA value of an ANONA analysis was 14.6% (278 hits), and the best PVCA value/hit ratio was 14.3% (570 hits). The predictive accuracy for age (numerical) was calculated to determine the limits of the Boruta selection, which increased from 0.544 (*ϰ*: 0.434) to 0.841 (*ϰ*: 0.801) when just the 489 selected features were used instead of all variables.

The PCA of the 489 hits demonstrates a pronounced age progression, mainly along the first component, indicating a solid representation of aging within the identified candidates (Figure 1f). The corresponding loading plot indicates the variables responsible for this progression (Figure 1g), with an accumulation of CiR genes lying along the top left (Npas2, Arntl, and Nfil3) and bottom right (Dbp, Per3, and Bhlhe41) of the two main PCs, indicating diagnostic relevance of these nonlinear parameters as aging predictors across organs (Figure 1h).

### 2.2 Patterns of collective organ aging

**FIGURE 2:**
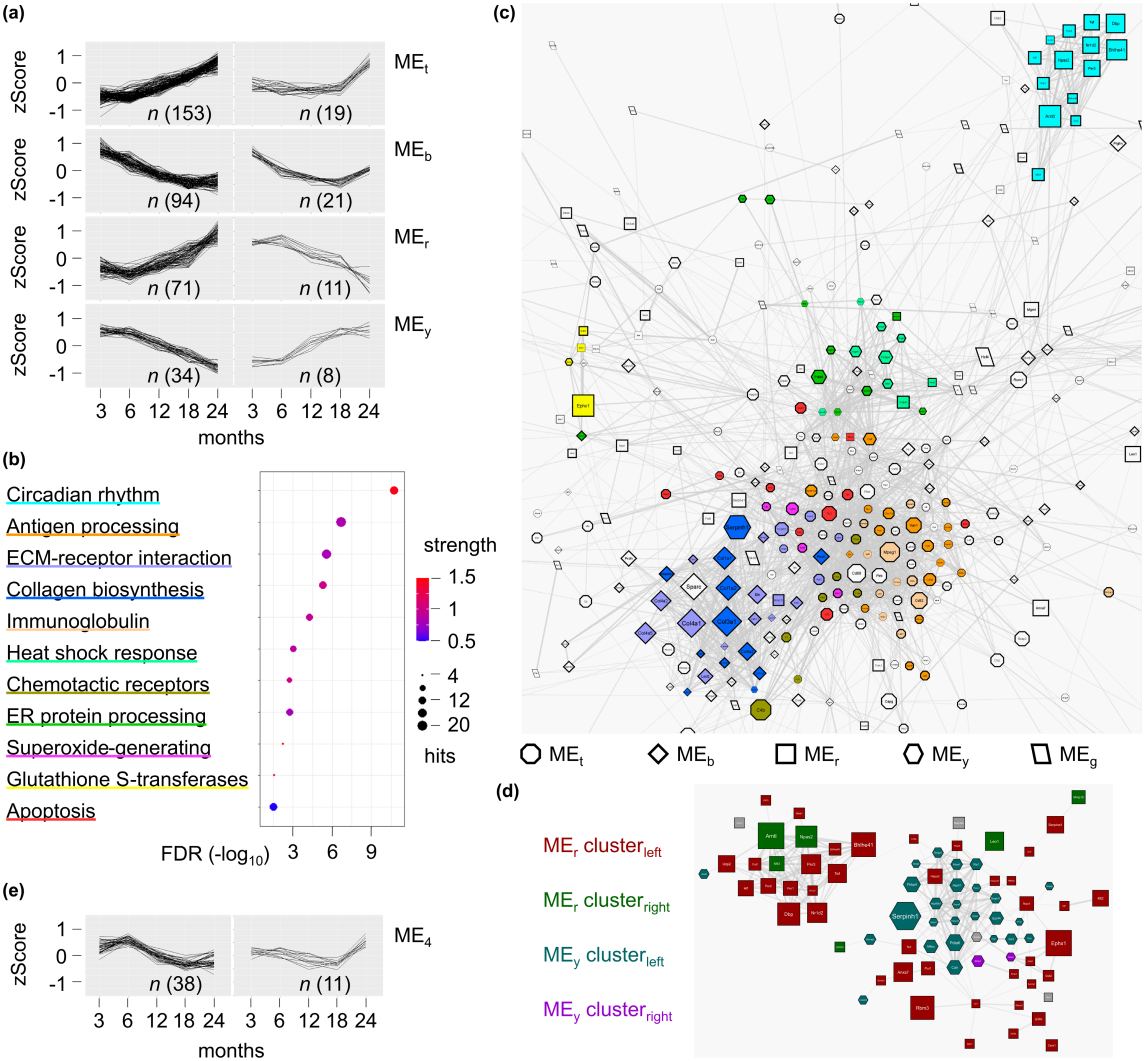
Analogous gene expression profiles of biological ontologies among collective organ aging. (a) zScore averaged line plot of the four informative module eigengene (MEs), which were additionally subdivided according to hierarchical cluster to reveal potential antiparallel expression behavior within the MEs. Amount of hits as *n*. (b) Term description of local network ontologies by strength, number of hits per pathway, and false discovery rate (FDR) enrichment. (c) The upper part of StringDB protein‐based network analysis with clusters according to WGCNA and color ontology mapping. The edge‐weighted spring‐embedded layout arrangement is based on the inverse interaction score (confidence from 0.4 to 1) and automatic overlap removal. The size of nodes is based on the iteration average of the numerical‐dependent variable analysis meanImp (Table S3). Colors are classified in figure (b). (d) The opposing expression profiles ME_r_ and ME_y_ are visualized, as in figure (a), with organic arrangements and automatic overlap removal. Primarily encompassing the circadian rhythms (CiR)‐proteostasis axis, clarifying the particular deregulation of the CiR feedback loop. (e) zScore averaged line plot of the unique ME_4_. Amount of hits as *n*.

In hierarchical cluster (HC) analysis, a general classification of the behavior of transcript clusters during aging remains complex, with simplification such as using the median for each age improving clarity (Figure S2A) but obscuring organ‐specific patterns (Figure S2B). Weighted gene co‐expression network analysis (WGCNA) groups similar features based on a correlation matrix to create a connected network that is as scale‐free as possible by flexibly adjusting the weights of edges (Langfelder & Horvath, 2008). By identifying the first principal component of a given module (cluster of highly linked genes), the so‐called module eigengene (ME) is generated. Among the 489 hits, the WGCNA analysis results in four informative MEs (Table S3). In the WGCNA, the network type is analyzed unsigned in this study, which means that antiparallel expression behavior can also be contained in an ME, which reveals their affiliation. To identify these opposing hits, the MEs were divided into smaller subgroups using HC (distance threshold: > 0.22), and the first (left) and second (right) most frequent groups per ME were visualized by line plots (Figure 2a).

Local network STRING clusters (LNSs) and KEGG pathways are identified (Table S4), whereby the 11 putative most relevant LNSs (Figure 2b) are mapped in a protein‐based interaction network (Figure 2c), all of which can be broadly categorized into one of the four MEs discovered. The CiR, glutathione‐associated (Gstt1, Gstt2, Gsta4), and a part of the proteostasis transcripts are in ME_r_. The bulk of proteostasis‐linked heat shock and endoplasmic reticulum protein processing transcripts are in ME_y_, the extracellular matrix protein, and collagen biosynthesis transcripts in ME_b_, the LNS annotations antigen processing, immunoglobulin, chemotactic receptors, superoxide‐generating activity, and apoptosis are in ME_t_. No distinct LNS appears to be prevalent in the residual ME_g_. The most substantial meanImp aging values are identified mainly in ME_b_ and ME_r_. Antiparallel patterns within a ME can be observed in ME_r_ and ME_y_ (Figure 2a, right‐sided). If such patterns are mapped in the protein‐based interaction network instead of the LNS, it visualizes that the negative feedback loop of the CiR genes gets disturbed (Figure 2d).

A categorical‐dependent variable analysis (CVA) is implemented to identify progression‐independent alterations at distinct ages and identified 571 nonrejected features (Table S3), with fatty acid beta‐oxidation/biosynthesis as well as S‐100/ICaBP type calcium‐binding domain and annexin as additional LNS annotations (Table S4). The PVCA age variance of the 571 candidates is 18.9%. Predictive accuracy (categorical) increases from 0.683 (*ϰ*: 0.604) to 0.789 (*ϰ*: 0.736) when the 571 selected features are used instead of all variables.

WGCNA analysis provides five informative MEs whose compositions largely match NVA analysis, although a novel pattern (ME_4_) also emerged (Figure 2e) where no LNS could be determined. Biological processes such as steroid hormone receptor signaling and KEGG pathways like endocrine resistance, longevity, thyroid signaling, apoptosis, or regulating pluripotency can instead be classified based on a limited number of hits, which includes biological rhythms candidates as Clock, Ncor1, Klf9, Top1, and Sfpq (Table S5). Across the StringDB network, the hits of ME_4_ seem to be accumulated nearby candidates of the ME_3_ (similar ME_r_) and ME_5_ (similar ME_y_) (Figure S3). A small hub (sH) between the predominant clusters seems notable, where the hits are related to the Foxo signaling pathway, cell cycle, and publications on self‐renewal, epigenetics, and maintaining stem cell function (Table S6). The average number of correlations within ME_4_ (0.1791) is the third highest after ME_5_ (0.1932) and ME_1_ (0.1931). Adjusted for the number of genes, ME_4_ is second after ME_5_, indicating a compact network.

Core clock transcripts undergo only minor alterations with age in various tissues, while the output clock genes exhibit significant shifts (Wolff et al., 2023). Therefore, 18 genes considered relevant in the collective organ analysis were analyzed for impact strength within the organ‐specific transcriptome matrix. The CVA was used to include the Clock gene. No clear tendency regarding the meanImp between core and output genes seems given. The differences are rather gene‐specific, so Arntl, Bhlhe41, Hlf, and Tef seem relevant as they are associations in at least five tissues (Figure S4).

The characteristics of the predominant clusters (Figure 2a,e) frequently turn around after the sixth month of life. The fundamental change in expression could indicate a biological aging process that begins after the peak of sexual maturity is achieved.

### 2.3 Interdependencies between age‐related features

**FIGURE 3:**
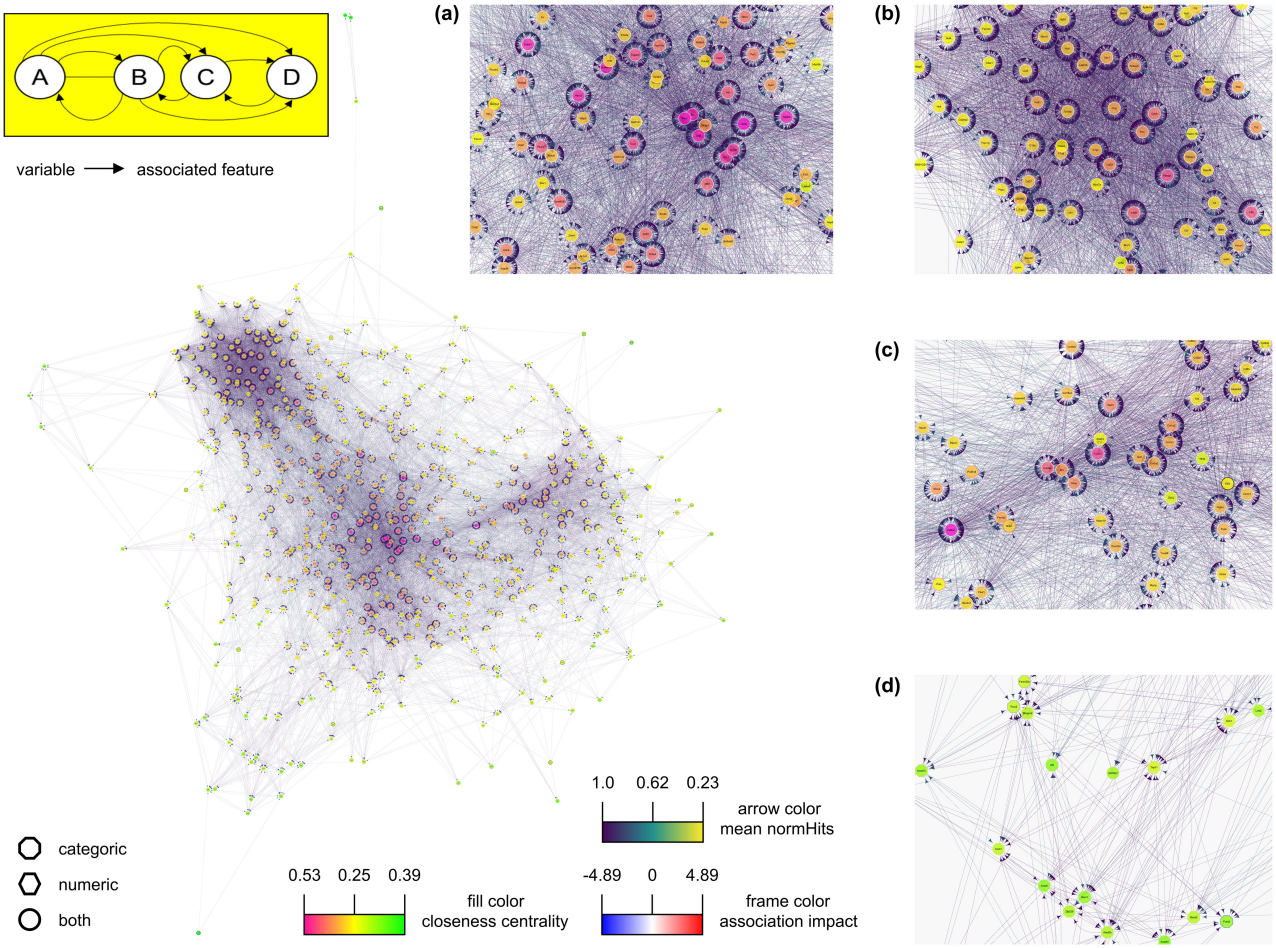
The transcript interaction network of aging, created based on the CrossBoruta results. (a) Center, (b) upper left, (c) right, and (d) lower left accumulation. The shape indicates whether the hit is detected numerically, categorically, or both. The arrow directs from a target variable to its correlated feature. The arrow color matches the normHits' iteration average value (the darker the arrow color, the more confident the feature relation). An edge‐weighted spring‐embedded layout creates the arrangement of nodes based on the inverse meanImp iteration average value (inverse arrow length, the closer, the stronger feature associated). Nodes are colored according to closeness centrality. The frame color depicts log_2_ asymmetric association strength (AAS), a ratio between the transcript dependence and its impact on the confirmed features (black, no value). Inset: Simplified network of transcripts (A–D). If a target variable has a feature that associates, an arrow points to this feature. The arrow length is inversely proportional to the strength of the association. The black line between A and B symbolizes a value of the simple bivariate correlation. Transcript A would have a high ASS value, and transcript D would have a low ASS value.

Previously, age was used as the target variable, and depending on the analysis, whether NVA (489) or CVA (571), a total of 645 hits were identified from both. Each of these 645 hits was analyzed separately as a target variable to determine gene–gene interactions. The feature selection results of this multiple machine learning search called CrossBoruta were summarized and yielded 7453 transcript associations. In this study, 631 of these 7453 hits belong to the 645 age‐associated genes. To obtain an aging network, only the 16,347 interactions (edges) between the 645 hits (nodes) are considered (Table S7). These data were used to visualize the network, where the distance between nodes is inversely proportional to association strength (meanImp) between the gene–gene interactions of the CrossBoruta outcome. Genes that interact more strongly are located closer together in the network, resulting in four areas of accumulation (Figure 3, Data S2).

The most connected hits above the 90% quantile of closeness centrality with a threshold ≥0.4469 (Table S8) are located in the network center (Figure 3a) and primarily associated with biological rhythms and stress response/chaperon as keywords (Table S9). Further accumulations are associated with immunity and inflammation (Figure 3b), the ECM proteins/receptors, and their mediated signaling or interaction partners (Figure 3c), involved in cell growth/morphology. Transcripts of the least defined aggregation affect energy metabolism, RNA regulation, and DNA binding (Figure 3d).

To determine those players of the aging network that might have a substantial influence, nonrejected target variables are summed in a transcript‐specific manner (Table S10). About half (16 hits) of the ~5% strongest candidates are assigned to ME_4_, indicating a relevant contribution of this cluster, whose keywords are the nucleus, methylation, DNA‐binding, stress response, and biological rhythms (Table S10).

The usual bivariate correlation generates only one value between the interaction partners (insert Figure 3 (iF3), the black line between A and B). For the feature selection of each transcript with Boruta, the link attributes are also determined separately for each of these analyzed variables, whereby the interaction partners, their number, and the respective strength and probability of the association are unique. For instance, the feature B of the analyzed target variable A (iF3, an arrow from A to B) can associate more strongly with A than in the opposite direction, in which the target variable B was analyzed and its feature A associates less strongly with B (iF3, a longer arrow from B to A). Occasionally, only unilateral interactions appear relevant (iF3, no arrow from C to A). Consequently, an asymmetric association strength (AAS) between transcripts appears. To visualize the ASS of each transcript, the sum of all associations of a feature *i* is divided by the sum of all associations of the same target variable *i*, represented by frame color (Figure 3, Data S2, Table S11). Suppose a transcript is only associated with a few others (iF3, A only with B), but many are associated with it (iF3, B–D with A); then the AAS is above 1 (positive, if logarithmized as in Figure 3) and possibly indicates a hierarchically superior actor. Since transcripts of the CiR reveal some of the highest alterations (meanImp) during aging, are oppositely modulated, and are also of high centrality, it is promising to identify candidates that associate weaker with the central CiR transcripts than vice versa (iF3, as for A). For instance, the insulin‐like growth factor 2 mRNA‐binding protein 2 (Igf2bp2) might act as a superior regulator. Deletion of Igf2bp2 mimics age‐related changes in young mice (Suo et al., 2022), possibly through N6‐methyladenosine and their methyltransferases, known to be involved in the circadian clock (Wei, 2021). However, the hitherto barely characterized 0610009B22Rik, Klhl8, Slc41a1, and Slc39a10 received even more pronounced AAS (log_2_ > 3) and could be of particular interest concerning the potential control of CiR.

### 2.4 Feature hierarchy to determine influencing factors

The aim is to uncover (superordinate) mechanisms of promising age‐altered candidates outside the aging network. Therefore, all (11,830) transcripts are computed as target variables (CrossBoruta), similar to the approach used to create the aging network (645 hits) but more extensive. Target variables with any of the 645 hits as a selected feature are sought. In this way, it is possible to determine which transcripts have the same selected feature as aging.

Selected criteria for the CiR and further promising aims are compiled into groups (Table 1). This approach can be applied to only a few selected transcripts as there are 9523 target variables with 64,420 interactions (Table S15) for all 645 hits of the age network, which complicates an interpretation without further segmentation. Among others, diurnal hormone signaling (GH, GnRH, steroids, and thyroid), cell cycle, metabolism, senescence, and repair/damage mechanisms seem crucial (Table 1). Also, Cirbp was identified as remarkable as it is clearly the most frequently (450) confirmed feature (Table S15) of the 645 aging‐network hits and also links CiR to proteostasis (Figure 2c,d). Cirbp is also a case of the strong intertwining within proteostasis, which complicates the determination of its external factors. The protein is rhythmically expressed and regulates CiR genes (Hoekstra et al., 2019). In parallel with regulating cellular stress or bypassing replicative senescence (Artero‐Castro et al., 2009), Cirbp appears to be a critical factor in the genomic stability of the bowhead whale, where it may be essential for the longevity and cancer‐free lifespan of the longest‐lived mammal (Firsanov et al., 2023).

**TABLE 1:** Factors modulating aging features.

Criteria	Feature of *X* dependent variables	Source	Selection of functions as KEGG pathways
AAS with log_2_ > 3 to CiR hits (4 hits, Table S11)	(nonrejected) 651	Table S12	GH synthesis, secretion, and action Protein export GnRH signaling pathway Progesterone‐mediated oocyte maturation
CiR hits of closeness centrality >0.47 (11 hits, Table S8)	(nonrejected) 1666	Table S13	Metabolic pathways Cellular senescence MAPK signaling pathway
ME_4_ hits (72 hits, Table S3)	(confirmed) 3381	Table S14	Pathways in cancer ErbB signaling pathway Thyroid hormone signaling pathway Focal adhesion
sH hits of ME_4_ (9 hits, Figure S3)	(nonrejected) 2371		Pathways in cancer Cell cycle Proteoglycans in cancer AGE‐RAGE signaling pathway
	(confirmed) 774		AGE‐RAGE signaling pathway PFAM: Methyl‐CpG binding domain

### 2.5 Hormonal influence on aging transcripts

**FIGURE 4:**
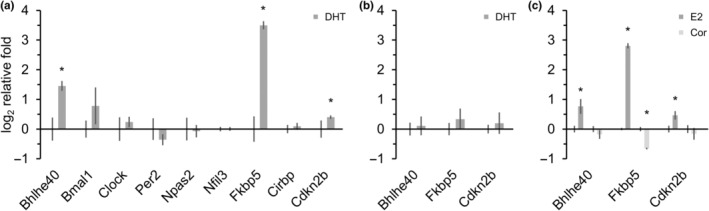
Steroids affect specific age‐associated targets. LNCaP with (a) 30 nM dihydrotestosterone (DHT) for 24 h (*n*_CTL&DHT_ = 6), (b) followed by a 72 h recovery (*n*_CTL&DHT_ = 6), (c) or 30 nM estradiol (E2), respectively 300 nM hydrocortisone (Cor) for 24 h (*n*_CTL&Cor_ = 6, *n*_E2_ = 5). The relative log_2_ changes, normalized to one compared to the tested stable reference gene Hprt1, are plotted for specific genes of interest. For each candidate, the dimethyl sulfoxide (DMSO) control is shown on the left, and the treatment is on the right. Significance is marked with an asterisk (*p* > 0.05).

Since this in silico study assumes that different hormone classes (such as GH, thyroid, or sex hormones) influence the age‐modulated transcripts, their effects on hormone‐sensitive cells were examined in part. In LNCaP cells, a triiodothyronine‐dependent increase in Bhlhe40 was identified and reported to induce cellular senescence via an unconventional p15‐associated (Cdkn2b) pathway (Kotolloshi et al., 2020). This p15‐associated cellular senescence also appears relevant for androgen‐induced senescence (Mirzakhani et al., 2022), where Fkbp5 was strongly upregulated. Bhlhe40 and Fkbp5 are among the most tightly regulated candidates of the CiR‐proteostasis axis, modulated by age (Figure 2c,d). Since steroids were not tested for CiR transcripts, LNCaPs were incubated for 24 h with supraphysiological senescence‐inducing androgen levels (dihydrotestosterone, DHT). They exhibited a significantly increased expression of Bhlhe40 (175%), Fkbp5 (1030%), and Cdkn2b (32%), while additional CiR transcripts seem unaffected (Figure 4a). After a recovery period of 3 days, the cells showed no permanent upregulation (Figure 4b). Further, estrogens (estradiol, E2) also significantly increase the expression of Bhlhe40 (70%), Fkbp5 (599%), and Cdkn2b (38%), whereas the non‐sexual steroid (hydrocortisone, Cor) does not (Figure 4c) and instead reduces Fkbp5 (58%).

## 3 DISCUSSION

The order of variances in an age cohort, starting with residual (61.3%), tissue (36.3%), sex (1.1%), and age (0.3%), has already been described (Schaum et al., 2020). However, the proportions in the data set of this study varied by organ (87.3%), residual (5.75%), mouse strain (2.74%), sex (0.31%), and age (0.26%). The unexpectedly substantial variance of the mouse strain was novel. Since many variances were assigned, the remaining residual values should have negatively impacted the subsequent age analysis less and helped reveal hidden correlations more effectively.

The mentioned study speculated about a possible underestimated contribution of CiR to health deterioration based on gene ontology annotation of a few transcripts among the top 30 DEGs (Schaum et al., 2020). Furthermore, the number of rhythmically expressed genes decreases with age, reflecting an essential hallmark (Wolff et al., 2023). Due to their extent and centrality, our data clearly demonstrate that CiR genes are unexpectedly substantially involved in the transcriptome of aging compared to various biological functions, that CiR genes are indeed up‐ and down‐regulated and, in particular, are closely linked to the regulation of glutathione associations and the proteostasis.

Altered biological functions during aging can often be categorized in patterns of expression, most of which are nonlinear, suggesting that full maturation can be assumed around the sixth month, as many transcriptional inversions at this stage indicate. The profile of the ME_4_ cluster is characterized by a pronounced peak during presumed adolescence, followed by a primary decline in expression by the 12th month of life, strikingly early and abrupt within the lifespan. Although associated with steroid hormone receptors, endocrine resistance, longevity, thyroid signaling, apoptosis, or regulation of pluripotency, but also including CiR genes such as Clock. A similar whole organism trajectory in another age cohort (cluster 7), a divergent result shows protein folding to be the most functionally enriched pathway (Schaum et al., 2020), with a possible interaction to be explored. Unlike other clusters, most ME_4_ candidates seem unconnected at the protein level, except for the sH comprising relevant DNA (Dnmt3a), histone methylation (Ezh2), and longevity factors such as Foxo3. Hits were also involved in thyroid hormone signaling as the thyroid‐stimulating hormone (TSH), a pathway impaired, for example, in long‐lived Ames‐Dwarf mice (Brown‐Borg et al., 1996). Due to the suspected influence on the network that changes with age, the interaction and effects of the ME_4_ genes should be investigated. The Ames‐Dwarf mice revealed radically diminished amounts of prolactin and growth hormone (GH) (Brown‐Borg et al., 1996). The undeniable importance of GH in aging was demonstrated by the GH receptor knockout (GHRKO) mice, winner of the Methuselah Prize for the world's longest‐lived laboratory mouse. Notably, the GH‐associated genes, among others, potentially influence the genes with the highest AAS, which might be superior to the CiR genes. The better‐characterized Igf2bp2 also exhibits increased ASS and may provide a link between CiR proteostasis and GH that is better suited for investigation, as members of the IGF family are strongly associated. Little is known about the influence of hormones of several classes on CiR or proteostasis. Ames‐Dwarf mice are Prop1‐impaired, as a result of which, in addition to TSH and GH, for example, the luteinizing hormone and, thus, the sex hormones are also reduced. Crossing Ames‐Dwarf mice with an Alzheimer's disease model resulted in reduced Aβ plaque deposition and Aβ 1–40 and Aβ 1–42 concentrations (Puig et al., 2016), indicating a relevant impact of hormones on proteostasis. Growth, thyroid, and sex hormones are involved in the regulation of glutathione S‐transferases (Coecke et al., 2000), as suggested via the CiR‐proteostasis axis.

Candidates upstream of CiR are elusive; however, associations with some diurnal cycle‐dependent hormones (Ikegami et al., 2019) emerge as possible modulators, suggesting that cell‐external parameters may be involved in cellular aging. To this end, it might be crucial to determine to what extent delayed maturation, as observed in dwarf mice, affects lifespan by modulating the CiR‐proteostasis axis and to what extent hormones contribute. Indeed, thyroid gland hormones, such as triiodothyronine, trigger DNA damage and induce senescence p16‐independent (Zambrano et al., 2014) but can also modulate CiR genes, such as Bhlhe40, responsible for p15‐dependent cellular senescence (Kotolloshi et al., 2020). The cell culture experiments suggest a short‐term effect of steroids on the CiR gene Bhlhe40, although more accurate recording by multiple temporal capture or real‐time detection (reporter genes such as luciferase) could reveal missed effects of additional clock genes, as shown for dexamethasone (corticosteroid), causing a wave‐like expression (Kiessling et al., 2017). Steroids also affect numerous players in the CiR network in animals (Zhao et al., 2018) and humans (Johnson et al., 2022) when permanently elevated. Androgens can also trigger p15‐senescence (Mirzakhani et al., 2022), and both DHT and E2 cause overexpression of Bhlhe40. The senescence triggered by sex hormones may be regulated using this CiR gene. The long‐term steroid increase, even in a physiological range, can cause alterations in the CiR in humans, such as an increase in Bhlhe40 (Dec1) or a reduction in Arntl (Bmal1) (Johnson et al., 2022). Might the unavoidable hormonal stimulation during lifetime deregulate the CiR to such an extent that senescence occurs? In any case, the influence of hormones on the CiR‐proteostasis axis needs to be clarified. Understanding whether the CiR proteostasis axis might be affected in species that undergo behavioral and lifespan changes due to steroids might be helpful (Schmidt et al., 2006; Wang et al., 2022).

Regardless of how a change in CiR may lead to senescence, it has been proven that manipulating CiR genes contributes to this process. Knockouts of Arntl or Clock lead to premature aging and shortened life expectancy in mice (Dubrovsky et al., 2010; Khapre et al., 2011). The early onset of cellular senescence in the Arntl knockout animals is remarkable (Khapre et al., 2011), forcing the idea of a direct link to the aging mechanism. In Drosophila, it was confirmed that network alterations reveal diametric effects on lifespan since deleting the hetero‐dimer Per or Tim results in extension and in contrast, the opposite occurs with Clock or Cycle (Ulgherait et al., 2020). Interestingly, the Clock gene can also contribute to rejuvenating stem cells and (cartilage) regeneration (Liang et al., 2021). However, CiR transcripts are altered during life‐prolonging calorie restriction (Patel et al., 2016) and appear to reverse the aging transcriptome through its reprogramming (Sato et al., 2017), raising the question of the degree to which food restriction or the thereby often‐used feeding interval and the putative calibration of the CiR affects aging.

Daily oscillating cytosine modifications expressively overlap with age‐related epigenetic DNA changes (Oh et al., 2018), and the loss of epigenetic information is considered a cause of aging (Yang et al., 2023). Imperfect restoration of CiR gene methylation could contribute to a gradual breakdown of the network and, thus, the progression of aging. Since Ames‐Dwarf mice exhibit age‐related hypo‐ and hypermethylation (Cole et al., 2017), examining the correlation with the daily oscillation seems promising.

Changes in cyclical processes during aging were reported early on. In 1851, for instance, a change in the amplitude of rhythmic body temperature in humans with increasing age was reported, while in 1912, a more dampened and fragmented day‐night activity rhythm was demonstrated in rats with progressing age (Weinert, 2000). It would be interesting to determine whether the qualitative improvement in nocturnal sleep achieved by orexin blockers (Roecker & Coleman, 2008) also increases life expectancy.

The study faces several limitations. The most influential factors include the study design and the choice of analysis methods. A closer look at between intermediate ages, such as 6–18 months, may provide more relevant information on the development of the process. The comparison between the youngest and oldest animals can reveal numerous differences but may seem less meaningful as far as the process is concerned since the gene expression profiles reveal abnormalities in the trajectory, especially for the youngest and oldest. It suggests aging only after the growth phase toward adulthood and that old age adds effects that may distort the analysis, such as chronic inflammation.

As the Boruta settings (number of decision trees, iterations, etc.), the method used to align the data concerning tissue, strain, and sex differences will significantly affect feature detection. They resulted in various candidates indicating different pathways or functions in the GO analysis at lower enrichment levels (FDR). However, the CiR and proteostasis are among the most highlighted age changes in the statistical filter‐based ANOVA. Machine learning algorithms, such as the Elastic net (98.94%), seem to perform slightly better than Boruta (96.19%) in a homogeneous dataset measured by the area under the receiver operator characteristic curve, but Boruta selected 2.3 (1090/473) times more features (Zhang et al., 2021). If data are not perfectly homogeneous, the random forest algorithm may have an advantage for higher predictor reliability (Zhang et al., 2021). While evaluating six variable selection methods to find a small subset of essential variables with an optimal prediction performance, Boruta was the most powerful approach in the two used simulation studies. However, the Vita algorithm revealed slightly better stability in variable selection than Boruta. In a pure null model, where no predictor variables are associated with the outcome, Vita was also the most robust approach (Degenhardt et al., 2019). The consistency and stability of the selection seem to depend on the effect size of the predictor variables, and many overlapping variables are likely to be identified if many predictor variables are strongly associated with the outcome (Degenhardt et al., 2019). However, comparing several feature selection methods would identify additional relevant candidates.

According to the expression patterns, linear progressions across the lifespan appear atypical. The ratio of differentially regulated CiR genes could be informative in determining biological age after maturation. In contrast, standardization of diurnal transcripts will be challenging. The ratio of antagonistically regulated hits of ME_y_ may be a more promising age marker.

We hypothesize that CiR and its associated mechanisms have a more significant impact on aging than previously thought. Investigating modulated CiR from different perspectives of molecular machinery (e.g., rebalancing), cellular senescence, geriatrics, or pharmacotherapy may improve the quality and duration of life.

## 4 METHODS

Virgin mice (C57BL/6N & DBA/2J) with an age range of 5–7 weeks were provided by Janvier Labs and aged in IVC cages in our in‐house animal facility (ZMG), with a maximum occupancy of 4 females or 3 males, with males kept singly in case of incompatibility. Food and water were provided ad libitum. Housing conditions were 21°C, 55% humidity, and a 12‐h day‐night cycle. Usually, the animals were checked twice a week, daily, if there were problems. The organs were collected across the mouse's lifespan (Figure 1a, Table 2).

**TABLE 2:** Comparison of the sampling of (a) (Tabula Muris, 2020), (b) (Schaum et al., 2020), and (c) (this study). C57BL/6JN form Charles River (†‡), C57BL/6N (‡), and DBA/2 (•) by Janvier Labs; male (♂), female (♀).

*n* mice	Sex	Strain	Age (months)											Per sex	All
			1	3	6	9	12	15	18	21	24	27	30		
(a)	**♂**	†‡	2	7					2		4		4	19	30
	**♀**	†‡		4					4	3				11	
(b)	**♂**	†‡	4	4	4	4	4	4	4	4	4	4		40	56
	**♀**	†‡	2	2	2	2	2	2	2	2				16	
(c)	**♂**	‡		5	5		5		5		5			50	100
	**♂**	‡		5	5		5		5		5				
	**♀**	•		5	5		5		5		5			50	
	**♀**	•		5	5		5		5		5				

Final cervical dislocation (approved code: K2bM3) was applied before harvesting and freezing the tissue within 15 min. The heart, aorta, and colon were washed with 1x PBS. Total RNA was isolated by Invitrogen™ TRIzol™ reagent (#15596026) according to their standard protocol (~50–150 mg/mL), and samples were blended (Table S16). The purity ratios were approximately 2.0 A_260/280_ (−0.07, +0.12) and 2.2 A_260/230_ (−0.854, +0.21) (Table S16), mainly liver samples deviated with a lower A_260/230_ ratio, presumably because of glycogen. The RNA integrity number (Agilent 2100 Bioanalyzer) was ≥7 (for brain, heart, liver, kidney, colon, muscle: ≥7, ≥8, ≥9 for 7, 257, 336 samples | for aorta: ≥7, ≥8, ≥9 for 29, 68, 3 samples) (Table S16). The brain, heart, liver, kidney, colon, and muscle samples originated from the same animals. RNA was dissolved in 70 μL TE‐buffer (#AM9860) from Invitrogen™, and 30 μL (> 50 ng/μL) was sent to NovoGene Co, Ltd (UK) for RNA sequencing by eukaryotic strand‐specific transcriptome library, NEBNext® Ultra™, directional RNA Library Prep Kit, NovaSeq6000 PE150. NovoGene Co, Ltd (UK) standard pipeline (2019) with a GRCm38.p6 (GCA_000001635.8) assembly. Shortly, mouse genome mapping was performed using HISAT2 (2.0.5, default parameters). For quality control reads with adaptor contamination, reads if unsafe nucleotides account for more than 10% of a read (*N* > 10%), and reads if low‐quality nucleotides (base quality less than 20) account for more than 50% of the read were discarded. HTSeq (0.6.1, m union) was used for quantification. The software used (Table S17) and the data analysis procedures (Data S3) are described.

A data frame with organ, strain, sex, age, and 11,830 candidates was used for Boruta (Table S2). The mean of meanImp and normHits from the iterations was used for calculation. The computations of all (11,830) transcripts as target variables were performed on the UNIX‐based High‐Performance Computing IANVS of the University Halle (Saale) by R (4.2.2), an intel‐compiler (2019.1.144) and Boruta (8.0.0). Despite the same data input, the results of similar variables, as in the aging network, differ slightly from the results, likely due to the different versions. Code to compute Boruta (Data S4) and Tables S10 and S11 (Data S5).

For cell culture, LNCaPs (lymph node carcinoma of the prostate) were seeded in 60 mm plates with a density of 5000 cells/cm^2^ 48 h before treatment. As medium RPMI 1640 (no phenol red, Gibco, #11835030) was used with 1% penicillin–streptomycin (10.000 U/m, Gibco, #15140122), 1% sodium pyruvate (100 mM, Gibco, #11360039), 1% l‐glutamin (200 mM, Gibco, #25030081), 10% fetal bovine serum (Capricorn Scientific, #FBS.12A, Lot.:CP22‐5255), and 25 mM HEPES (1 M, pH 7.3, C‐C‐pro, #Z‐23‐M) was used. Cells were harvested with 0.5 mL TRIzol, and RNA was isolated as prior. Approximately, 0.5 μg total RNA was used for the cDNA transcription (Maxima H Minus First Strand cDNA‐Synthese‐Kit, Thermo Scientific™, #K1682), both the oligo(dT)18‐ und the random‐hexamer‐primer were used. The qPCR was performed according to the protocol (GoTaq® qPCR and RT‐qPCR Systems, Promega, #A6002) and measured with the CFX Connect Real‐Time PCR Detection System (Bio‐Rad, #1855201). The calculation of the Cq‐values was according to the Bio‐Rad procedure. Metabion Primers (Table S18) were tested for an efficiency of 2 (±0.2) and one PCR product.

The ANOVA results (Table S19), the prediction accuracy (Data S6), and the network characteristics, as correlations within the MEs (Table S20) are presented for the limitation assessment. The soft‐threshold power for the WGCNA was chosen to obtain a network that is as scale‐free as possible (a value of 1 would be perfect) with the highest possible number of categorized transcripts within informative ME. The ME_grey_ (ME_g_ and ME_6_ in this study) is the only less informative ME, in which remaining transcripts not associated with any network are outsourced. The higher the power parameter, the lower the categorizability of the hits. A balance must be determined between a high scale‐free network index and a high categorizable amount of hits. A power parameter of 5 is the lowest possible value to achieve a scale‐free fit index greater than 0.94 and was selected as the threshold value (Table S20).

## AUTHOR CONTRIBUTIONS

P.R.W. and A.S. contributed to the conceptualization. P.R.W. was involved in data collection, analysis, and visualization. A‐I.G. advised on the application of Boruta. N.J.G. assisted in the creation of the codes. P.R.W. and A.S. were involved in project management. A.S. was engaged in the acquisition of funding. A.S. and S.G. provided the resources. P.R.W. wrote the original draft. All authors reviewed and edited.

## FUNDING INFORMATION

German Research Foundation grant (RTG 2155, ProMoAge, DGF). Wilhelm Roux program of the Medical Faculty, Martin‐Luther‐University Halle‐Wittenberg.

## CONFLICT OF INTEREST STATEMENT

The authors declare no competing interests.

## Supporting information


Figure S1.

Figure S2.

Figure S3.

Figure S4.

Table S1.

Table S2.

Table S3.

Table S4.

Table S5.

Table S6.

Table S7.

Table S8.

Table S9.

Table S10.

Table S11.

Table S12.

Table S13.

Table S14.

Table S15.

Table S16.

Table S17.

Table S18.

Table S19.

Table S20.

Data S1–S6.

## Data Availability

All data, including counts and FPKM, are available in the supplementary materials. Any other details will be provided upon reasonable request.
