# Supplementary material for: The circadian rhythm: A key variable in aging?
Source: Aging Cell. 2024 Jul 30;23(11):e14268. doi: 10.1111/acel.14268 (PMC11561671; doi:10.1111/acel.14268)
Supplement: Supplementary file 1 — Figure S1. [file ACEL-23-e14268-s013.zip › acel14268-sup-0002-FigureS1.docx]

Figure S1. Variance analysis for feature detection.
(A) Composition plot of age variances by *t*-SNE (left) and PCA (right). (B) Variance proportion per organ by PVCA. (C) Schemes for pairwise comparisons, the resulting DEGs (up and down), and the variance of age. Here, 676 of 783 DEGs (86.3%) have already been derived from the analysis with the most extreme difference (3 M:24 M), and therefore, relatively little information is available from the course. In the consecutive comparison, age variance is relatively high (3.60%). Still, most data are derived from a single comparison (18:24 M). When comparing all of them with each other, there are a pleasing number of candidates (1475) but with a correspondingly poor representation of the variance of aging (1.78%), which is unfavorable. (D) Variance proportion for all features using PVCA. (E) Data adjustment procedure (c: correction; s: splitting; m: merging). (F) The proportion of age variables of the data set per processing method acquired by PVCA. (G) The plot of quantitative mean importance (0.1 increments) of all features according to the NVA decisions (rej: rejected; ten: tentative; con: confirmed). DEG, differentially expressed genes; NVA, numerical-dependent variable analysis; PVCA, principal variance component analysis; *t*-SNE, *t*-distributed stochastic neighbor embedding
