# Supplementary figures and images for: The circadian rhythm: A key variable in aging? (part 1 of 3)
Source: Aging Cell. 2024 Jul 30;23(11):e14268. doi: 10.1111/acel.14268 (PMC11561671; doi:10.1111/acel.14268)

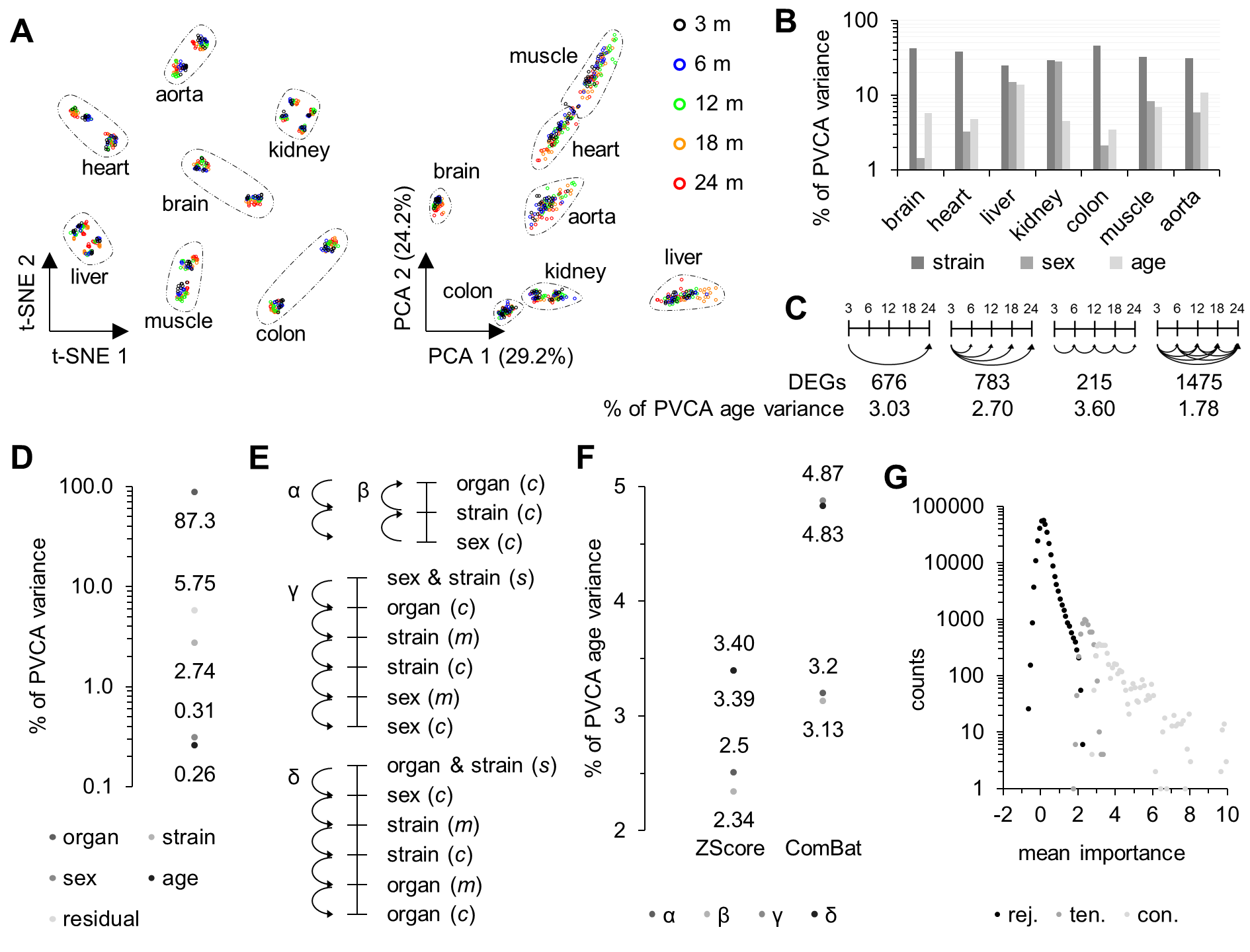

Supplement: Supplementary file 1 — Figure S1. [file ACEL-23-e14268-s013.zip › acel14268-sup-0001-FigureS1.png]

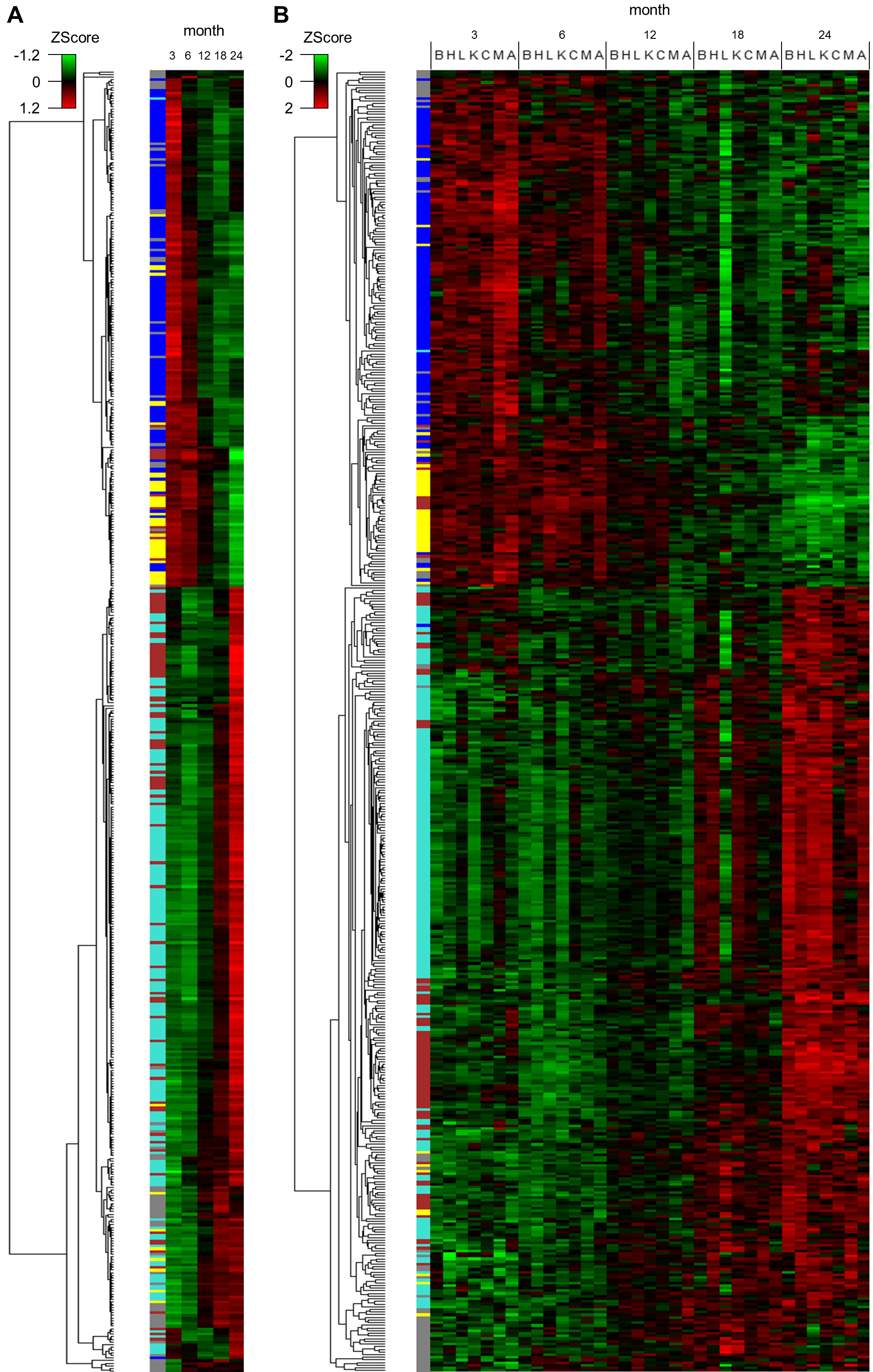

Supplement: Supplementary file 2 — Figure S2. [file ACEL-23-e14268-s015.zip › acel14268-sup-0002-FigureS2.png]

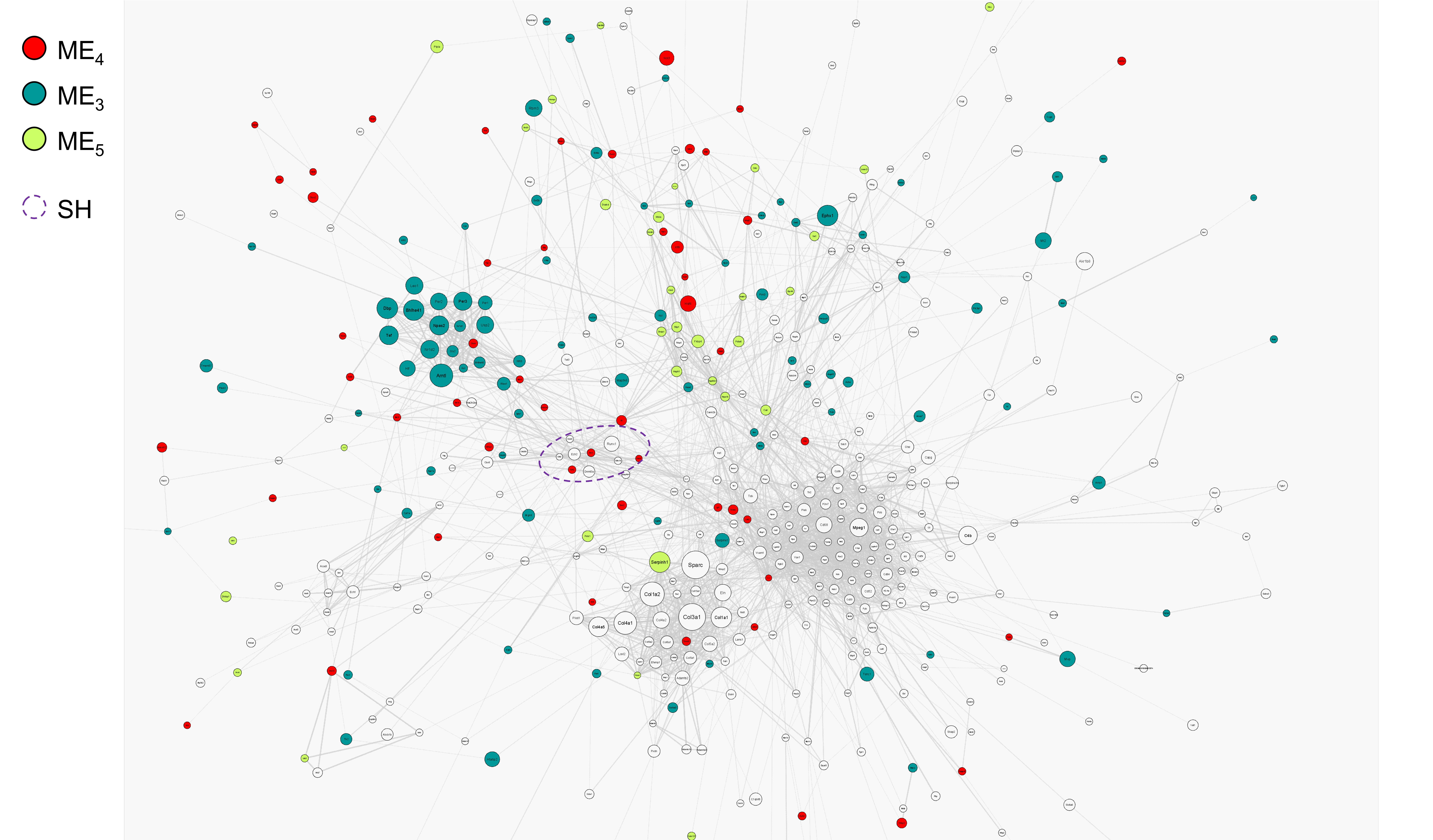

Supplement: Supplementary file 3 — Figure S3. [file ACEL-23-e14268-s021.zip › acel14268-sup-0003-FigureS3.png]

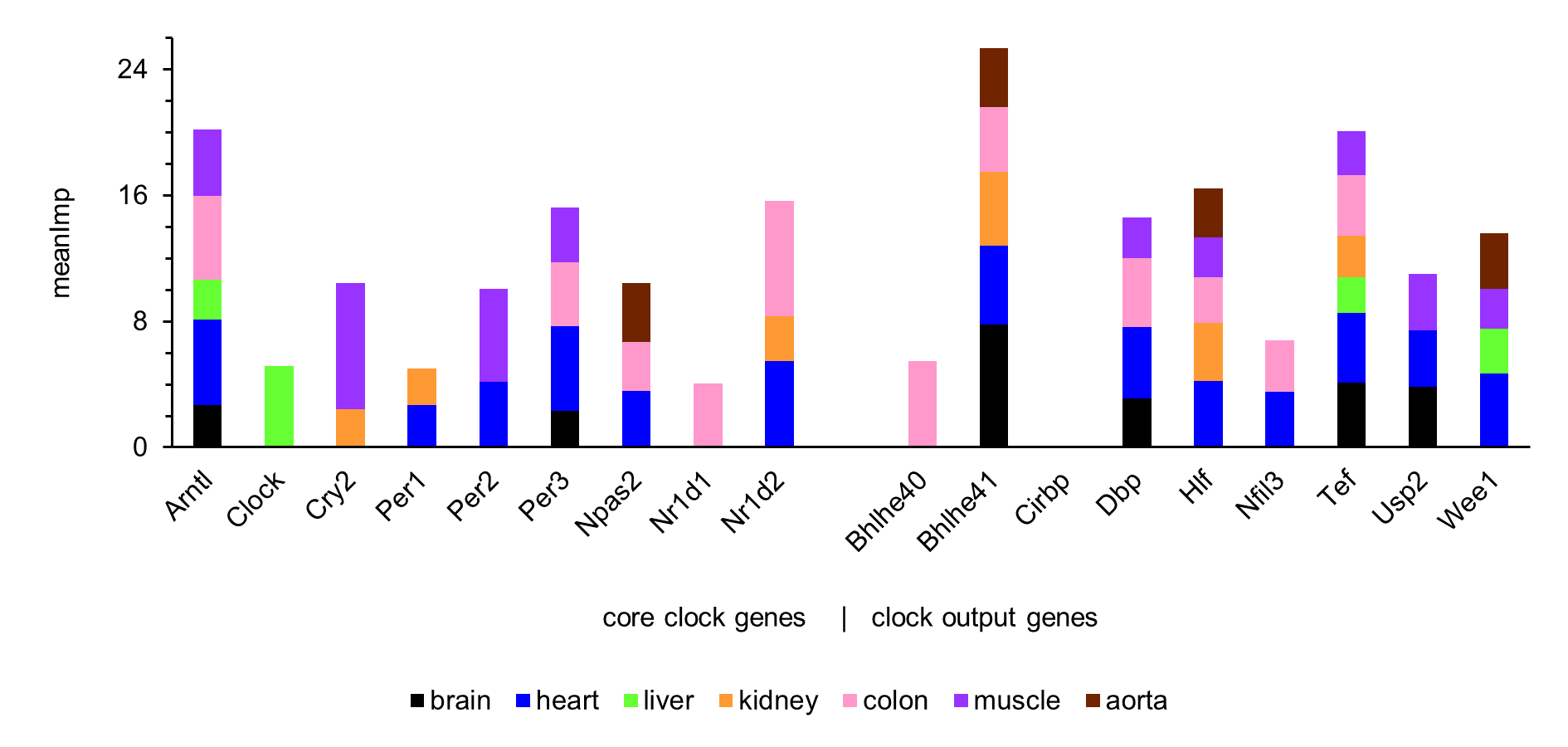

Supplement: Supplementary file 4 — Figure S4. [file ACEL-23-e14268-s003.zip › acel14268-sup-0004-FigureS4.png]

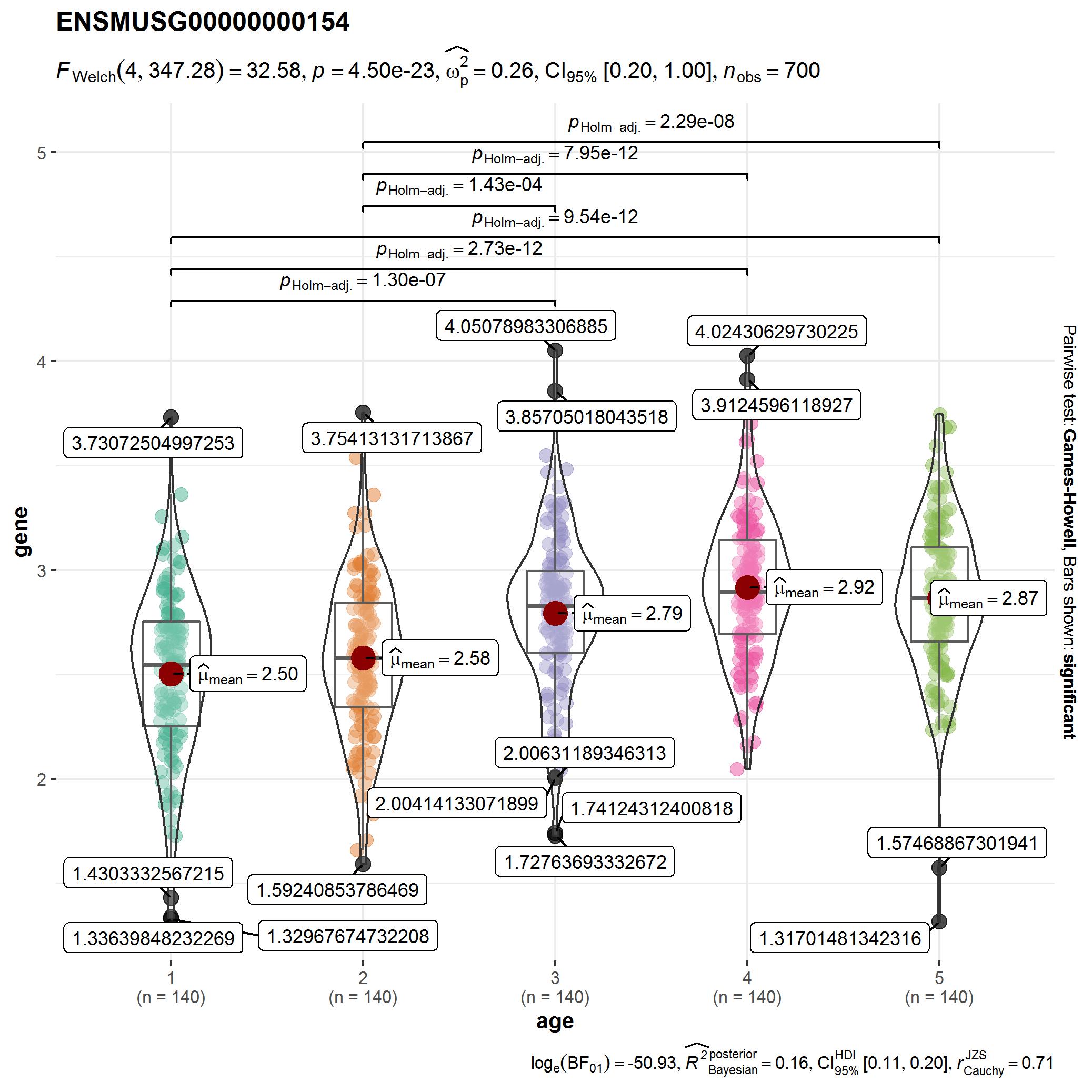

Supplement: Supplementary file 25 — Data S1–S6. [file ACEL-23-e14268-s017.zip › Data S1/ENSMUSG00000000154.jpeg]

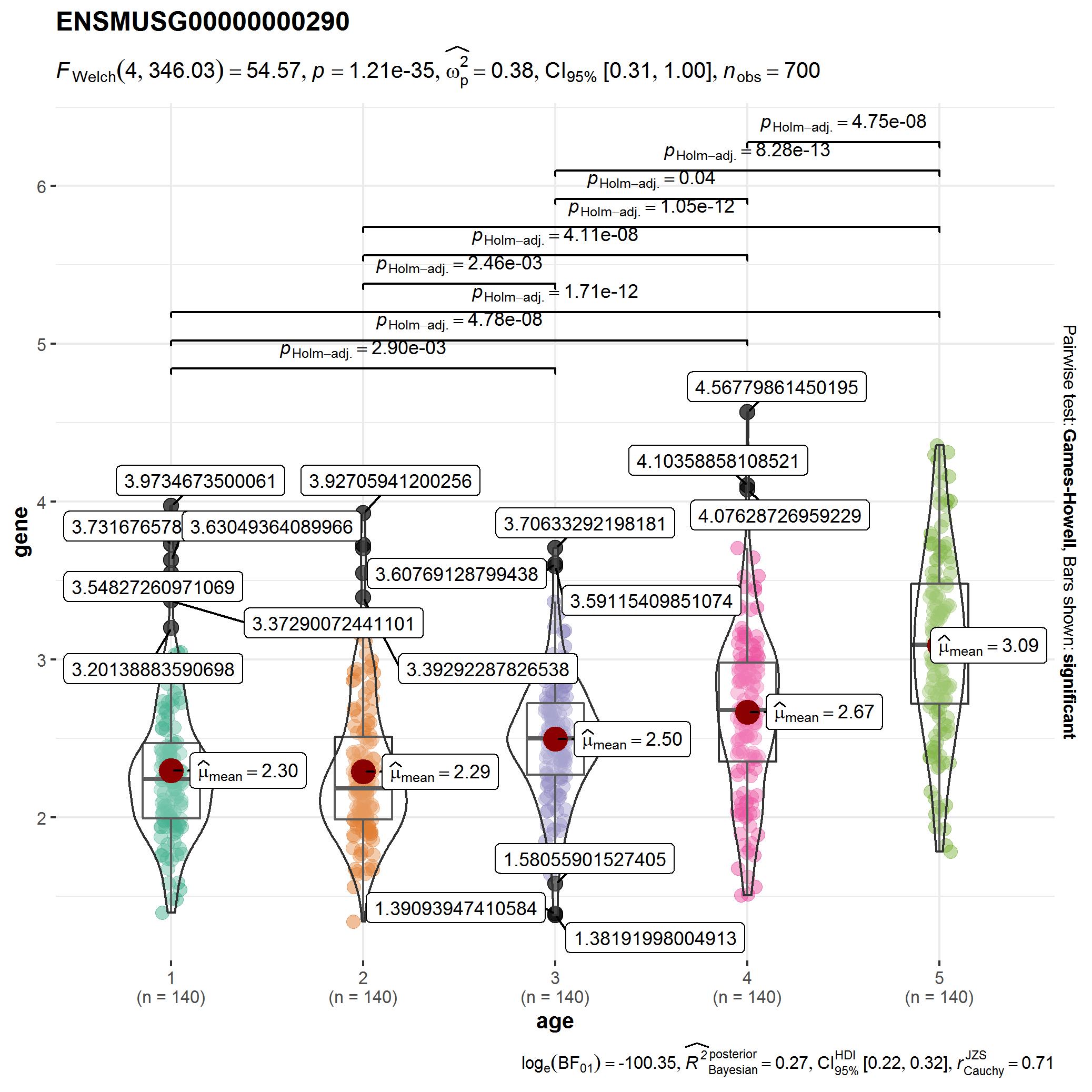

Supplement: Supplementary file 25 — Data S1–S6. [file ACEL-23-e14268-s017.zip › Data S1/ENSMUSG00000000290.jpeg]

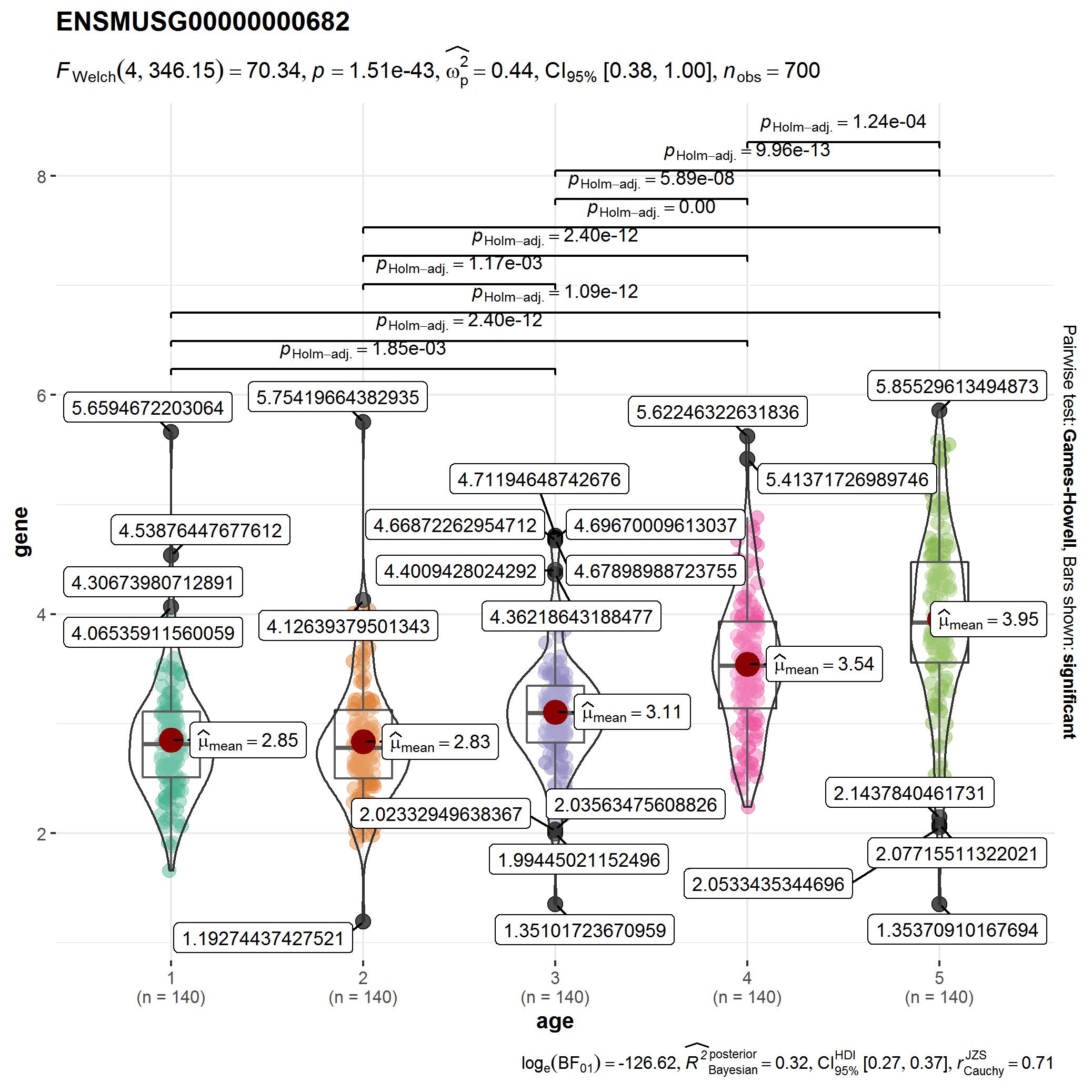

Supplement: Supplementary file 25 — Data S1–S6. [file ACEL-23-e14268-s017.zip › Data S1/ENSMUSG00000000682.jpeg]

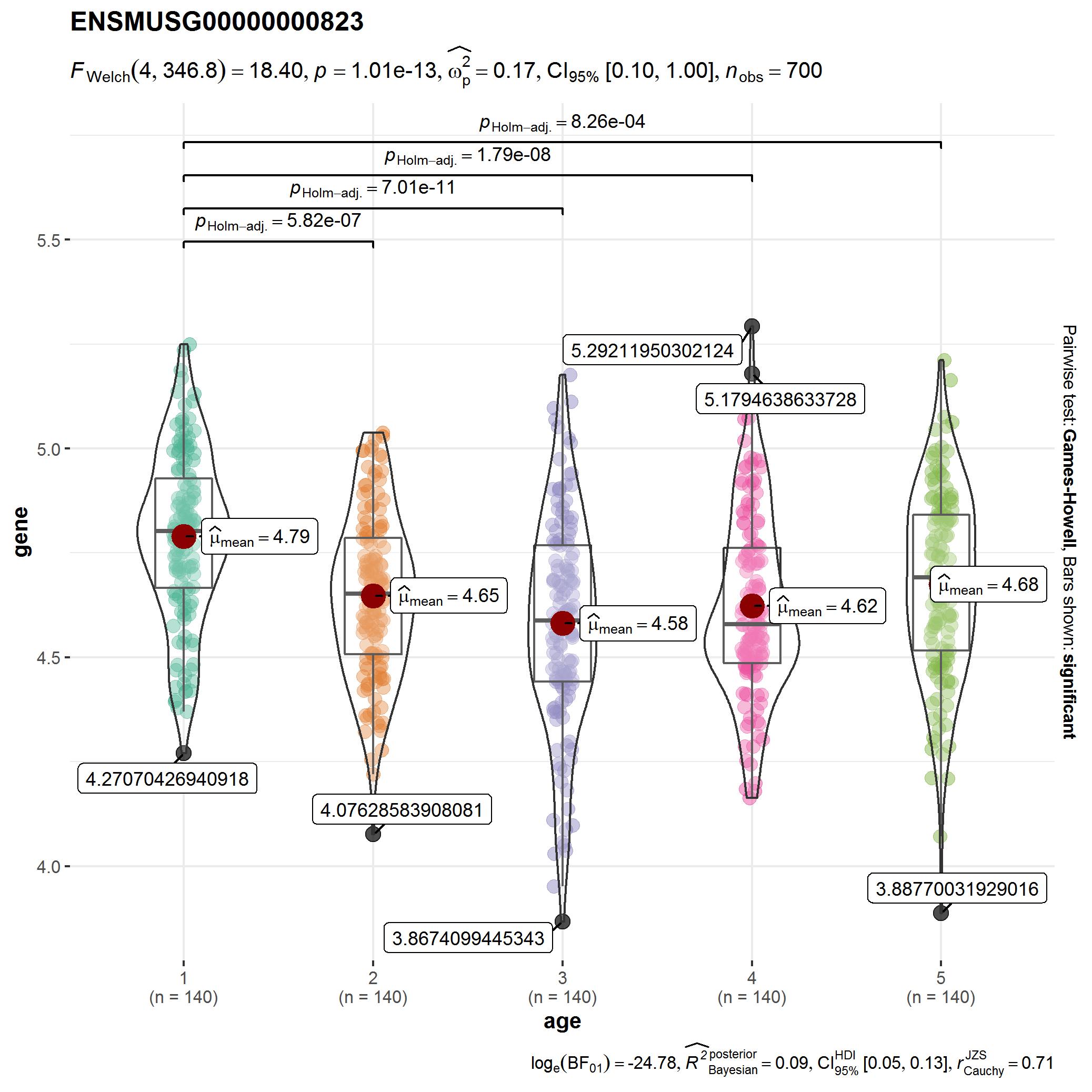

Supplement: Supplementary file 25 — Data S1–S6. [file ACEL-23-e14268-s017.zip › Data S1/ENSMUSG00000000823.jpeg]

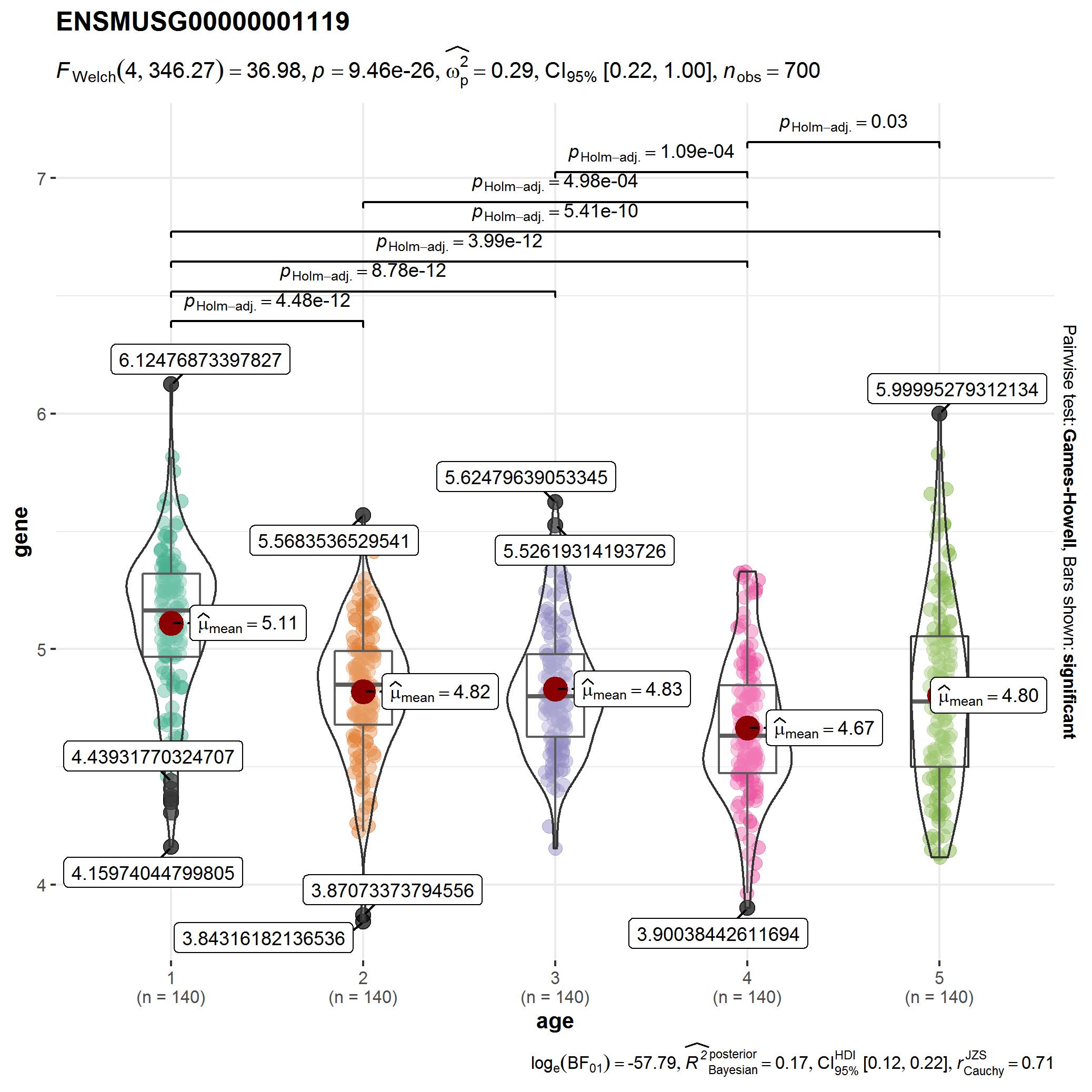

Supplement: Supplementary file 25 — Data S1–S6. [file ACEL-23-e14268-s017.zip › Data S1/ENSMUSG00000001119.jpeg]

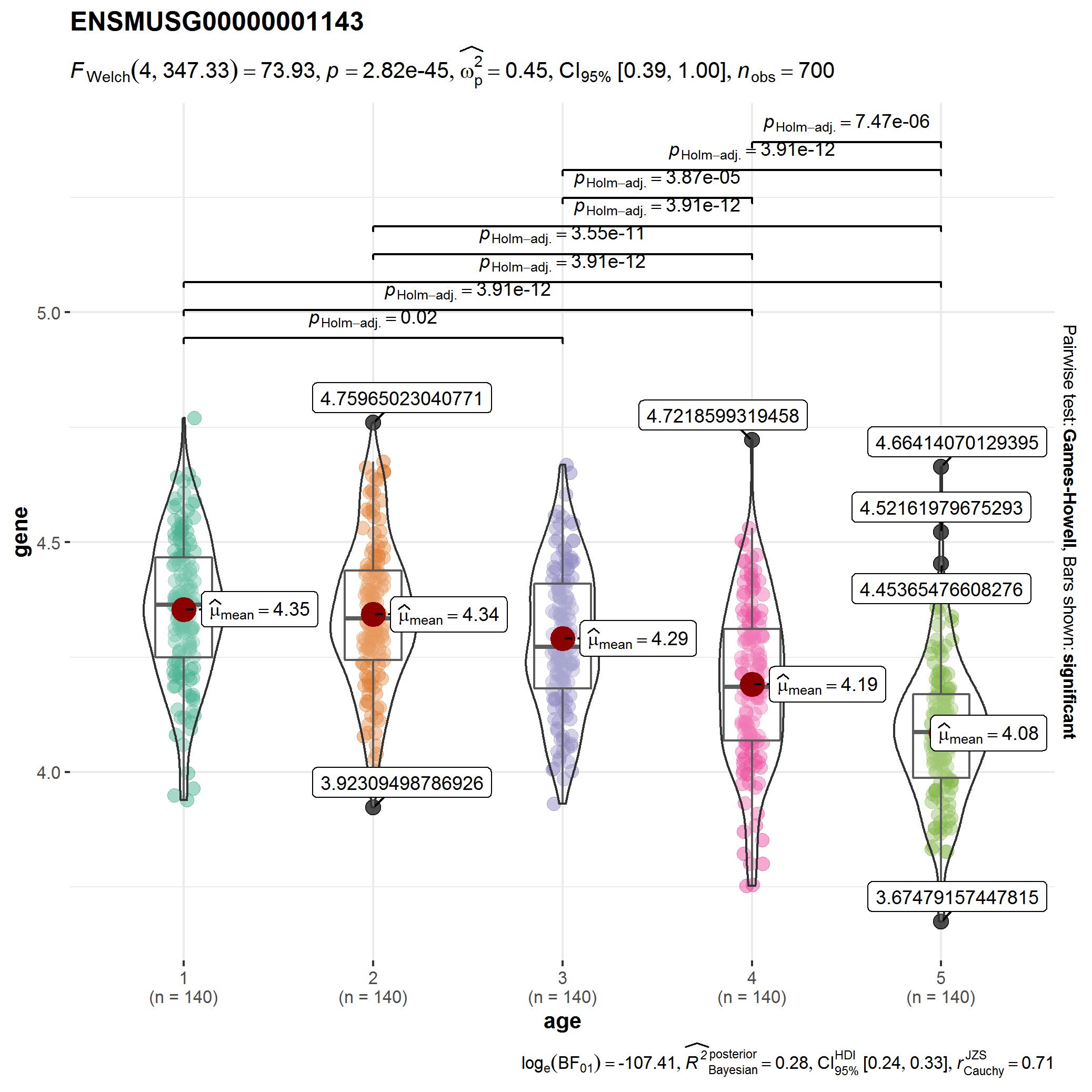

Supplement: Supplementary file 25 — Data S1–S6. [file ACEL-23-e14268-s017.zip › Data S1/ENSMUSG00000001143.jpeg]

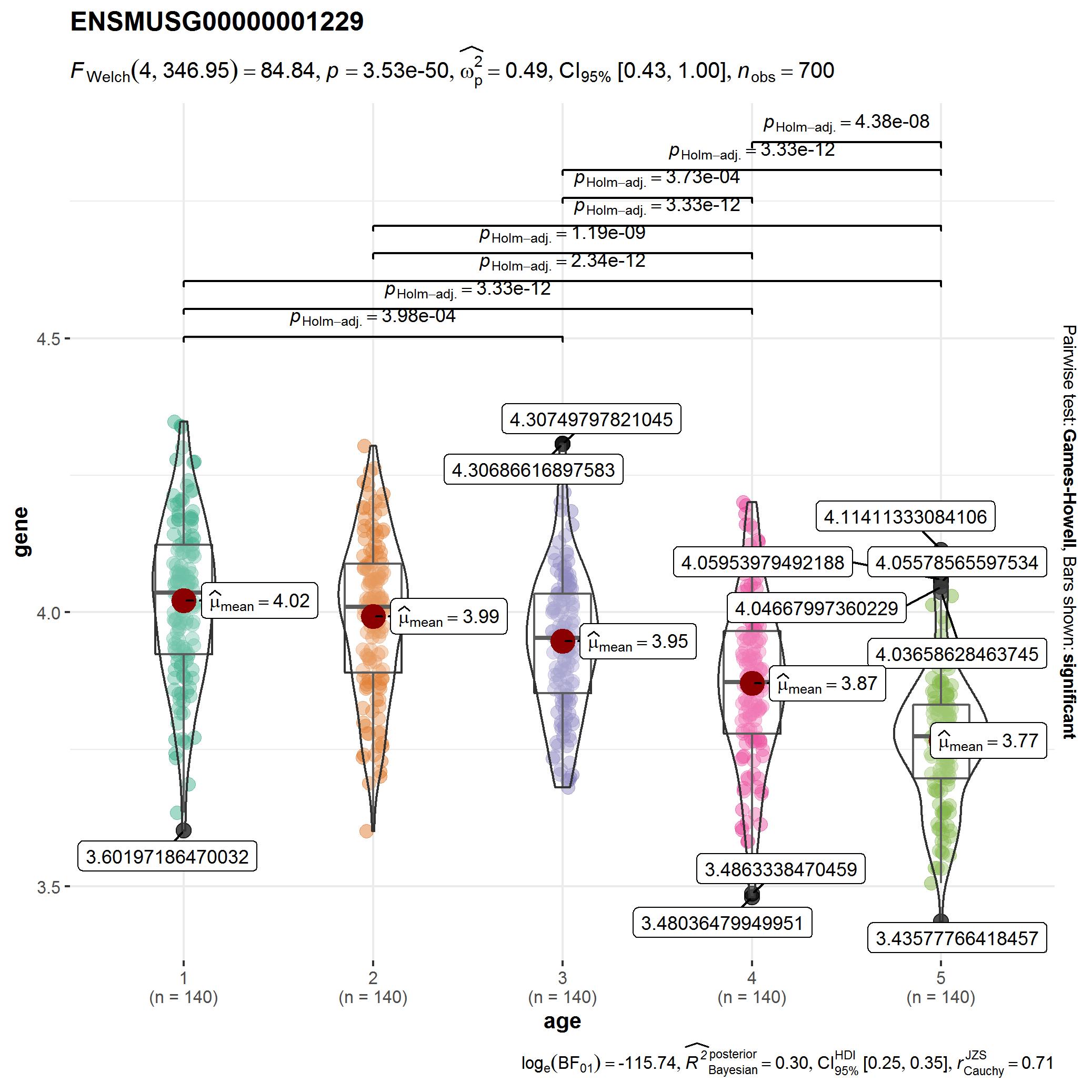

Supplement: Supplementary file 25 — Data S1–S6. [file ACEL-23-e14268-s017.zip › Data S1/ENSMUSG00000001229.jpeg]

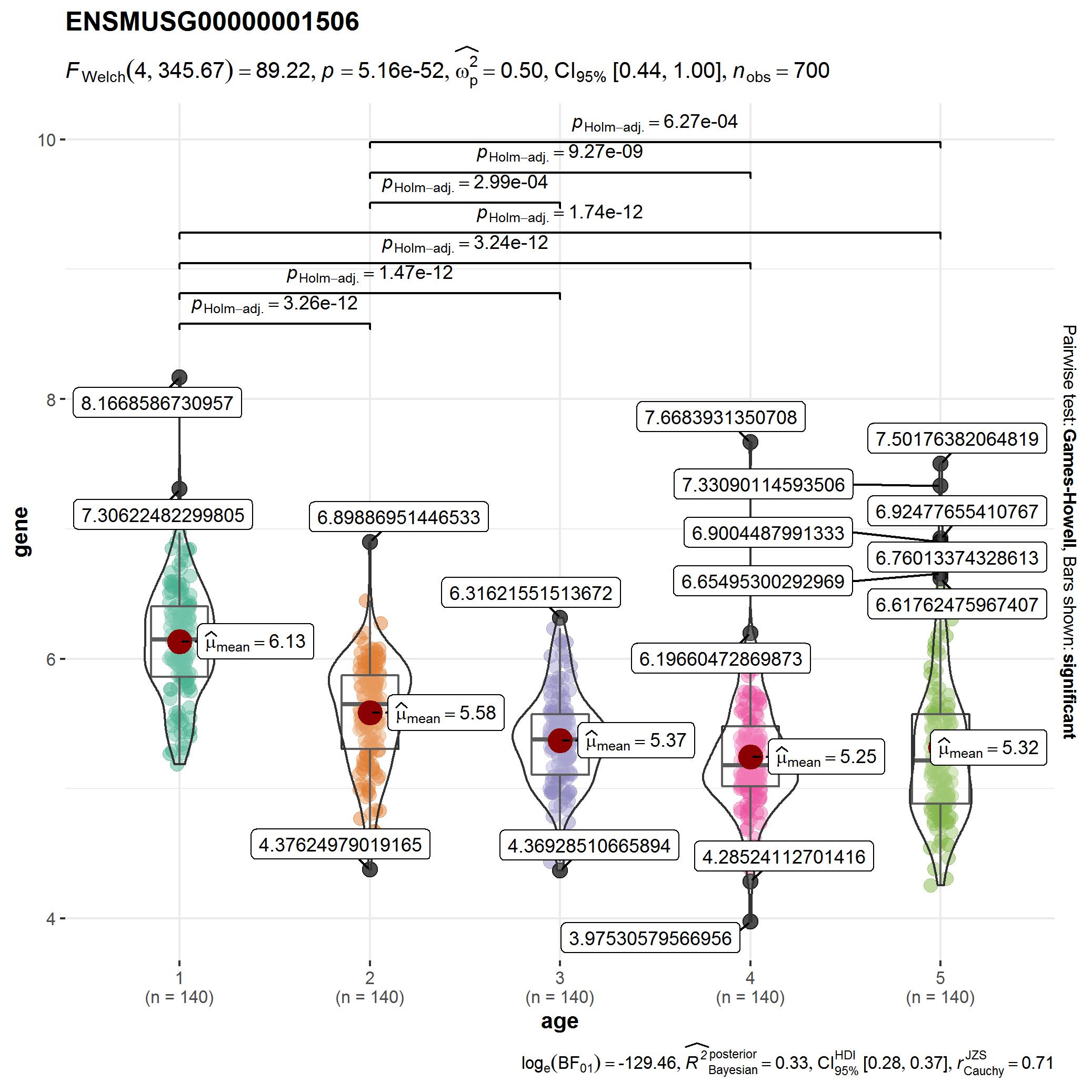

Supplement: Supplementary file 25 — Data S1–S6. [file ACEL-23-e14268-s017.zip › Data S1/ENSMUSG00000001506.jpeg]

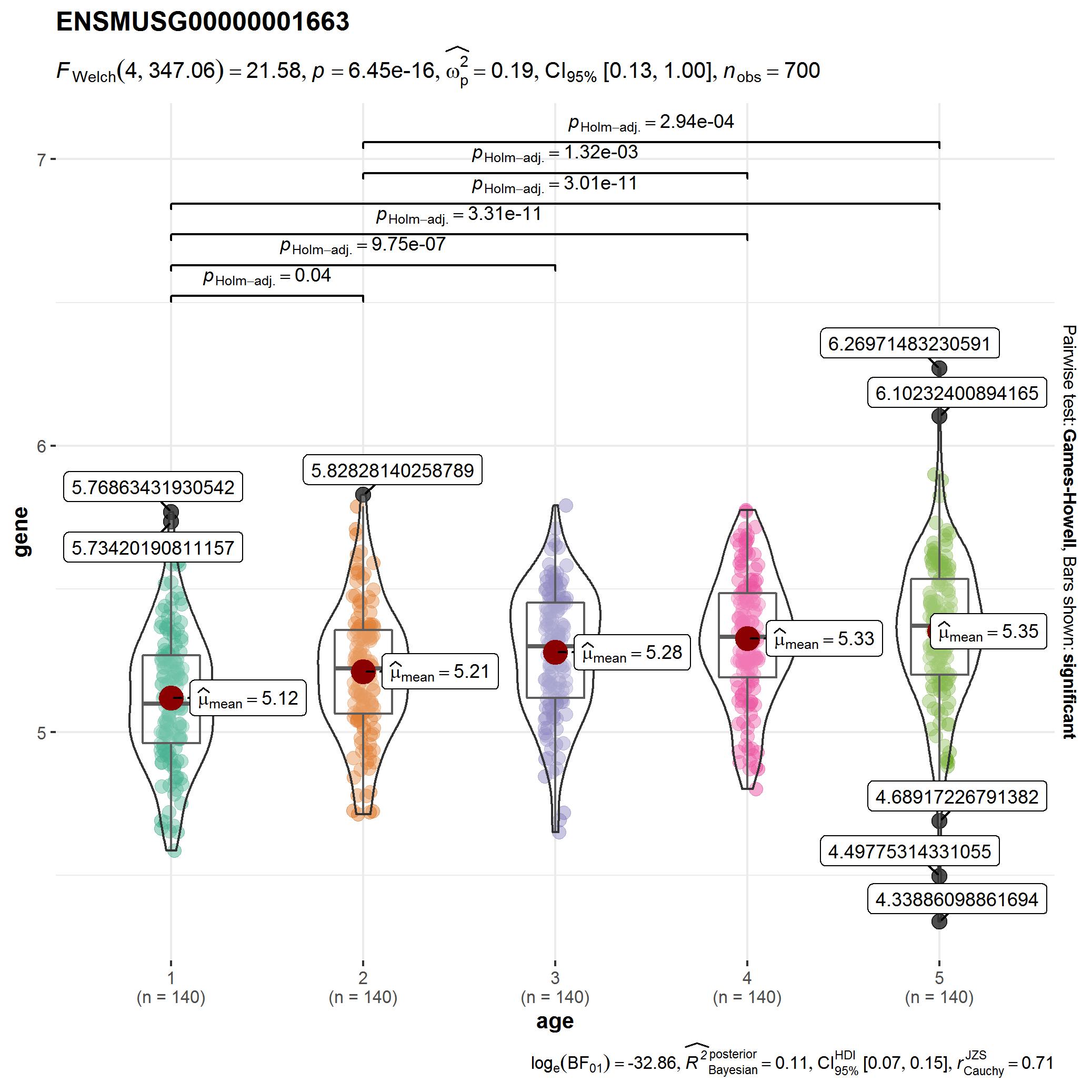

Supplement: Supplementary file 25 — Data S1–S6. [file ACEL-23-e14268-s017.zip › Data S1/ENSMUSG00000001663.jpeg]

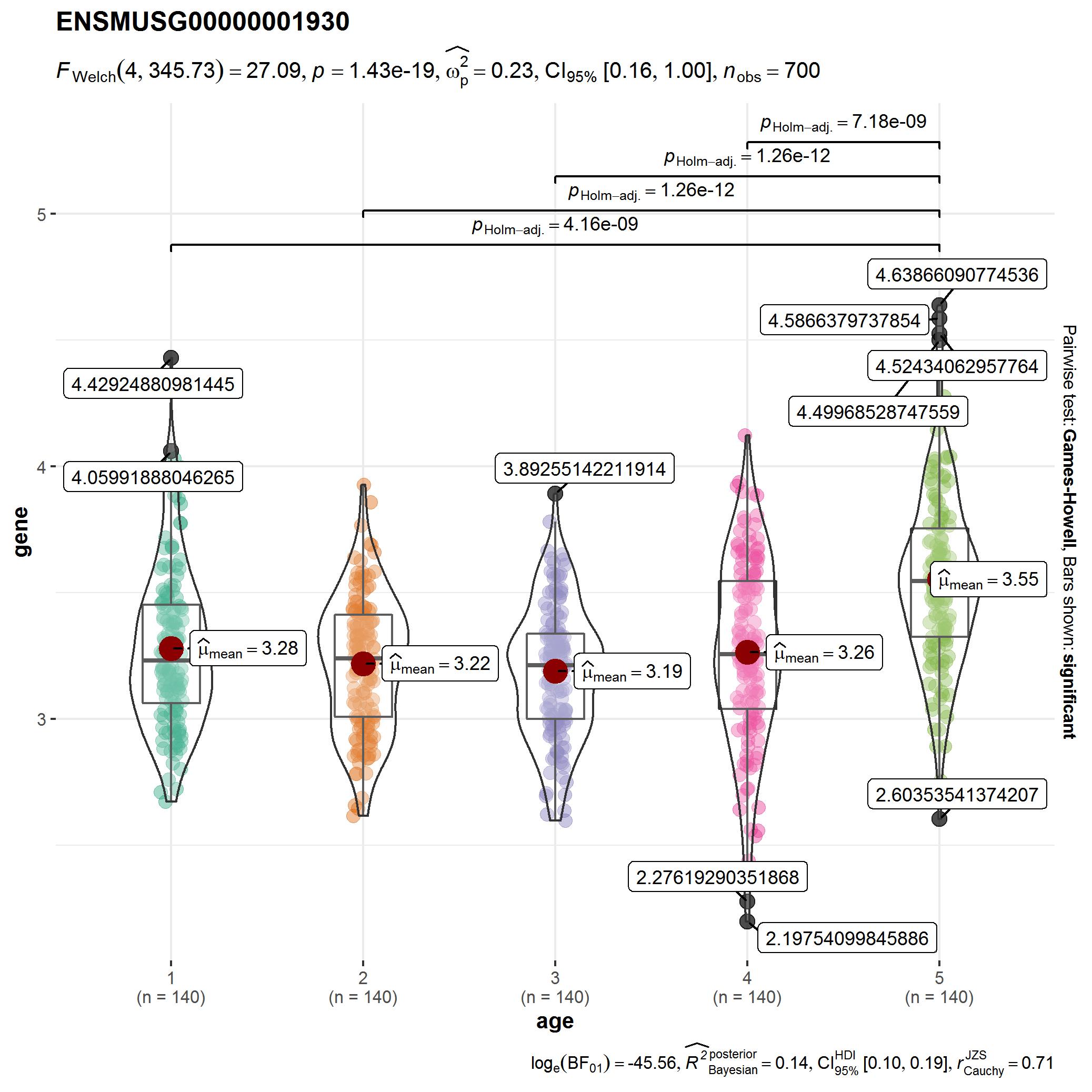

Supplement: Supplementary file 25 — Data S1–S6. [file ACEL-23-e14268-s017.zip › Data S1/ENSMUSG00000001930.jpeg]

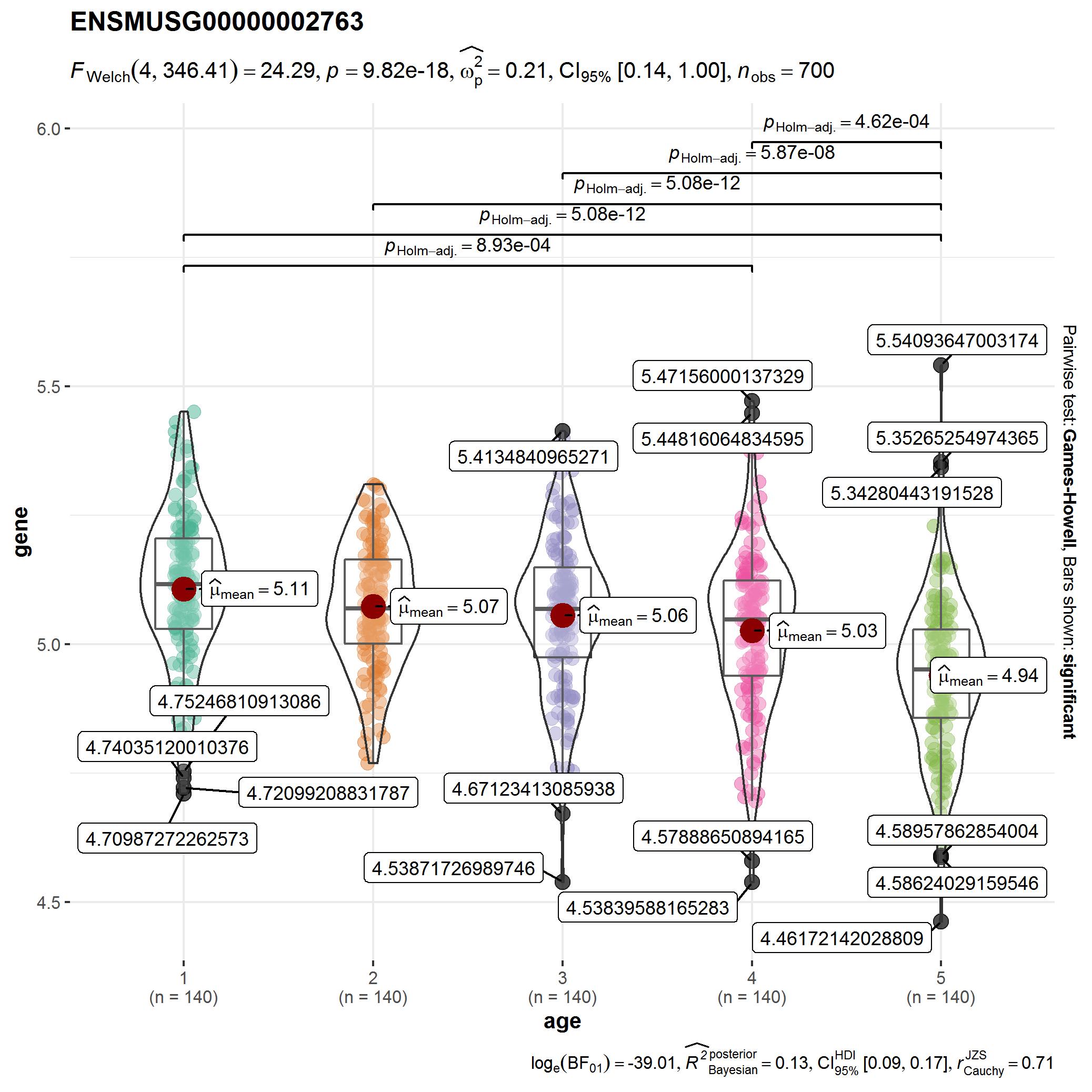

Supplement: Supplementary file 25 — Data S1–S6. [file ACEL-23-e14268-s017.zip › Data S1/ENSMUSG00000002763.jpeg]

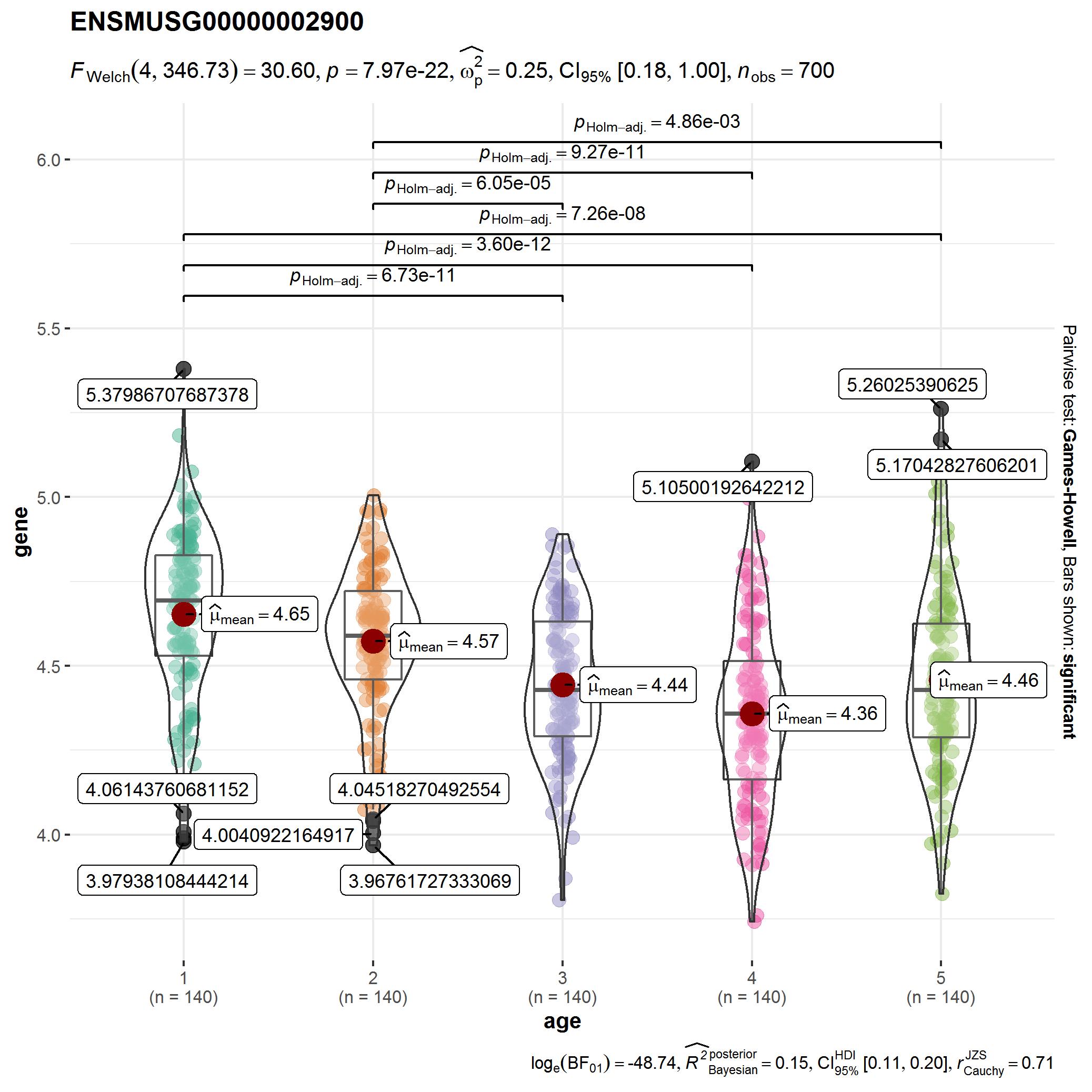

Supplement: Supplementary file 25 — Data S1–S6. [file ACEL-23-e14268-s017.zip › Data S1/ENSMUSG00000002900.jpeg]

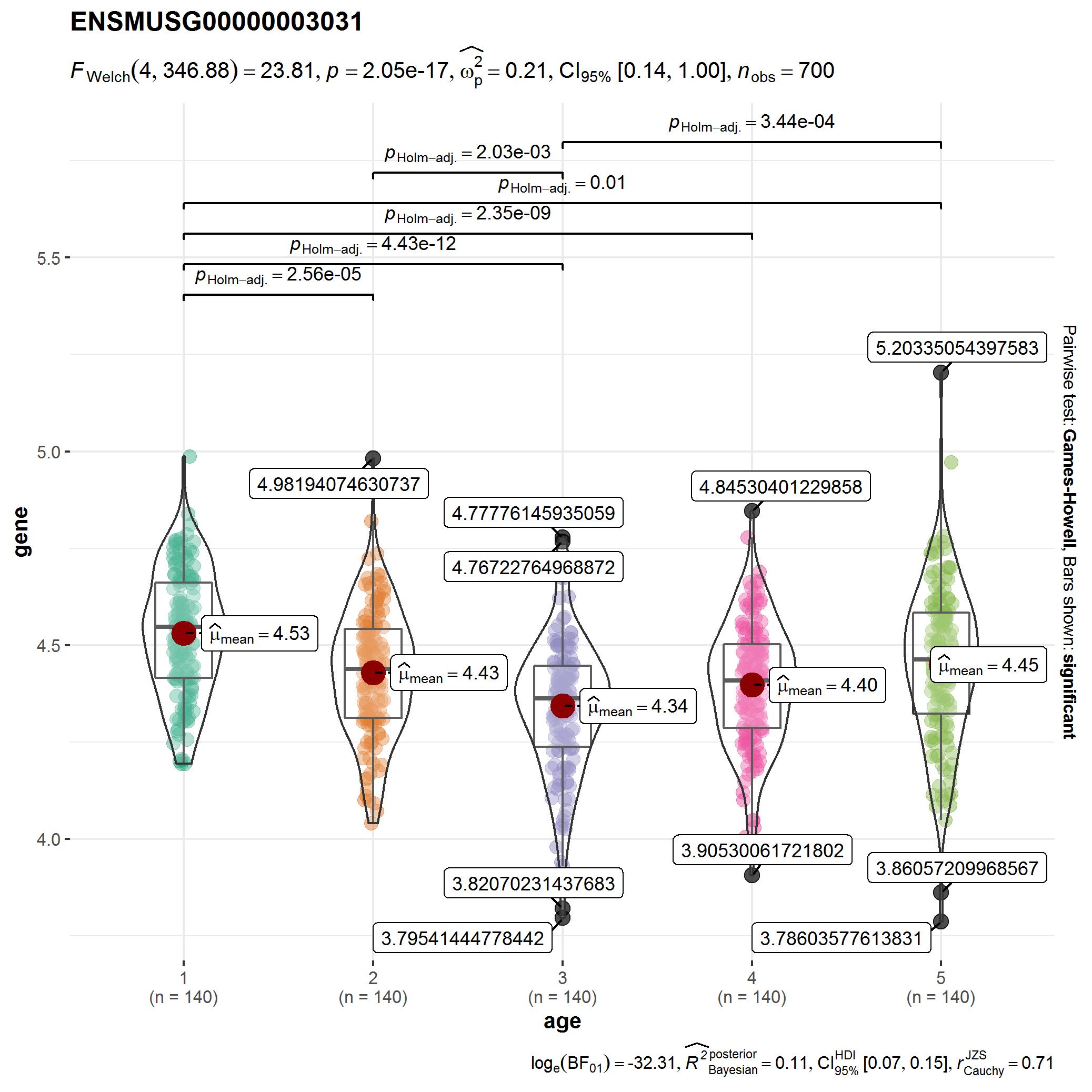

Supplement: Supplementary file 25 — Data S1–S6. [file ACEL-23-e14268-s017.zip › Data S1/ENSMUSG00000003031.jpeg]

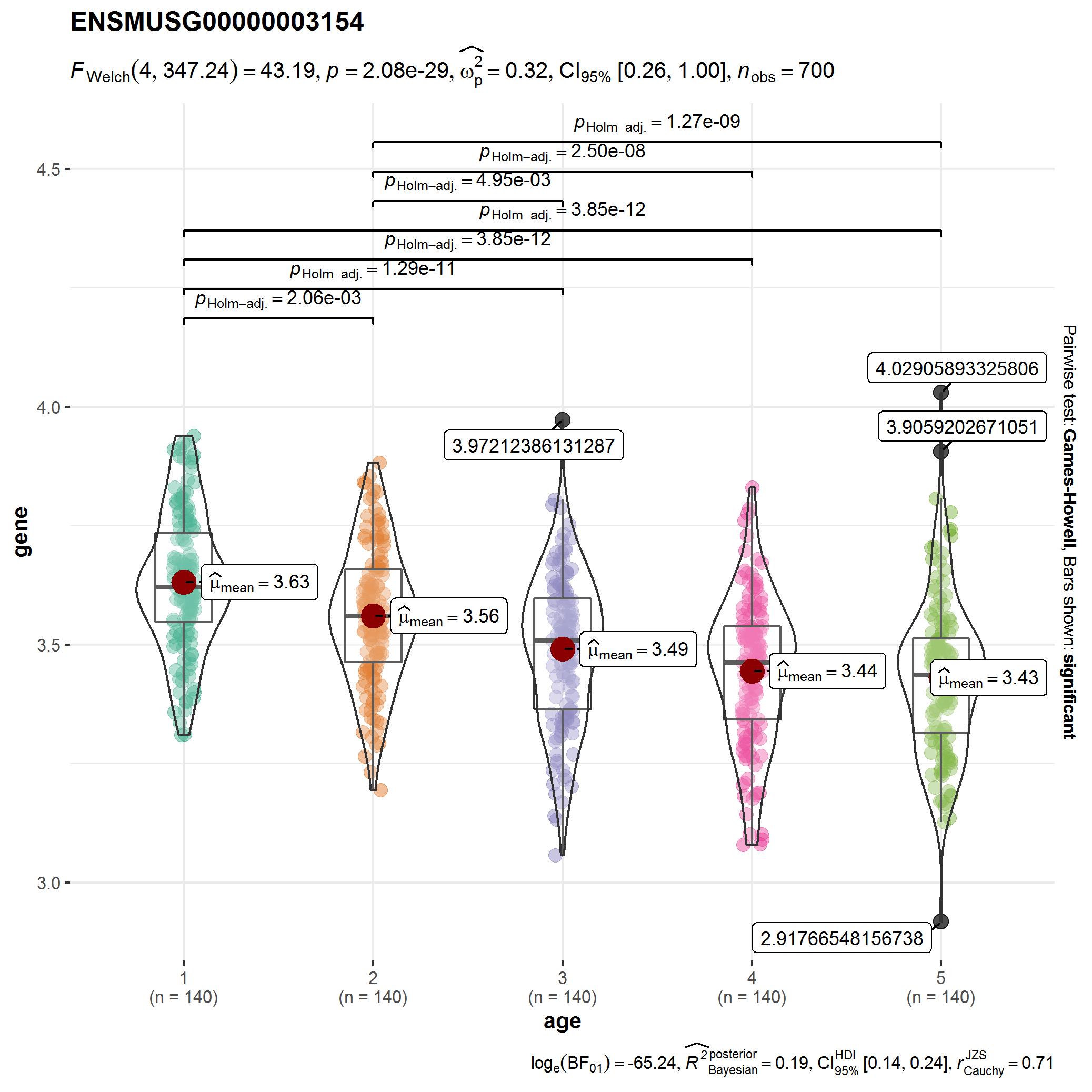

Supplement: Supplementary file 25 — Data S1–S6. [file ACEL-23-e14268-s017.zip › Data S1/ENSMUSG00000003154.jpeg]

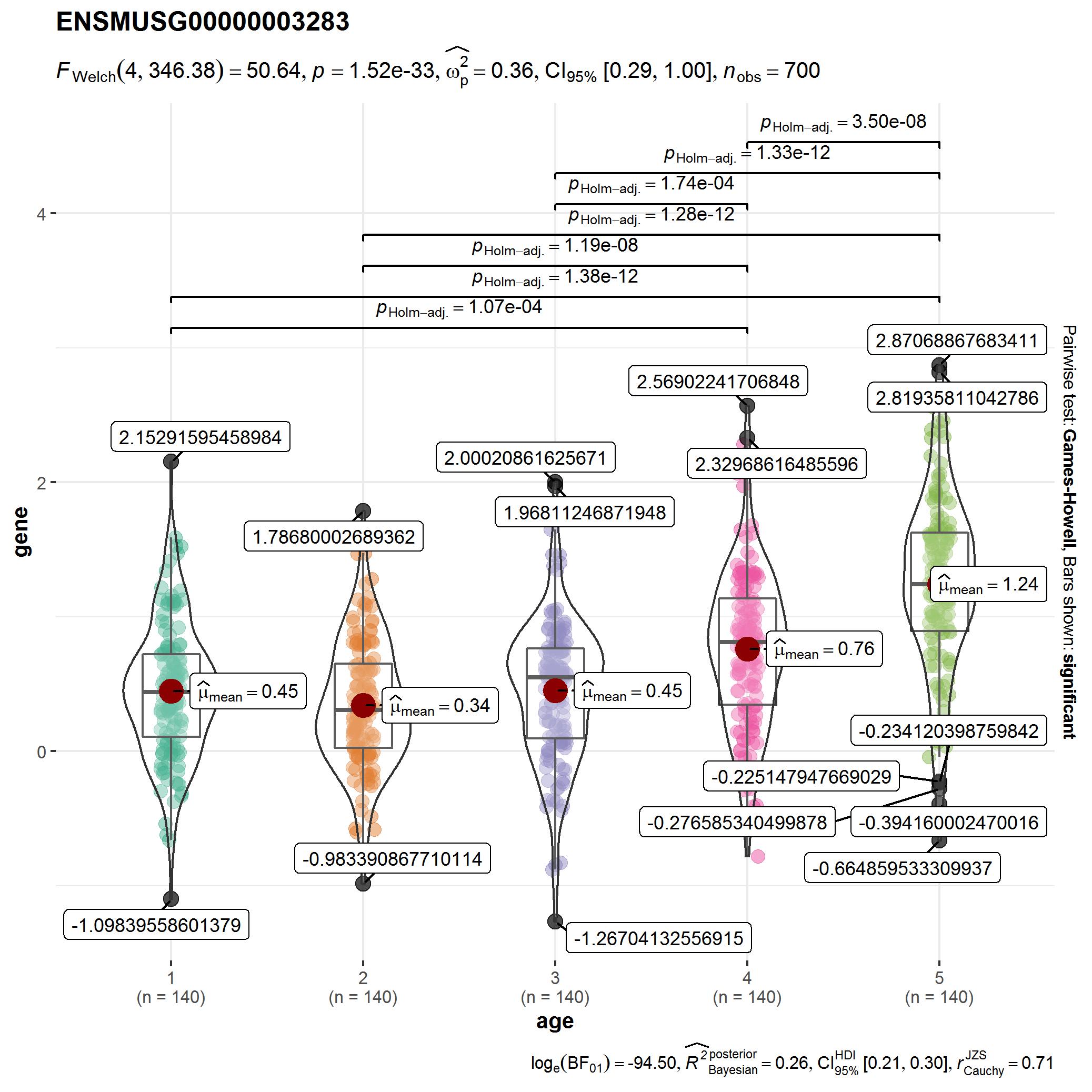

Supplement: Supplementary file 25 — Data S1–S6. [file ACEL-23-e14268-s017.zip › Data S1/ENSMUSG00000003283.jpeg]

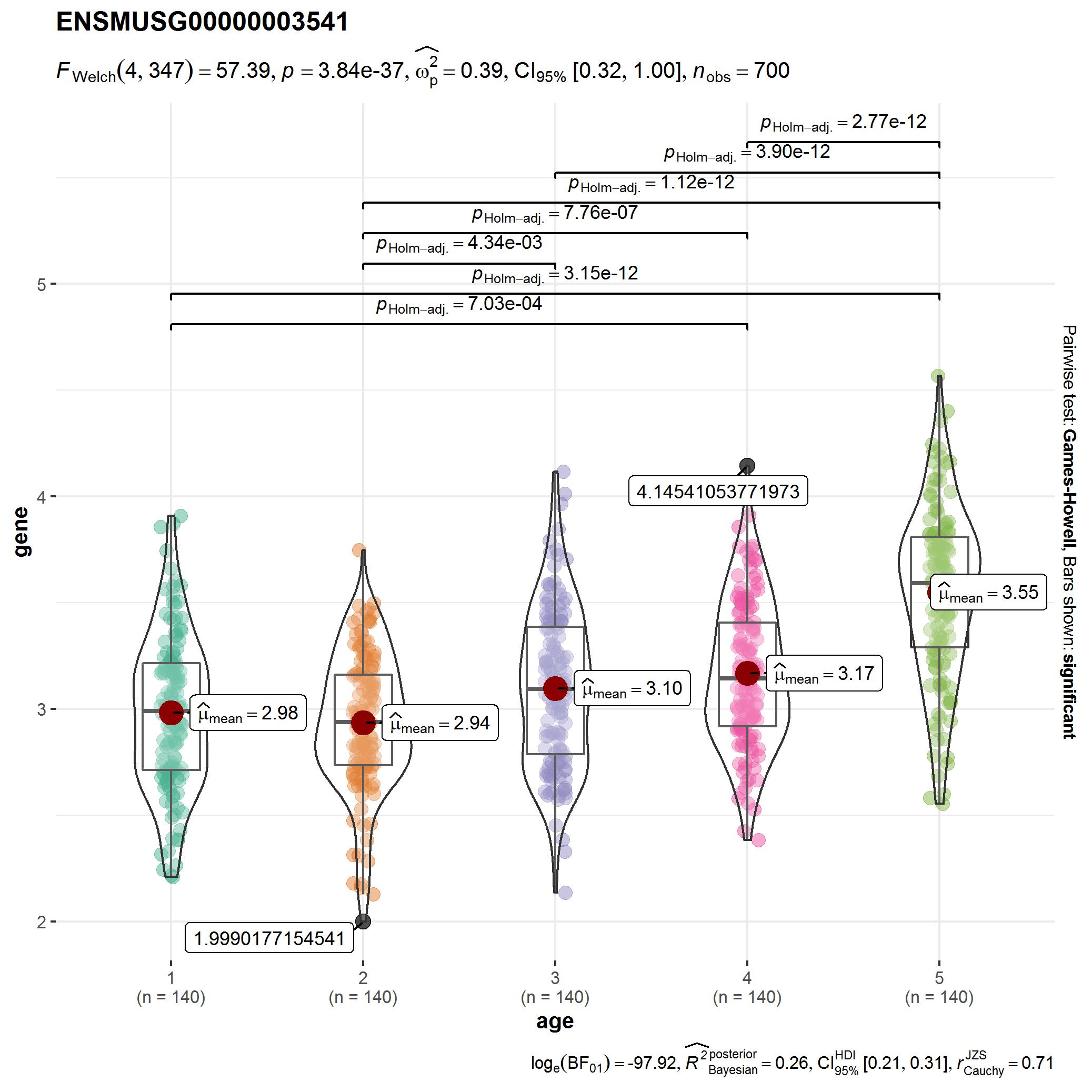

Supplement: Supplementary file 25 — Data S1–S6. [file ACEL-23-e14268-s017.zip › Data S1/ENSMUSG00000003541.jpeg]

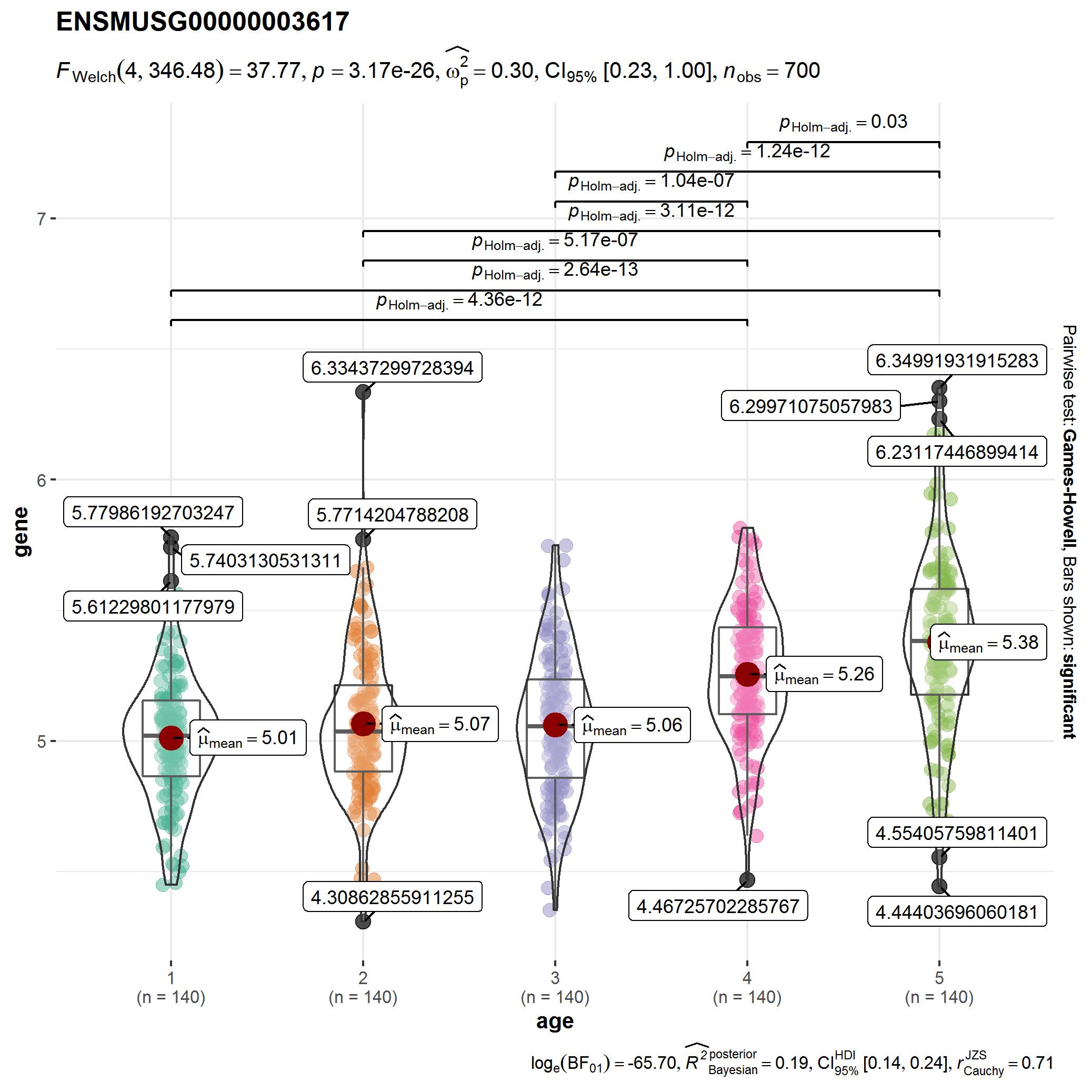

Supplement: Supplementary file 25 — Data S1–S6. [file ACEL-23-e14268-s017.zip › Data S1/ENSMUSG00000003617.jpeg]

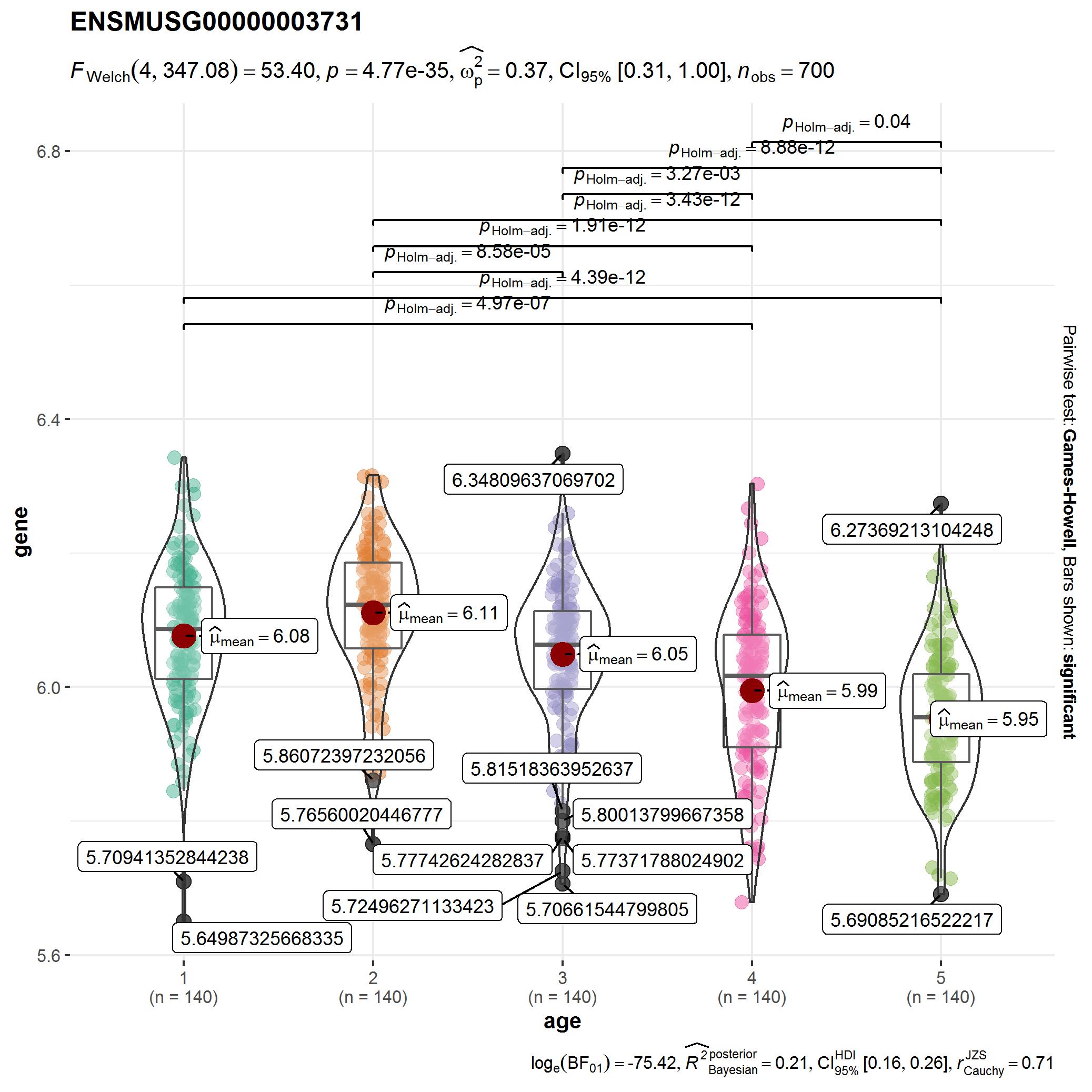

Supplement: Supplementary file 25 — Data S1–S6. [file ACEL-23-e14268-s017.zip › Data S1/ENSMUSG00000003731.jpeg]

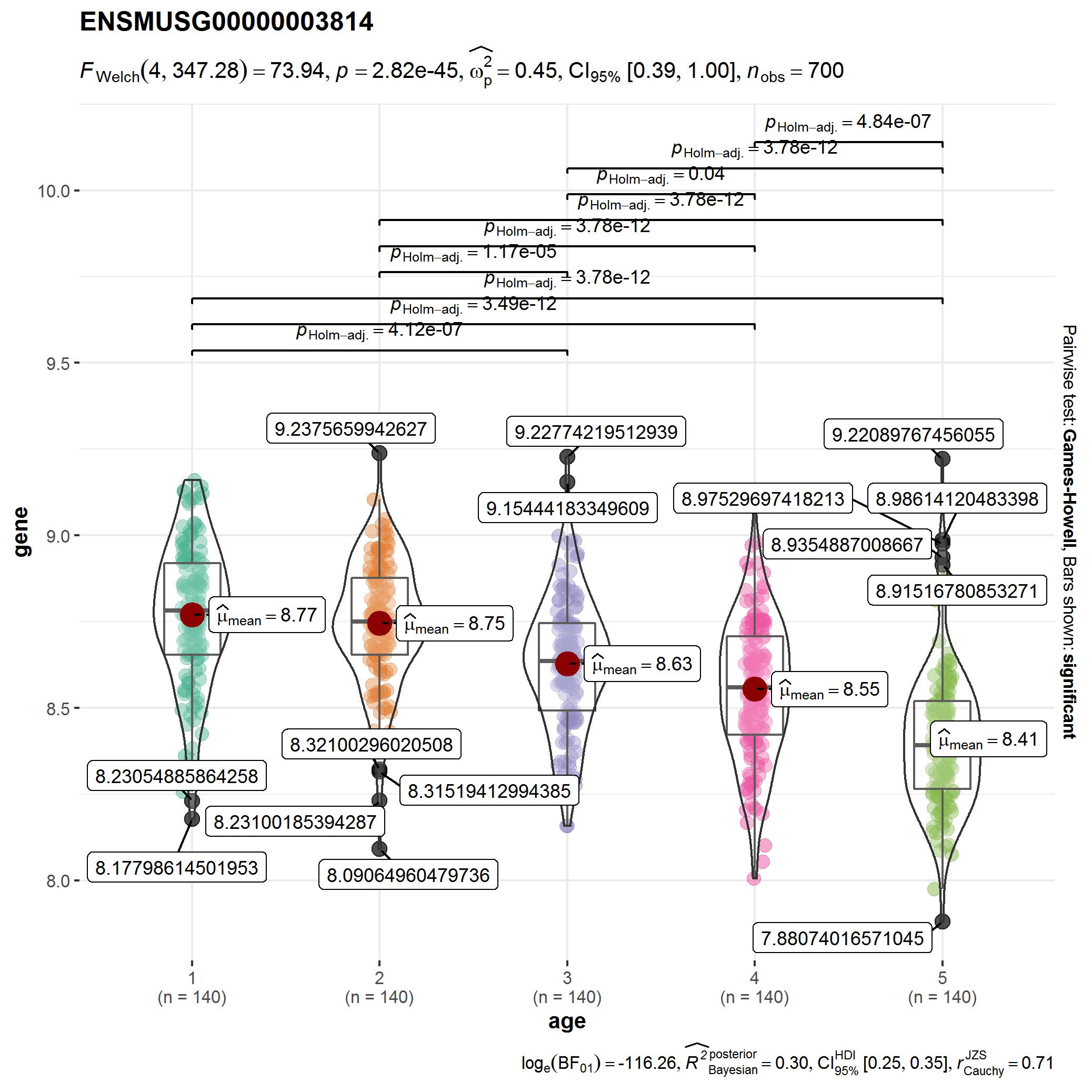

Supplement: Supplementary file 25 — Data S1–S6. [file ACEL-23-e14268-s017.zip › Data S1/ENSMUSG00000003814.jpeg]

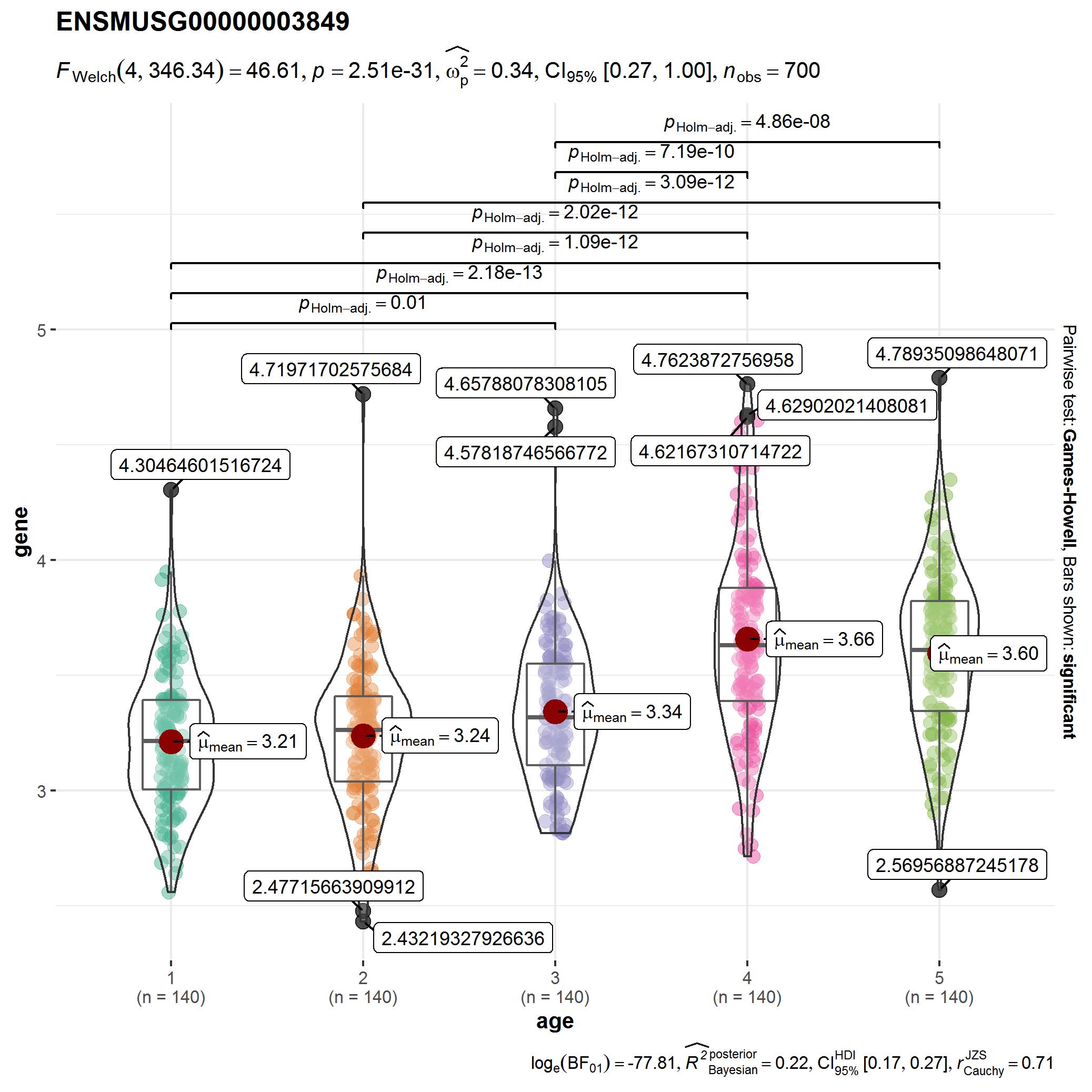

Supplement: Supplementary file 25 — Data S1–S6. [file ACEL-23-e14268-s017.zip › Data S1/ENSMUSG00000003849.jpeg]

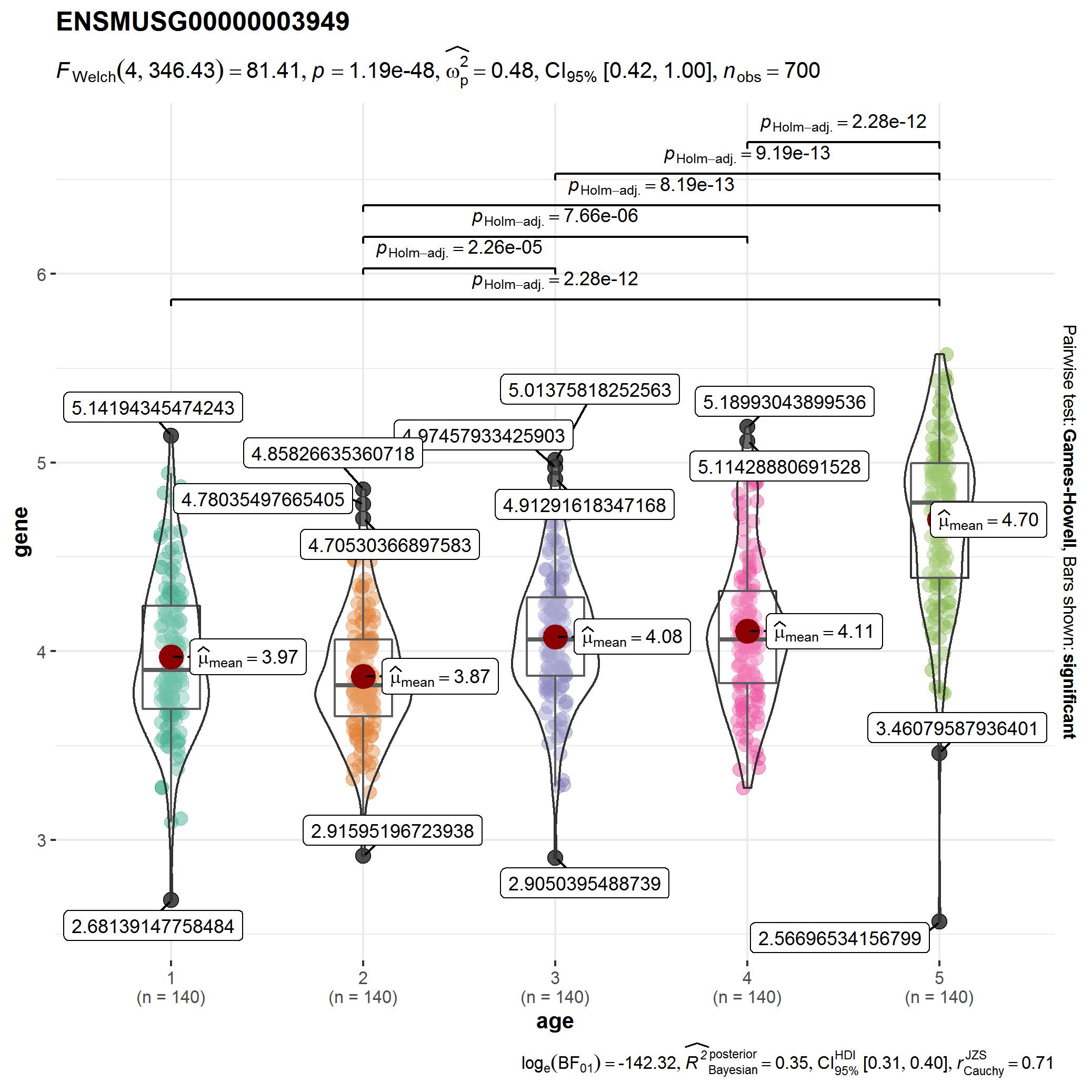

Supplement: Supplementary file 25 — Data S1–S6. [file ACEL-23-e14268-s017.zip › Data S1/ENSMUSG00000003949.jpeg]

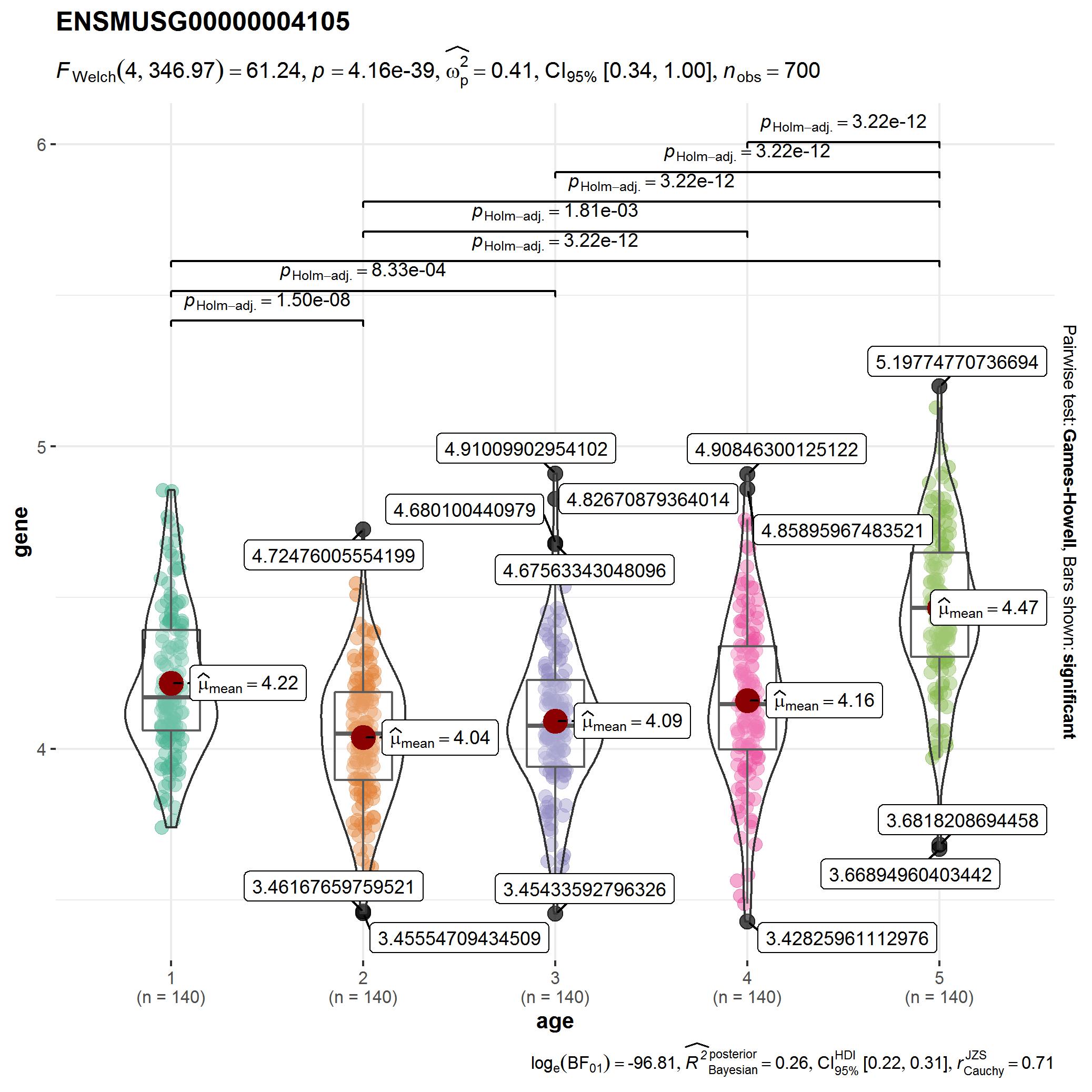

Supplement: Supplementary file 25 — Data S1–S6. [file ACEL-23-e14268-s017.zip › Data S1/ENSMUSG00000004105.jpeg]

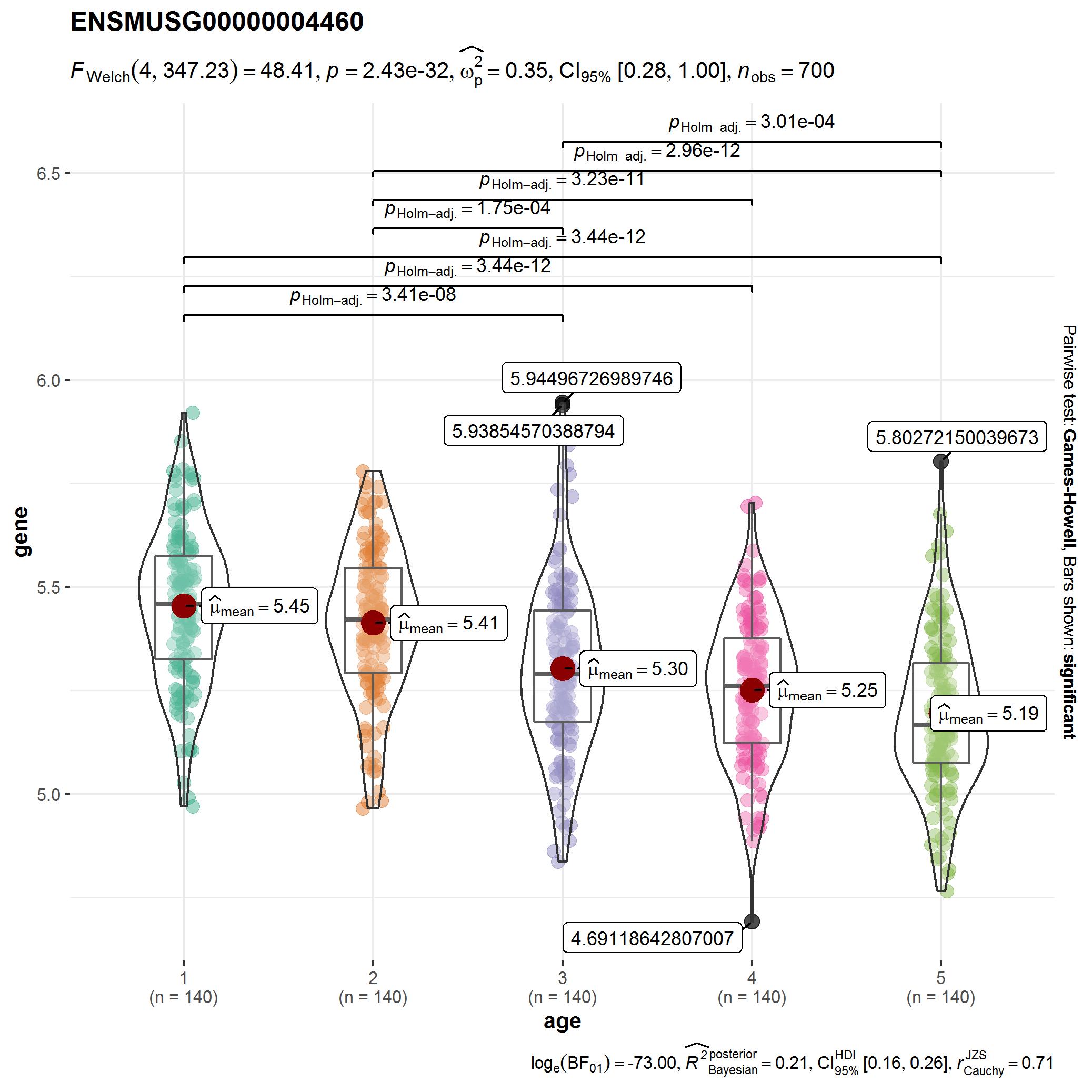

Supplement: Supplementary file 25 — Data S1–S6. [file ACEL-23-e14268-s017.zip › Data S1/ENSMUSG00000004460.jpeg]

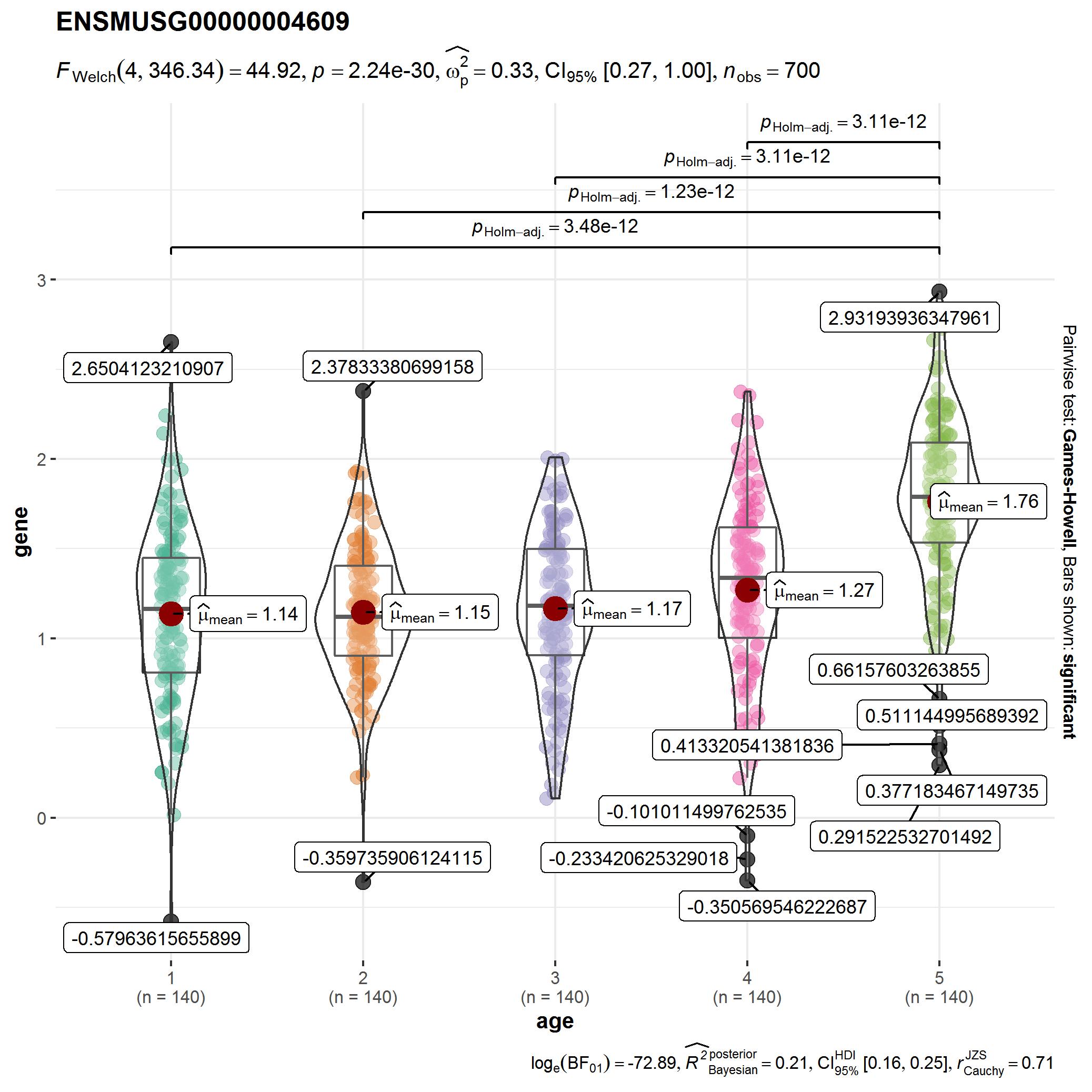

Supplement: Supplementary file 25 — Data S1–S6. [file ACEL-23-e14268-s017.zip › Data S1/ENSMUSG00000004609.jpeg]

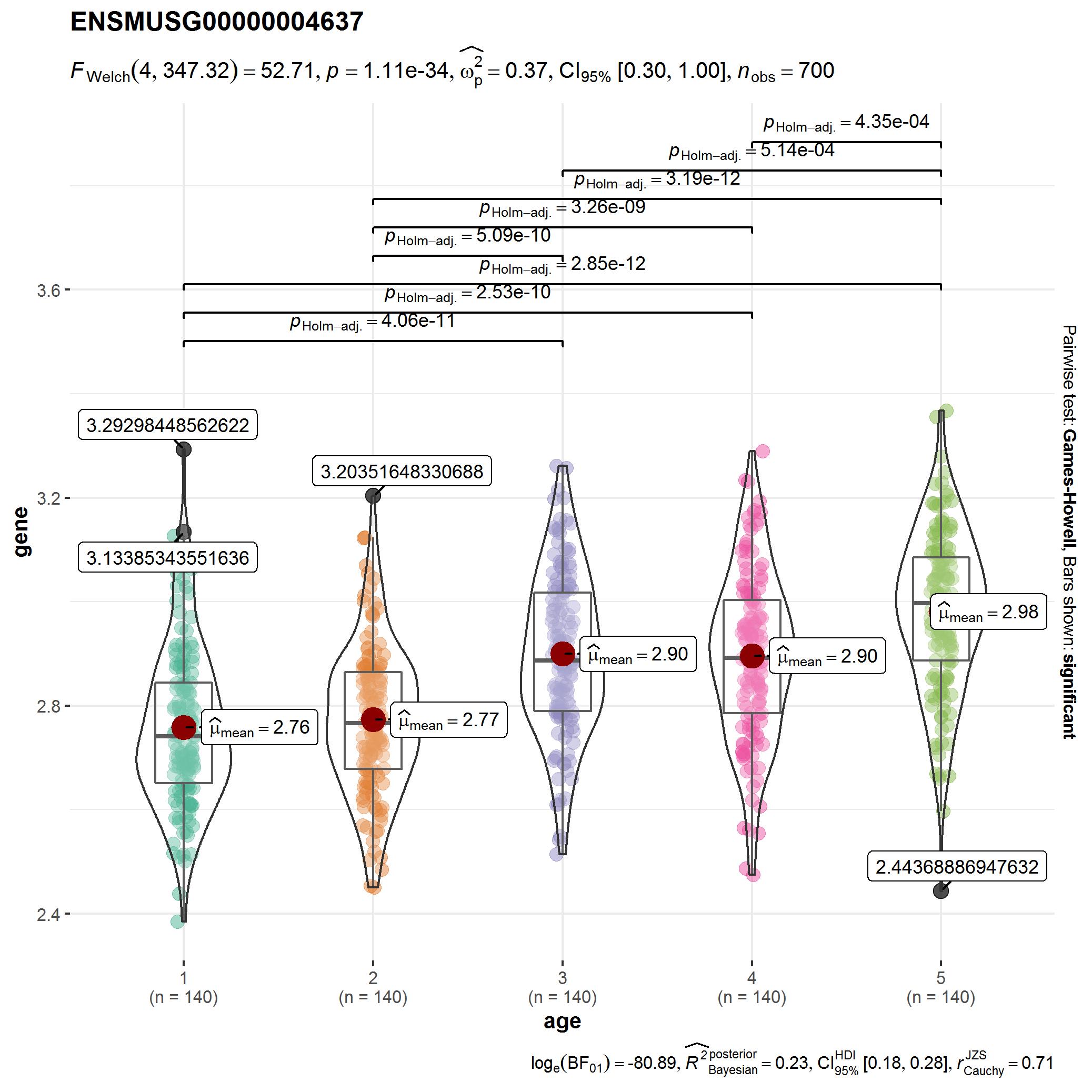

Supplement: Supplementary file 25 — Data S1–S6. [file ACEL-23-e14268-s017.zip › Data S1/ENSMUSG00000004637.jpeg]

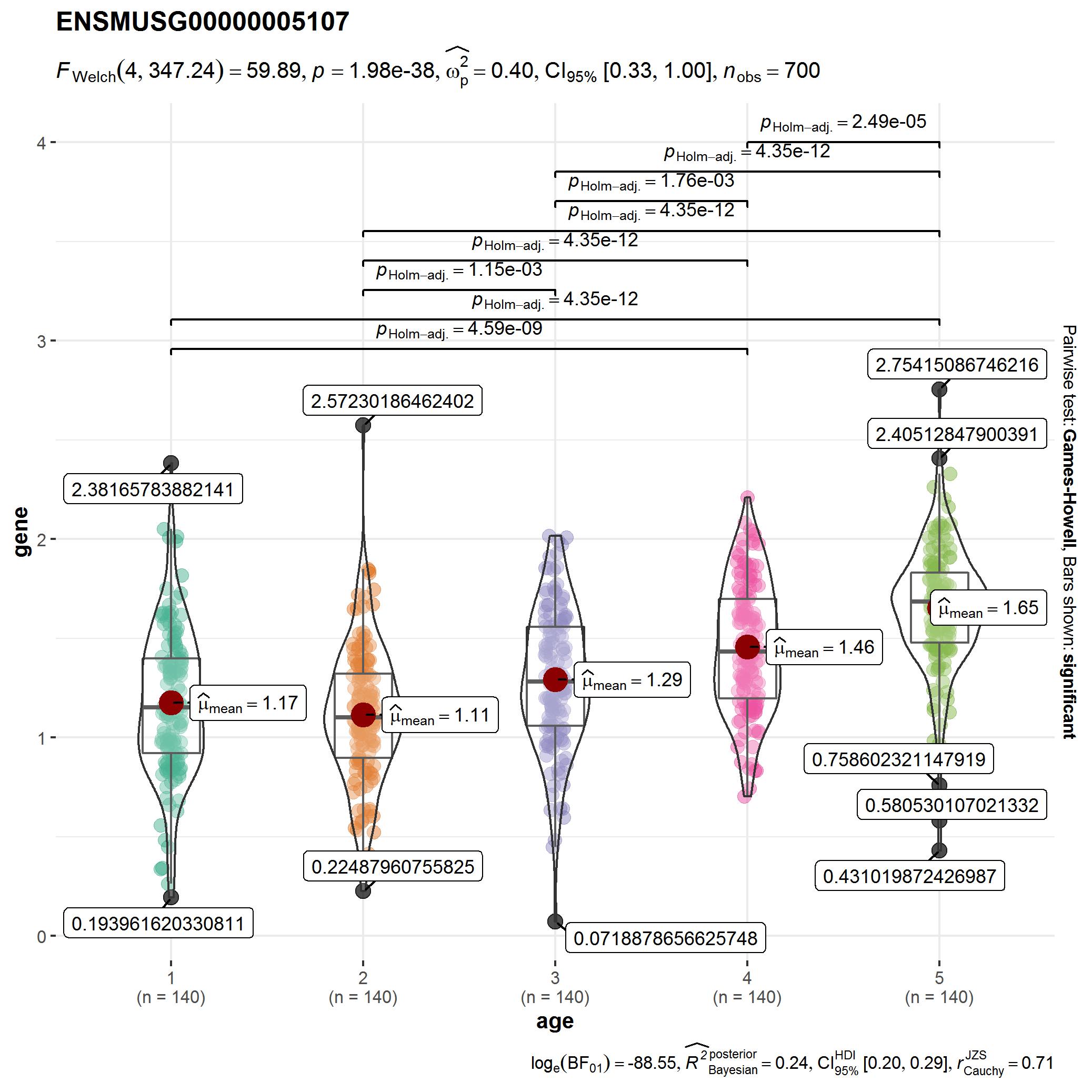

Supplement: Supplementary file 25 — Data S1–S6. [file ACEL-23-e14268-s017.zip › Data S1/ENSMUSG00000005107.jpeg]

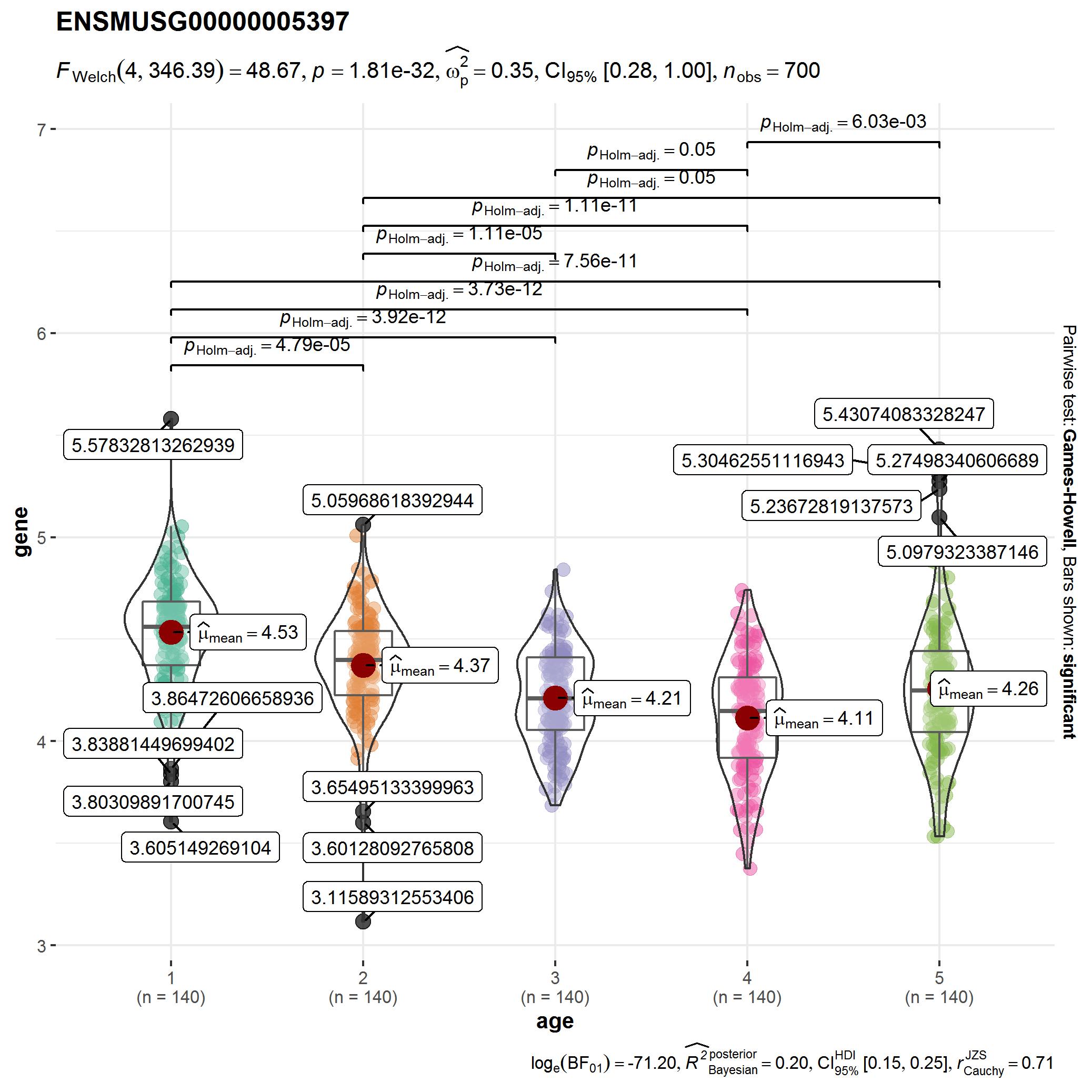

Supplement: Supplementary file 25 — Data S1–S6. [file ACEL-23-e14268-s017.zip › Data S1/ENSMUSG00000005397.jpeg]

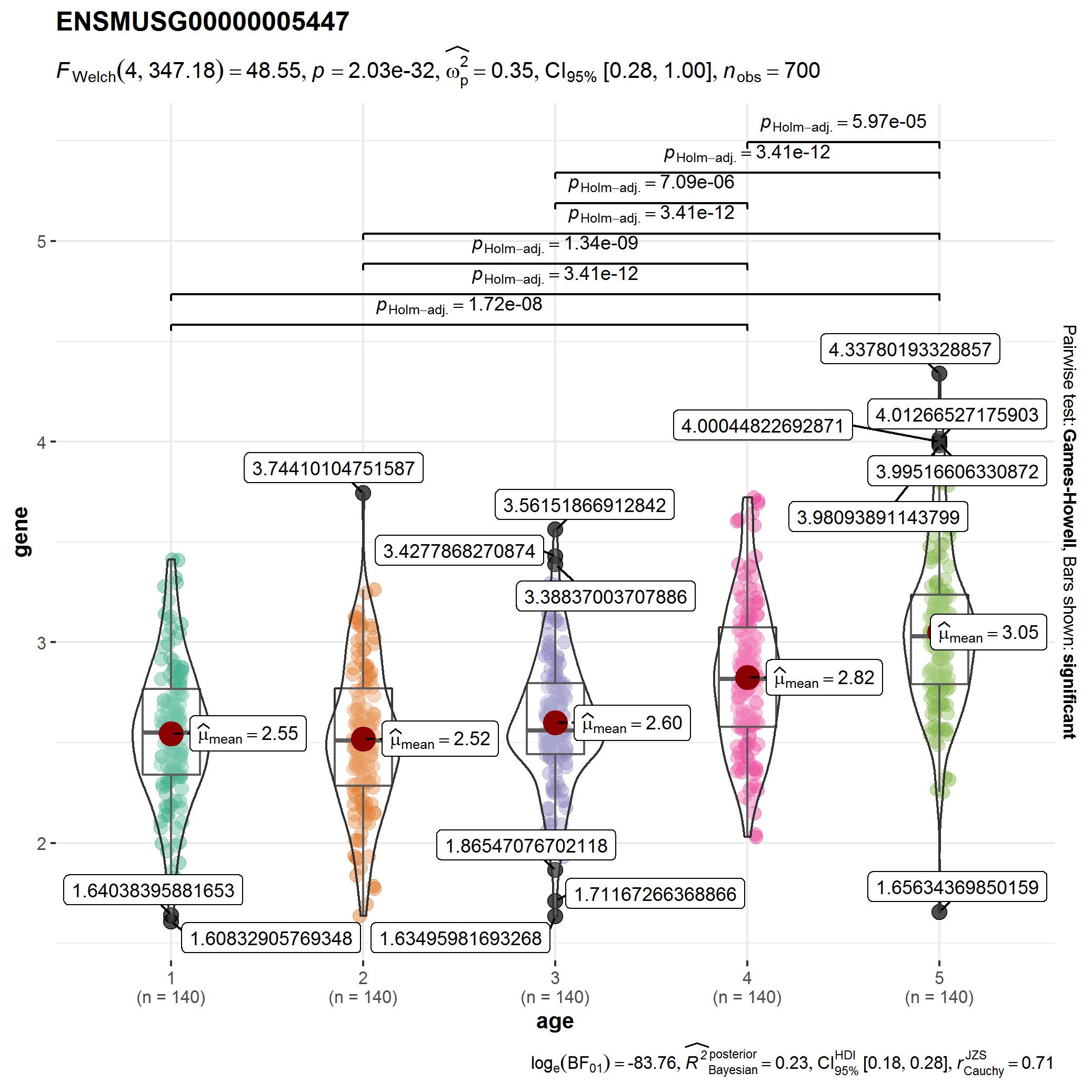

Supplement: Supplementary file 25 — Data S1–S6. [file ACEL-23-e14268-s017.zip › Data S1/ENSMUSG00000005447.jpeg]

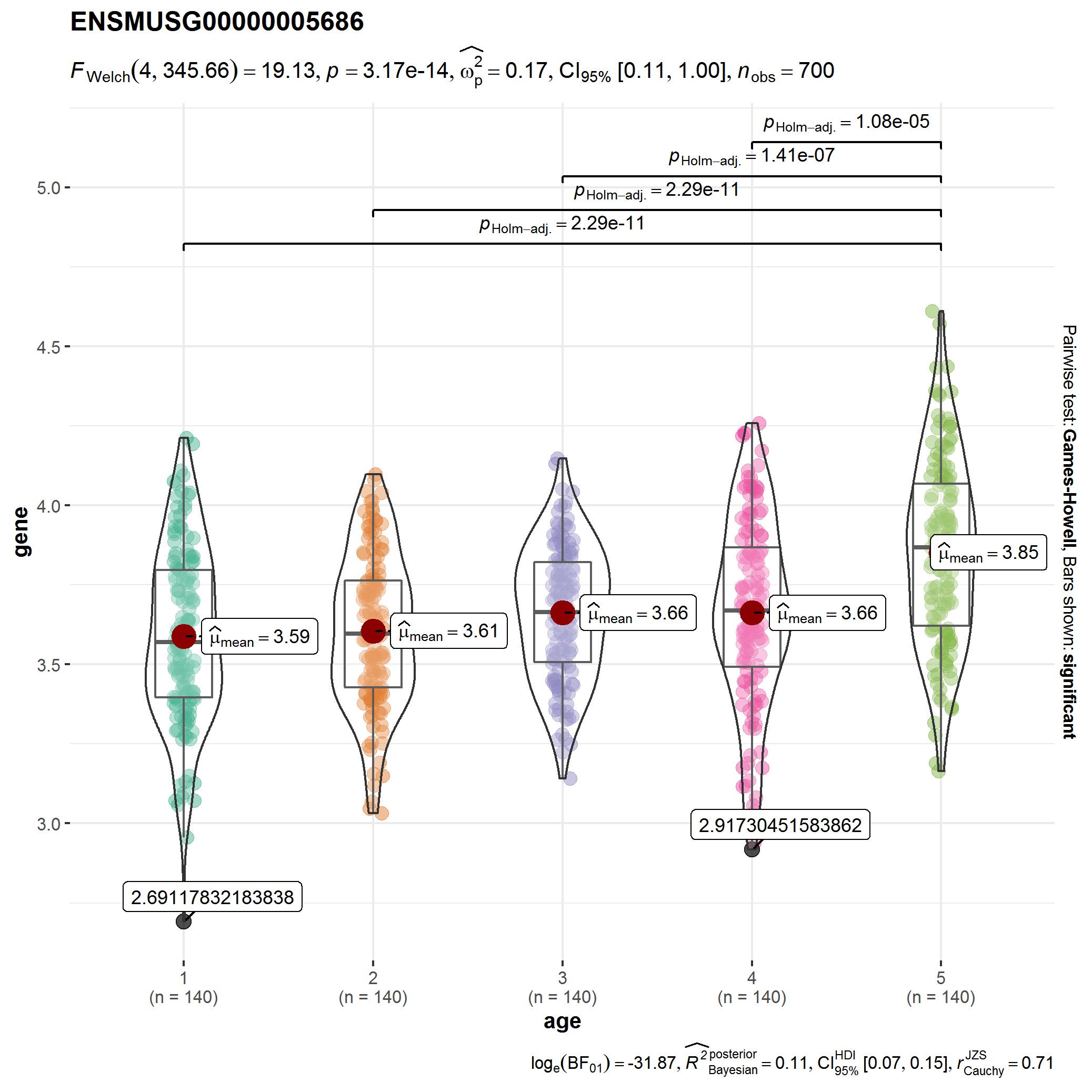

Supplement: Supplementary file 25 — Data S1–S6. [file ACEL-23-e14268-s017.zip › Data S1/ENSMUSG00000005686.jpeg]

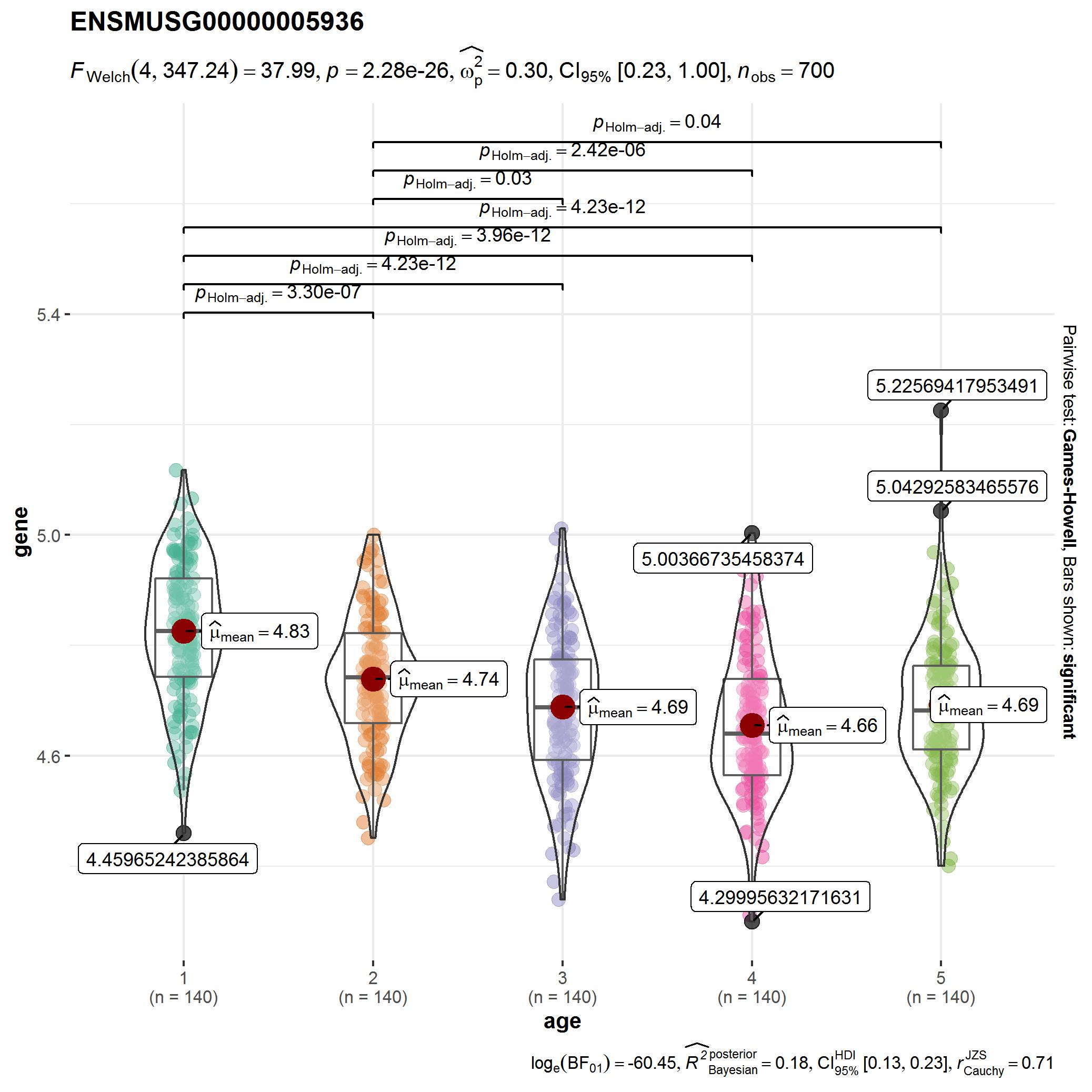

Supplement: Supplementary file 25 — Data S1–S6. [file ACEL-23-e14268-s017.zip › Data S1/ENSMUSG00000005936.jpeg]

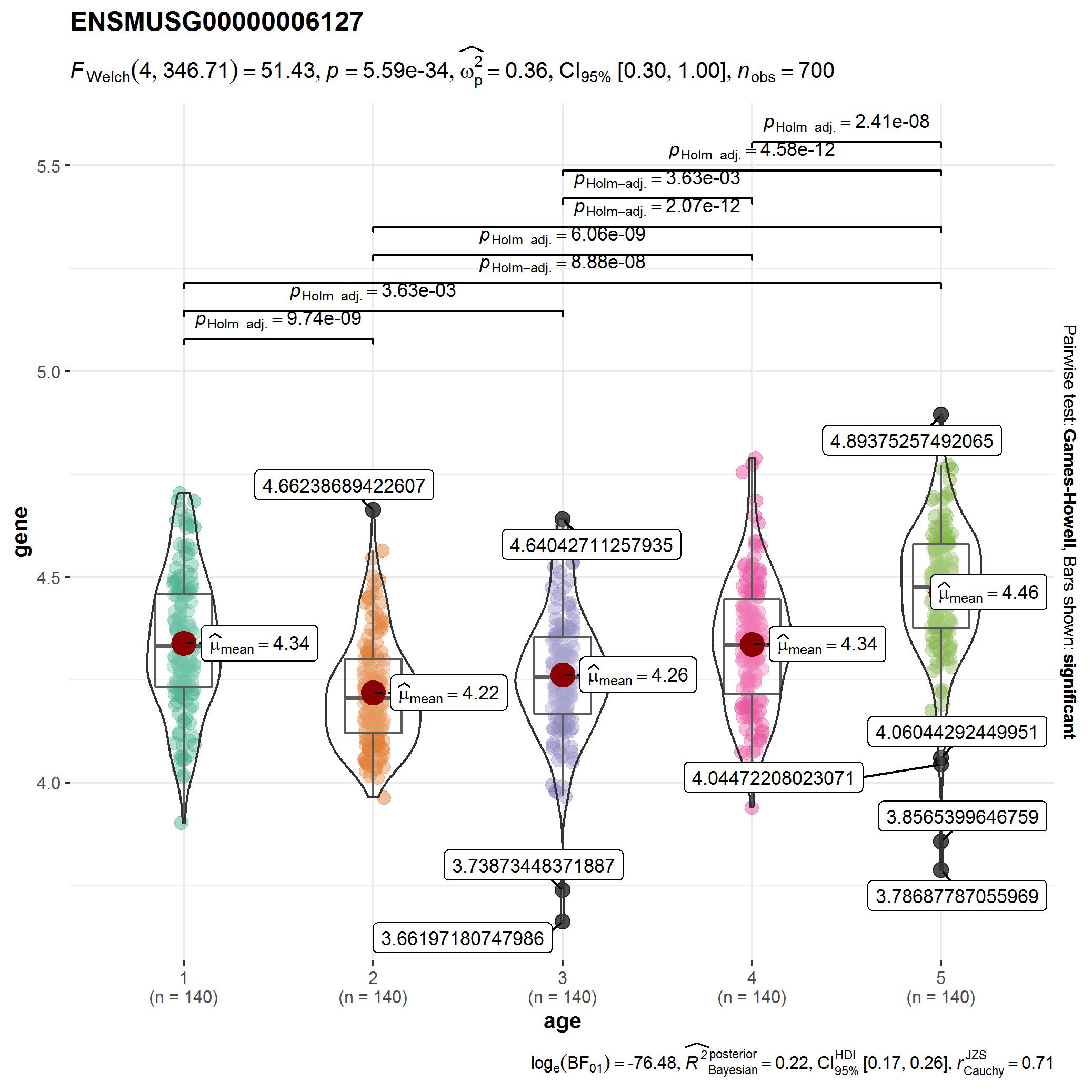

Supplement: Supplementary file 25 — Data S1–S6. [file ACEL-23-e14268-s017.zip › Data S1/ENSMUSG00000006127.jpeg]

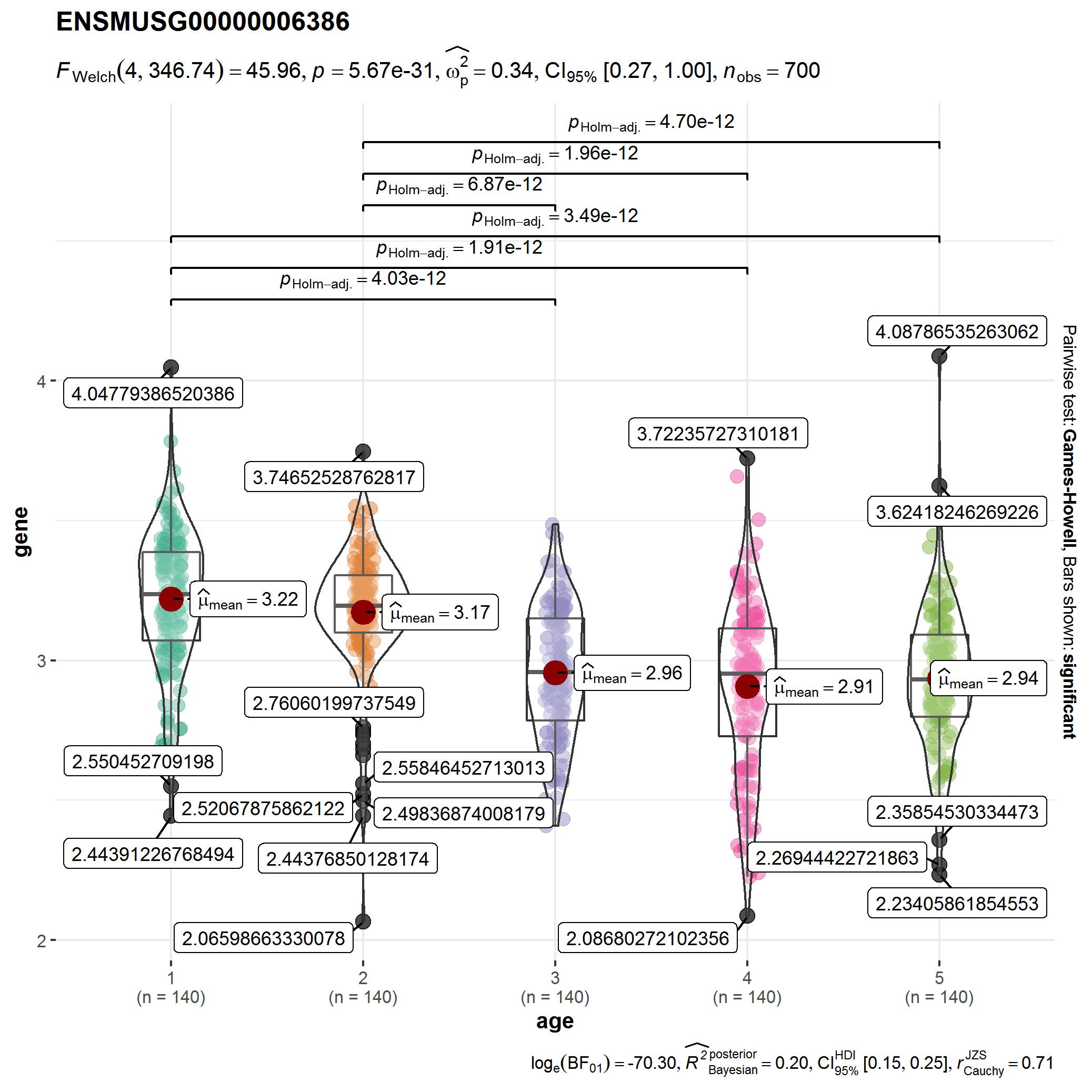

Supplement: Supplementary file 25 — Data S1–S6. [file ACEL-23-e14268-s017.zip › Data S1/ENSMUSG00000006386.jpeg]

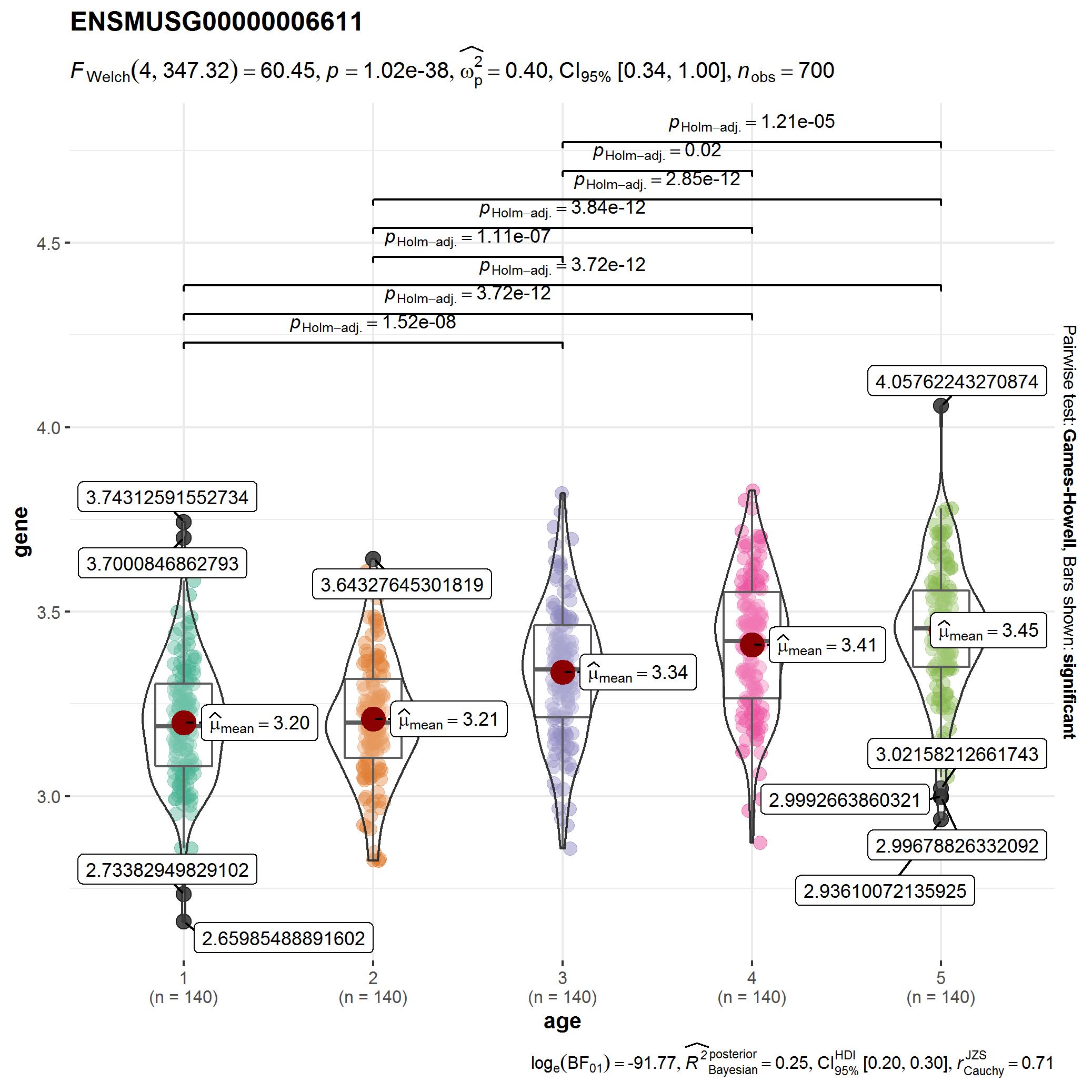

Supplement: Supplementary file 25 — Data S1–S6. [file ACEL-23-e14268-s017.zip › Data S1/ENSMUSG00000006611.jpeg]

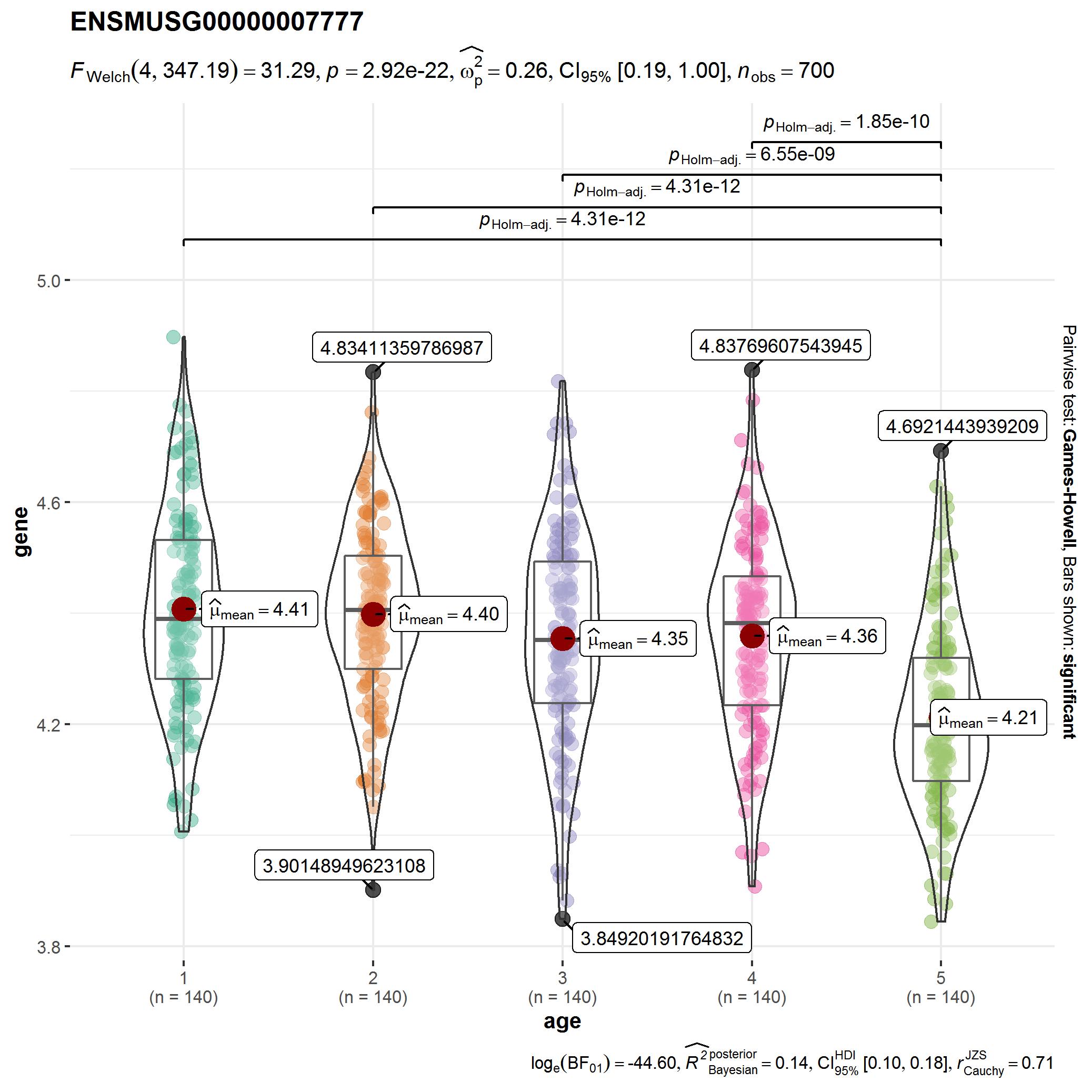

Supplement: Supplementary file 25 — Data S1–S6. [file ACEL-23-e14268-s017.zip › Data S1/ENSMUSG00000007777.jpeg]

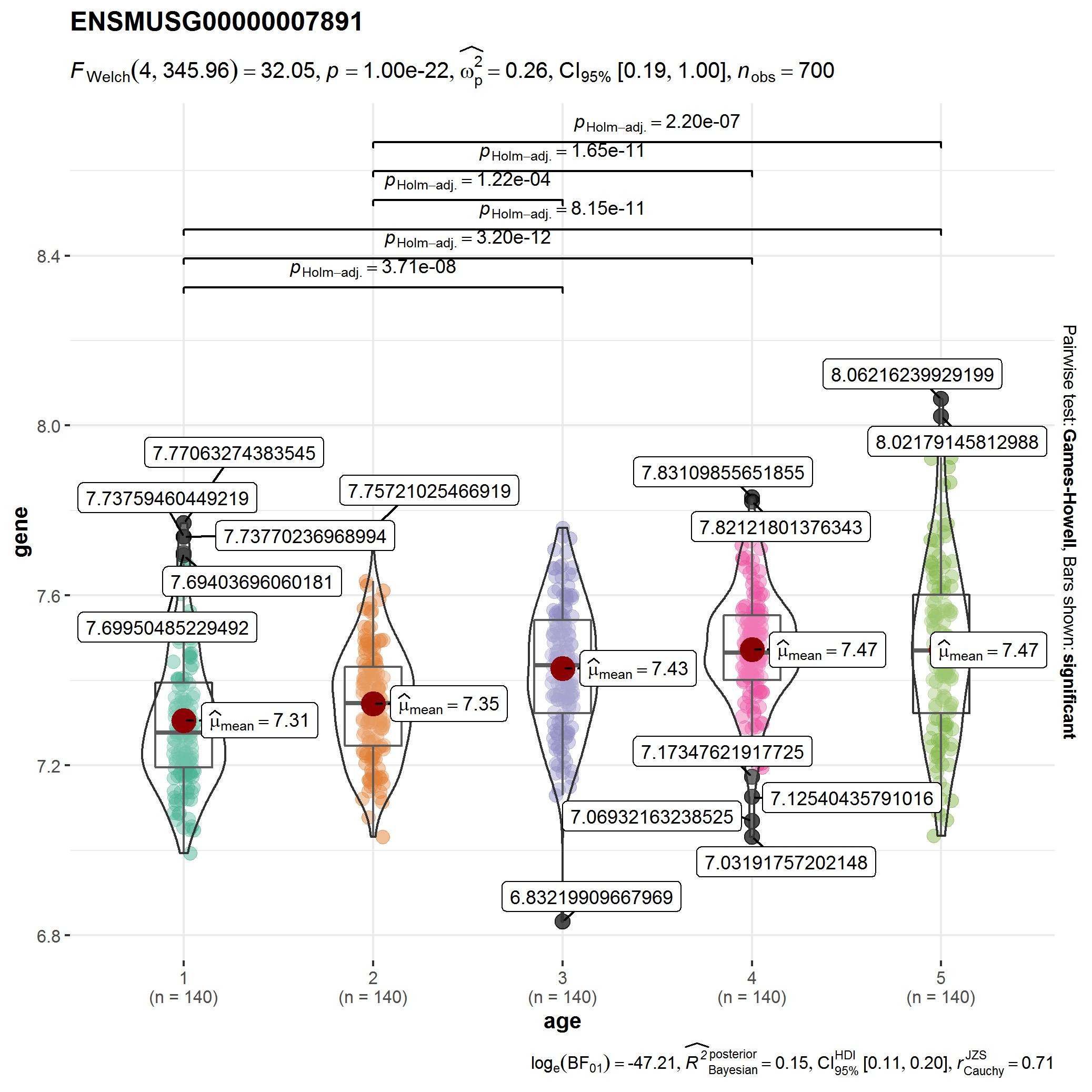

Supplement: Supplementary file 25 — Data S1–S6. [file ACEL-23-e14268-s017.zip › Data S1/ENSMUSG00000007891.jpeg]

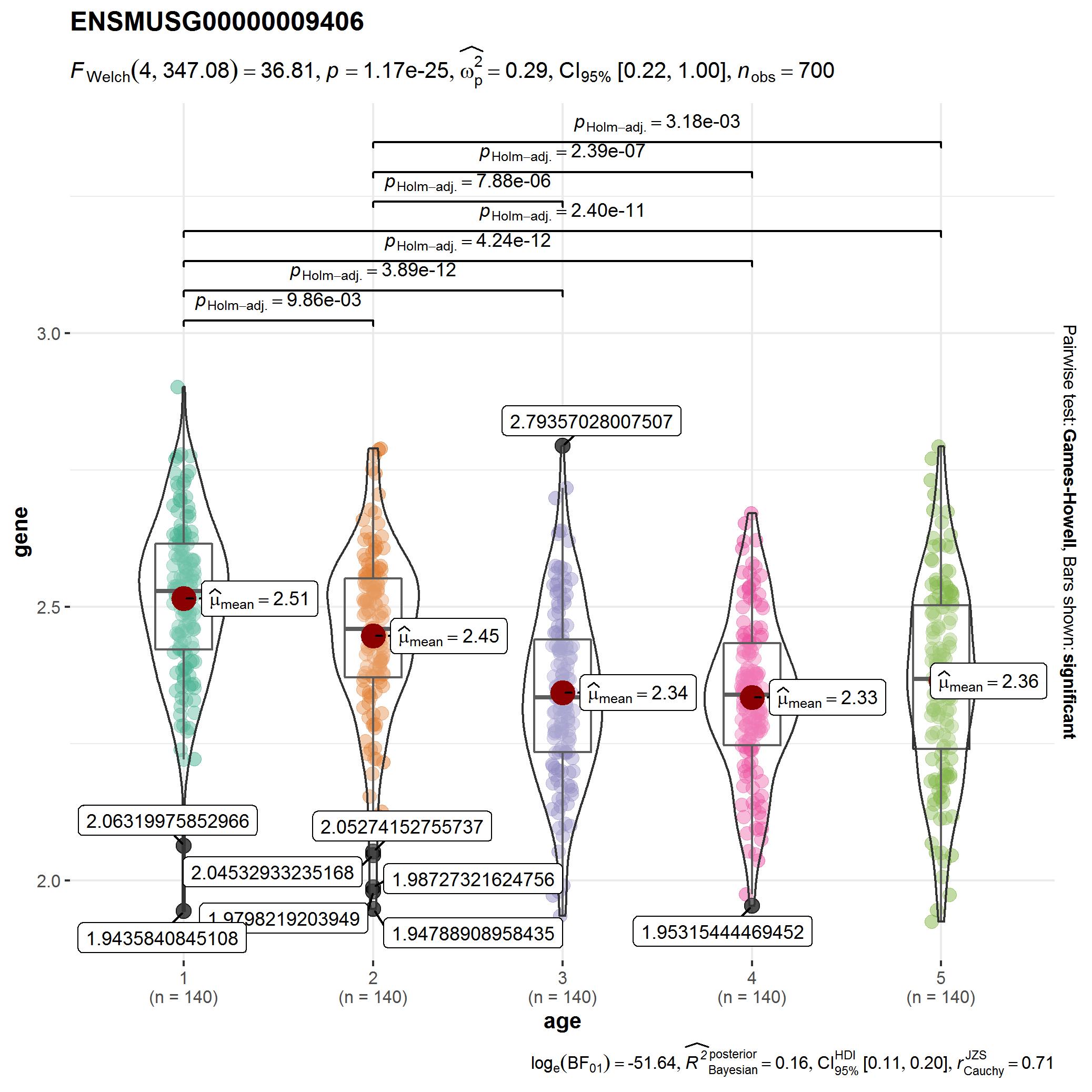

Supplement: Supplementary file 25 — Data S1–S6. [file ACEL-23-e14268-s017.zip › Data S1/ENSMUSG00000009406.jpeg]

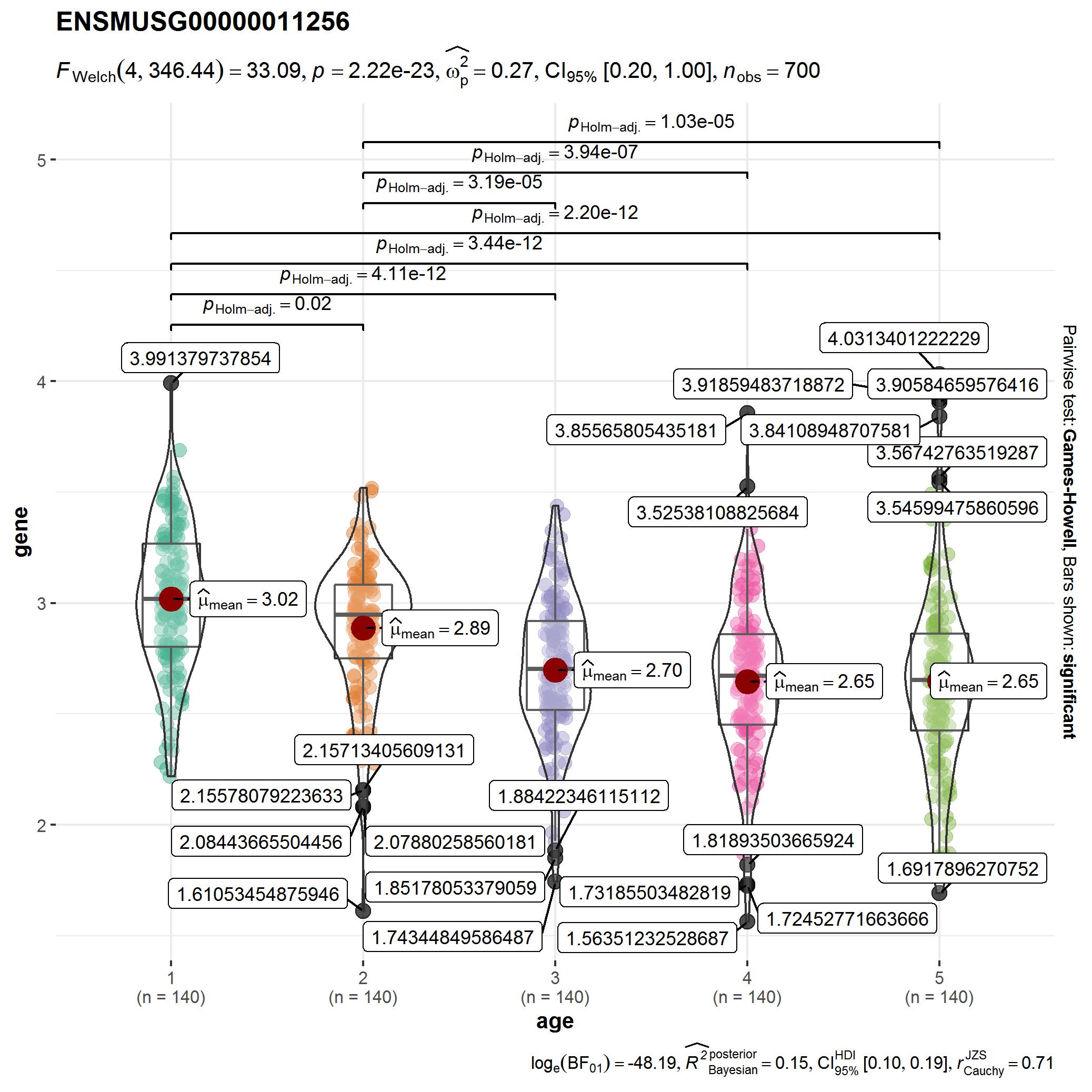

Supplement: Supplementary file 25 — Data S1–S6. [file ACEL-23-e14268-s017.zip › Data S1/ENSMUSG00000011256.jpeg]

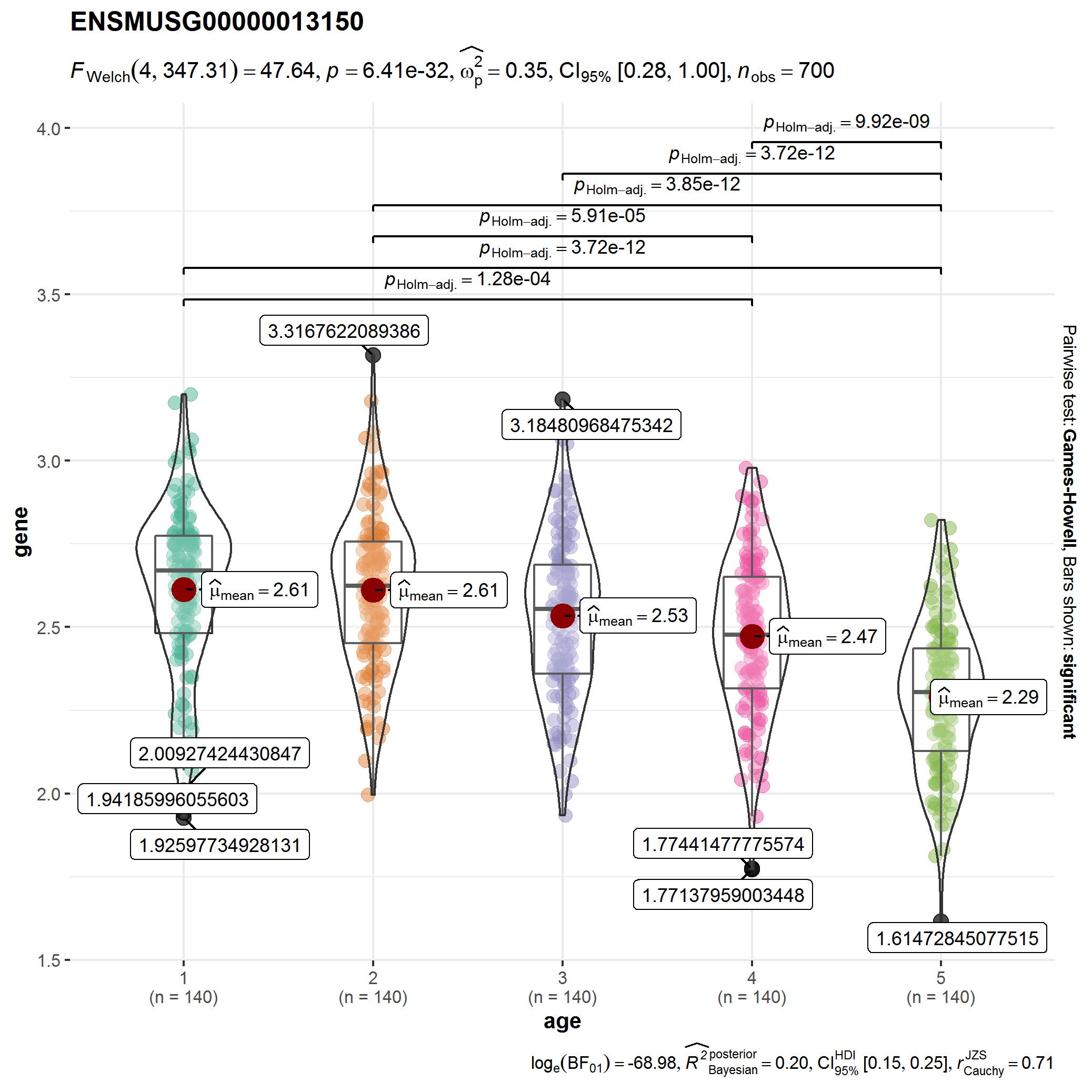

Supplement: Supplementary file 25 — Data S1–S6. [file ACEL-23-e14268-s017.zip › Data S1/ENSMUSG00000013150.jpeg]

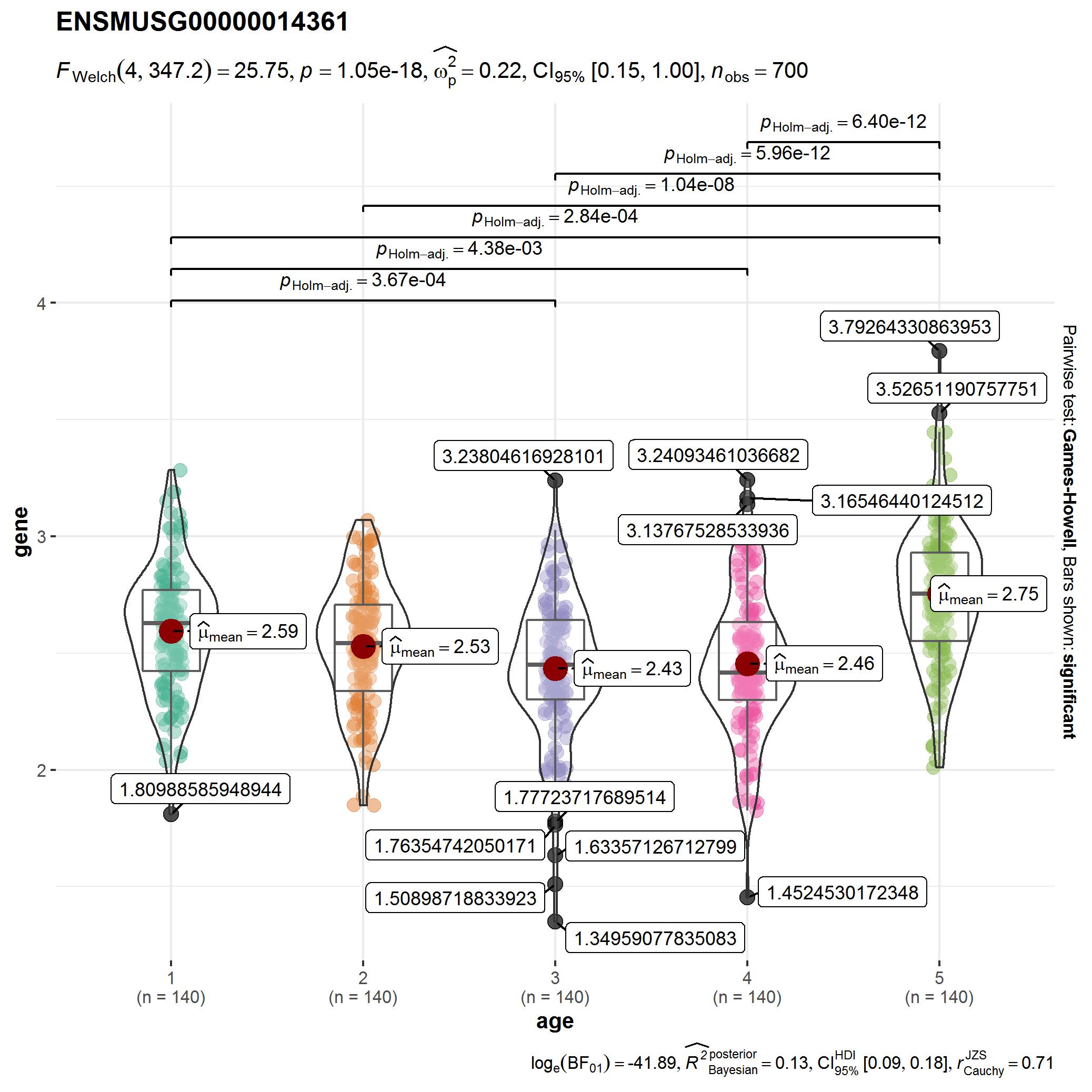

Supplement: Supplementary file 25 — Data S1–S6. [file ACEL-23-e14268-s017.zip › Data S1/ENSMUSG00000014361.jpeg]

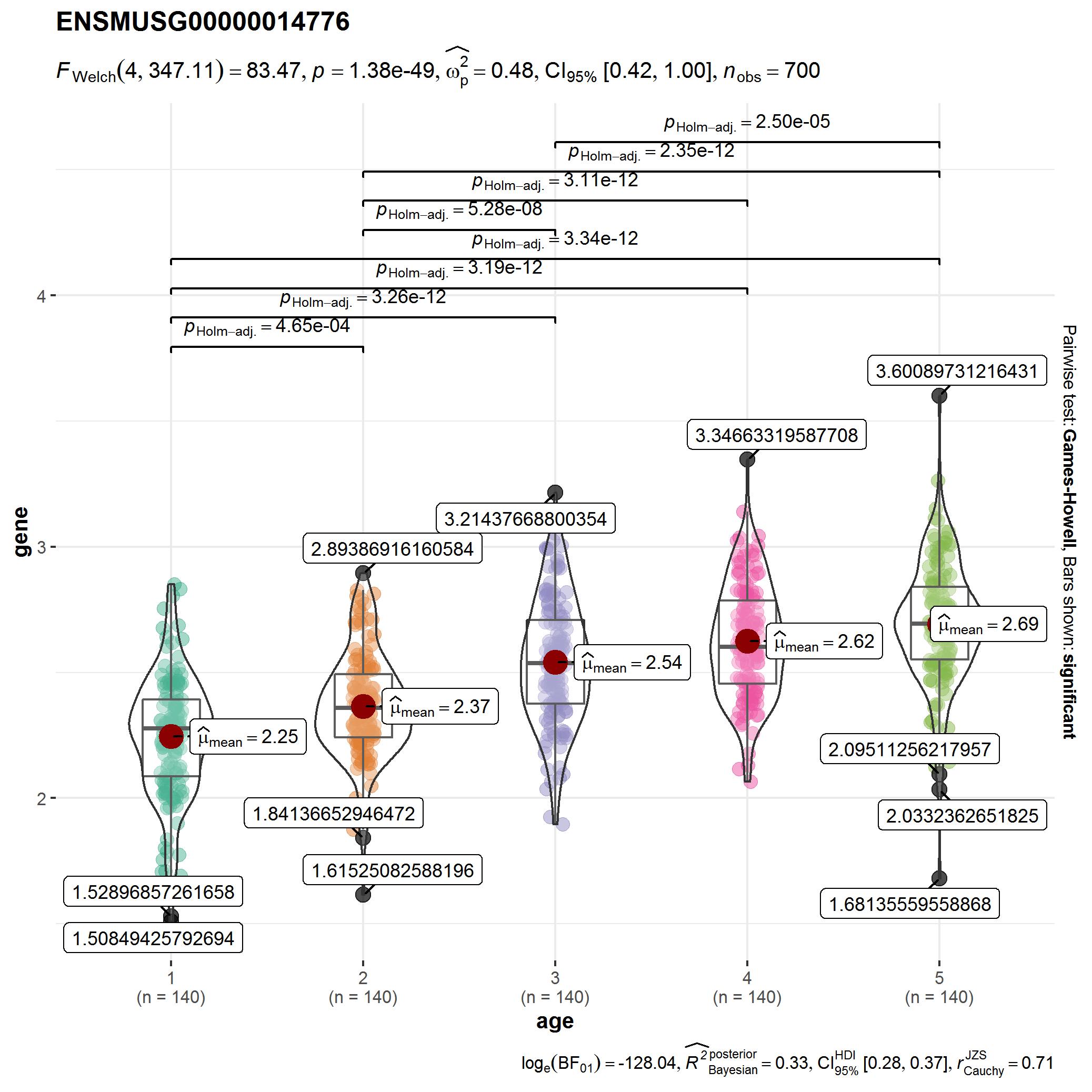

Supplement: Supplementary file 25 — Data S1–S6. [file ACEL-23-e14268-s017.zip › Data S1/ENSMUSG00000014776.jpeg]

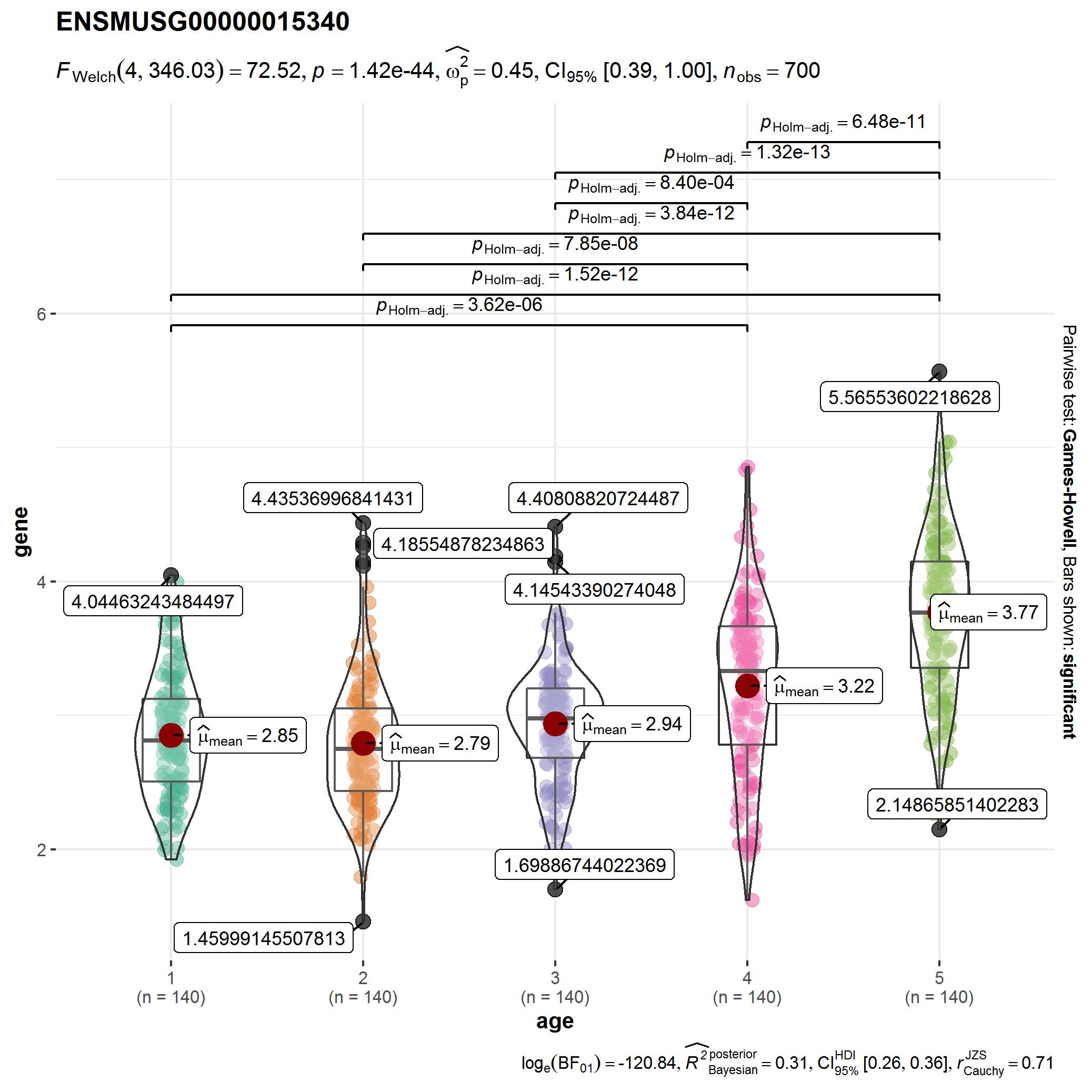

Supplement: Supplementary file 25 — Data S1–S6. [file ACEL-23-e14268-s017.zip › Data S1/ENSMUSG00000015340.jpeg]

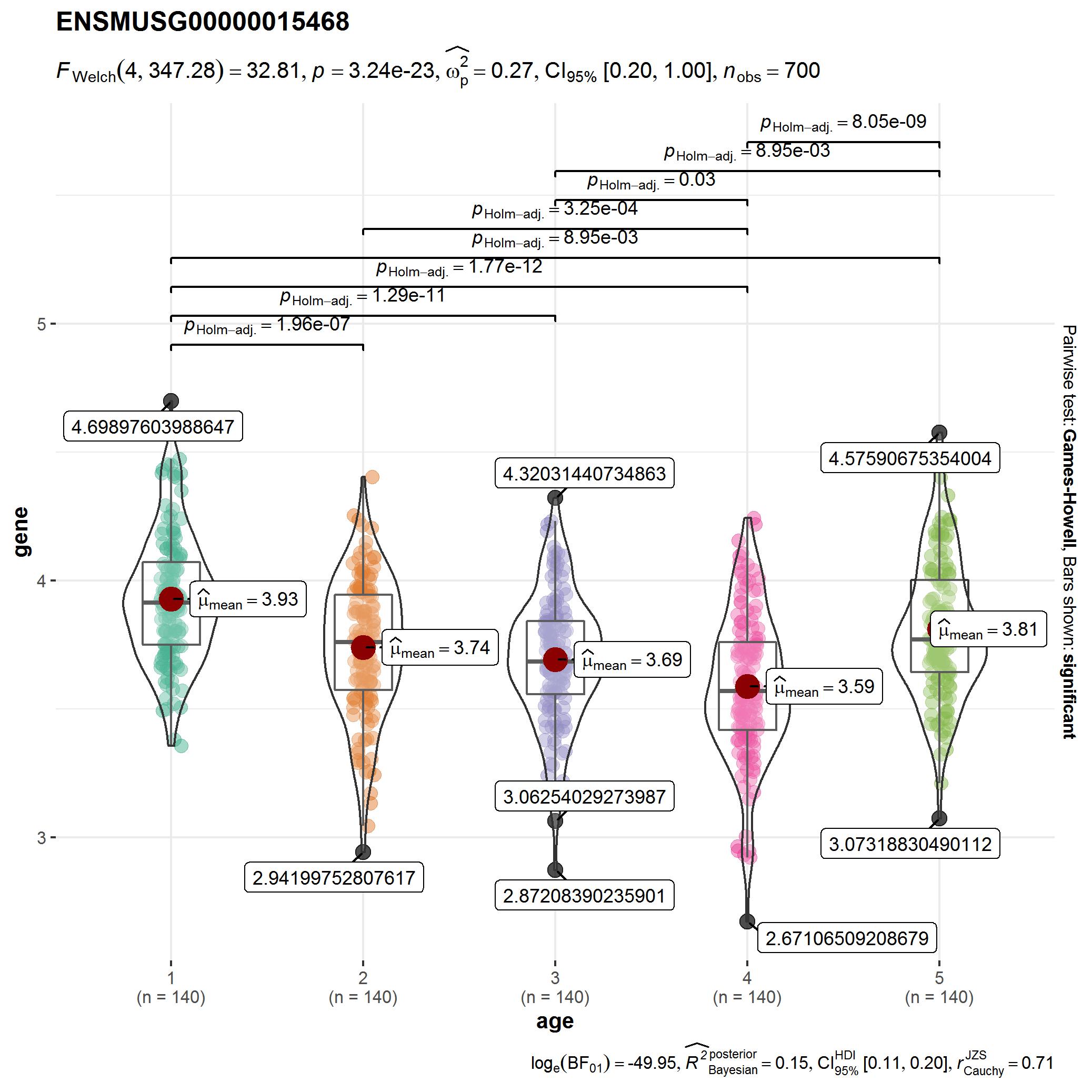

Supplement: Supplementary file 25 — Data S1–S6. [file ACEL-23-e14268-s017.zip › Data S1/ENSMUSG00000015468.jpeg]

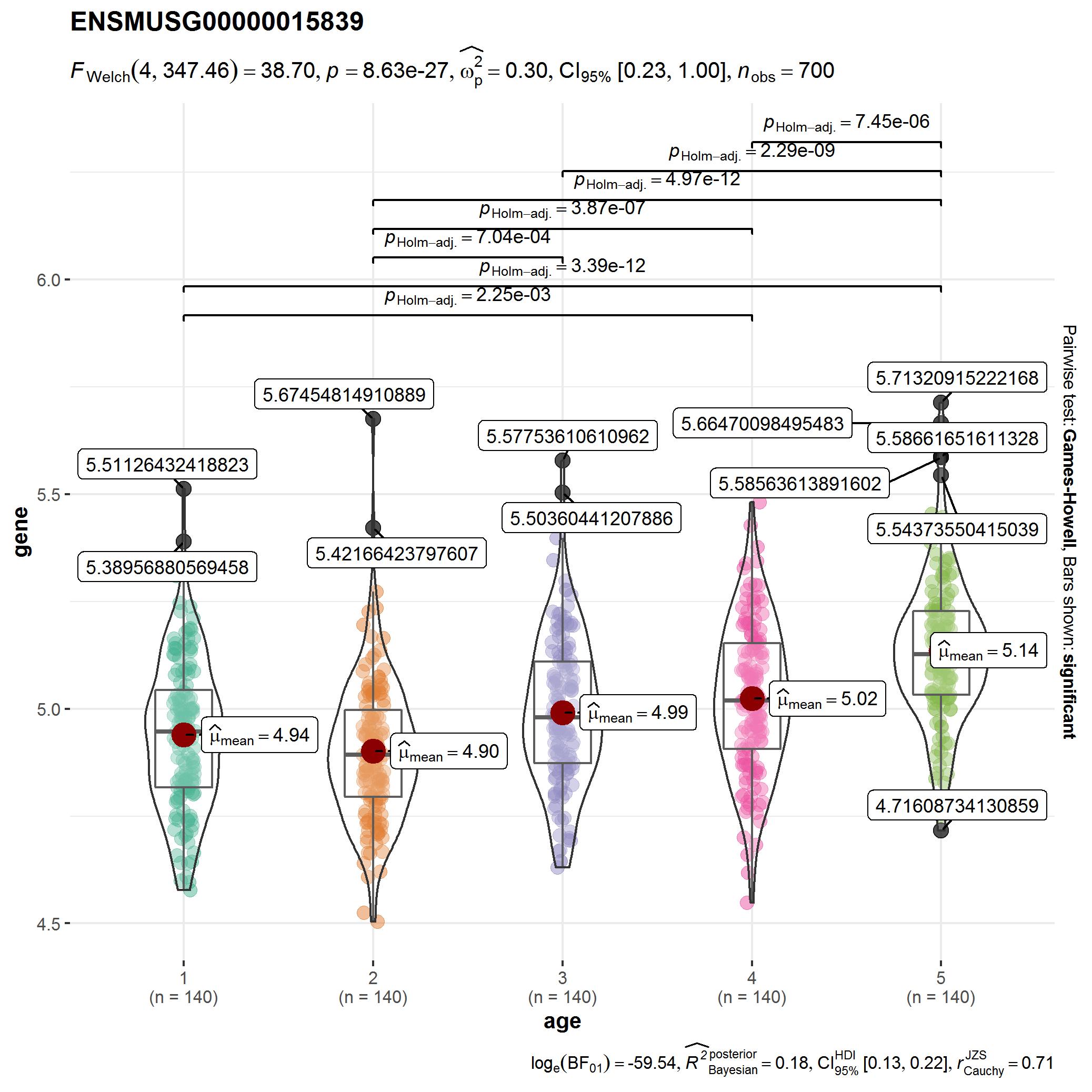

Supplement: Supplementary file 25 — Data S1–S6. [file ACEL-23-e14268-s017.zip › Data S1/ENSMUSG00000015839.jpeg]

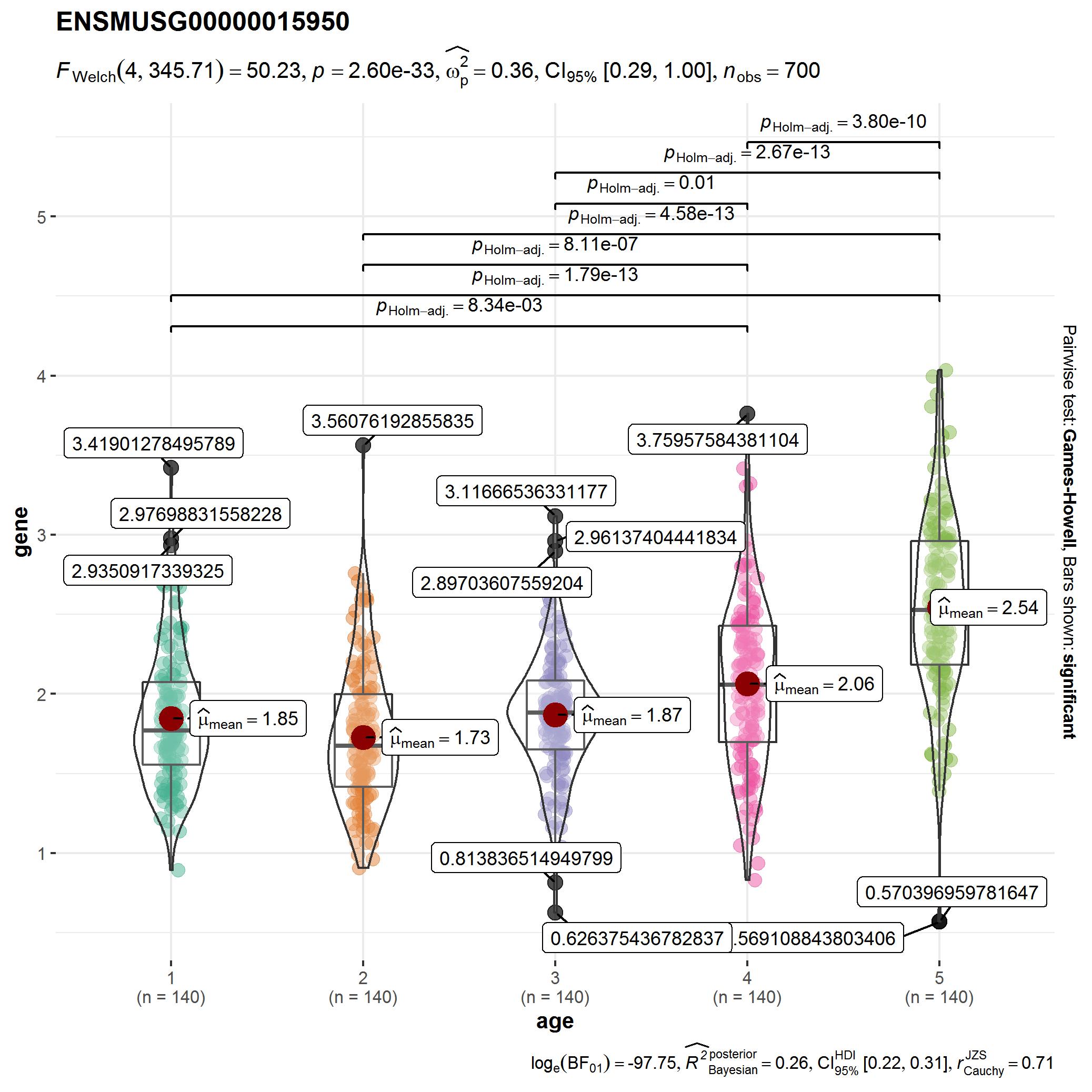

Supplement: Supplementary file 25 — Data S1–S6. [file ACEL-23-e14268-s017.zip › Data S1/ENSMUSG00000015950.jpeg]

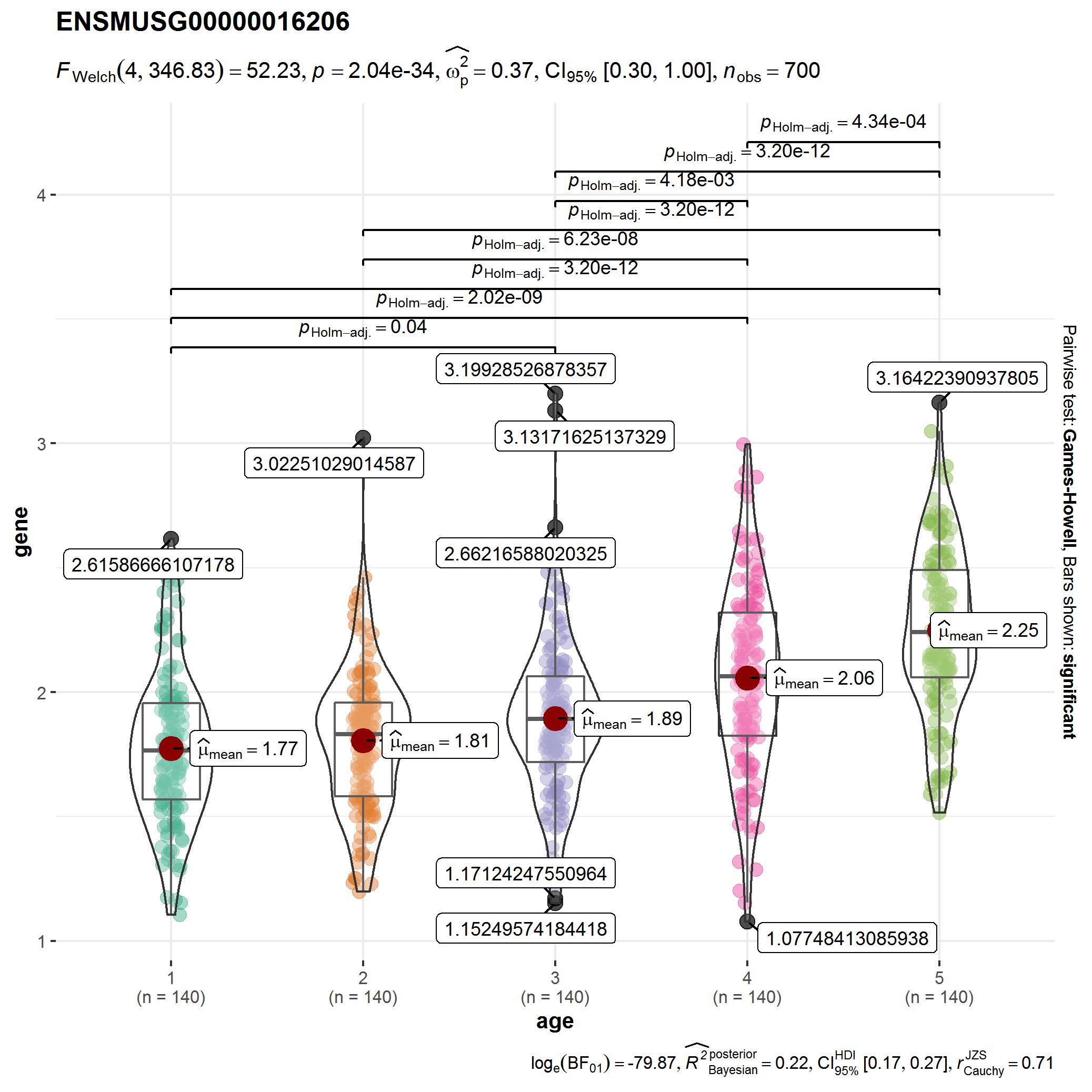

Supplement: Supplementary file 25 — Data S1–S6. [file ACEL-23-e14268-s017.zip › Data S1/ENSMUSG00000016206.jpeg]

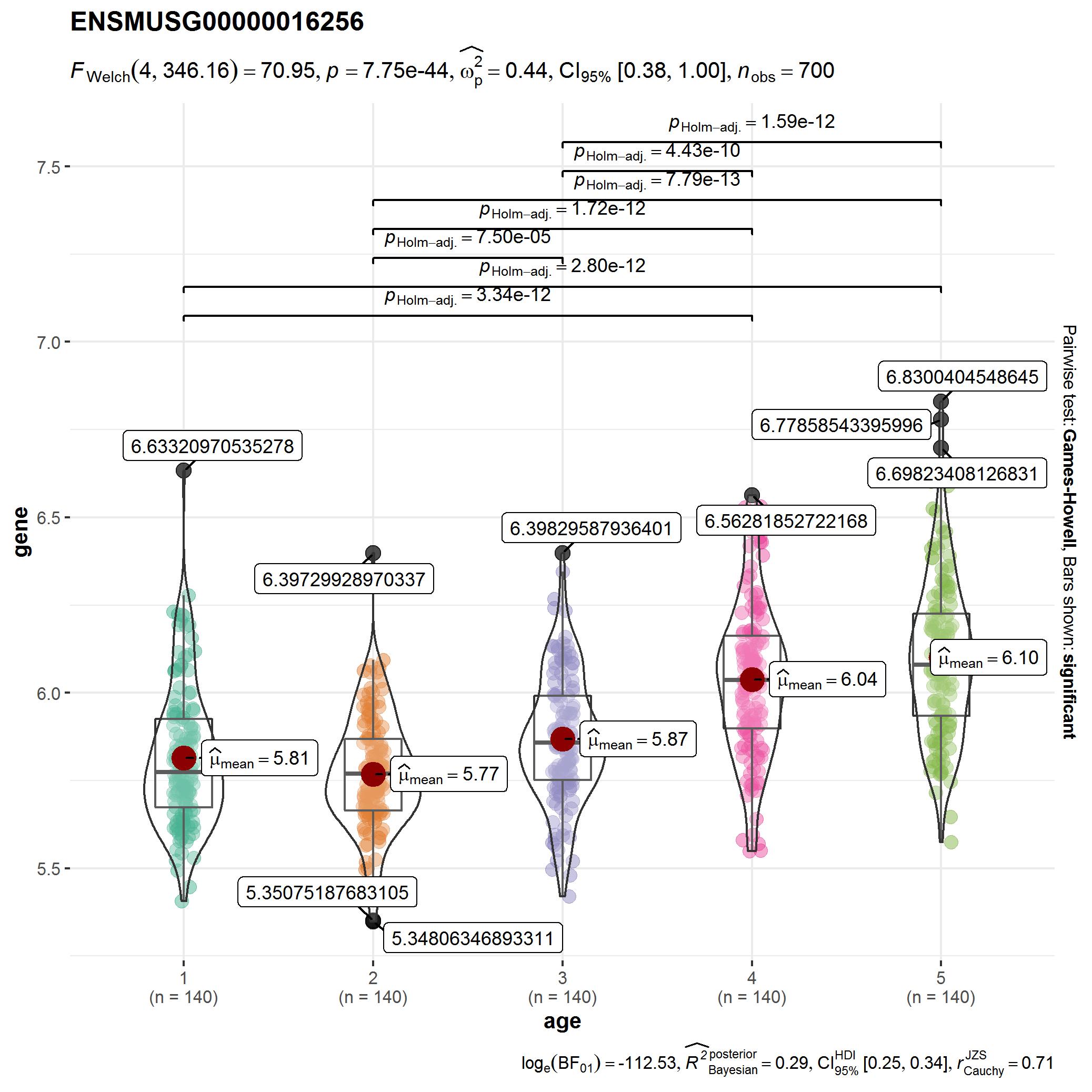

Supplement: Supplementary file 25 — Data S1–S6. [file ACEL-23-e14268-s017.zip › Data S1/ENSMUSG00000016256.jpeg]

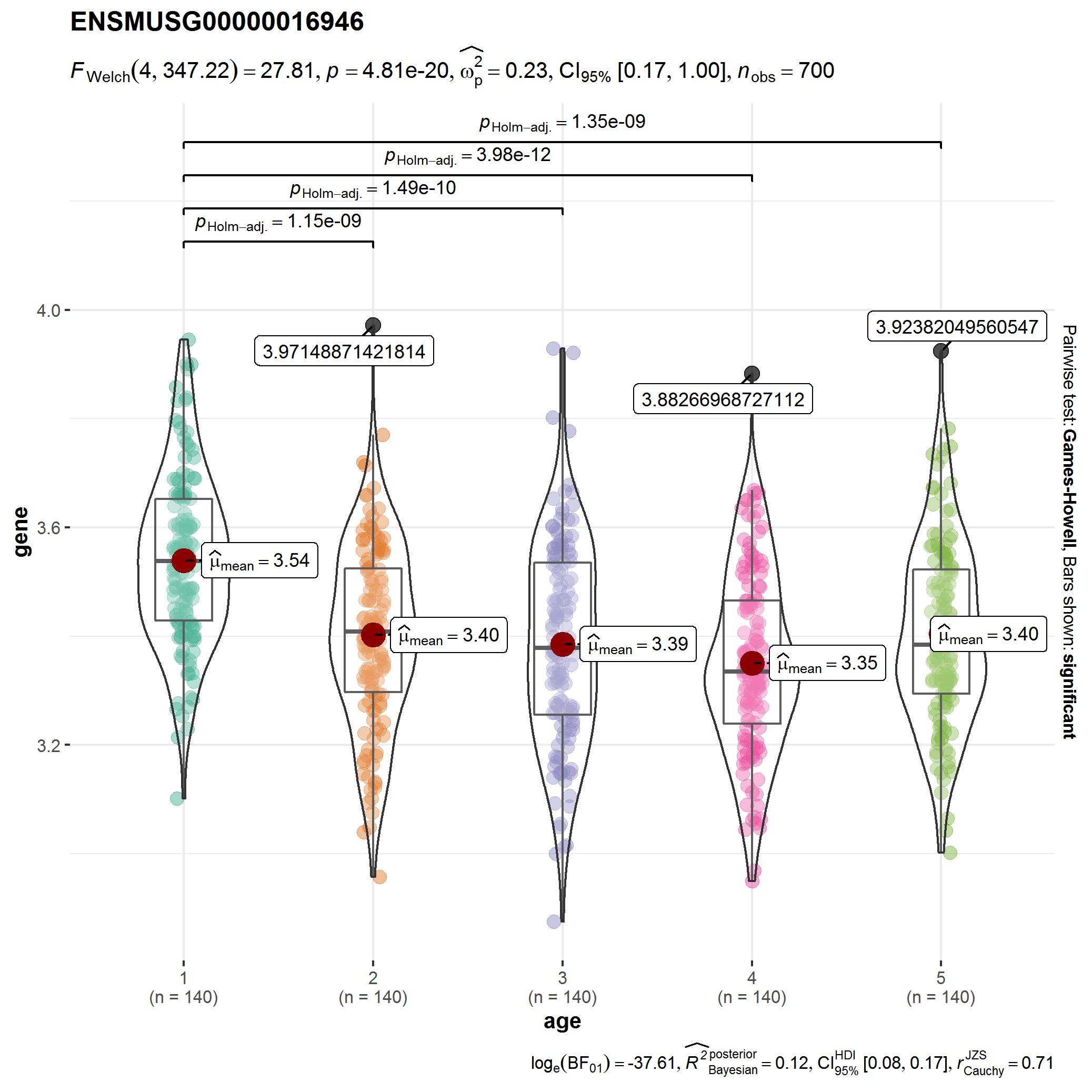

Supplement: Supplementary file 25 — Data S1–S6. [file ACEL-23-e14268-s017.zip › Data S1/ENSMUSG00000016946.jpeg]

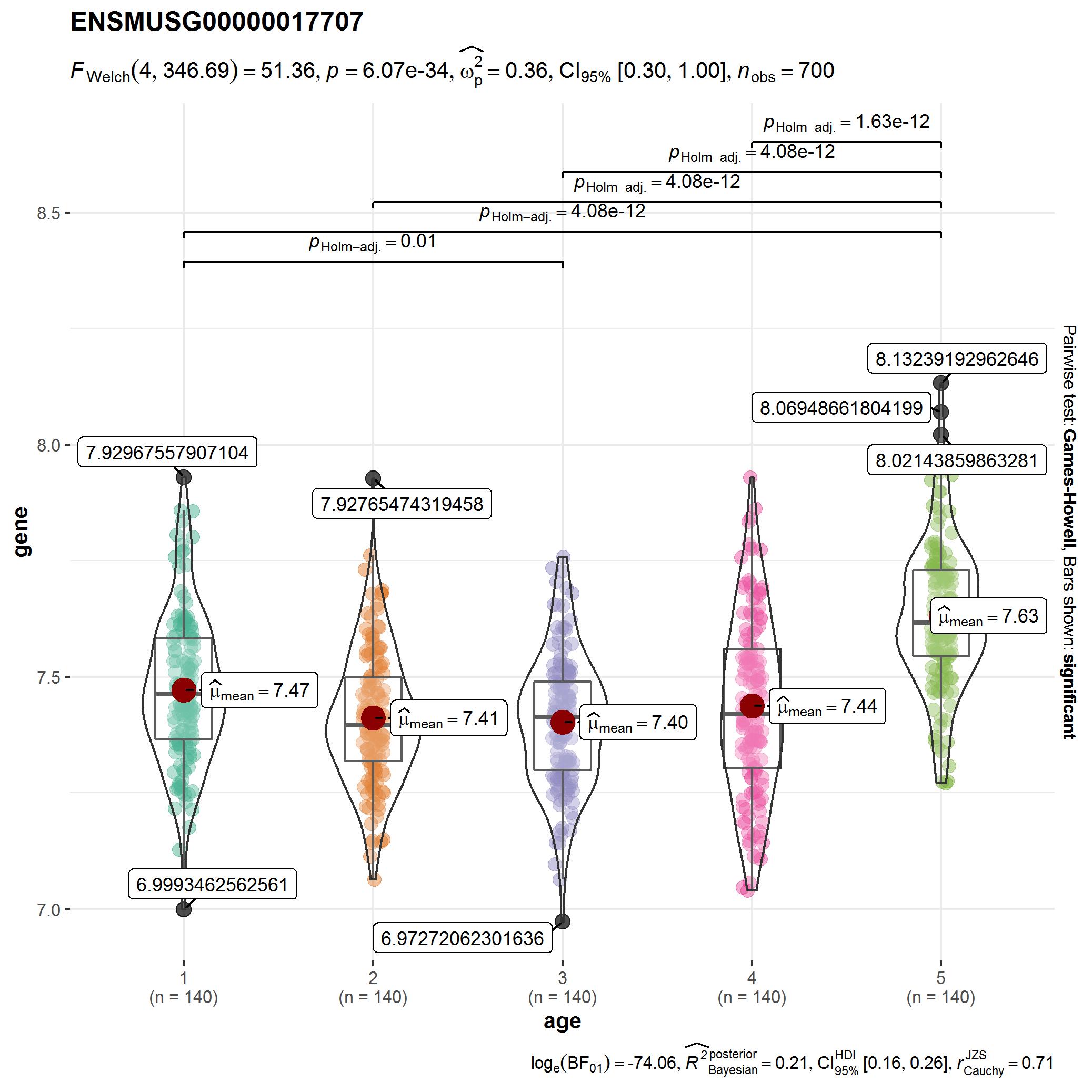

Supplement: Supplementary file 25 — Data S1–S6. [file ACEL-23-e14268-s017.zip › Data S1/ENSMUSG00000017707.jpeg]

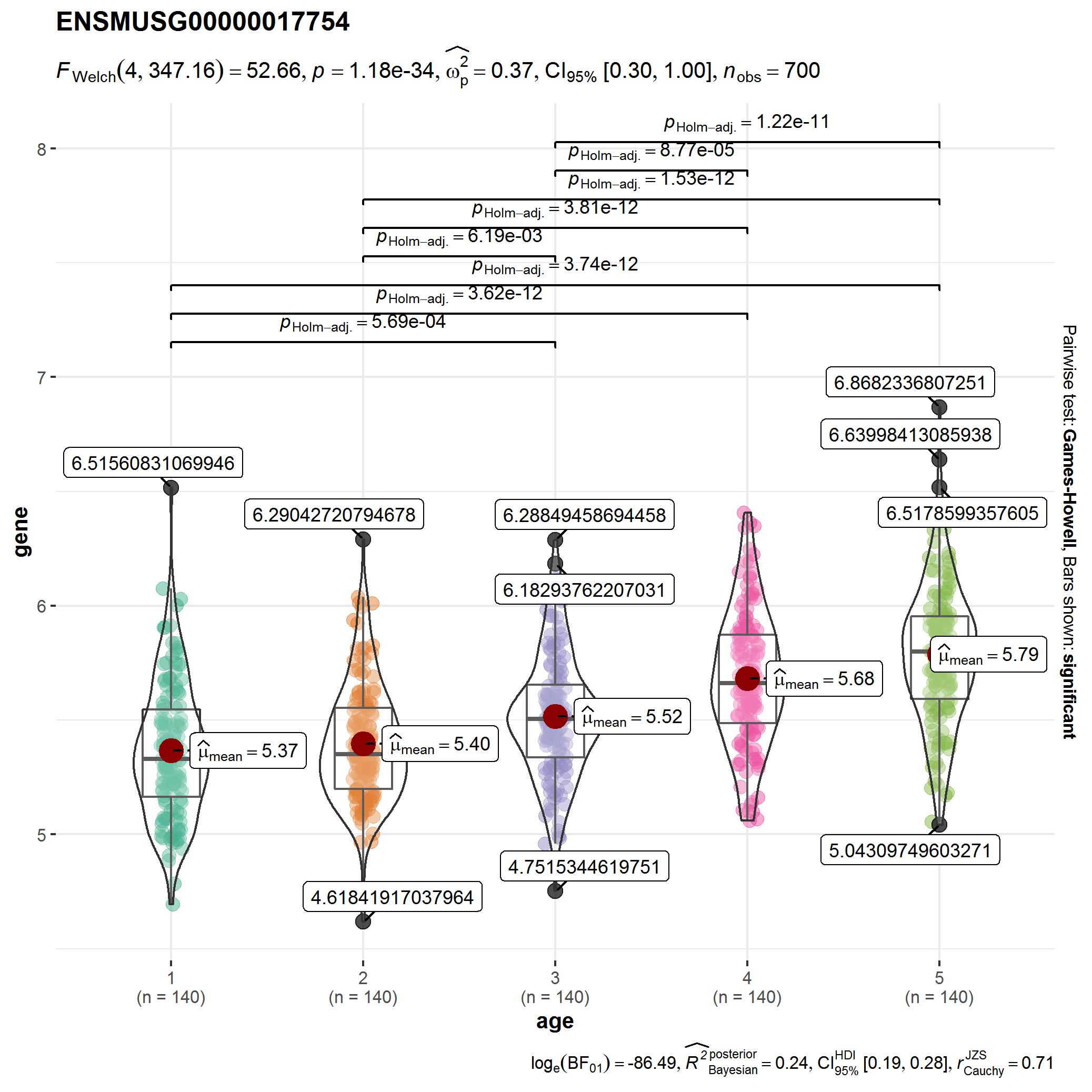

Supplement: Supplementary file 25 — Data S1–S6. [file ACEL-23-e14268-s017.zip › Data S1/ENSMUSG00000017754.jpeg]

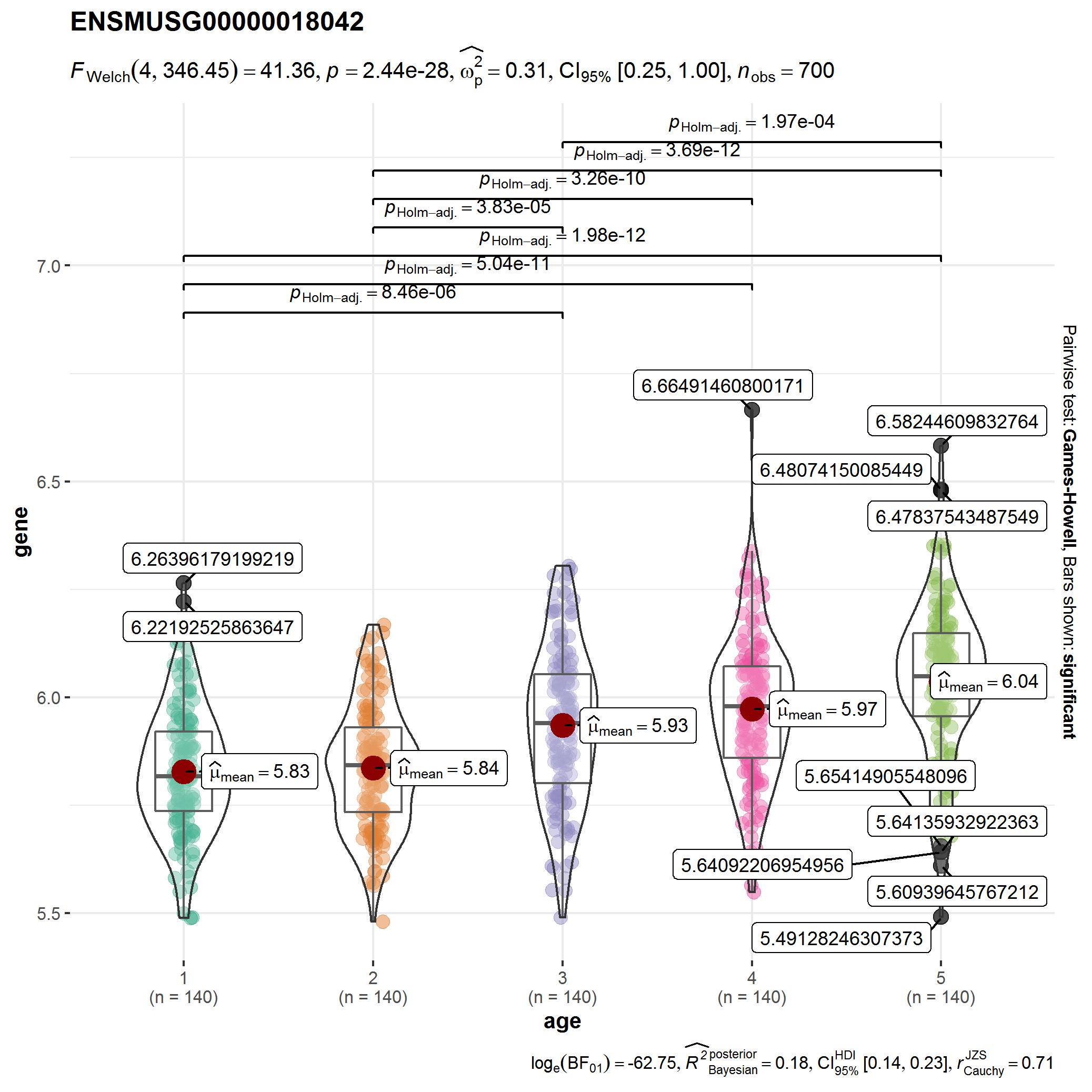

Supplement: Supplementary file 25 — Data S1–S6. [file ACEL-23-e14268-s017.zip › Data S1/ENSMUSG00000018042.jpeg]

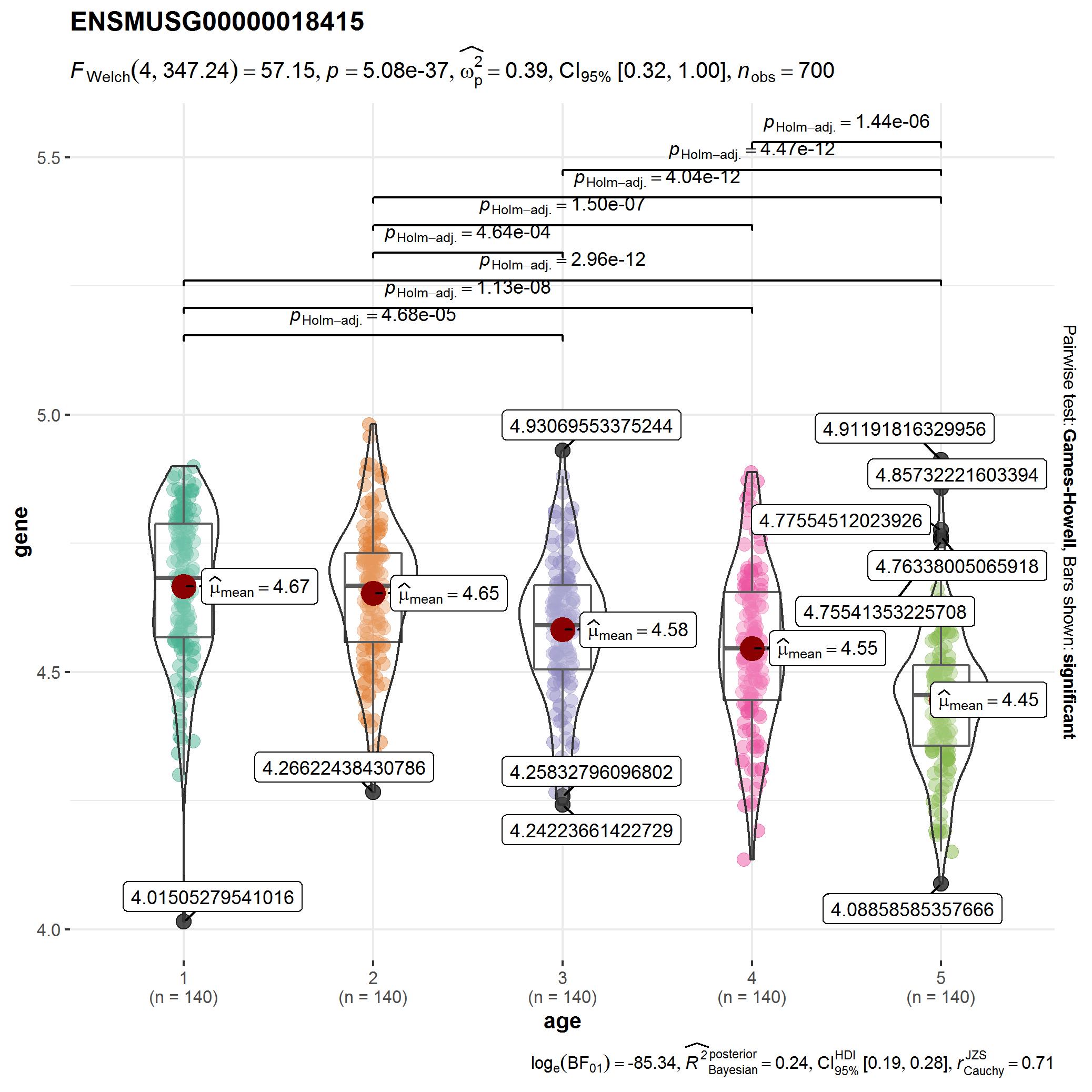

Supplement: Supplementary file 25 — Data S1–S6. [file ACEL-23-e14268-s017.zip › Data S1/ENSMUSG00000018415.jpeg]

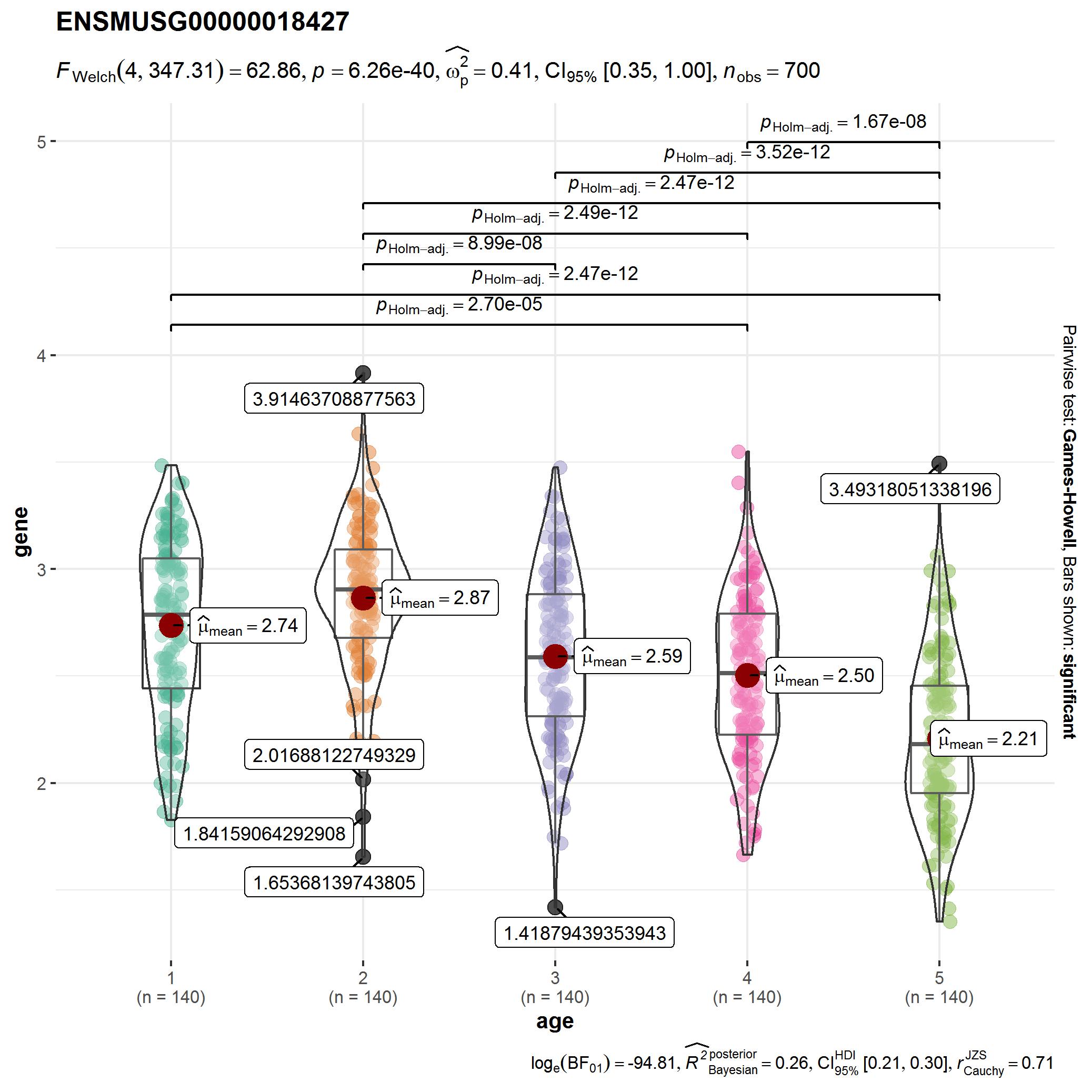

Supplement: Supplementary file 25 — Data S1–S6. [file ACEL-23-e14268-s017.zip › Data S1/ENSMUSG00000018427.jpeg]

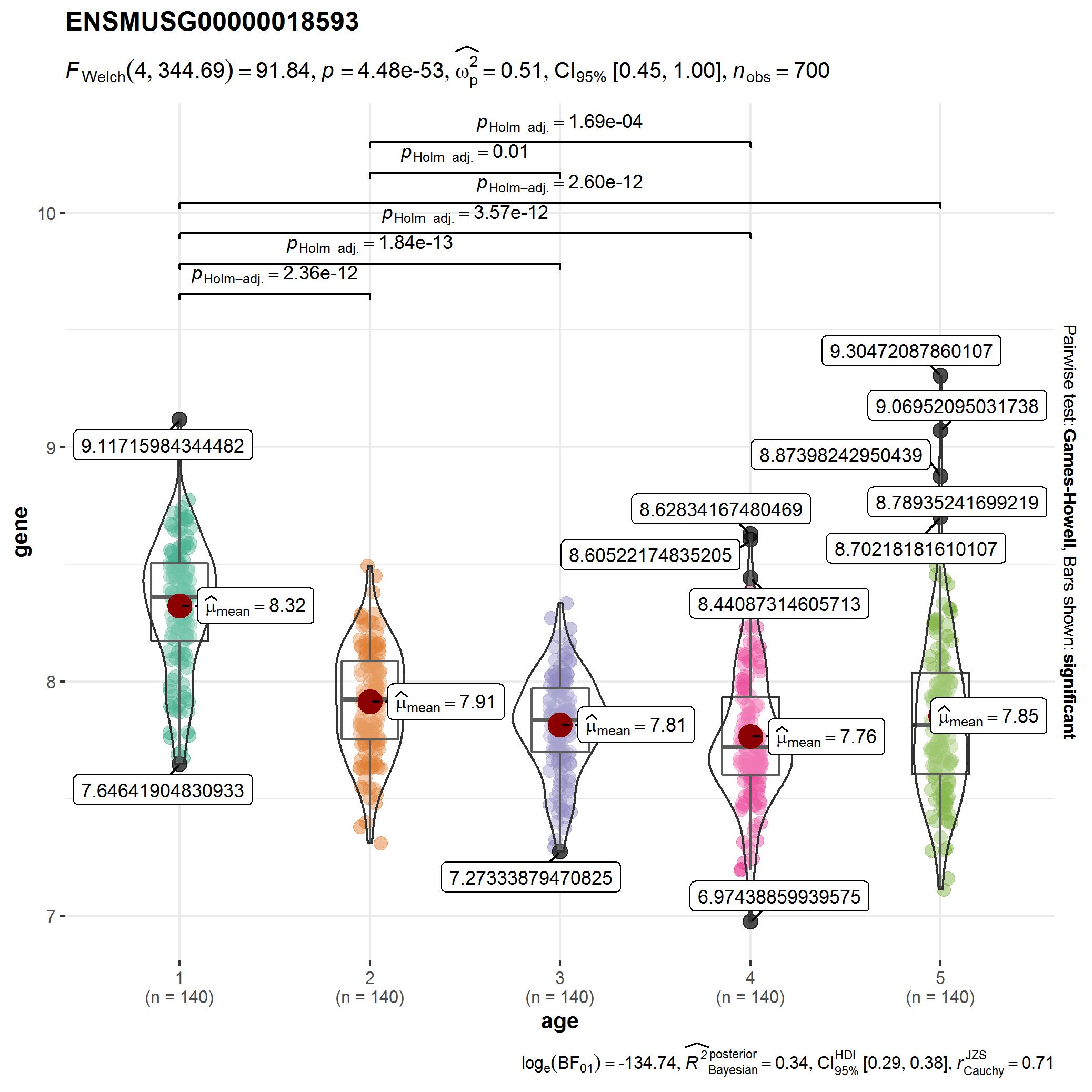

Supplement: Supplementary file 25 — Data S1–S6. [file ACEL-23-e14268-s017.zip › Data S1/ENSMUSG00000018593.jpeg]

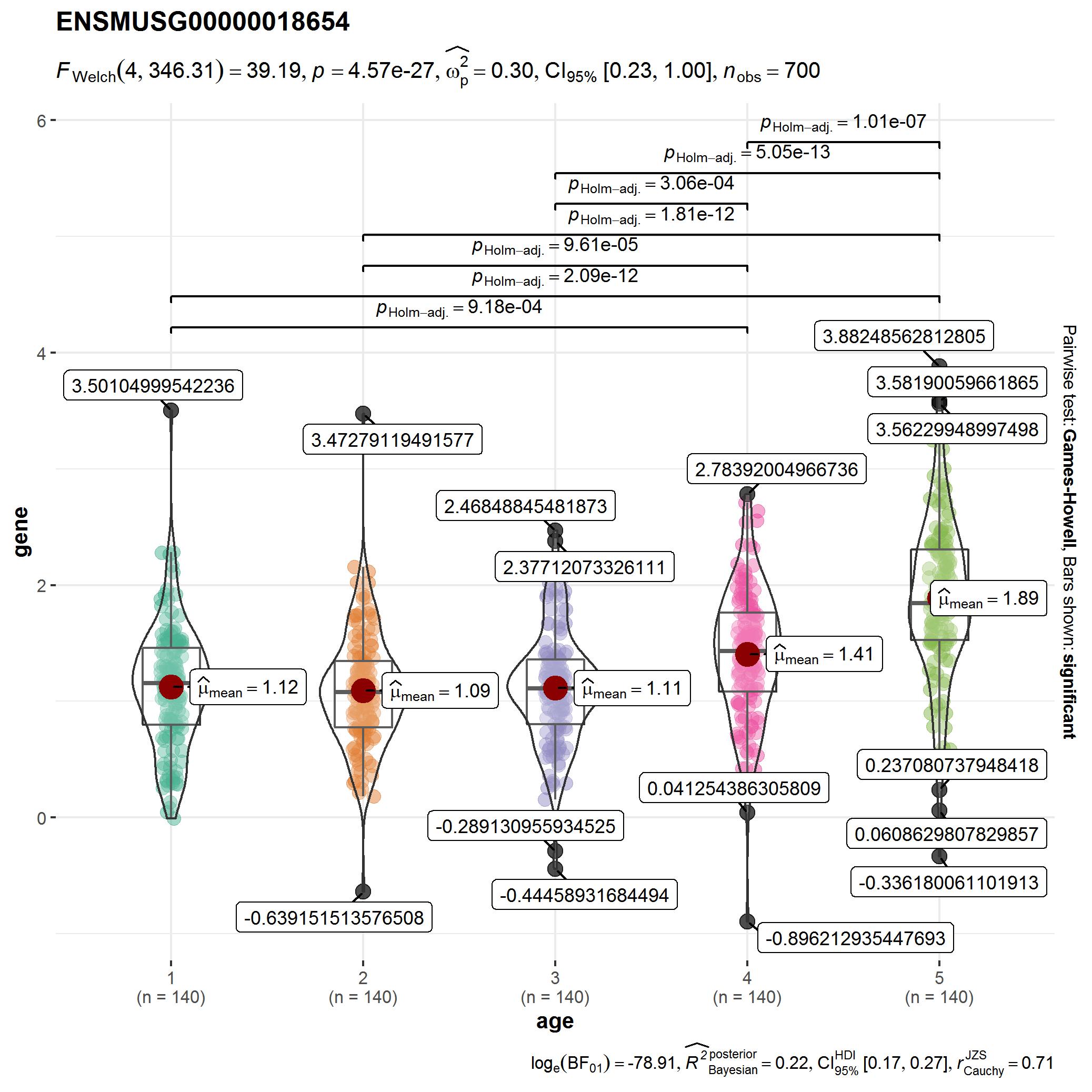

Supplement: Supplementary file 25 — Data S1–S6. [file ACEL-23-e14268-s017.zip › Data S1/ENSMUSG00000018654.jpeg]

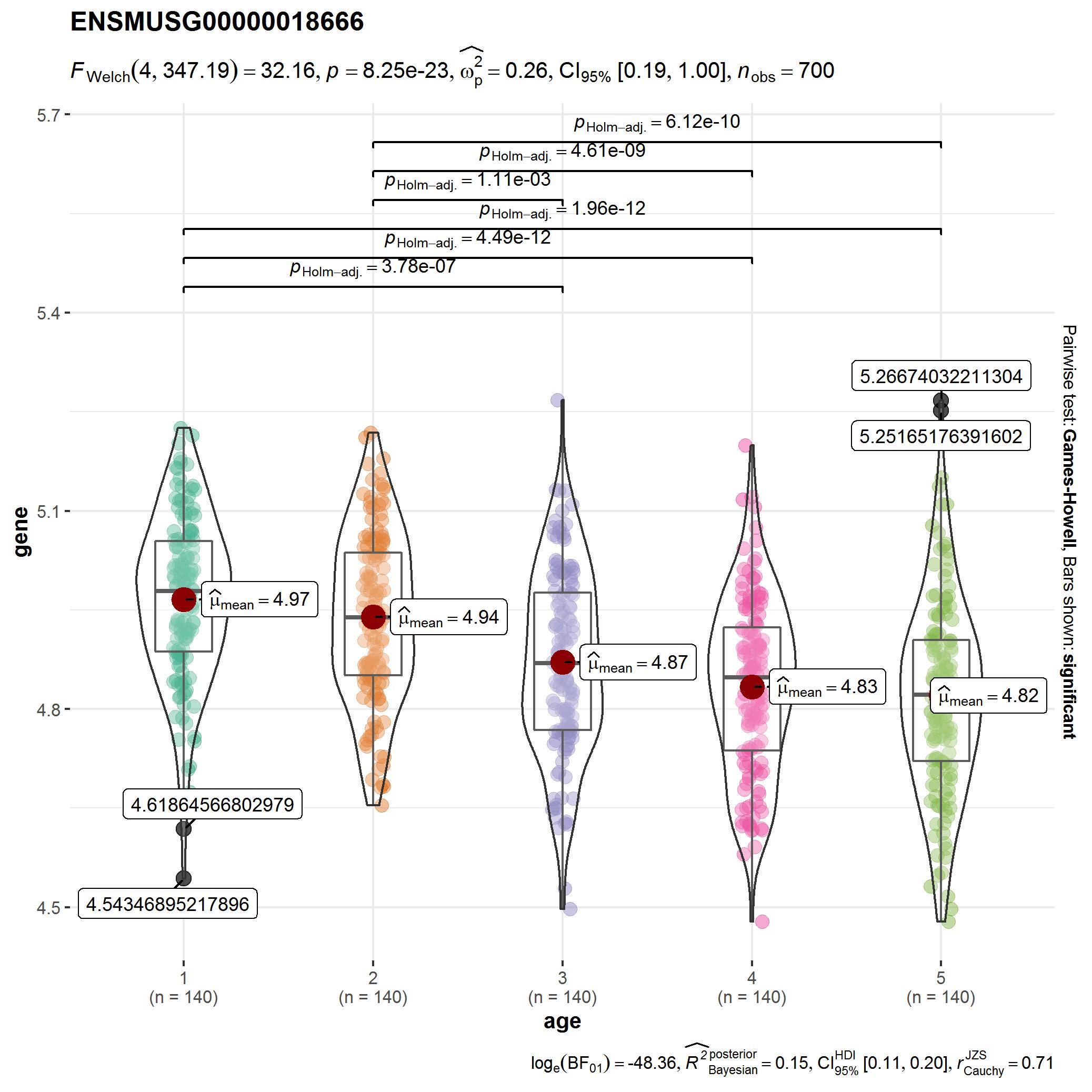

Supplement: Supplementary file 25 — Data S1–S6. [file ACEL-23-e14268-s017.zip › Data S1/ENSMUSG00000018666.jpeg]

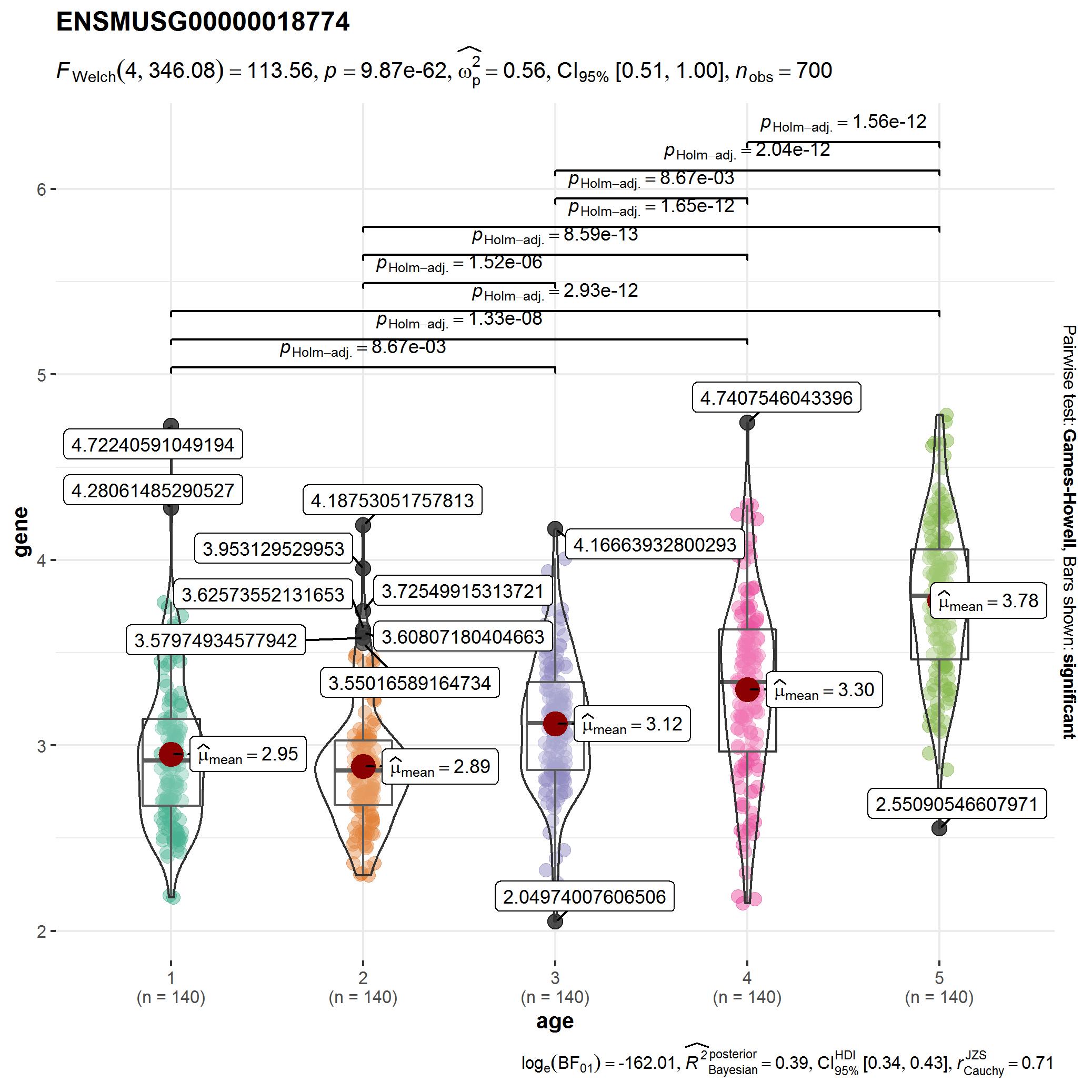

Supplement: Supplementary file 25 — Data S1–S6. [file ACEL-23-e14268-s017.zip › Data S1/ENSMUSG00000018774.jpeg]

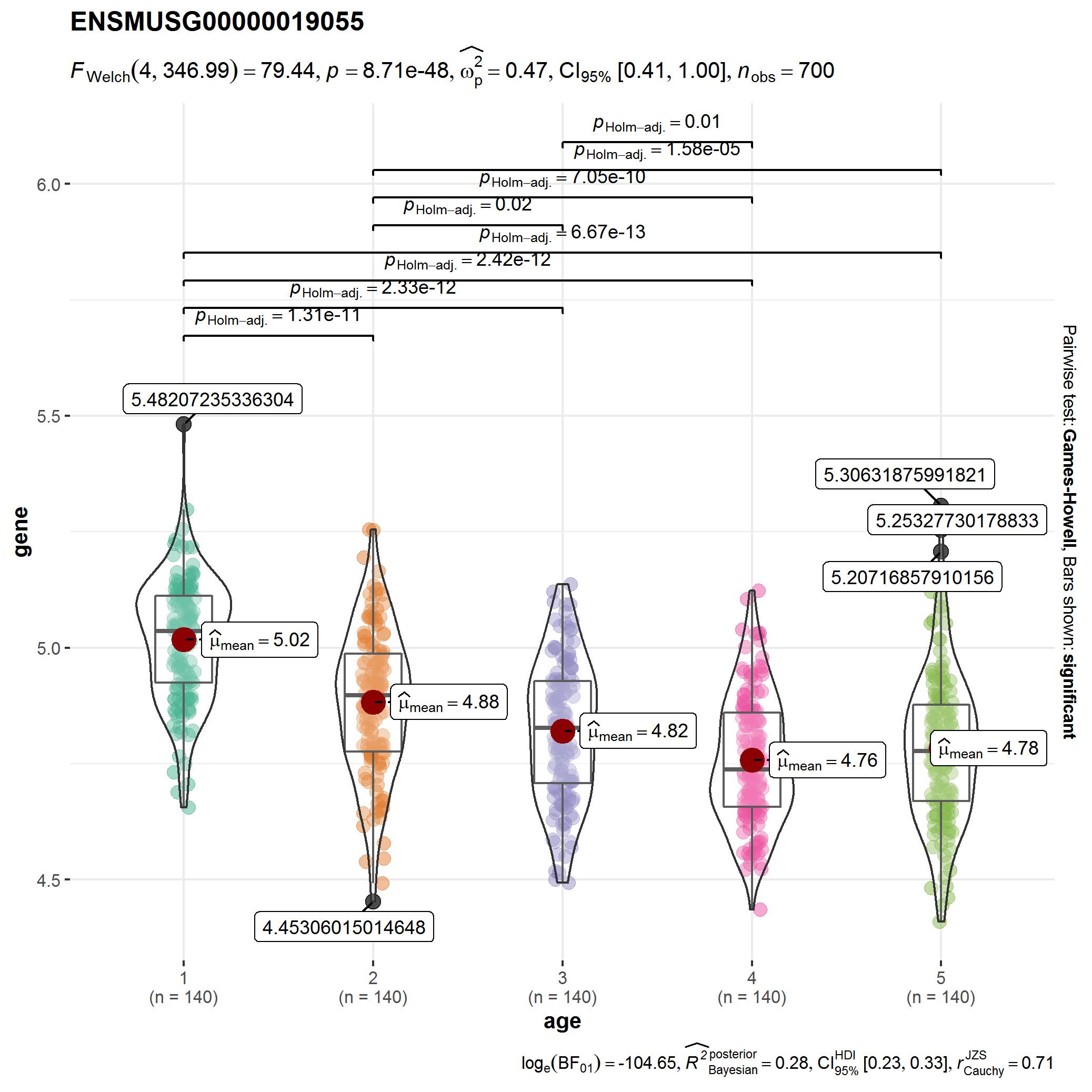

Supplement: Supplementary file 25 — Data S1–S6. [file ACEL-23-e14268-s017.zip › Data S1/ENSMUSG00000019055.jpeg]

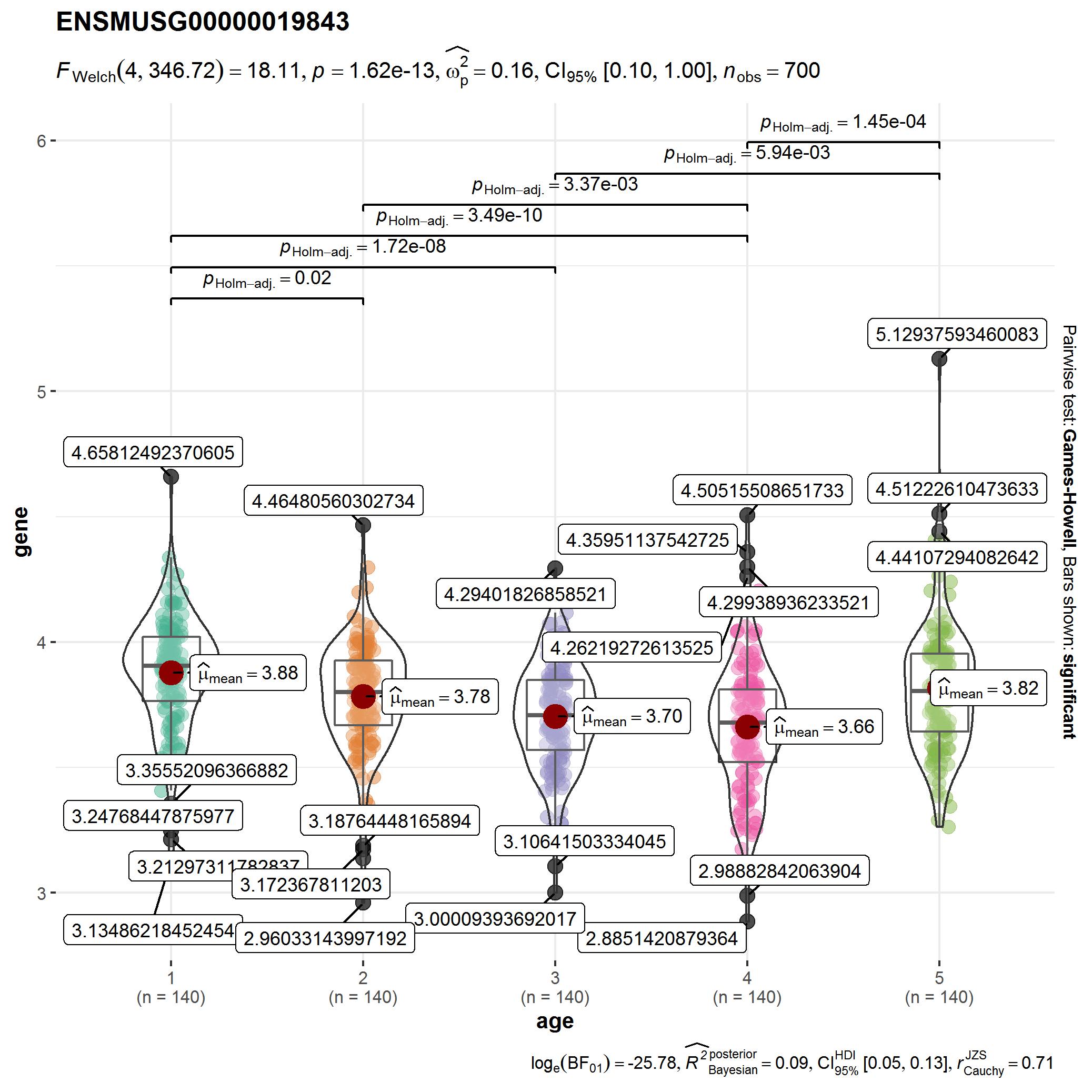

Supplement: Supplementary file 25 — Data S1–S6. [file ACEL-23-e14268-s017.zip › Data S1/ENSMUSG00000019843.jpeg]

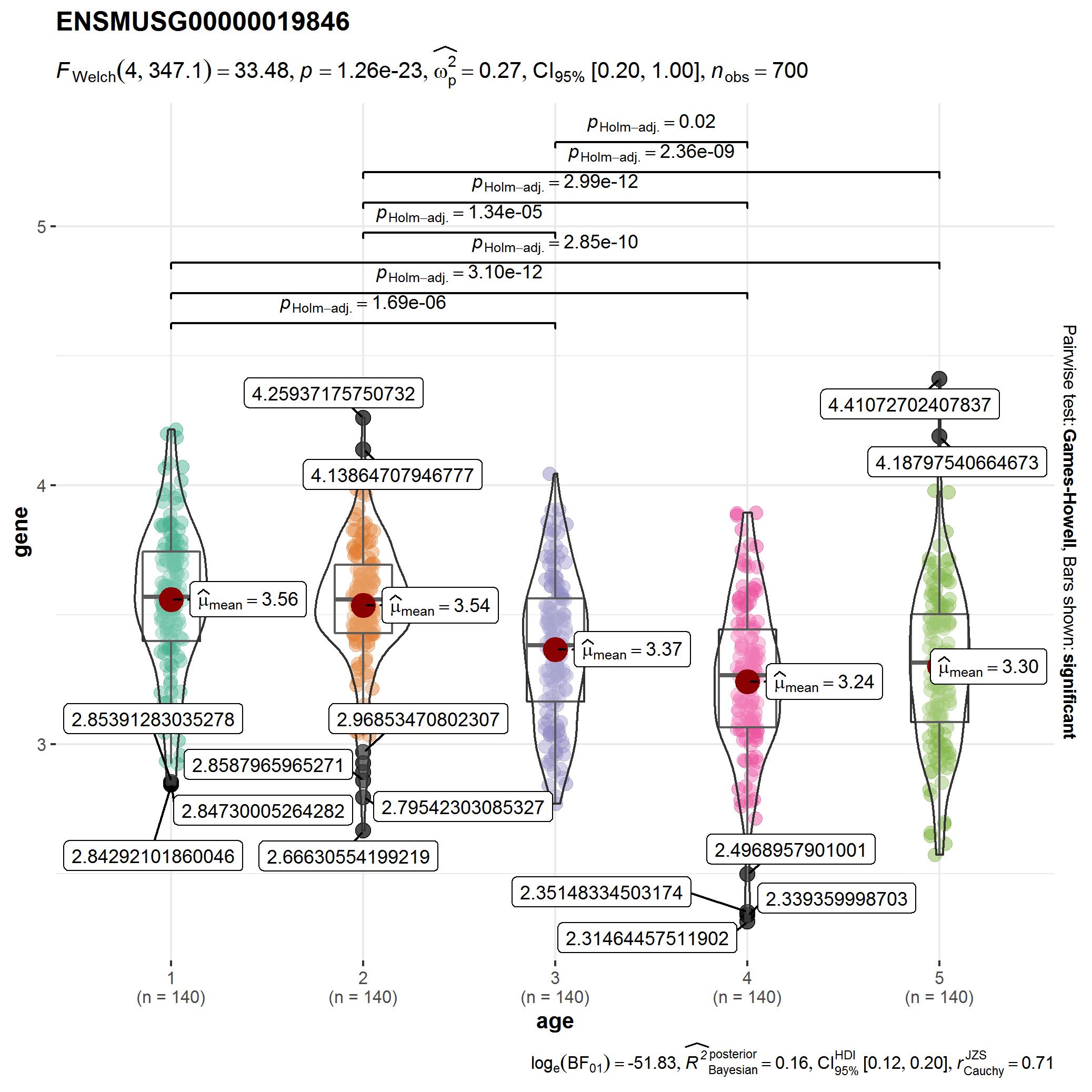

Supplement: Supplementary file 25 — Data S1–S6. [file ACEL-23-e14268-s017.zip › Data S1/ENSMUSG00000019846.jpeg]

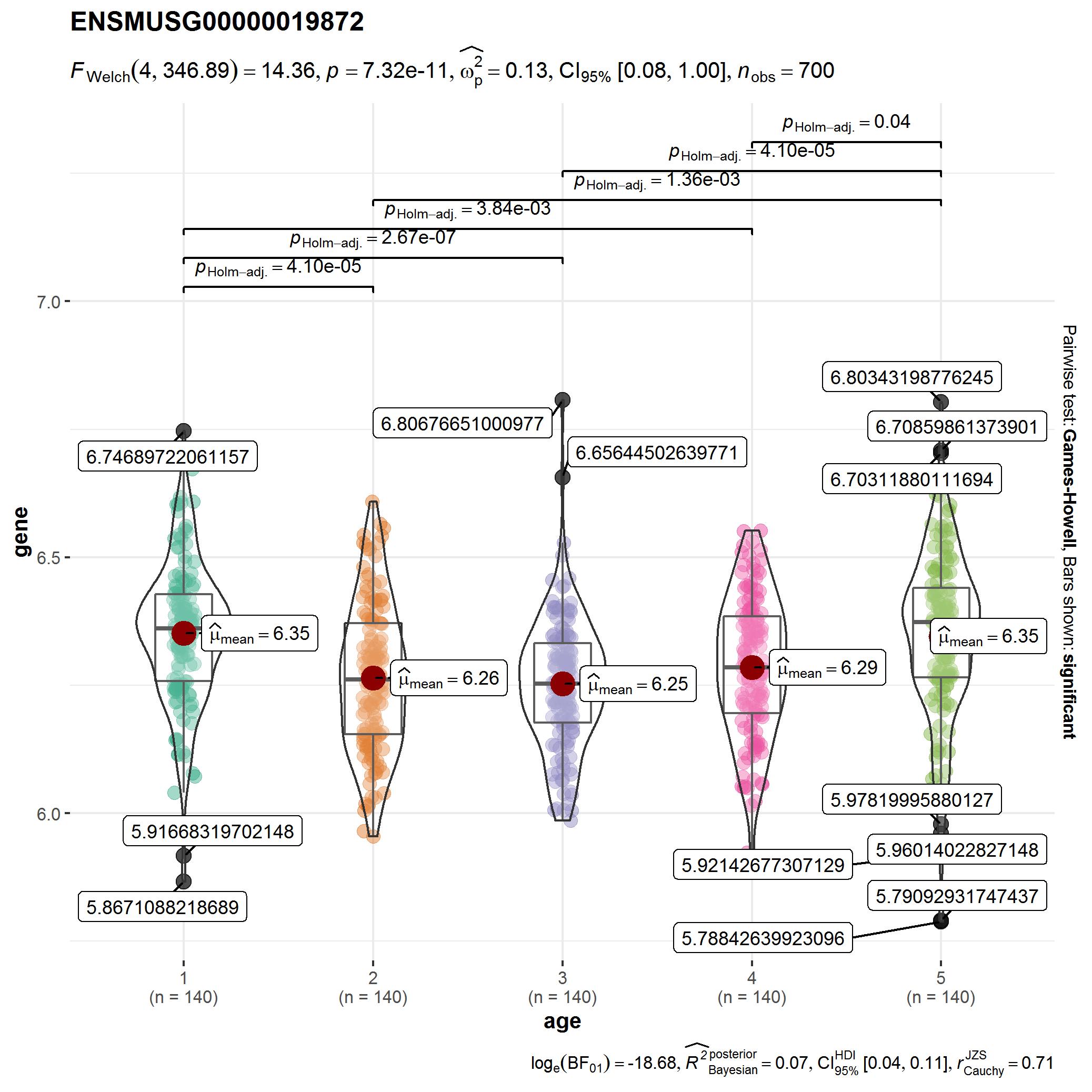

Supplement: Supplementary file 25 — Data S1–S6. [file ACEL-23-e14268-s017.zip › Data S1/ENSMUSG00000019872.jpeg]

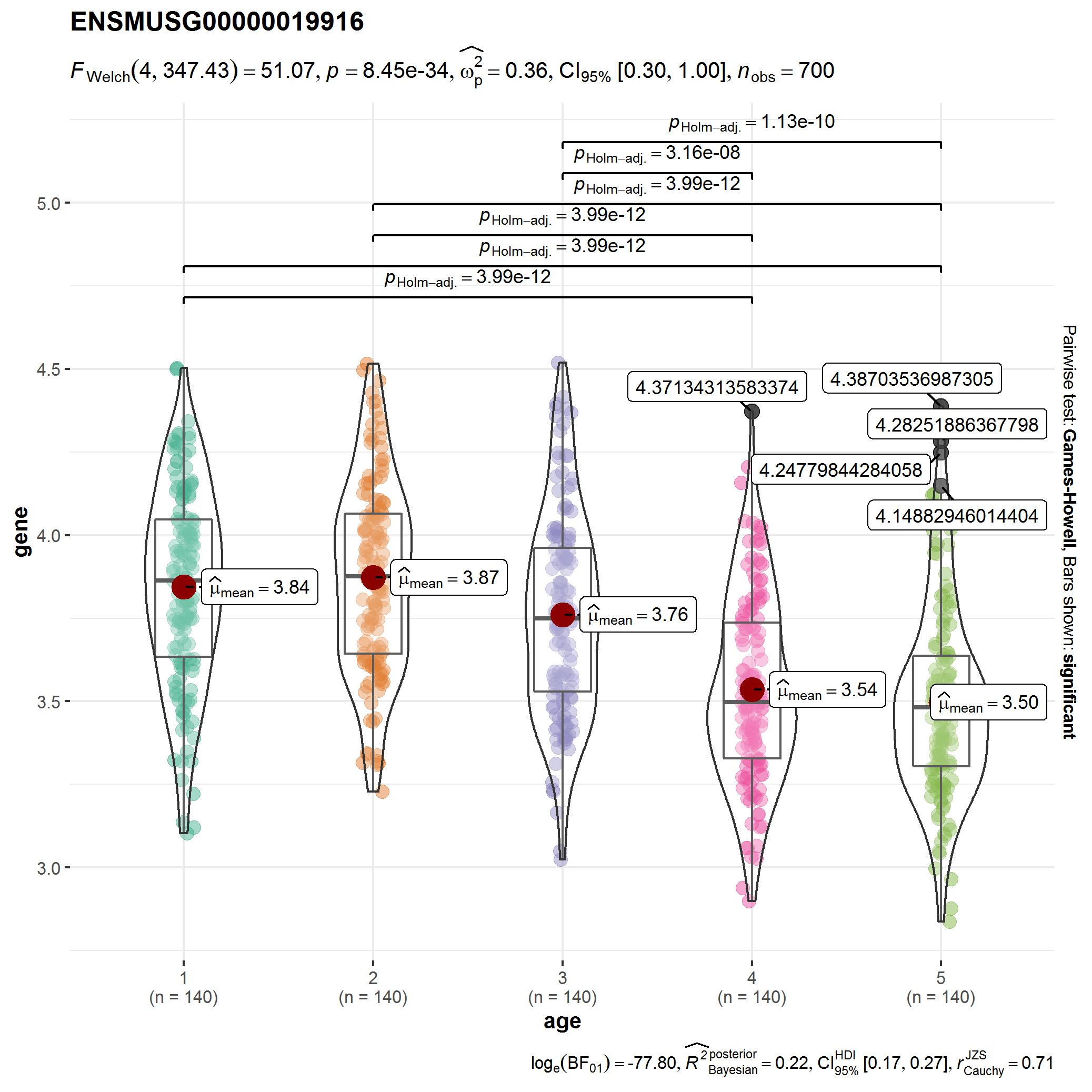

Supplement: Supplementary file 25 — Data S1–S6. [file ACEL-23-e14268-s017.zip › Data S1/ENSMUSG00000019916.jpeg]

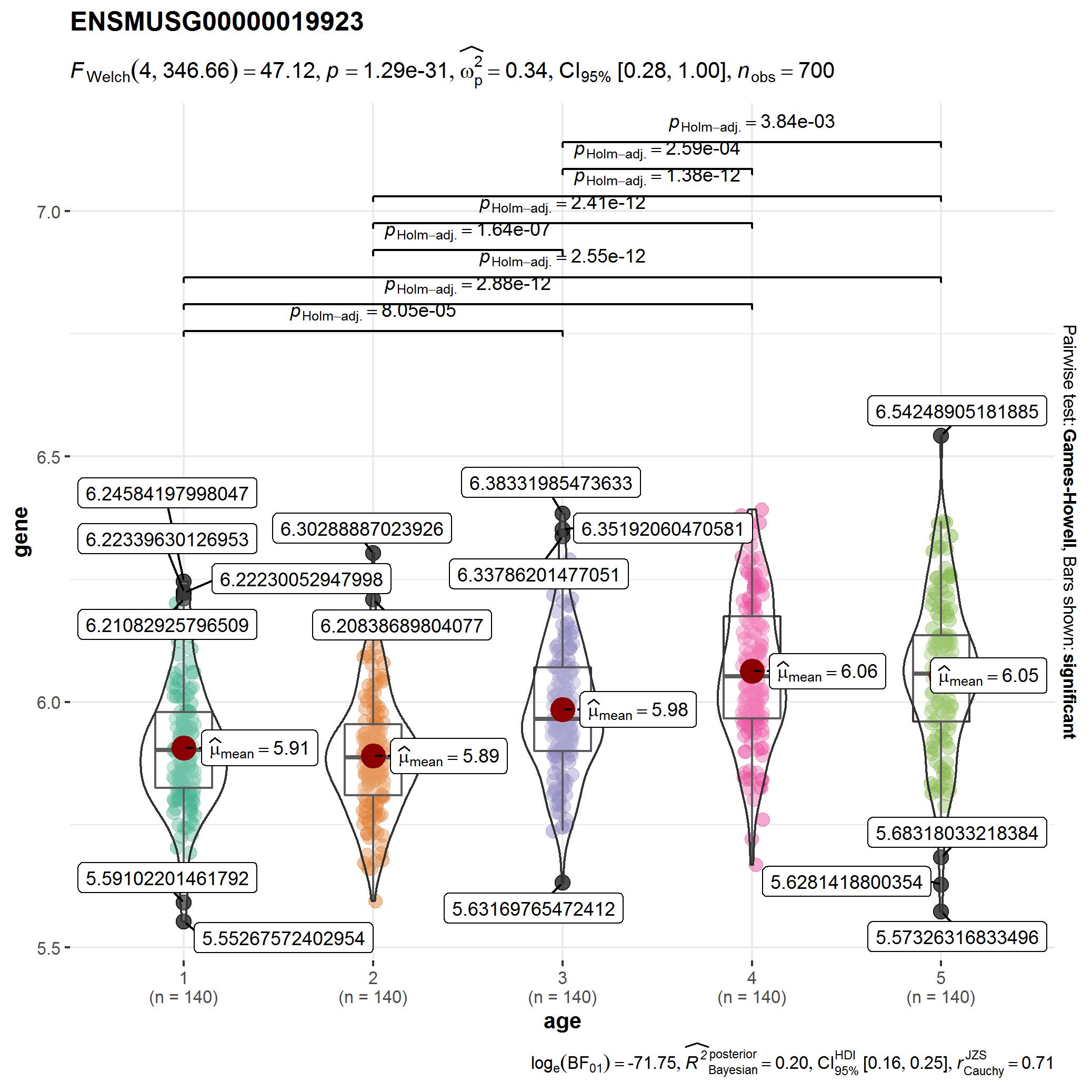

Supplement: Supplementary file 25 — Data S1–S6. [file ACEL-23-e14268-s017.zip › Data S1/ENSMUSG00000019923.jpeg]

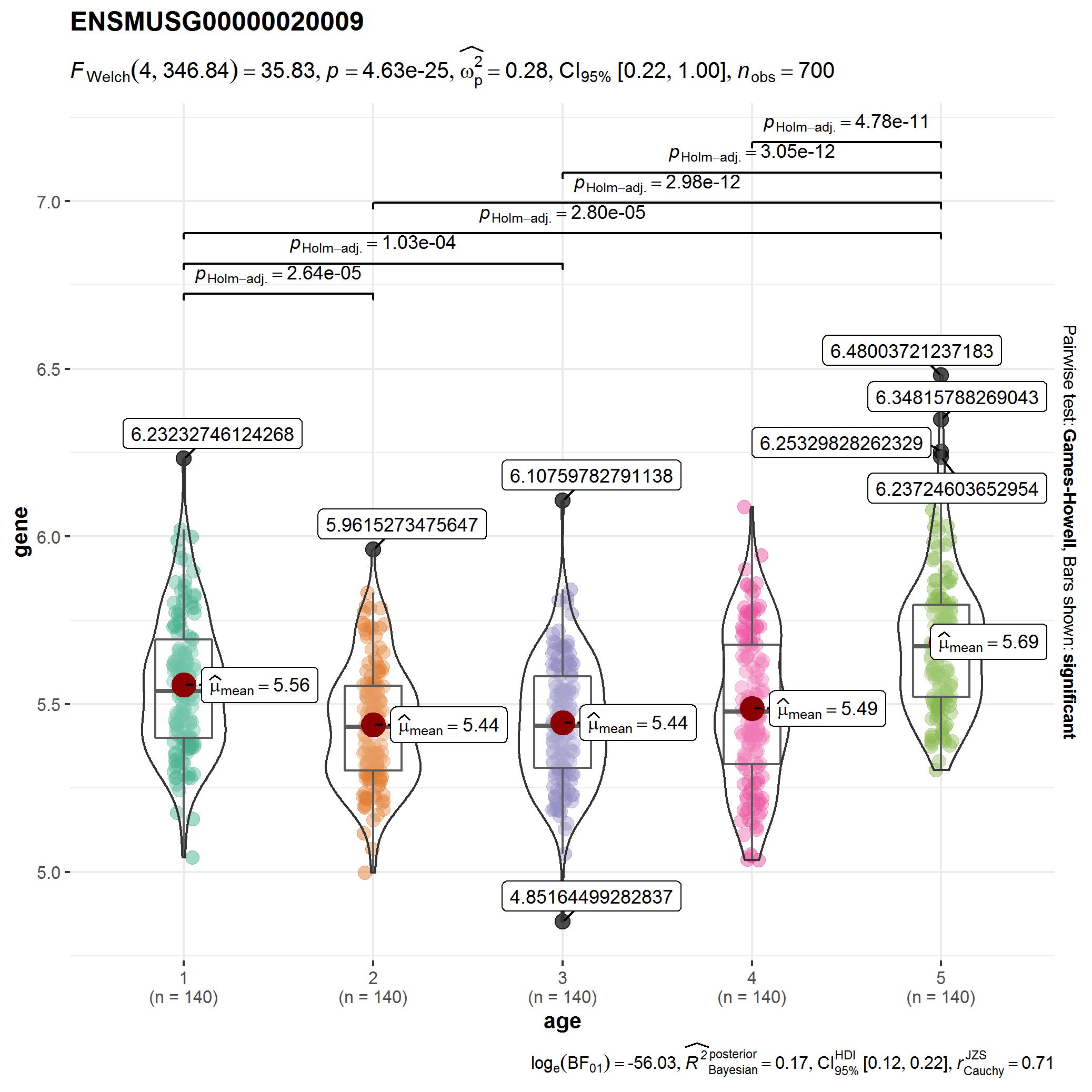

Supplement: Supplementary file 25 — Data S1–S6. [file ACEL-23-e14268-s017.zip › Data S1/ENSMUSG00000020009.jpeg]

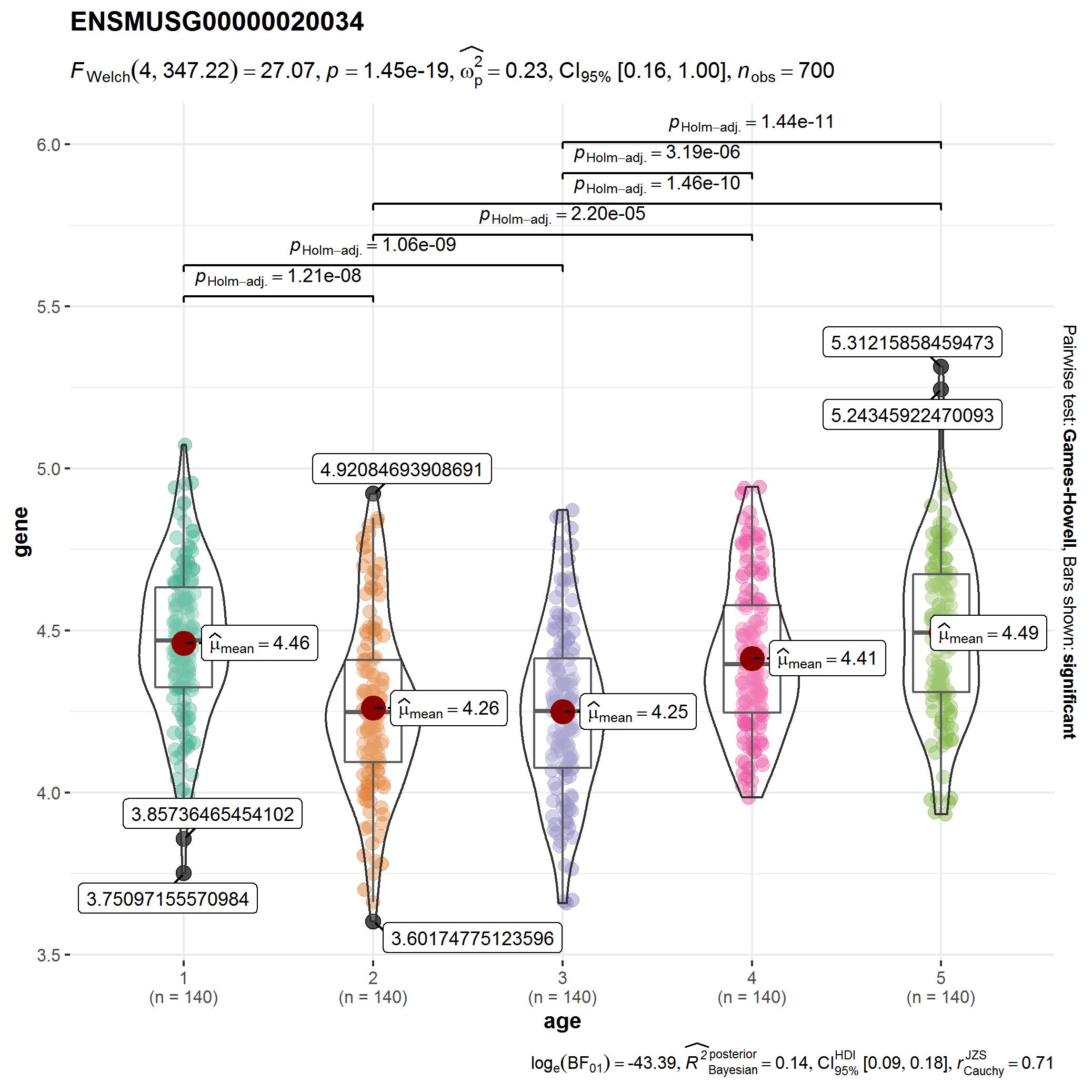

Supplement: Supplementary file 25 — Data S1–S6. [file ACEL-23-e14268-s017.zip › Data S1/ENSMUSG00000020034.jpeg]

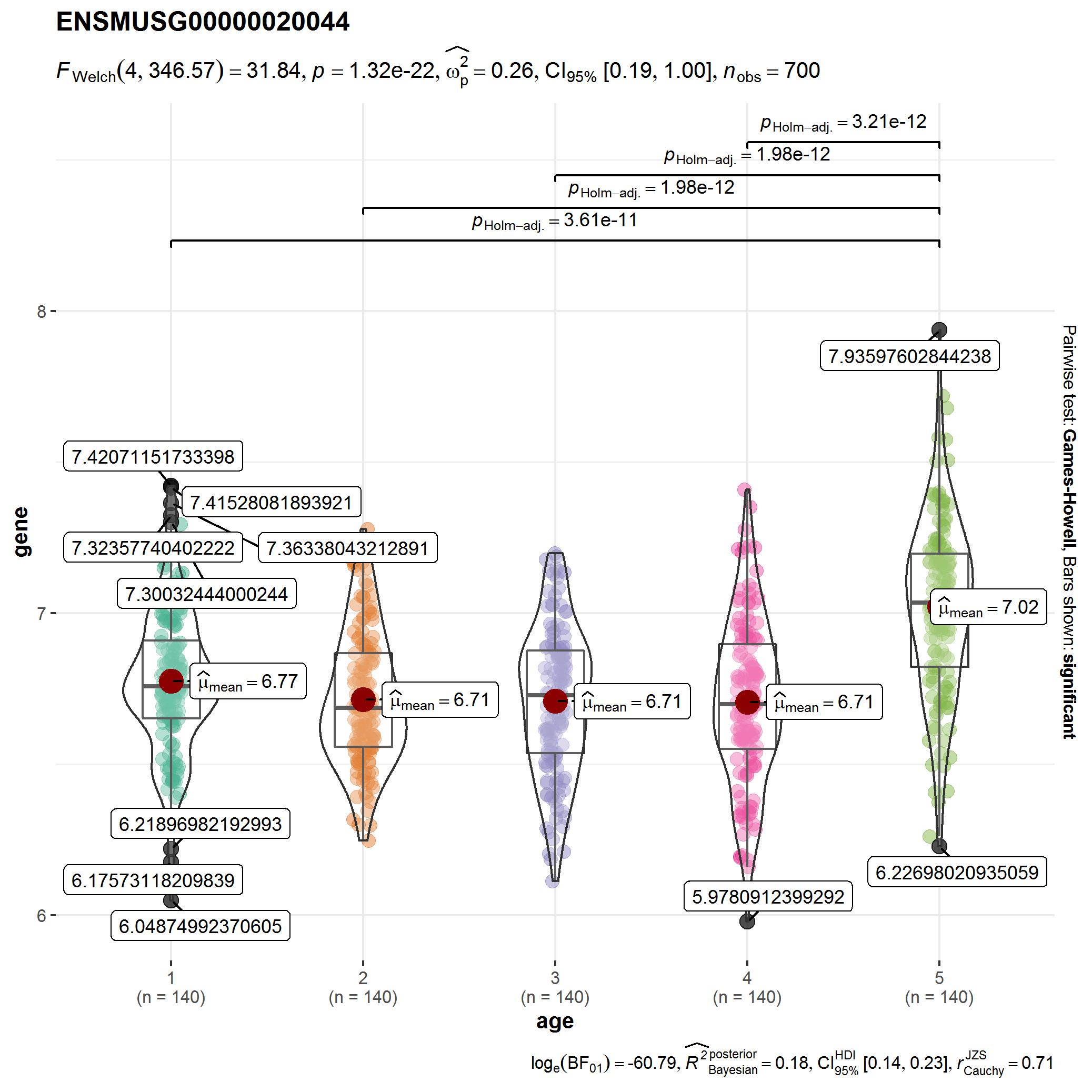

Supplement: Supplementary file 25 — Data S1–S6. [file ACEL-23-e14268-s017.zip › Data S1/ENSMUSG00000020044.jpeg]

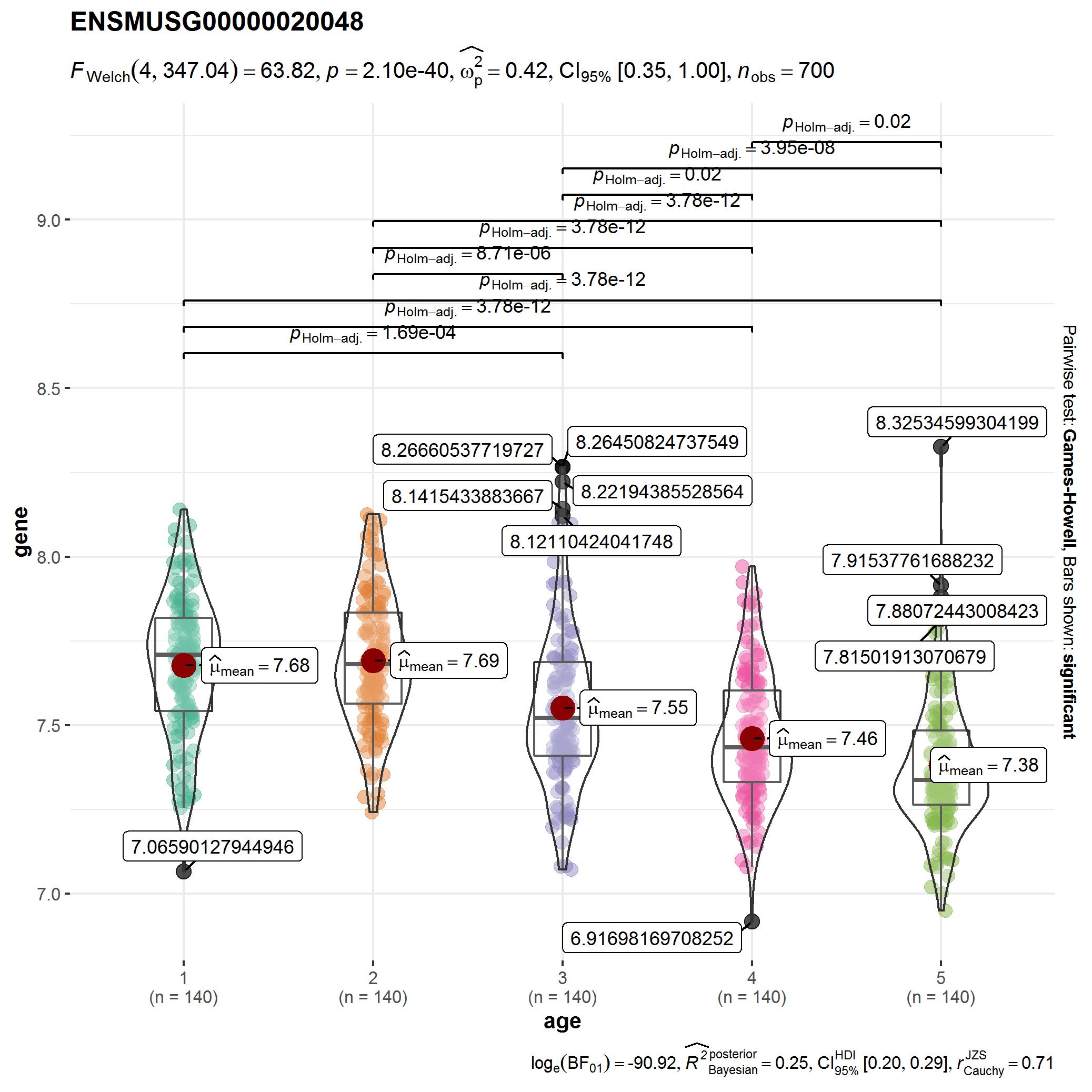

Supplement: Supplementary file 25 — Data S1–S6. [file ACEL-23-e14268-s017.zip › Data S1/ENSMUSG00000020048.jpeg]

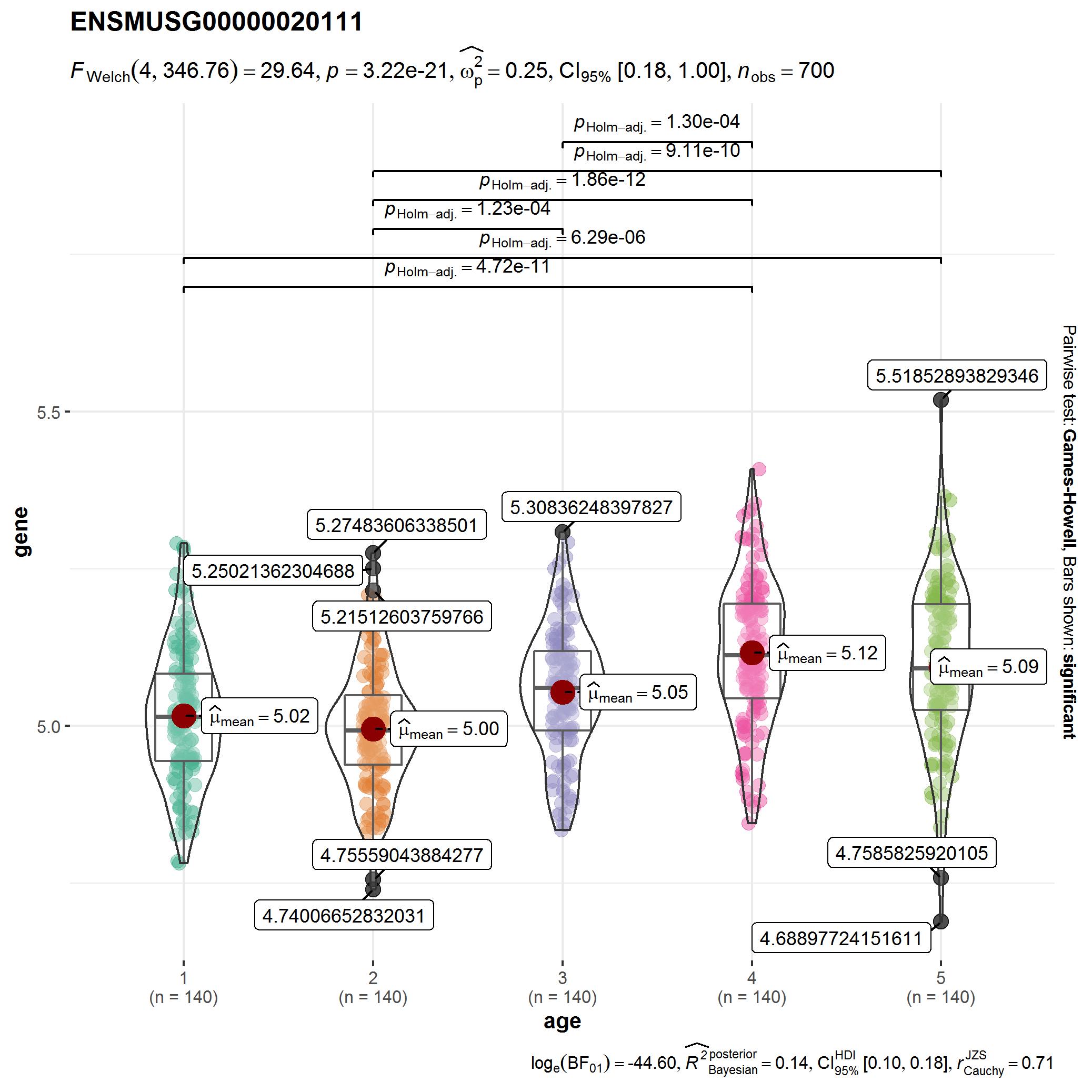

Supplement: Supplementary file 25 — Data S1–S6. [file ACEL-23-e14268-s017.zip › Data S1/ENSMUSG00000020111.jpeg]

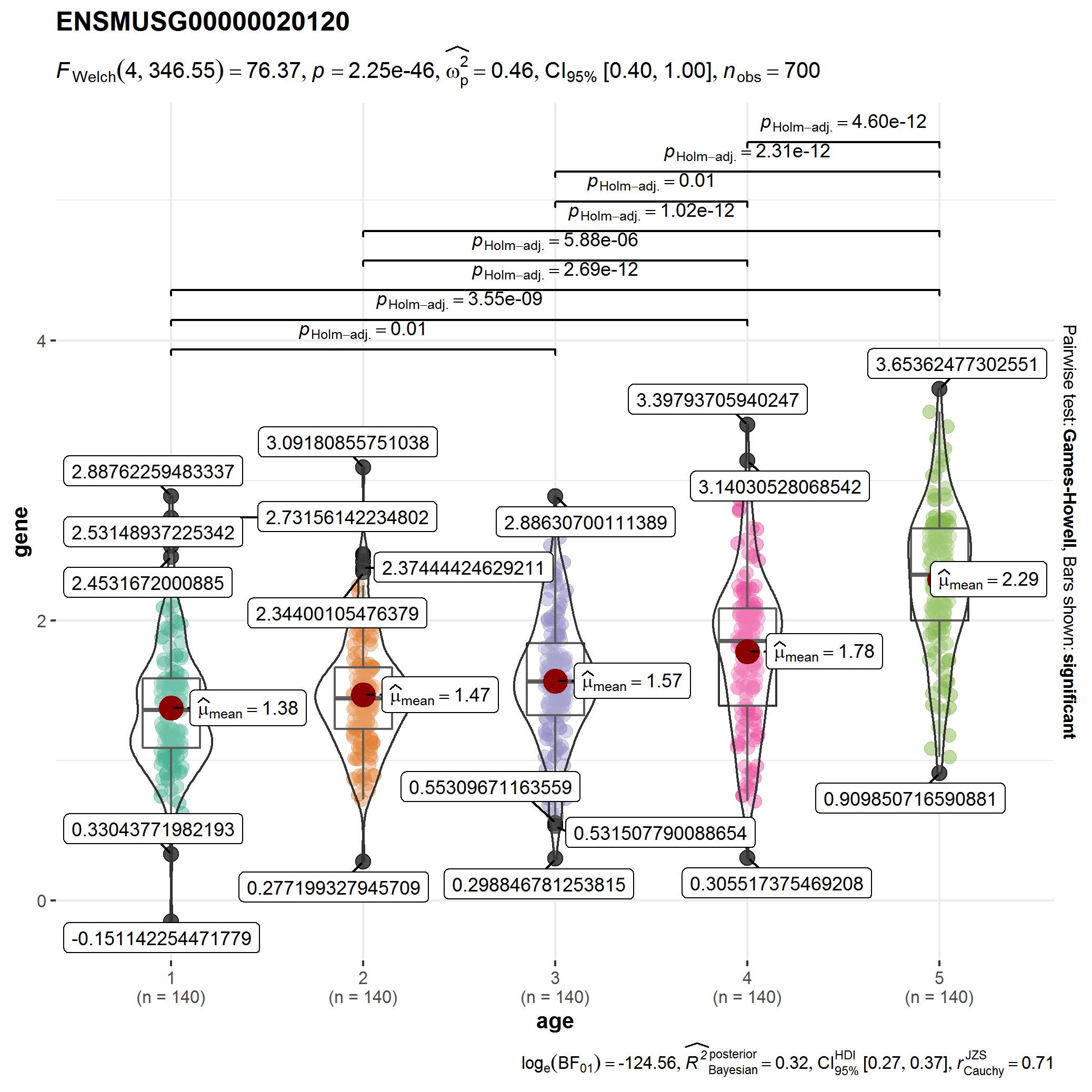

Supplement: Supplementary file 25 — Data S1–S6. [file ACEL-23-e14268-s017.zip › Data S1/ENSMUSG00000020120.jpeg]

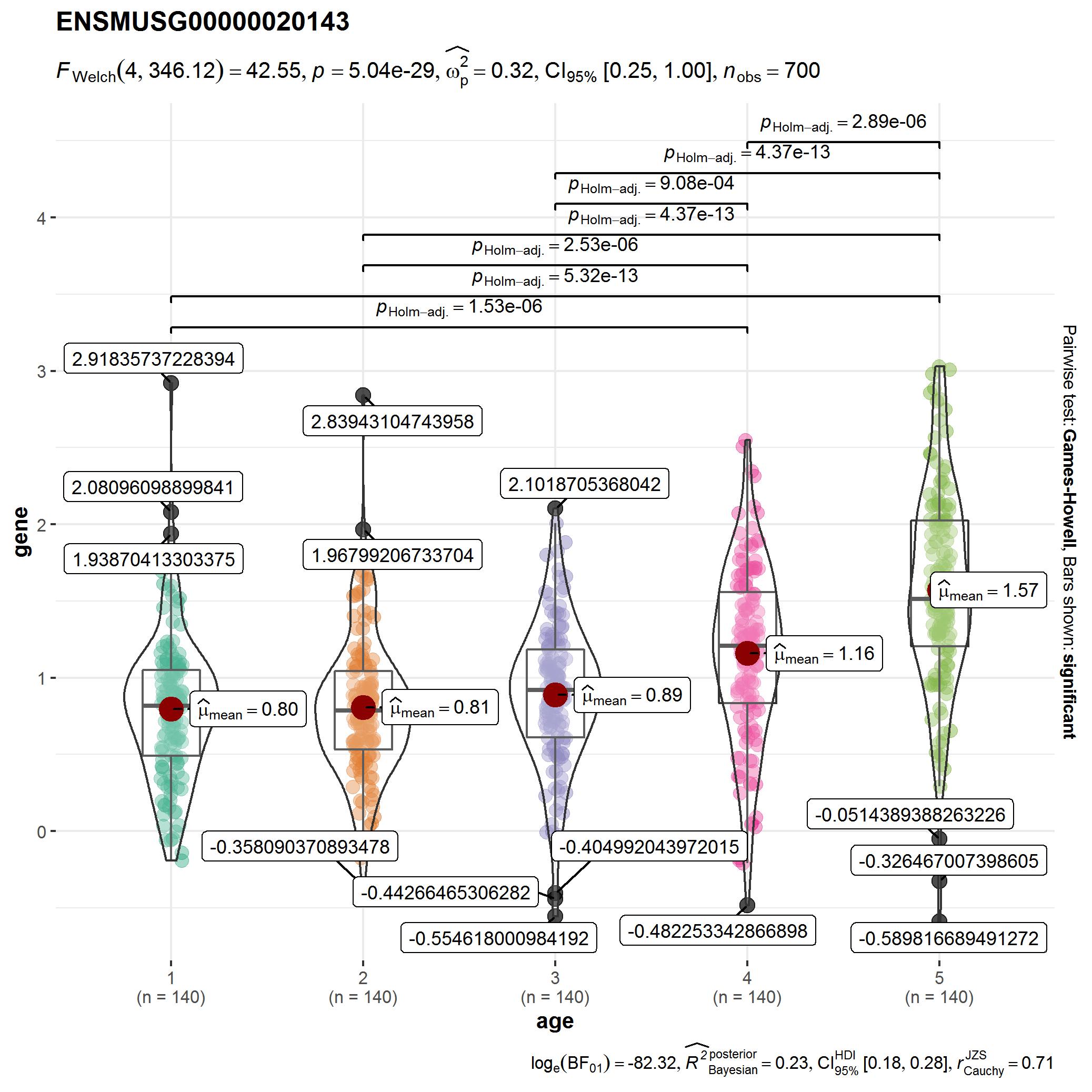

Supplement: Supplementary file 25 — Data S1–S6. [file ACEL-23-e14268-s017.zip › Data S1/ENSMUSG00000020143.jpeg]

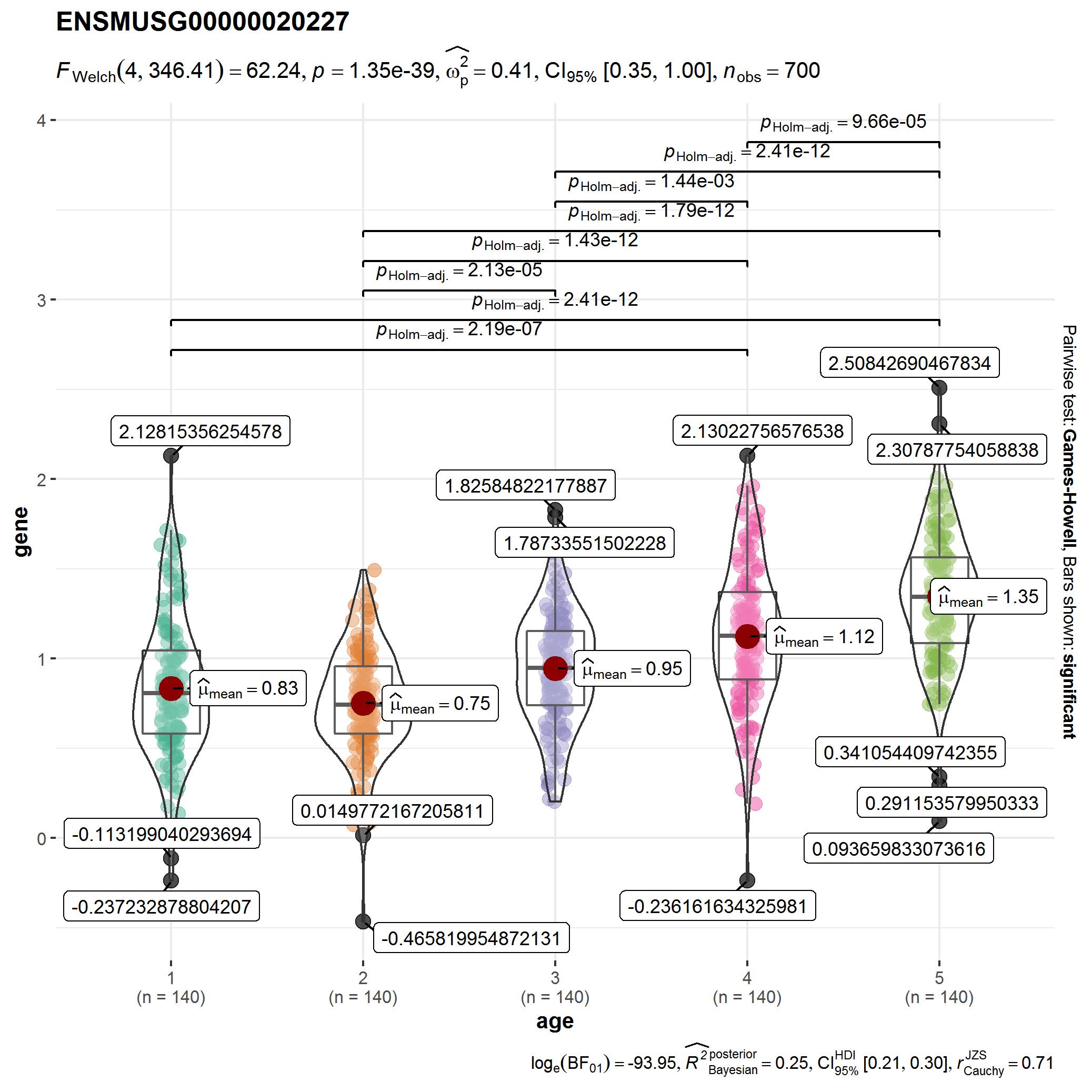

Supplement: Supplementary file 25 — Data S1–S6. [file ACEL-23-e14268-s017.zip › Data S1/ENSMUSG00000020227.jpeg]

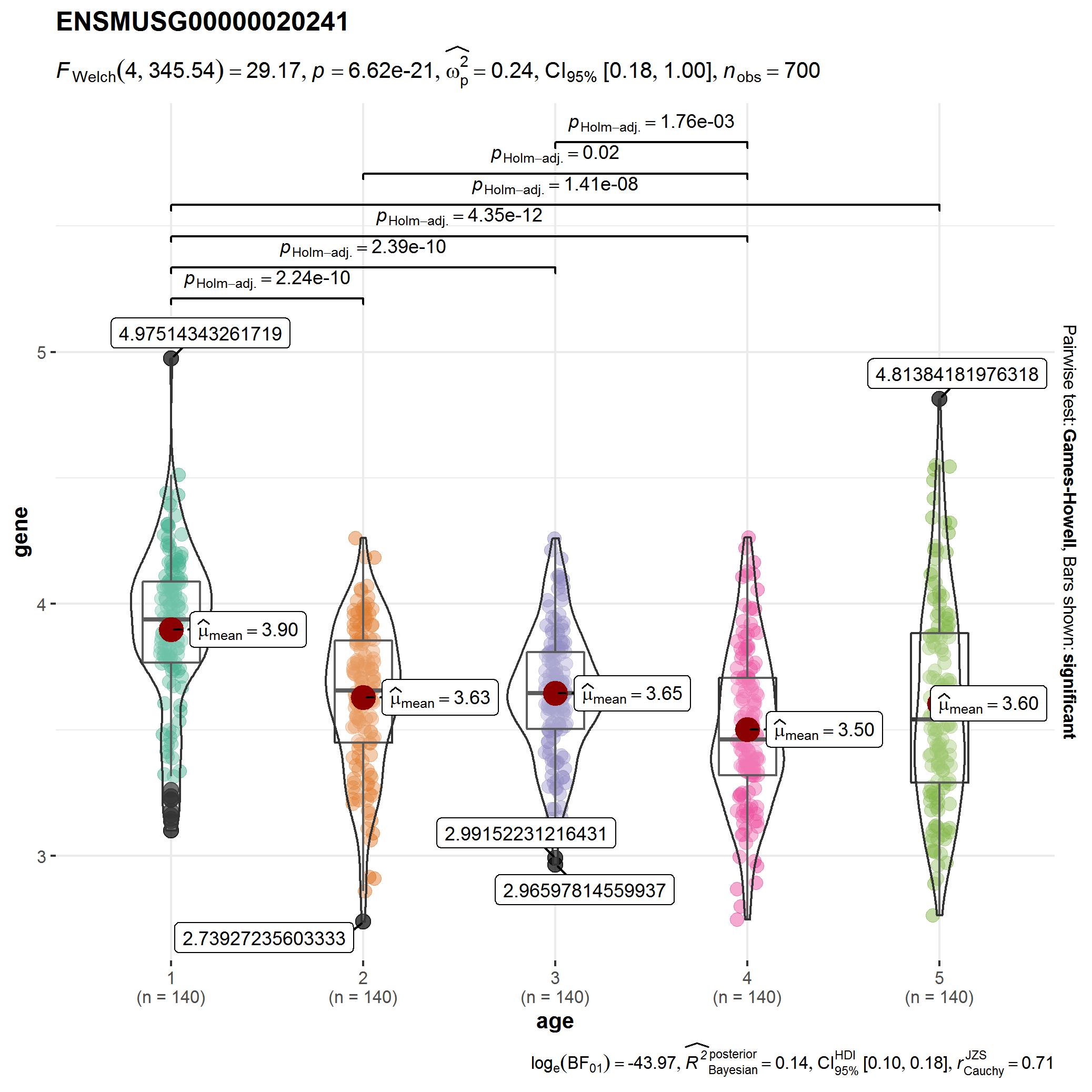

Supplement: Supplementary file 25 — Data S1–S6. [file ACEL-23-e14268-s017.zip › Data S1/ENSMUSG00000020241.jpeg]

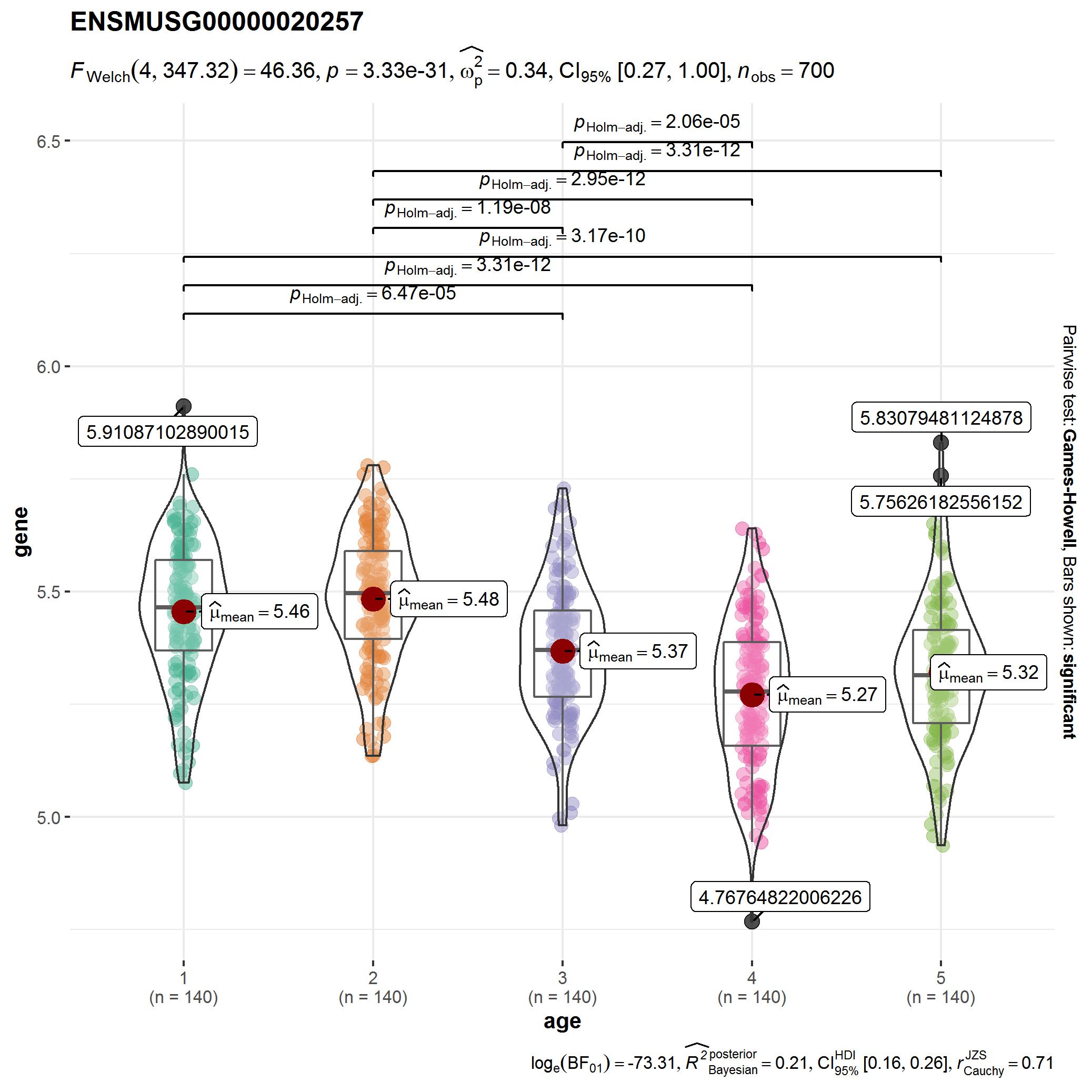

Supplement: Supplementary file 25 — Data S1–S6. [file ACEL-23-e14268-s017.zip › Data S1/ENSMUSG00000020257.jpeg]

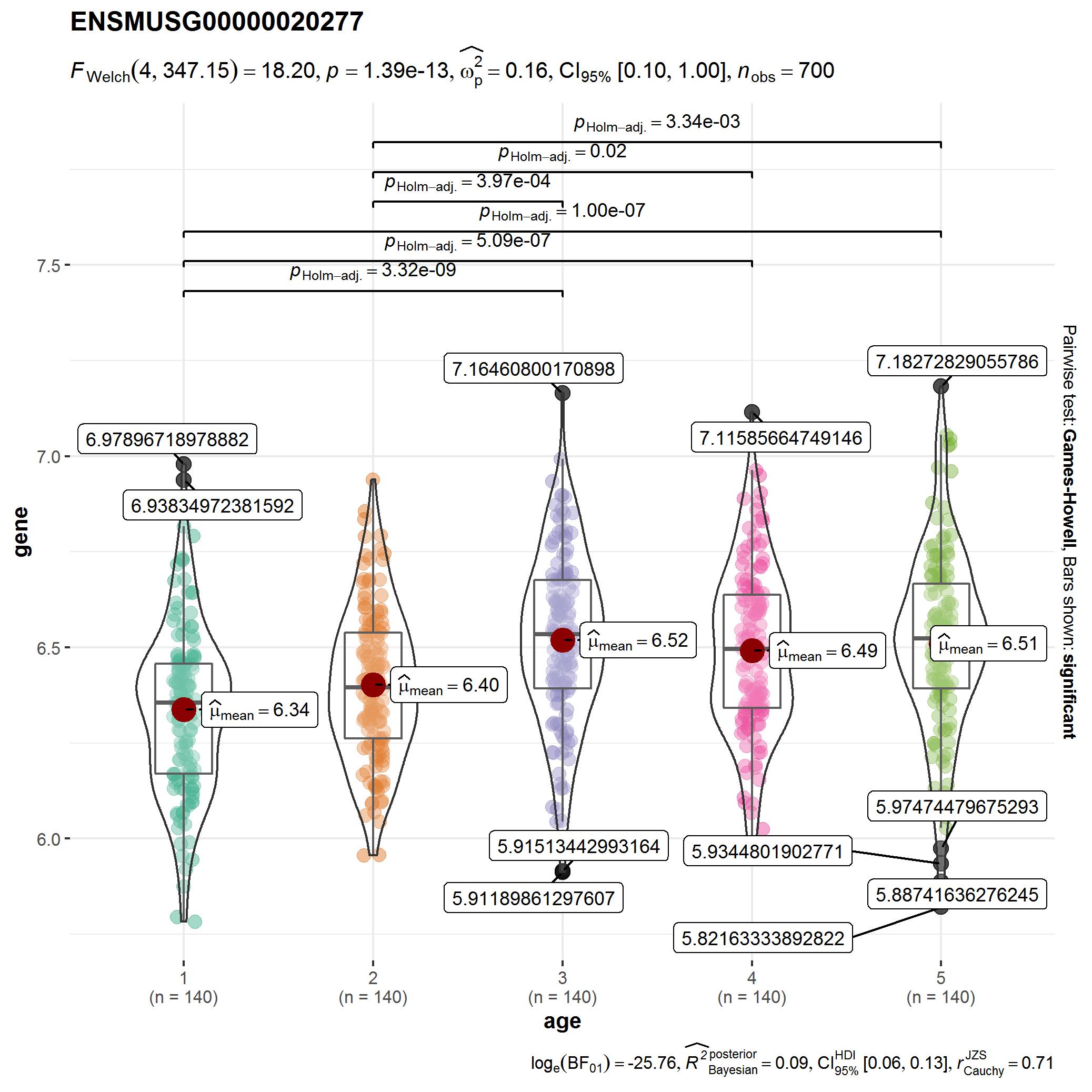

Supplement: Supplementary file 25 — Data S1–S6. [file ACEL-23-e14268-s017.zip › Data S1/ENSMUSG00000020277.jpeg]

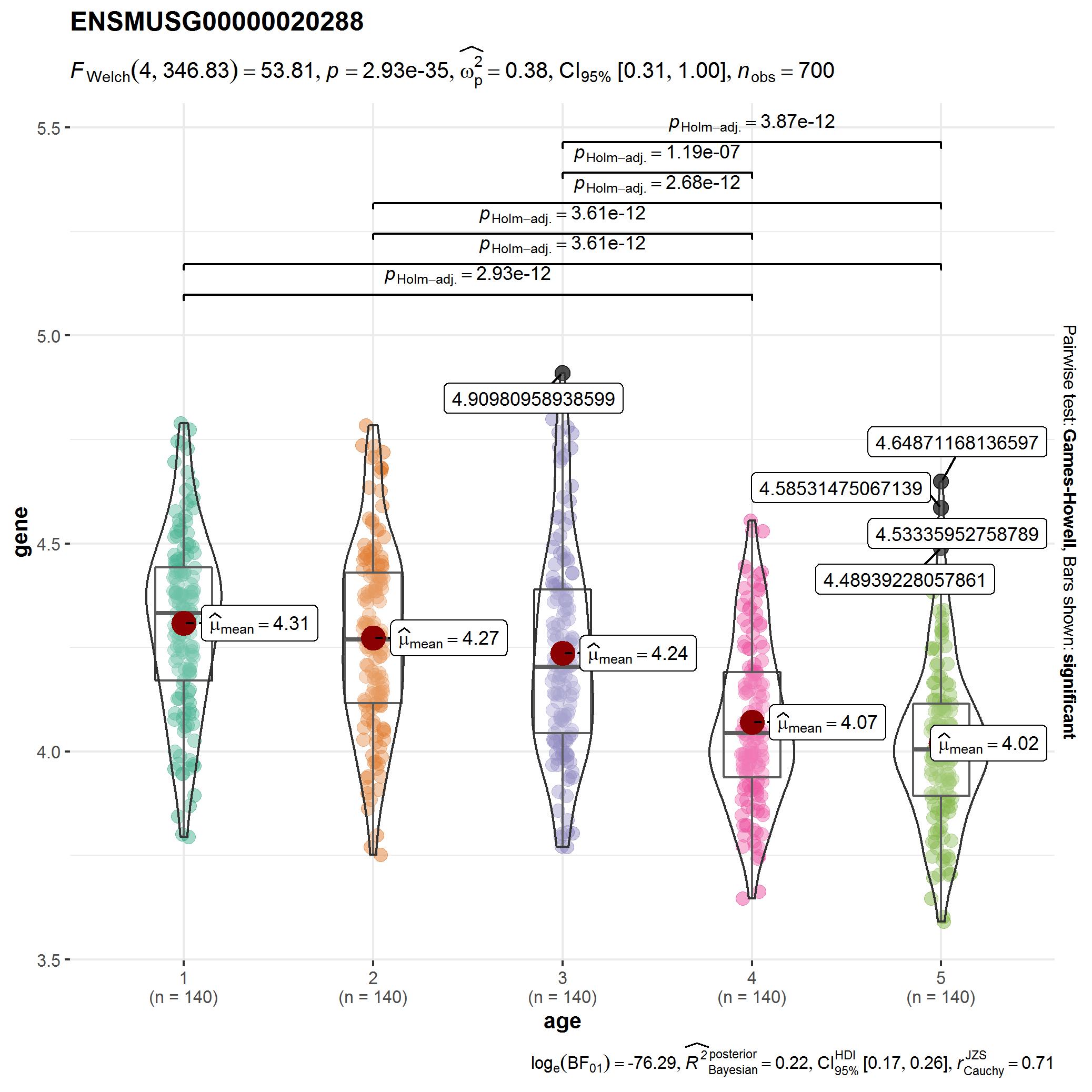

Supplement: Supplementary file 25 — Data S1–S6. [file ACEL-23-e14268-s017.zip › Data S1/ENSMUSG00000020288.jpeg]

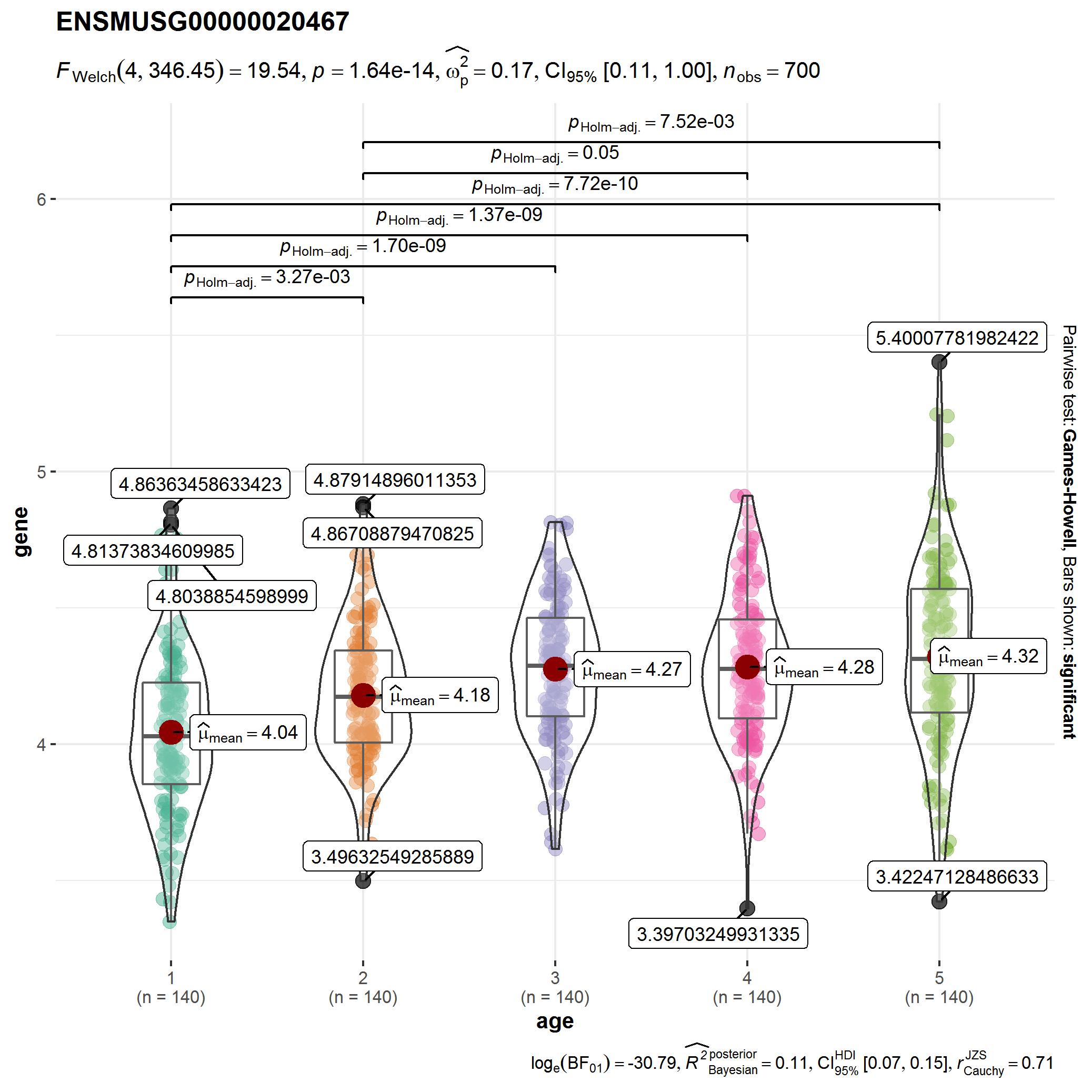

Supplement: Supplementary file 25 — Data S1–S6. [file ACEL-23-e14268-s017.zip › Data S1/ENSMUSG00000020467.jpeg]

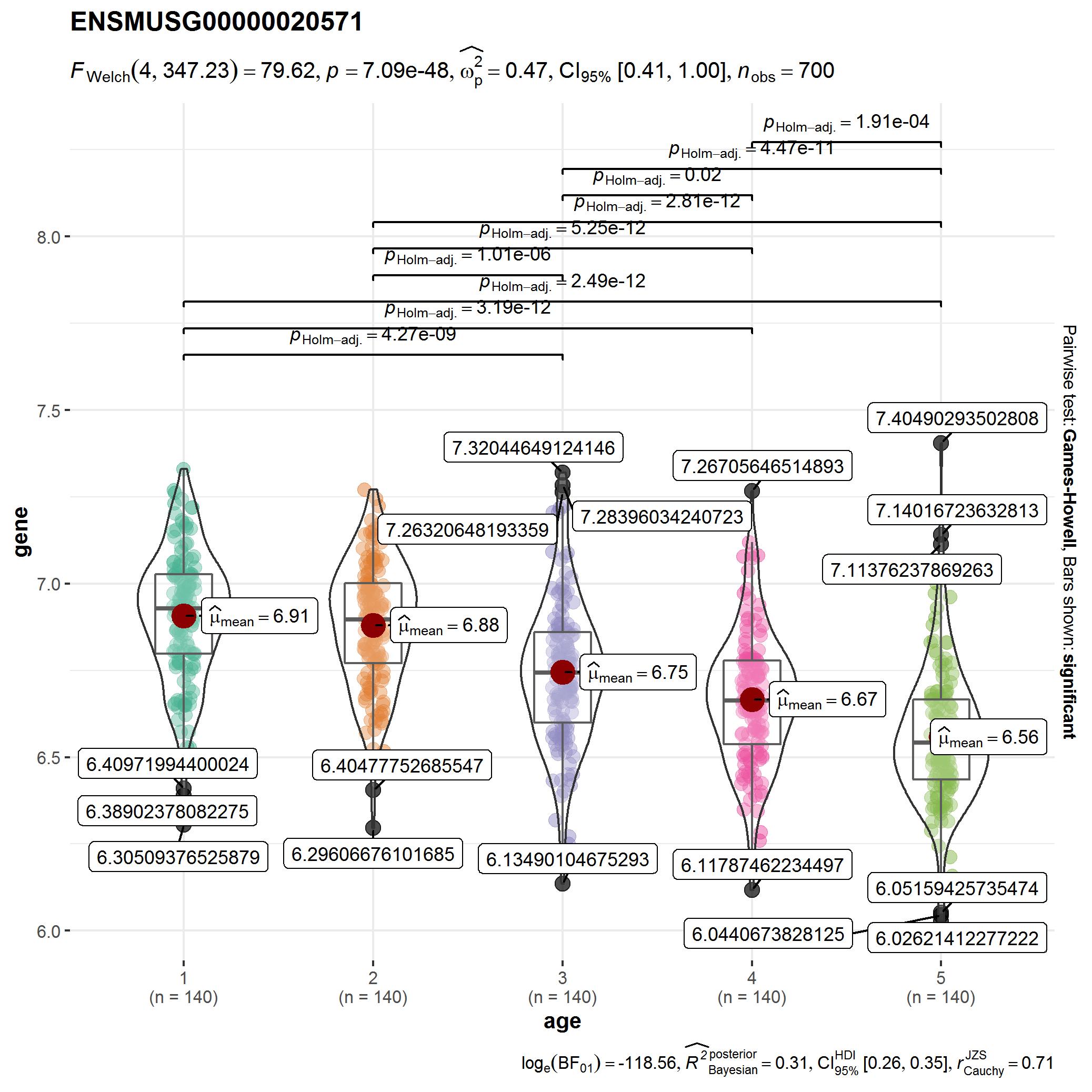

Supplement: Supplementary file 25 — Data S1–S6. [file ACEL-23-e14268-s017.zip › Data S1/ENSMUSG00000020571.jpeg]

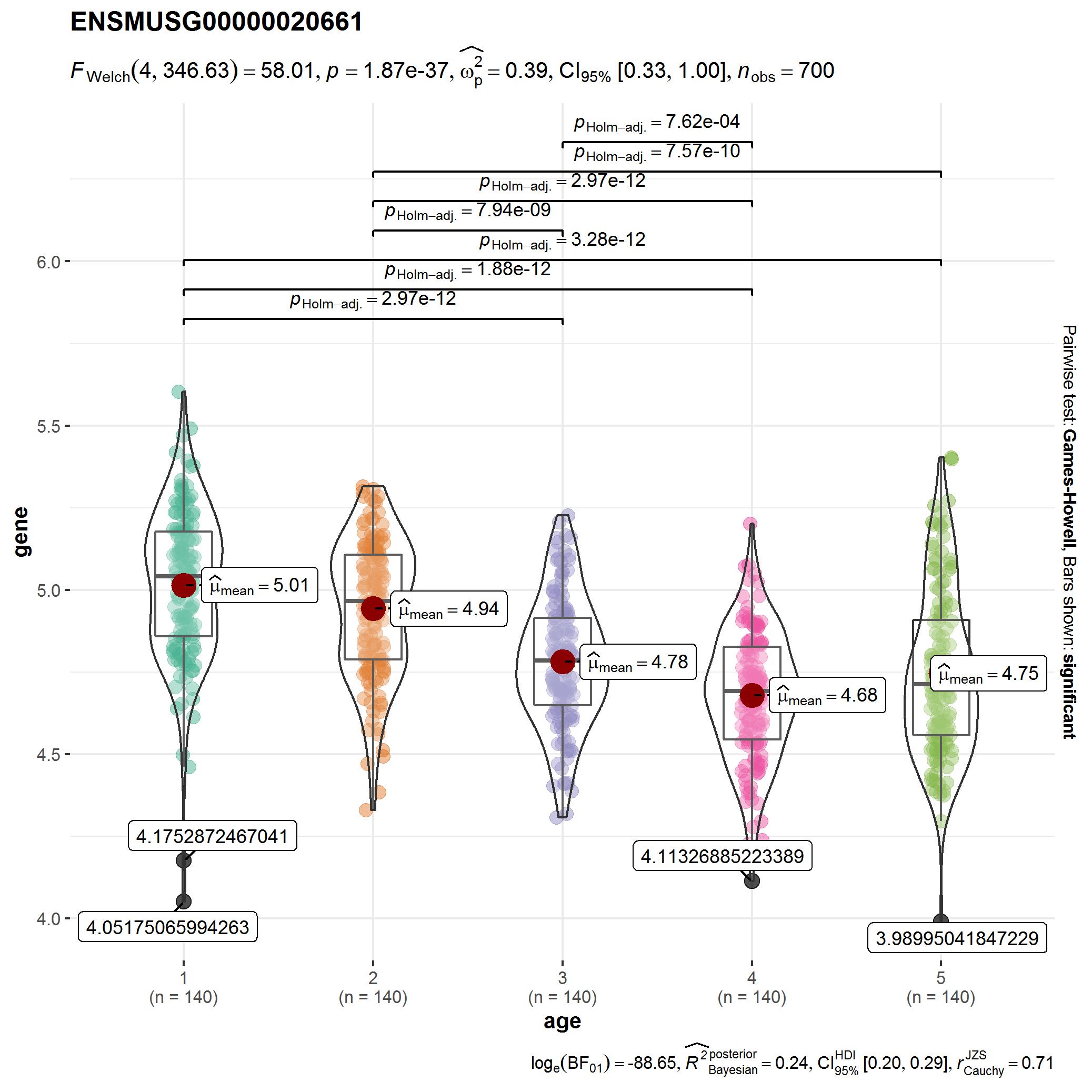

Supplement: Supplementary file 25 — Data S1–S6. [file ACEL-23-e14268-s017.zip › Data S1/ENSMUSG00000020661.jpeg]

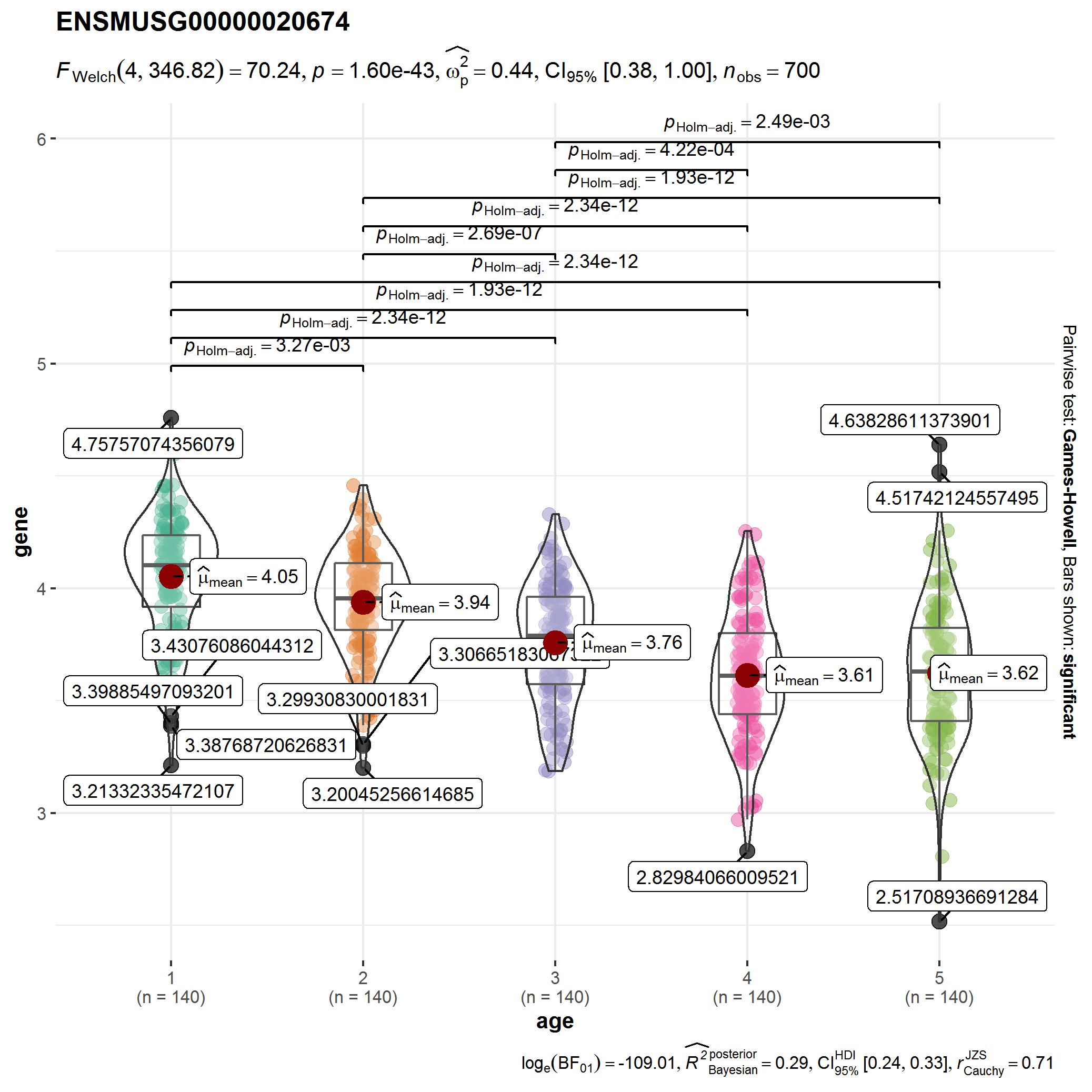

Supplement: Supplementary file 25 — Data S1–S6. [file ACEL-23-e14268-s017.zip › Data S1/ENSMUSG00000020674.jpeg]

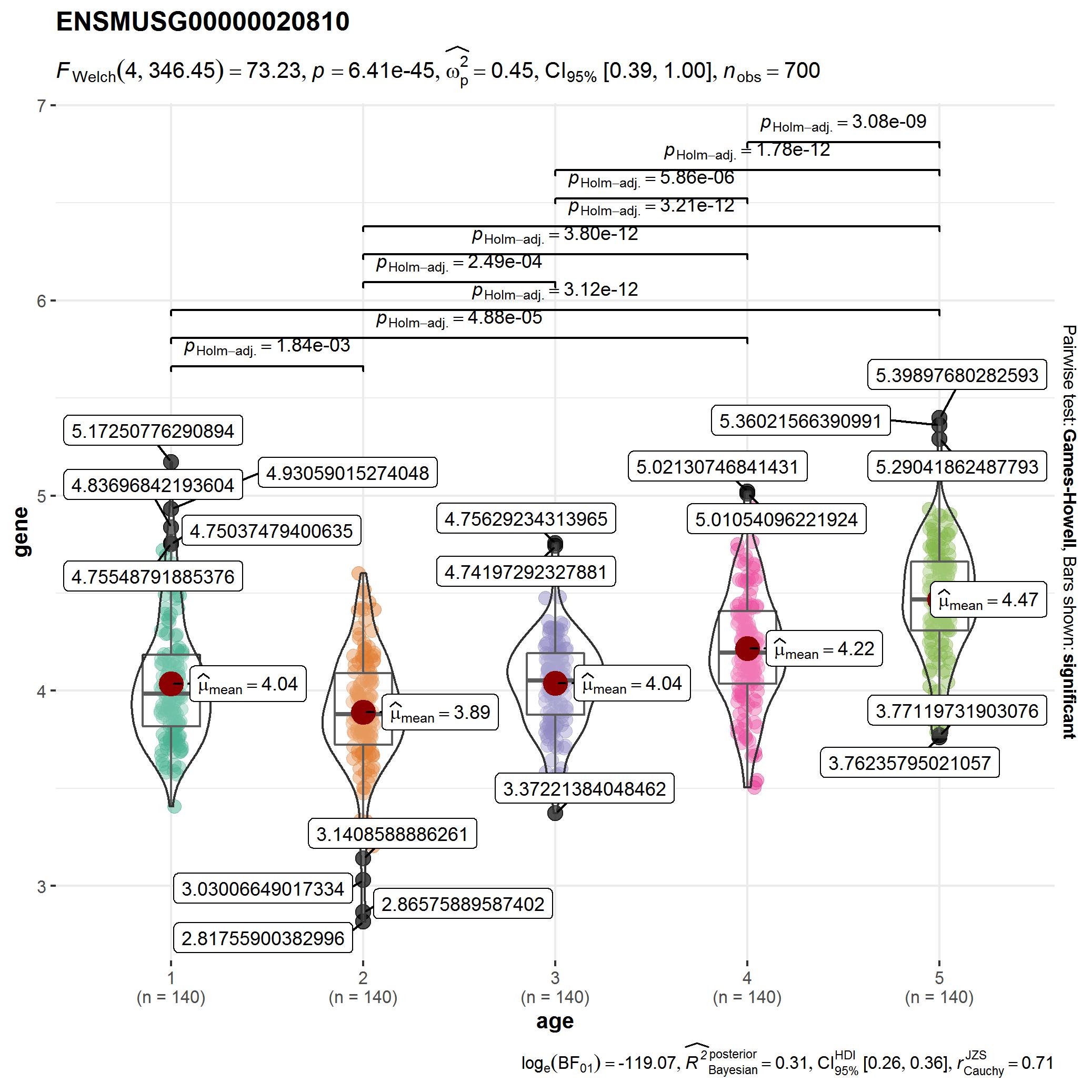

Supplement: Supplementary file 25 — Data S1–S6. [file ACEL-23-e14268-s017.zip › Data S1/ENSMUSG00000020810.jpeg]

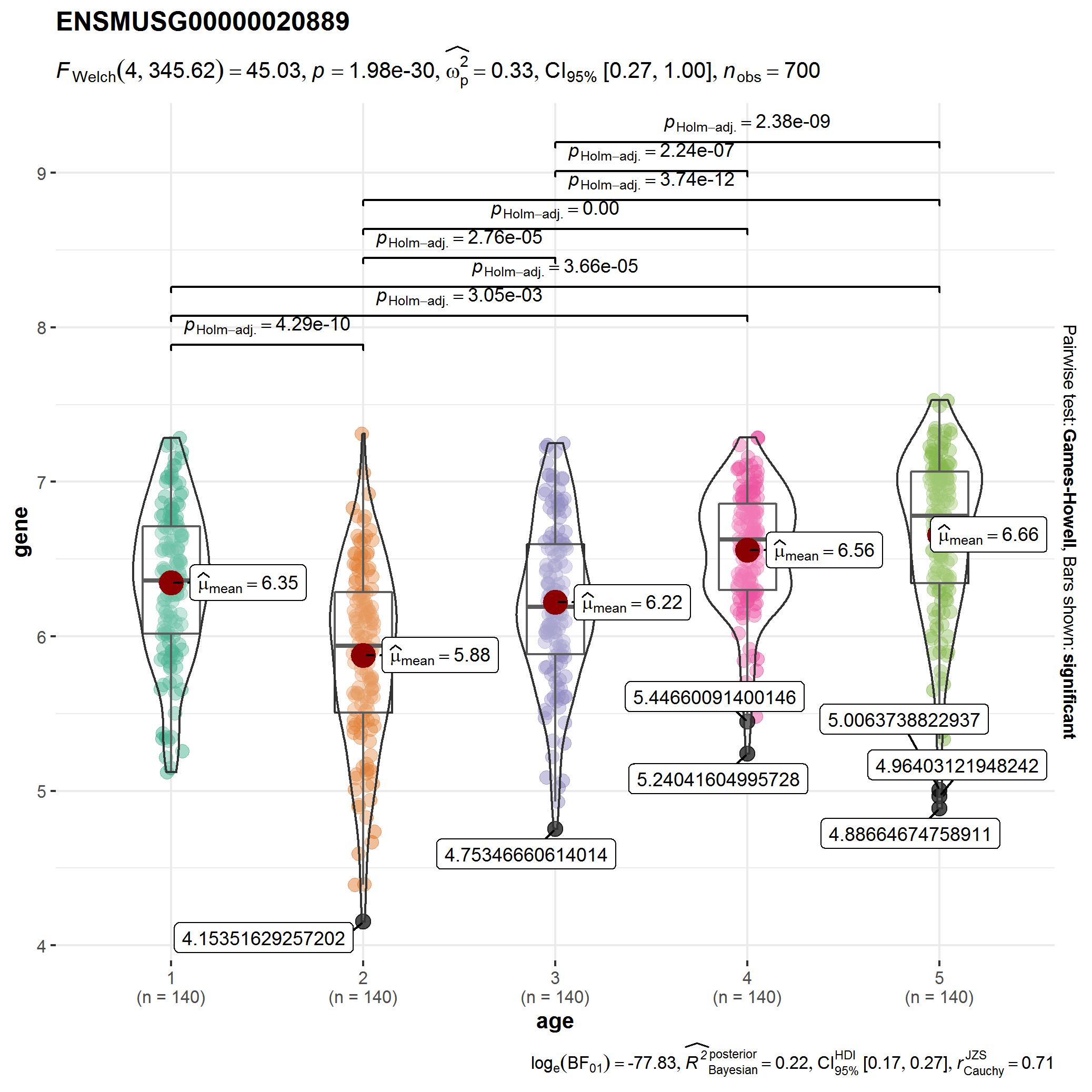

Supplement: Supplementary file 25 — Data S1–S6. [file ACEL-23-e14268-s017.zip › Data S1/ENSMUSG00000020889.jpeg]

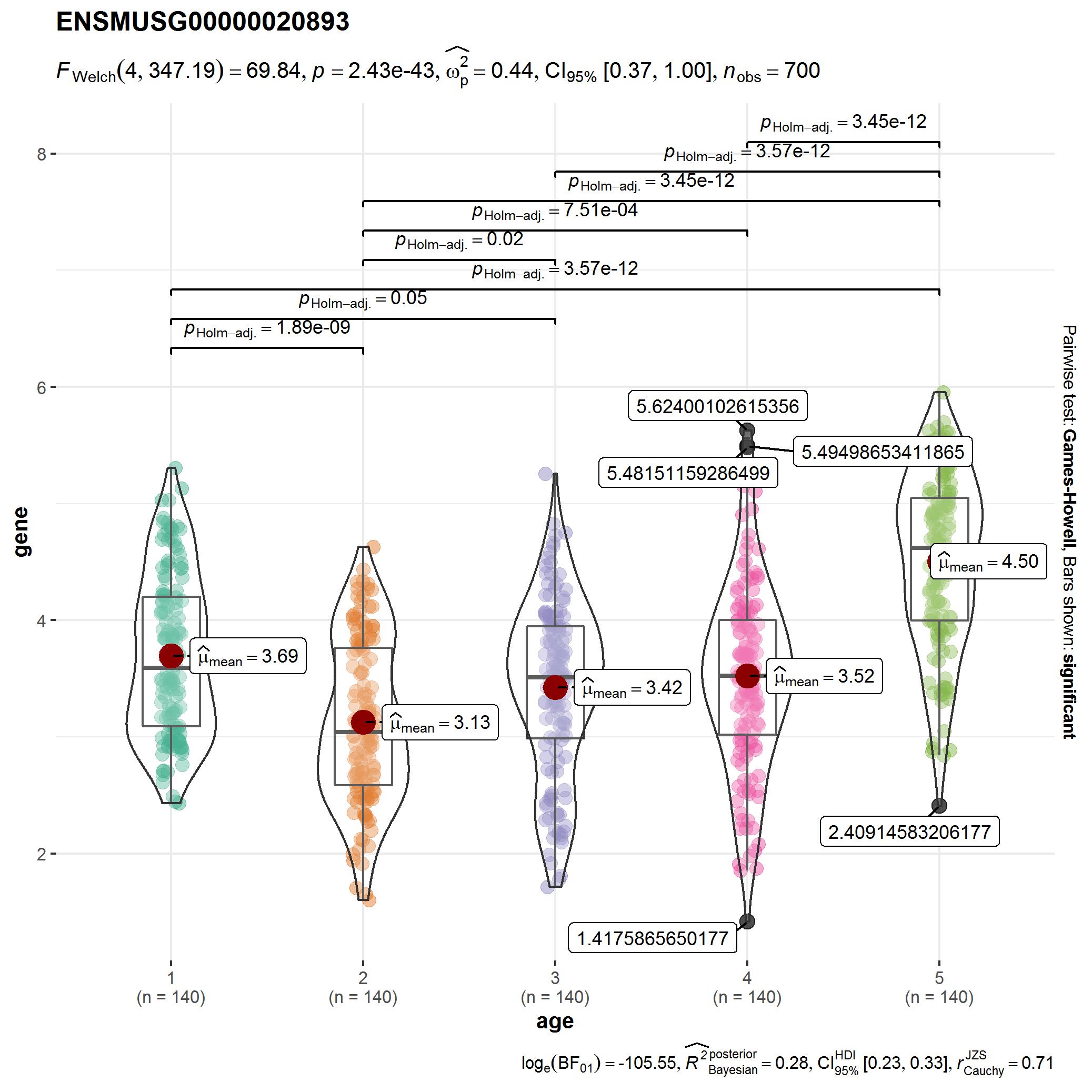

Supplement: Supplementary file 25 — Data S1–S6. [file ACEL-23-e14268-s017.zip › Data S1/ENSMUSG00000020893.jpeg]

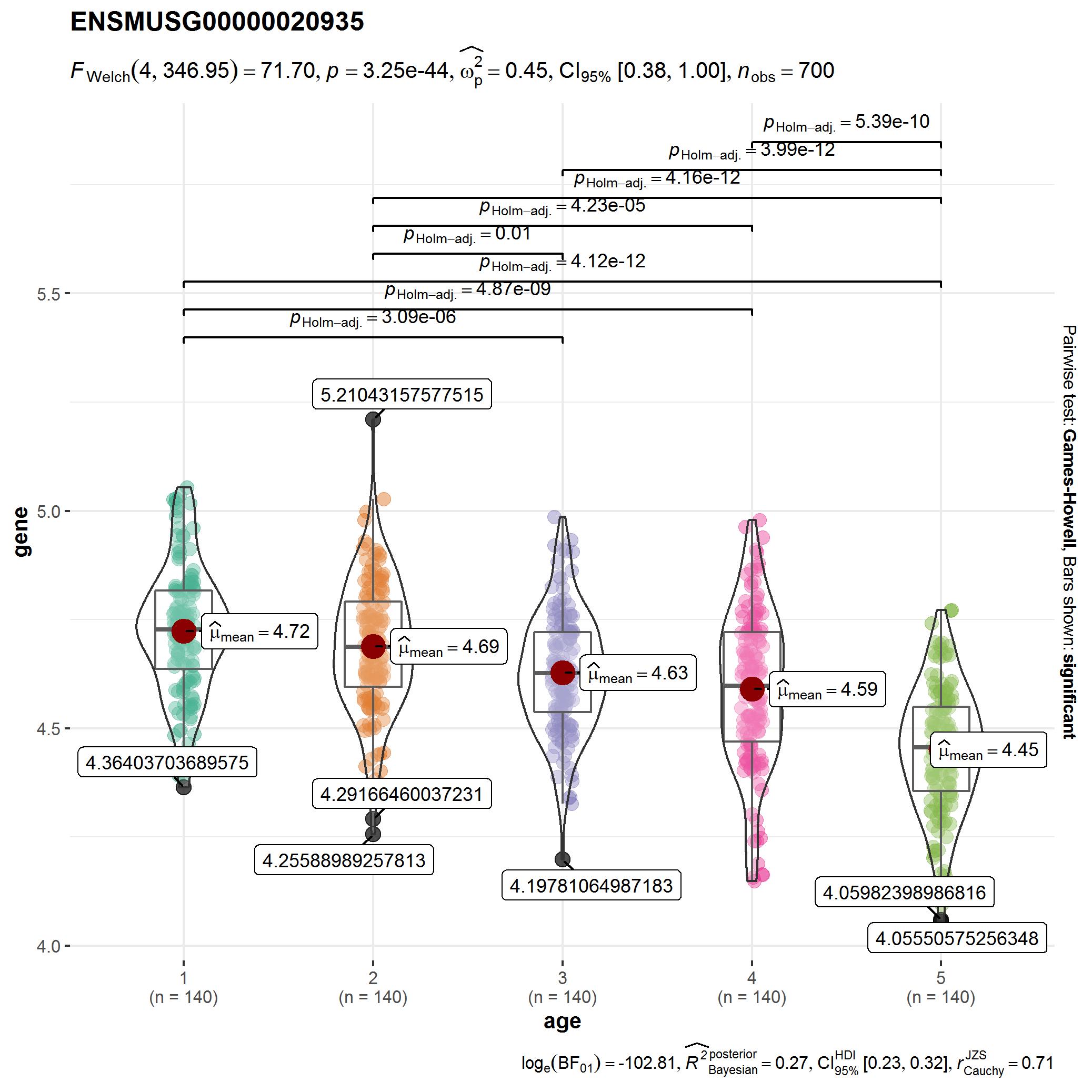

Supplement: Supplementary file 25 — Data S1–S6. [file ACEL-23-e14268-s017.zip › Data S1/ENSMUSG00000020935.jpeg]

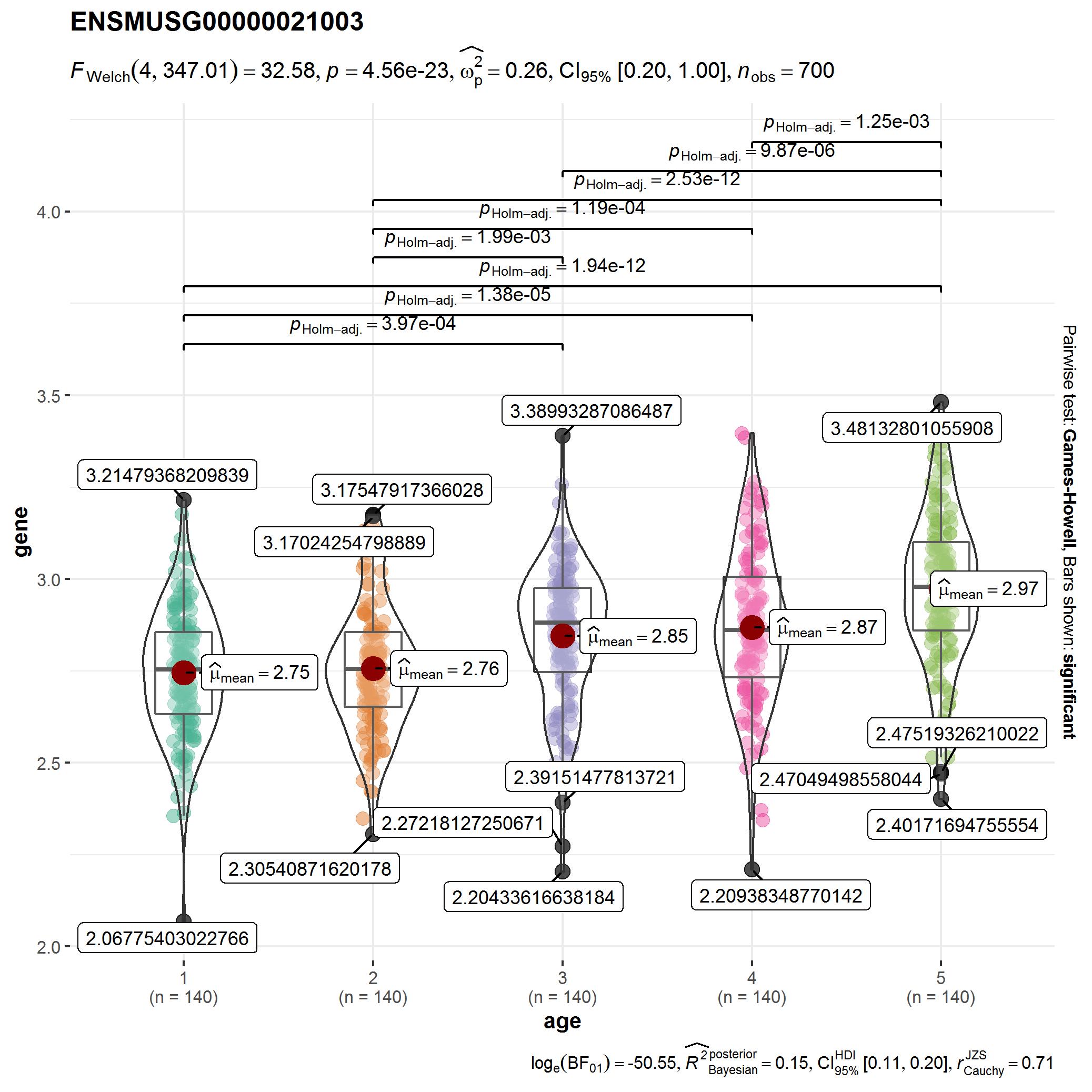

Supplement: Supplementary file 25 — Data S1–S6. [file ACEL-23-e14268-s017.zip › Data S1/ENSMUSG00000021003.jpeg]

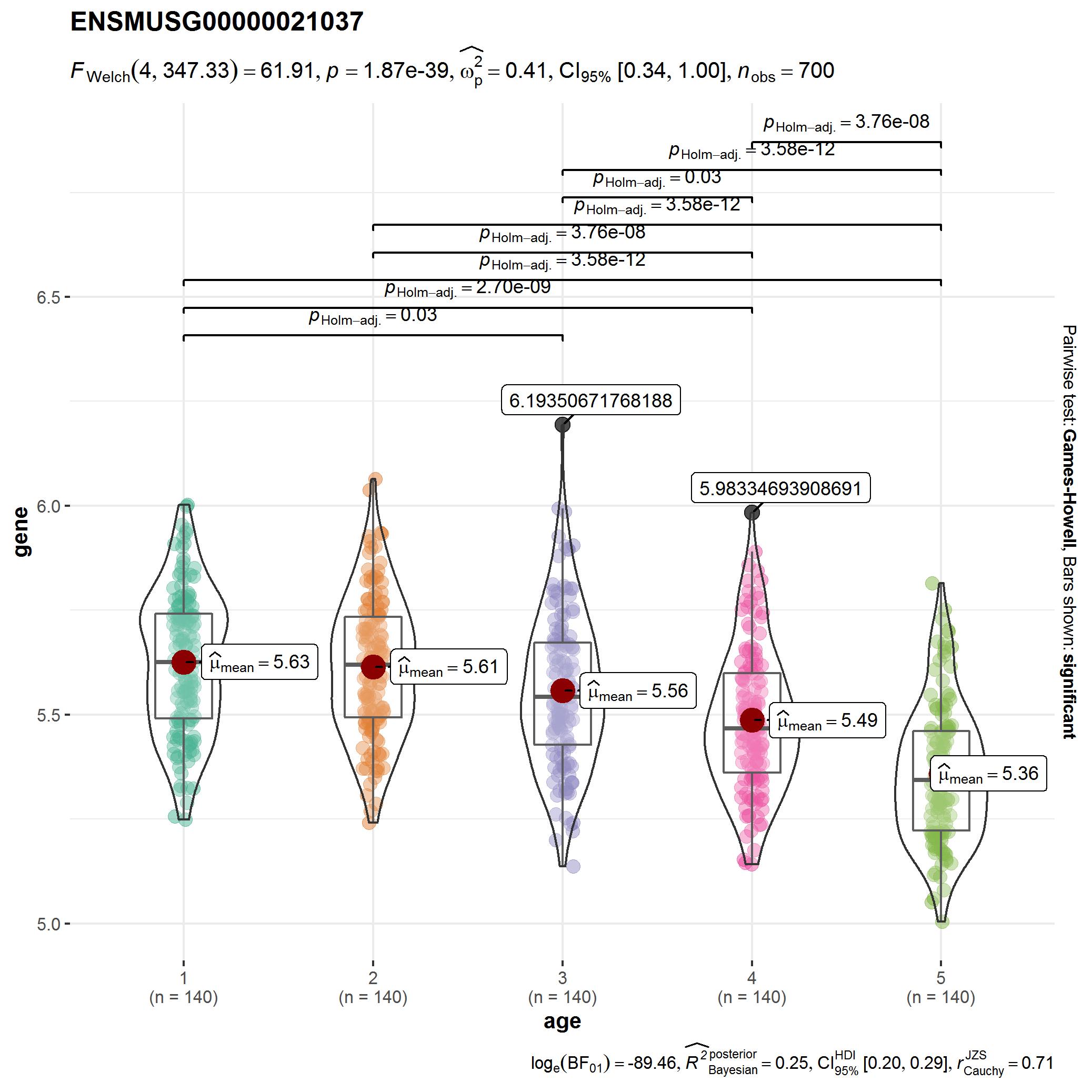

Supplement: Supplementary file 25 — Data S1–S6. [file ACEL-23-e14268-s017.zip › Data S1/ENSMUSG00000021037.jpeg]

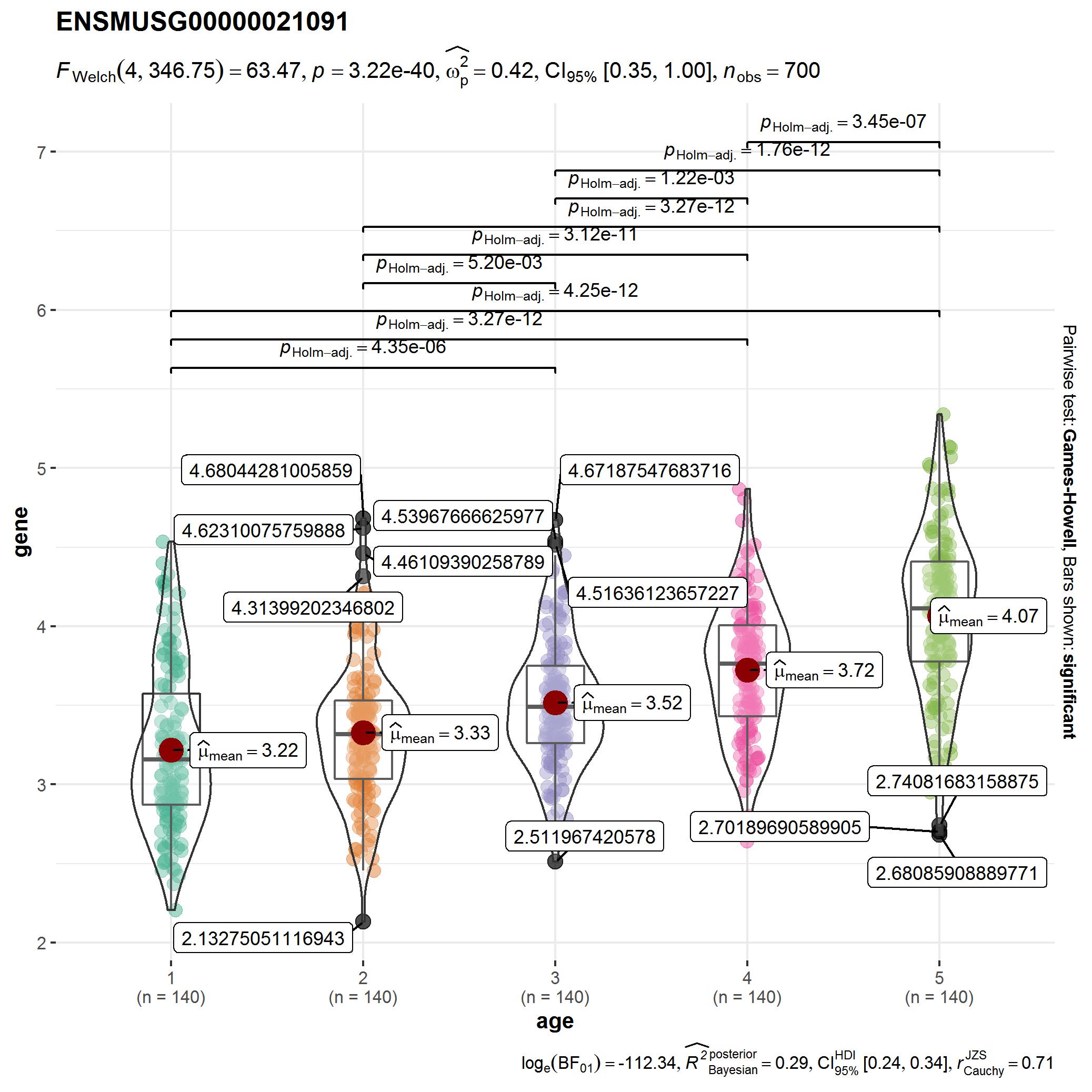

Supplement: Supplementary file 25 — Data S1–S6. [file ACEL-23-e14268-s017.zip › Data S1/ENSMUSG00000021091.jpeg]

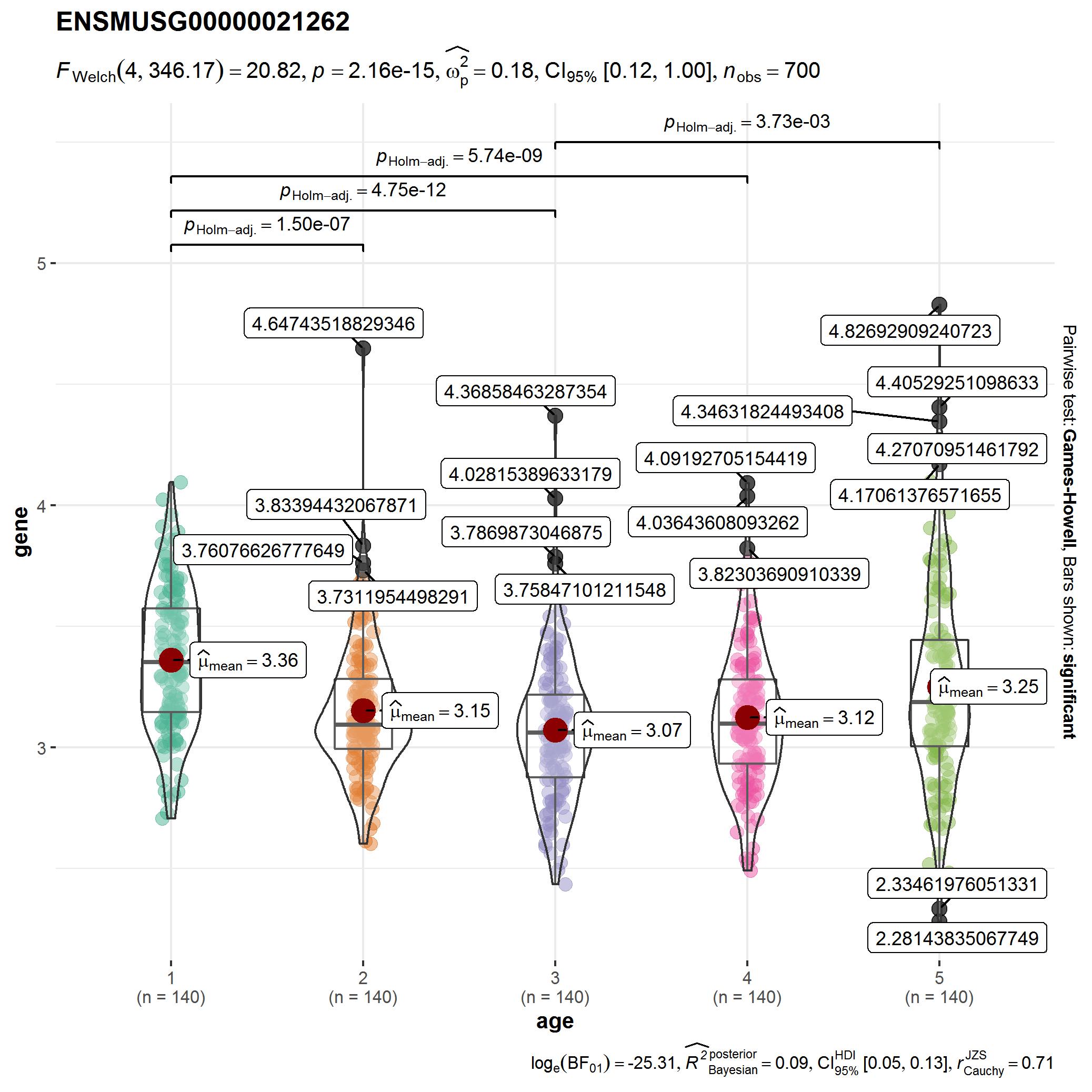

Supplement: Supplementary file 25 — Data S1–S6. [file ACEL-23-e14268-s017.zip › Data S1/ENSMUSG00000021262.jpeg]

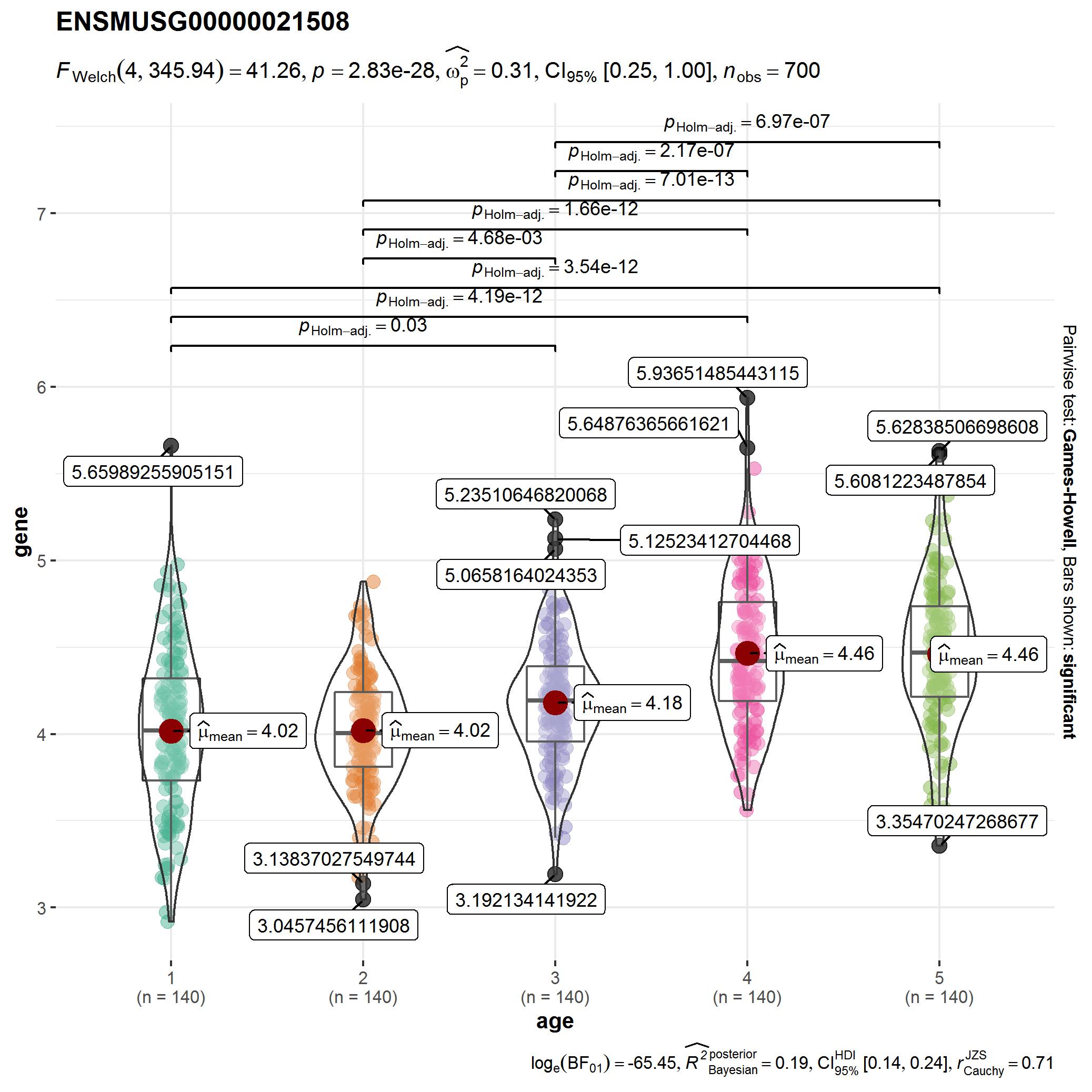

Supplement: Supplementary file 25 — Data S1–S6. [file ACEL-23-e14268-s017.zip › Data S1/ENSMUSG00000021508.jpeg]

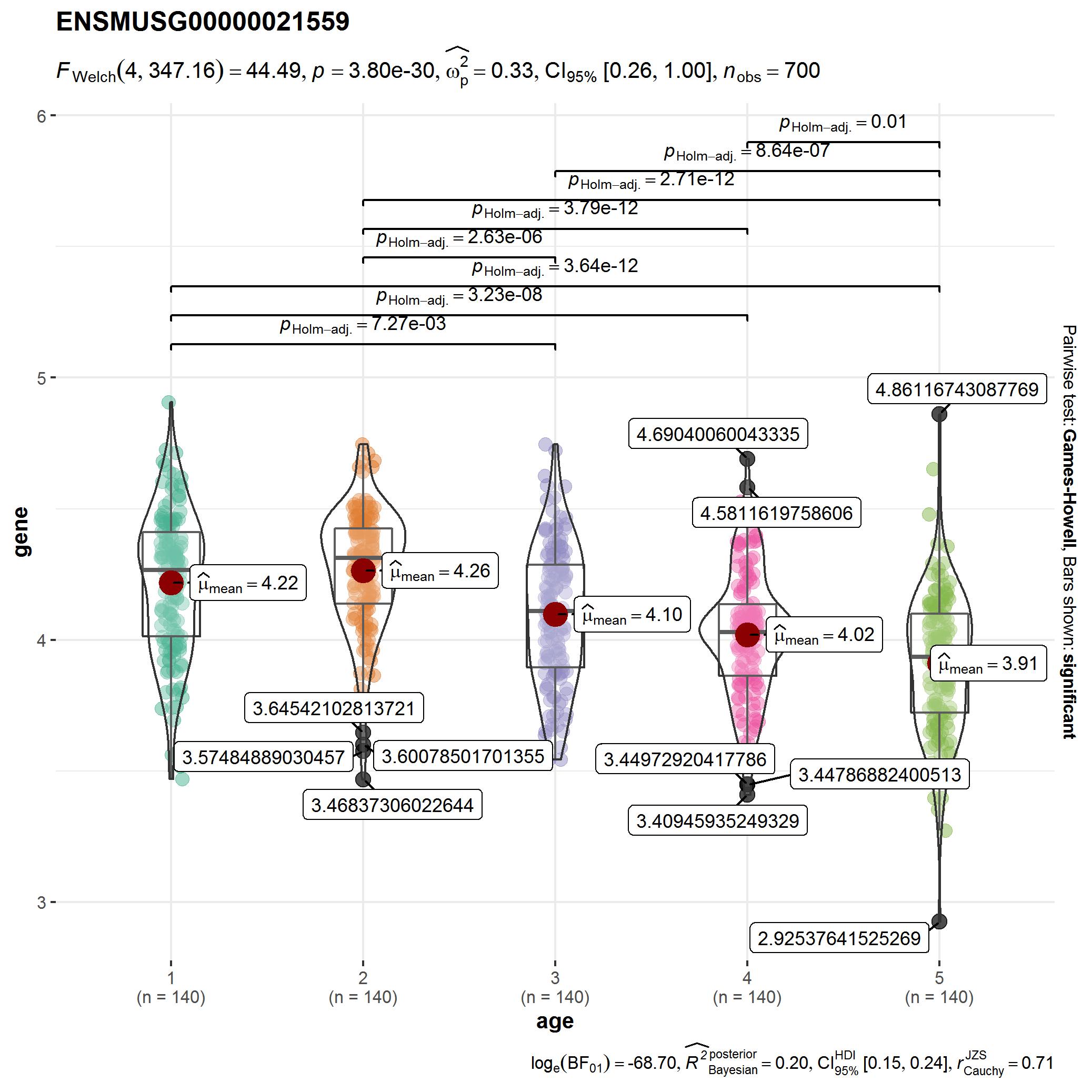

Supplement: Supplementary file 25 — Data S1–S6. [file ACEL-23-e14268-s017.zip › Data S1/ENSMUSG00000021559.jpeg]

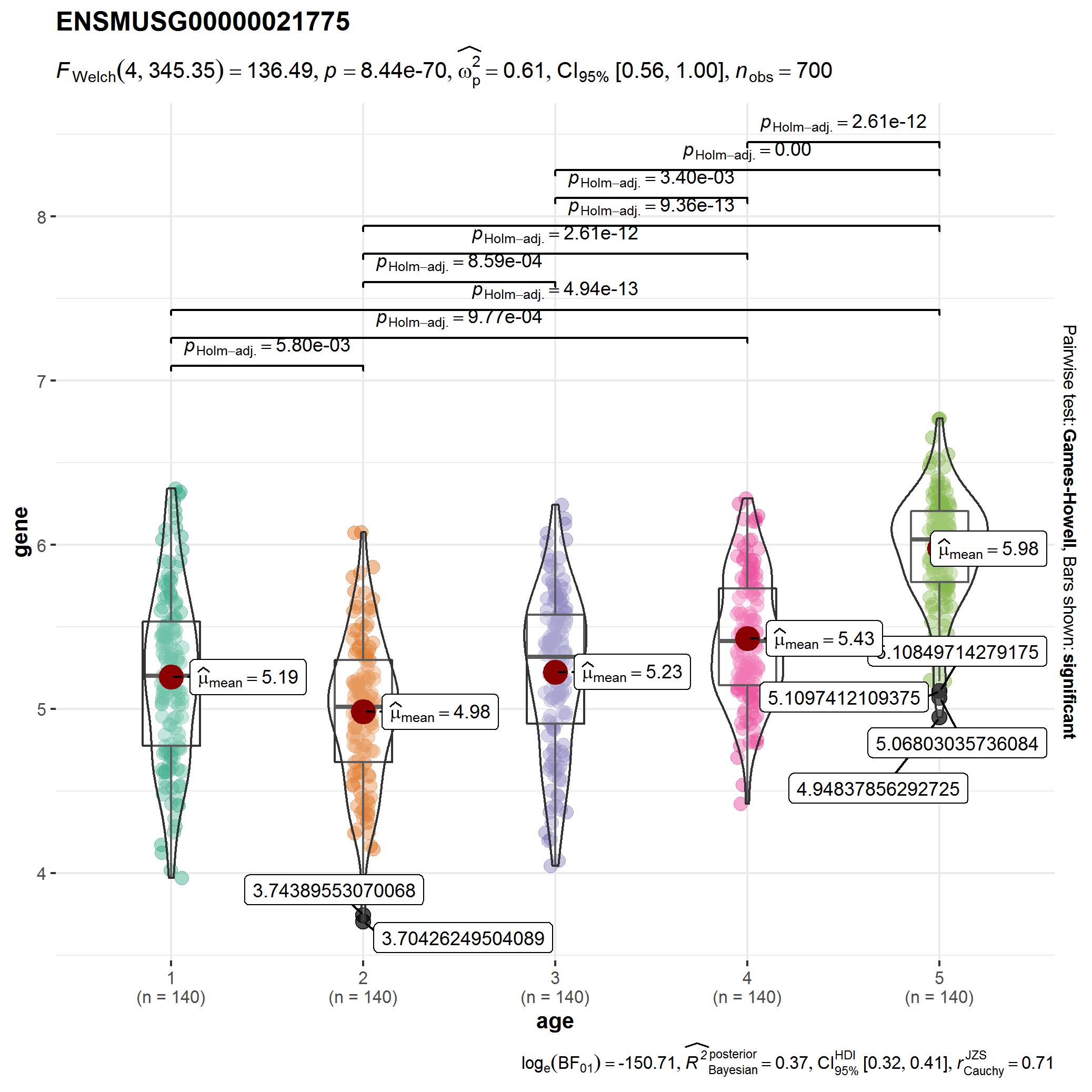

Supplement: Supplementary file 25 — Data S1–S6. [file ACEL-23-e14268-s017.zip › Data S1/ENSMUSG00000021775.jpeg]

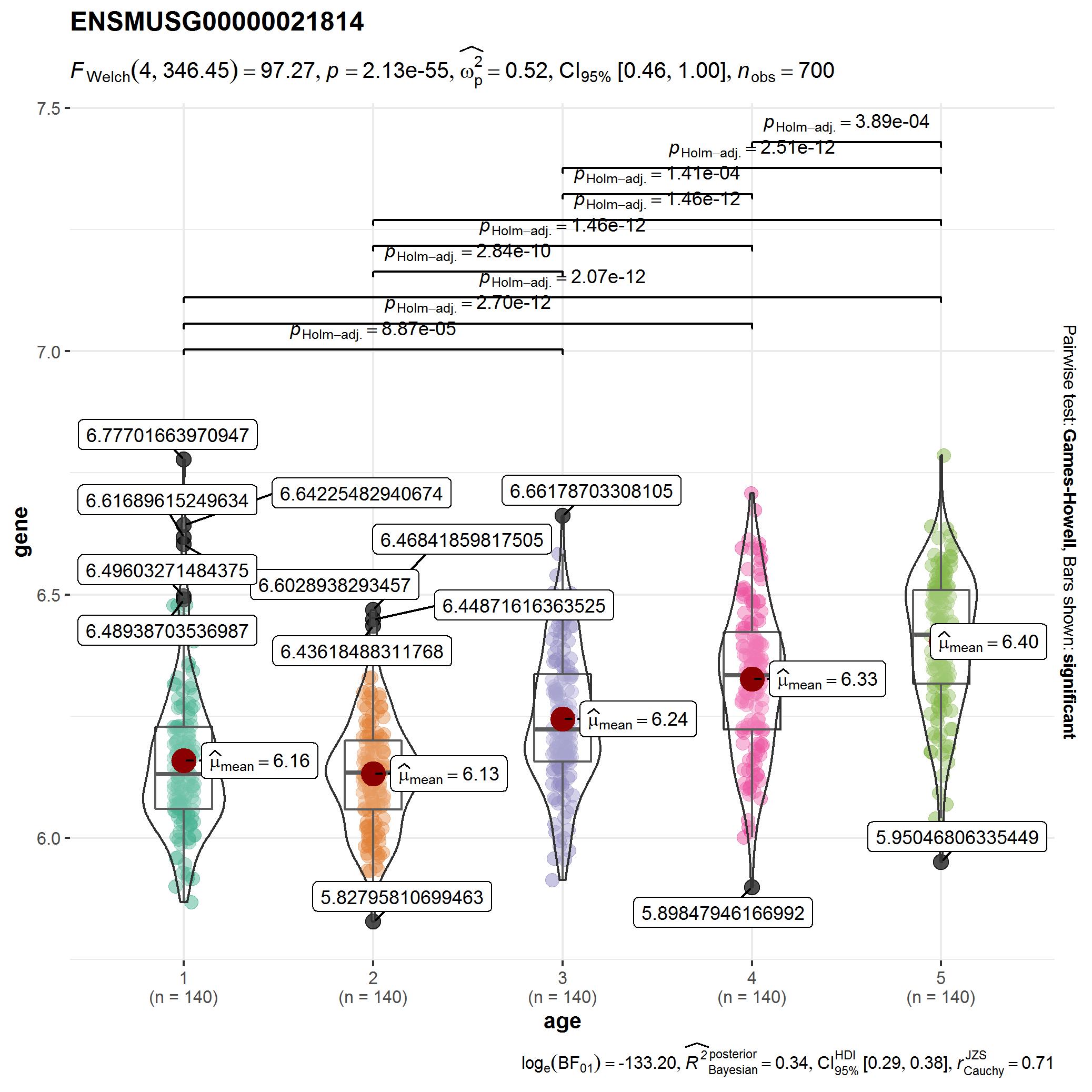

Supplement: Supplementary file 25 — Data S1–S6. [file ACEL-23-e14268-s017.zip › Data S1/ENSMUSG00000021814.jpeg]

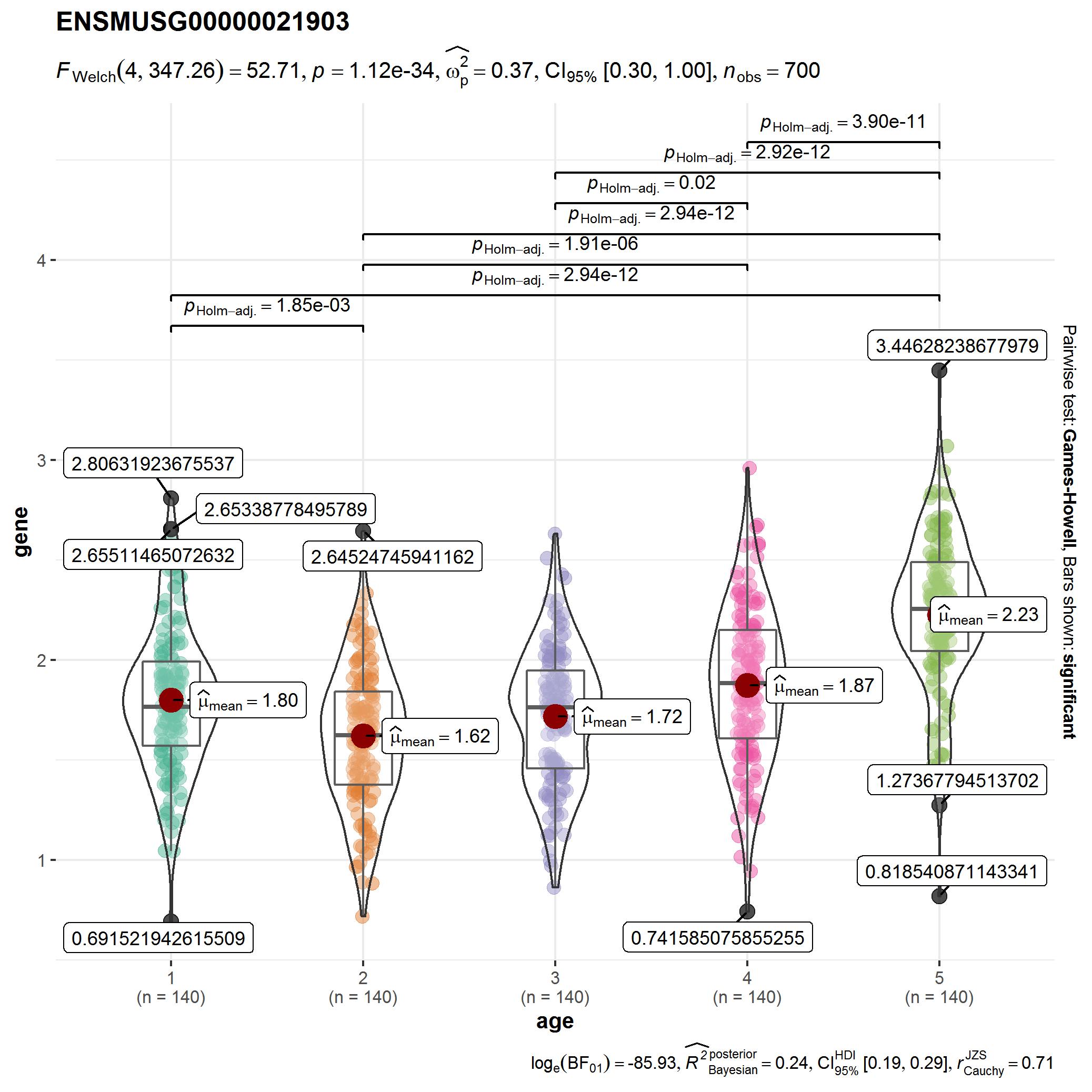

Supplement: Supplementary file 25 — Data S1–S6. [file ACEL-23-e14268-s017.zip › Data S1/ENSMUSG00000021903.jpeg]

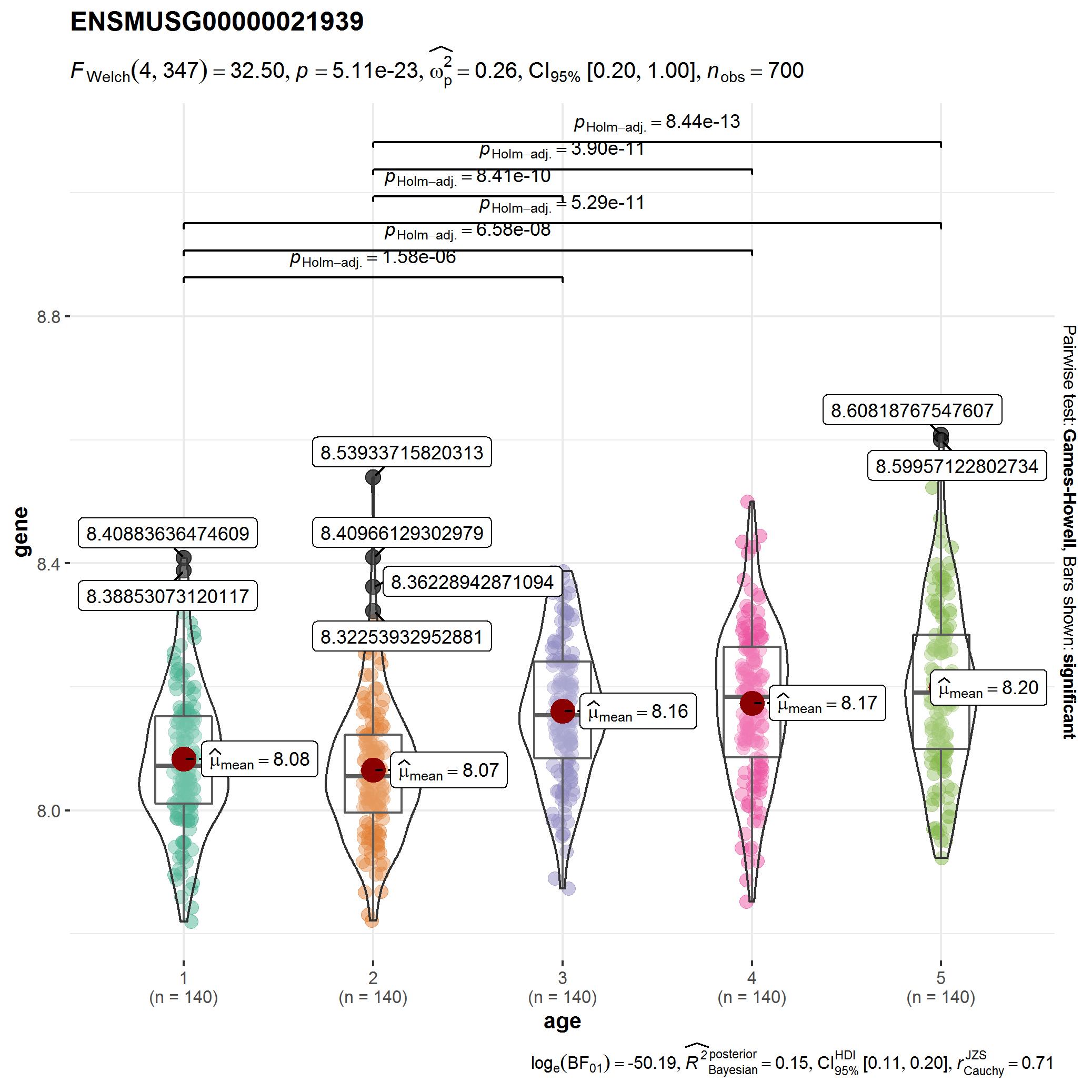

Supplement: Supplementary file 25 — Data S1–S6. [file ACEL-23-e14268-s017.zip › Data S1/ENSMUSG00000021939.jpeg]

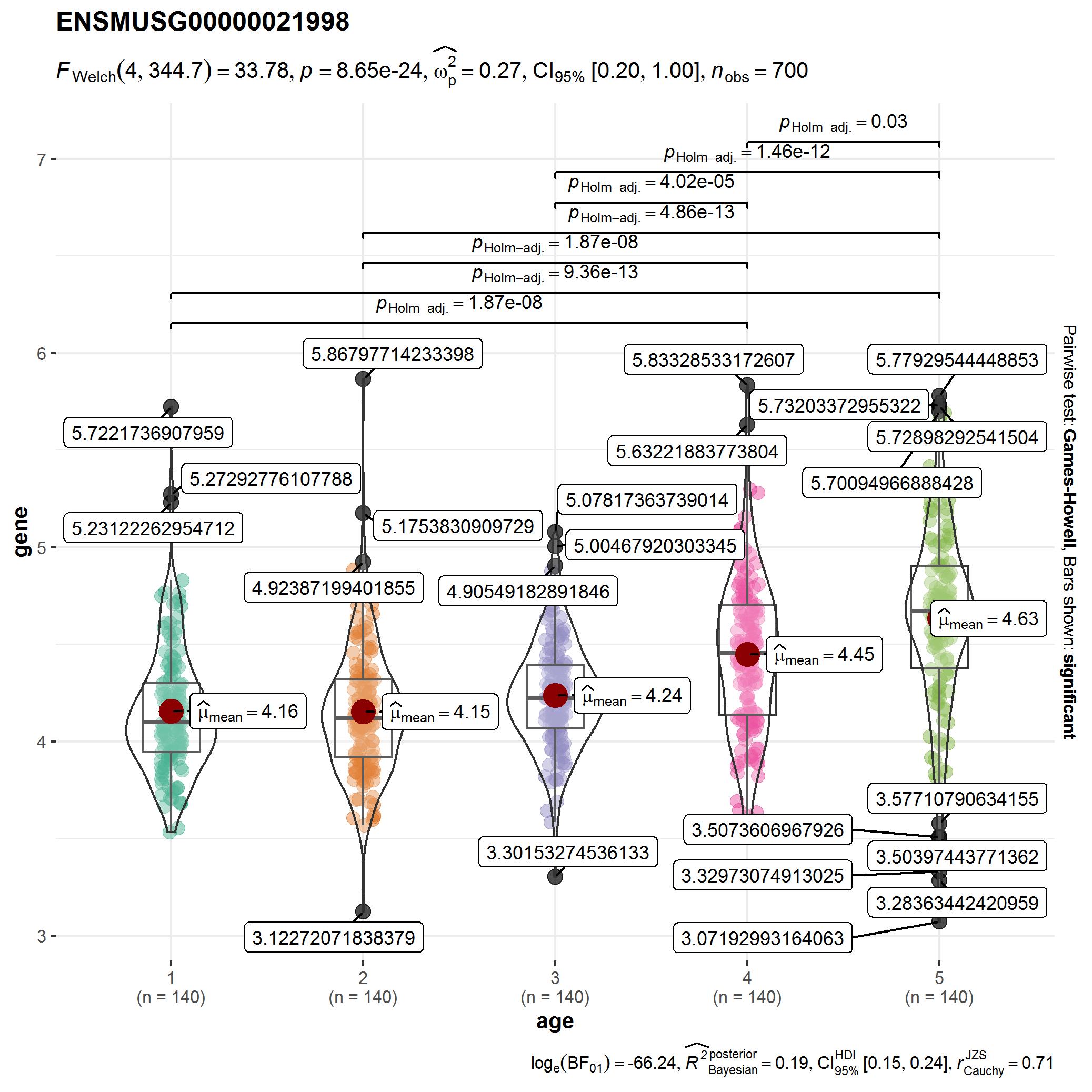

Supplement: Supplementary file 25 — Data S1–S6. [file ACEL-23-e14268-s017.zip › Data S1/ENSMUSG00000021998.jpeg]

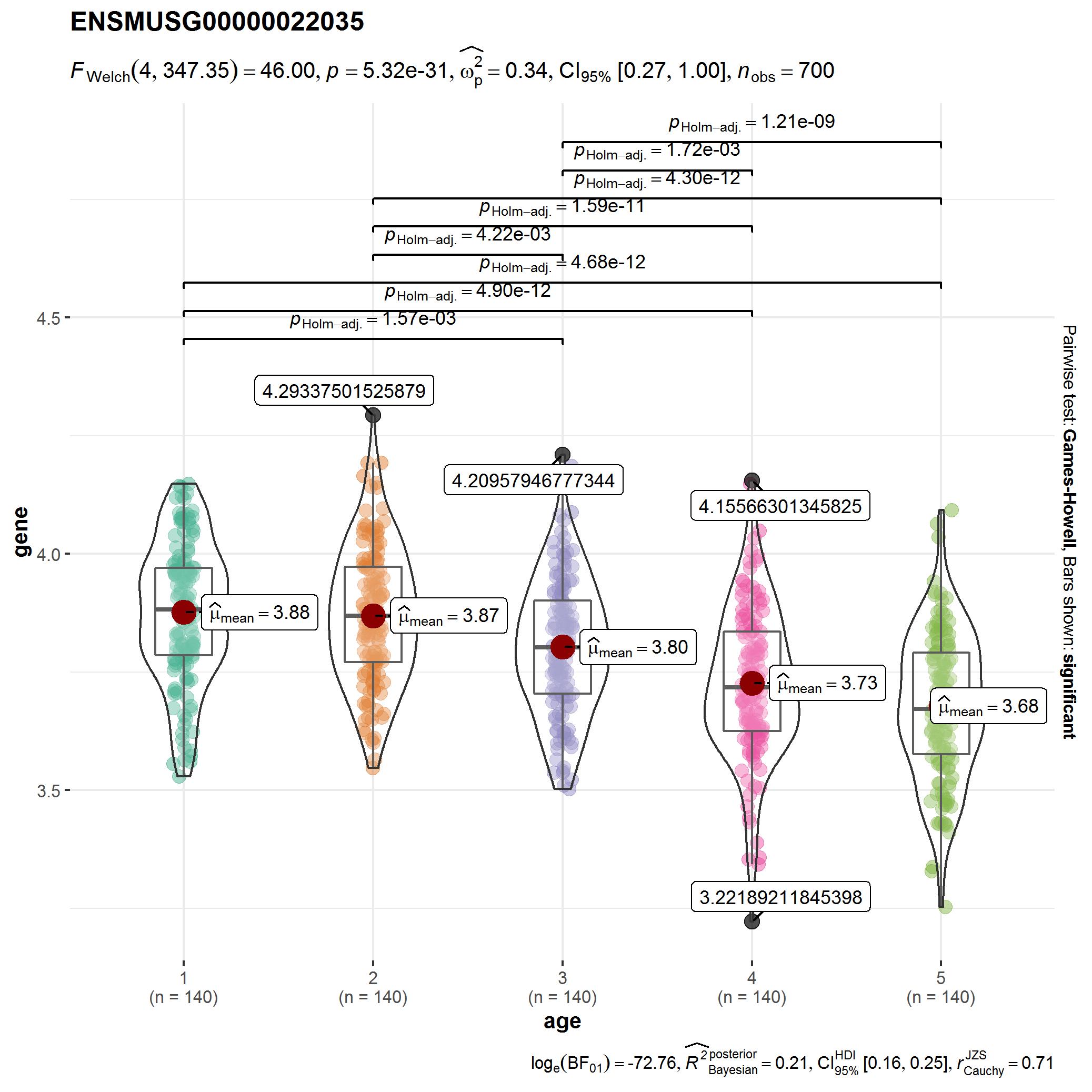

Supplement: Supplementary file 25 — Data S1–S6. [file ACEL-23-e14268-s017.zip › Data S1/ENSMUSG00000022035.jpeg]

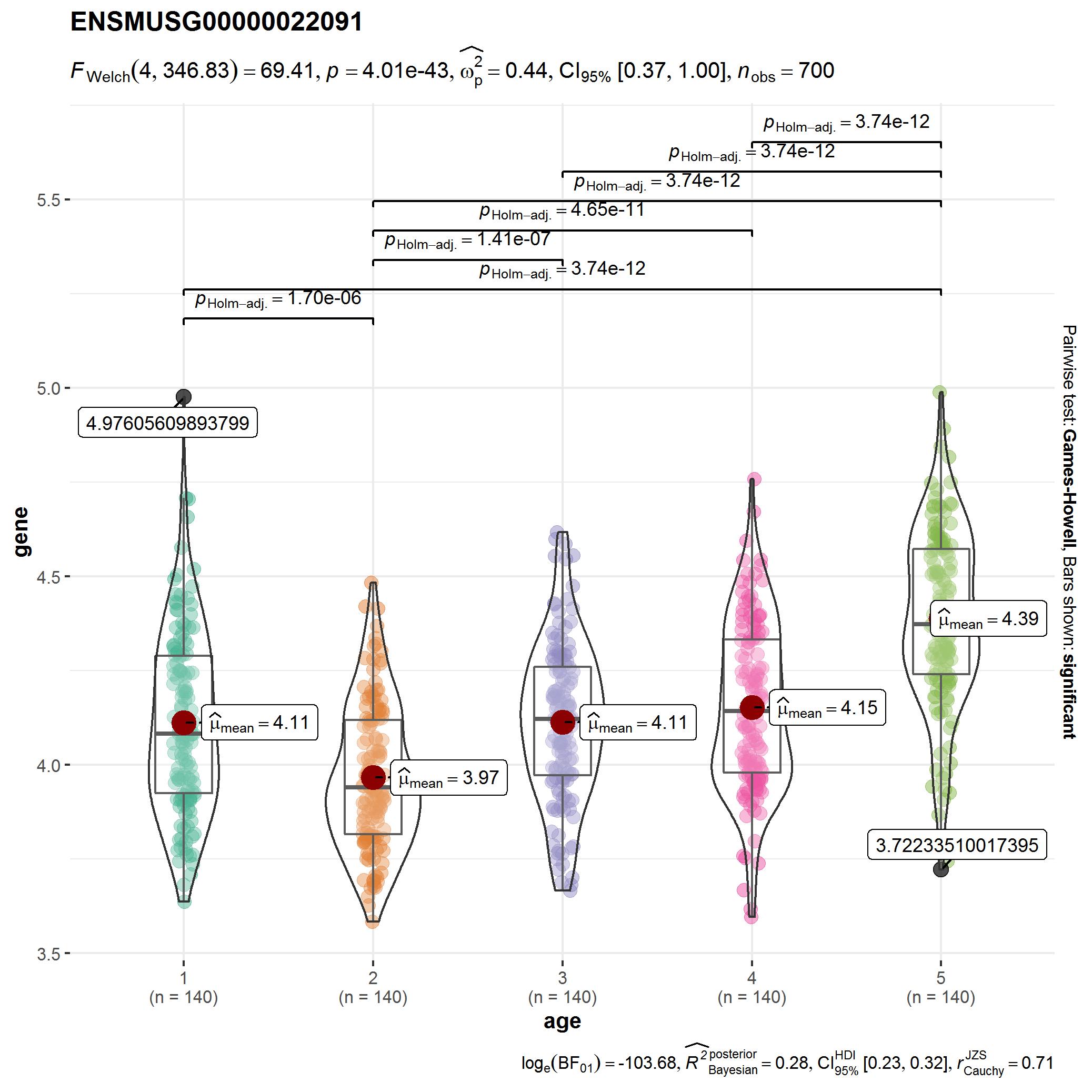

Supplement: Supplementary file 25 — Data S1–S6. [file ACEL-23-e14268-s017.zip › Data S1/ENSMUSG00000022091.jpeg]

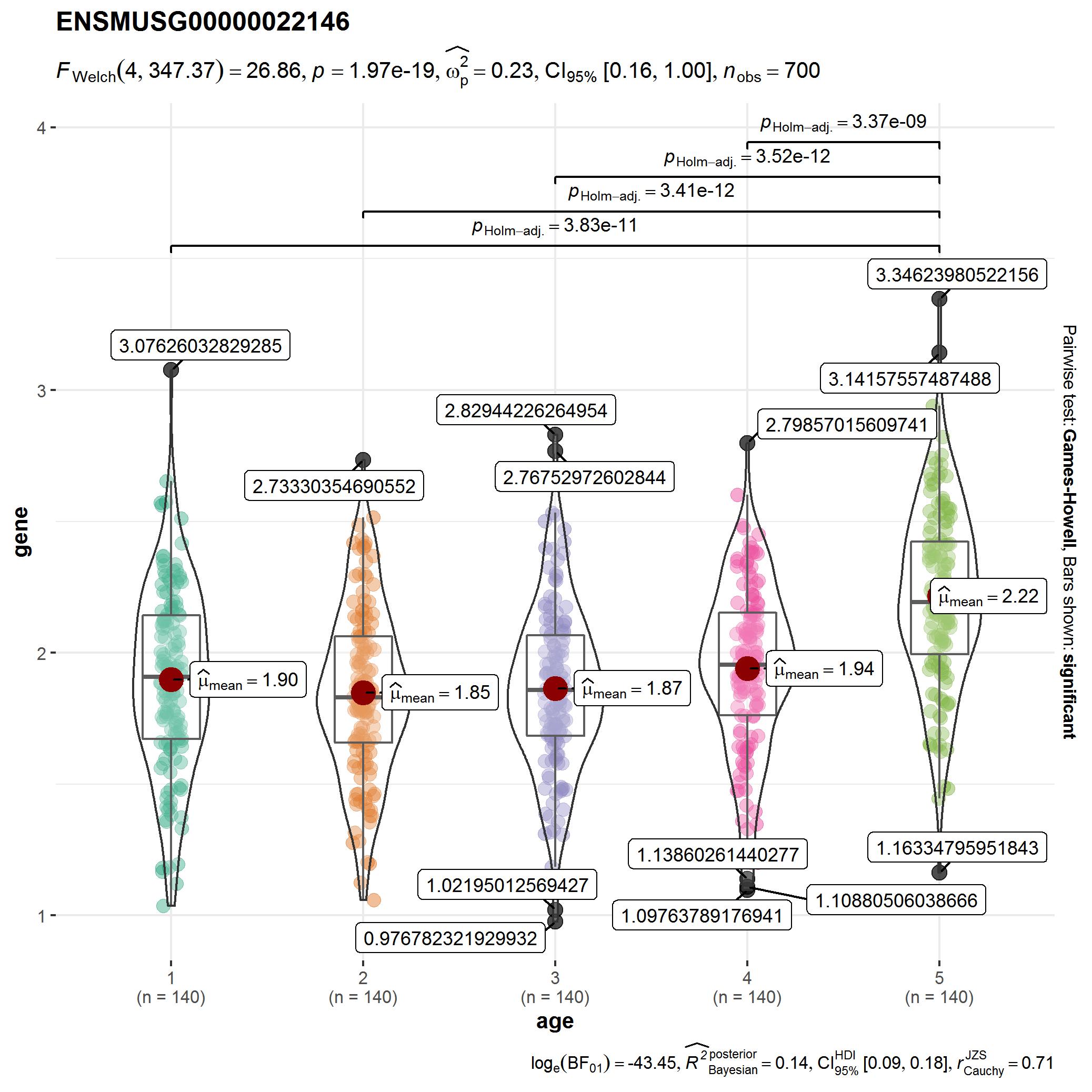

Supplement: Supplementary file 25 — Data S1–S6. [file ACEL-23-e14268-s017.zip › Data S1/ENSMUSG00000022146.jpeg]
