# Supplementary material for: The circadian rhythm: A key variable in aging?
Source: Aging Cell. 2024 Jul 30;23(11):e14268. doi: 10.1111/acel.14268 (PMC11561671; doi:10.1111/acel.14268)
Supplement: Supplementary file 2 — Figure S2. [file ACEL-23-e14268-s015.zip › acel14268-sup-0001-FigureS2.docx]

Figure S2. Hierarchical clustering during aging.
(A) Cross-organ zScore expression profiles averaged for age by the median. (B) Tissue-specific zScore alterations for the brain (B), heart (H), liver (L), kidney (K), colon (C), muscle (M), and aorta (A) by the median of 20 samples each (including the both corrected strains and sexes). The colors on the left are assigned to the respective module eigengene (MEs). Nonclustered hits were grouped into MEg. Row trees by Pearson correlation: Number of clusters (3000), the maximum number of iterations (100), and the number of restarts (10).
