# Supplementary material for: The circadian rhythm: A key variable in aging?
Source: Aging Cell. 2024 Jul 30;23(11):e14268. doi: 10.1111/acel.14268 (PMC11561671; doi:10.1111/acel.14268)
Supplement: Supplementary file 3 — Figure S3. [file ACEL-23-e14268-s021.zip › acel14268-sup-0003-FigureS3.docx]

Figure S3. Distinct network characteristics of CVA features.
No distinct grouping of ME_4_ transcripts is detected. However, there is frequent adjacency to ME_3_ or ME_5_ clusters in the StringDB-based network. A conspicuous sH in the network center consists of Runx1, Tcf12, Dnmt3a, Ezh2, Bmi1, Ccnd2, Cdk2, Cdkn1b, and Foxo3. The edge thickness and edge-weighted spring-embedded layout arrangement were based on the interaction score (confidence from 0.4 to 1). The size of nodes is based on the iteration average of meanImp. CVA, categorical-dependent variable analysis
