# Supplementary material for: The circadian rhythm: A key variable in aging?
Source: Aging Cell. 2024 Jul 30;23(11):e14268. doi: 10.1111/acel.14268 (PMC11561671; doi:10.1111/acel.14268)
Supplement: Supplementary file 4 — Figure S4. [file ACEL-23-e14268-s003.zip › acel14268-sup-0004-FigureS4.docx]

Figure S4. Organ-specific impact of CiR genes.
Association strength (meanImp) per transcript with aging for each organ is represented. For multiple iterations, the average of meanImp was chosen. Organ data were processed individually according to the identical methods as in the combined analysis (per gene 95% of counts ≥20, data correction by ComBat for strain and subsequently for sex, 30 iterations in Boruta setting ntree to 3500). CiR, circadian rhythms.
