# Supplementary figures and images for: The circadian rhythm: A key variable in aging? (part 3 of 3)
Source: Aging Cell. 2024 Jul 30;23(11):e14268. doi: 10.1111/acel.14268 (PMC11561671; doi:10.1111/acel.14268)

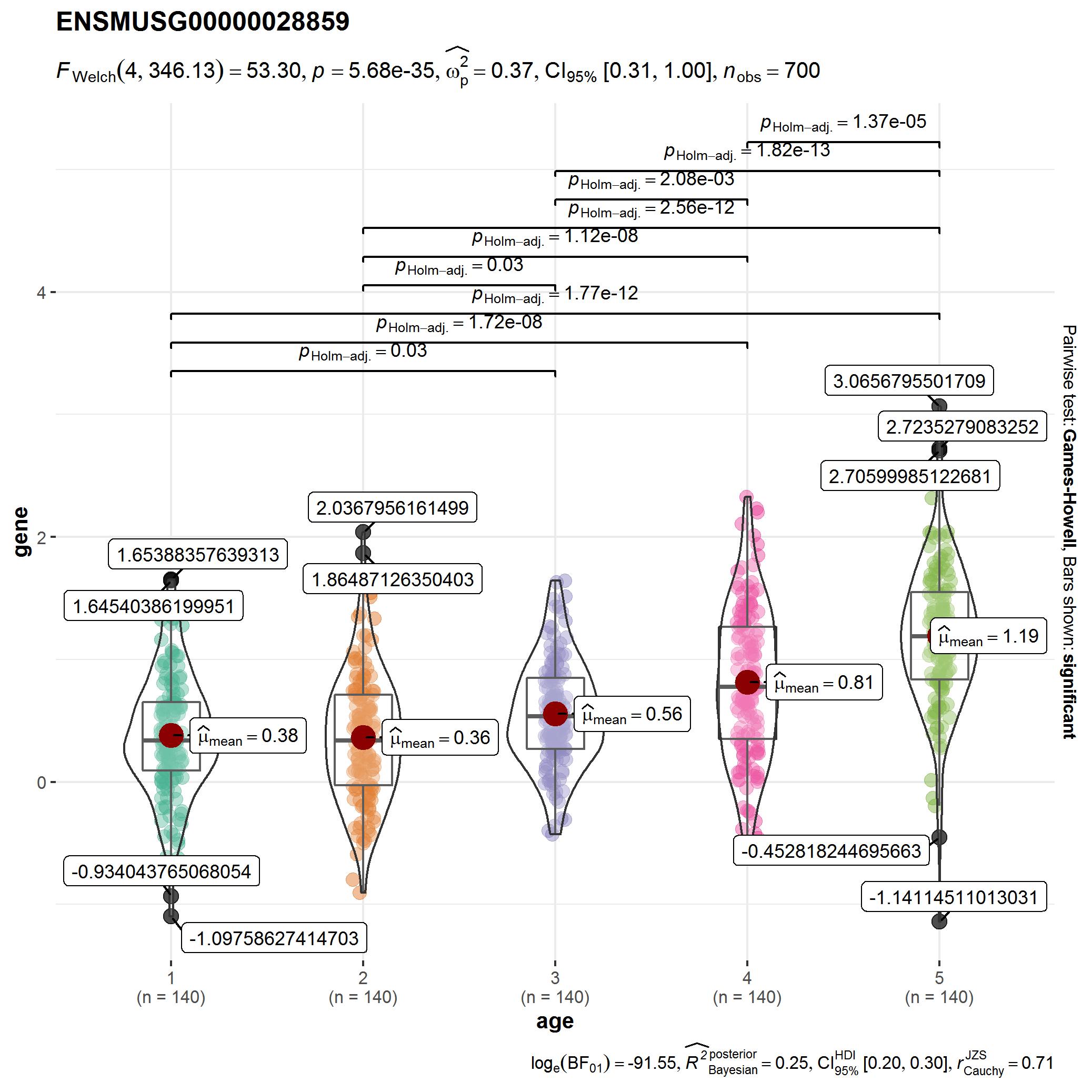

Supplement: Supplementary file 25 — Data S1–S6. [file ACEL-23-e14268-s017.zip › Data S1/ENSMUSG00000028859.jpeg]

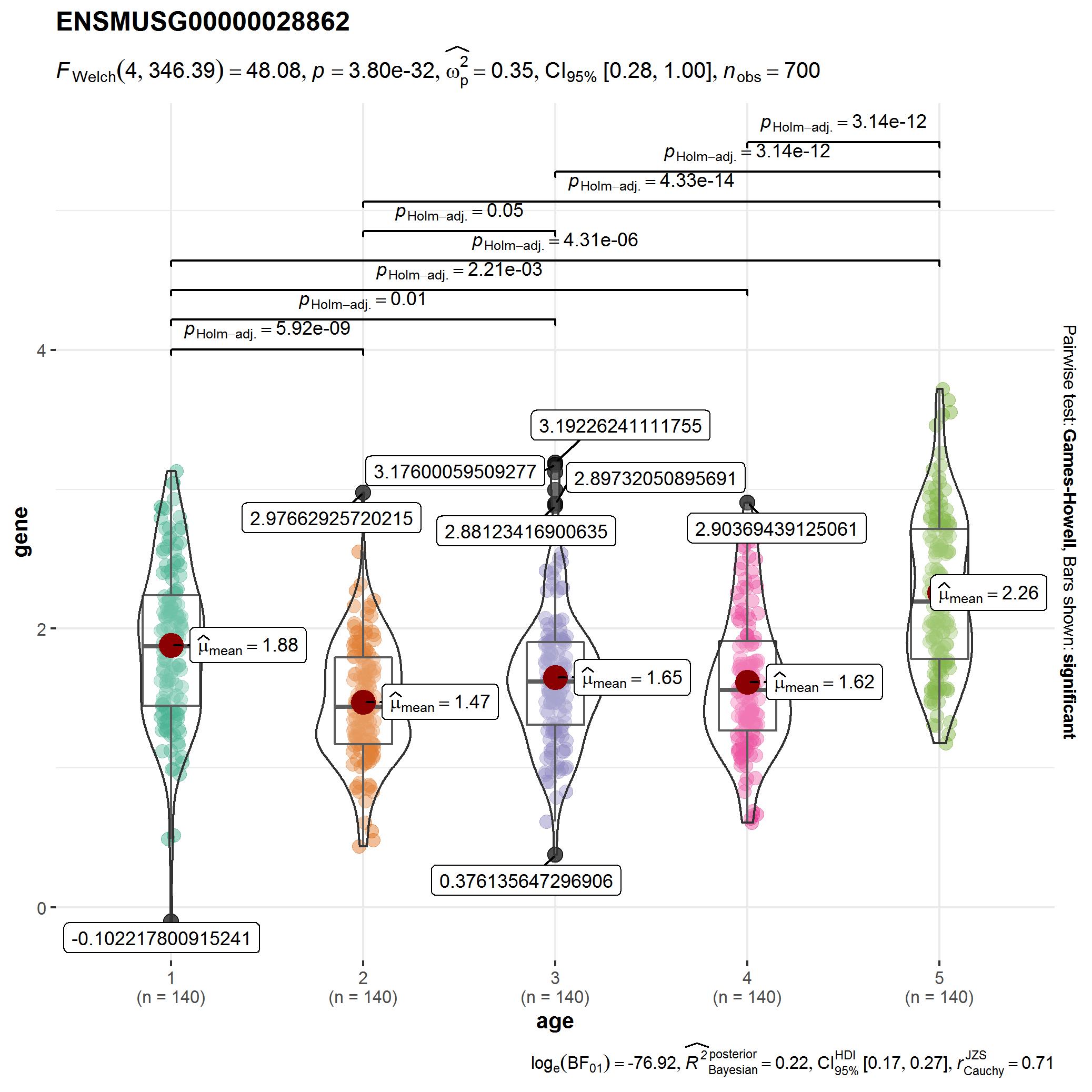

Supplement: Supplementary file 25 — Data S1–S6. [file ACEL-23-e14268-s017.zip › Data S1/ENSMUSG00000028862.jpeg]

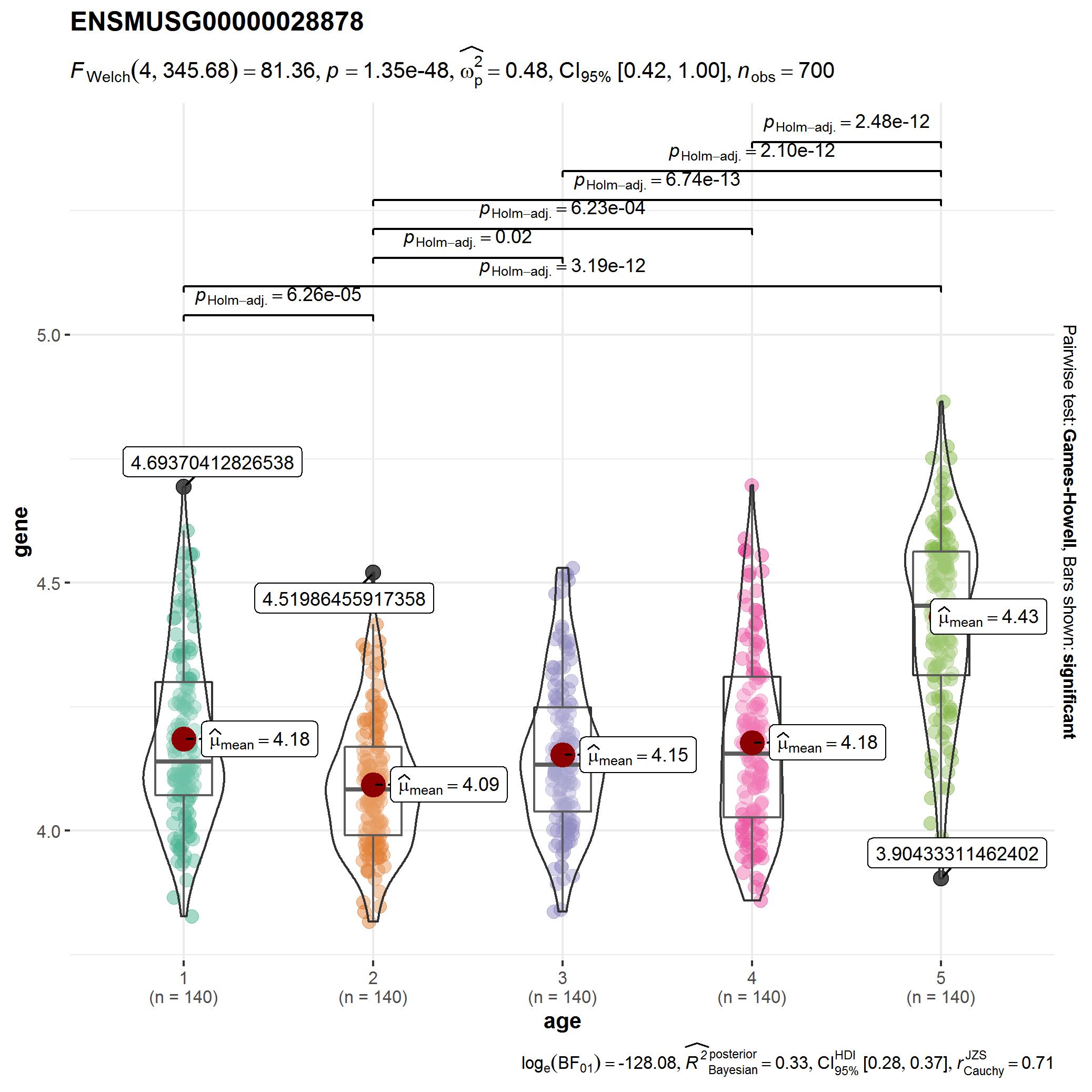

Supplement: Supplementary file 25 — Data S1–S6. [file ACEL-23-e14268-s017.zip › Data S1/ENSMUSG00000028878.jpeg]

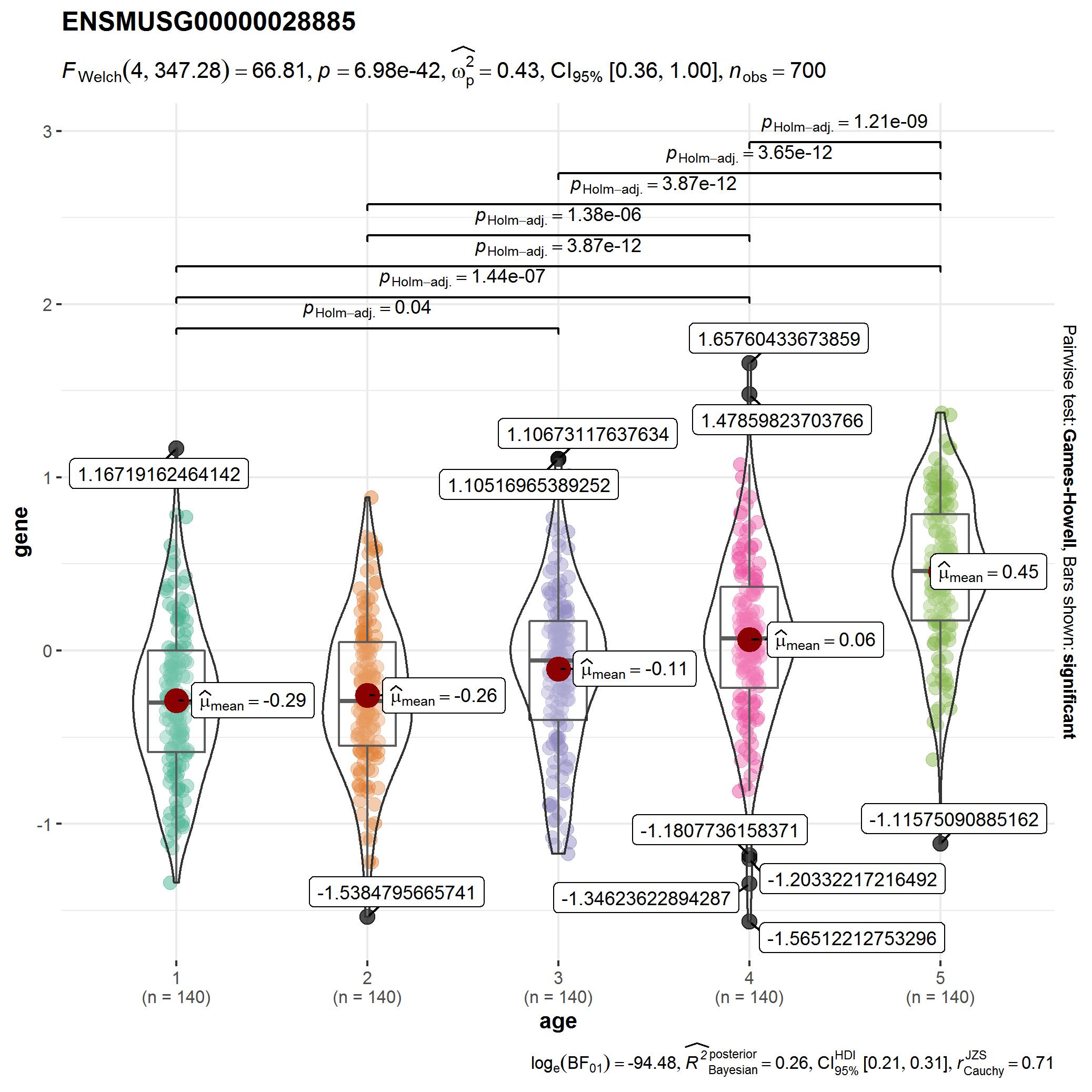

Supplement: Supplementary file 25 — Data S1–S6. [file ACEL-23-e14268-s017.zip › Data S1/ENSMUSG00000028885.jpeg]
