# Supplementary figures and images for: The circadian rhythm: A key variable in aging? (part 2 of 3)
Source: Aging Cell. 2024 Jul 30;23(11):e14268. doi: 10.1111/acel.14268 (PMC11561671; doi:10.1111/acel.14268)

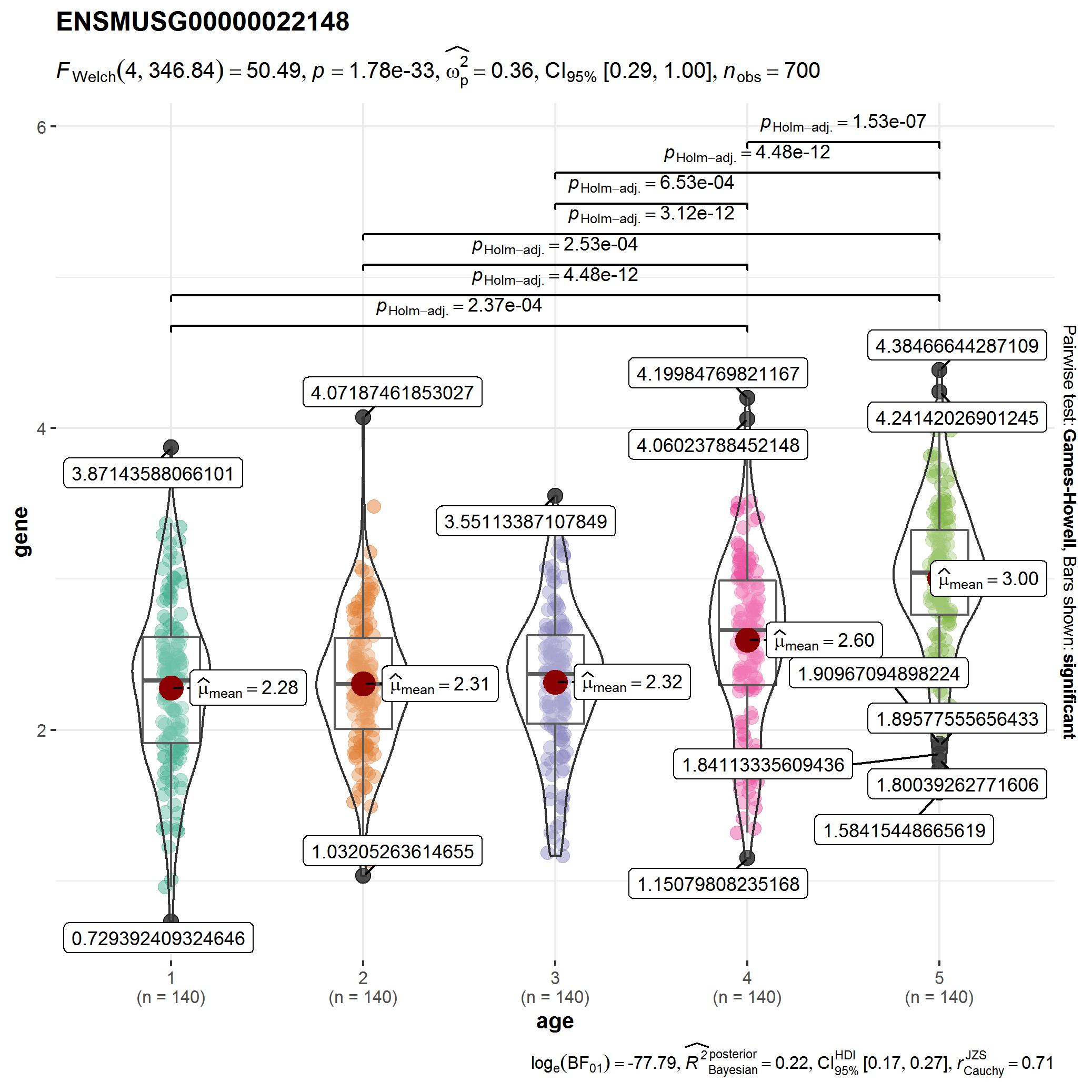

Supplement: Supplementary file 25 — Data S1–S6. [file ACEL-23-e14268-s017.zip › Data S1/ENSMUSG00000022148.jpeg]

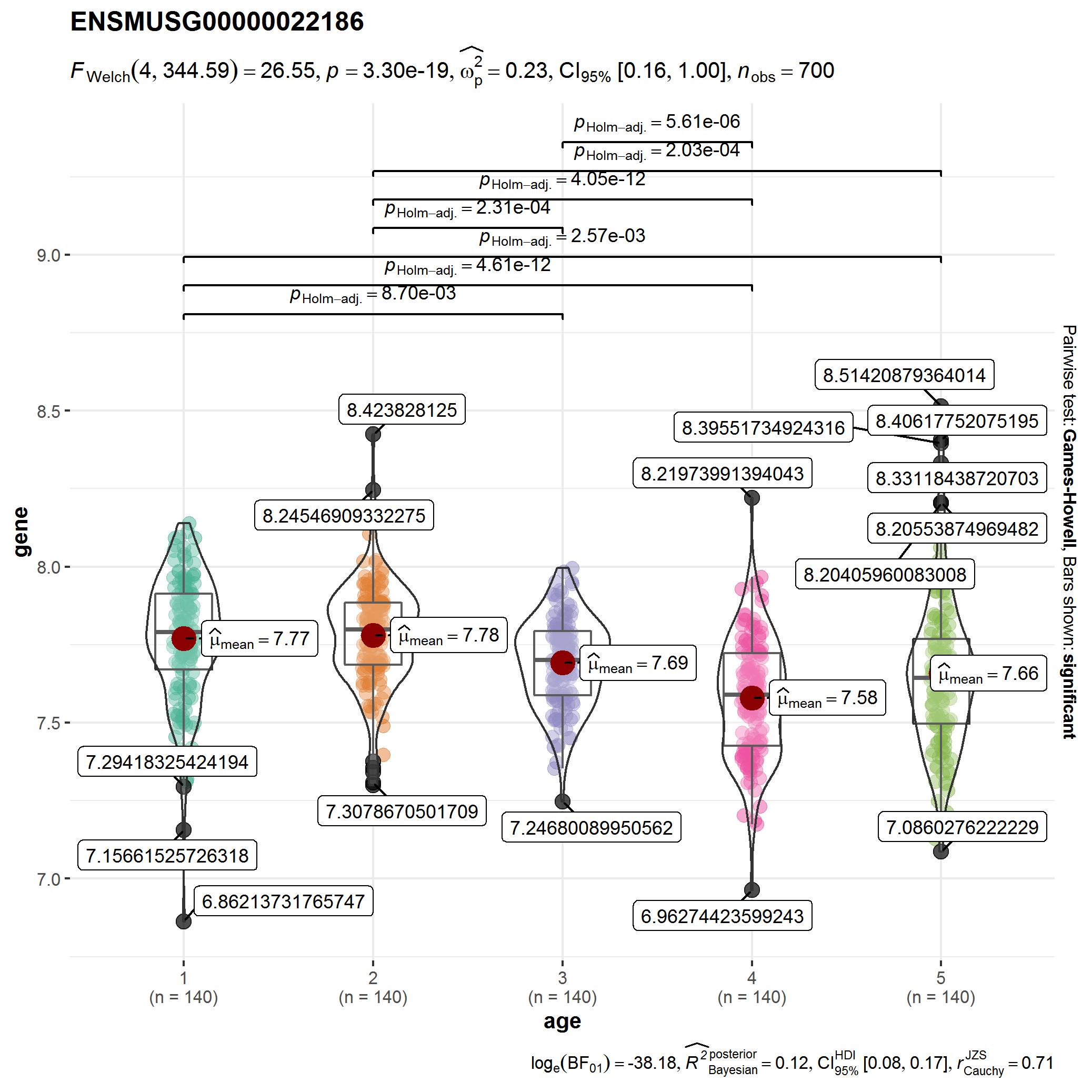

Supplement: Supplementary file 25 — Data S1–S6. [file ACEL-23-e14268-s017.zip › Data S1/ENSMUSG00000022186.jpeg]

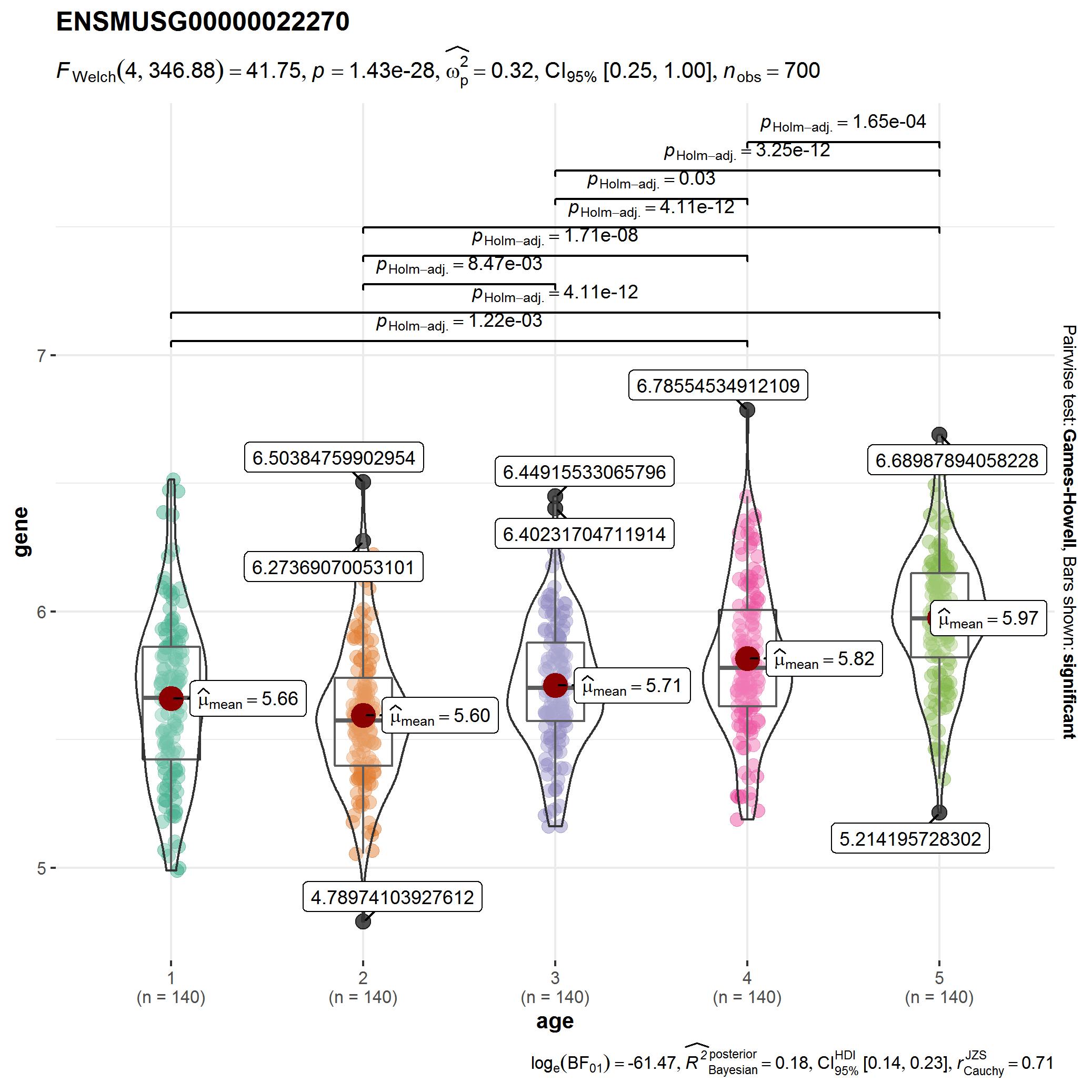

Supplement: Supplementary file 25 — Data S1–S6. [file ACEL-23-e14268-s017.zip › Data S1/ENSMUSG00000022270.jpeg]

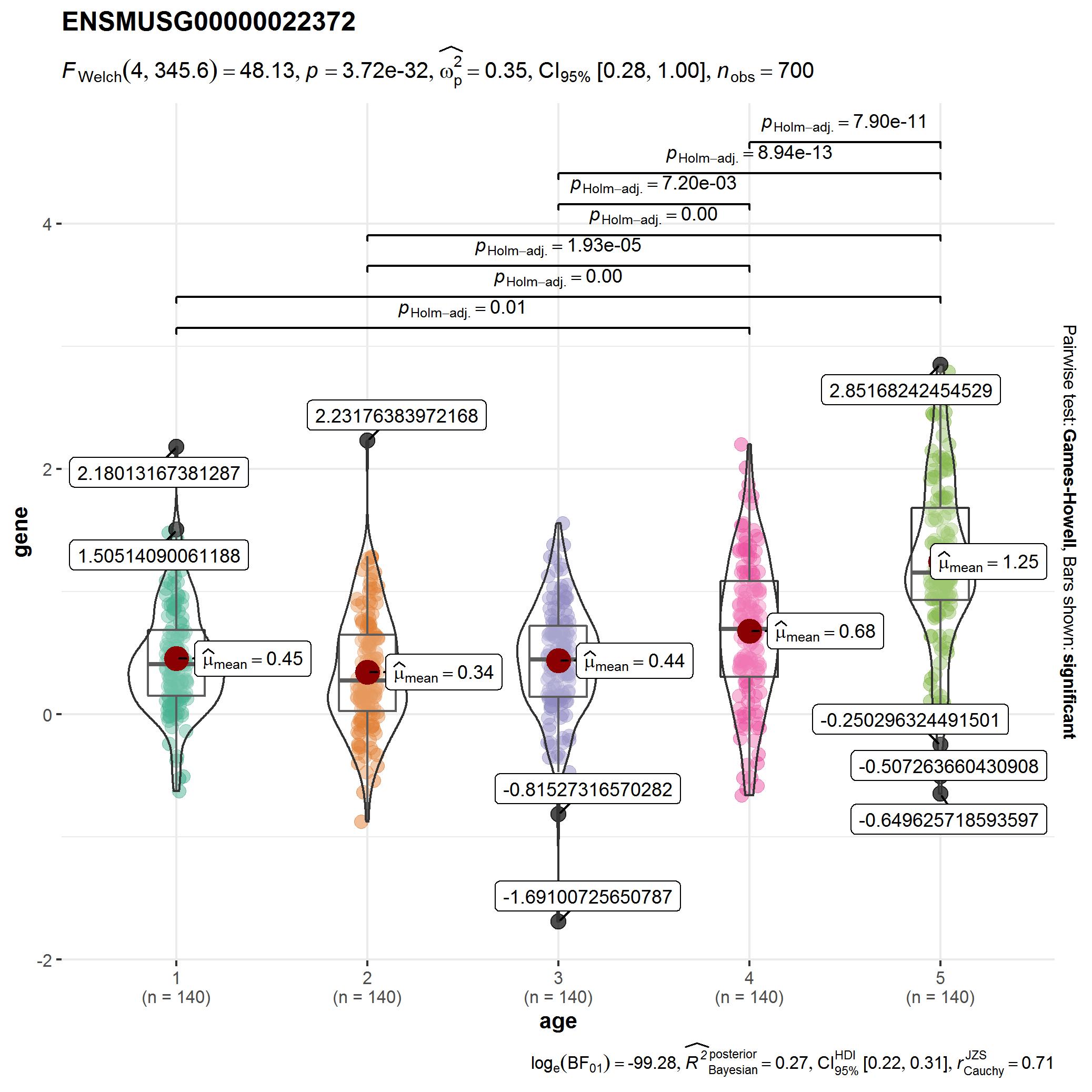

Supplement: Supplementary file 25 — Data S1–S6. [file ACEL-23-e14268-s017.zip › Data S1/ENSMUSG00000022372.jpeg]

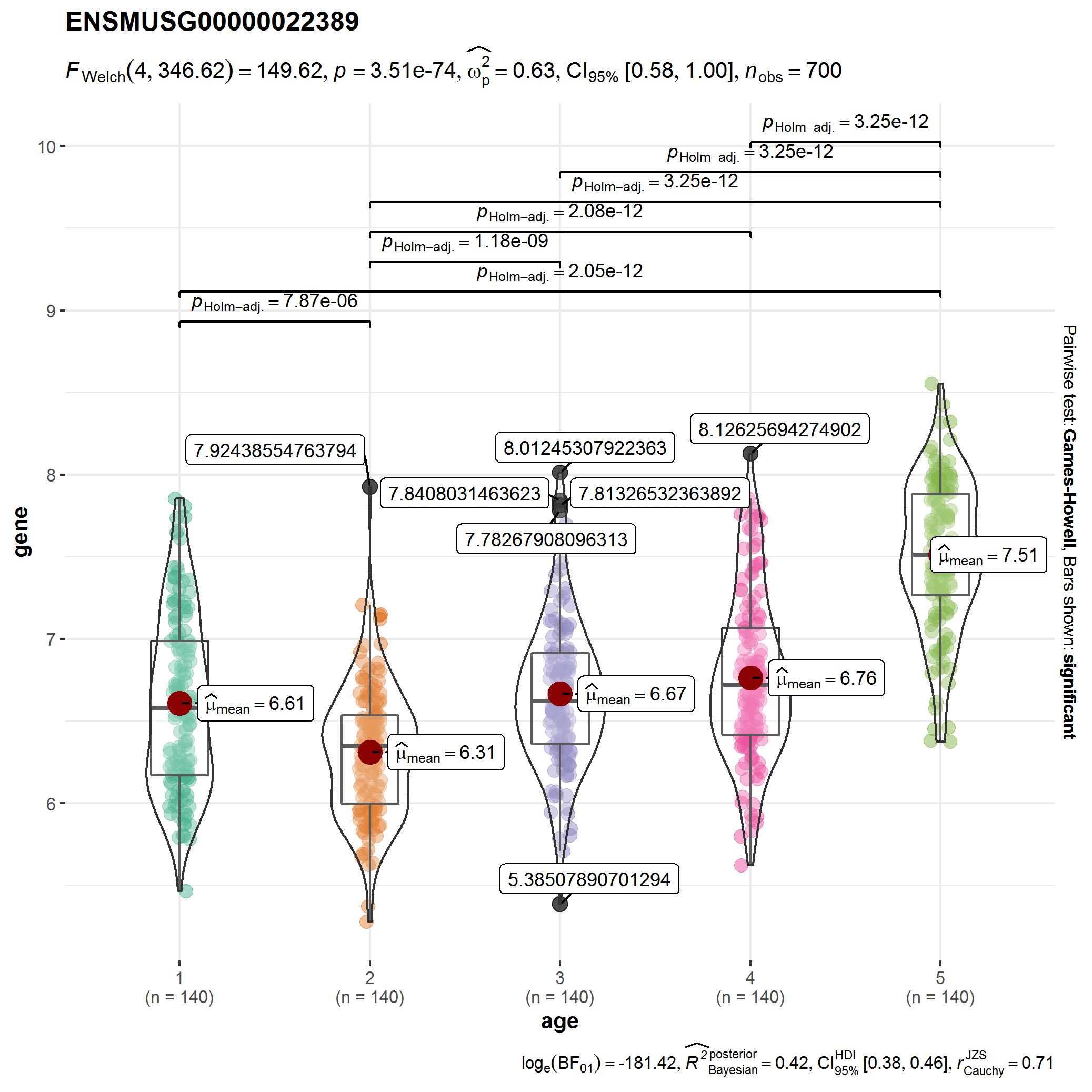

Supplement: Supplementary file 25 — Data S1–S6. [file ACEL-23-e14268-s017.zip › Data S1/ENSMUSG00000022389.jpeg]

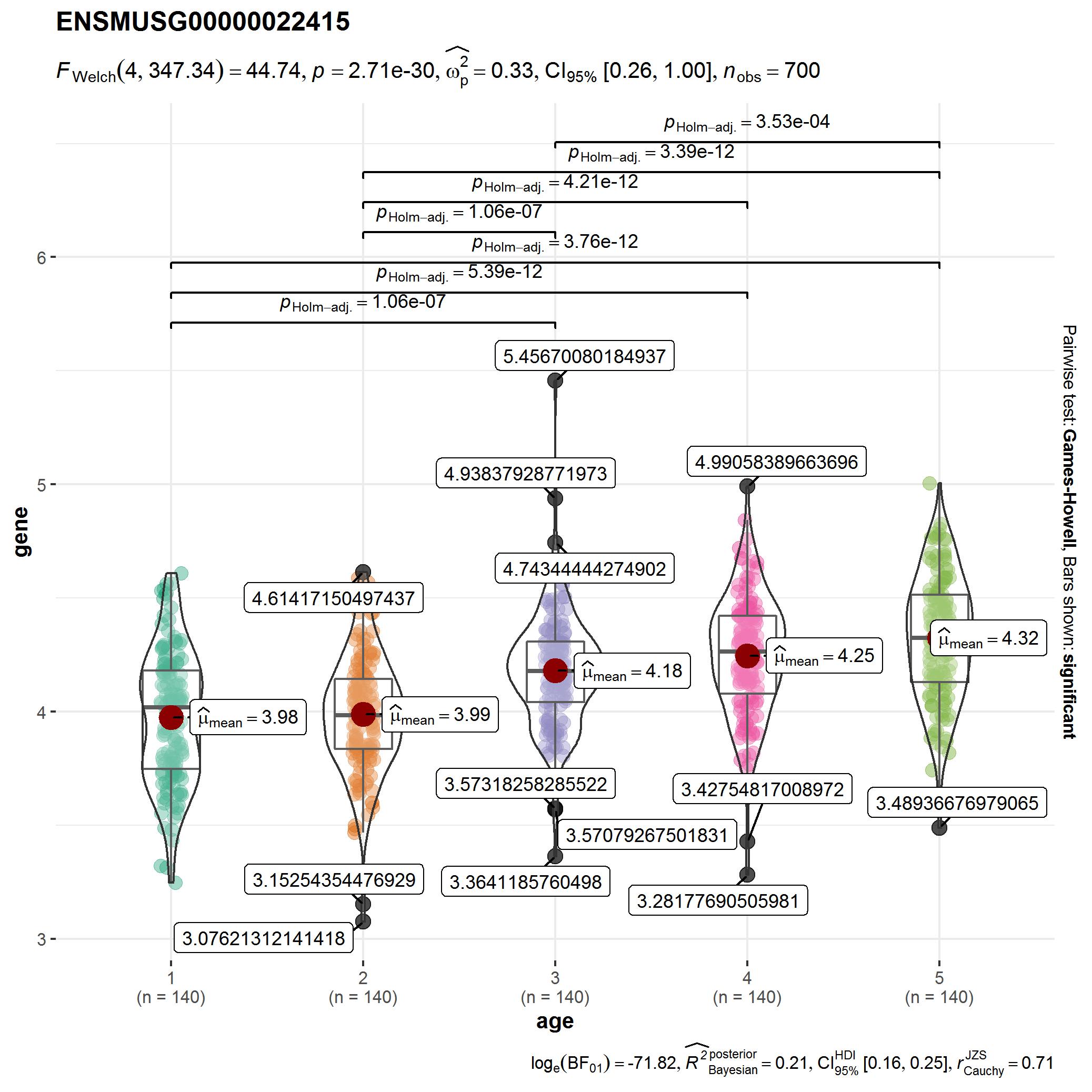

Supplement: Supplementary file 25 — Data S1–S6. [file ACEL-23-e14268-s017.zip › Data S1/ENSMUSG00000022415.jpeg]

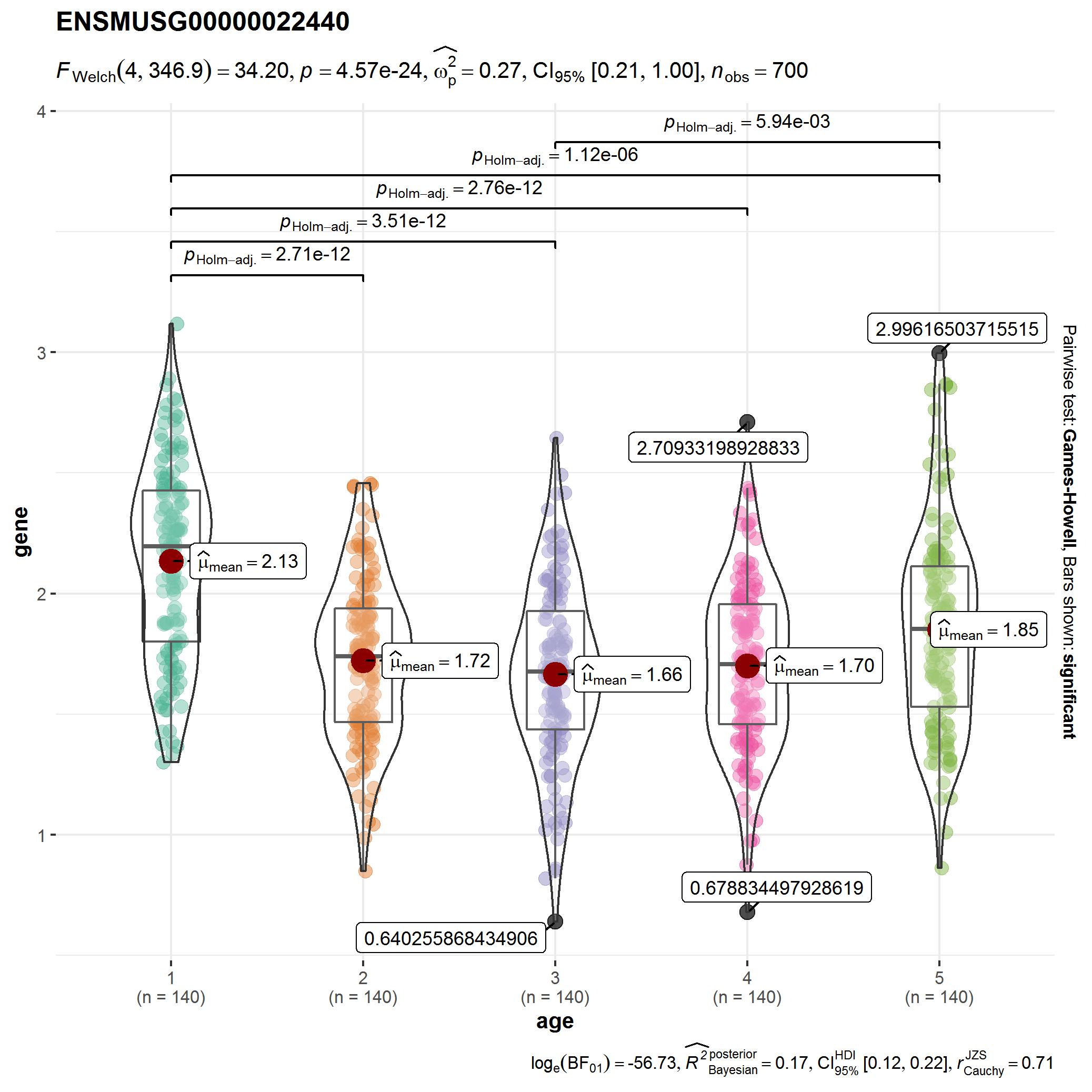

Supplement: Supplementary file 25 — Data S1–S6. [file ACEL-23-e14268-s017.zip › Data S1/ENSMUSG00000022440.jpeg]

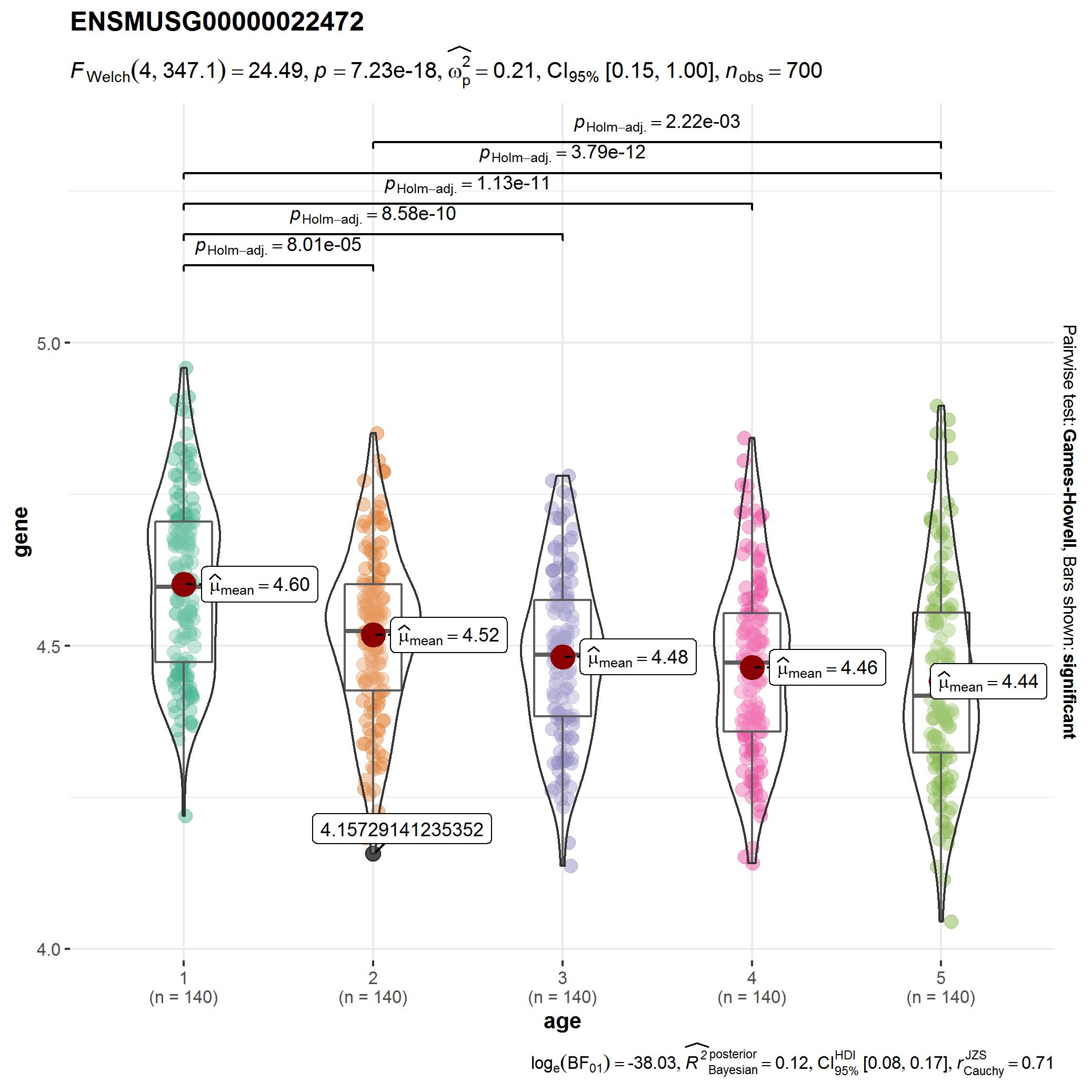

Supplement: Supplementary file 25 — Data S1–S6. [file ACEL-23-e14268-s017.zip › Data S1/ENSMUSG00000022472.jpeg]

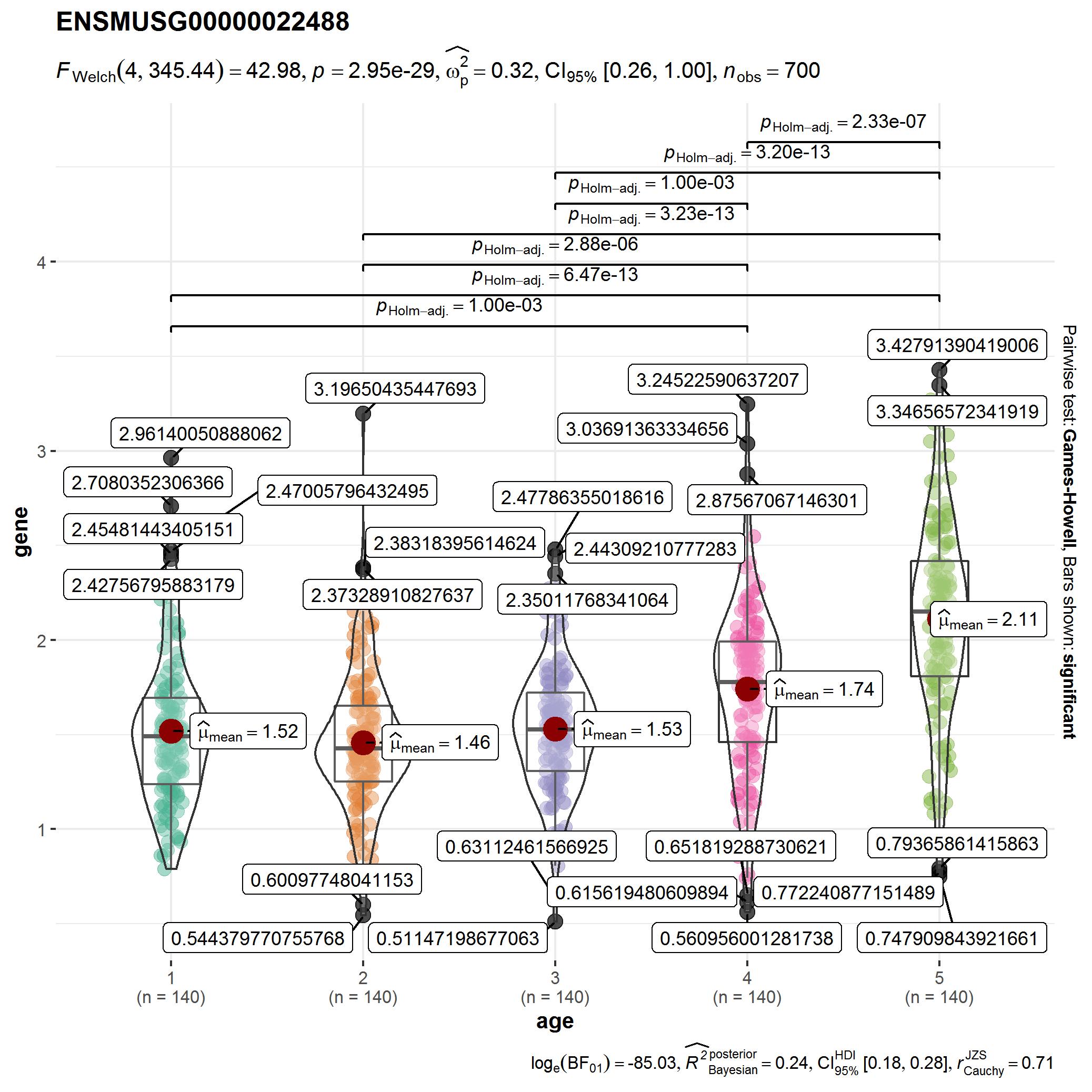

Supplement: Supplementary file 25 — Data S1–S6. [file ACEL-23-e14268-s017.zip › Data S1/ENSMUSG00000022488.jpeg]

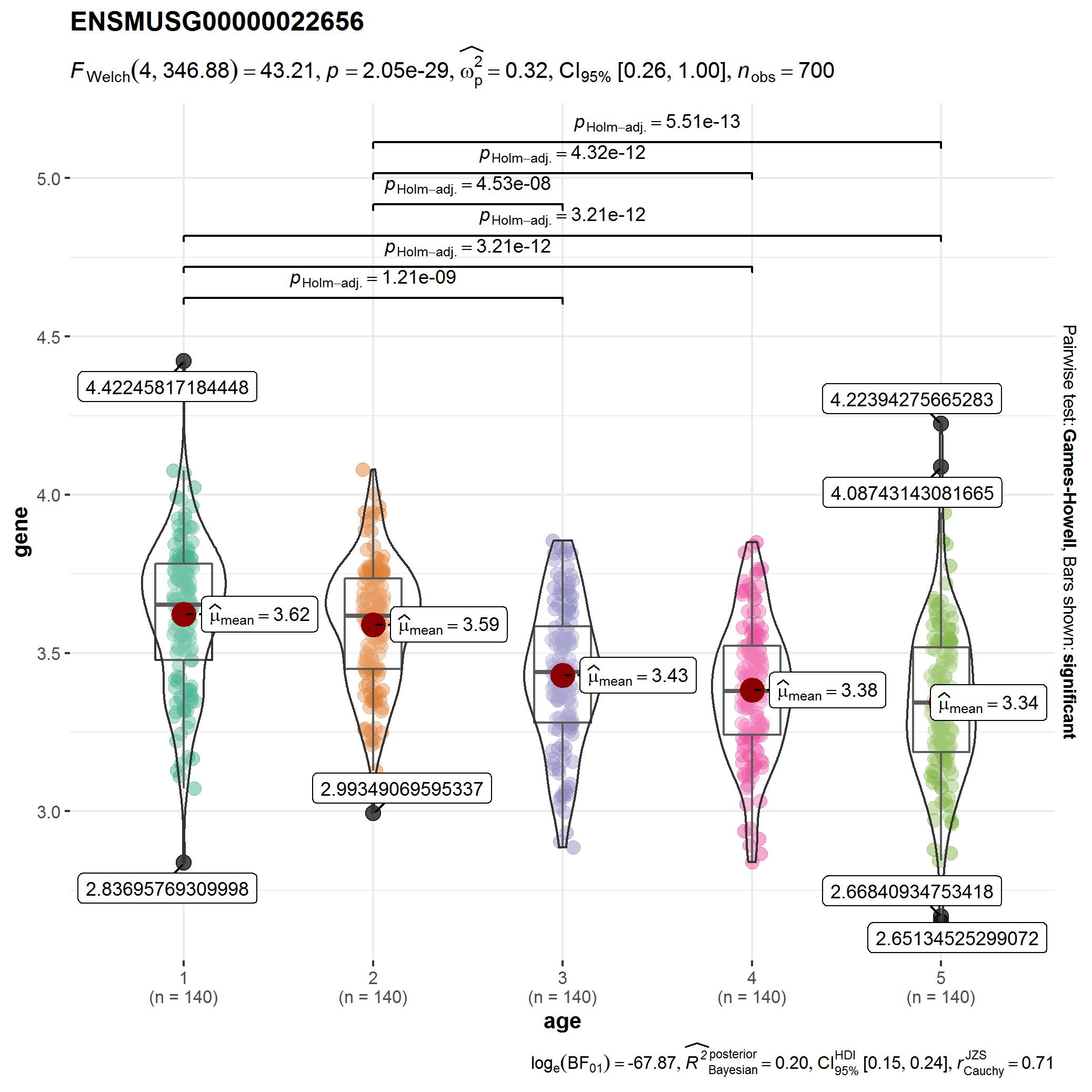

Supplement: Supplementary file 25 — Data S1–S6. [file ACEL-23-e14268-s017.zip › Data S1/ENSMUSG00000022656.jpeg]

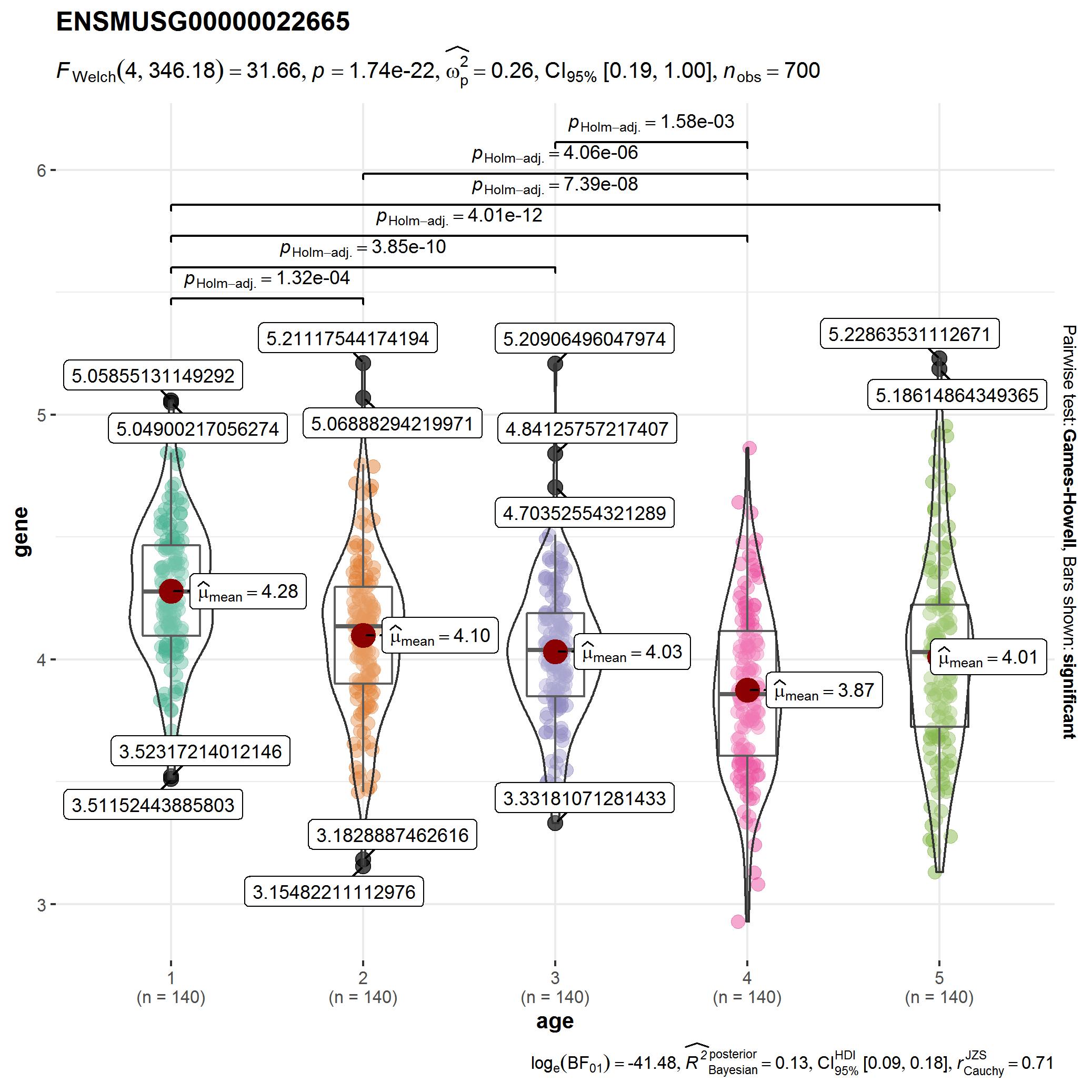

Supplement: Supplementary file 25 — Data S1–S6. [file ACEL-23-e14268-s017.zip › Data S1/ENSMUSG00000022665.jpeg]

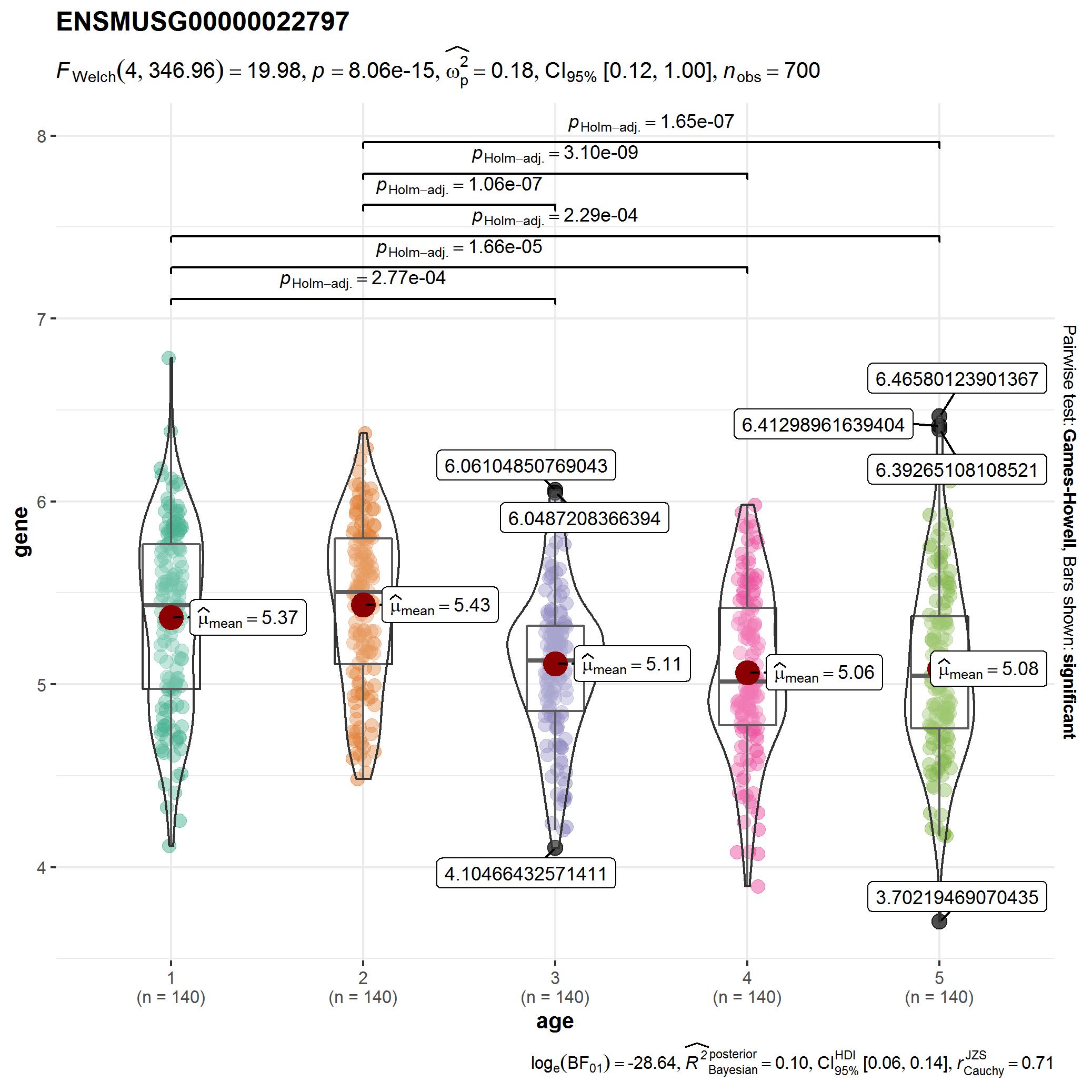

Supplement: Supplementary file 25 — Data S1–S6. [file ACEL-23-e14268-s017.zip › Data S1/ENSMUSG00000022797.jpeg]

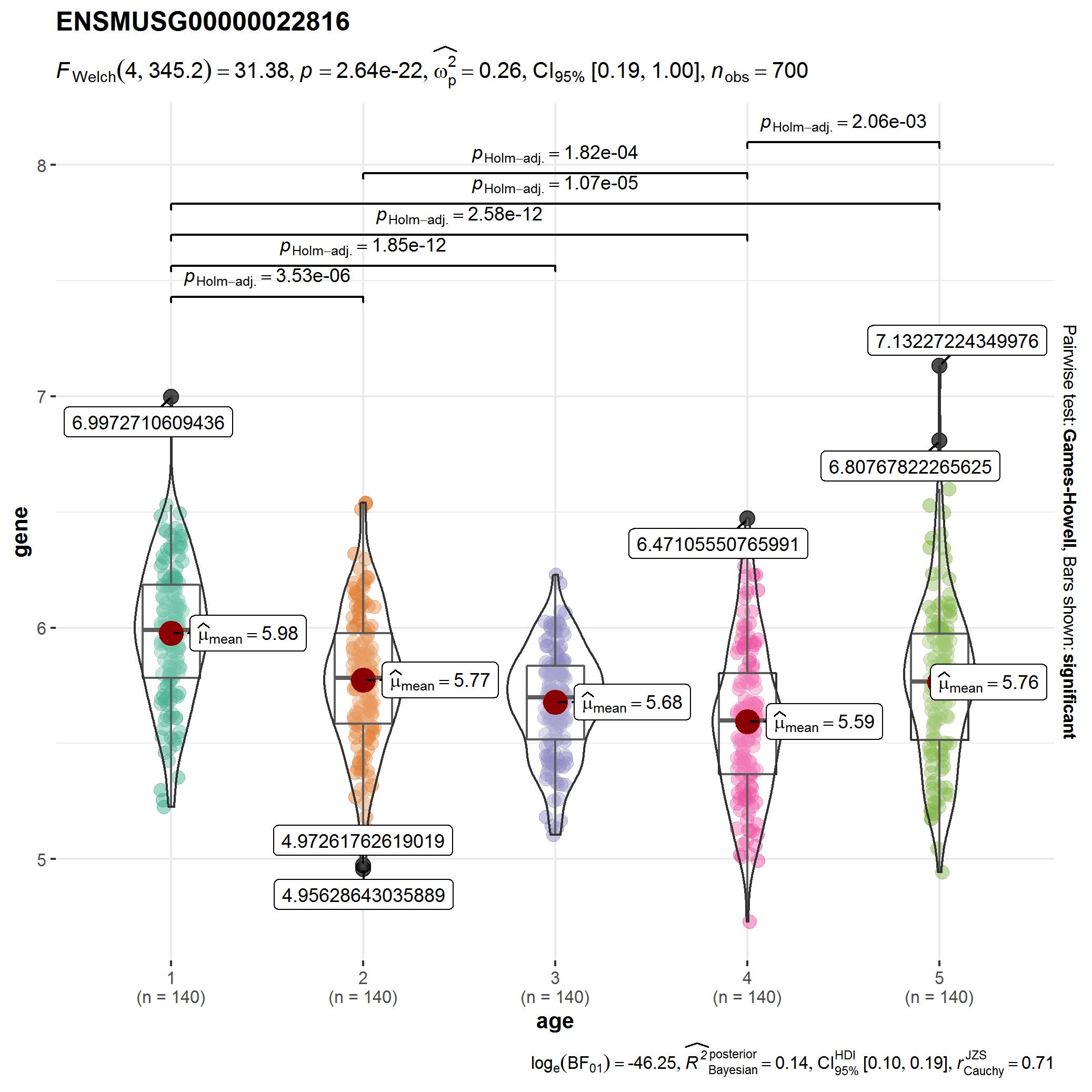

Supplement: Supplementary file 25 — Data S1–S6. [file ACEL-23-e14268-s017.zip › Data S1/ENSMUSG00000022816.jpeg]

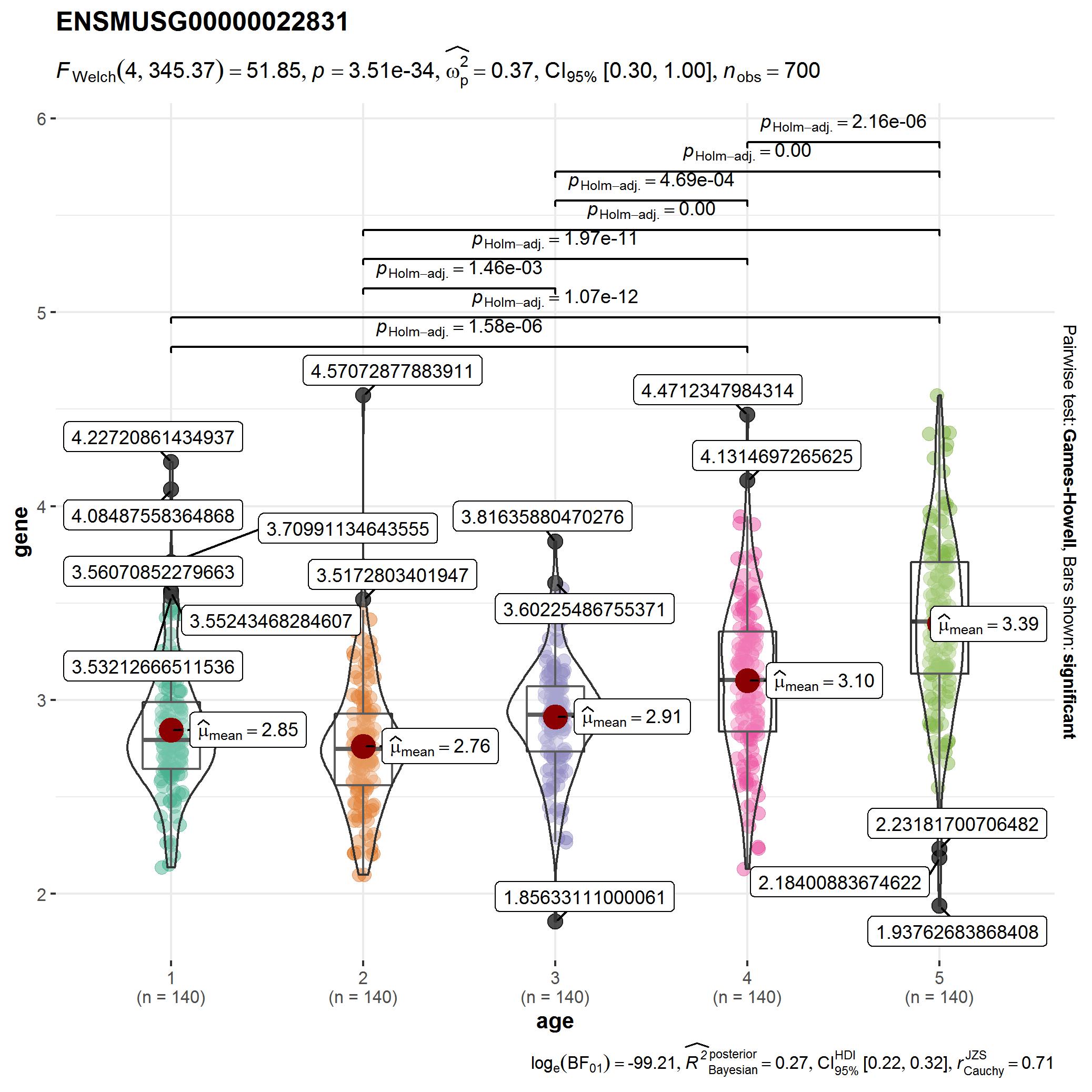

Supplement: Supplementary file 25 — Data S1–S6. [file ACEL-23-e14268-s017.zip › Data S1/ENSMUSG00000022831.jpeg]

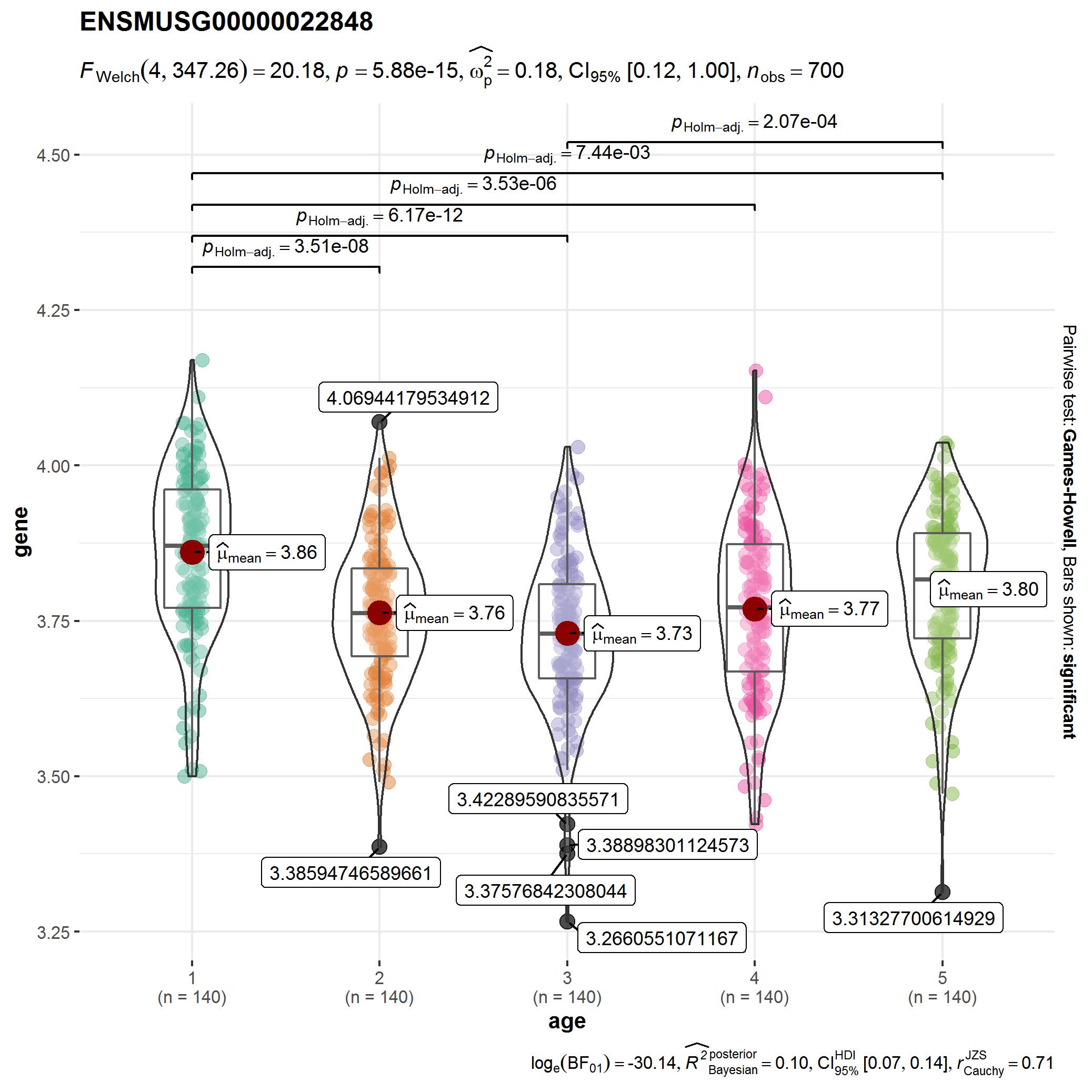

Supplement: Supplementary file 25 — Data S1–S6. [file ACEL-23-e14268-s017.zip › Data S1/ENSMUSG00000022848.jpeg]

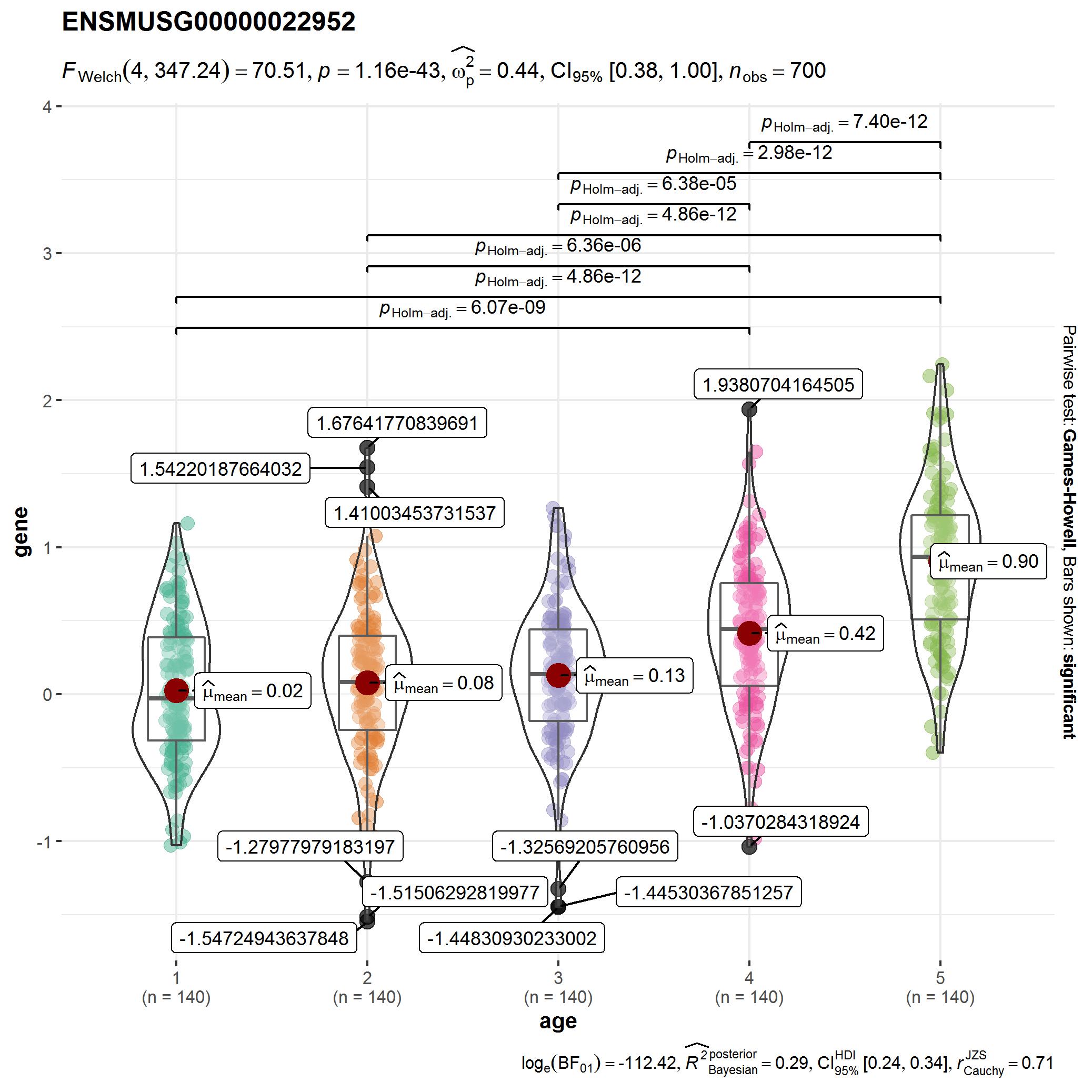

Supplement: Supplementary file 25 — Data S1–S6. [file ACEL-23-e14268-s017.zip › Data S1/ENSMUSG00000022952.jpeg]

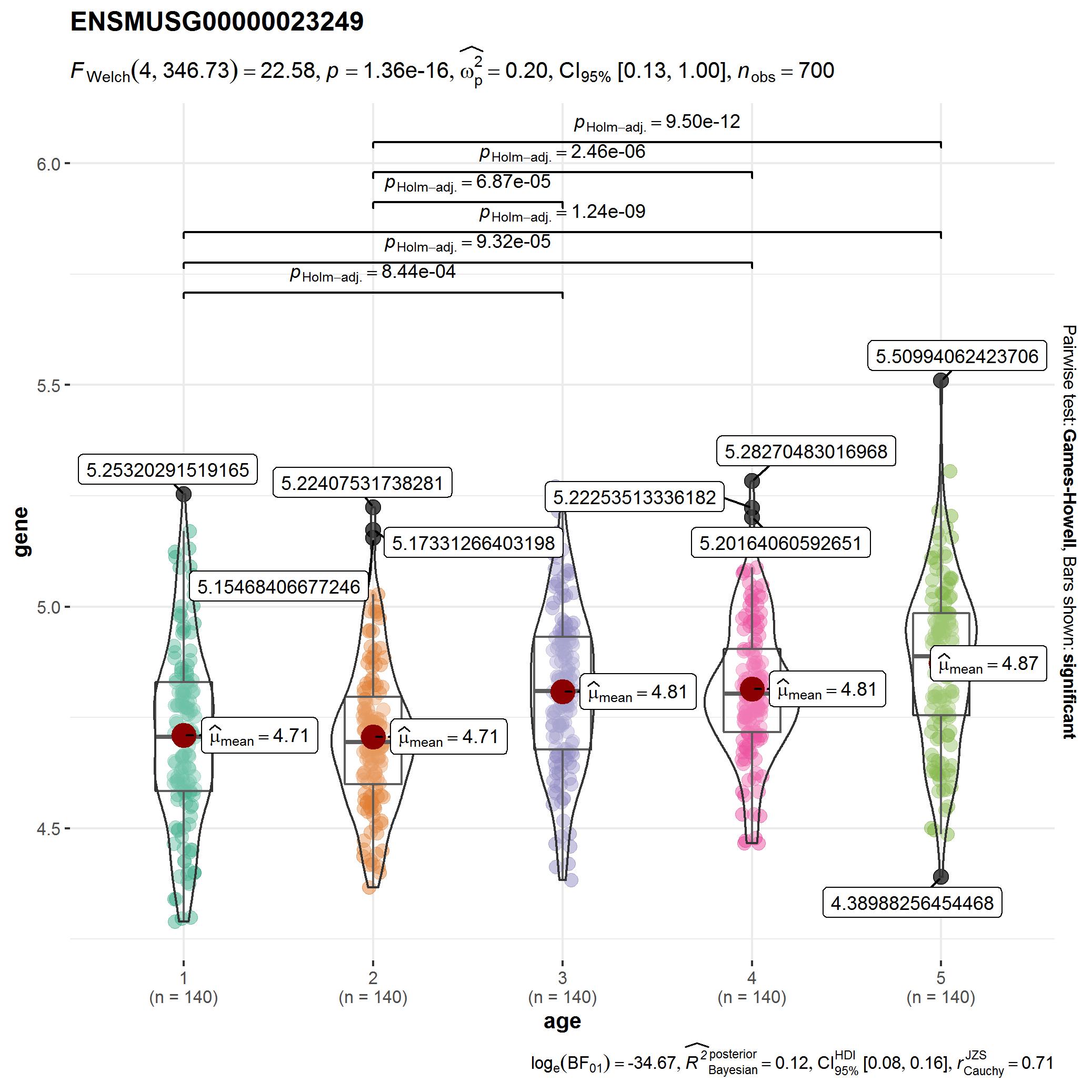

Supplement: Supplementary file 25 — Data S1–S6. [file ACEL-23-e14268-s017.zip › Data S1/ENSMUSG00000023249.jpeg]

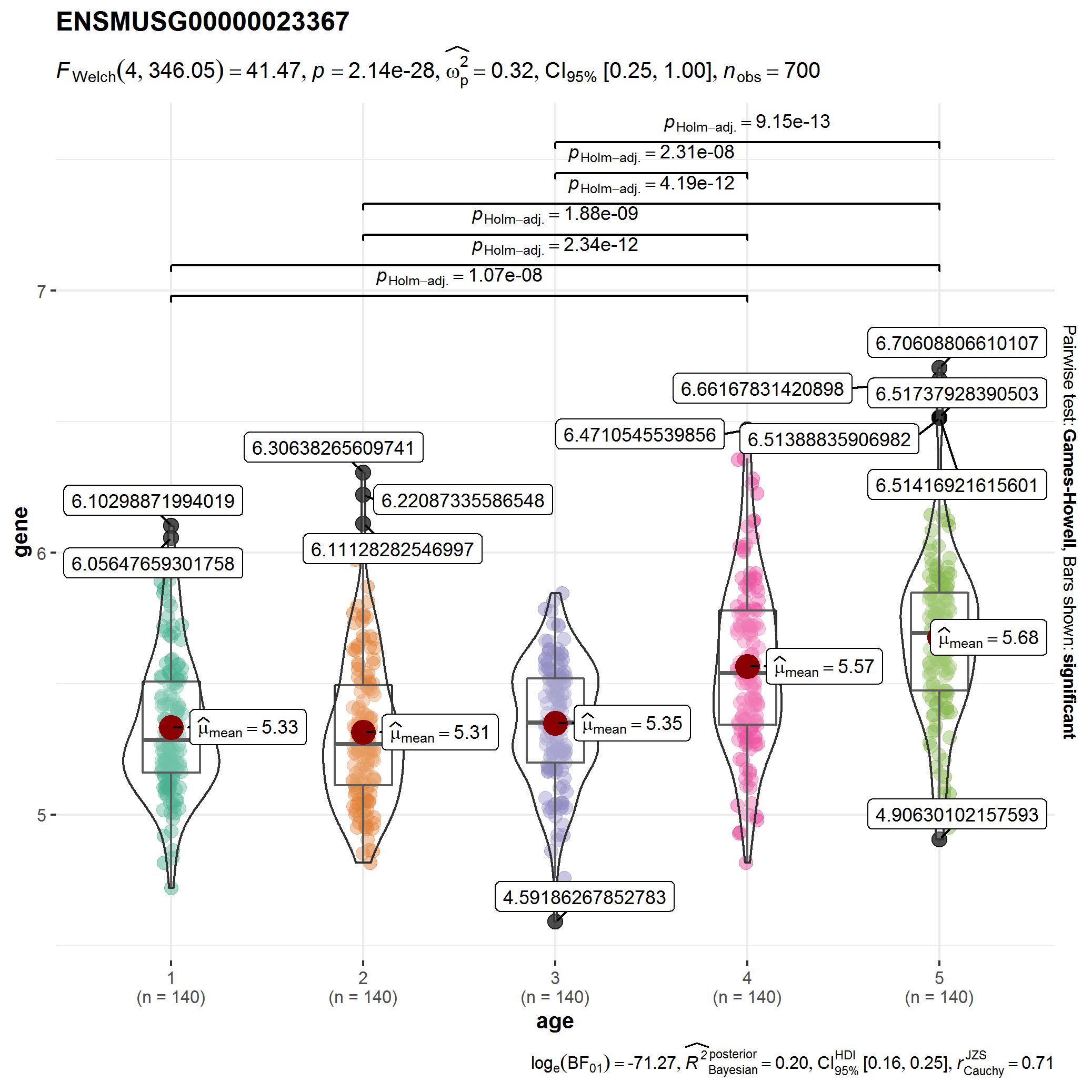

Supplement: Supplementary file 25 — Data S1–S6. [file ACEL-23-e14268-s017.zip › Data S1/ENSMUSG00000023367.jpeg]

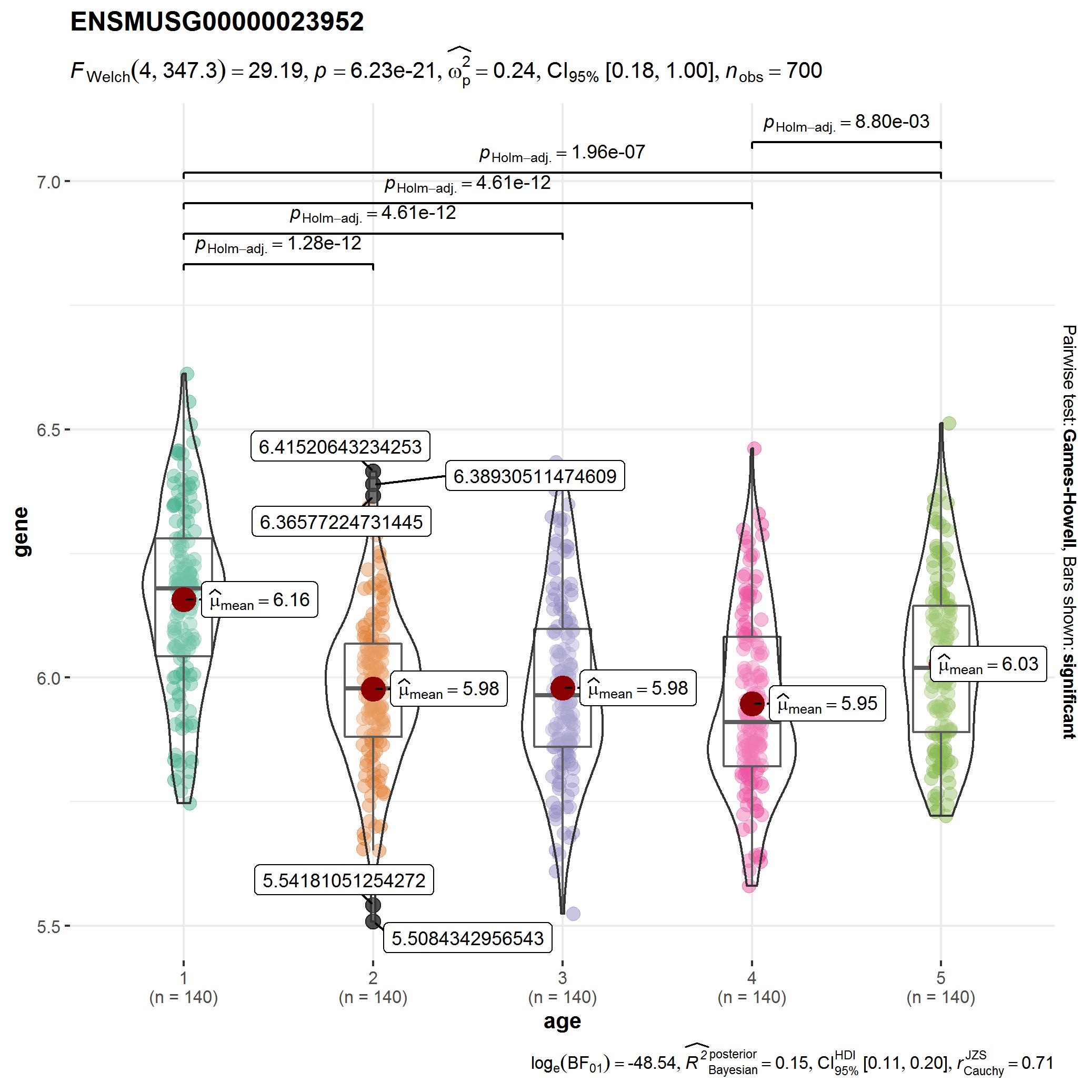

Supplement: Supplementary file 25 — Data S1–S6. [file ACEL-23-e14268-s017.zip › Data S1/ENSMUSG00000023952.jpeg]

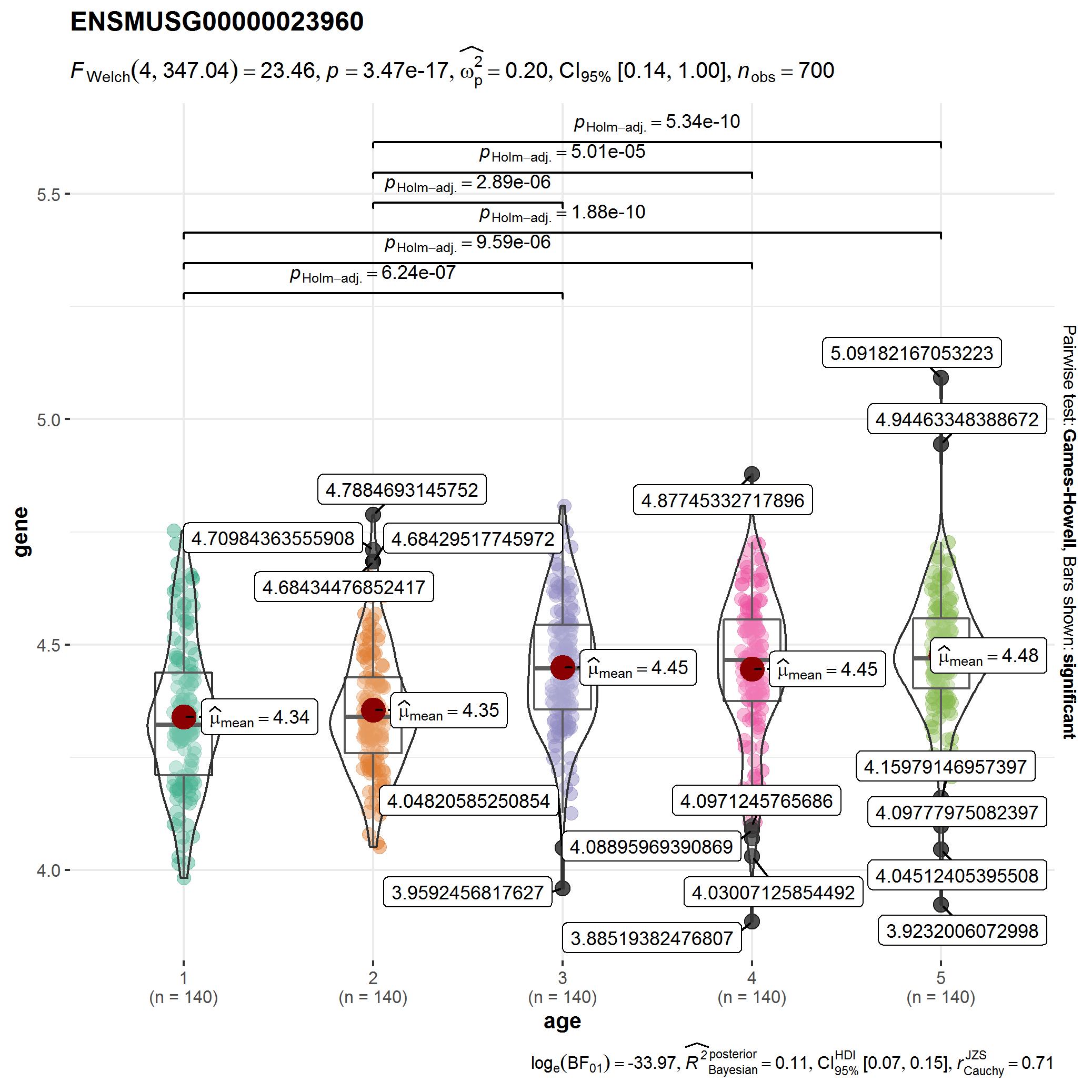

Supplement: Supplementary file 25 — Data S1–S6. [file ACEL-23-e14268-s017.zip › Data S1/ENSMUSG00000023960.jpeg]

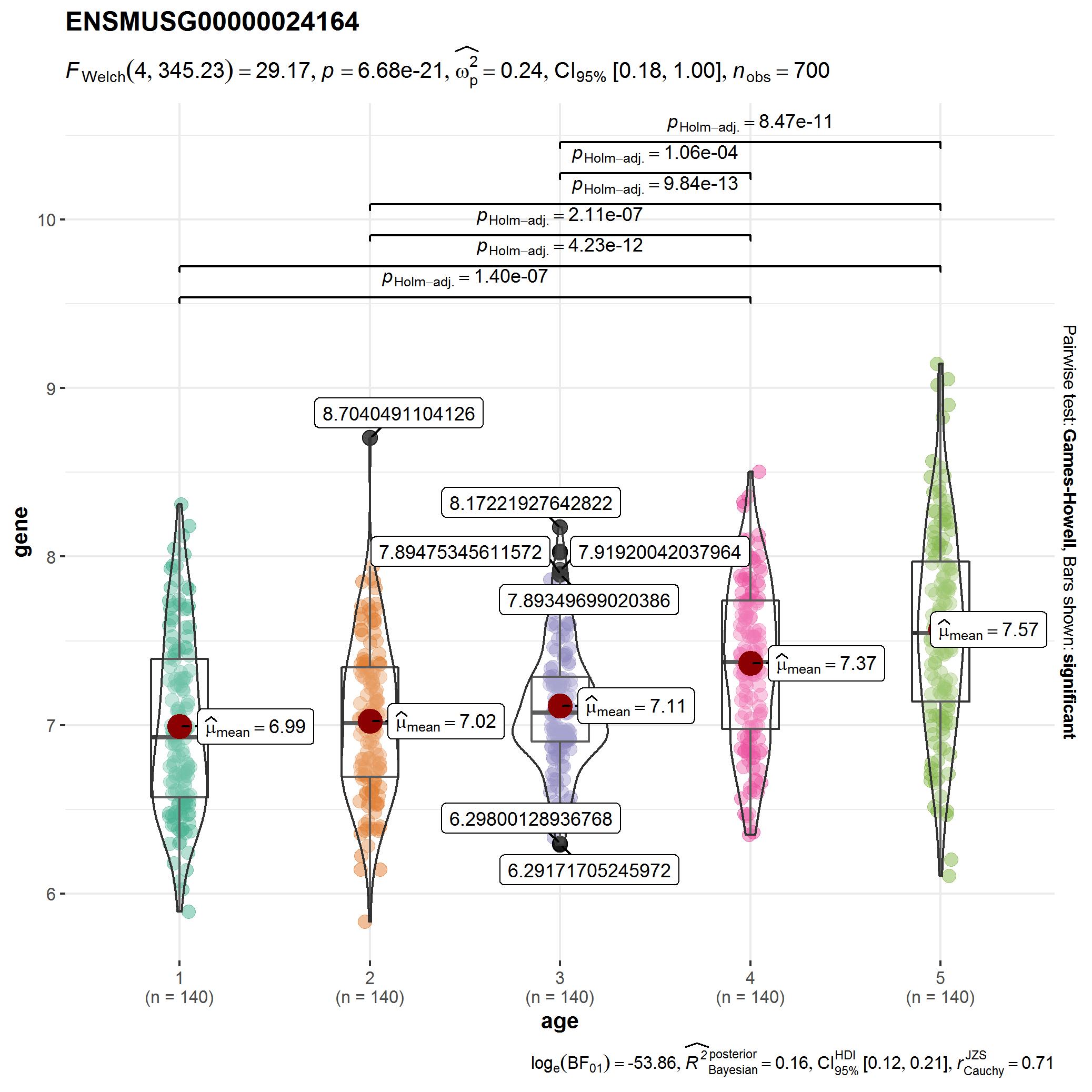

Supplement: Supplementary file 25 — Data S1–S6. [file ACEL-23-e14268-s017.zip › Data S1/ENSMUSG00000024164.jpeg]

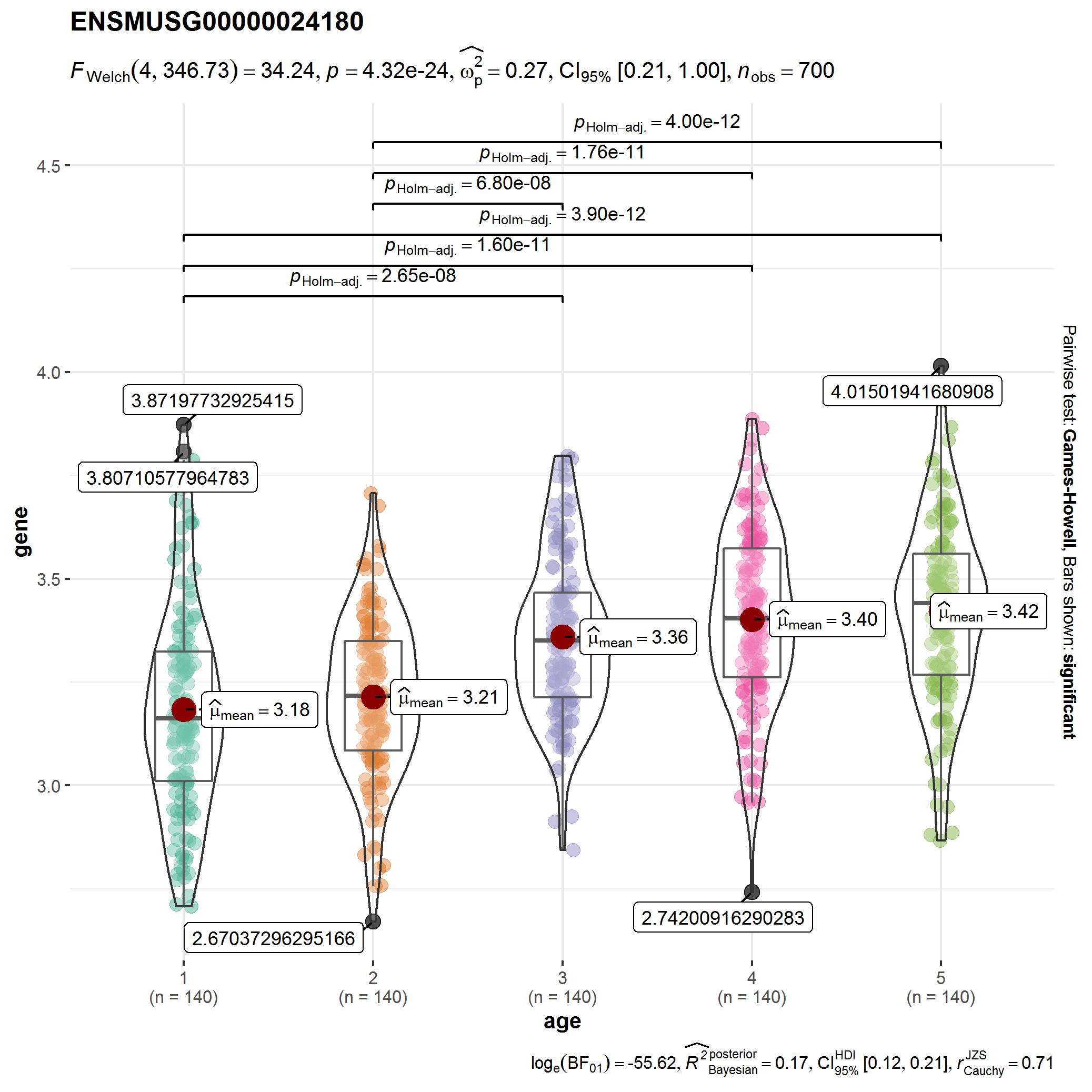

Supplement: Supplementary file 25 — Data S1–S6. [file ACEL-23-e14268-s017.zip › Data S1/ENSMUSG00000024180.jpeg]

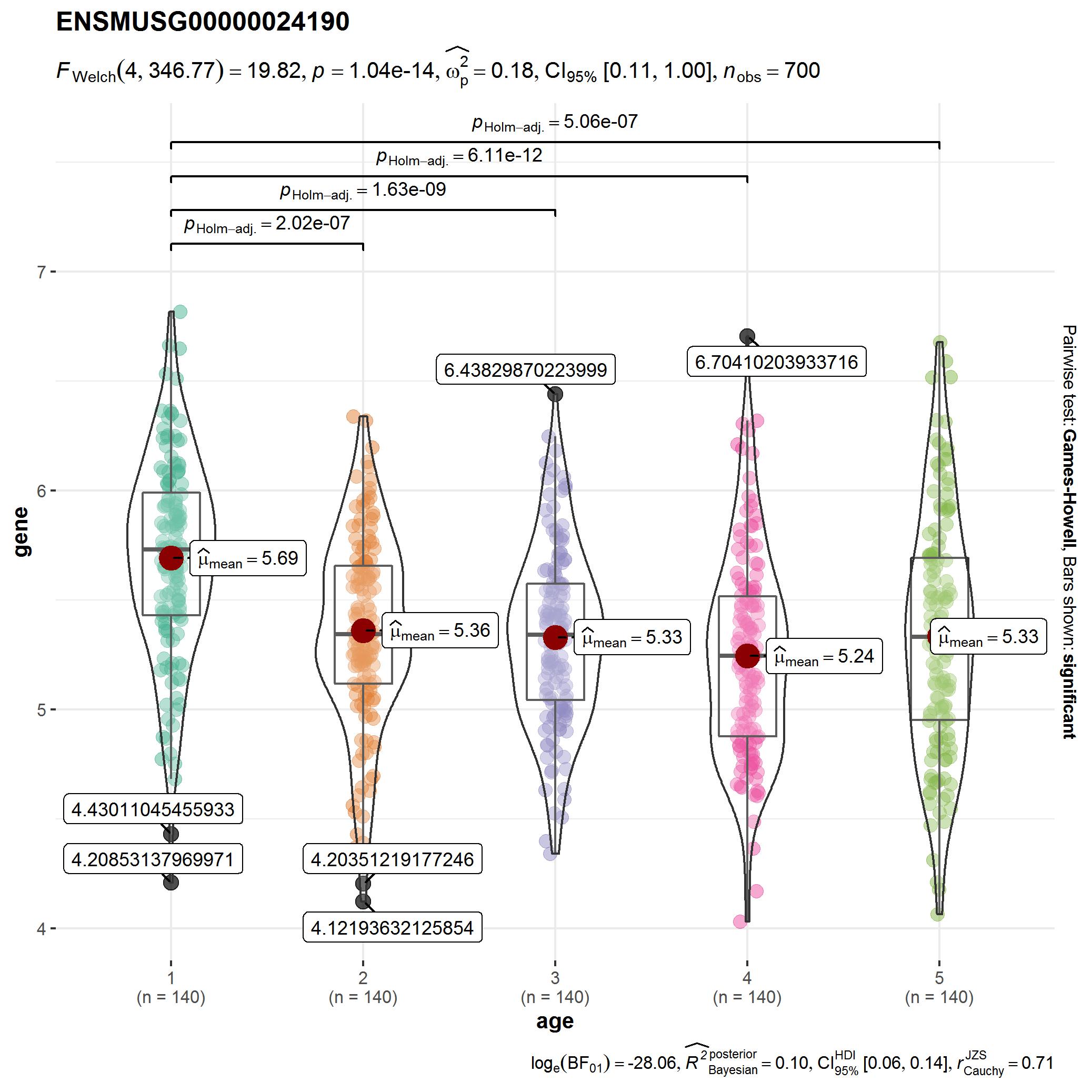

Supplement: Supplementary file 25 — Data S1–S6. [file ACEL-23-e14268-s017.zip › Data S1/ENSMUSG00000024190.jpeg]

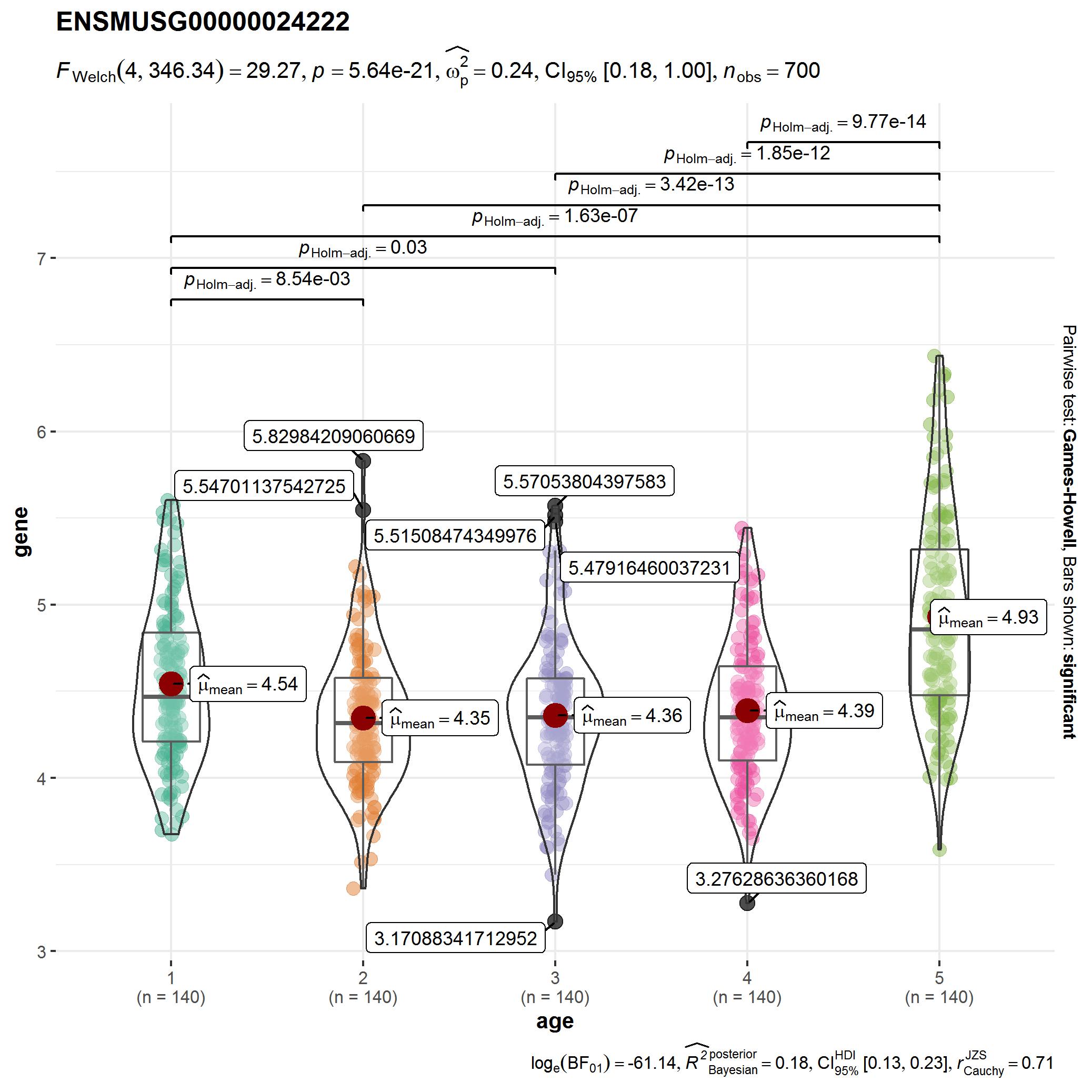

Supplement: Supplementary file 25 — Data S1–S6. [file ACEL-23-e14268-s017.zip › Data S1/ENSMUSG00000024222.jpeg]

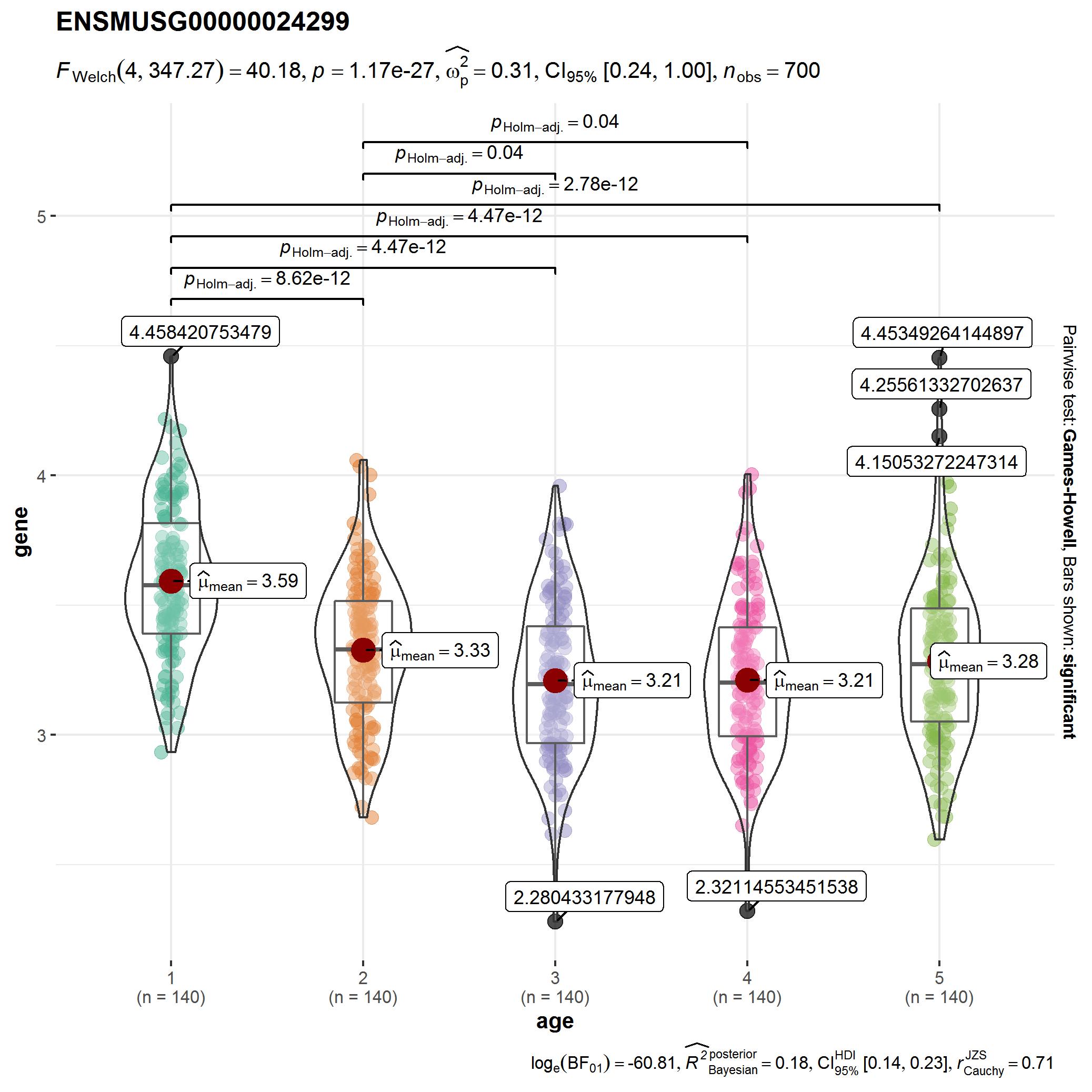

Supplement: Supplementary file 25 — Data S1–S6. [file ACEL-23-e14268-s017.zip › Data S1/ENSMUSG00000024299.jpeg]

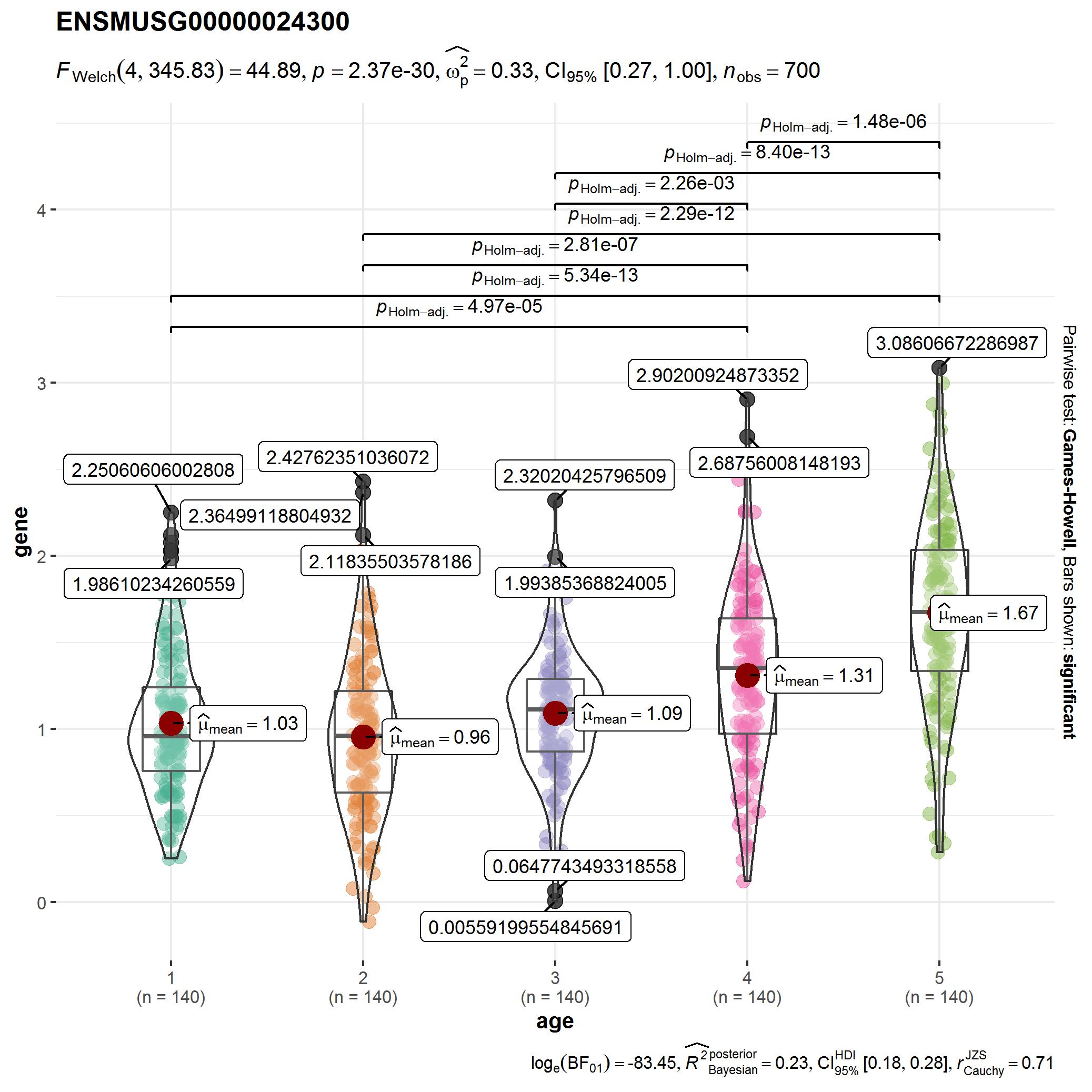

Supplement: Supplementary file 25 — Data S1–S6. [file ACEL-23-e14268-s017.zip › Data S1/ENSMUSG00000024300.jpeg]

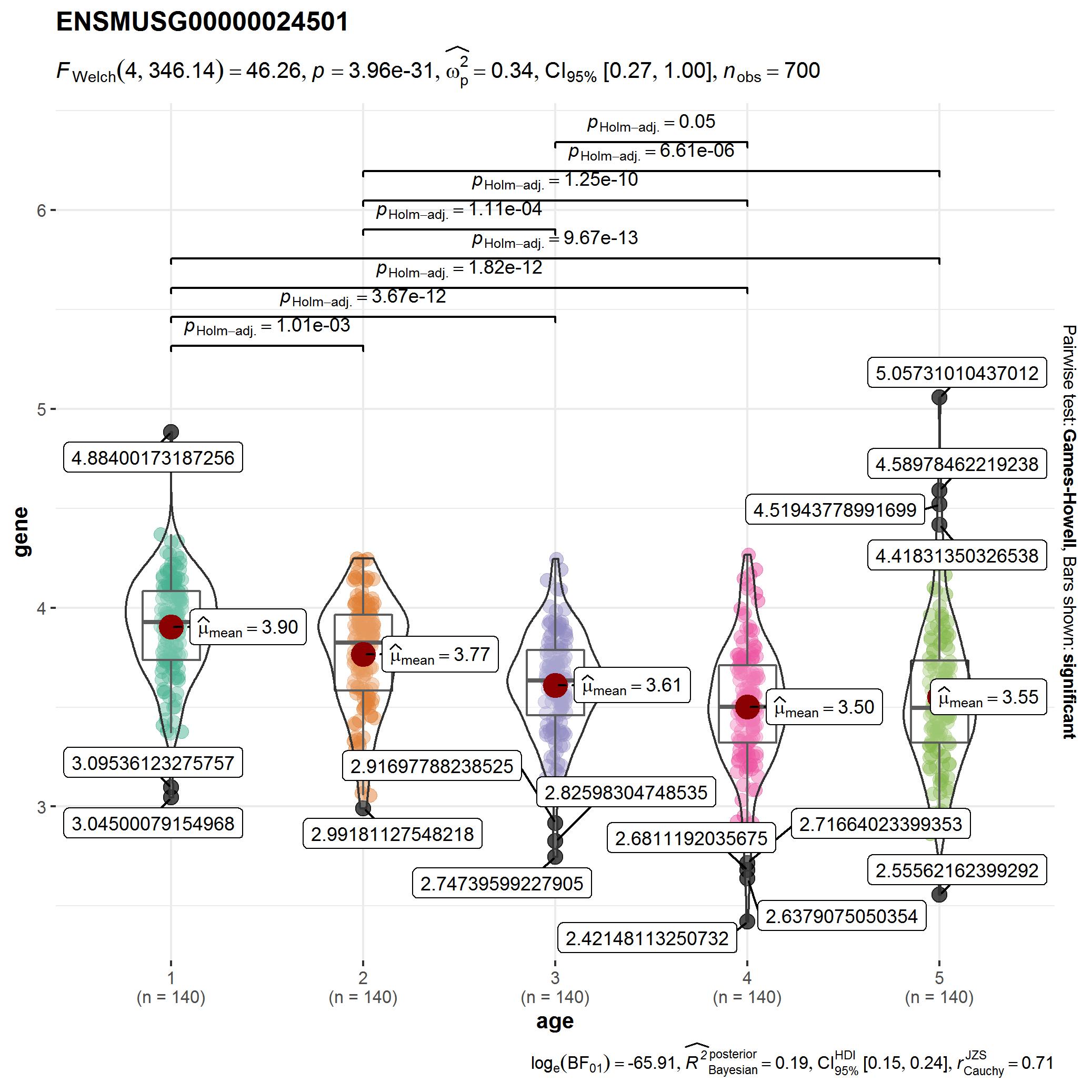

Supplement: Supplementary file 25 — Data S1–S6. [file ACEL-23-e14268-s017.zip › Data S1/ENSMUSG00000024501.jpeg]

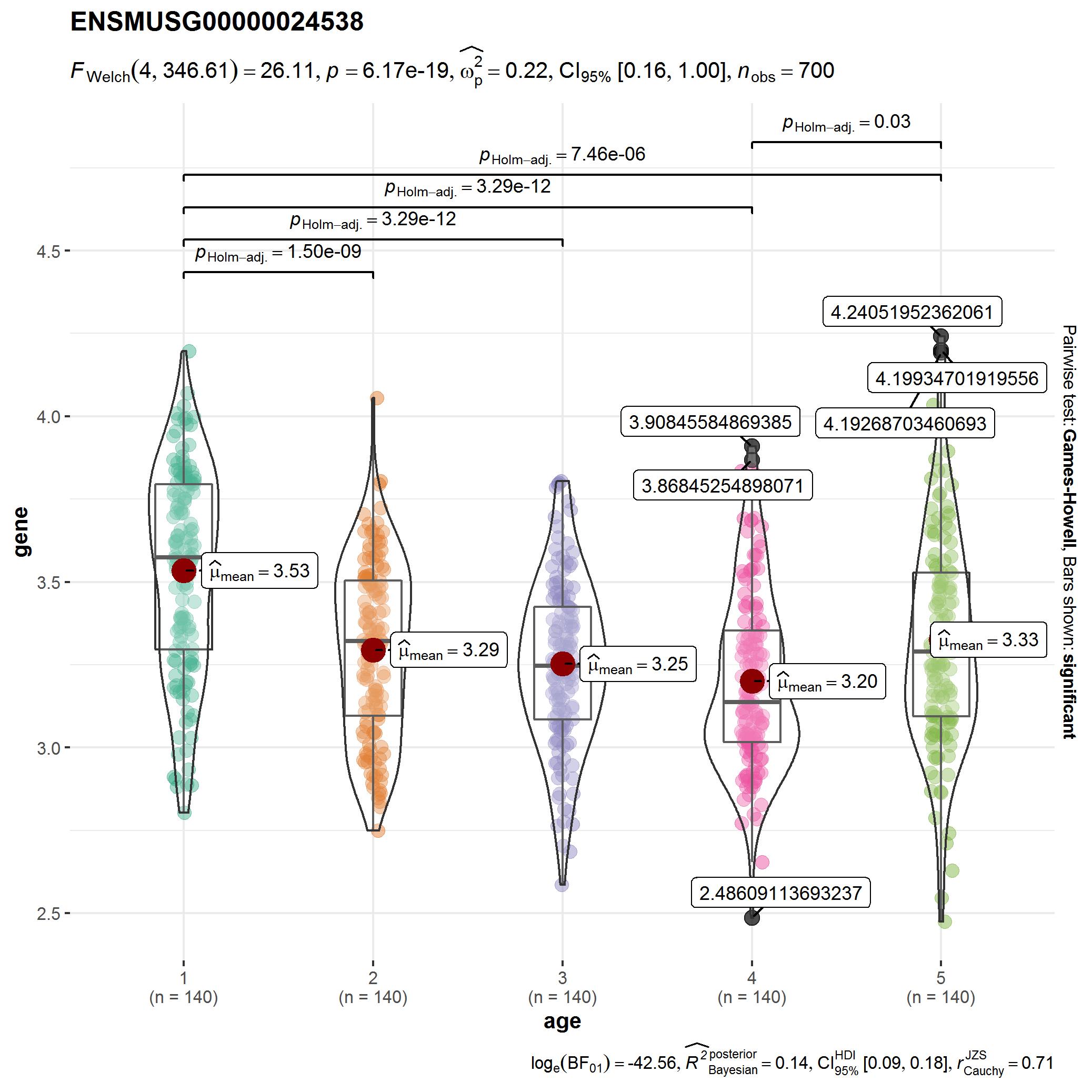

Supplement: Supplementary file 25 — Data S1–S6. [file ACEL-23-e14268-s017.zip › Data S1/ENSMUSG00000024538.jpeg]

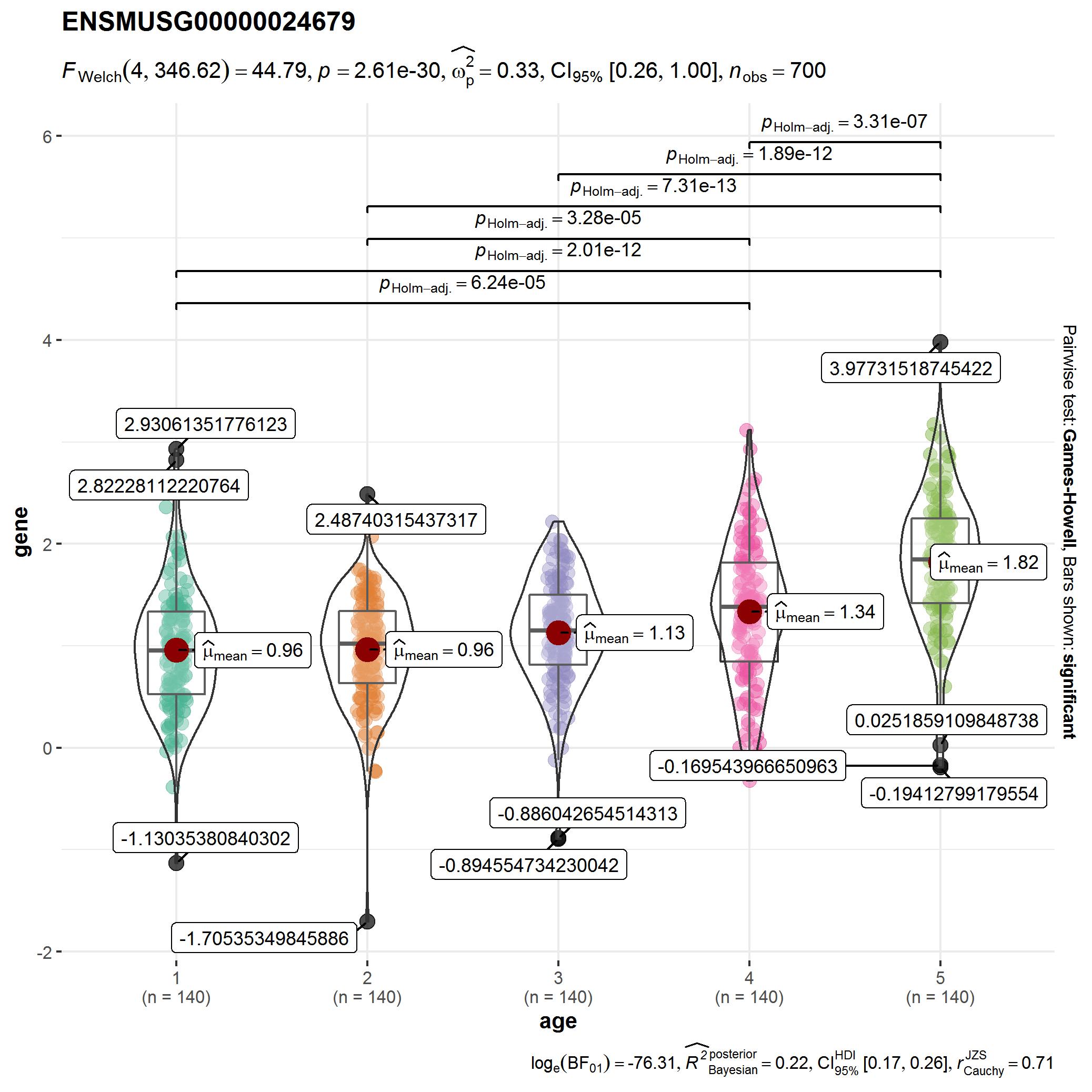

Supplement: Supplementary file 25 — Data S1–S6. [file ACEL-23-e14268-s017.zip › Data S1/ENSMUSG00000024679.jpeg]

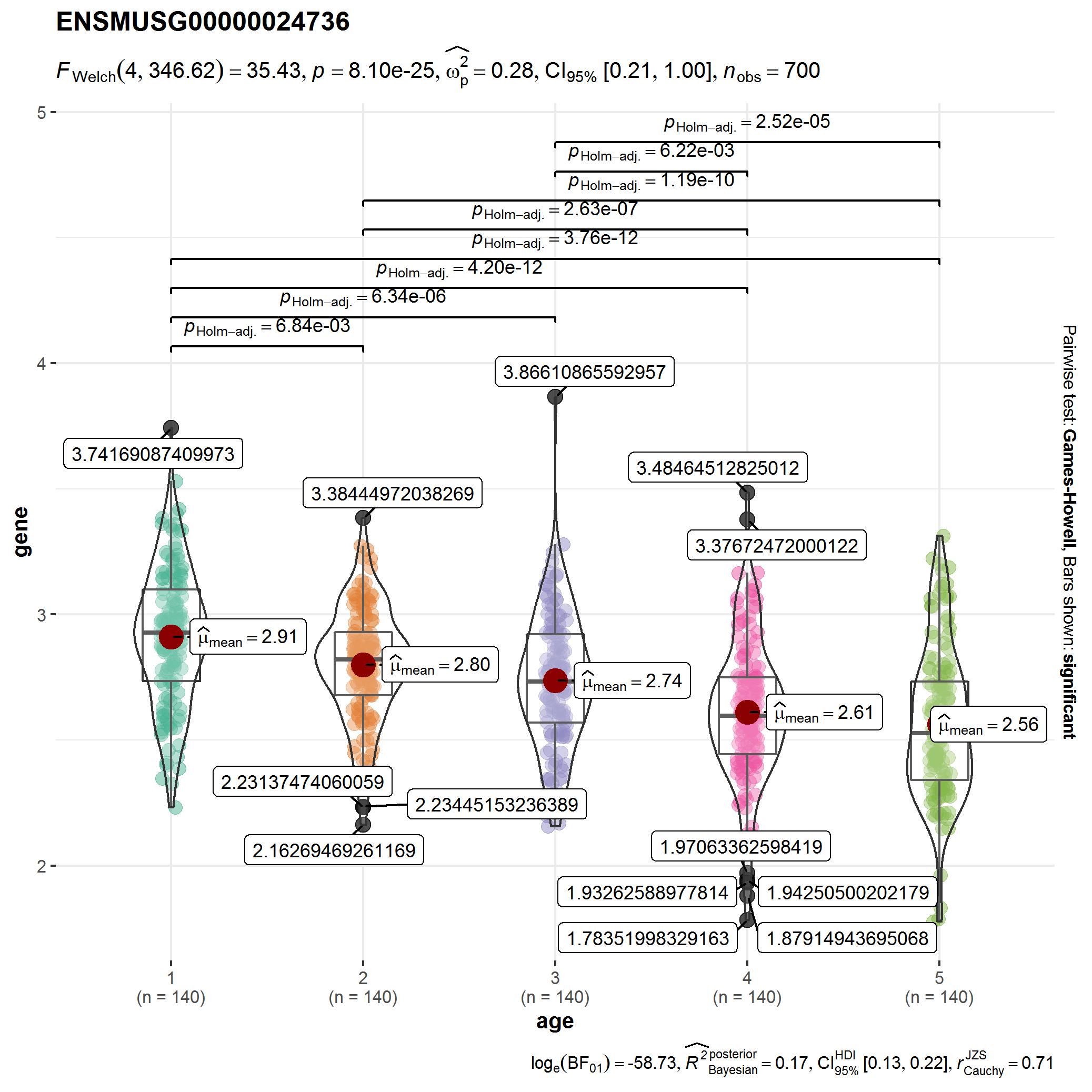

Supplement: Supplementary file 25 — Data S1–S6. [file ACEL-23-e14268-s017.zip › Data S1/ENSMUSG00000024736.jpeg]

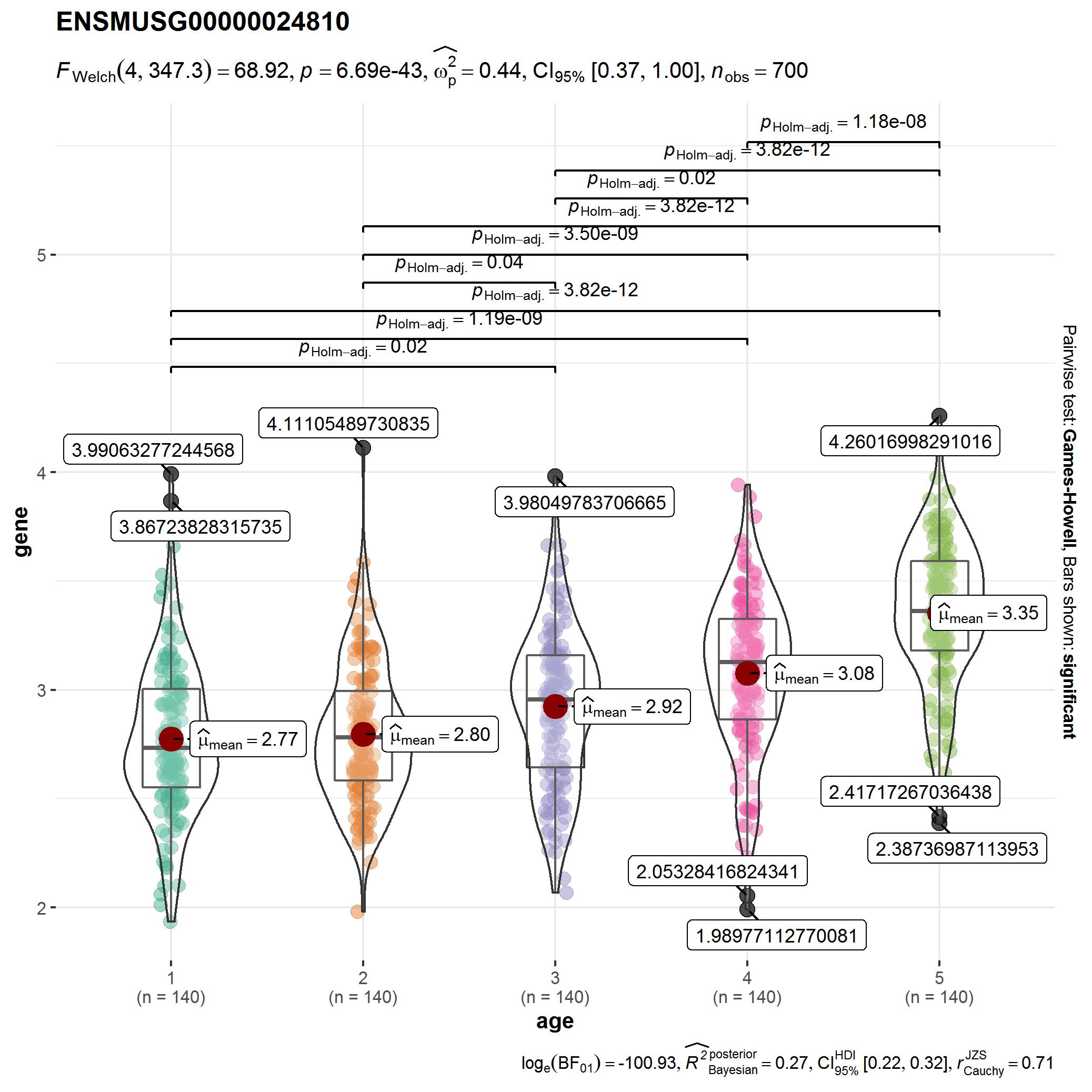

Supplement: Supplementary file 25 — Data S1–S6. [file ACEL-23-e14268-s017.zip › Data S1/ENSMUSG00000024810.jpeg]

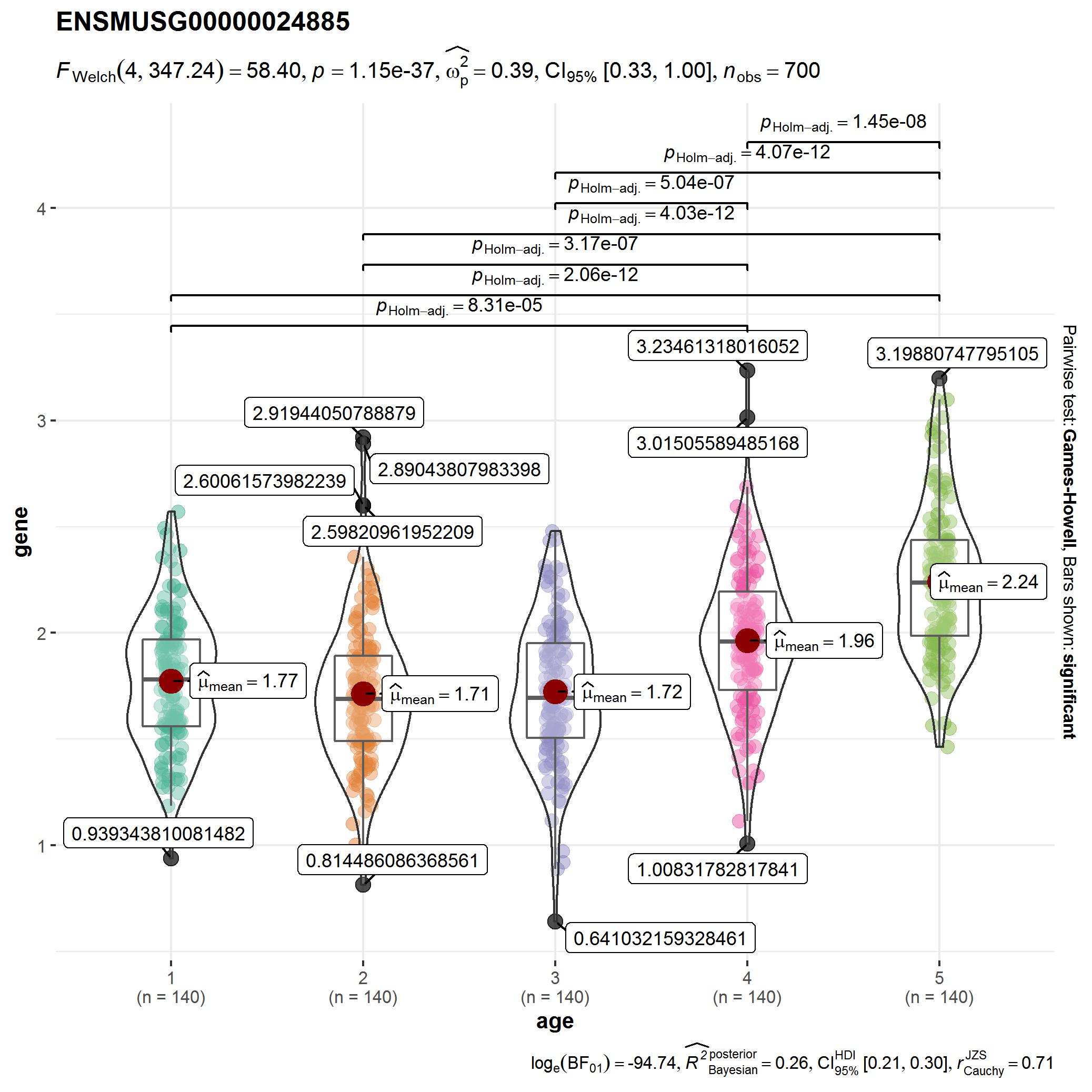

Supplement: Supplementary file 25 — Data S1–S6. [file ACEL-23-e14268-s017.zip › Data S1/ENSMUSG00000024885.jpeg]

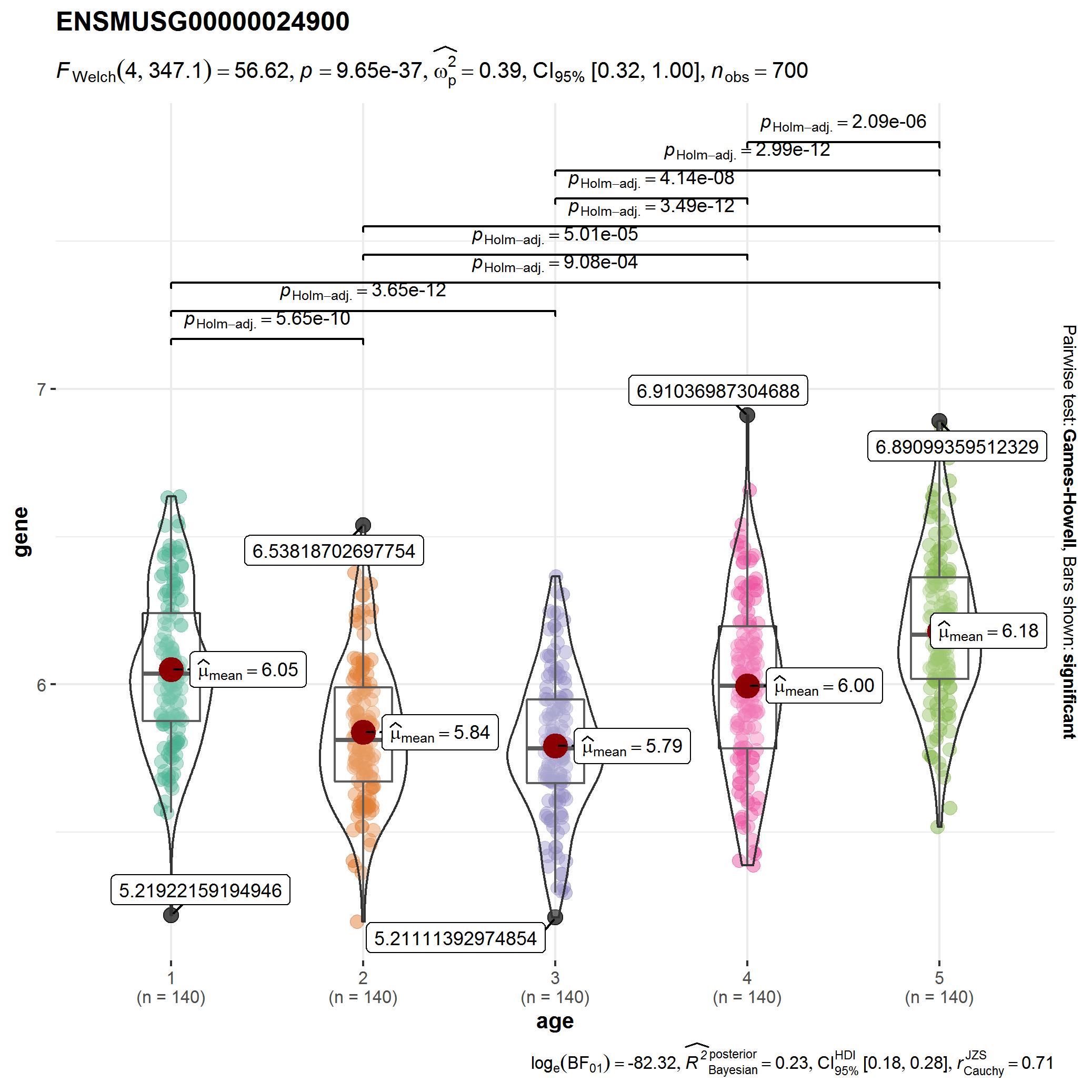

Supplement: Supplementary file 25 — Data S1–S6. [file ACEL-23-e14268-s017.zip › Data S1/ENSMUSG00000024900.jpeg]

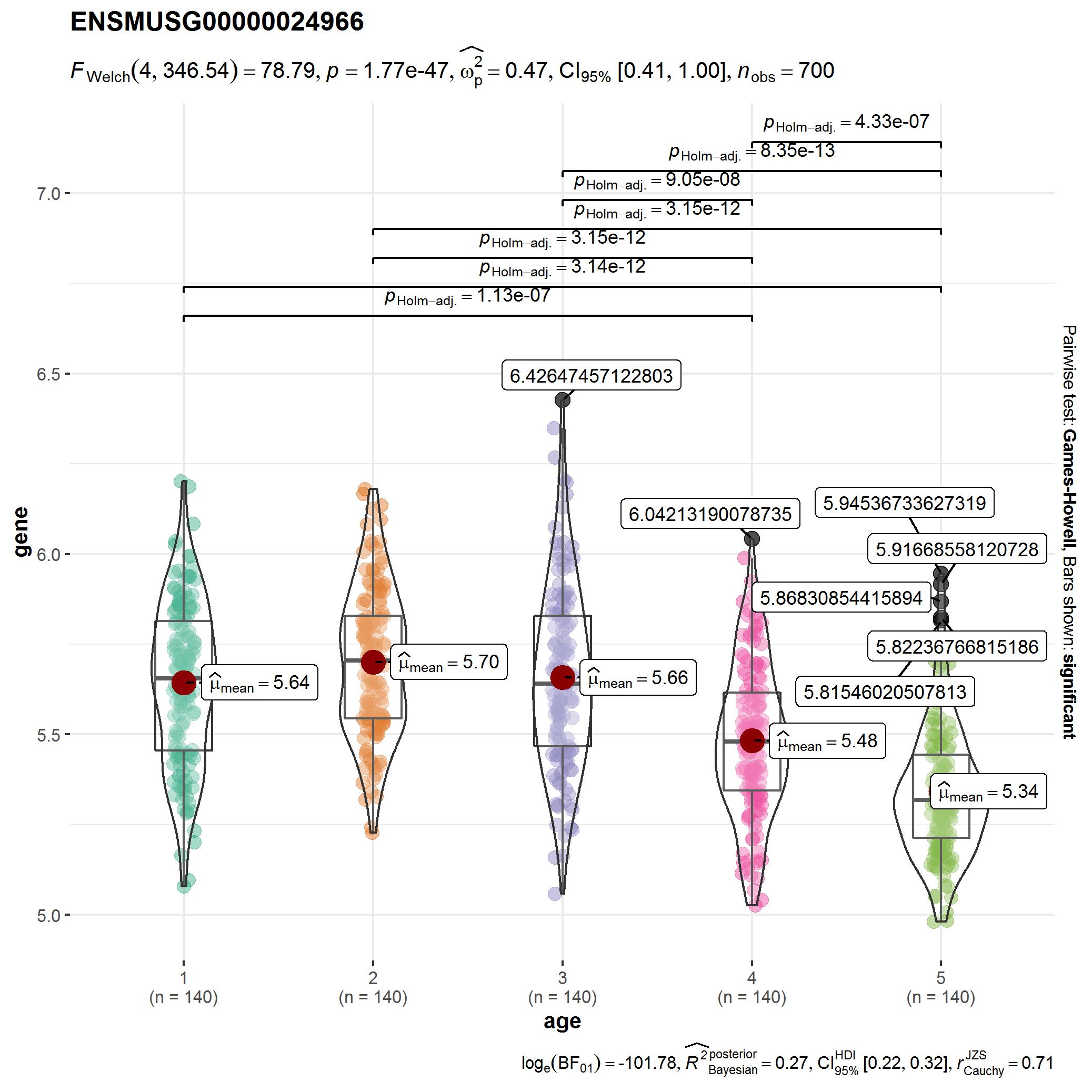

Supplement: Supplementary file 25 — Data S1–S6. [file ACEL-23-e14268-s017.zip › Data S1/ENSMUSG00000024966.jpeg]

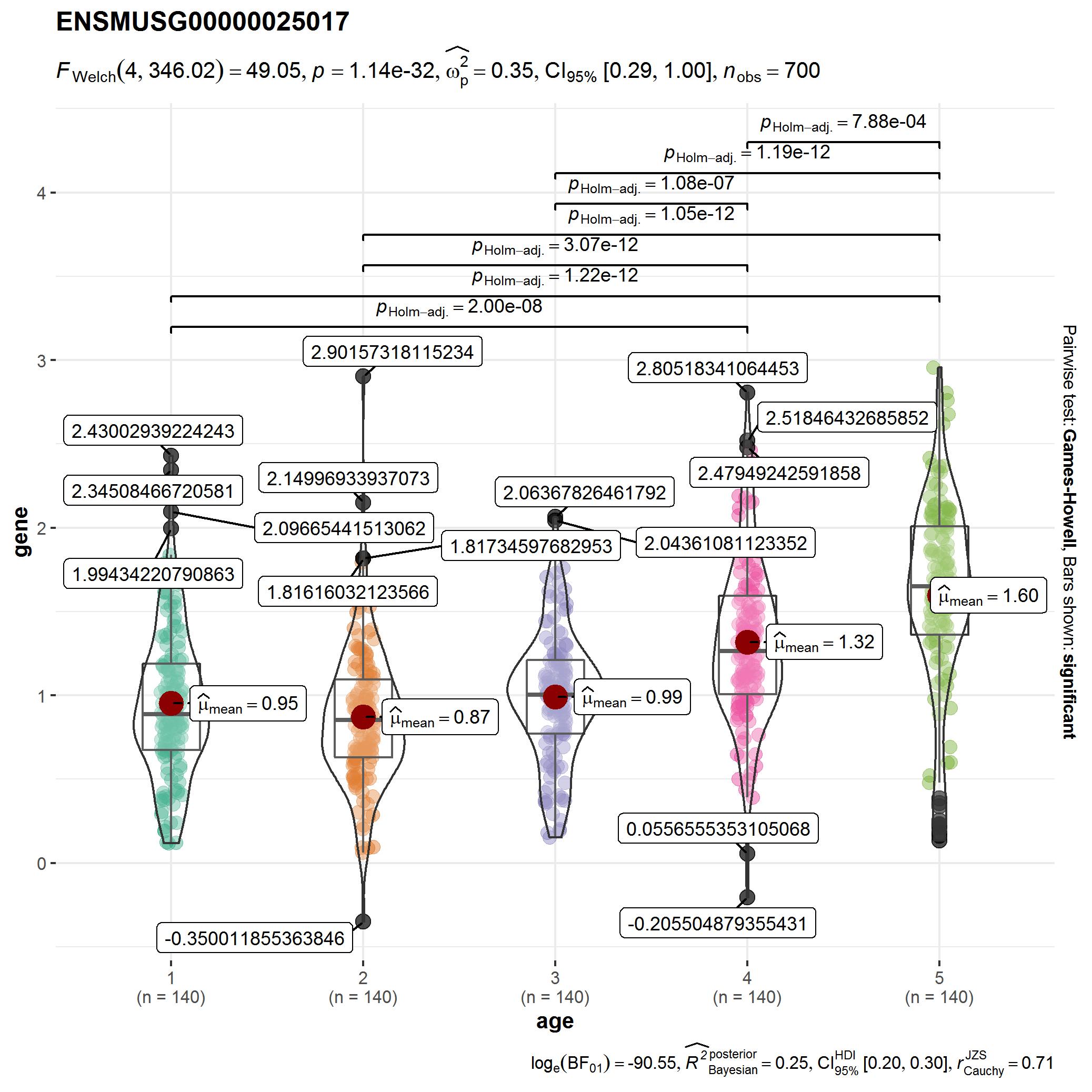

Supplement: Supplementary file 25 — Data S1–S6. [file ACEL-23-e14268-s017.zip › Data S1/ENSMUSG00000025017.jpeg]

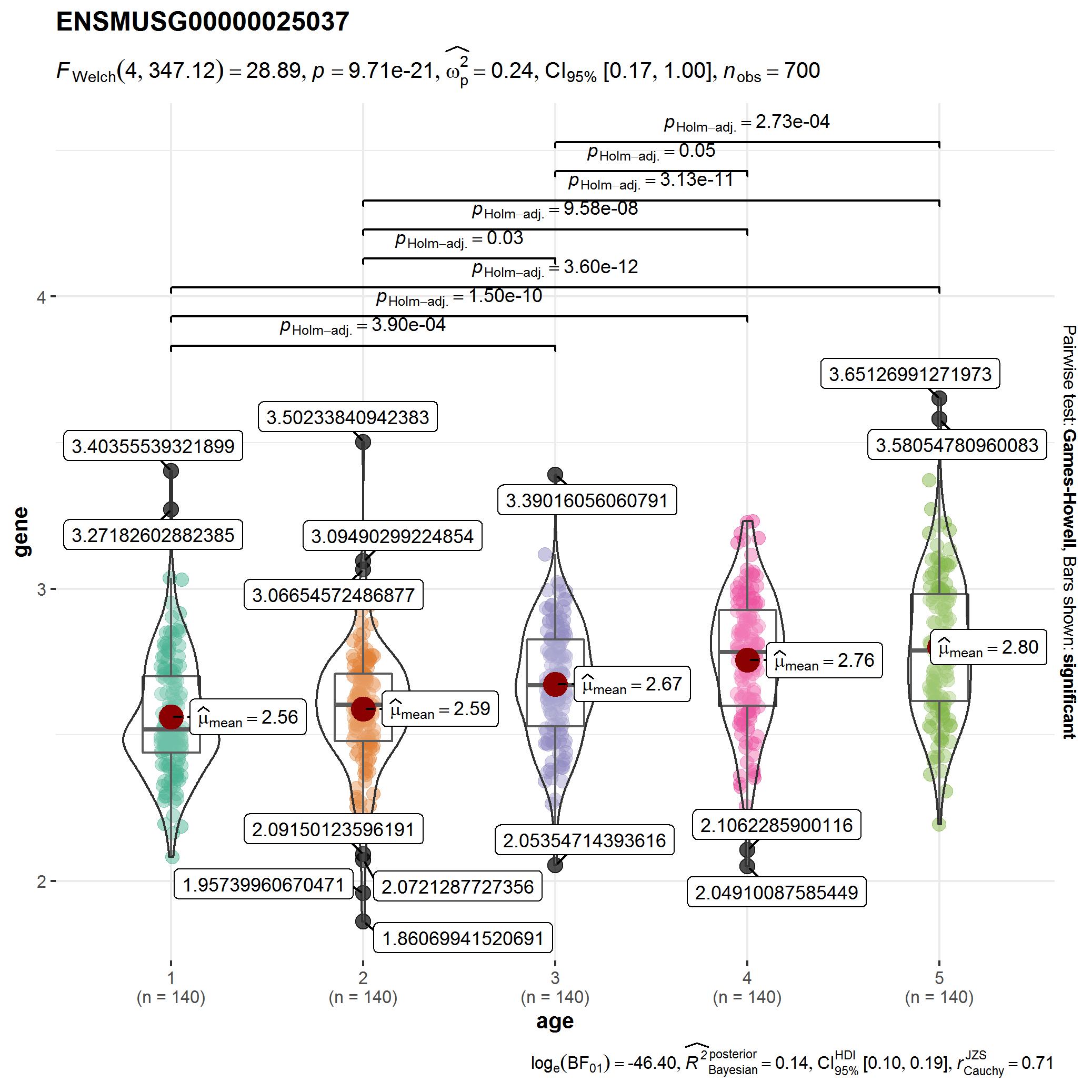

Supplement: Supplementary file 25 — Data S1–S6. [file ACEL-23-e14268-s017.zip › Data S1/ENSMUSG00000025037.jpeg]

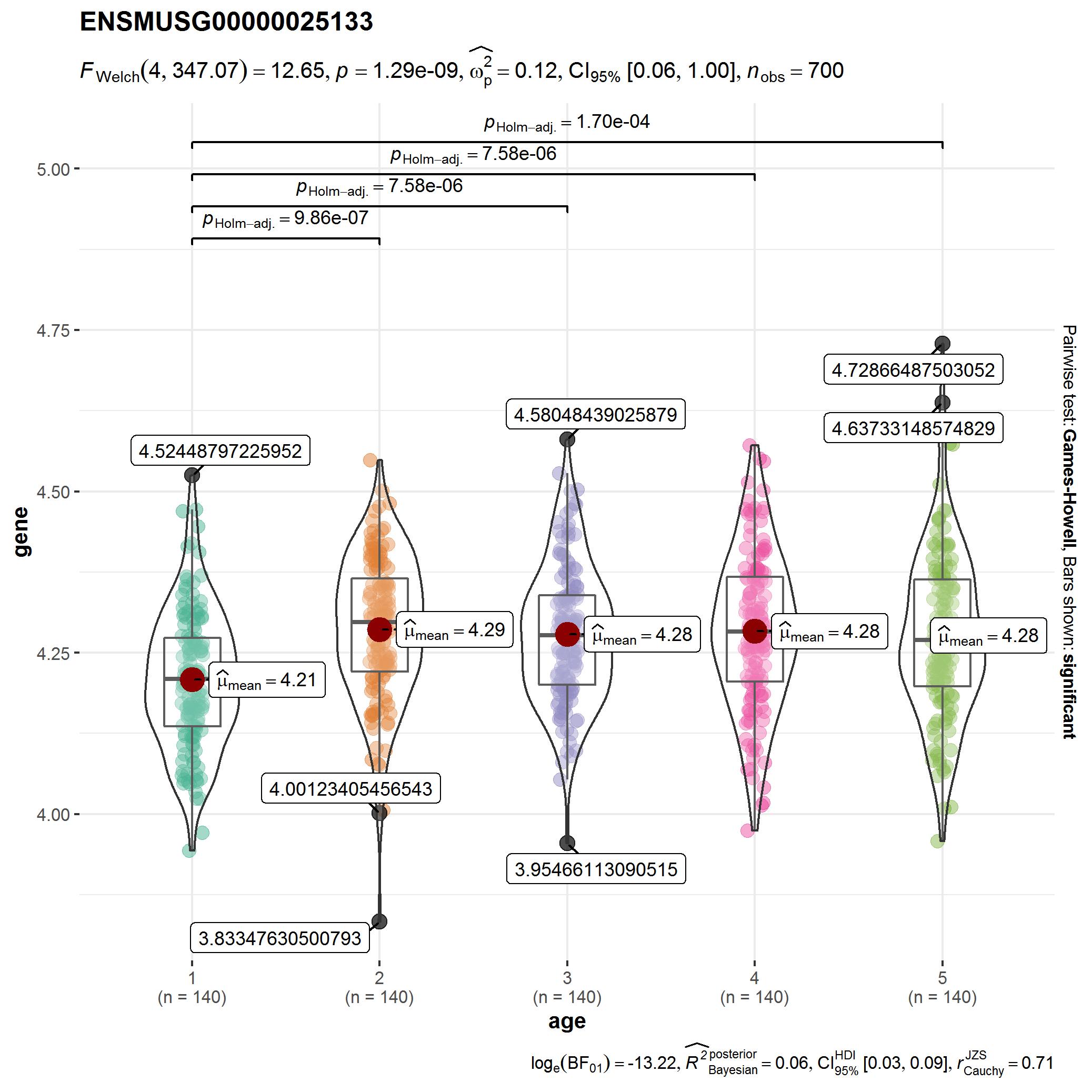

Supplement: Supplementary file 25 — Data S1–S6. [file ACEL-23-e14268-s017.zip › Data S1/ENSMUSG00000025133.jpeg]

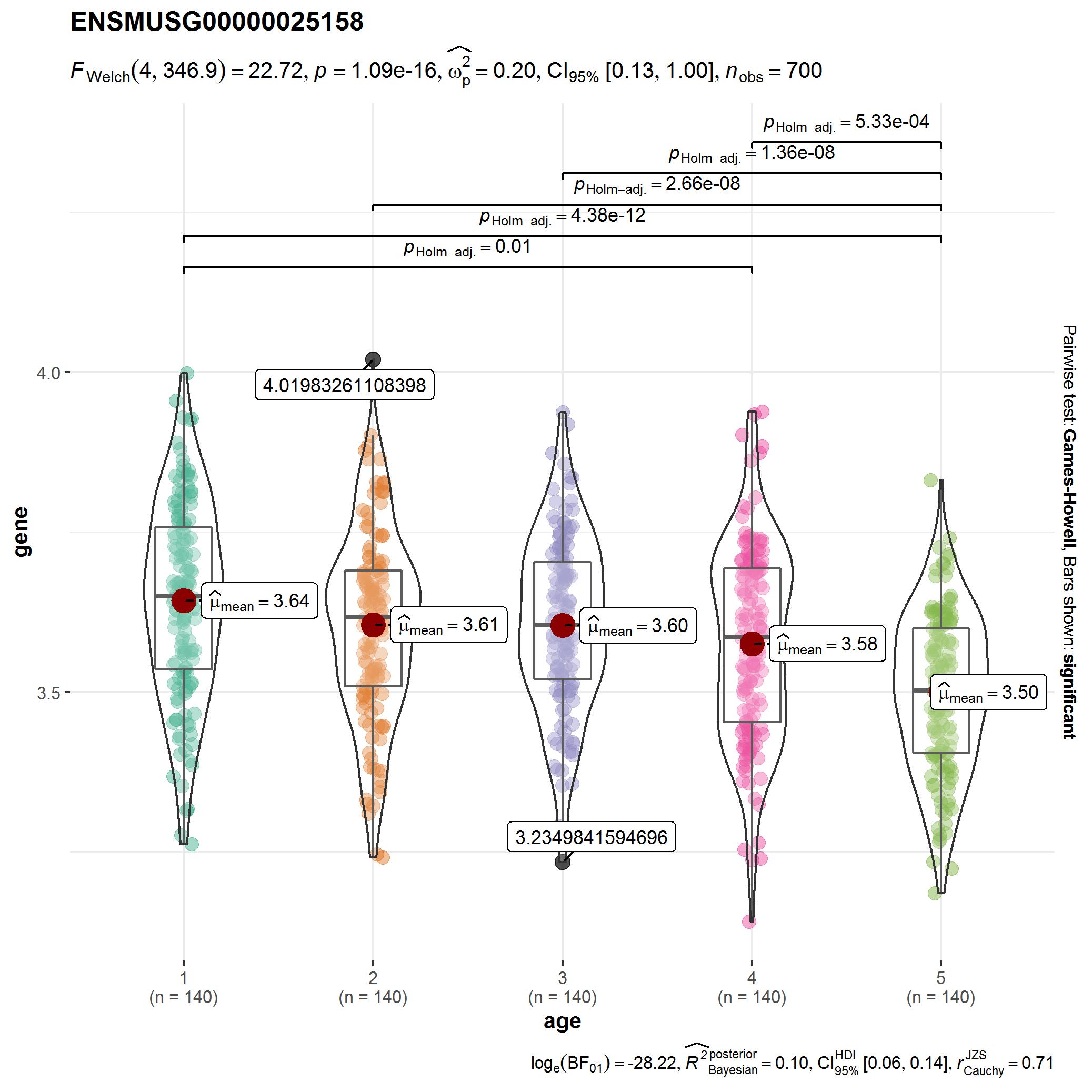

Supplement: Supplementary file 25 — Data S1–S6. [file ACEL-23-e14268-s017.zip › Data S1/ENSMUSG00000025158.jpeg]

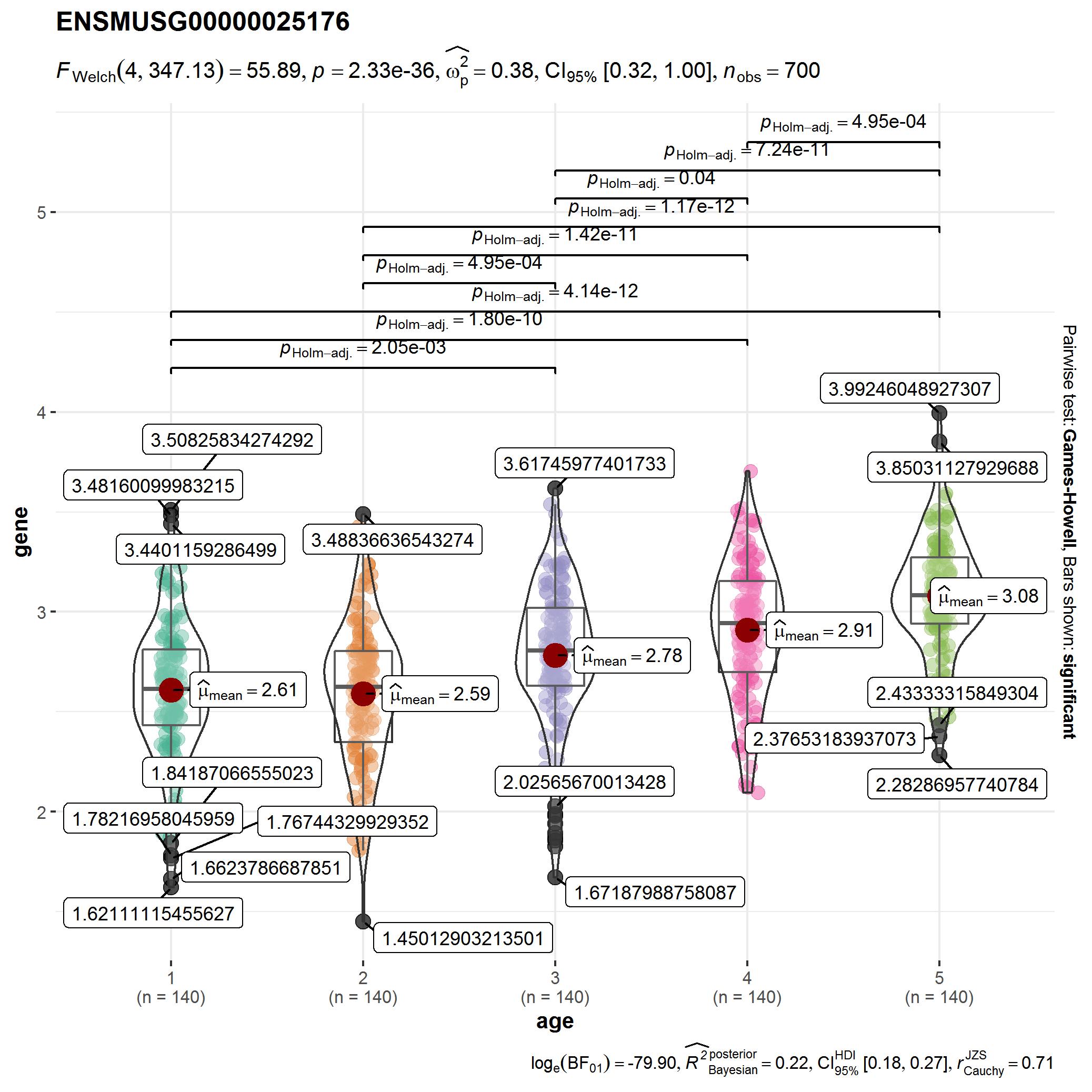

Supplement: Supplementary file 25 — Data S1–S6. [file ACEL-23-e14268-s017.zip › Data S1/ENSMUSG00000025176.jpeg]

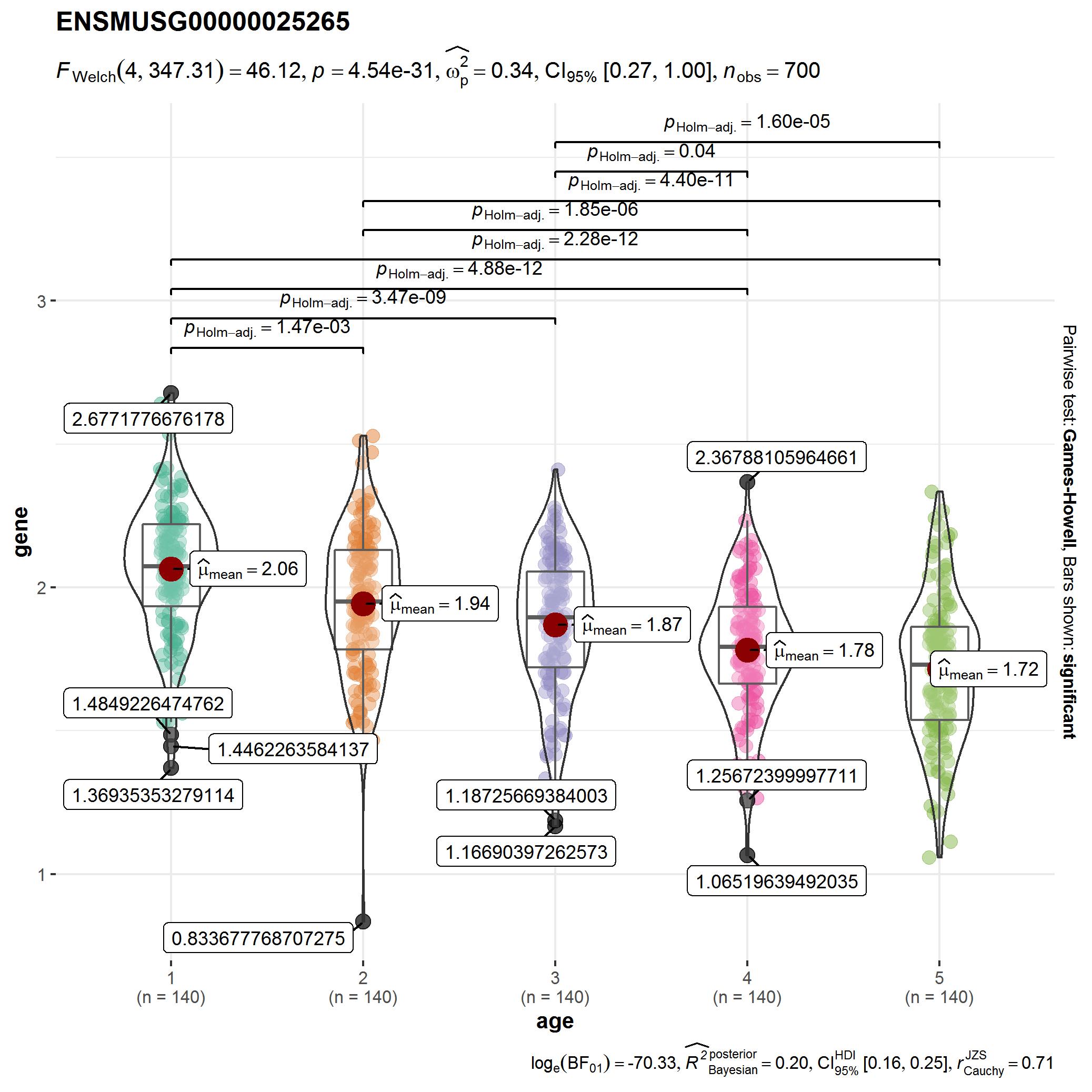

Supplement: Supplementary file 25 — Data S1–S6. [file ACEL-23-e14268-s017.zip › Data S1/ENSMUSG00000025265.jpeg]

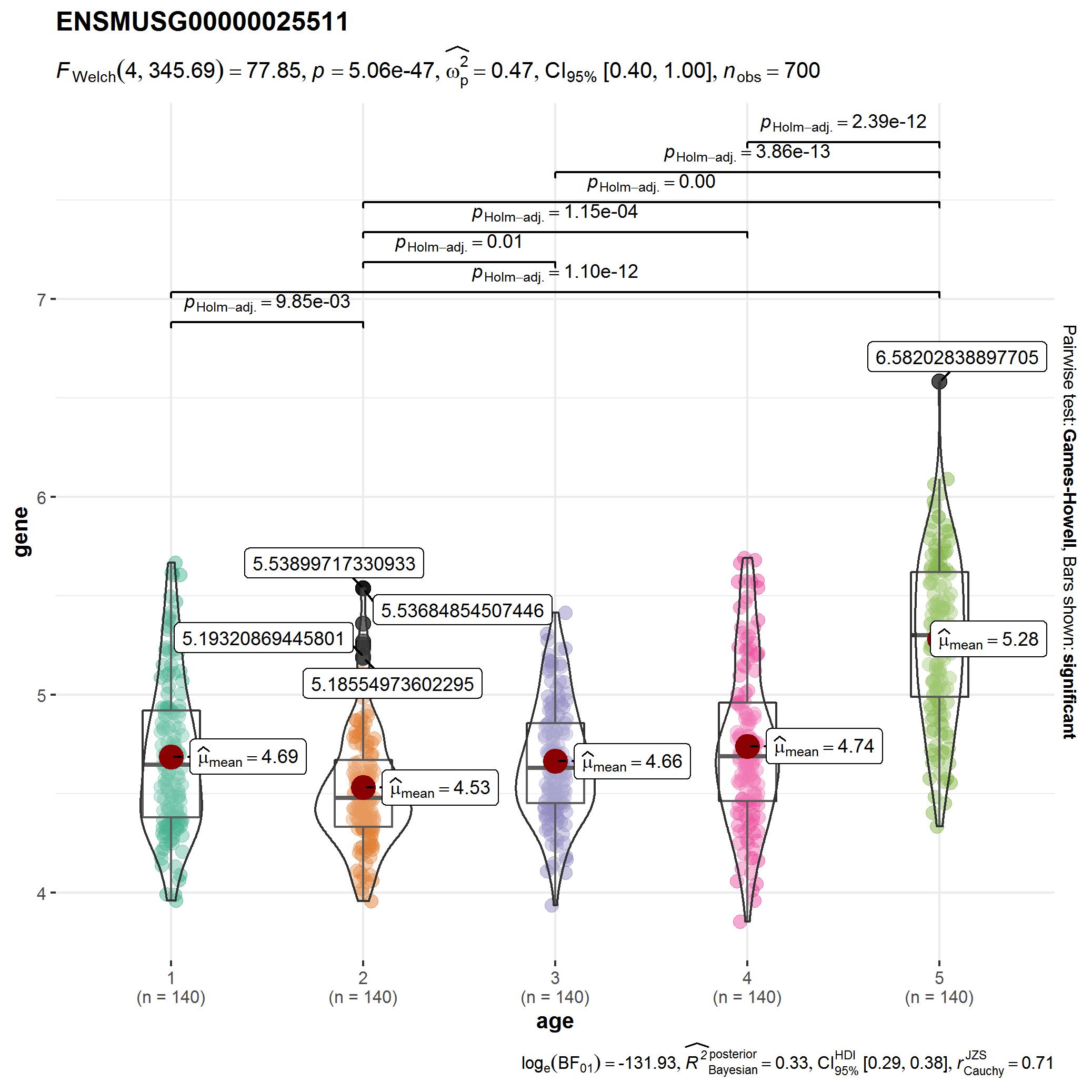

Supplement: Supplementary file 25 — Data S1–S6. [file ACEL-23-e14268-s017.zip › Data S1/ENSMUSG00000025511.jpeg]

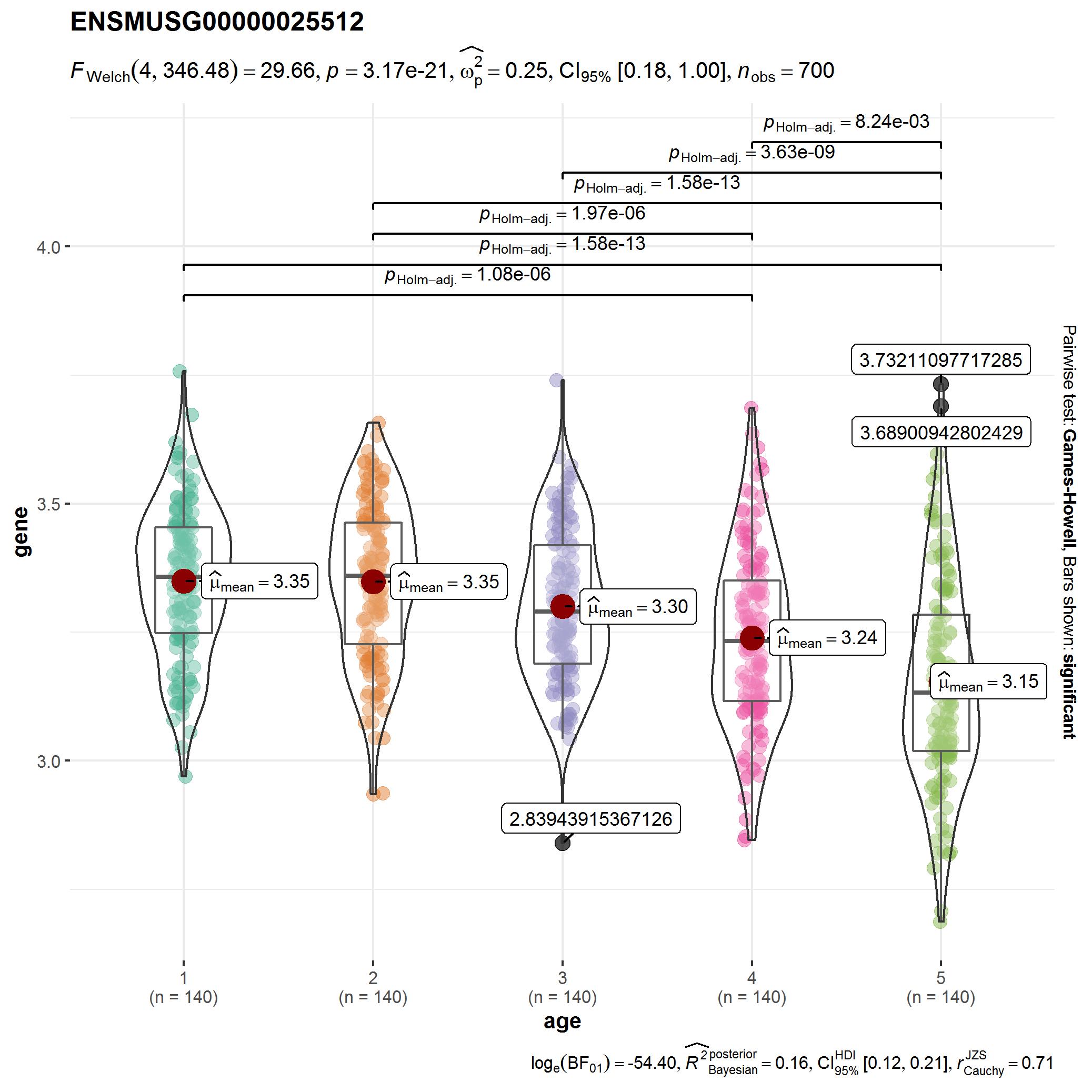

Supplement: Supplementary file 25 — Data S1–S6. [file ACEL-23-e14268-s017.zip › Data S1/ENSMUSG00000025512.jpeg]

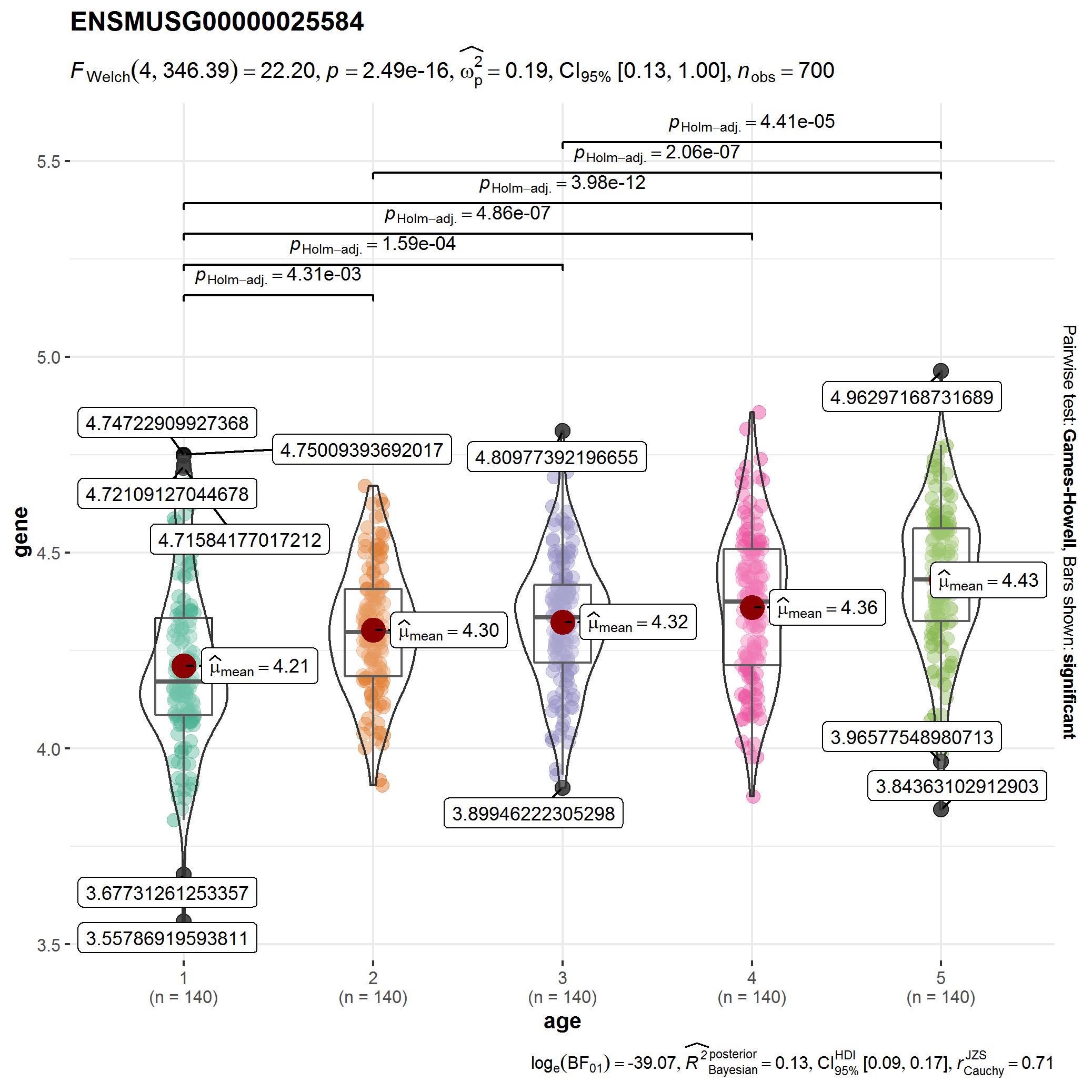

Supplement: Supplementary file 25 — Data S1–S6. [file ACEL-23-e14268-s017.zip › Data S1/ENSMUSG00000025584.jpeg]

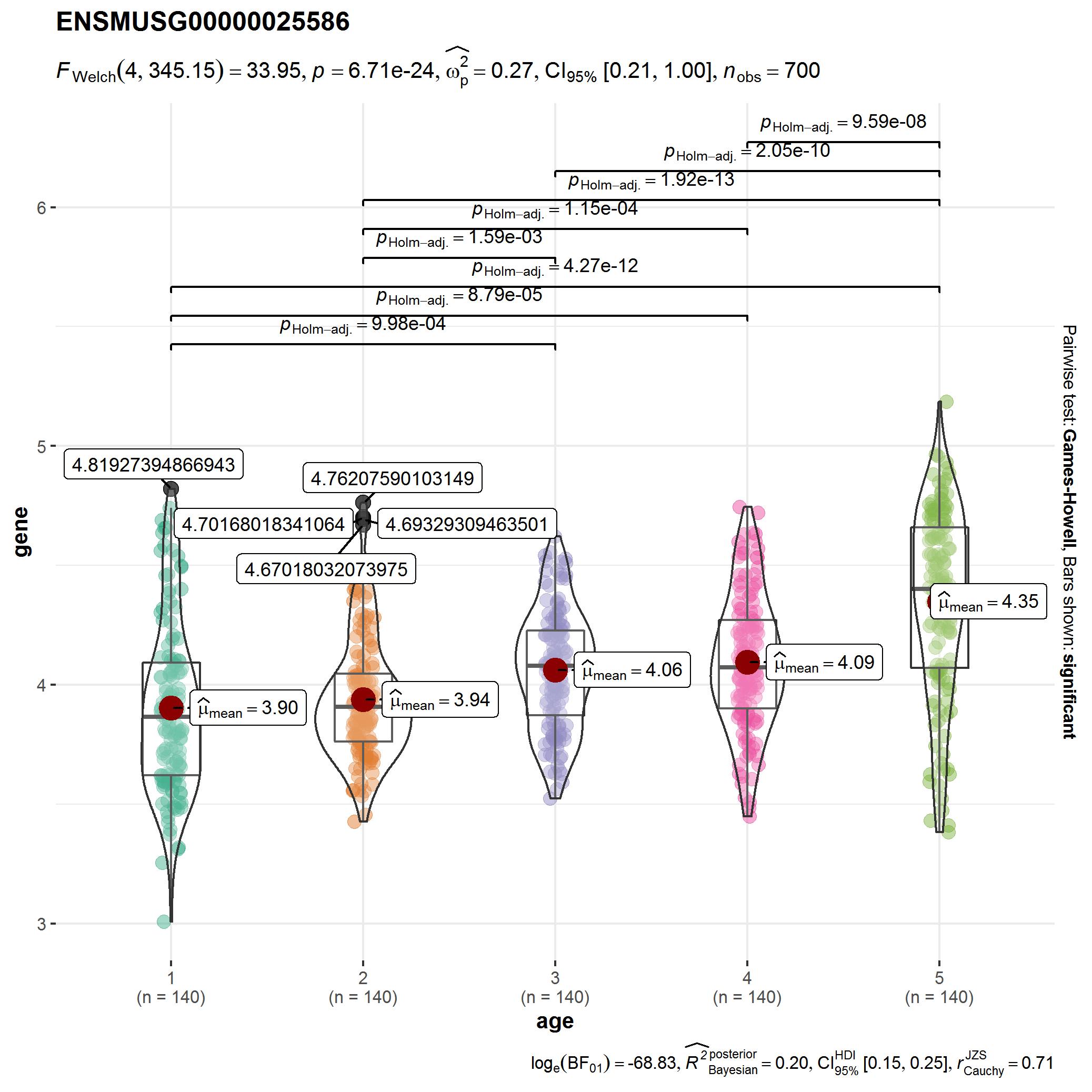

Supplement: Supplementary file 25 — Data S1–S6. [file ACEL-23-e14268-s017.zip › Data S1/ENSMUSG00000025586.jpeg]

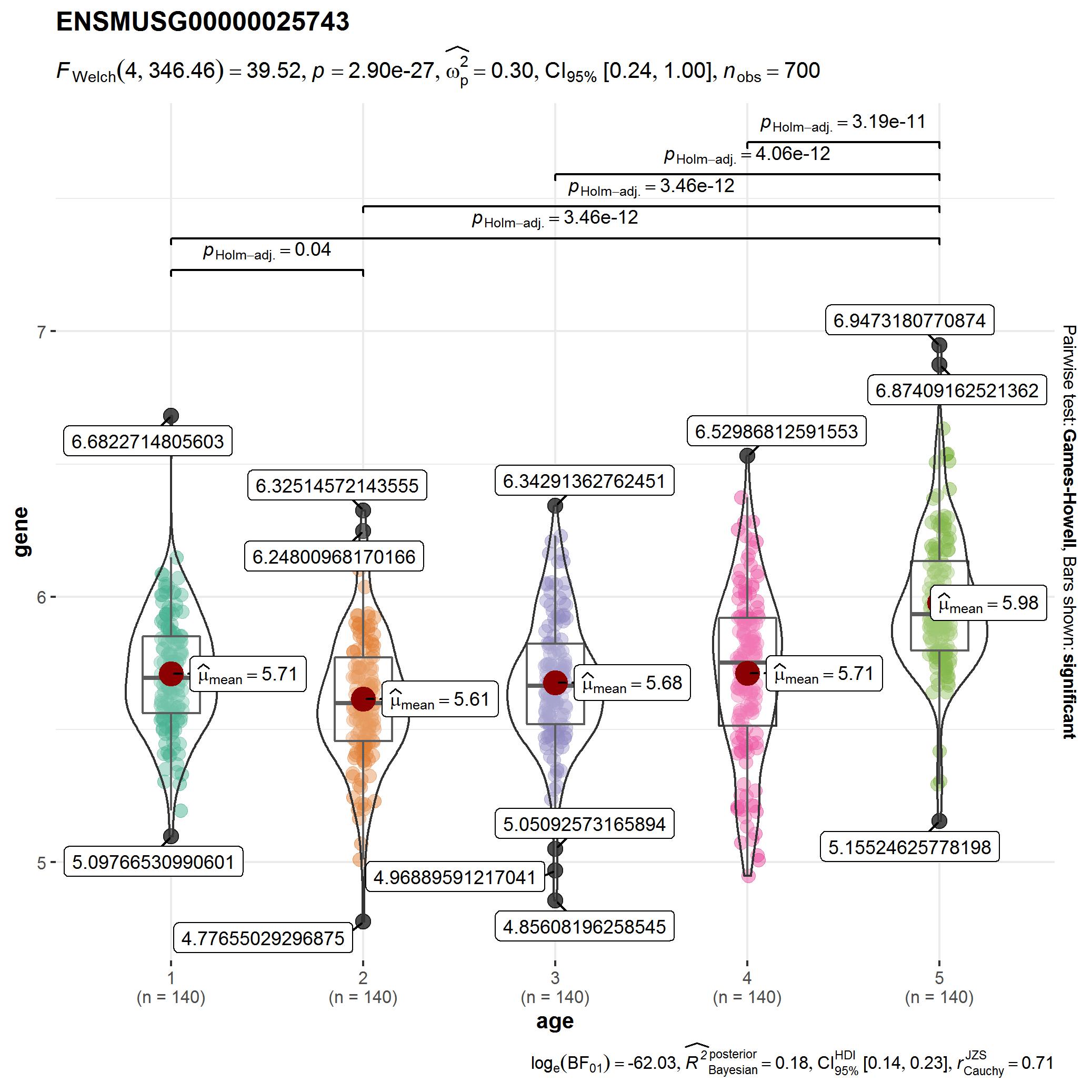

Supplement: Supplementary file 25 — Data S1–S6. [file ACEL-23-e14268-s017.zip › Data S1/ENSMUSG00000025743.jpeg]

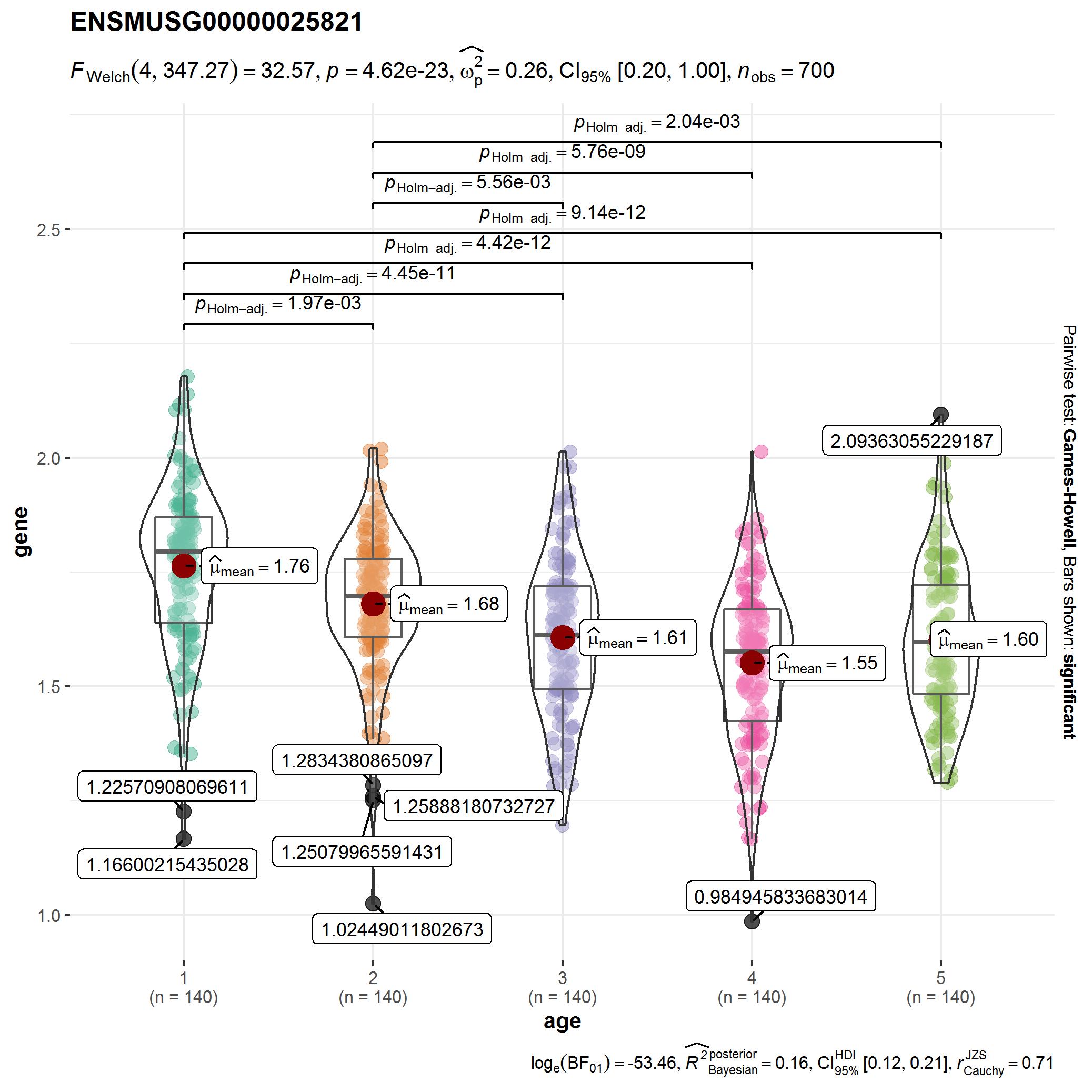

Supplement: Supplementary file 25 — Data S1–S6. [file ACEL-23-e14268-s017.zip › Data S1/ENSMUSG00000025821.jpeg]

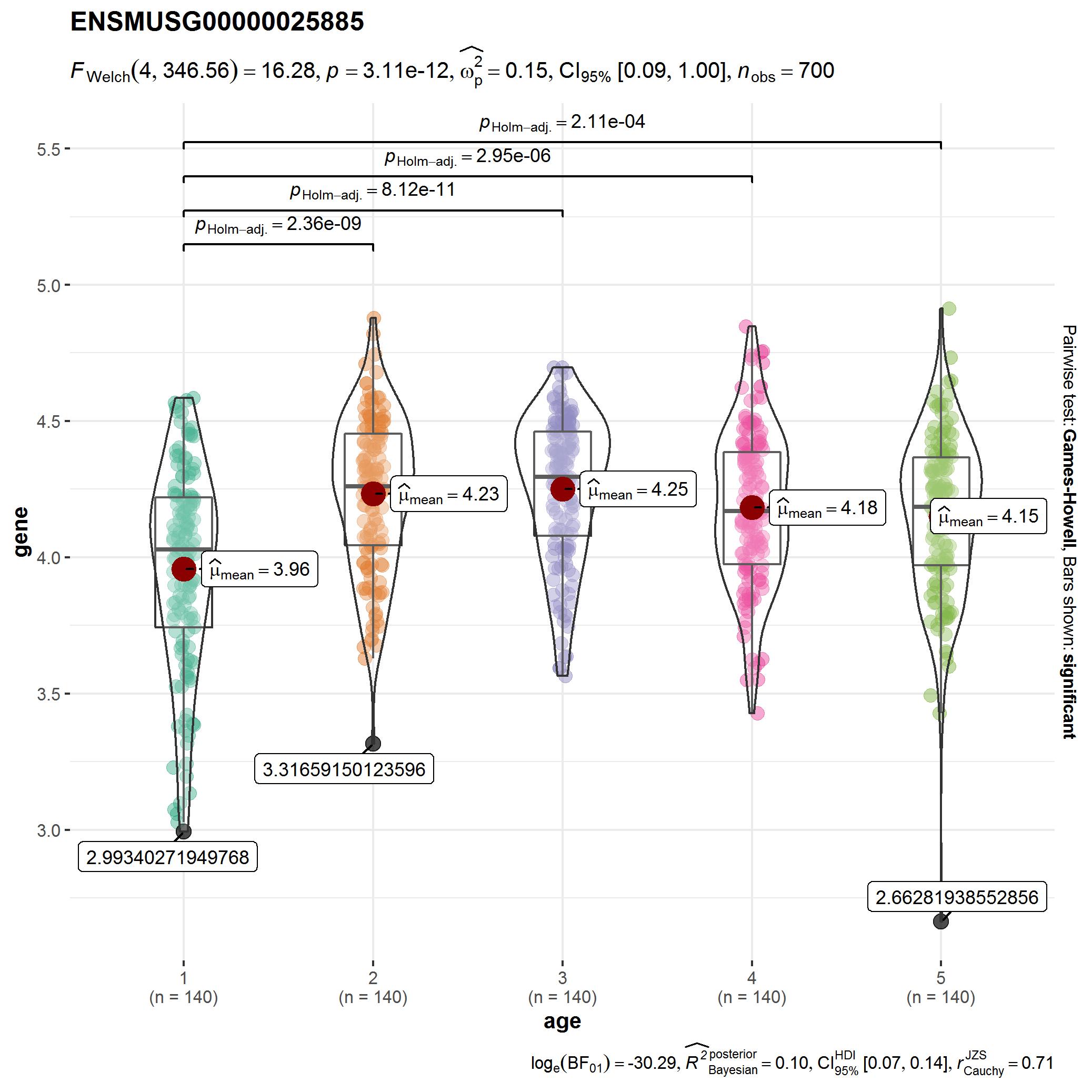

Supplement: Supplementary file 25 — Data S1–S6. [file ACEL-23-e14268-s017.zip › Data S1/ENSMUSG00000025885.jpeg]

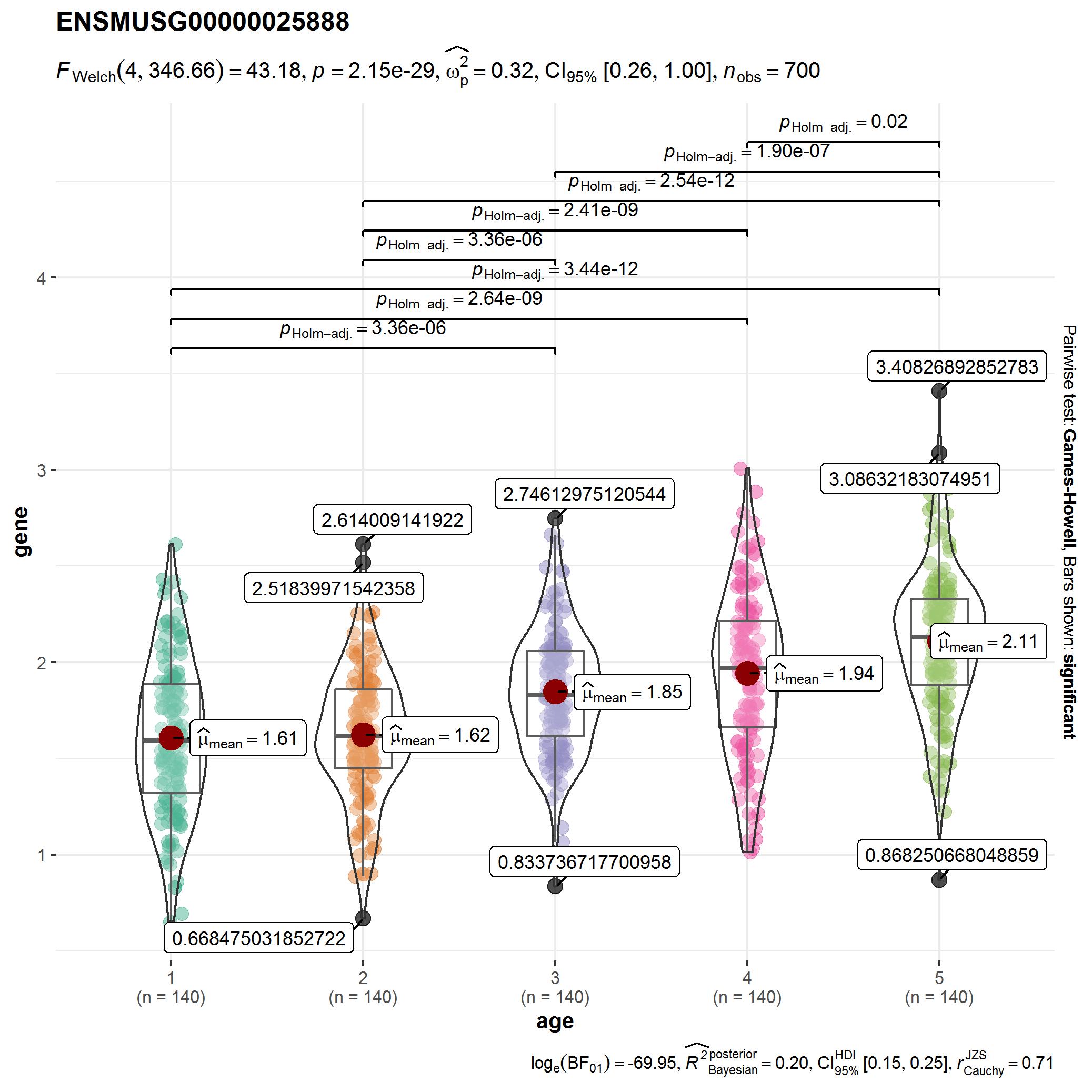

Supplement: Supplementary file 25 — Data S1–S6. [file ACEL-23-e14268-s017.zip › Data S1/ENSMUSG00000025888.jpeg]

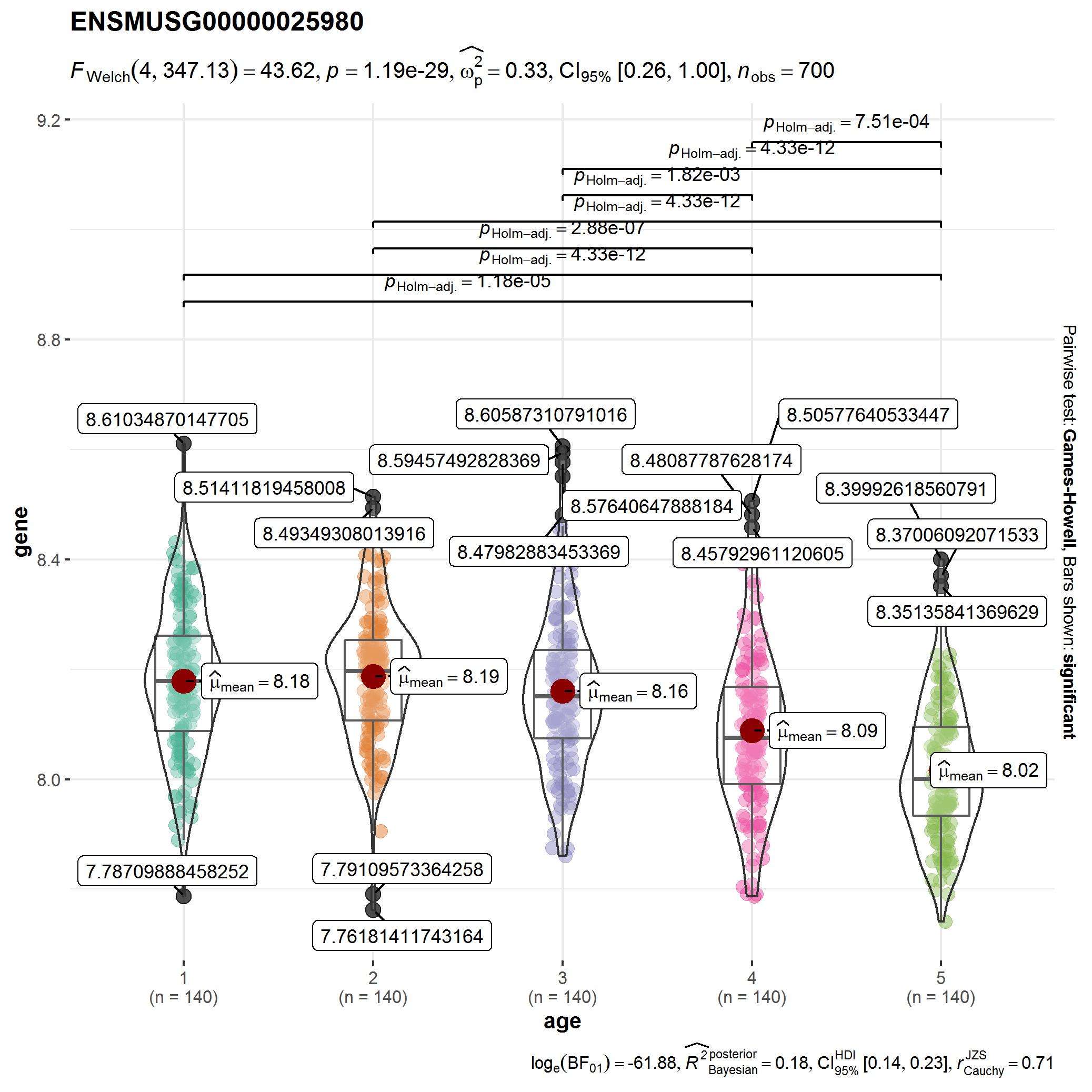

Supplement: Supplementary file 25 — Data S1–S6. [file ACEL-23-e14268-s017.zip › Data S1/ENSMUSG00000025980.jpeg]

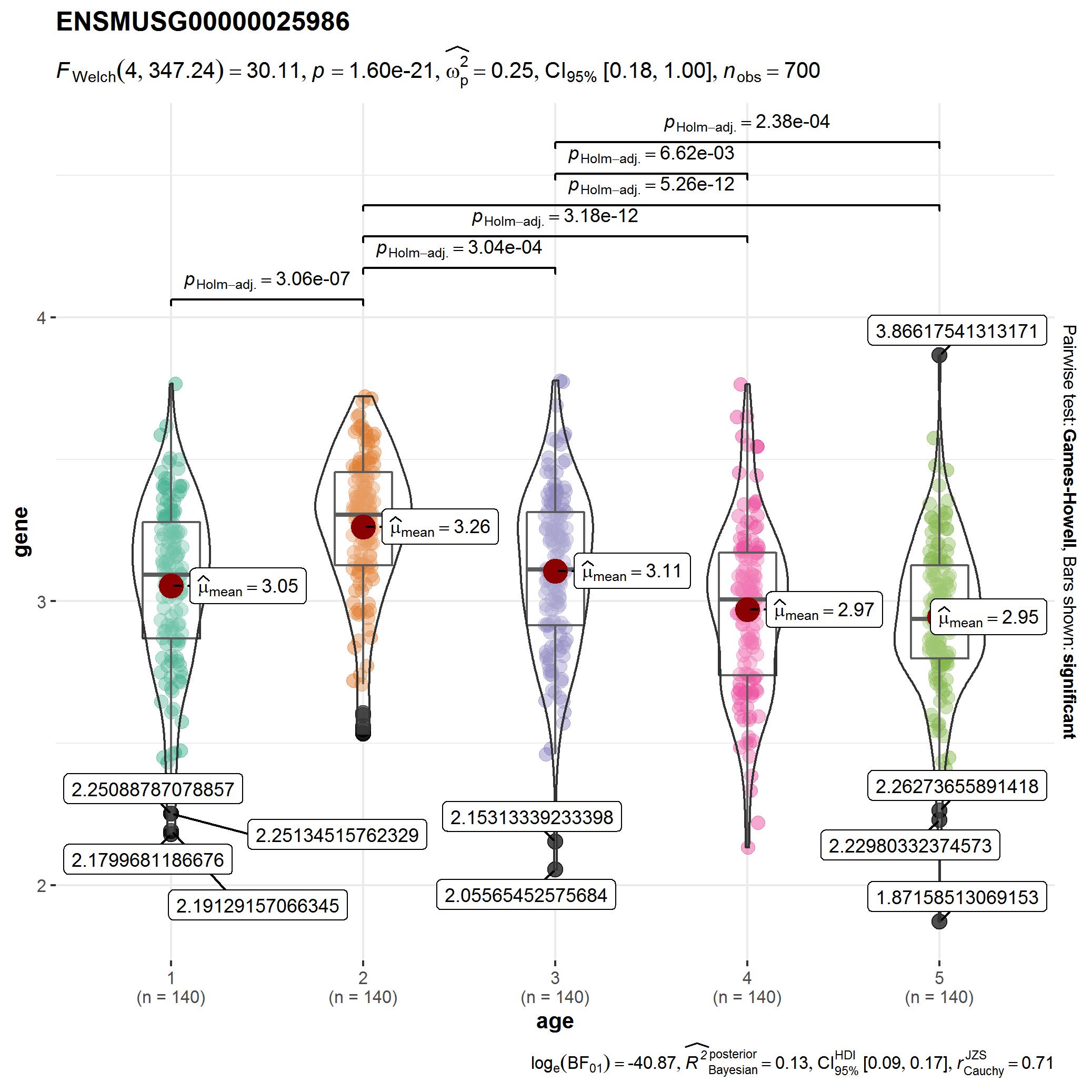

Supplement: Supplementary file 25 — Data S1–S6. [file ACEL-23-e14268-s017.zip › Data S1/ENSMUSG00000025986.jpeg]

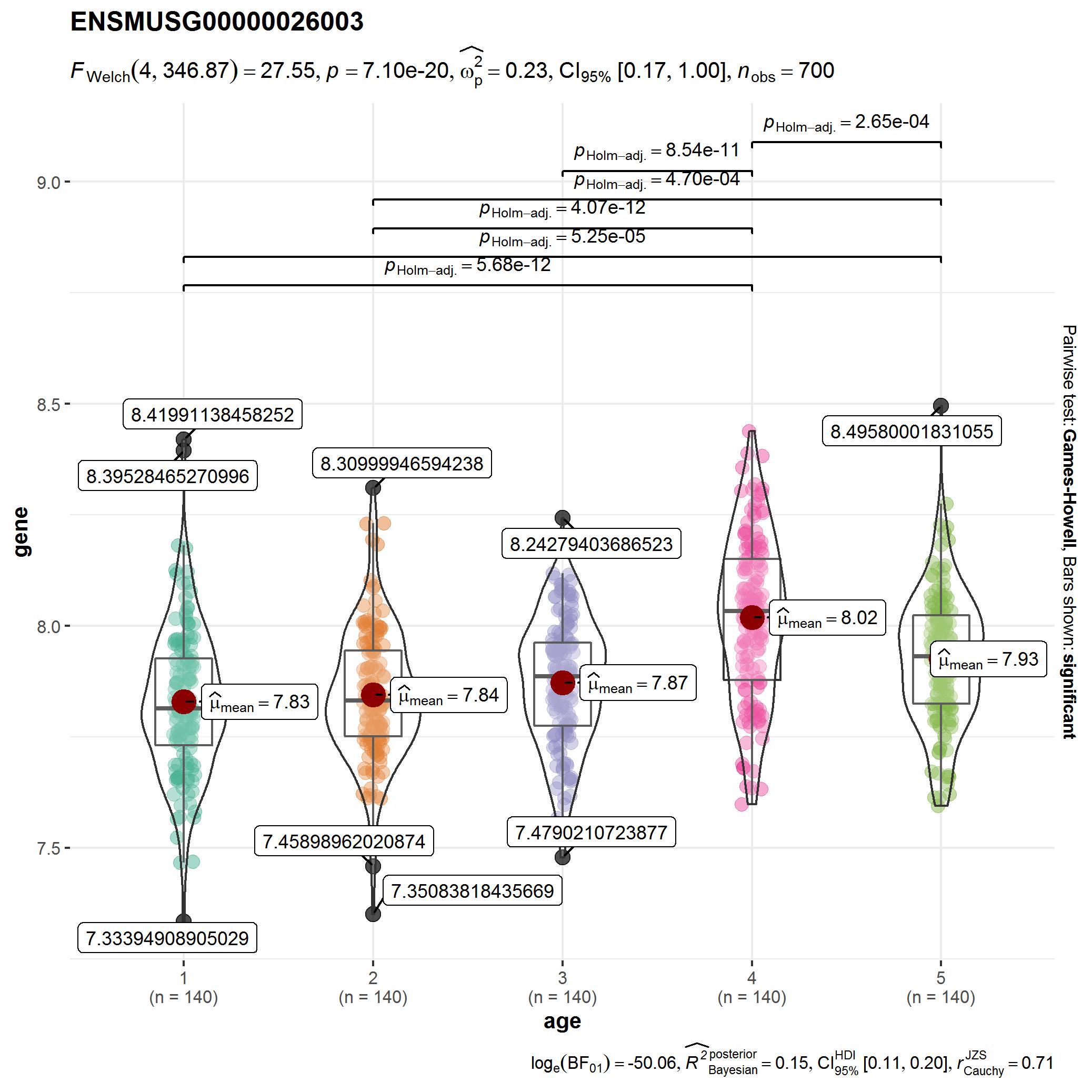

Supplement: Supplementary file 25 — Data S1–S6. [file ACEL-23-e14268-s017.zip › Data S1/ENSMUSG00000026003.jpeg]

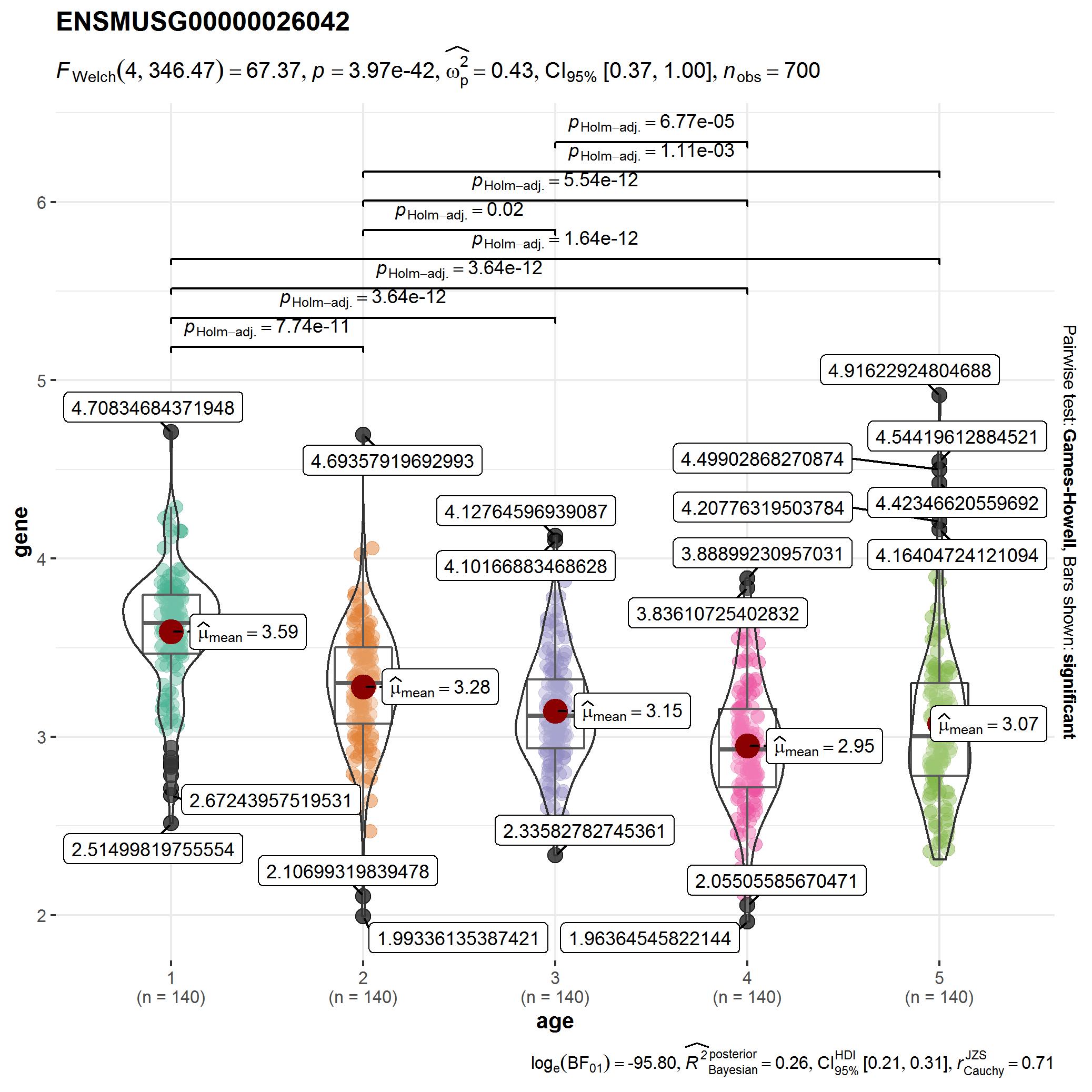

Supplement: Supplementary file 25 — Data S1–S6. [file ACEL-23-e14268-s017.zip › Data S1/ENSMUSG00000026042.jpeg]

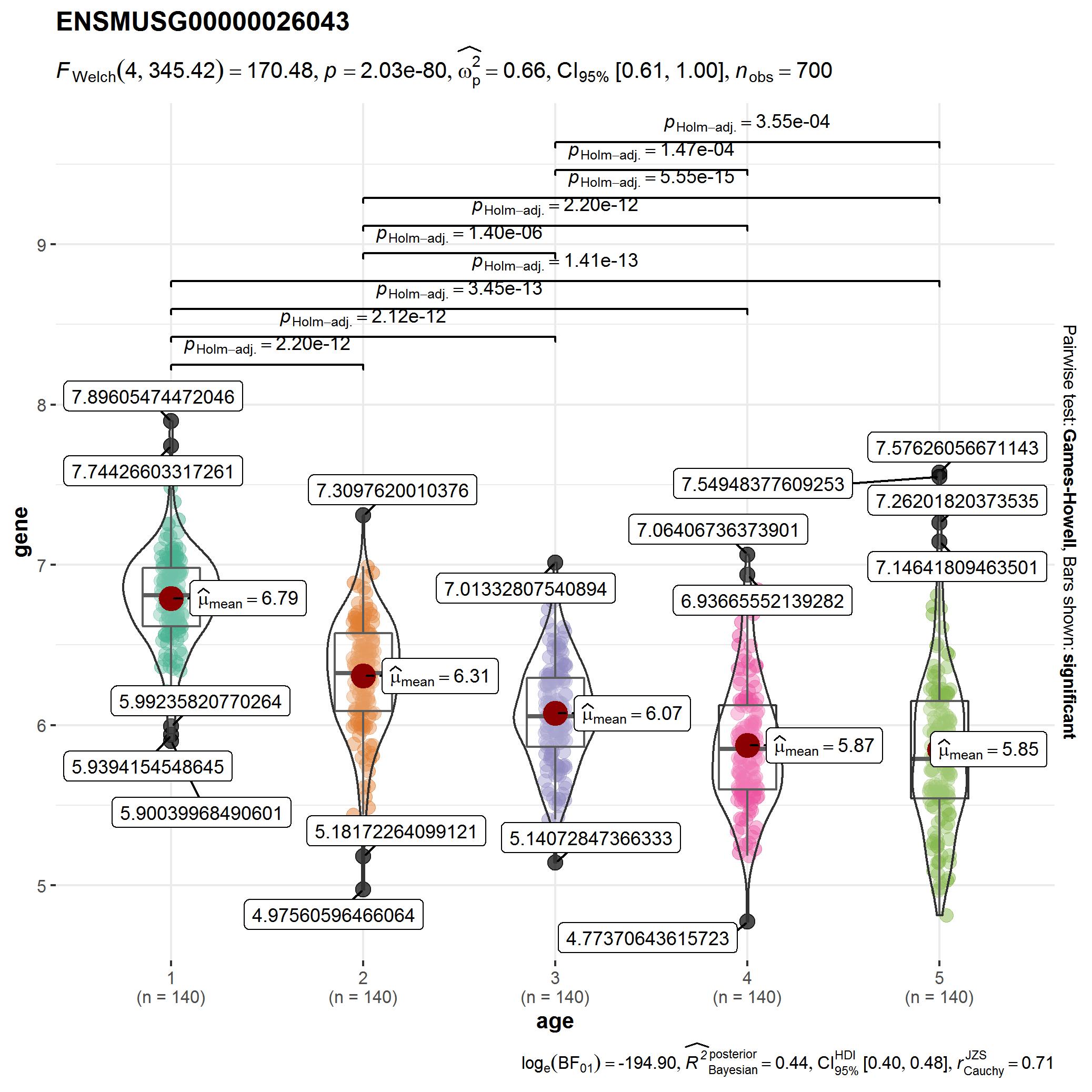

Supplement: Supplementary file 25 — Data S1–S6. [file ACEL-23-e14268-s017.zip › Data S1/ENSMUSG00000026043.jpeg]

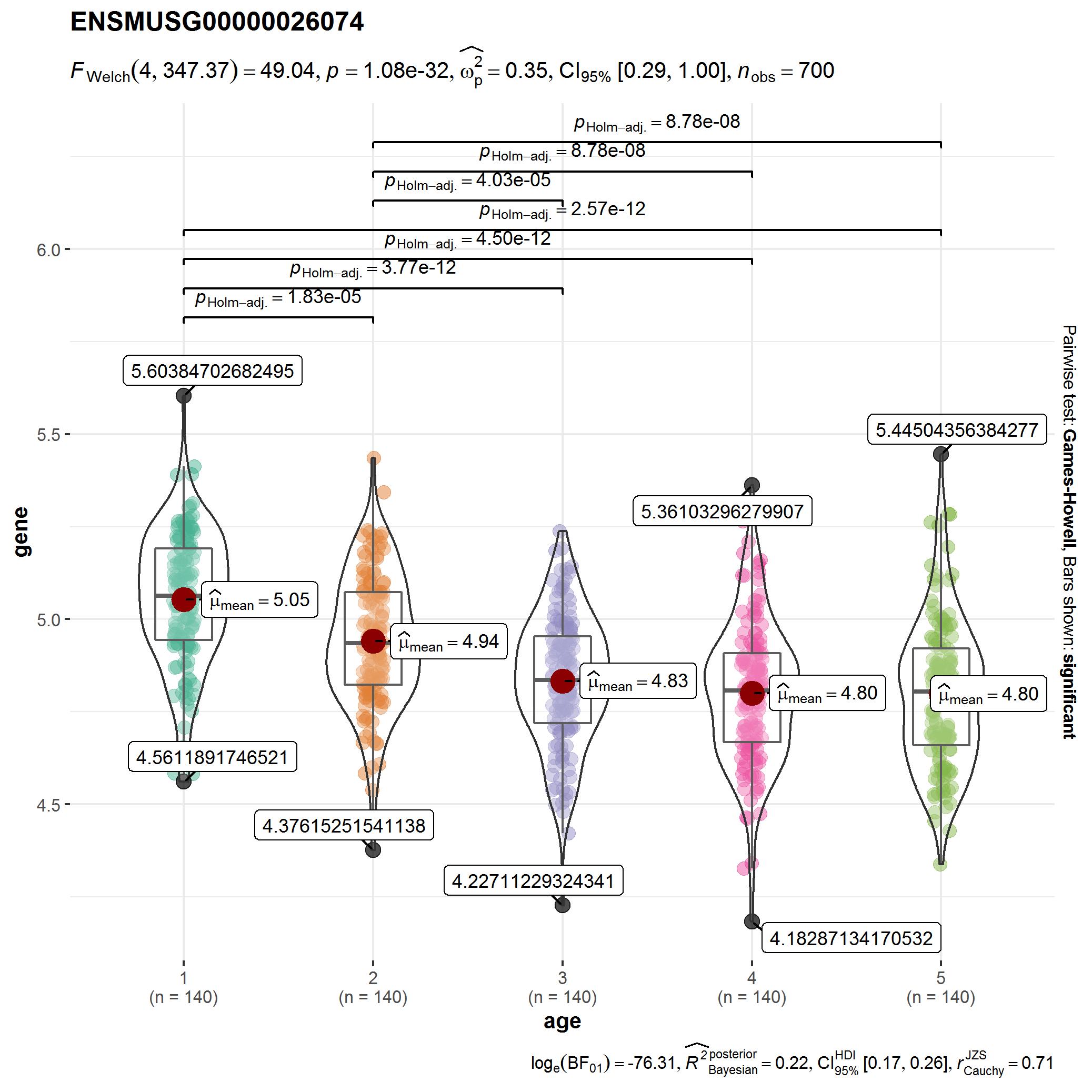

Supplement: Supplementary file 25 — Data S1–S6. [file ACEL-23-e14268-s017.zip › Data S1/ENSMUSG00000026074.jpeg]

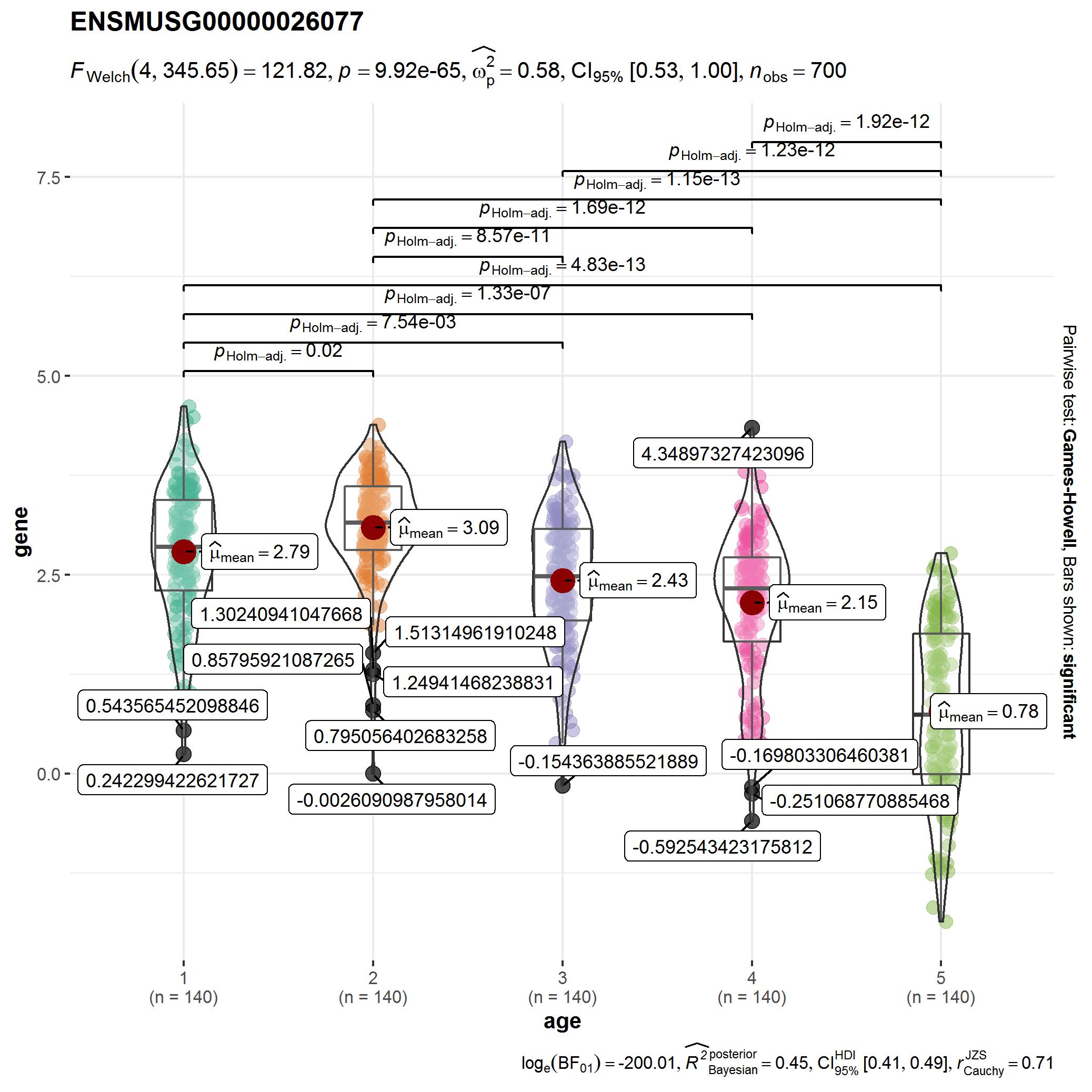

Supplement: Supplementary file 25 — Data S1–S6. [file ACEL-23-e14268-s017.zip › Data S1/ENSMUSG00000026077.jpeg]

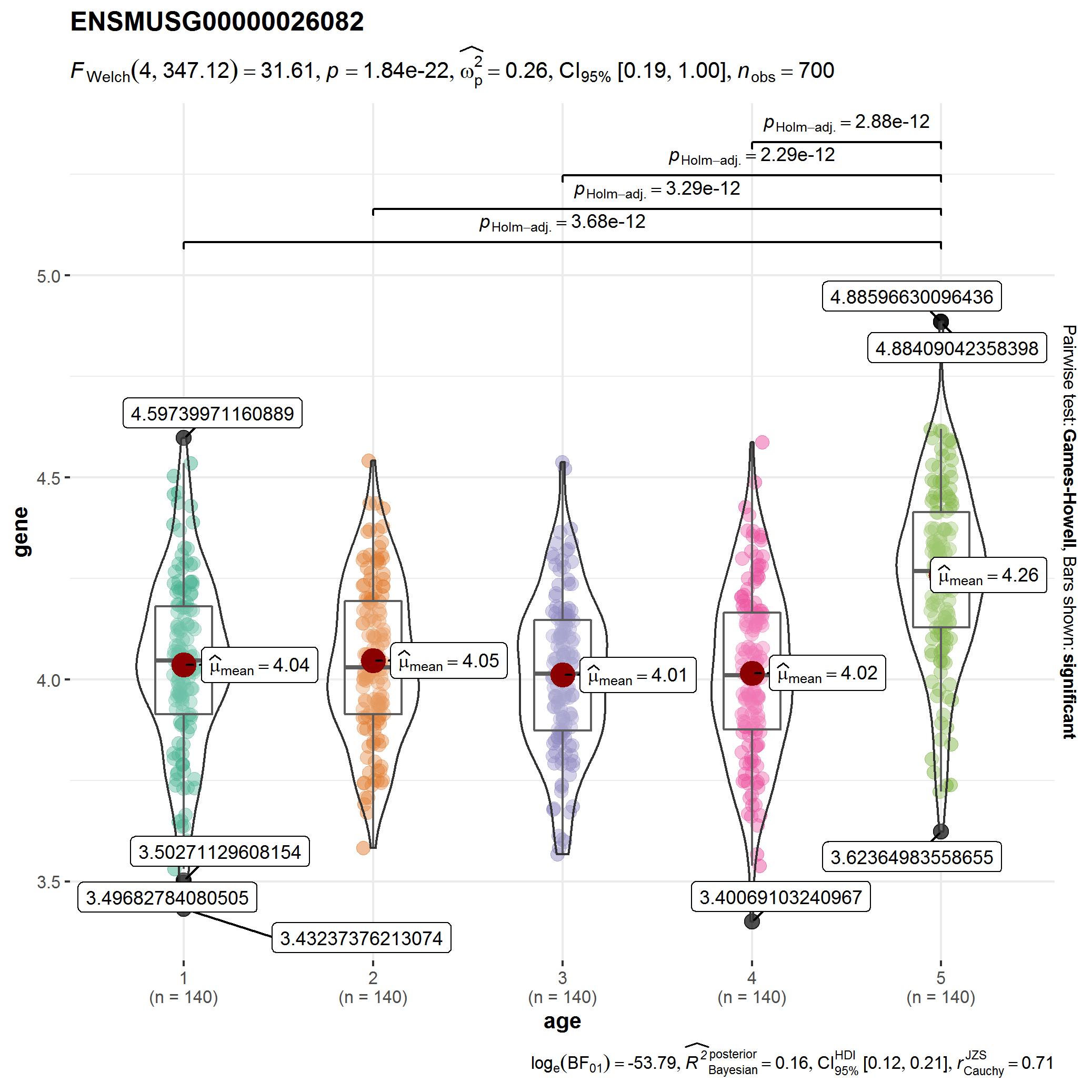

Supplement: Supplementary file 25 — Data S1–S6. [file ACEL-23-e14268-s017.zip › Data S1/ENSMUSG00000026082.jpeg]

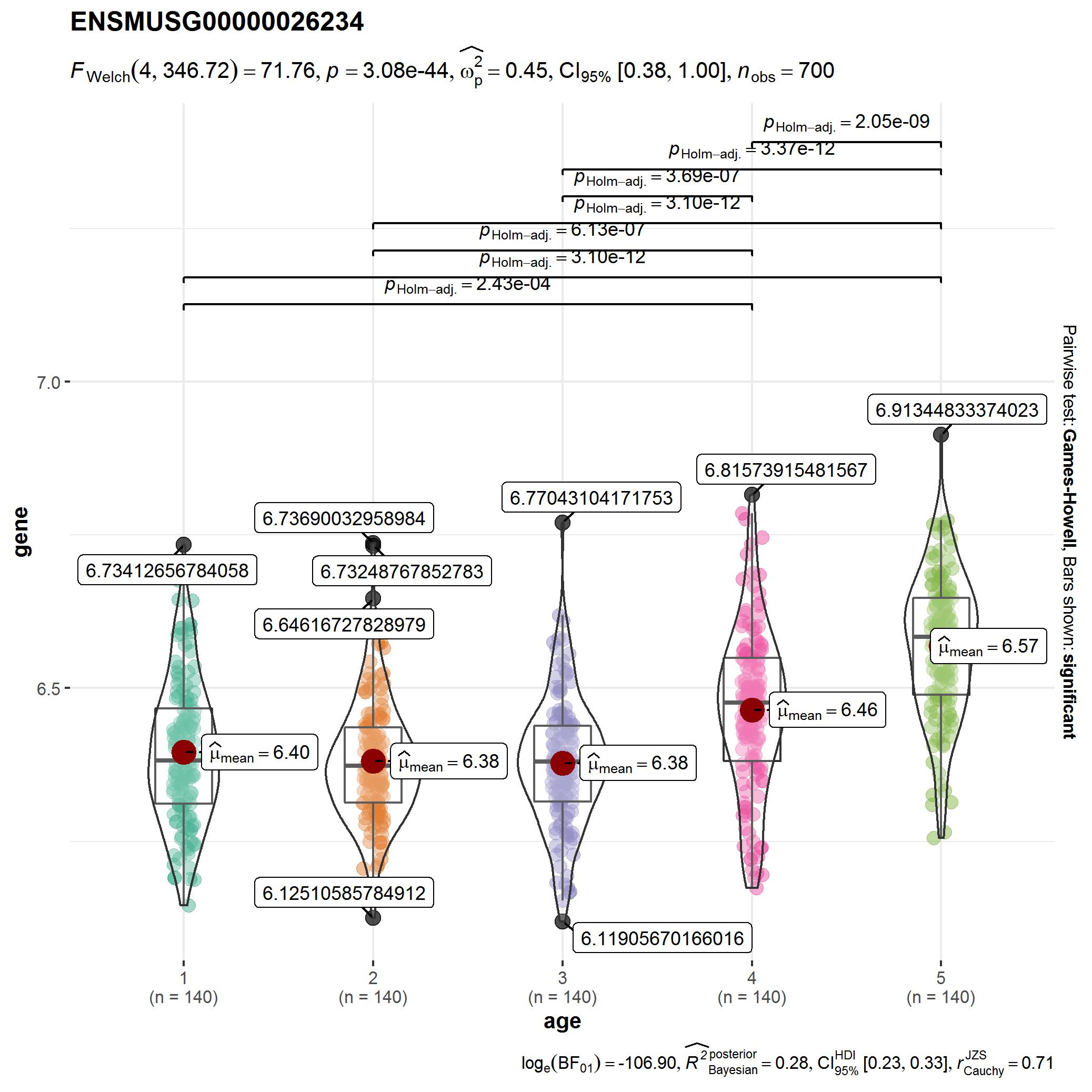

Supplement: Supplementary file 25 — Data S1–S6. [file ACEL-23-e14268-s017.zip › Data S1/ENSMUSG00000026234.jpeg]

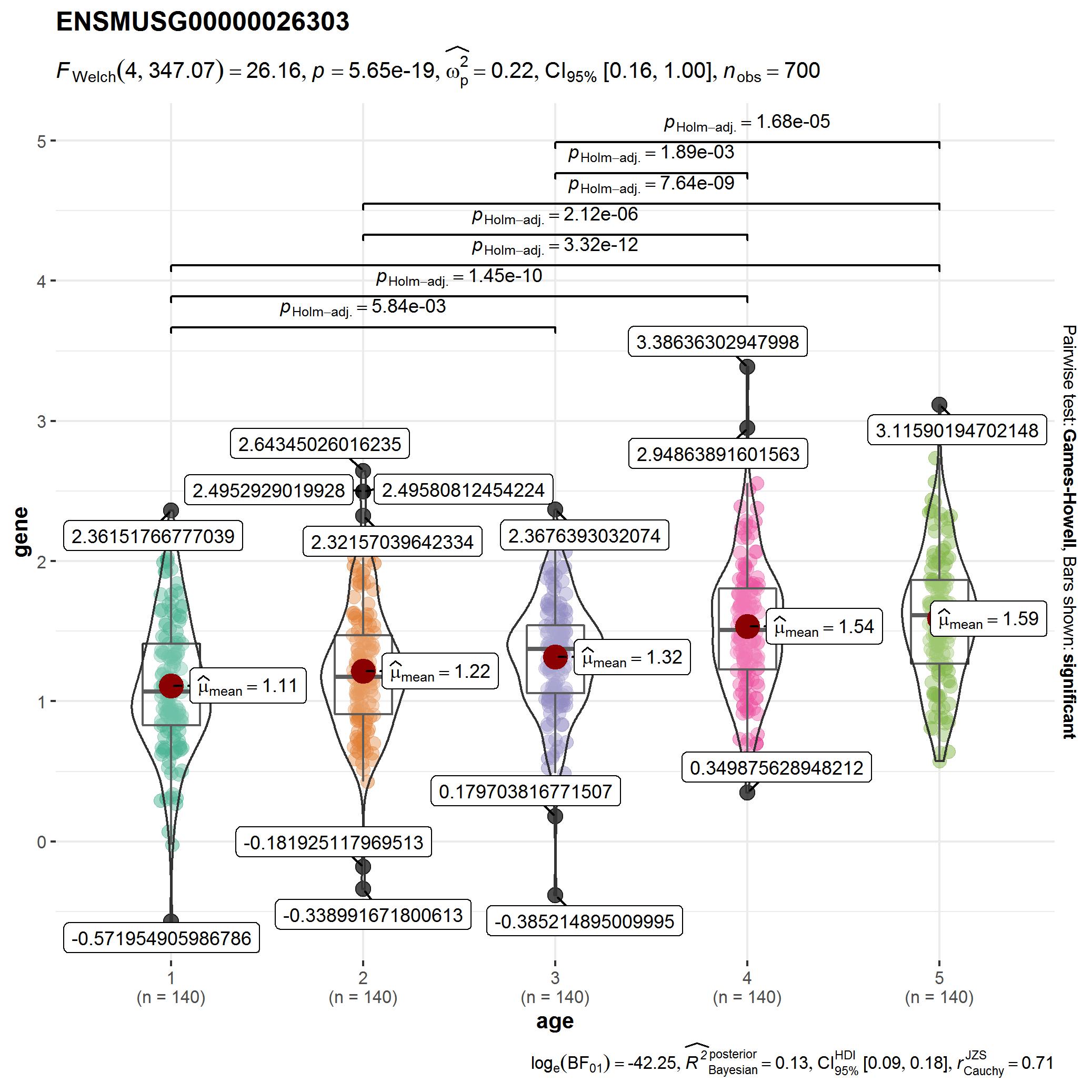

Supplement: Supplementary file 25 — Data S1–S6. [file ACEL-23-e14268-s017.zip › Data S1/ENSMUSG00000026303.jpeg]

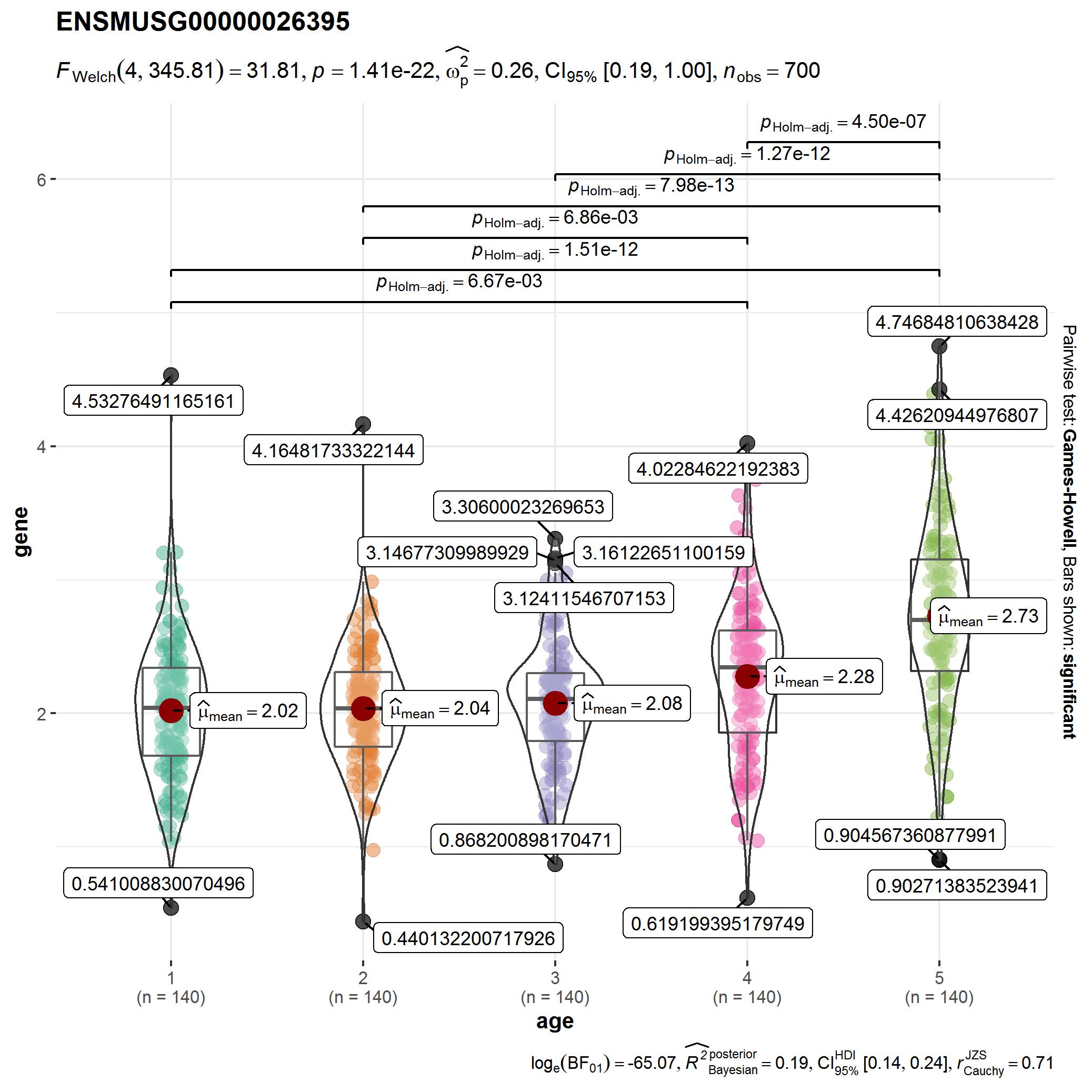

Supplement: Supplementary file 25 — Data S1–S6. [file ACEL-23-e14268-s017.zip › Data S1/ENSMUSG00000026395.jpeg]

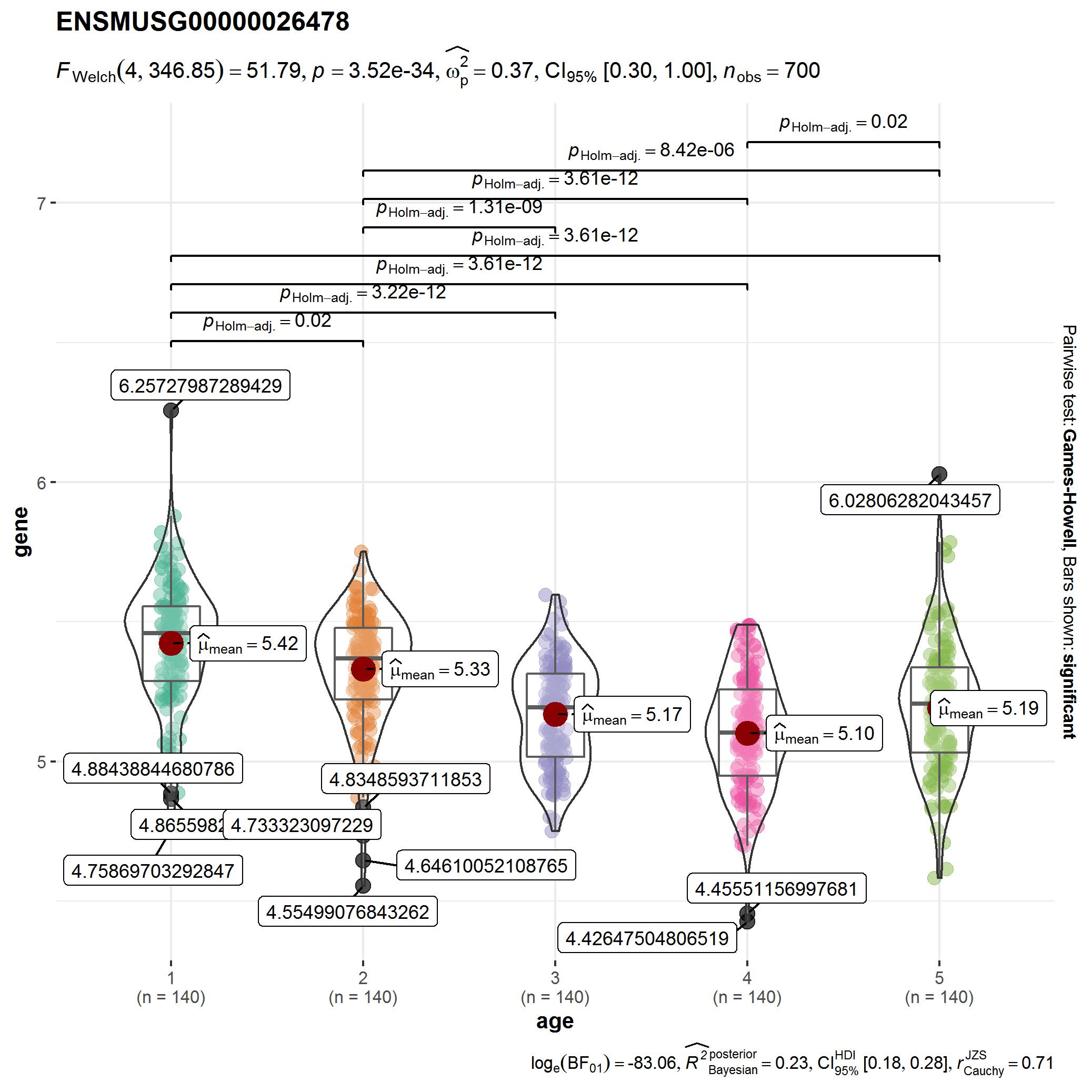

Supplement: Supplementary file 25 — Data S1–S6. [file ACEL-23-e14268-s017.zip › Data S1/ENSMUSG00000026478.jpeg]

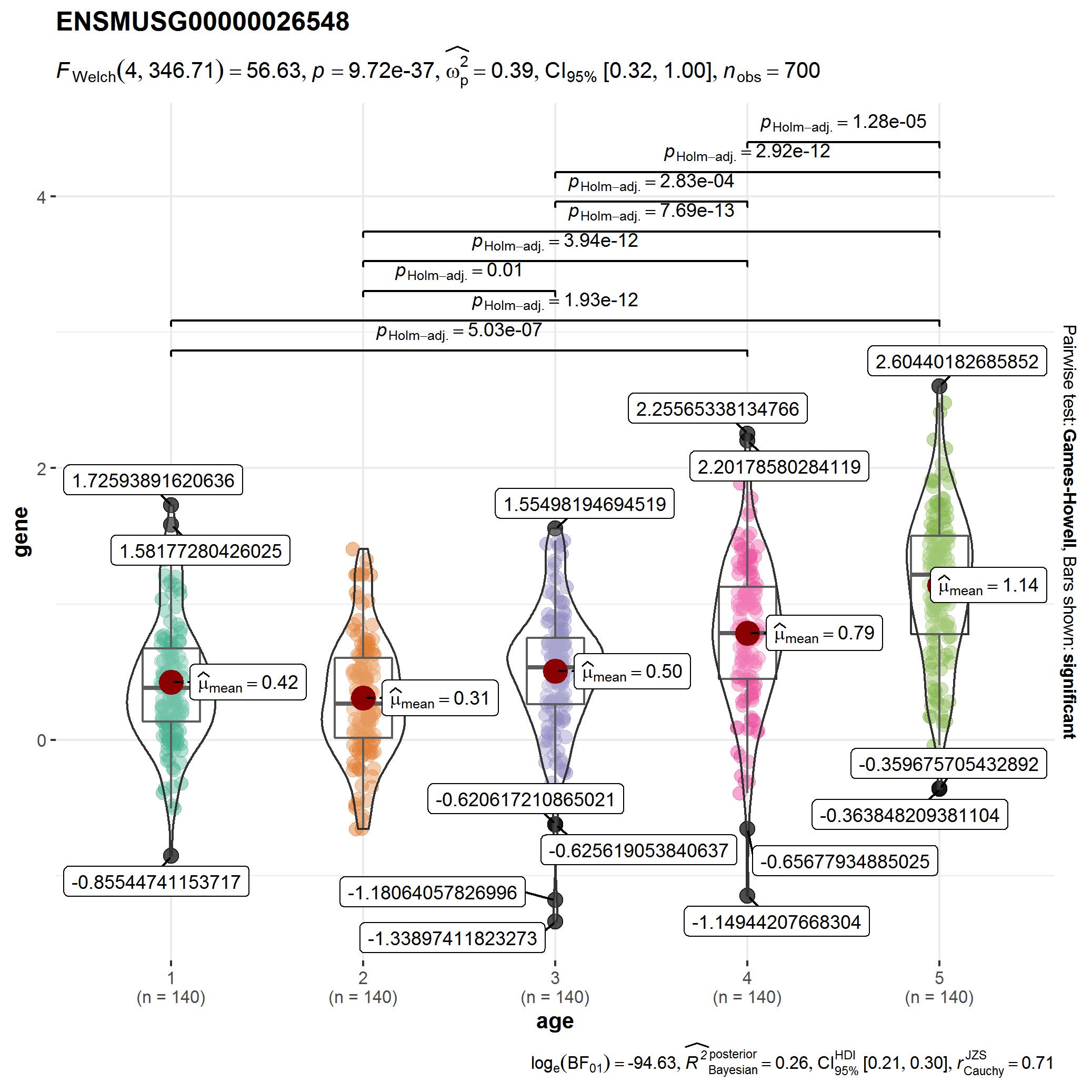

Supplement: Supplementary file 25 — Data S1–S6. [file ACEL-23-e14268-s017.zip › Data S1/ENSMUSG00000026548.jpeg]

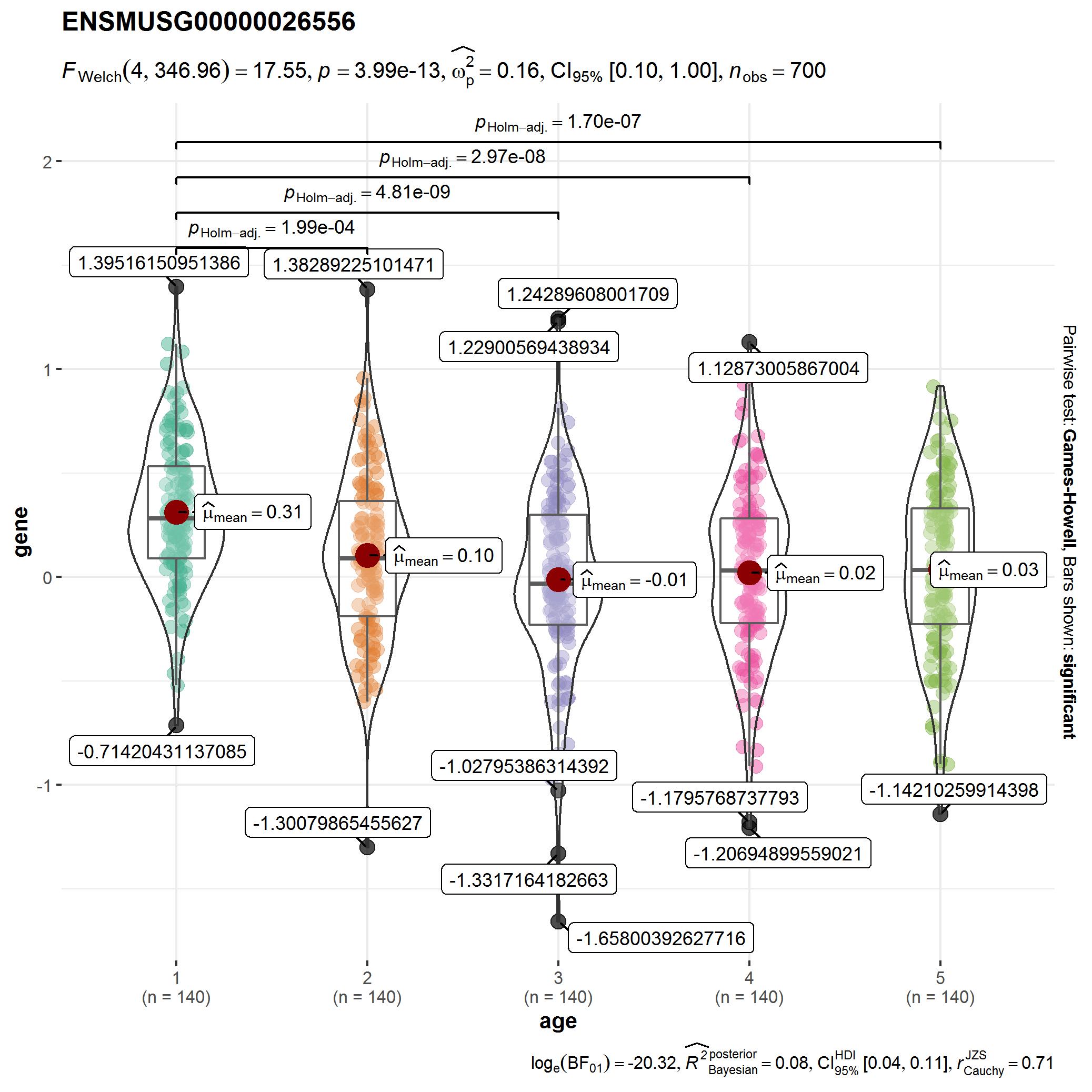

Supplement: Supplementary file 25 — Data S1–S6. [file ACEL-23-e14268-s017.zip › Data S1/ENSMUSG00000026556.jpeg]

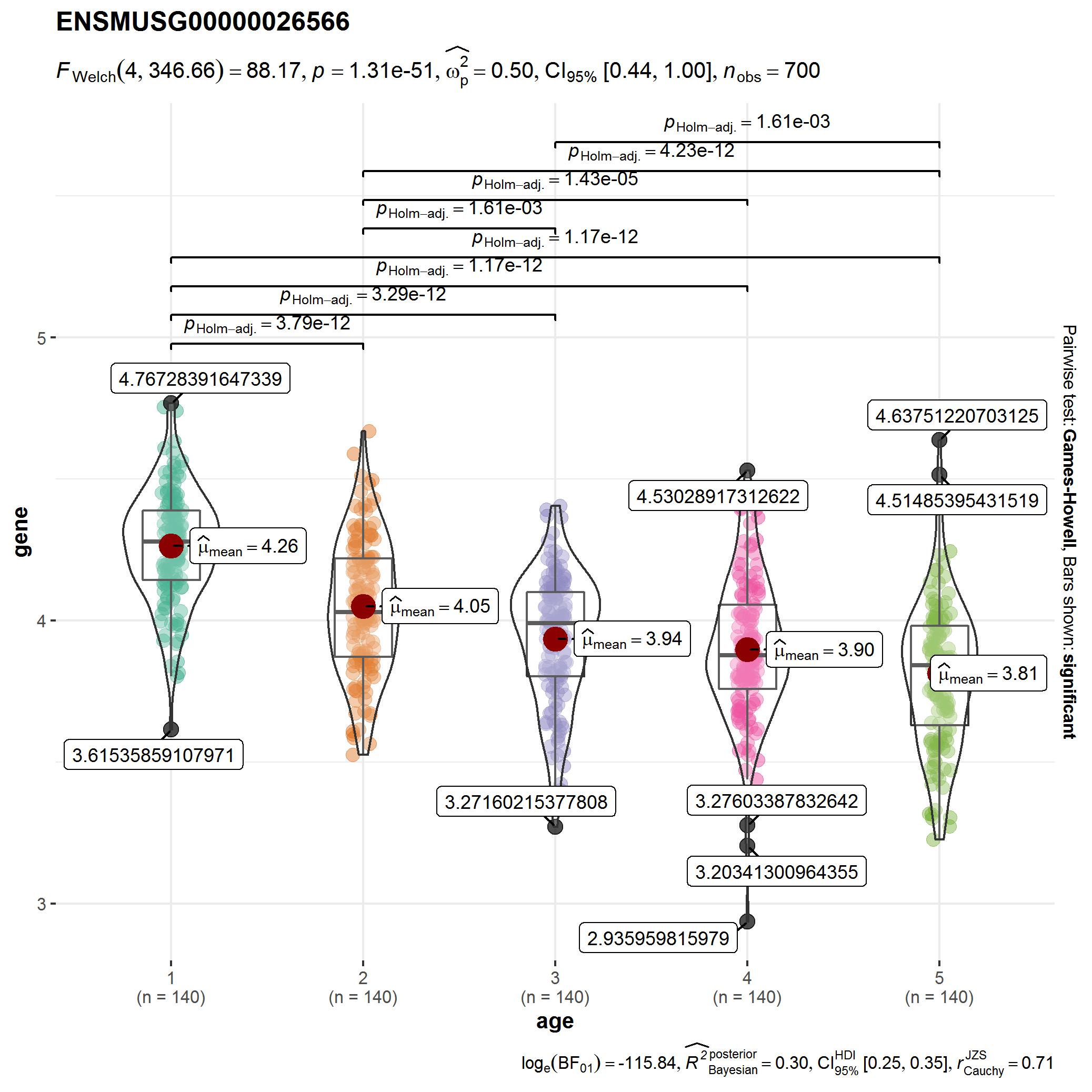

Supplement: Supplementary file 25 — Data S1–S6. [file ACEL-23-e14268-s017.zip › Data S1/ENSMUSG00000026566.jpeg]

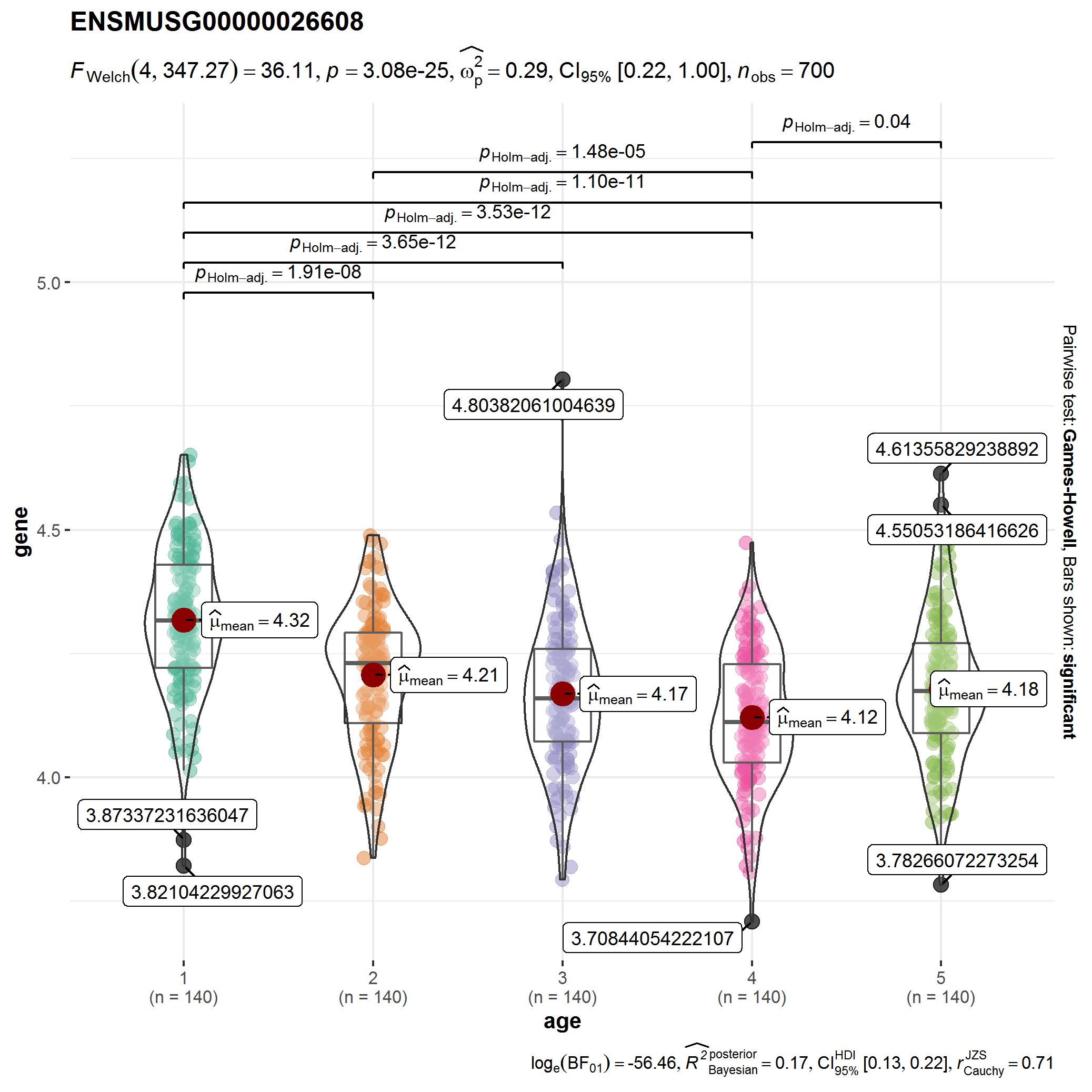

Supplement: Supplementary file 25 — Data S1–S6. [file ACEL-23-e14268-s017.zip › Data S1/ENSMUSG00000026608.jpeg]

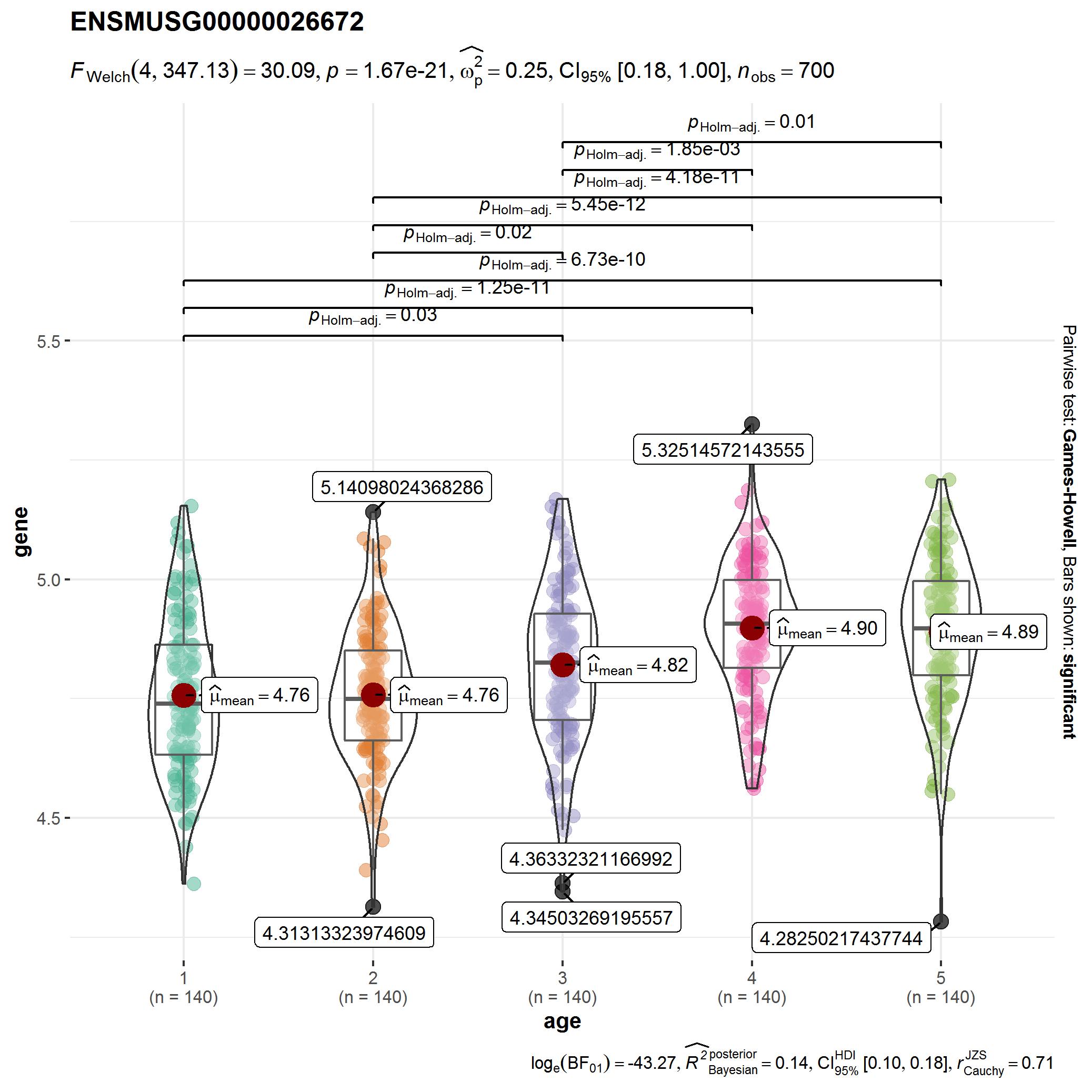

Supplement: Supplementary file 25 — Data S1–S6. [file ACEL-23-e14268-s017.zip › Data S1/ENSMUSG00000026672.jpeg]

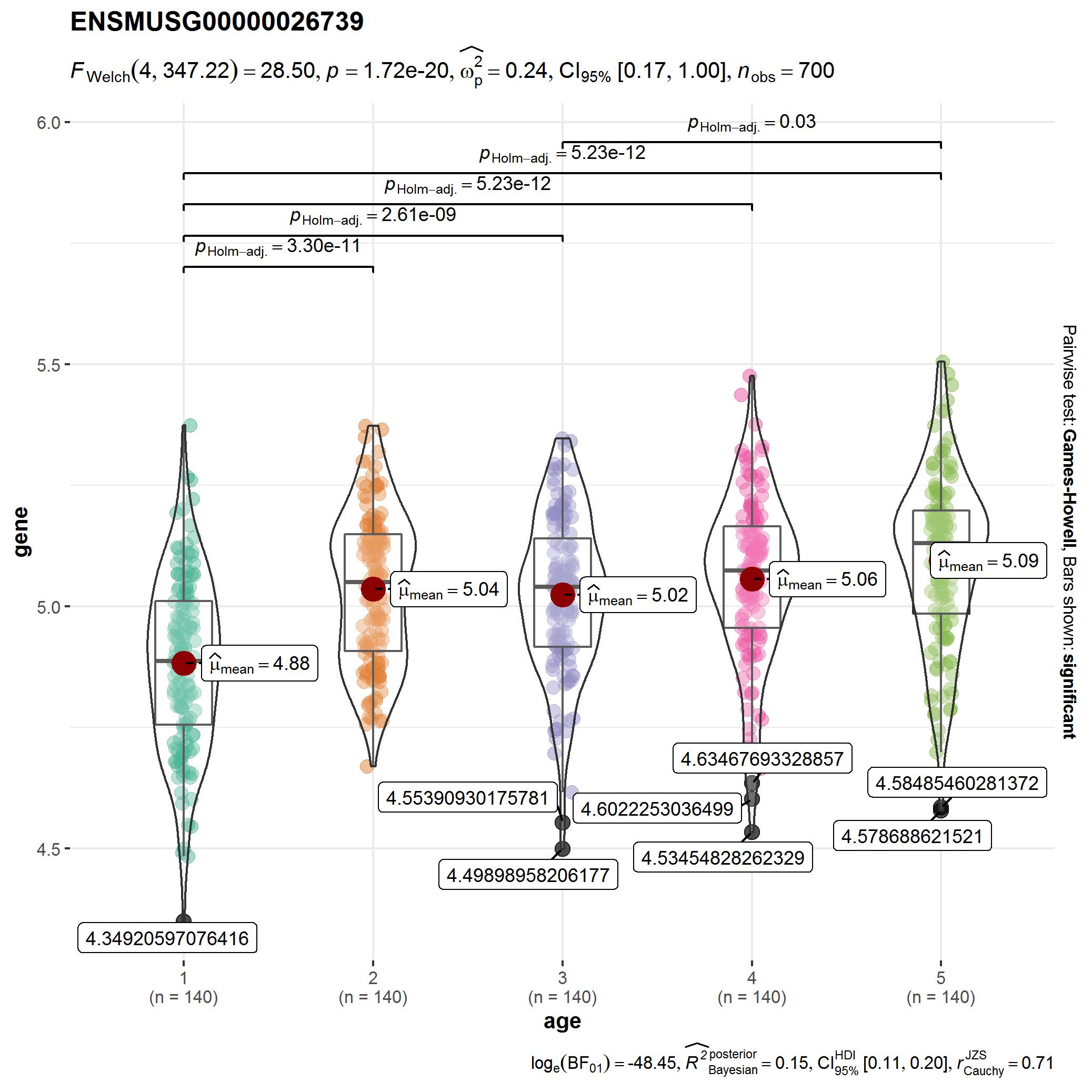

Supplement: Supplementary file 25 — Data S1–S6. [file ACEL-23-e14268-s017.zip › Data S1/ENSMUSG00000026739.jpeg]

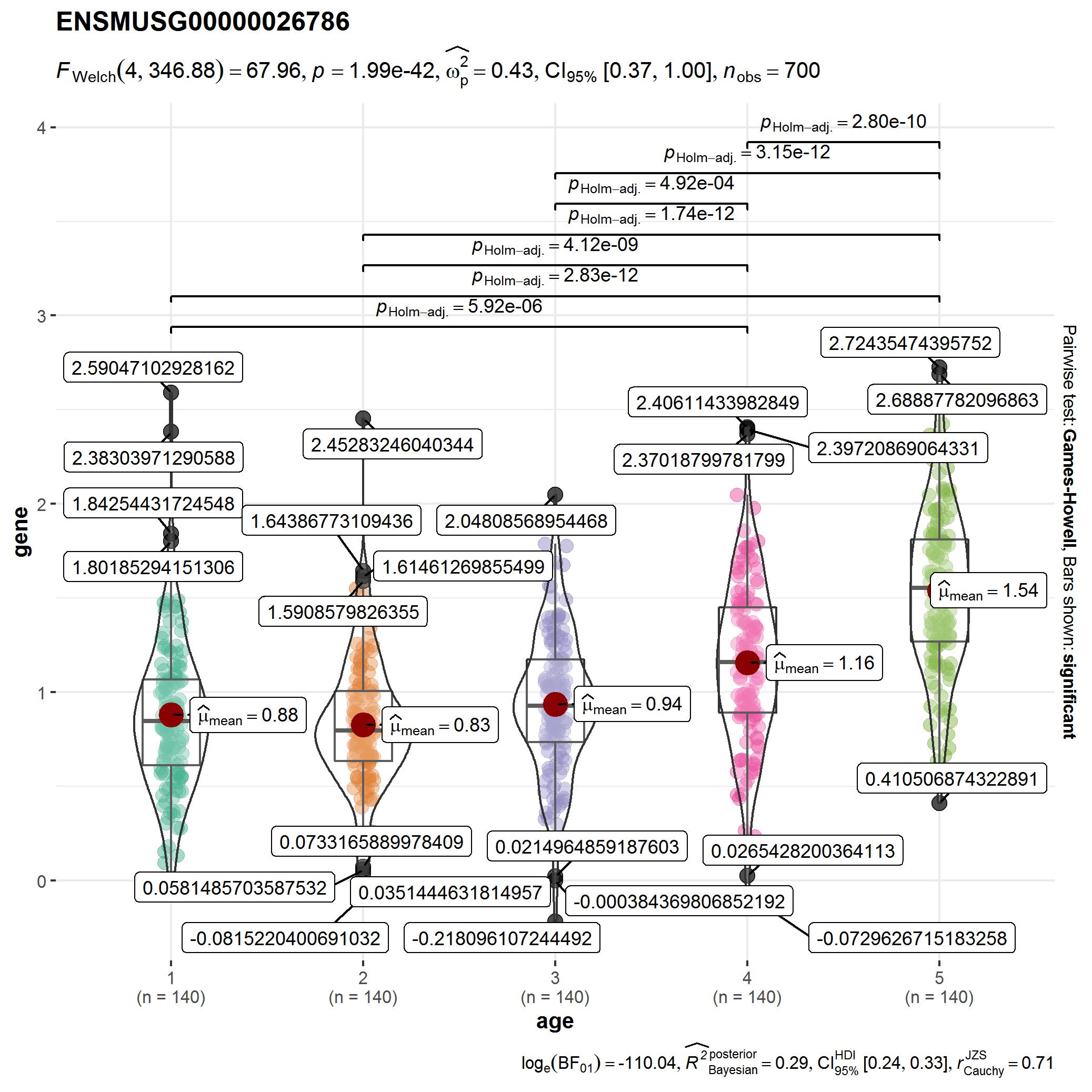

Supplement: Supplementary file 25 — Data S1–S6. [file ACEL-23-e14268-s017.zip › Data S1/ENSMUSG00000026786.jpeg]

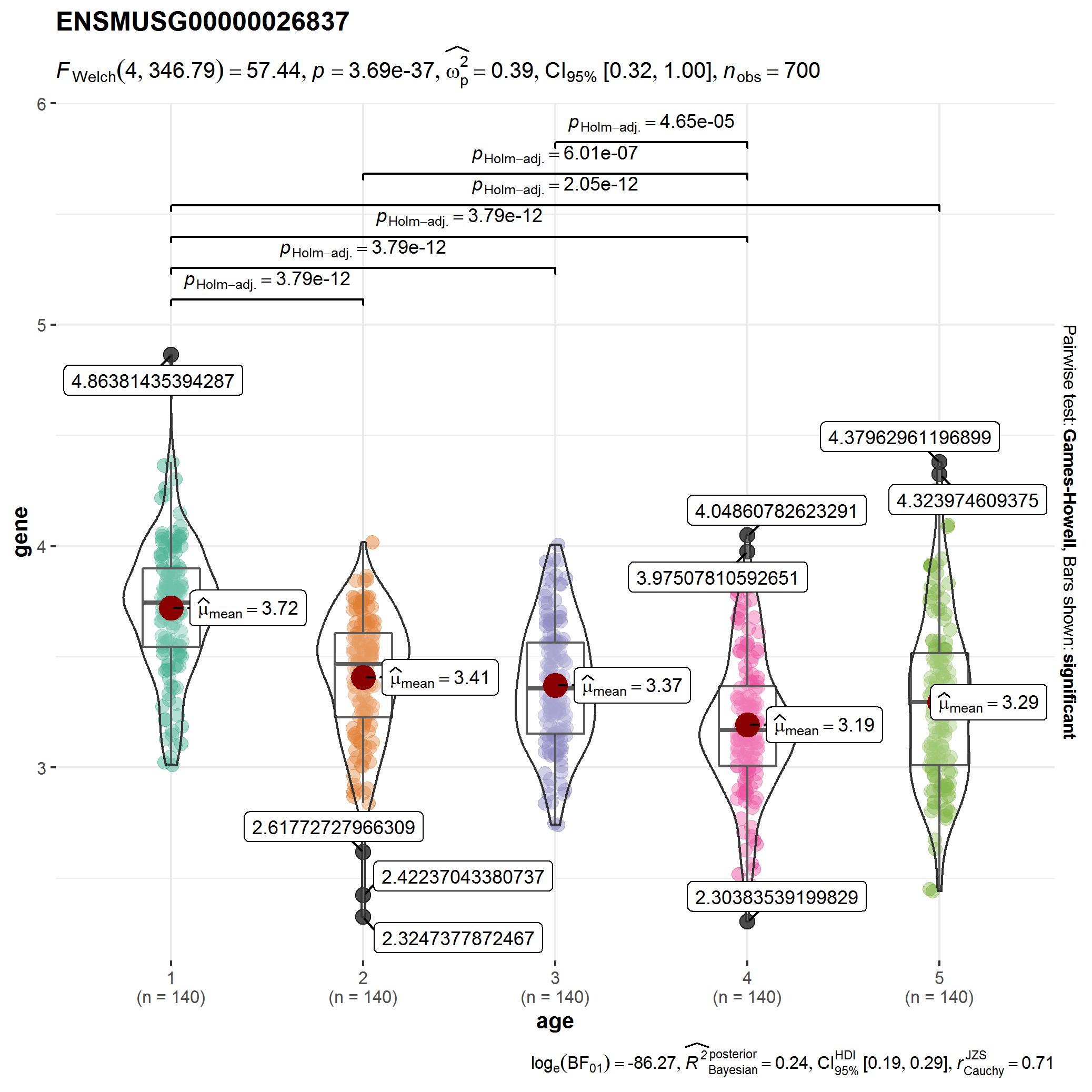

Supplement: Supplementary file 25 — Data S1–S6. [file ACEL-23-e14268-s017.zip › Data S1/ENSMUSG00000026837.jpeg]

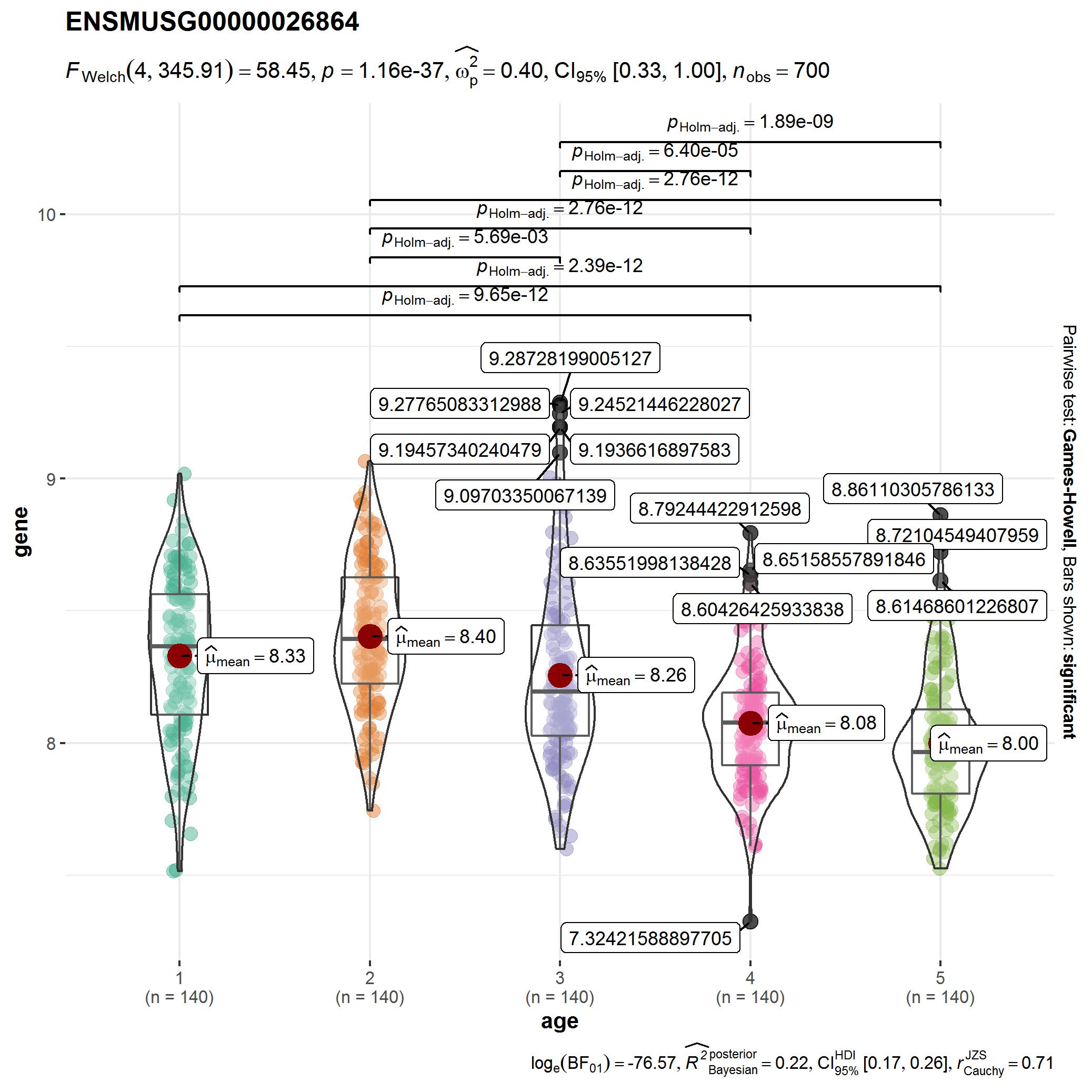

Supplement: Supplementary file 25 — Data S1–S6. [file ACEL-23-e14268-s017.zip › Data S1/ENSMUSG00000026864.jpeg]

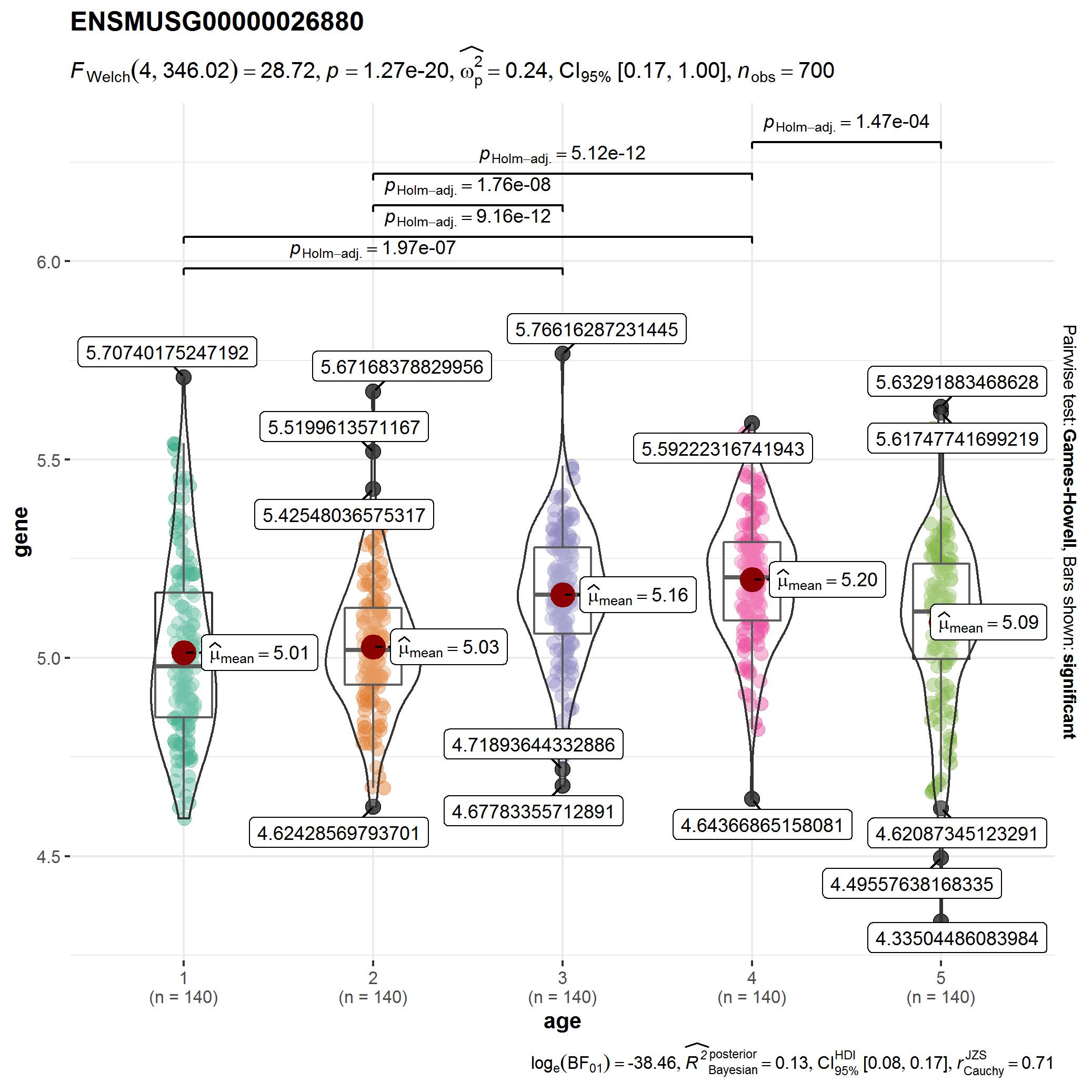

Supplement: Supplementary file 25 — Data S1–S6. [file ACEL-23-e14268-s017.zip › Data S1/ENSMUSG00000026880.jpeg]

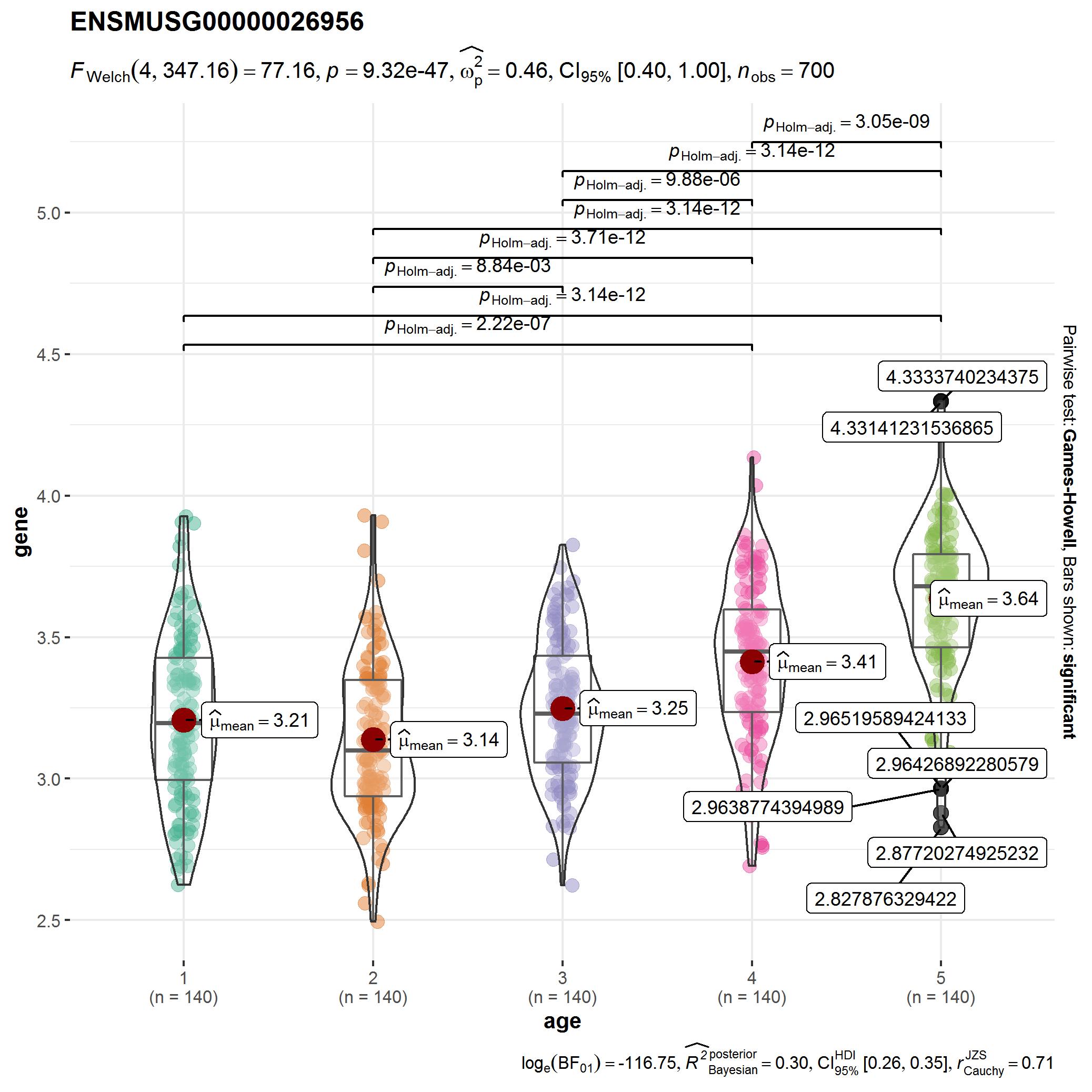

Supplement: Supplementary file 25 — Data S1–S6. [file ACEL-23-e14268-s017.zip › Data S1/ENSMUSG00000026956.jpeg]

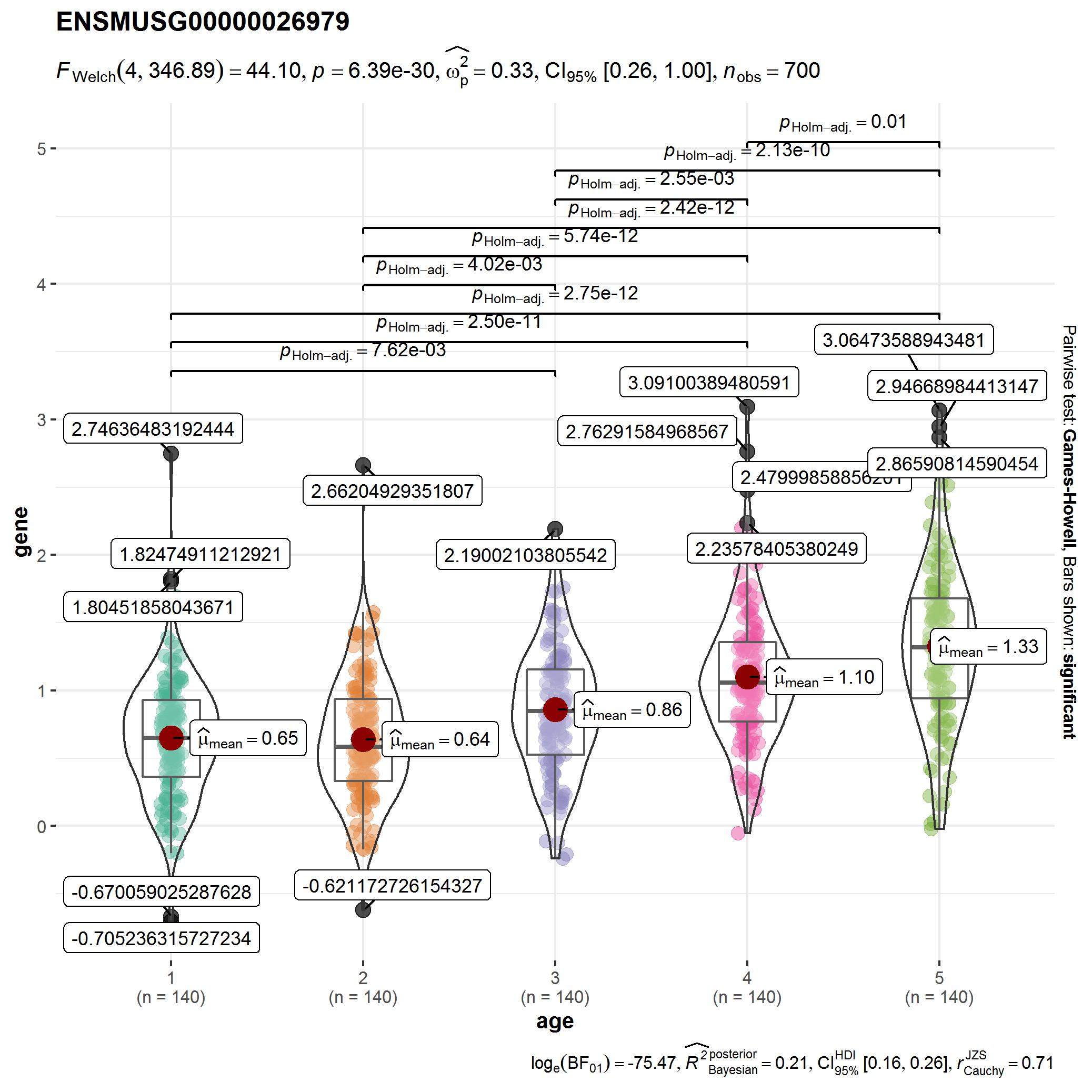

Supplement: Supplementary file 25 — Data S1–S6. [file ACEL-23-e14268-s017.zip › Data S1/ENSMUSG00000026979.jpeg]

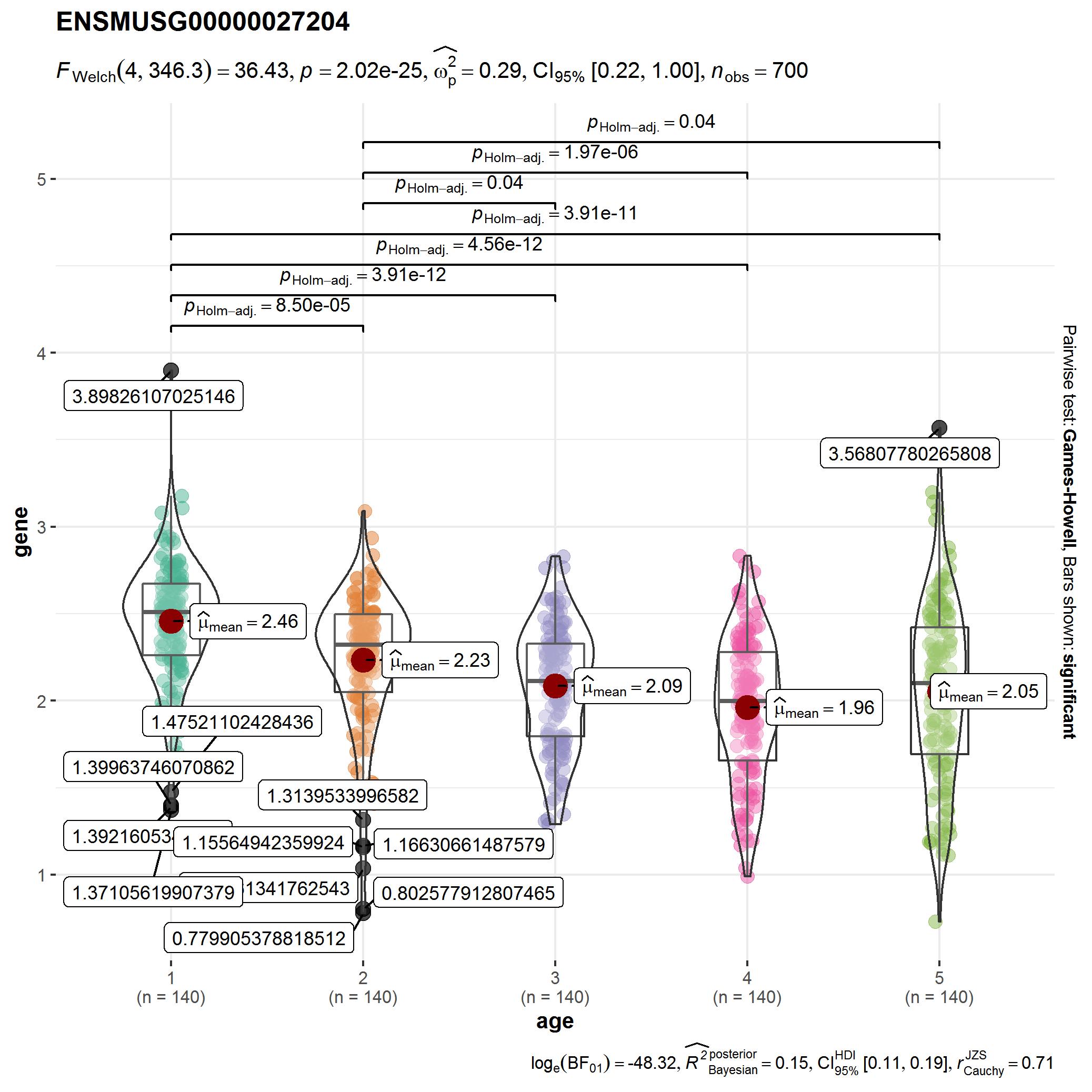

Supplement: Supplementary file 25 — Data S1–S6. [file ACEL-23-e14268-s017.zip › Data S1/ENSMUSG00000027204.jpeg]

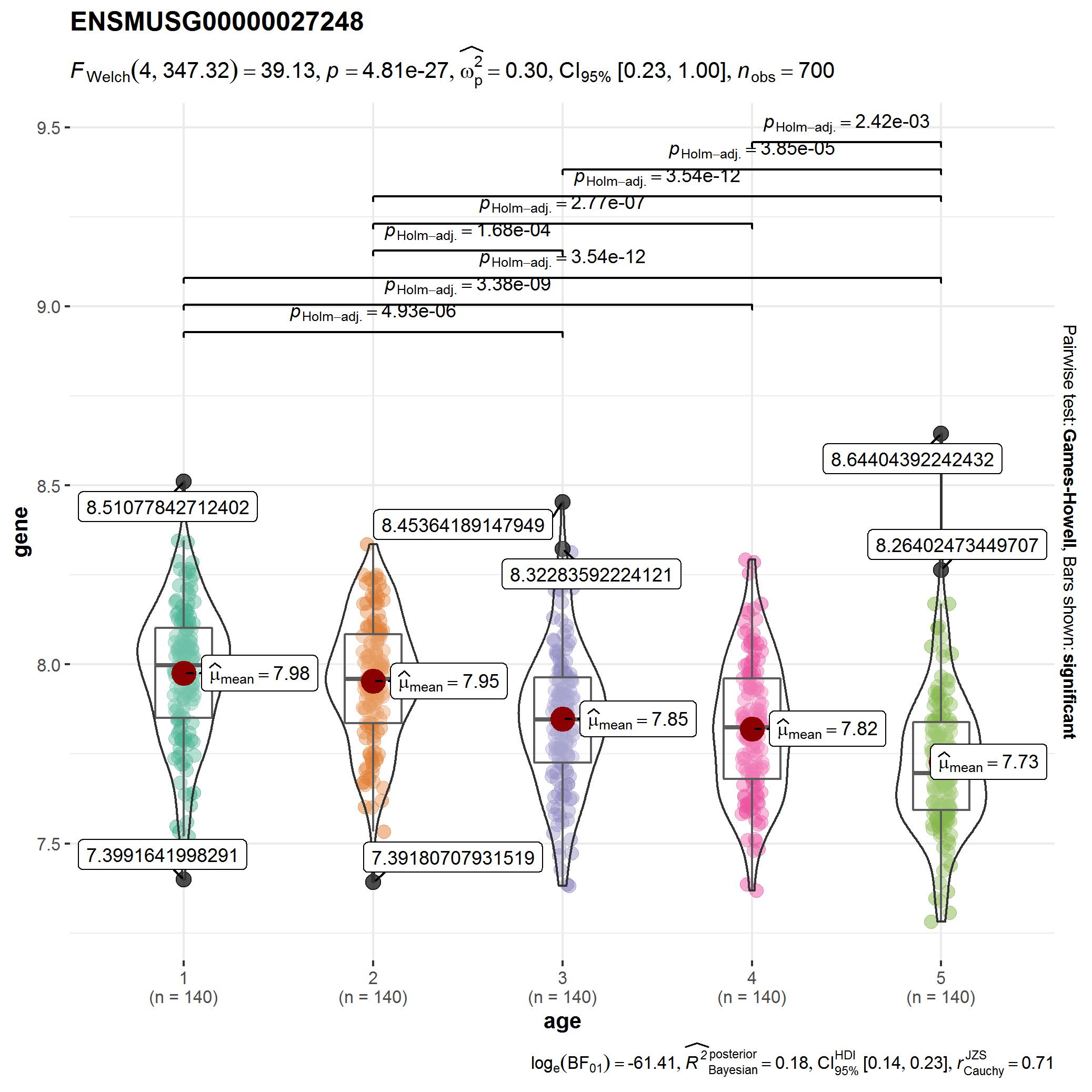

Supplement: Supplementary file 25 — Data S1–S6. [file ACEL-23-e14268-s017.zip › Data S1/ENSMUSG00000027248.jpeg]

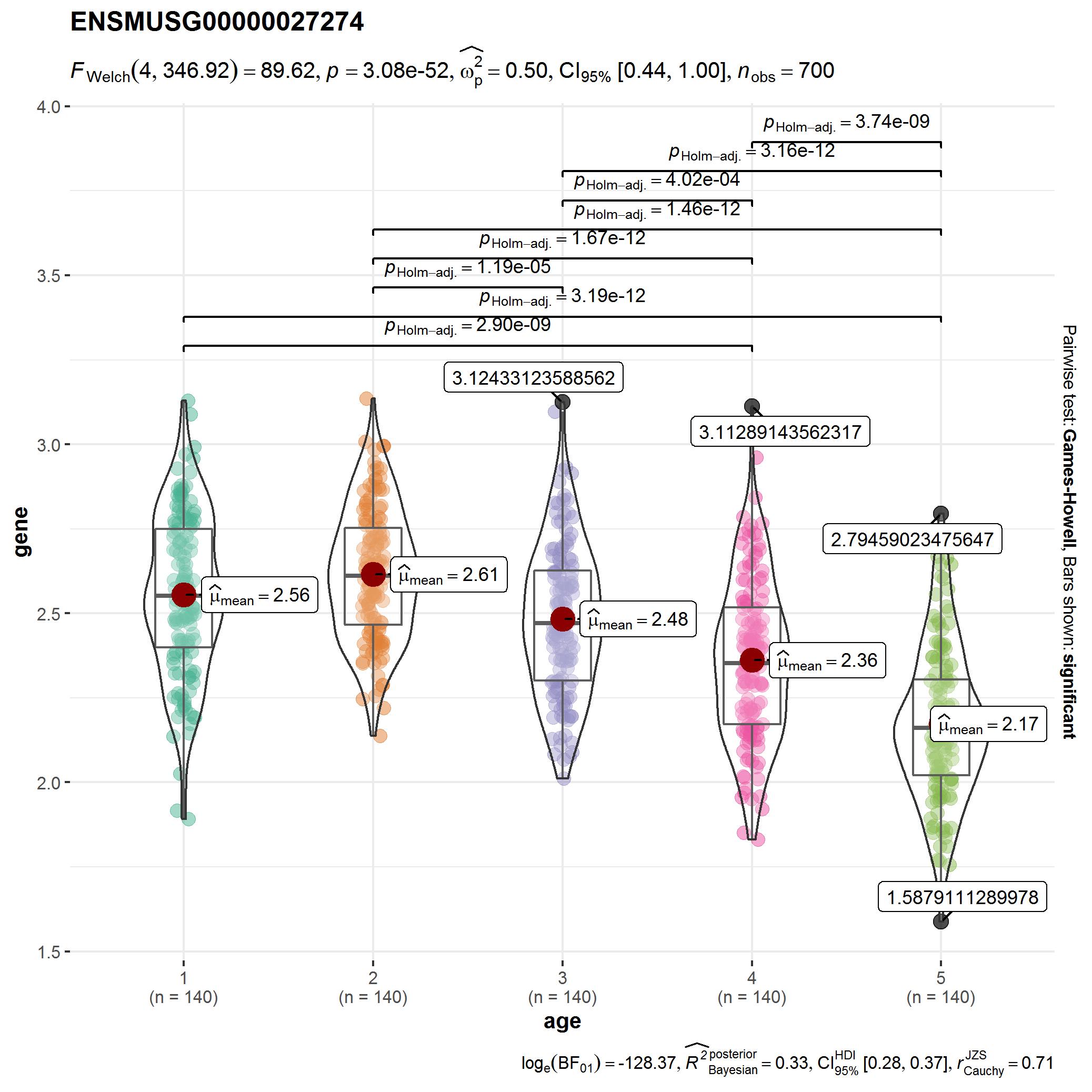

Supplement: Supplementary file 25 — Data S1–S6. [file ACEL-23-e14268-s017.zip › Data S1/ENSMUSG00000027274.jpeg]

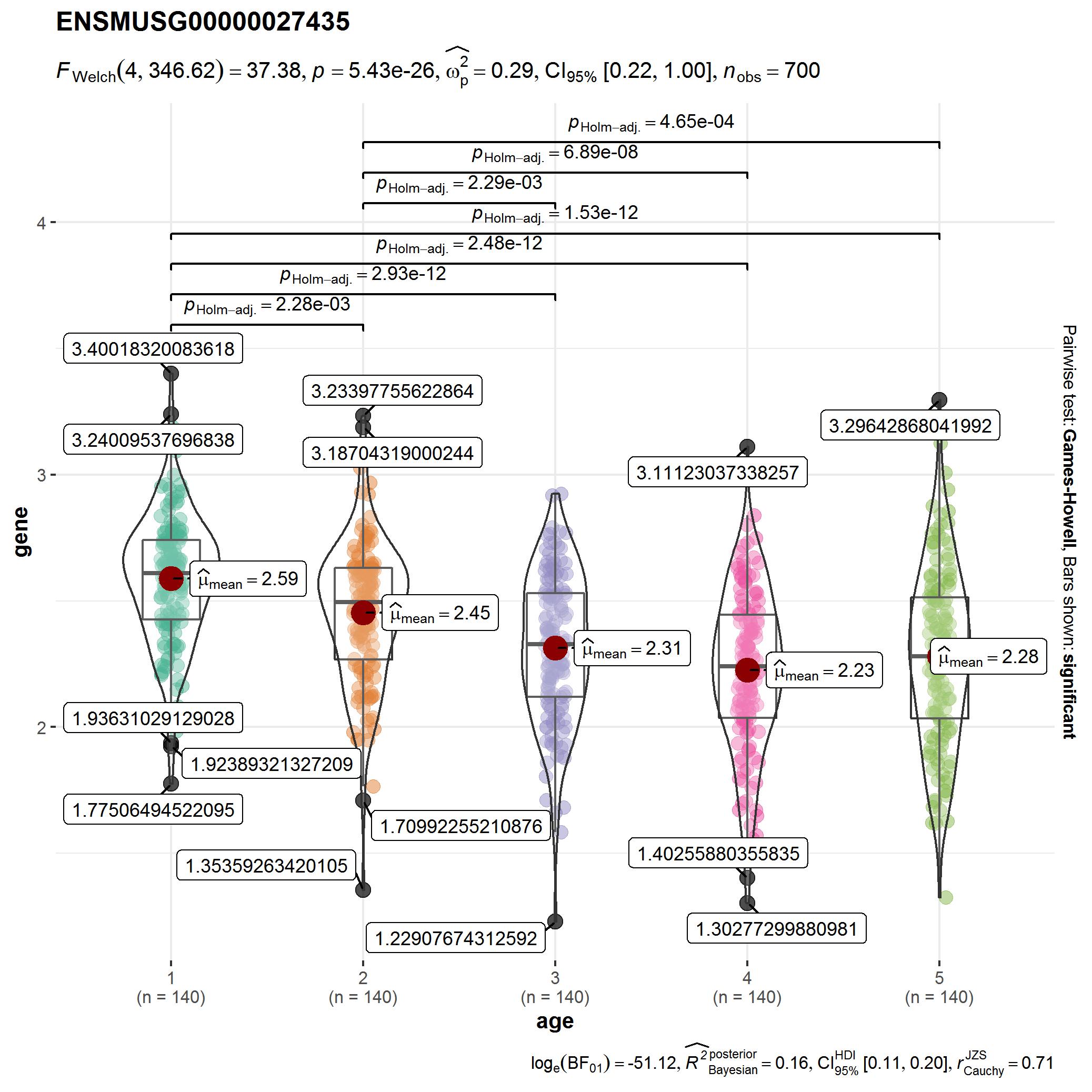

Supplement: Supplementary file 25 — Data S1–S6. [file ACEL-23-e14268-s017.zip › Data S1/ENSMUSG00000027435.jpeg]

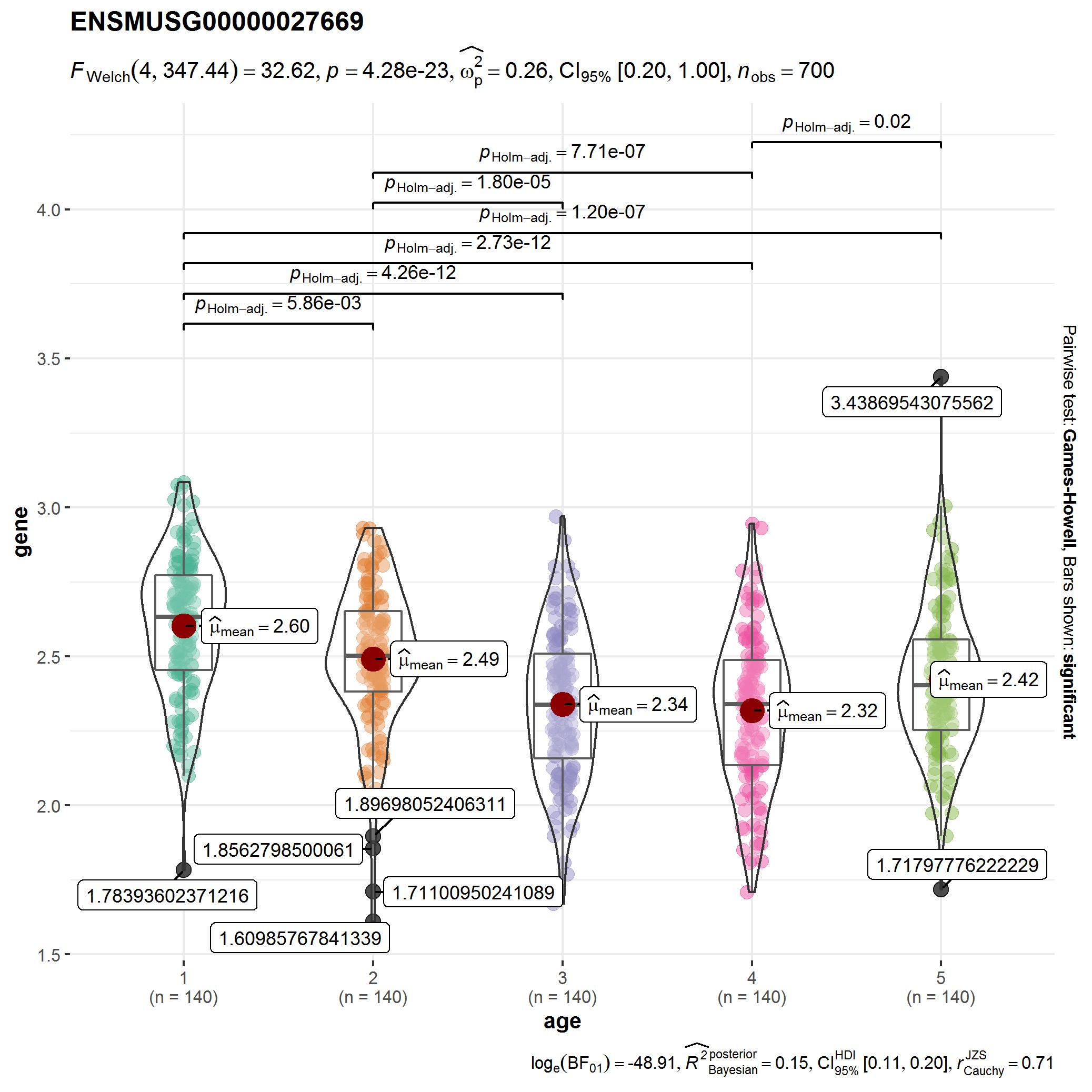

Supplement: Supplementary file 25 — Data S1–S6. [file ACEL-23-e14268-s017.zip › Data S1/ENSMUSG00000027669.jpeg]

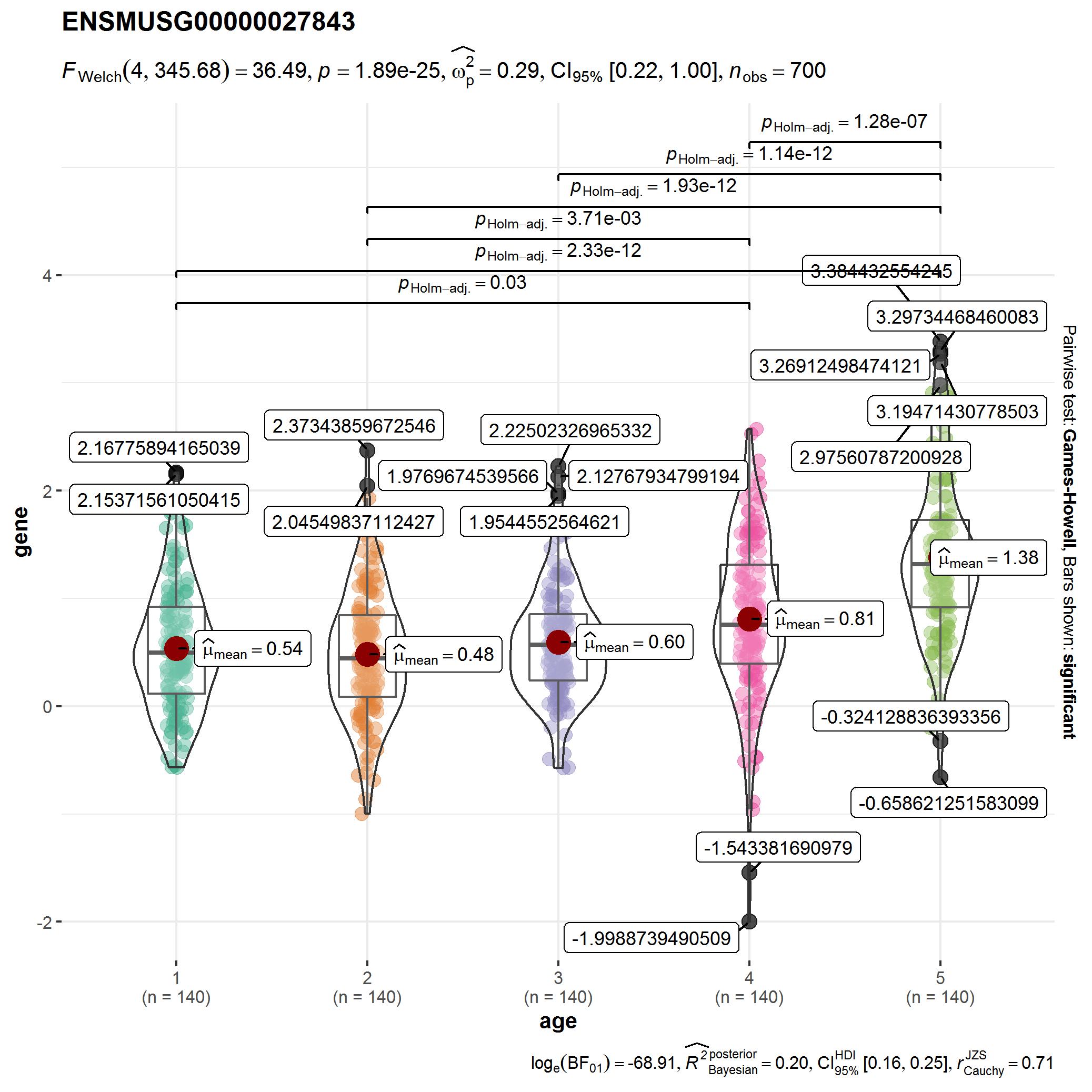

Supplement: Supplementary file 25 — Data S1–S6. [file ACEL-23-e14268-s017.zip › Data S1/ENSMUSG00000027843.jpeg]

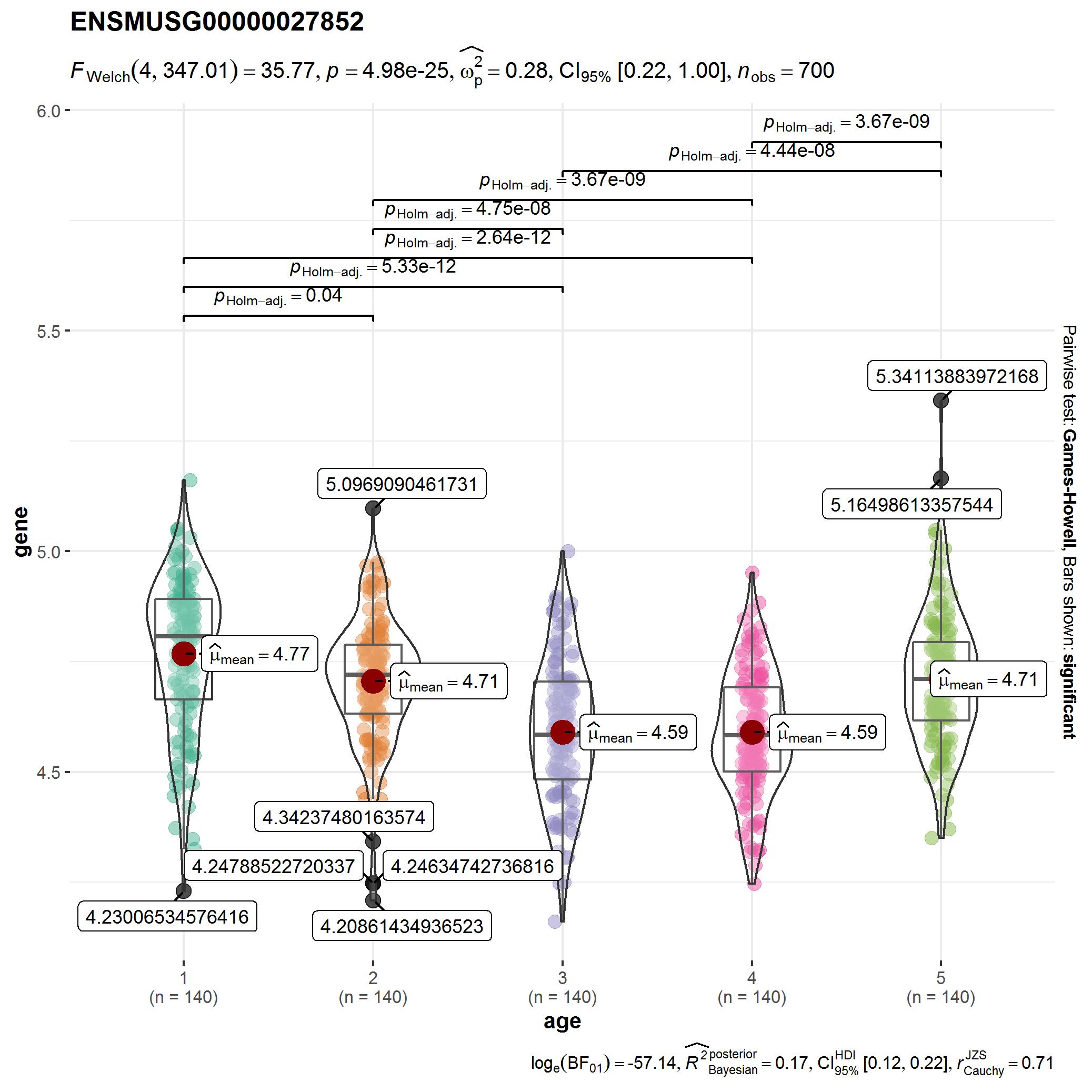

Supplement: Supplementary file 25 — Data S1–S6. [file ACEL-23-e14268-s017.zip › Data S1/ENSMUSG00000027852.jpeg]

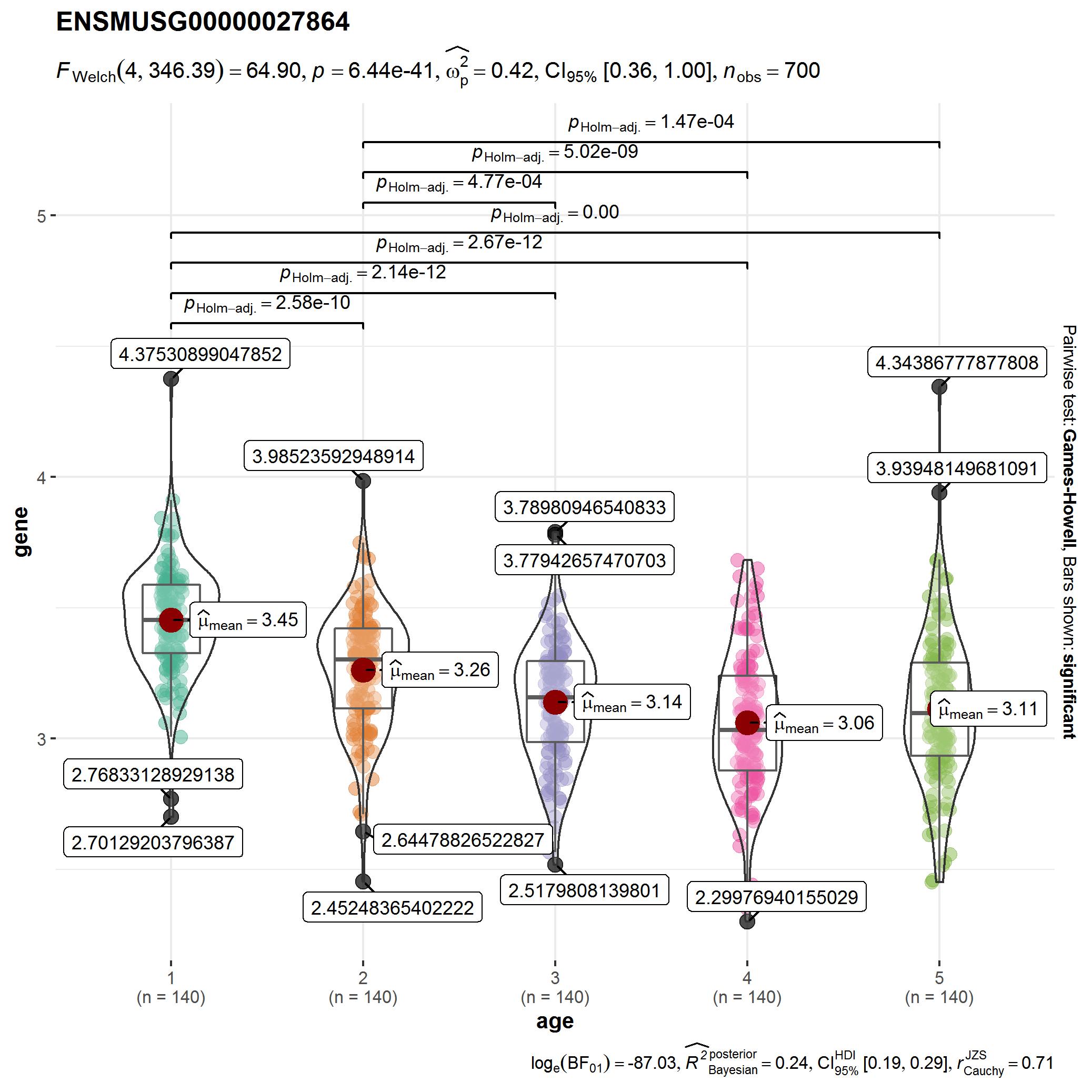

Supplement: Supplementary file 25 — Data S1–S6. [file ACEL-23-e14268-s017.zip › Data S1/ENSMUSG00000027864.jpeg]

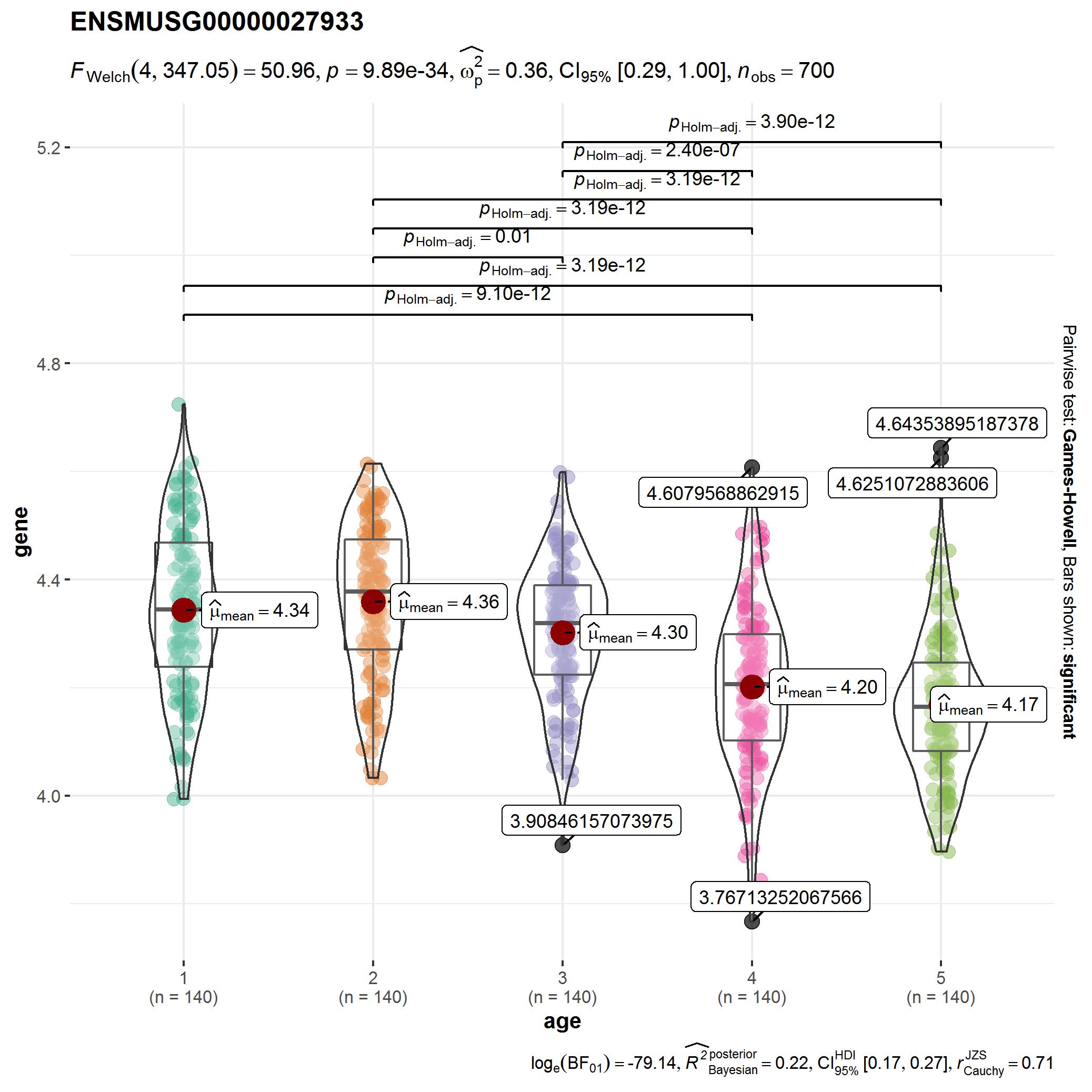

Supplement: Supplementary file 25 — Data S1–S6. [file ACEL-23-e14268-s017.zip › Data S1/ENSMUSG00000027933.jpeg]

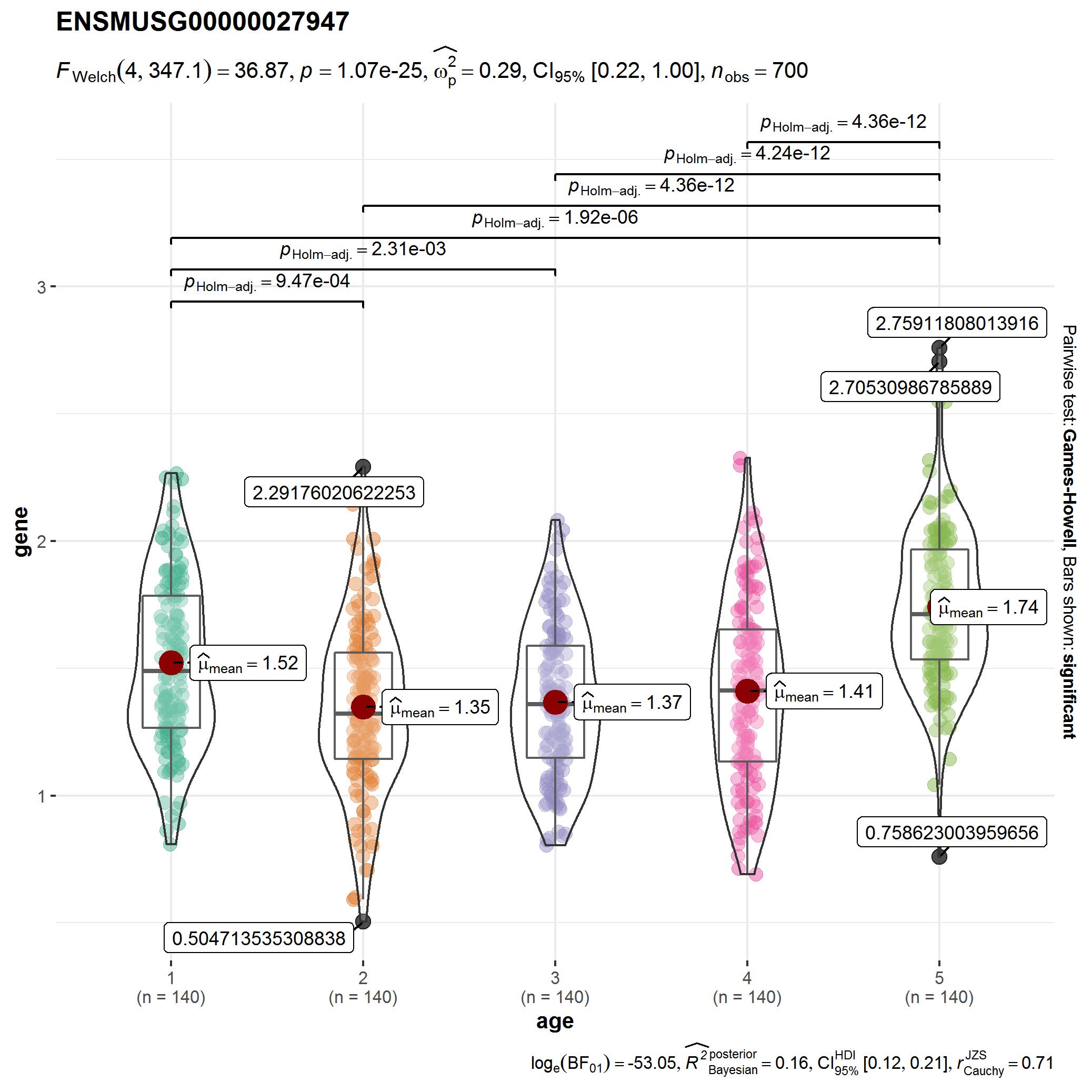

Supplement: Supplementary file 25 — Data S1–S6. [file ACEL-23-e14268-s017.zip › Data S1/ENSMUSG00000027947.jpeg]

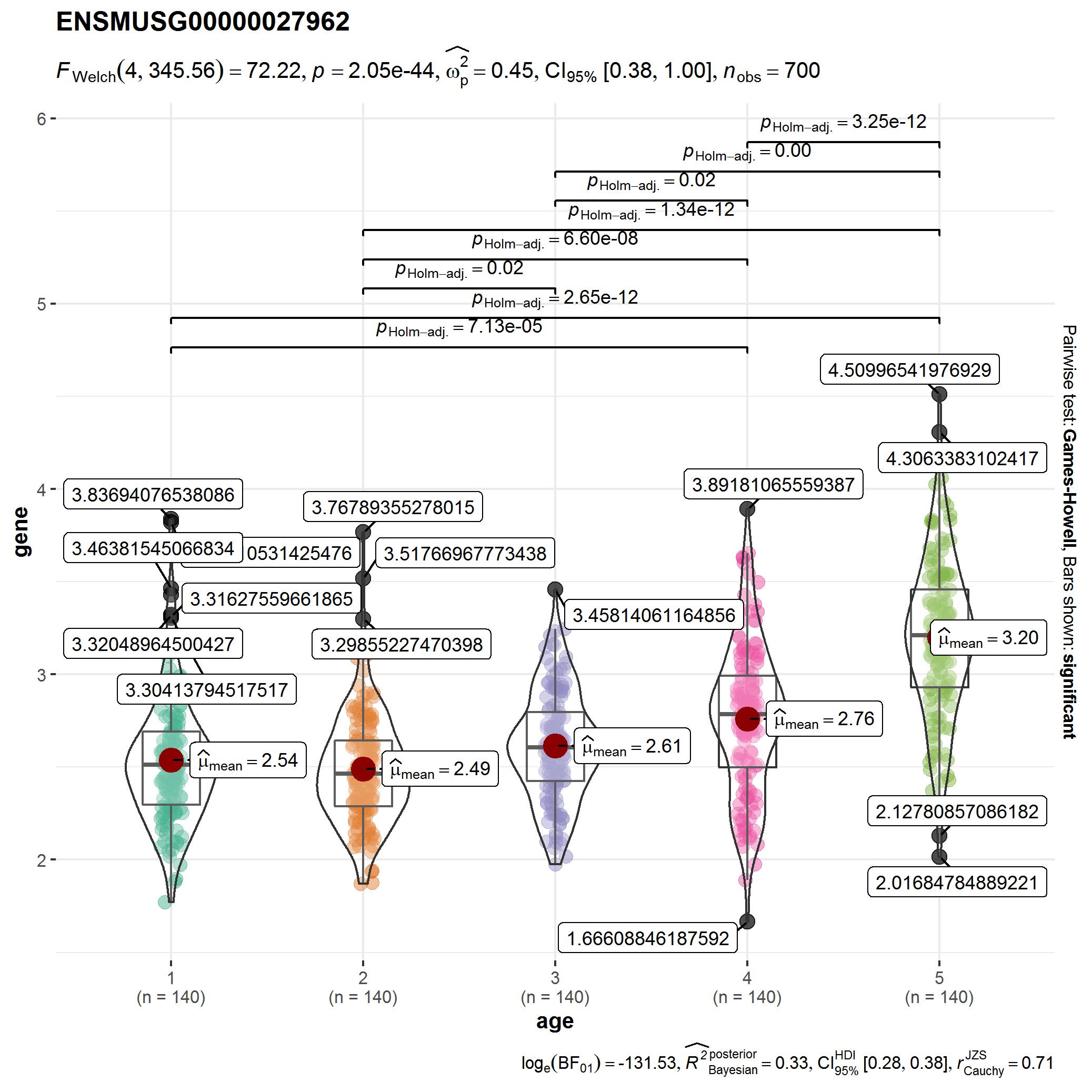

Supplement: Supplementary file 25 — Data S1–S6. [file ACEL-23-e14268-s017.zip › Data S1/ENSMUSG00000027962.jpeg]

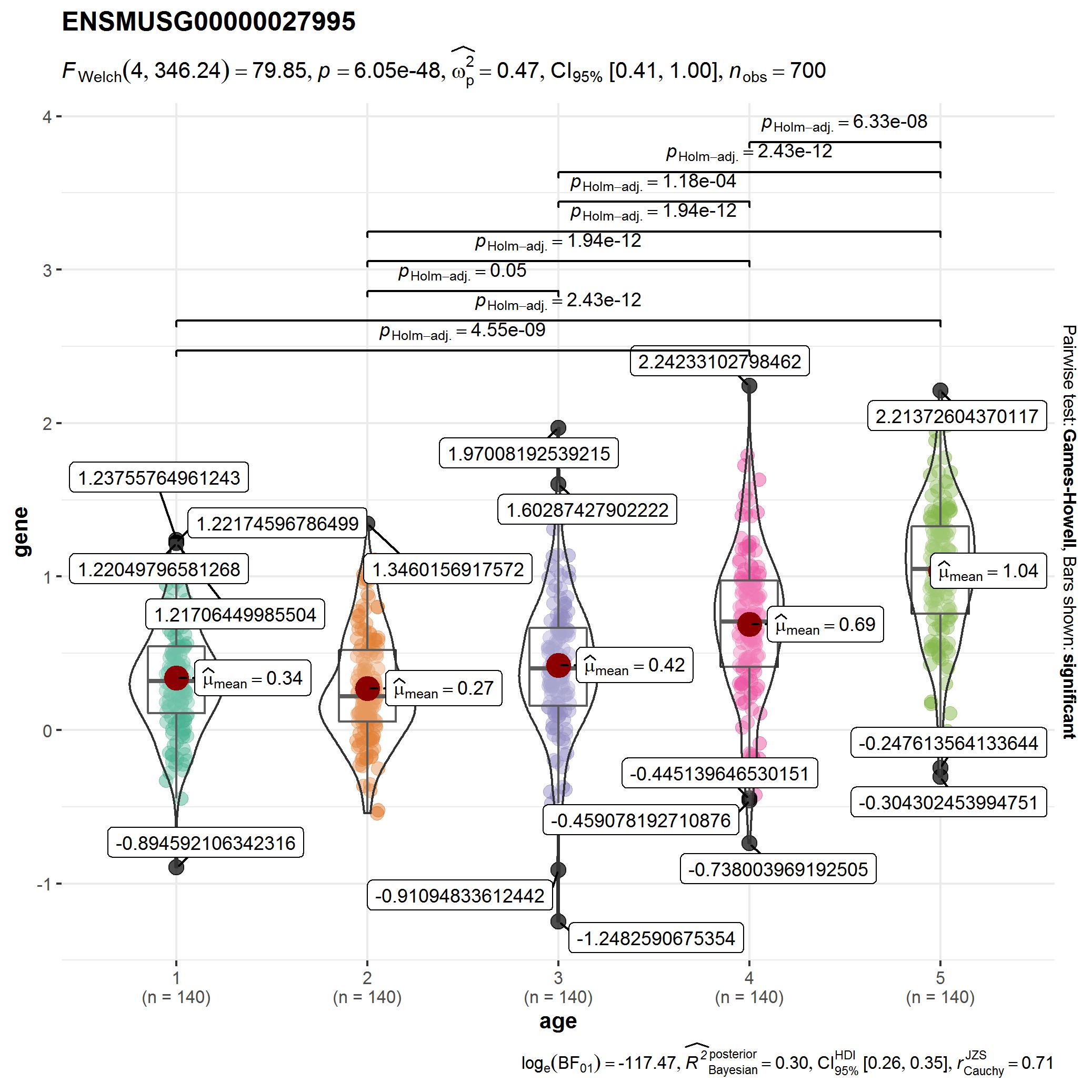

Supplement: Supplementary file 25 — Data S1–S6. [file ACEL-23-e14268-s017.zip › Data S1/ENSMUSG00000027995.jpeg]

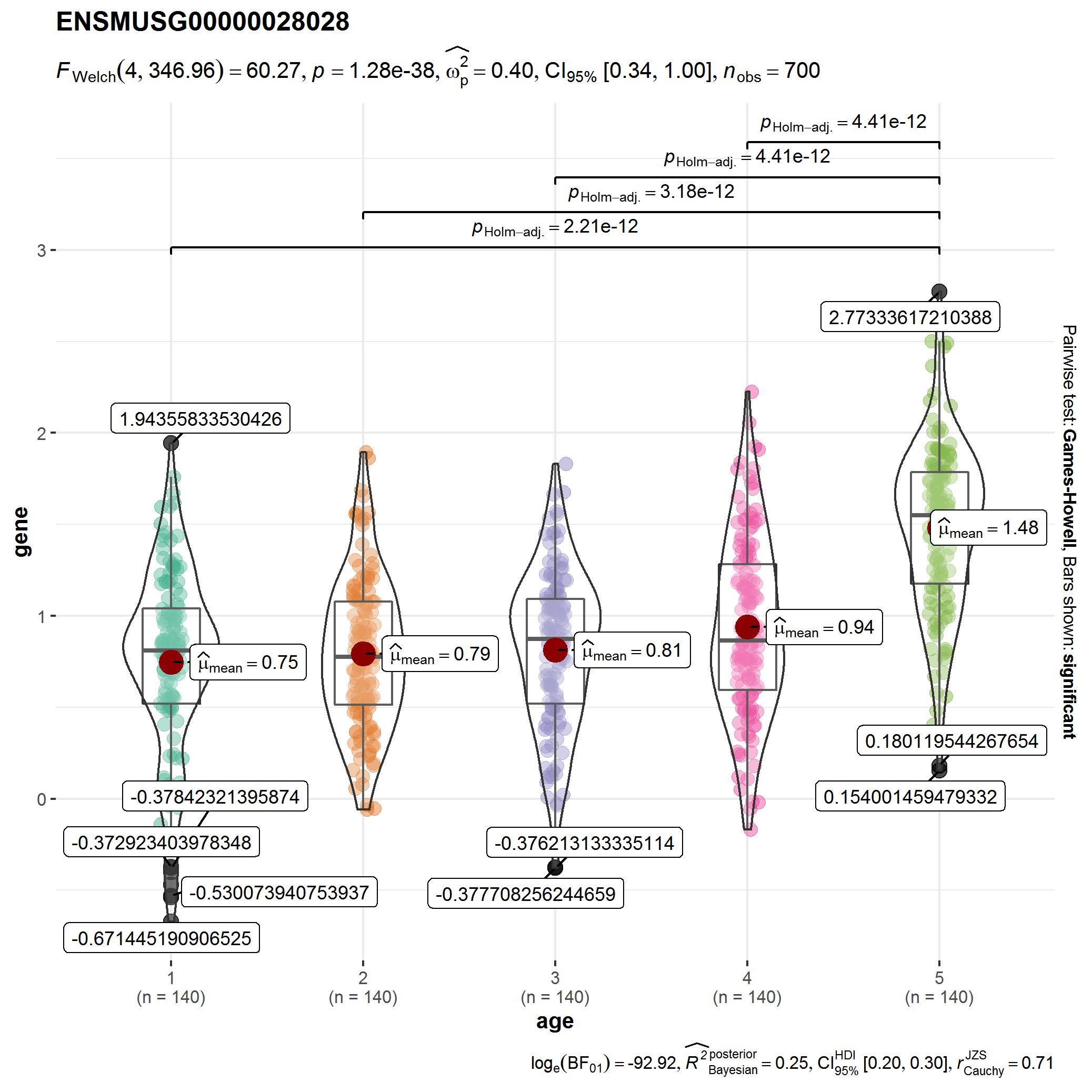

Supplement: Supplementary file 25 — Data S1–S6. [file ACEL-23-e14268-s017.zip › Data S1/ENSMUSG00000028028.jpeg]

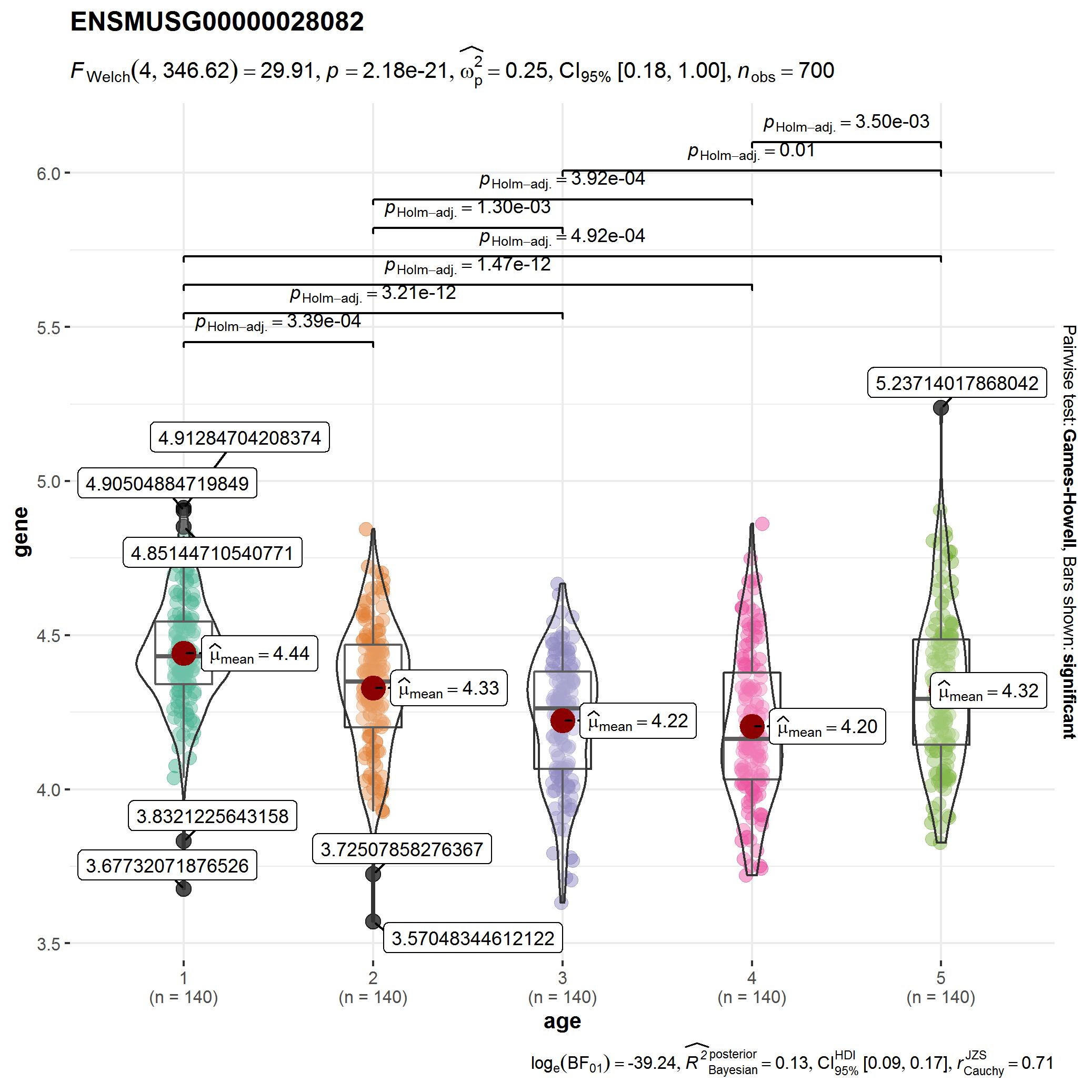

Supplement: Supplementary file 25 — Data S1–S6. [file ACEL-23-e14268-s017.zip › Data S1/ENSMUSG00000028082.jpeg]

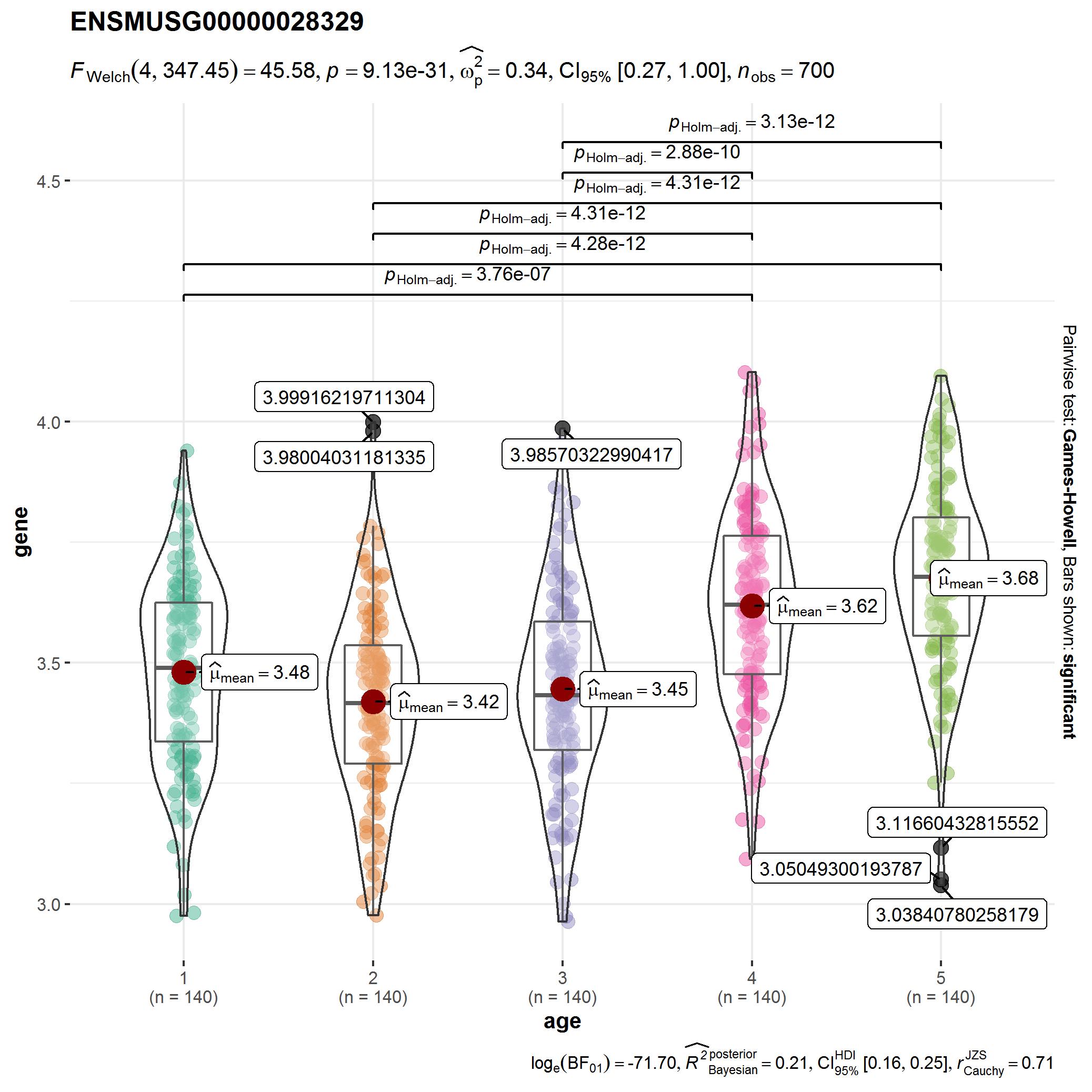

Supplement: Supplementary file 25 — Data S1–S6. [file ACEL-23-e14268-s017.zip › Data S1/ENSMUSG00000028329.jpeg]

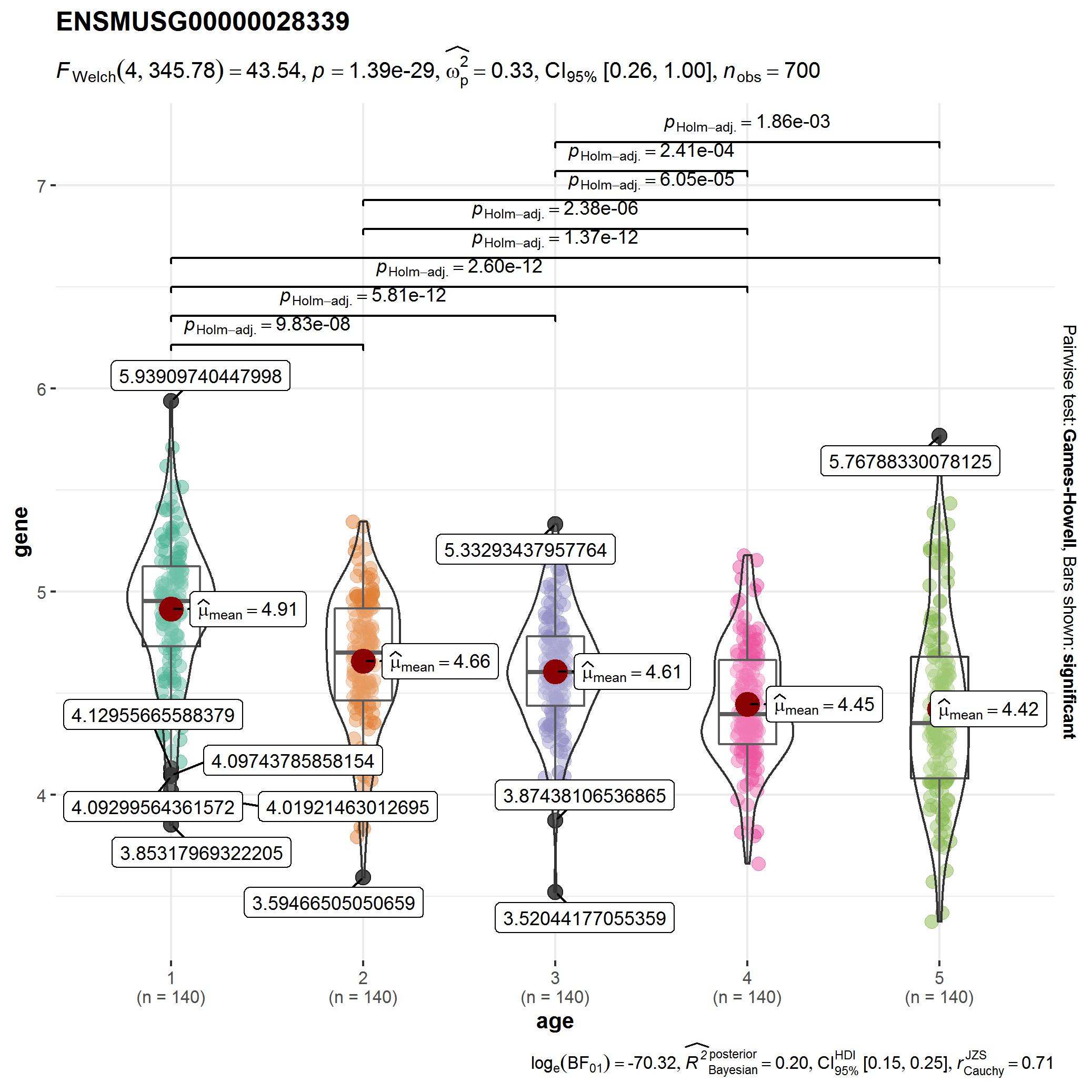

Supplement: Supplementary file 25 — Data S1–S6. [file ACEL-23-e14268-s017.zip › Data S1/ENSMUSG00000028339.jpeg]

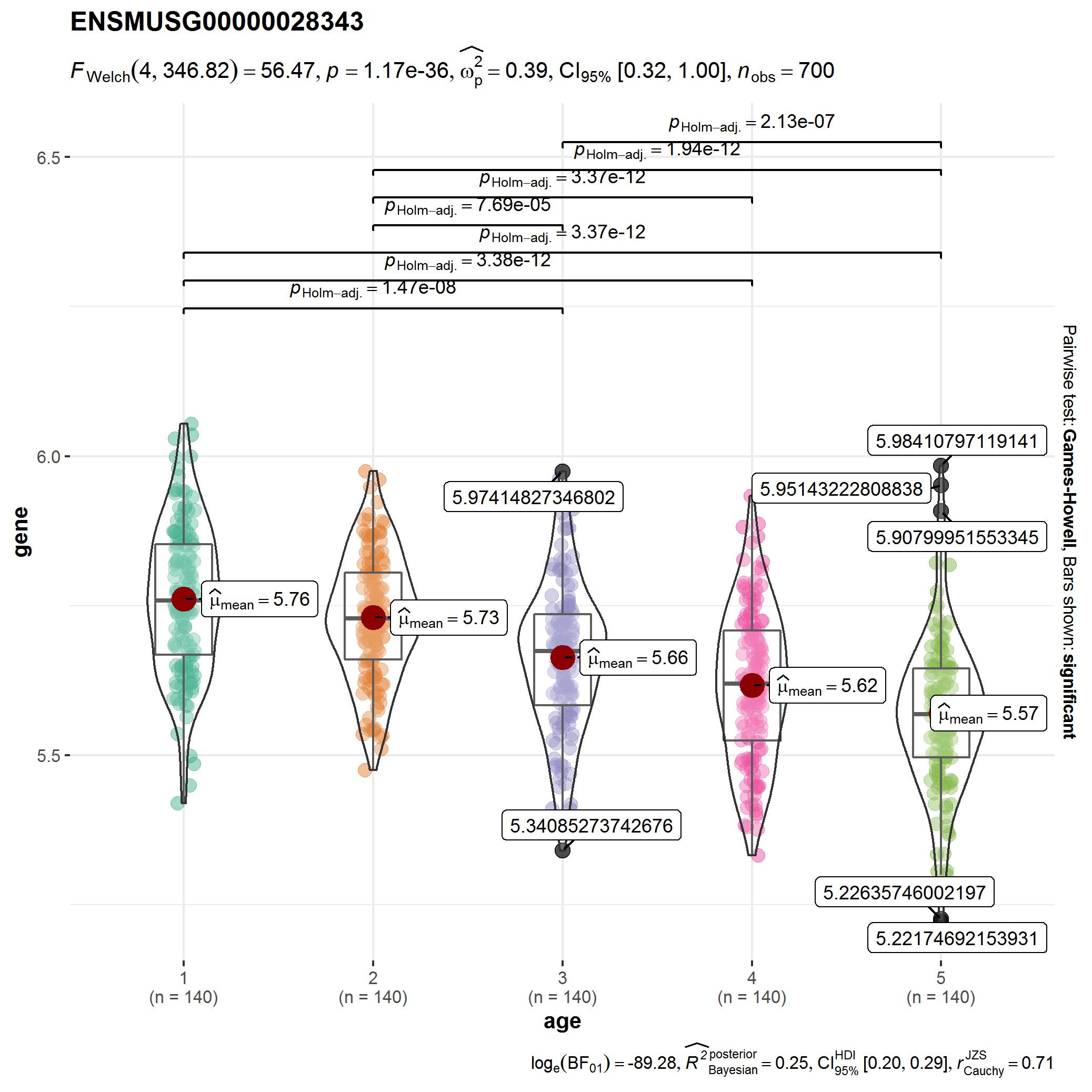

Supplement: Supplementary file 25 — Data S1–S6. [file ACEL-23-e14268-s017.zip › Data S1/ENSMUSG00000028343.jpeg]

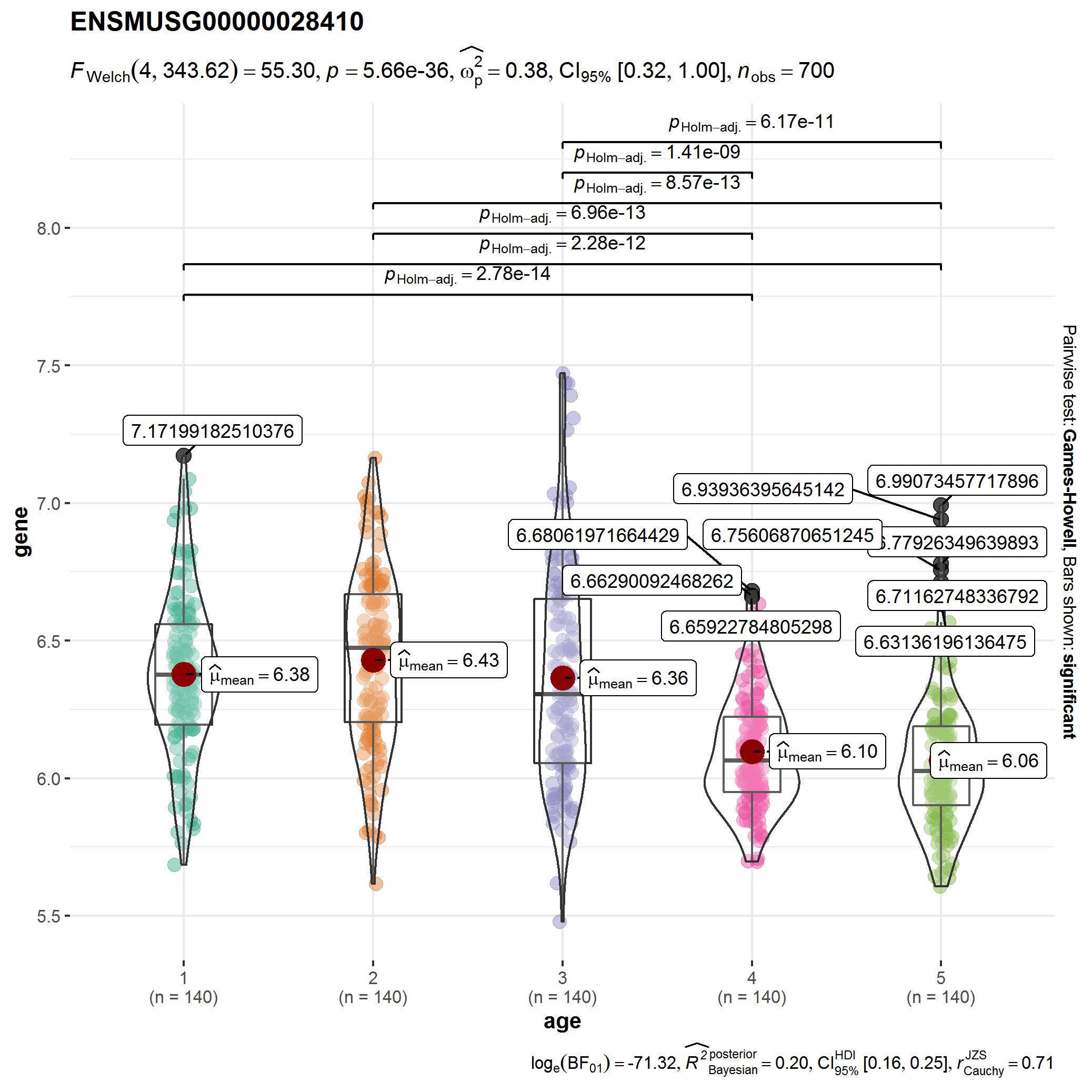

Supplement: Supplementary file 25 — Data S1–S6. [file ACEL-23-e14268-s017.zip › Data S1/ENSMUSG00000028410.jpeg]

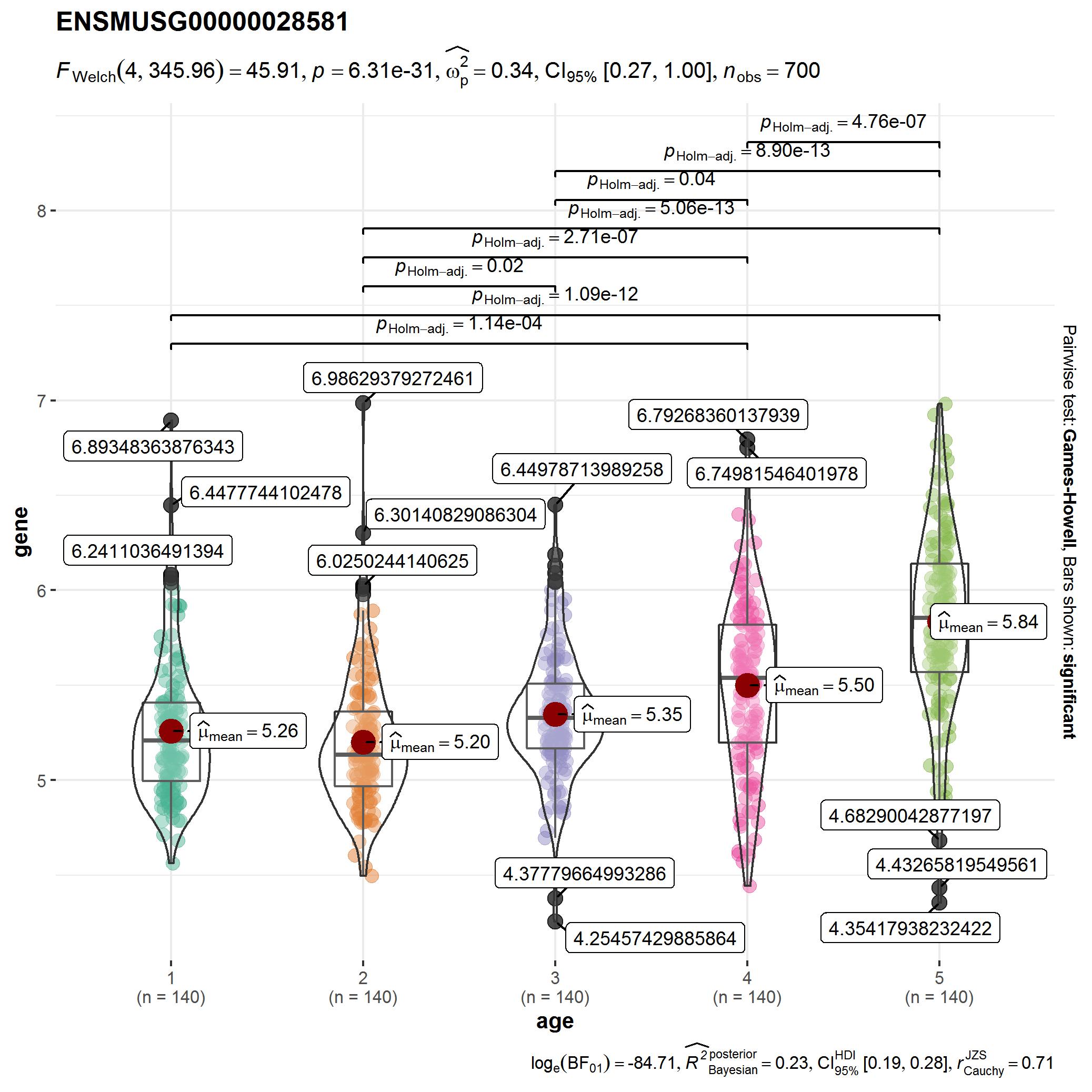

Supplement: Supplementary file 25 — Data S1–S6. [file ACEL-23-e14268-s017.zip › Data S1/ENSMUSG00000028581.jpeg]

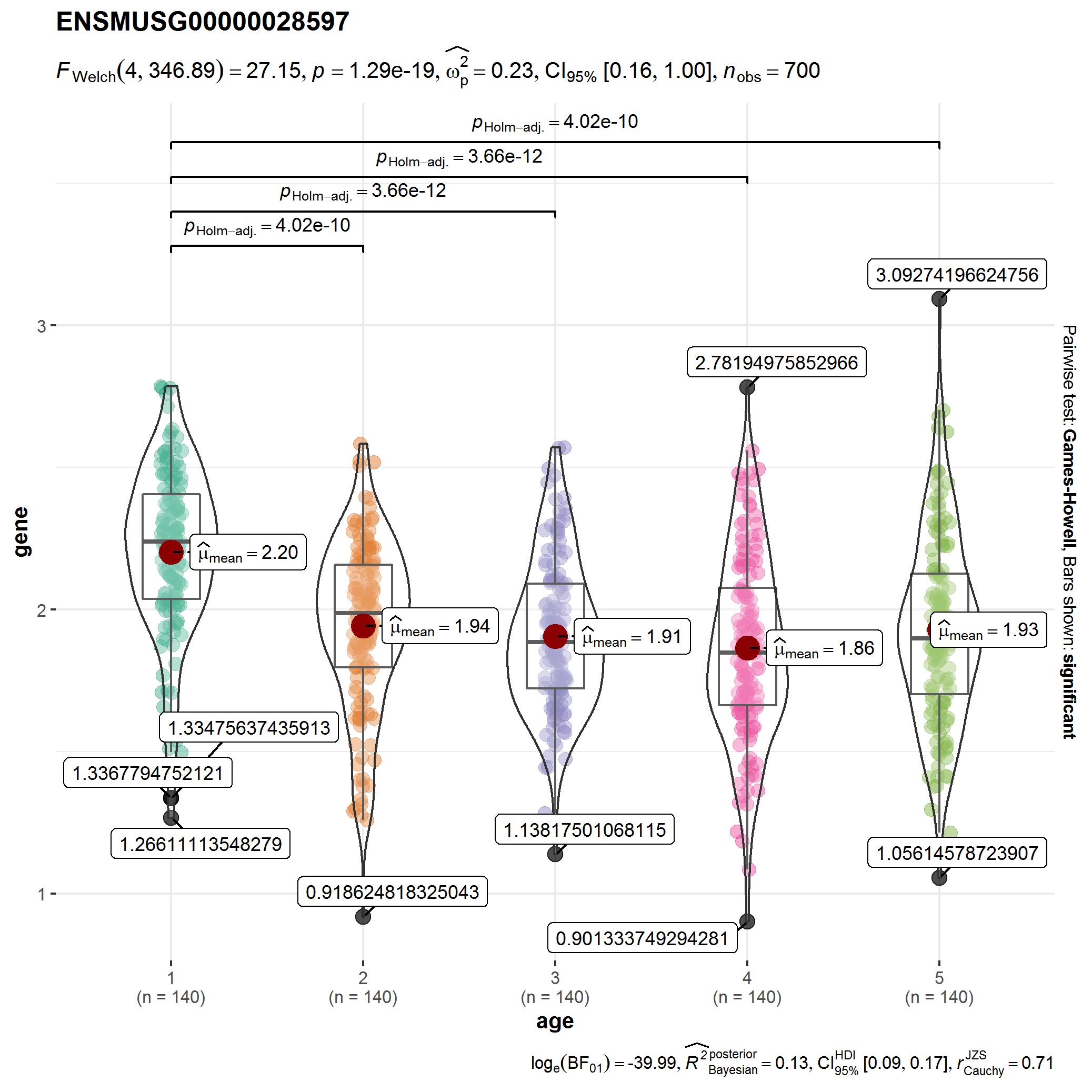

Supplement: Supplementary file 25 — Data S1–S6. [file ACEL-23-e14268-s017.zip › Data S1/ENSMUSG00000028597.jpeg]

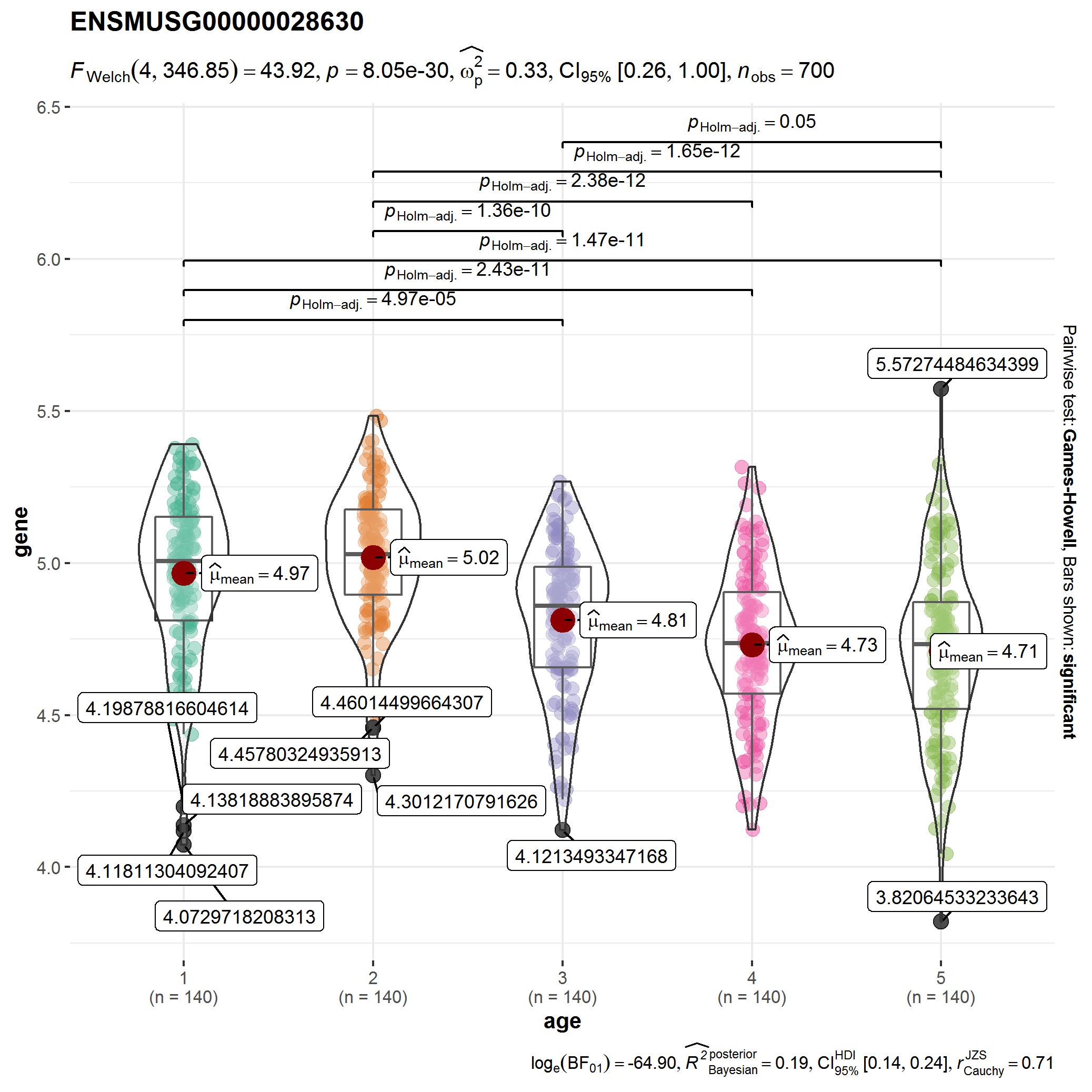

Supplement: Supplementary file 25 — Data S1–S6. [file ACEL-23-e14268-s017.zip › Data S1/ENSMUSG00000028630.jpeg]

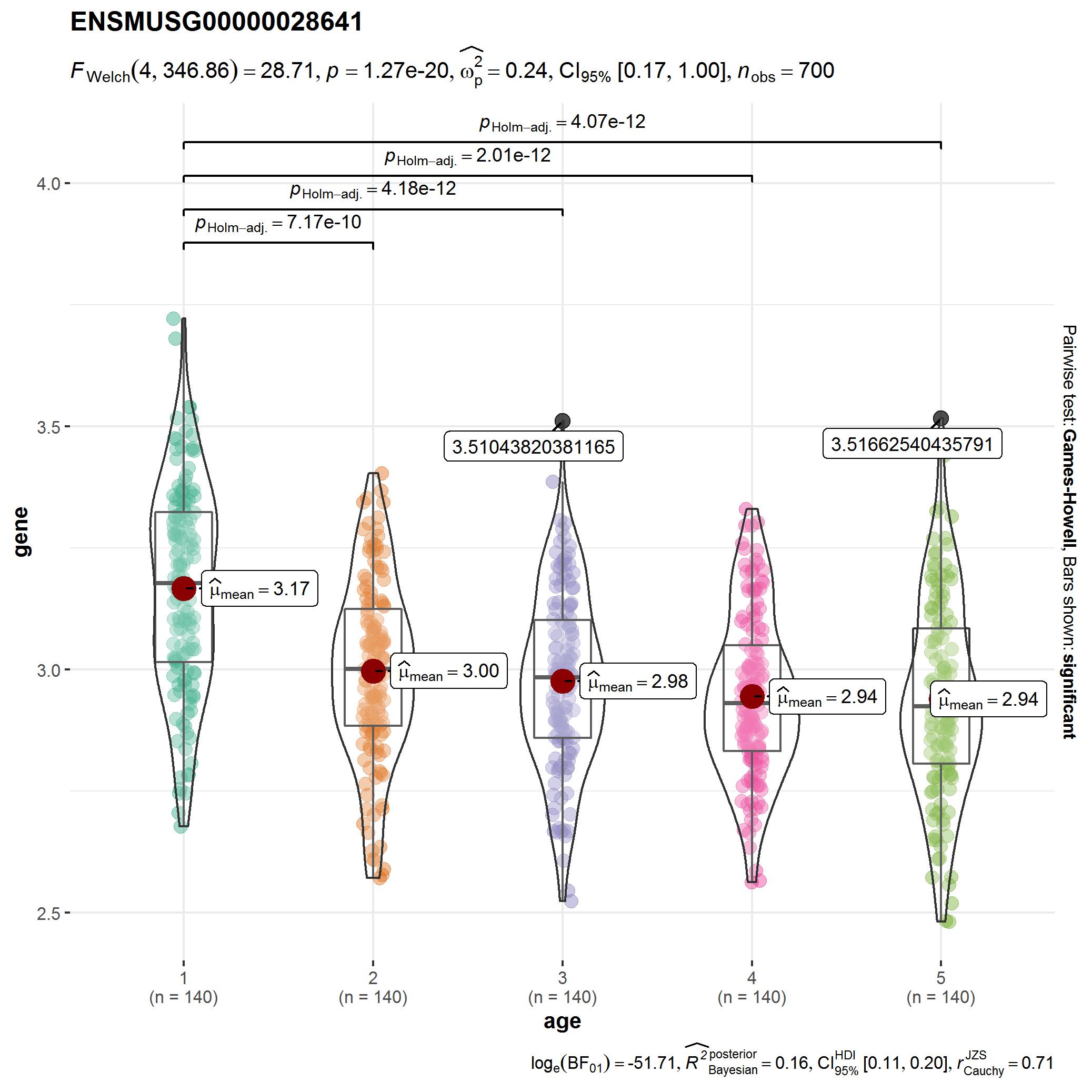

Supplement: Supplementary file 25 — Data S1–S6. [file ACEL-23-e14268-s017.zip › Data S1/ENSMUSG00000028641.jpeg]

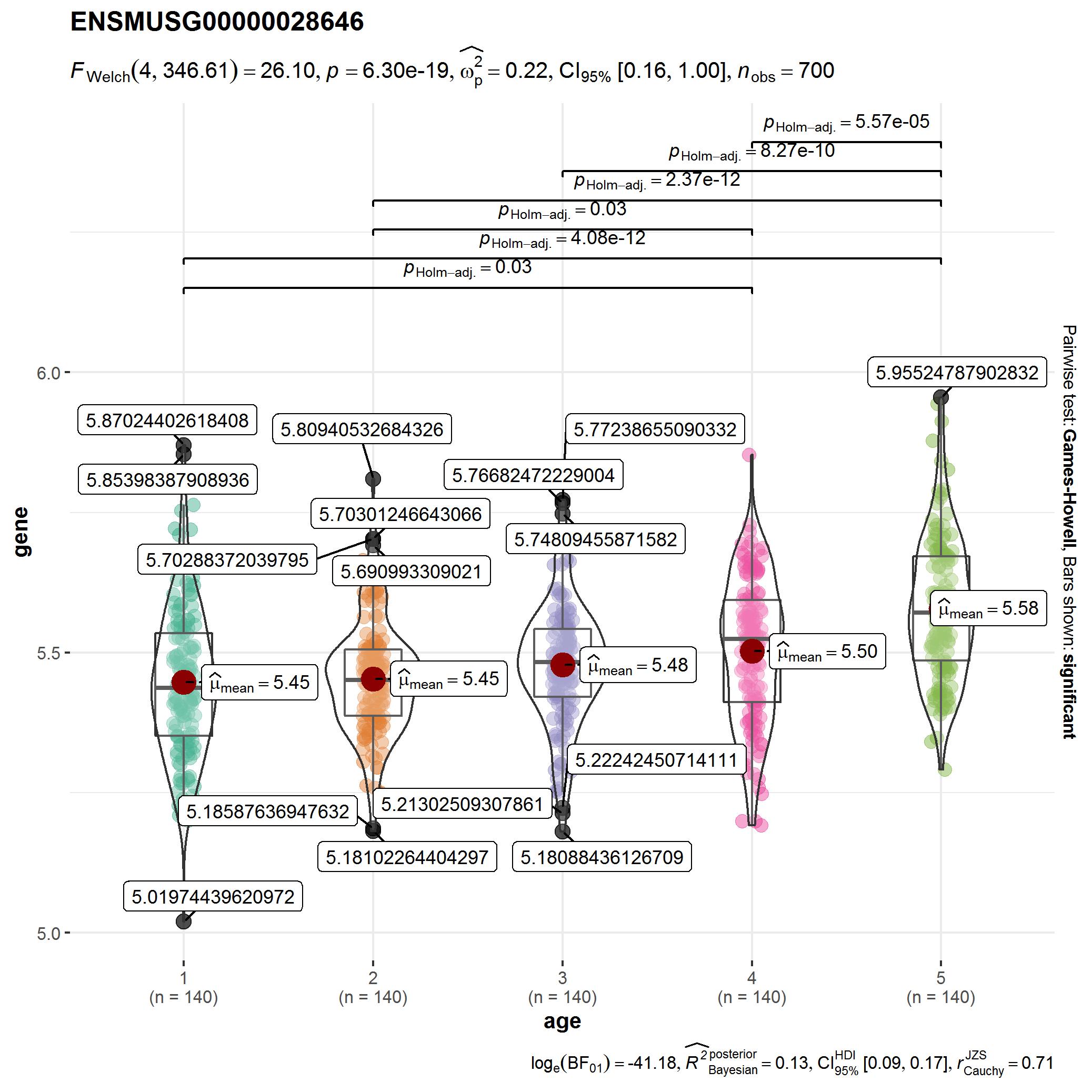

Supplement: Supplementary file 25 — Data S1–S6. [file ACEL-23-e14268-s017.zip › Data S1/ENSMUSG00000028646.jpeg]

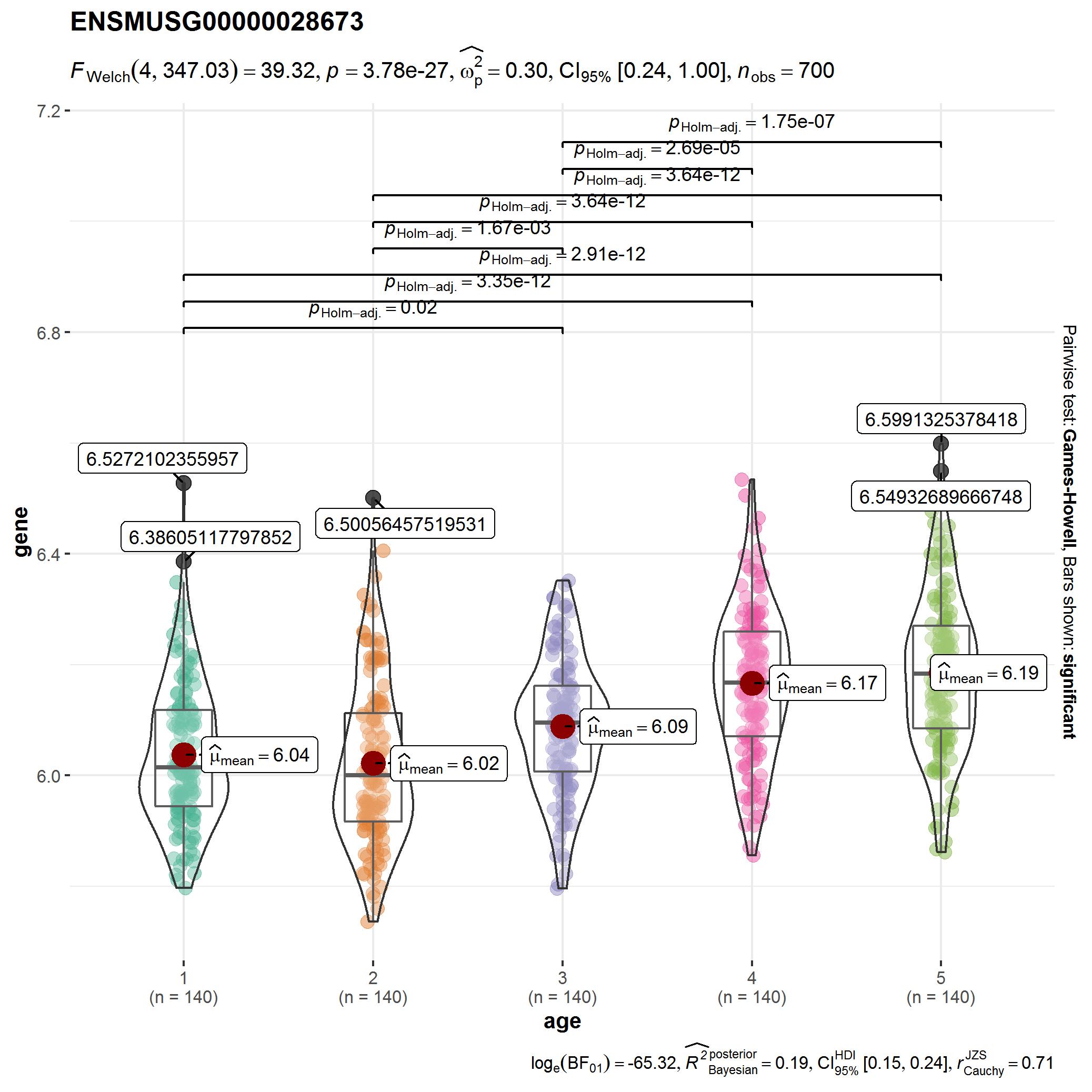

Supplement: Supplementary file 25 — Data S1–S6. [file ACEL-23-e14268-s017.zip › Data S1/ENSMUSG00000028673.jpeg]

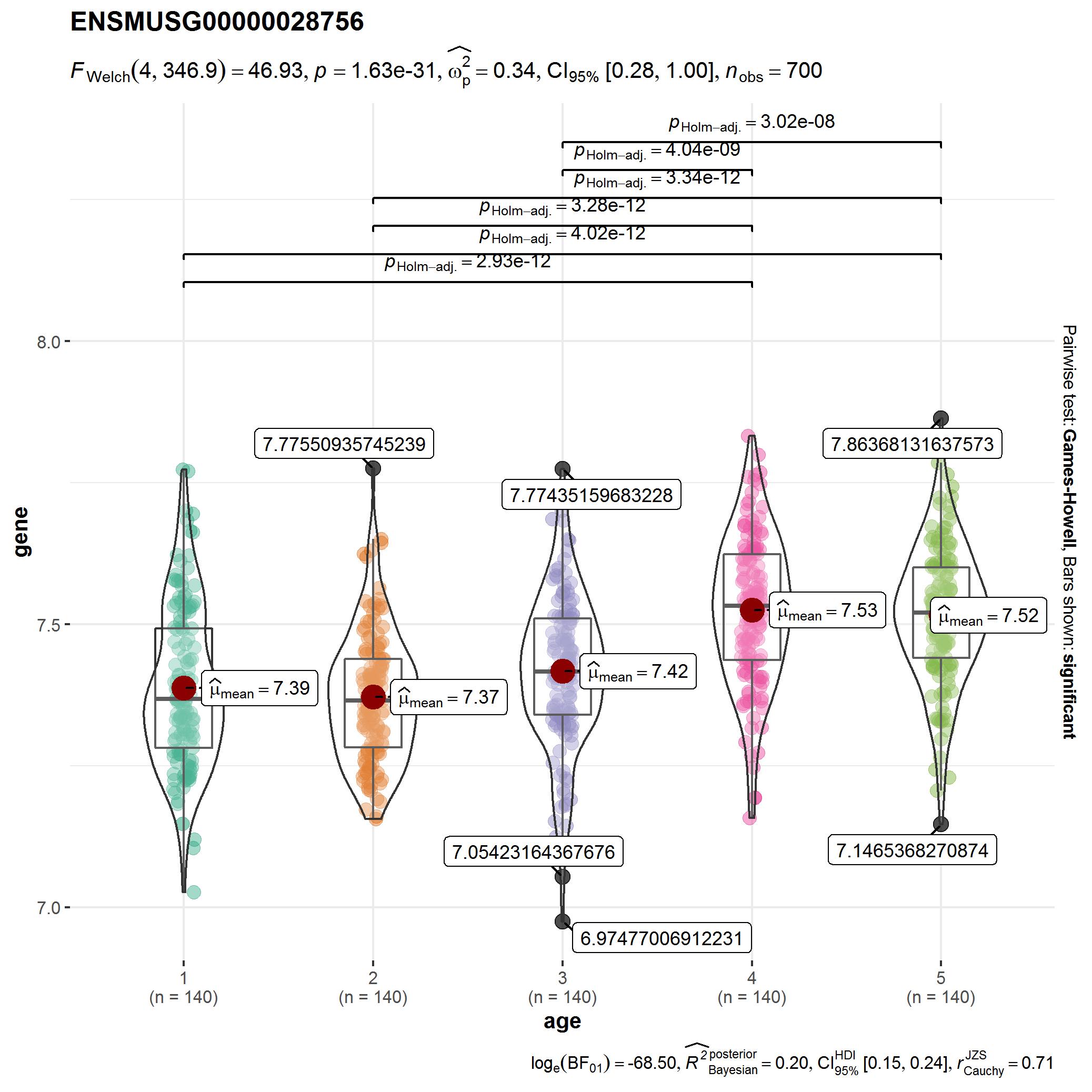

Supplement: Supplementary file 25 — Data S1–S6. [file ACEL-23-e14268-s017.zip › Data S1/ENSMUSG00000028756.jpeg]

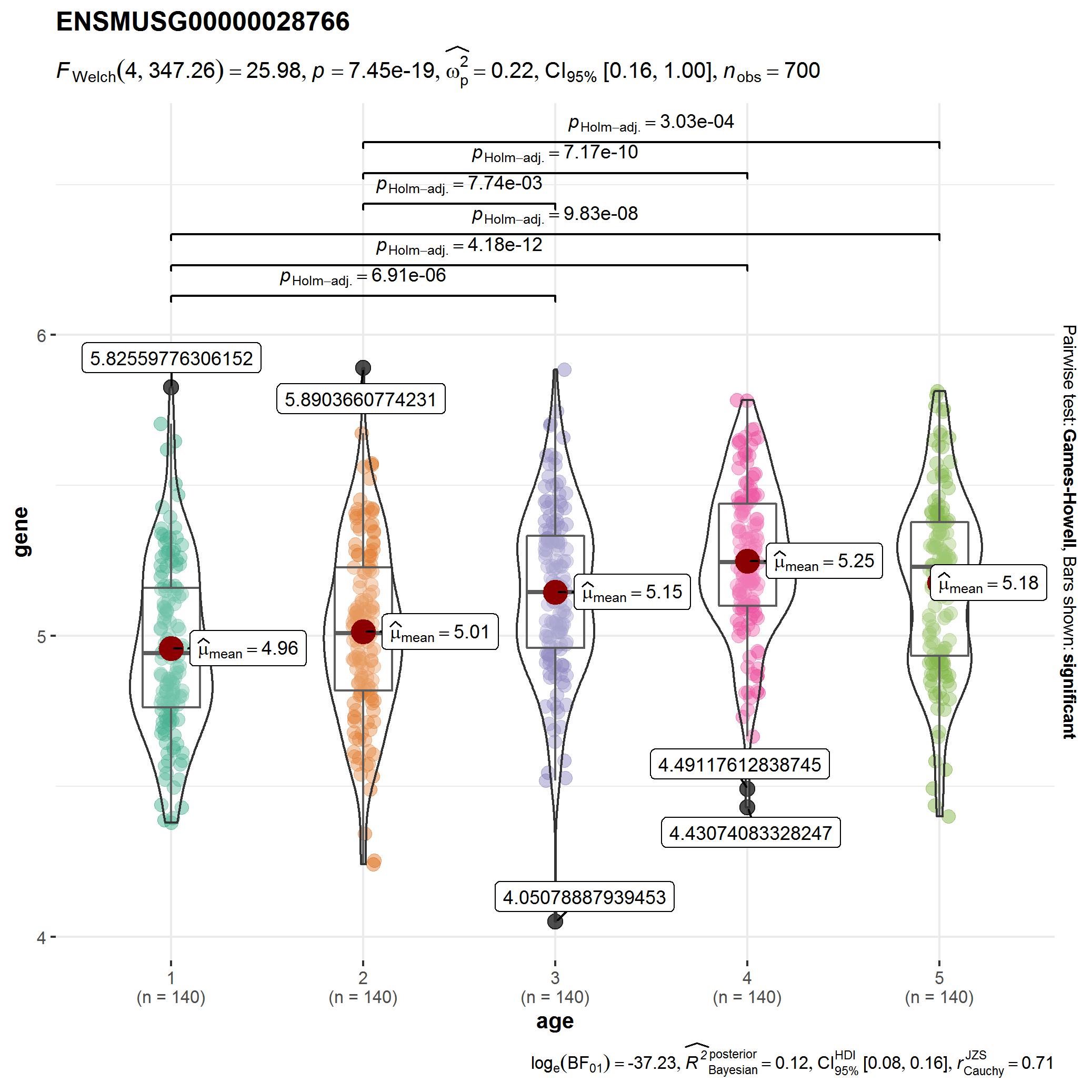

Supplement: Supplementary file 25 — Data S1–S6. [file ACEL-23-e14268-s017.zip › Data S1/ENSMUSG00000028766.jpeg]

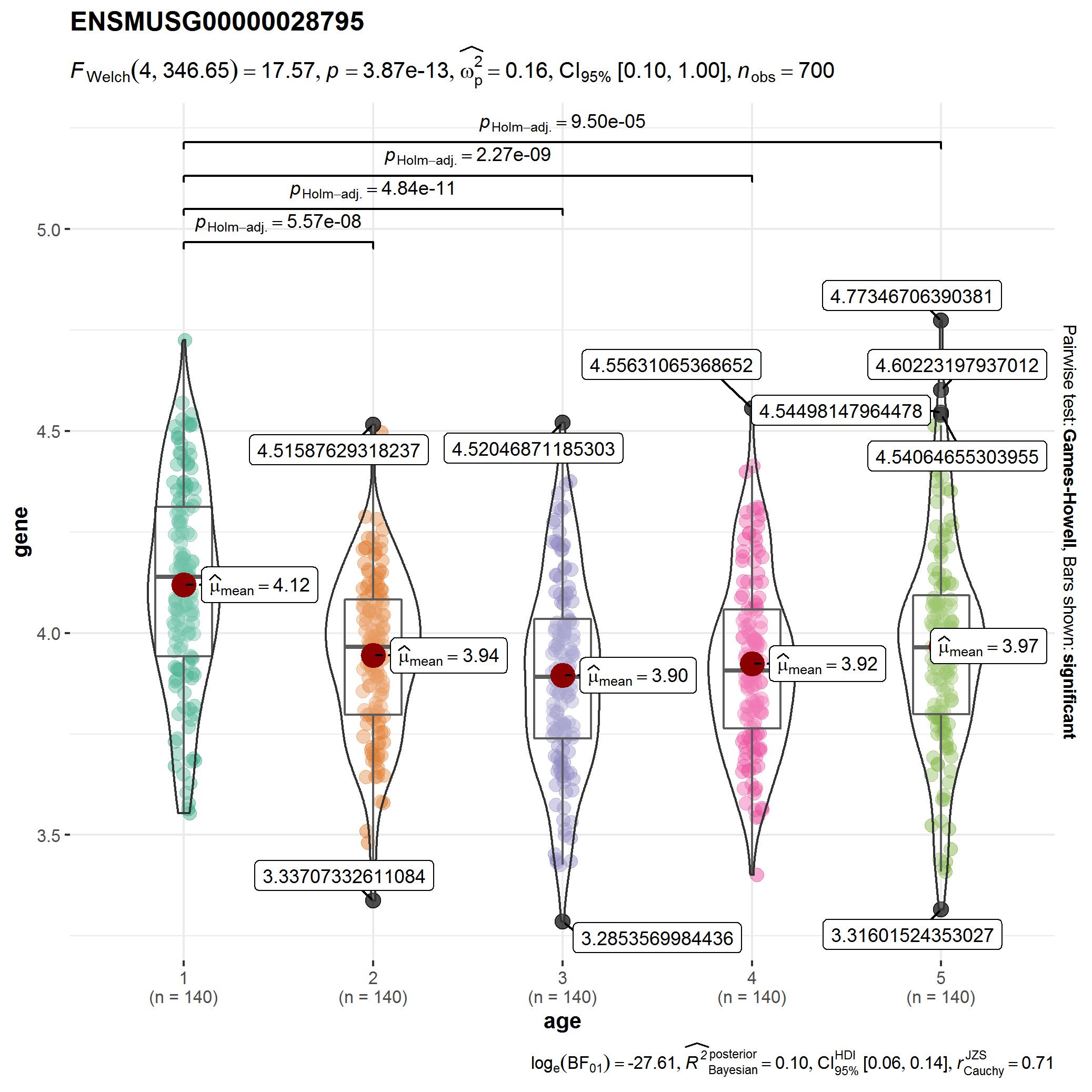

Supplement: Supplementary file 25 — Data S1–S6. [file ACEL-23-e14268-s017.zip › Data S1/ENSMUSG00000028795.jpeg]

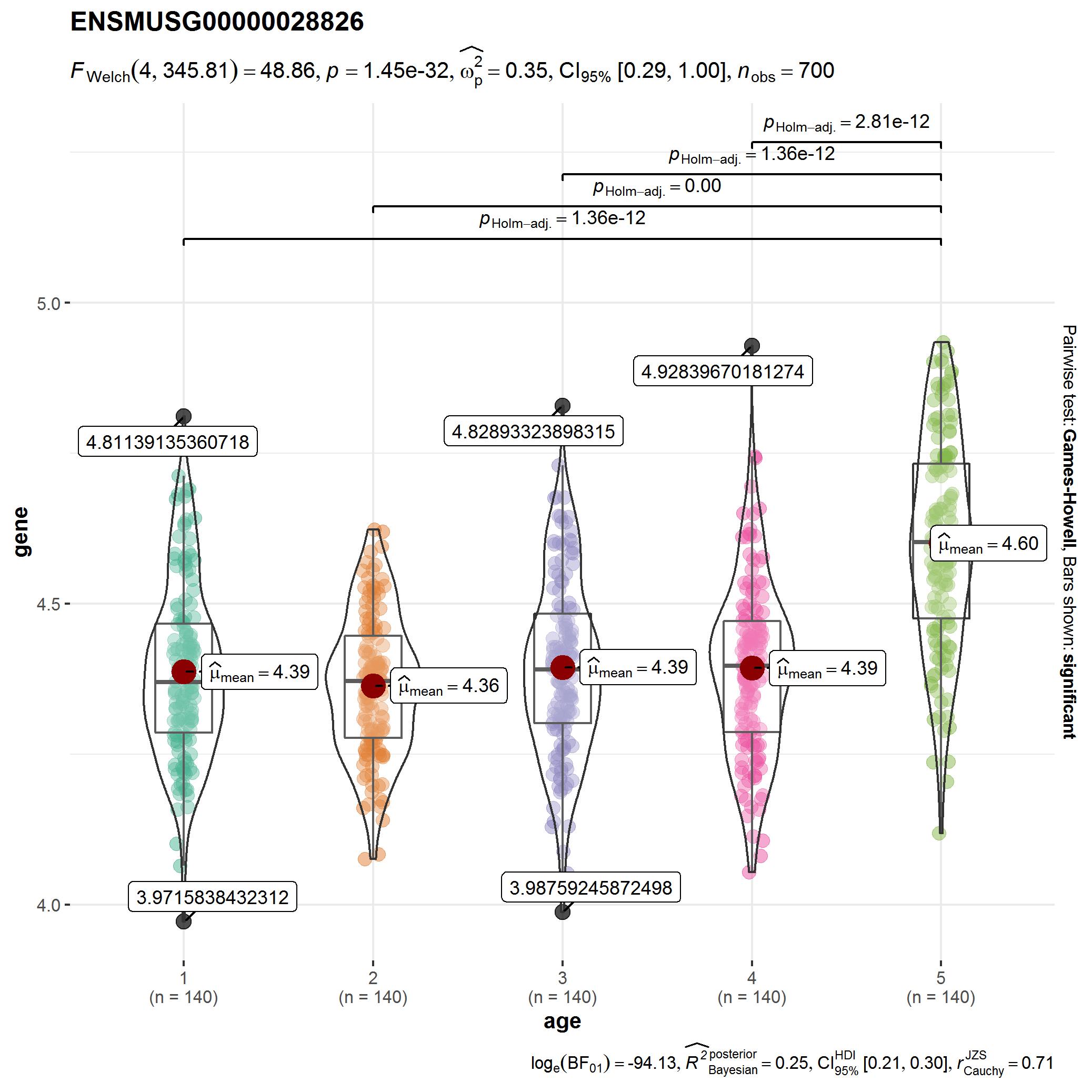

Supplement: Supplementary file 25 — Data S1–S6. [file ACEL-23-e14268-s017.zip › Data S1/ENSMUSG00000028826.jpeg]
