# Supplementary material for: The circadian rhythm: A key variable in aging?
Source: Aging Cell. 2024 Jul 30;23(11):e14268. doi: 10.1111/acel.14268 (PMC11561671; doi:10.1111/acel.14268)
Supplement: Supplementary file 25 — Data S1–S6. [file ACEL-23-e14268-s017.zip › Data S2-S6/Data S3 new.pdf]

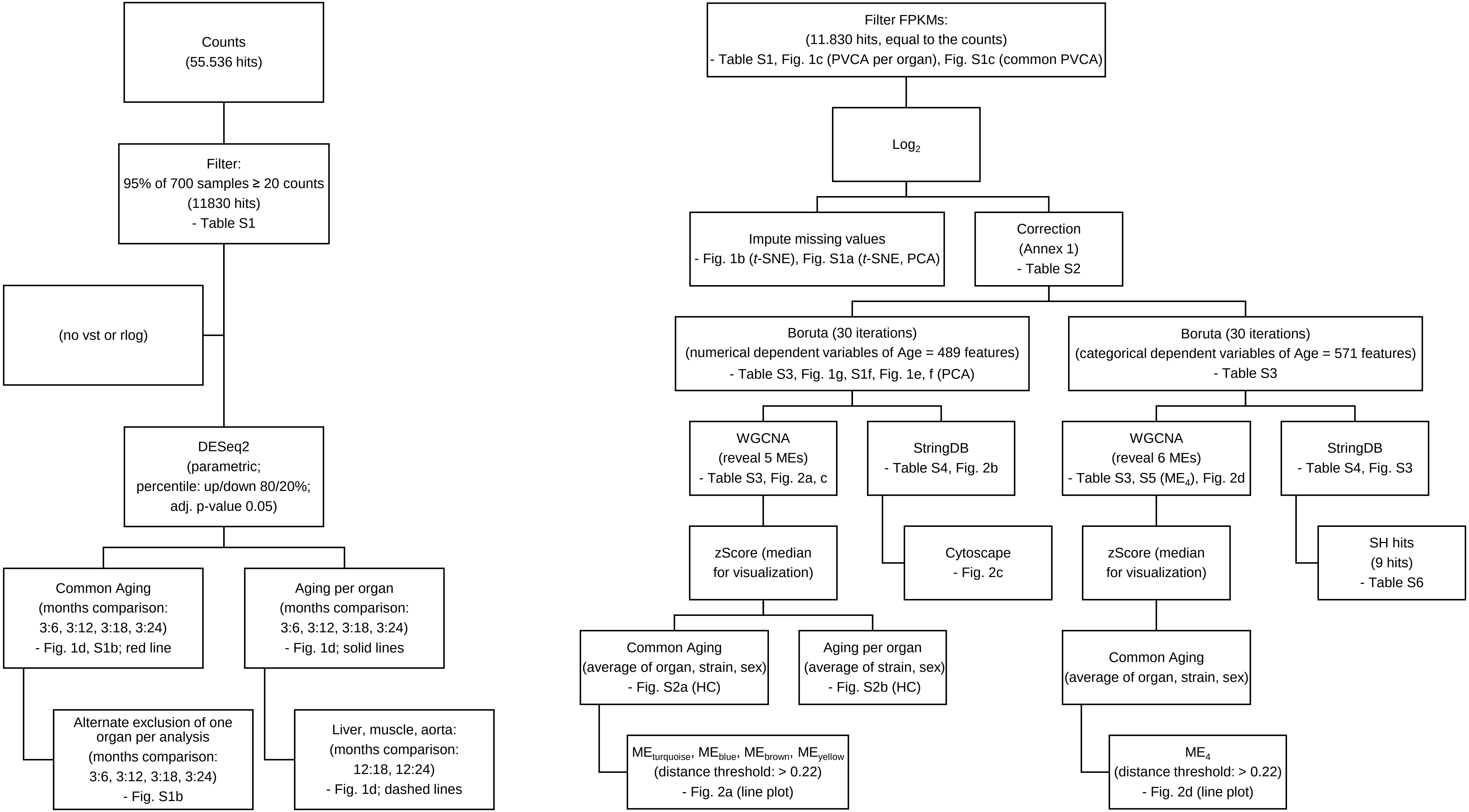

Counts
(55.536 hits)
Filter:
95% of 700 samples ≥ 20 counts
(11830 hits)
- Table S1
(no vst or rlog)
DESeq2
(parametric;
percentile: up/down 80/20%;
adj. p-value 0.05)
Common Aging
(months comparison:
3:6, 3:12, 3:18, 3:24)
- Fig. 1d, S1b; red line
Aging per organ
(months comparison:
3:6, 3:12, 3:18, 3:24)
- Fig. 1d; solid lines
Alternate exclusion of one organ per analysis
(months comparison:
3:6, 3:12, 3:18, 3:24)
- Fig. S1b
Liver, muscle, aorta:
(months comparison:
12:18, 12:24)
- Fig. 1d; dashed lines
Filter FPKMs:
(11.830 hits, equal to the counts)
- Table S1, Fig. 1c (PVCA per organ), Fig. S1c (common PVCA)
Log_2
Impute missing values
- Fig. 1b (*t*-SNE), Fig. S1a (*t*-SNE, PCA)
Correction
(Annex 1)
- Table S2
Boruta (30 iterations)
(numerical dependent variables of Age = 489 features)
- Table S3, Fig. 1g, S1f, Fig. 1e, f (PCA)
Boruta (30 iterations)
(categorical dependent variables of Age = 571 features)
- Table S3
WGCNA
(reveal 5 MEs)
- Table S3, Fig. 2a, c
StringDB
- Table S4, Fig. 2b
WGCNA
(reveal 6 MEs)
- Table S3, S5 (ME_4), Fig. 2d
StringDB
- Table S4, Fig. S3
zScore (median
for visualization)
Cytoscape
- Fig. 2c
zScore (median
for visualization)
SH hits
(9 hits)
- Table S6
Common Aging
(average of organ, strain, sex)
- Fig. S2a (HC)
Aging per organ
(average of strain, sex)
- Fig. S2b (HC)
Common Aging
(average of organ, strain, sex)
$ME_{turquoise}$, ME_{blue}, ME_{brown}, ME_{yellow}
(distance threshold: > 0.22)
- Fig. 2a (line plot)
ME_4
(distance threshold: > 0.22)
- Fig. 2d (line plot)

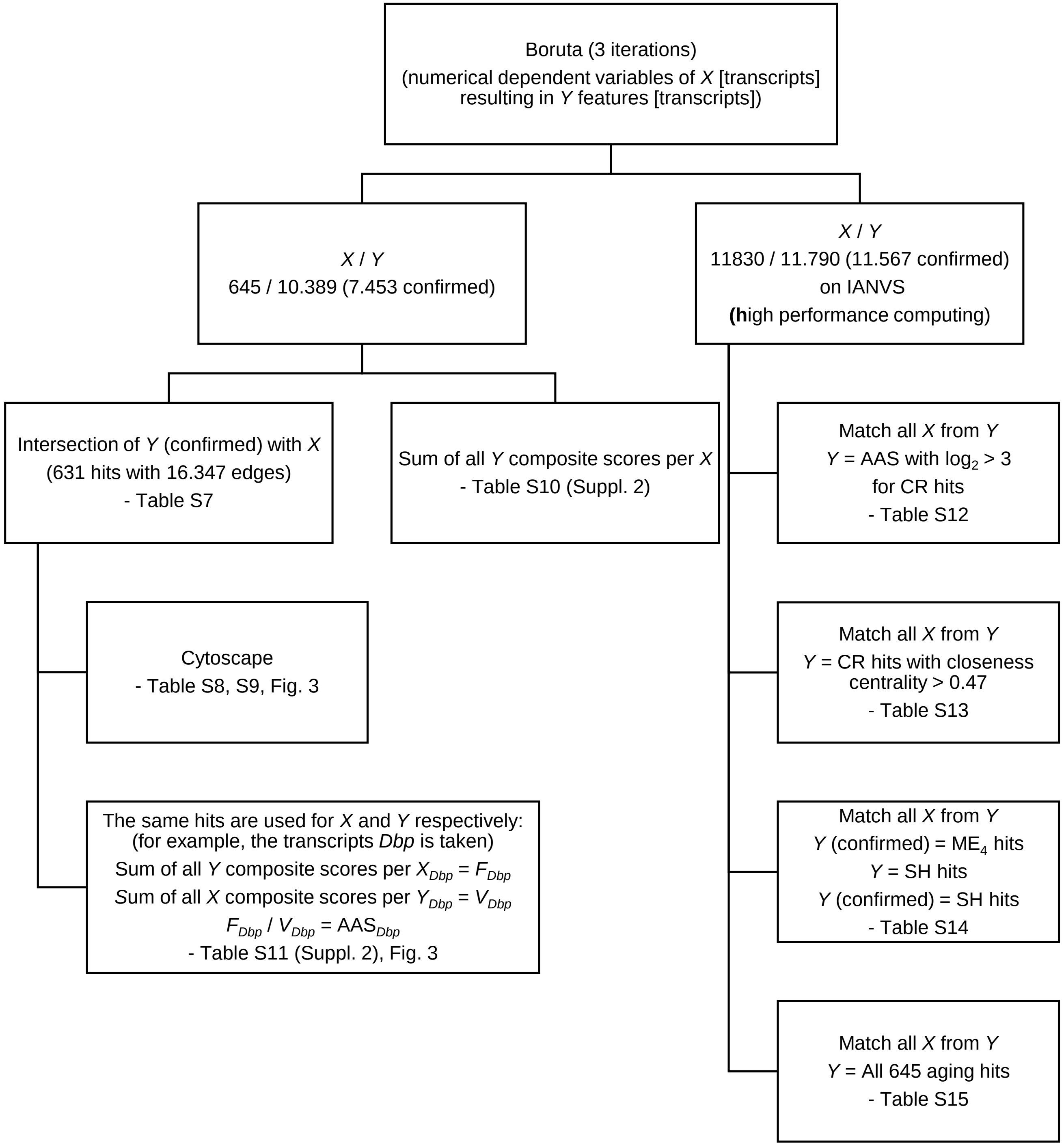
Boruta (3 iterations)
(numerical dependent variables of *X* [transcripts] resulting in *Y* features [transcripts])
X / *Y*
645 / 10.389 (7.453 confirmed)
X / *Y*
11830 / 11.790 (11.567 confirmed)
on IANVS
(**h**igh performance computing)
Intersection of *Y* (confirmed) with *X*
(631 hits with 16.347 edges)
- Table S7
Sum of all *Y* composite scores per *X*
- Table S10 (Suppl. 2)
Cytoscape
- Table S8, S9, Fig. 3
The same hits are used for *X* and *Y* respectively:
(for example, the transcripts *Dbp* is taken)
Sum of all *Y* composite scores per $X_{Dbp} = F_{Dbp}$
Sum of all *X* composite scores per $Y_{Dbp} = V_{Dbp}$
$F_{Dbp} / V_{Dbp} = AAS_{Dbp}$
- Table S11 (Suppl. 2), Fig. 3
Match all *X* from *Y*
Y = AAS with $\log_2 > 3$
for CR hits
- Table S12
Match all *X* from *Y*
Y = CR hits with closeness centrality > 0.47
- Table S13
Match all *X* from *Y*
Y (confirmed) = ME_4 hits
Y = SH hits
Y (confirmed) = SH hits
- Table S14
Match all *X* from *Y*
Y = All 645 aging hits
- Table S15

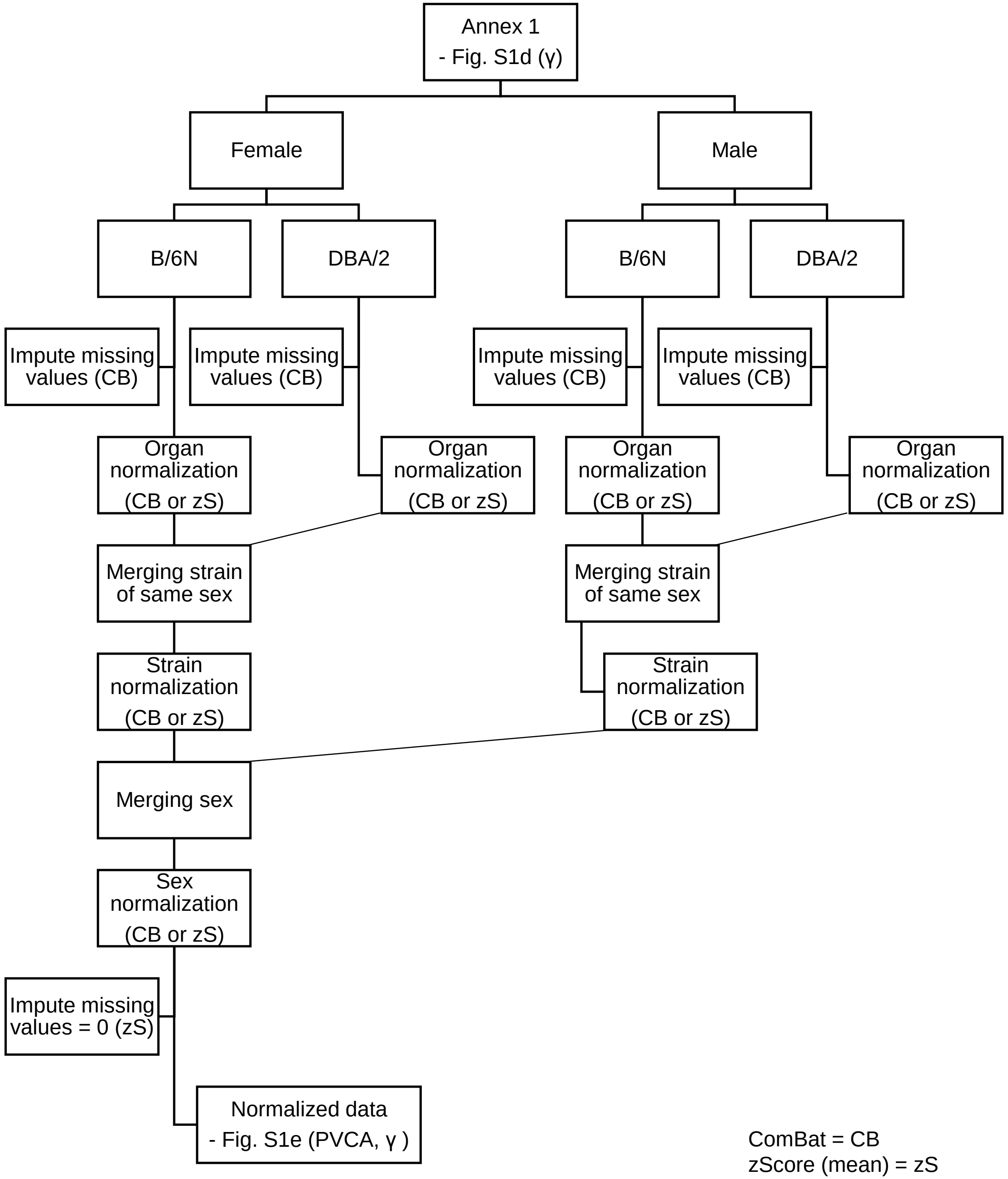
Annex 1
- Fig. S1d (γ)
Female
Male
B/6N
DBA/2
B/6N
DBA/2
Impute missing values (CB)
Impute missing values (CB)
Impute missing values (CB)
Impute missing values (CB)
Organ normalization (CB or zS)
Organ normalization (CB or zS)
Organ normalization (CB or zS)
Organ normalization (CB or zS)
Merging strain of same sex
Merging strain of same sex
Strain normalization (CB or zS)
Strain normalization (CB or zS)
Merging sex
Sex normalization (CB or zS)
Impute missing values = 0 (zS)
Normalized data
- Fig. S1e (PVCA, γ)
ComBat = CB
zScore (mean) = zS
