# Supplementary material for: The circadian rhythm: A key variable in aging?
Source: Aging Cell. 2024 Jul 30;23(11):e14268. doi: 10.1111/acel.14268 (PMC11561671; doi:10.1111/acel.14268)
Supplement: Supplementary file 25 — Data S1–S6. [file ACEL-23-e14268-s017.zip › Data S2-S6/Data S4 new.pdf]

Boruta Age:

```
library(readxl)

library(mlbench)
library(Boruta)
library(caret)
library(randomForest)

A <-read_excel("Table S2")

A$Organ <-as.factor(A$Organ)
A$Sex <-as.factor(A$Sex)
A$Strain <-as.factor(A$Strain)

N<- A
A$Age <-as.factor(A$Age)
C<- A


results_N = list()
for (i in 1:30){
  print(i)
  set.seed(i)
  output <- Boruta(Age ~ ., data = N, doTrace = 2)
  results_N[[i]] = output
}
Sys.time()

attlist = list()
for (i in 1:30){
  tmp = attStats(results_N[[i]])
  attlist[[i]] = tmp
}

Boruta_N_stacked = dplyr::bind_rows(attlist)
write.csv((Boruta_N_stacked), "Table N", row.names = TRUE)


results_C = list()
for (i in 1:30){
  print(i)
  set.seed(i)
  output <- Boruta(Age ~ ., data = C, doTrace = 2)
  results_C[[i]] = output
}
Sys.time()

attlist = list()
for (i in 1:30){
  tmp = attStats(results_C[[i]])
  attlist[[i]] = tmp
}

Boruta_C_stacked = dplyr::bind_rows(attlist)
write.csv((Boruta_C_stacked), "Table C", row.names = TRUE)
```

Boruta 645 aging genes:

```
AgeNC645CB = list()
for (i in
c("ENSMUSG00000000056","ENSMUSG00000000154","ENSMUSG00000000184","ENSMUSG00
000000290","ENSMUSG00000000682","ENSMUSG00000000823","ENSMUSG00000001119","
ENSMUSG00000001143","ENSMUSG00000001229","ENSMUSG00000001506","ENSMUSG00000
001663","ENSMUSG00000001774","ENSMUSG00000001930","ENSMUSG00000002017","ENS
MUSG00000002289","ENSMUSG00000002763","ENSMUSG00000002900","ENSMUSG00000002
985","ENSMUSG00000002996","ENSMUSG00000003031","ENSMUSG00000003154","ENSMUS
G00000003283","ENSMUSG00000003348","ENSMUSG00000003458","ENSMUSG00000003541
","ENSMUSG00000003617","ENSMUSG00000003731","ENSMUSG00000003814","ENSMUSG00
000003849","ENSMUSG00000003949","ENSMUSG00000004105","ENSMUSG00000004460","
ENSMUSG00000004609","ENSMUSG00000004637","ENSMUSG00000005107","ENSMUSG00000
005397","ENSMUSG00000005417","ENSMUSG00000005447","ENSMUSG00000005686","ENS
MUSG00000005936","ENSMUSG00000006127","ENSMUSG00000006373","ENSMUSG00000006
386","ENSMUSG00000006611","ENSMUSG00000007617","ENSMUSG00000007777","ENSMUS
G00000007891","ENSMUSG00000008540","ENSMUSG00000008845","ENSMUSG00000009406
","ENSMUSG00000010554","ENSMUSG00000010651","ENSMUSG00000011256","ENSMUSG00
000013150","ENSMUSG00000013275","ENSMUSG00000014361","ENSMUSG00000014776","
ENSMUSG00000015189","ENSMUSG00000015340","ENSMUSG00000015468","ENSMUSG00000
015839","ENSMUSG00000015950","ENSMUSG00000016206","ENSMUSG00000016256","ENS
MUSG00000016946","ENSMUSG00000017132","ENSMUSG00000017707","ENSMUSG00000017
754","ENSMUSG00000017774","ENSMUSG00000018042","ENSMUSG00000018160","ENSMUS
G00000018217","ENSMUSG00000018415","ENSMUSG00000018427","ENSMUSG00000018501
","ENSMUSG00000018574","ENSMUSG00000018593","ENSMUSG00000018654","ENSMUSG00
000018666","ENSMUSG00000018774","ENSMUSG00000019055","ENSMUSG00000019843","
ENSMUSG00000019846","ENSMUSG00000019872","ENSMUSG00000019916","ENSMUSG00000
019923","ENSMUSG00000020009","ENSMUSG00000020034","ENSMUSG00000020038","ENS
MUSG00000020044","ENSMUSG00000020048","ENSMUSG00000020053","ENSMUSG00000020
111","ENSMUSG00000020120","ENSMUSG00000020123","ENSMUSG00000020143","ENSMUS
G00000020227","ENSMUSG00000020241","ENSMUSG00000020257","ENSMUSG00000020277
","ENSMUSG00000020288","ENSMUSG00000020459","ENSMUSG00000020467","ENSMUSG00
000020472","ENSMUSG00000020571","ENSMUSG00000020594","ENSMUSG00000020653","
ENSMUSG00000020661","ENSMUSG00000020674","ENSMUSG00000020810","ENSMUSG00000
020889","ENSMUSG00000020893","ENSMUSG00000020935","ENSMUSG00000020954","ENS
MUSG00000021003","ENSMUSG00000021025","ENSMUSG00000021037","ENSMUSG00000021
091","ENSMUSG00000021188","ENSMUSG00000021190","ENSMUSG00000021242","ENSMUS
G00000021262","ENSMUSG00000021306","ENSMUSG00000021508","ENSMUSG00000021559
","ENSMUSG00000021775","ENSMUSG00000021814","ENSMUSG00000021903","ENSMUSG00
000021939","ENSMUSG00000021998","ENSMUSG00000022035","ENSMUSG00000022091","
ENSMUSG00000022098","ENSMUSG00000022146","ENSMUSG00000022148","ENSMUSG00000
022186","ENSMUSG00000022197","ENSMUSG00000022270","ENSMUSG00000022372","ENS
MUSG00000022389","ENSMUSG00000022415","ENSMUSG00000022440","ENSMUSG00000022
472","ENSMUSG00000022488","ENSMUSG00000022623","ENSMUSG00000022656","ENSMUS
G00000022665","ENSMUSG00000022684","ENSMUSG00000022797","ENSMUSG00000022816
","ENSMUSG00000022831","ENSMUSG00000022848","ENSMUSG00000022912","ENSMUSG00
000022952","ENSMUSG00000022999","ENSMUSG00000023055","ENSMUSG00000023249","
ENSMUSG00000023328","ENSMUSG00000023367","ENSMUSG00000023952","ENSMUSG00000
023960","ENSMUSG00000024132","ENSMUSG00000024160","ENSMUSG00000024164","ENS
MUSG00000024180","ENSMUSG00000024190","ENSMUSG00000024222","ENSMUSG00000024
299","ENSMUSG00000024300","ENSMUSG00000024421","ENSMUSG00000024451","ENSMUS
G00000024501","ENSMUSG00000024538","ENSMUSG00000024576","ENSMUSG00000024610
","ENSMUSG00000024654","ENSMUSG00000024679","ENSMUSG00000024736","ENSMUSG00
000024810","ENSMUSG00000024885","ENSMUSG00000024900","ENSMUSG00000024965","
ENSMUSG00000024966","ENSMUSG00000024975","ENSMUSG00000025017","ENSMUSG00000
025037","ENSMUSG00000025133","ENSMUSG00000025158","ENSMUSG00000025176","ENS
MUSG00000025262","ENSMUSG00000025265","ENSMUSG00000025270","ENSMUSG00000025
```

358","ENSMUSG00000025511","ENSMUSG00000025512","ENSMUSG00000025584","ENSMUS
G00000025586","ENSMUSG00000025743","ENSMUSG00000025821","ENSMUSG00000025885
","ENSMUSG00000025888","ENSMUSG00000025980","ENSMUSG00000025981","ENSMUSG00
000025986","ENSMUSG00000026003","ENSMUSG00000026042","ENSMUSG00000026043","
ENSMUSG00000026074","ENSMUSG00000026077","ENSMUSG00000026082","ENSMUSG00000
026234","ENSMUSG00000026288","ENSMUSG00000026303","ENSMUSG00000026395","ENS
MUSG00000026478","ENSMUSG00000026480","ENSMUSG00000026548","ENSMUSG00000026
556","ENSMUSG00000026566","ENSMUSG00000026608","ENSMUSG00000026672","ENSMUS
G00000026728","ENSMUSG00000026739","ENSMUSG00000026786","ENSMUSG00000026837
","ENSMUSG00000026864","ENSMUSG00000026872","ENSMUSG00000026880","ENSMUSG00
000026956","ENSMUSG00000026979","ENSMUSG00000027204","ENSMUSG00000027248","
ENSMUSG00000027274","ENSMUSG00000027330","ENSMUSG00000027412","ENSMUSG00000
027435","ENSMUSG00000027635","ENSMUSG00000027669","ENSMUSG00000027843","ENS
MUSG00000027852","ENSMUSG00000027864","ENSMUSG00000027875","ENSMUSG00000027
933","ENSMUSG00000027947","ENSMUSG00000027962","ENSMUSG00000027995","ENSMUS
G00000028028","ENSMUSG00000028082","ENSMUSG00000028223","ENSMUSG00000028309
","ENSMUSG00000028329","ENSMUSG00000028337","ENSMUSG00000028339","ENSMUSG00
000028343","ENSMUSG00000028410","ENSMUSG00000028581","ENSMUSG00000028597","
ENSMUSG00000028600","ENSMUSG00000028630","ENSMUSG00000028641","ENSMUSG00000
028646","ENSMUSG00000028673","ENSMUSG00000028756","ENSMUSG00000028766","ENS
MUSG00000028795","ENSMUSG00000028820","ENSMUSG00000028826","ENSMUSG00000028
859","ENSMUSG00000028862","ENSMUSG00000028878","ENSMUSG00000028885","ENSMUS
G00000028957","ENSMUSG00000028970","ENSMUSG00000029009","ENSMUSG00000029053
","ENSMUSG00000029090","ENSMUSG00000029119","ENSMUSG00000029177","ENSMUSG00
000029229","ENSMUSG00000029238","ENSMUSG00000029312","ENSMUSG00000029330","
ENSMUSG00000029468","ENSMUSG00000029471","ENSMUSG00000029570","ENSMUSG00000
029616","ENSMUSG00000029657","ENSMUSG00000029661","ENSMUSG00000029675","ENS
MUSG00000029687","ENSMUSG00000029762","ENSMUSG00000029778","ENSMUSG00000029
810","ENSMUSG00000029919","ENSMUSG00000029994","ENSMUSG00000030083","ENSMUS
G00000030087","ENSMUSG00000030096","ENSMUSG00000030103","ENSMUSG00000030172
","ENSMUSG00000030256","ENSMUSG00000030357","ENSMUSG00000030579","ENSMUSG00
000030678","ENSMUSG00000030681","ENSMUSG00000030701","ENSMUSG00000030711","
ENSMUSG00000030761","ENSMUSG00000030786","ENSMUSG00000030798","ENSMUSG00000
030921","ENSMUSG00000031004","ENSMUSG00000031016","ENSMUSG00000031024","ENS
MUSG00000031144","ENSMUSG00000031167","ENSMUSG00000031217","ENSMUSG00000031
221","ENSMUSG00000031232","ENSMUSG00000031239","ENSMUSG00000031274","ENSMUS
G00000031298","ENSMUSG00000031343","ENSMUSG00000031379","ENSMUSG00000031431
","ENSMUSG00000031442","ENSMUSG00000031467","ENSMUSG00000031479","ENSMUSG00
000031502","ENSMUSG00000031503","ENSMUSG00000031511","ENSMUSG00000031596","
ENSMUSG00000031740","ENSMUSG00000031762","ENSMUSG00000031765","ENSMUSG00000
031770","ENSMUSG00000031778","ENSMUSG00000031790","ENSMUSG00000031864","ENS
MUSG00000032000","ENSMUSG00000032010","ENSMUSG00000032086","ENSMUSG00000032
115","ENSMUSG00000032125","ENSMUSG00000032127","ENSMUSG00000032228","ENSMUS
G00000032348","ENSMUSG00000032349","ENSMUSG00000032363","ENSMUSG00000032369
","ENSMUSG00000032372","ENSMUSG00000032399","ENSMUSG00000032802","ENSMUSG00
000033032","ENSMUSG00000033082","ENSMUSG00000033159","ENSMUSG00000033191","
ENSMUSG00000033220","ENSMUSG00000033249","ENSMUSG00000033308","ENSMUSG00000
033318","ENSMUSG00000033460","ENSMUSG00000033499","ENSMUSG00000033581","ENS
MUSG00000033863","ENSMUSG00000033933","ENSMUSG00000034006","ENSMUSG00000034
007","ENSMUSG00000034116","ENSMUSG00000034154","ENSMUSG00000034157","ENSMUS
G00000034205","ENSMUSG00000034252","ENSMUSG00000034255","ENSMUSG00000034285
","ENSMUSG00000034341","ENSMUSG00000034349","ENSMUSG00000034403","ENSMUSG00
000034614","ENSMUSG00000034647","ENSMUSG00000034656","ENSMUSG00000034674","
ENSMUSG00000034707","ENSMUSG00000034795","ENSMUSG00000034832","ENSMUSG00000
034936","ENSMUSG00000034947","ENSMUSG00000034993","ENSMUSG00000034994","ENS
MUSG00000035004","ENSMUSG00000035064","ENSMUSG00000035206","ENSMUSG00000035
212","ENSMUSG00000035245","ENSMUSG00000035273","ENSMUSG00000035504","ENSMUS
G00000035513","ENSMUSG00000035772","ENSMUSG00000035824","ENSMUSG00000035828

","ENSMUSG00000035834","ENSMUSG00000035877","ENSMUSG00000035936","ENSMUSG00
000036040","ENSMUSG00000036273","ENSMUSG00000036275","ENSMUSG00000036402","
ENSMUSG00000036427","ENSMUSG00000036545","ENSMUSG00000036594","ENSMUSG00000
036615","ENSMUSG00000036667","ENSMUSG00000036862","ENSMUSG00000036887","ENS
MUSG00000036893","ENSMUSG00000036896","ENSMUSG00000036905","ENSMUSG00000036
934","ENSMUSG00000036944","ENSMUSG00000037003","ENSMUSG00000037058","ENSMUS
G00000037235","ENSMUSG00000037347","ENSMUSG00000037405","ENSMUSG00000037411
","ENSMUSG00000037455","ENSMUSG00000037461","ENSMUSG00000037475","ENSMUSG00
000037613","ENSMUSG00000037712","ENSMUSG00000037824","ENSMUSG00000037860","
ENSMUSG00000037892","ENSMUSG00000038007","ENSMUSG00000038143","ENSMUSG00000
038147","ENSMUSG00000038175","ENSMUSG00000038235","ENSMUSG00000038305","ENS
MUSG00000038393","ENSMUSG00000038521","ENSMUSG00000038642","ENSMUSG00000038
695","ENSMUSG00000038776","ENSMUSG00000039096","ENSMUSG00000039126","ENSMUS
G00000039159","ENSMUSG00000039176","ENSMUSG00000039254","ENSMUSG00000039328
","ENSMUSG00000039382","ENSMUSG00000039745","ENSMUSG00000039982","ENSMUSG00
000040093","ENSMUSG00000040097","ENSMUSG00000040170","ENSMUSG00000040174","
ENSMUSG00000040552","ENSMUSG00000040612","ENSMUSG00000040631","ENSMUSG00000
040713","ENSMUSG00000040723","ENSMUSG00000040751","ENSMUSG00000040820","ENS
MUSG00000041040","ENSMUSG00000041084","ENSMUSG00000041117","ENSMUSG00000041
355","ENSMUSG00000041417","ENSMUSG00000041515","ENSMUSG00000041560","ENSMUS
G00000041779","ENSMUSG00000041801","ENSMUSG00000041828","ENSMUSG00000041849
","ENSMUSG00000041926","ENSMUSG00000041936","ENSMUSG00000041992","ENSMUSG00
000042035","ENSMUSG00000042099","ENSMUSG00000042129","ENSMUSG00000042155","
ENSMUSG00000042251","ENSMUSG00000042286","ENSMUSG00000042426","ENSMUSG00000
042436","ENSMUSG00000042487","ENSMUSG00000042594","ENSMUSG00000042644","ENS
MUSG00000042677","ENSMUSG00000042688","ENSMUSG00000042712","ENSMUSG00000042
834","ENSMUSG00000043631","ENSMUSG00000044349","ENSMUSG00000044562","ENSMUS
G00000044583","ENSMUSG00000044949","ENSMUSG00000045193","ENSMUSG00000046230
","ENSMUSG00000046312","ENSMUSG00000046318","ENSMUSG00000046532","ENSMUSG00
000046688","ENSMUSG00000046743","ENSMUSG00000046805","ENSMUSG00000046994","
ENSMUSG00000047045","ENSMUSG00000047238","ENSMUSG00000047866","ENSMUSG00000
047945","ENSMUSG00000048126","ENSMUSG00000048249","ENSMUSG00000048330","ENS
MUSG00000048489","ENSMUSG00000048756","ENSMUSG00000049047","ENSMUSG00000049
076","ENSMUSG00000049804","ENSMUSG00000049878","ENSMUSG00000050103","ENSMUS
G00000050335","ENSMUSG00000050708","ENSMUSG00000050714","ENSMUSG00000051483
","ENSMUSG00000051506","ENSMUSG00000051579","ENSMUSG00000051864","ENSMUSG00
000052040","ENSMUSG00000052139","ENSMUSG00000052305","ENSMUSG00000052593","
ENSMUSG00000052760","ENSMUSG00000052812","ENSMUSG00000052915","ENSMUSG00000
053063","ENSMUSG00000053279","ENSMUSG00000053898","ENSMUSG00000054072","ENS
MUSG00000054404","ENSMUSG00000054509","ENSMUSG00000054612","ENSMUSG00000054
733","ENSMUSG00000054863","ENSMUSG00000055067","ENSMUSG00000055116","ENSMUS
G00000055541","ENSMUSG00000055866","ENSMUSG00000055980","ENSMUSG00000056228
","ENSMUSG00000056515","ENSMUSG00000056666","ENSMUSG00000056737","ENSMUSG00
000056749","ENSMUSG00000057137","ENSMUSG00000057193","ENSMUSG00000057406","
ENSMUSG00000057497","ENSMUSG00000057531","ENSMUSG00000057894","ENSMUSG00000
057897","ENSMUSG00000058135","ENSMUSG00000058586","ENSMUSG00000058587","ENS
MUSG00000058655","ENSMUSG00000058715","ENSMUSG00000058818","ENSMUSG00000059
013","ENSMUSG00000059182","ENSMUSG00000059498","ENSMUSG00000059714","ENSMUS
G00000059824","ENSMUSG00000059851","ENSMUSG00000059970","ENSMUSG00000060036
","ENSMUSG00000060373","ENSMUSG00000060586","ENSMUSG00000060671","ENSMUSG00
000060771","ENSMUSG00000060862","ENSMUSG00000060989","ENSMUSG00000061601","
ENSMUSG00000061740","ENSMUSG00000061838","ENSMUSG00000061950","ENSMUSG00000
062203","ENSMUSG00000063060","ENSMUSG00000063065","ENSMUSG00000063171","ENS
MUSG00000063275","ENSMUSG00000063415","ENSMUSG00000063576","ENSMUSG00000063
663","ENSMUSG00000064267","ENSMUSG00000064346","ENSMUSG00000066245","ENSMUS
G00000066640","ENSMUSG00000066687","ENSMUSG00000067274","ENSMUSG00000068290
","ENSMUSG00000068742","ENSMUSG00000068854","ENSMUSG00000069516","ENSMUSG00
000069833","ENSMUSG00000070031","ENSMUSG00000070436","ENSMUSG00000070544","

```
ENSMUSG00000071203","ENSMUSG00000071637","ENSMUSG00000071650","ENSMUSG00000
071714","ENSMUSG00000071715","ENSMUSG00000072949","ENSMUSG00000073409","ENS
MUSG00000073418","ENSMUSG00000073421","ENSMUSG00000073940","ENSMUSG00000074
264","ENSMUSG00000074748","ENSMUSG00000075284","ENSMUSG00000076435","ENSMUS
G00000076617","ENSMUSG00000078566","ENSMUSG00000078771","ENSMUSG00000078853
","ENSMUSG00000078922","ENSMUSG00000078945","ENSMUSG00000079037","ENSMUSG00
000079293","ENSMUSG00000079363","ENSMUSG00000079419","ENSMUSG00000079491","
ENSMUSG00000079499","ENSMUSG00000080316","ENSMUSG00000089715","ENSMUSG00000
090877","ENSMUSG00000092981","ENSMUSG00000095041","ENSMUSG00000096929","ENS
MUSG00000097415","ENSMUSG00000099875","ENSMUSG00000108218","ENSMUSG00000110
588","ENSMUSG00000111027","ENSMUSG00000114277")){
  print(i)
  set.seed(1)
  data = N
  y=data.frame(data[,i])
  y=y[,1]
  x=data.frame(data[,colnames(data)!= i])
  output <- Boruta(y = y, x=x, doTrace = 2)
  AgeNC645CB[[i]] = output
}
Sys.time()

attlist = list()
for (i in
c("ENSMUSG00000000056","ENSMUSG00000000154","ENSMUSG00000000184","ENSMUSG00
000000290","ENSMUSG00000000682","ENSMUSG00000000823","ENSMUSG00000001119","
ENSMUSG00000001143","ENSMUSG00000001229","ENSMUSG00000001506","ENSMUSG00000
001663","ENSMUSG00000001774","ENSMUSG00000001930","ENSMUSG00000002017","ENS
MUSG00000002289","ENSMUSG00000002763","ENSMUSG00000002900","ENSMUSG00000002
985","ENSMUSG00000002996","ENSMUSG00000003031","ENSMUSG00000003154","ENSMUS
G00000003283","ENSMUSG00000003348","ENSMUSG00000003458","ENSMUSG00000003541
","ENSMUSG00000003617","ENSMUSG00000003731","ENSMUSG00000003814","ENSMUSG00
000003849","ENSMUSG00000003949","ENSMUSG00000004105","ENSMUSG00000004460","
ENSMUSG00000004609","ENSMUSG00000004637","ENSMUSG00000005107","ENSMUSG00000
005397","ENSMUSG00000005417","ENSMUSG00000005447","ENSMUSG00000005686","ENS
MUSG00000005936","ENSMUSG00000006127","ENSMUSG00000006373","ENSMUSG00000006
386","ENSMUSG00000006611","ENSMUSG00000007617","ENSMUSG00000007777","ENSMUS
G00000007891","ENSMUSG00000008540","ENSMUSG00000008845","ENSMUSG00000009406
","ENSMUSG00000010554","ENSMUSG00000010651","ENSMUSG00000011256","ENSMUSG00
000013150","ENSMUSG00000013275","ENSMUSG00000014361","ENSMUSG00000014776","
ENSMUSG00000015189","ENSMUSG00000015340","ENSMUSG00000015468","ENSMUSG00000
015839","ENSMUSG00000015950","ENSMUSG00000016206","ENSMUSG00000016256","ENS
MUSG00000016946","ENSMUSG00000017132","ENSMUSG00000017707","ENSMUSG00000017
754","ENSMUSG00000017774","ENSMUSG00000018042","ENSMUSG00000018160","ENSMUS
G00000018217","ENSMUSG00000018415","ENSMUSG00000018427","ENSMUSG00000018501
","ENSMUSG00000018574","ENSMUSG00000018593","ENSMUSG00000018654","ENSMUSG00
000018666","ENSMUSG00000018774","ENSMUSG00000019055","ENSMUSG00000019843","
ENSMUSG00000019846","ENSMUSG00000019872","ENSMUSG00000019916","ENSMUSG00000
019923","ENSMUSG00000020009","ENSMUSG00000020034","ENSMUSG00000020038","ENS
MUSG00000020044","ENSMUSG00000020048","ENSMUSG00000020053","ENSMUSG00000020
111","ENSMUSG00000020120","ENSMUSG00000020123","ENSMUSG00000020143","ENSMUS
G00000020227","ENSMUSG00000020241","ENSMUSG00000020257","ENSMUSG00000020277
","ENSMUSG00000020288","ENSMUSG00000020459","ENSMUSG00000020467","ENSMUSG00
000020472","ENSMUSG00000020571","ENSMUSG00000020594","ENSMUSG00000020653","
ENSMUSG00000020661","ENSMUSG00000020674","ENSMUSG00000020810","ENSMUSG00000
020889","ENSMUSG00000020893","ENSMUSG00000020935","ENSMUSG00000020954","ENS
MUSG00000021003","ENSMUSG00000021025","ENSMUSG00000021037","ENSMUSG00000021
091","ENSMUSG00000021188","ENSMUSG00000021190","ENSMUSG00000021242","ENSMUS
```

G00000021262","ENSMUSG00000021306","ENSMUSG00000021508","ENSMUSG00000021559
","ENSMUSG00000021775","ENSMUSG00000021814","ENSMUSG00000021903","ENSMUSG00
000021939","ENSMUSG00000021998","ENSMUSG00000022035","ENSMUSG00000022091","
ENSMUSG00000022098","ENSMUSG00000022146","ENSMUSG00000022148","ENSMUSG00000
022186","ENSMUSG00000022197","ENSMUSG00000022270","ENSMUSG00000022372","ENS
MUSG00000022389","ENSMUSG00000022415","ENSMUSG00000022440","ENSMUSG00000022
472","ENSMUSG00000022488","ENSMUSG00000022623","ENSMUSG00000022656","ENSMUS
G00000022665","ENSMUSG00000022684","ENSMUSG00000022797","ENSMUSG00000022816
","ENSMUSG00000022831","ENSMUSG00000022848","ENSMUSG00000022912","ENSMUSG00
000022952","ENSMUSG00000022999","ENSMUSG00000023055","ENSMUSG00000023249","
ENSMUSG00000023328","ENSMUSG00000023367","ENSMUSG00000023952","ENSMUSG00000
023960","ENSMUSG00000024132","ENSMUSG00000024160","ENSMUSG00000024164","ENS
MUSG00000024180","ENSMUSG00000024190","ENSMUSG00000024222","ENSMUSG00000024
299","ENSMUSG00000024300","ENSMUSG00000024421","ENSMUSG00000024451","ENSMUS
G00000024501","ENSMUSG00000024538","ENSMUSG00000024576","ENSMUSG00000024610
","ENSMUSG00000024654","ENSMUSG00000024679","ENSMUSG00000024736","ENSMUSG00
000024810","ENSMUSG00000024885","ENSMUSG00000024900","ENSMUSG00000024965","
ENSMUSG00000024966","ENSMUSG00000024975","ENSMUSG00000025017","ENSMUSG00000
025037","ENSMUSG00000025133","ENSMUSG00000025158","ENSMUSG00000025176","ENS
MUSG00000025262","ENSMUSG00000025265","ENSMUSG00000025270","ENSMUSG00000025
358","ENSMUSG00000025511","ENSMUSG00000025512","ENSMUSG00000025584","ENSMUS
G00000025586","ENSMUSG00000025743","ENSMUSG00000025821","ENSMUSG00000025885
","ENSMUSG00000025888","ENSMUSG00000025980","ENSMUSG00000025981","ENSMUSG00
000025986","ENSMUSG00000026003","ENSMUSG00000026042","ENSMUSG00000026043","
ENSMUSG00000026074","ENSMUSG00000026077","ENSMUSG00000026082","ENSMUSG00000
026234","ENSMUSG00000026288","ENSMUSG00000026303","ENSMUSG00000026395","ENS
MUSG00000026478","ENSMUSG00000026480","ENSMUSG00000026548","ENSMUSG00000026
556","ENSMUSG00000026566","ENSMUSG00000026608","ENSMUSG00000026672","ENSMUS
G00000026728","ENSMUSG00000026739","ENSMUSG00000026786","ENSMUSG00000026837
","ENSMUSG00000026864","ENSMUSG00000026872","ENSMUSG00000026880","ENSMUSG00
000026956","ENSMUSG00000026979","ENSMUSG00000027204","ENSMUSG00000027248","
ENSMUSG00000027274","ENSMUSG00000027330","ENSMUSG00000027412","ENSMUSG00000
027435","ENSMUSG00000027635","ENSMUSG00000027669","ENSMUSG00000027843","ENS
MUSG00000027852","ENSMUSG00000027864","ENSMUSG00000027875","ENSMUSG00000027
933","ENSMUSG00000027947","ENSMUSG00000027962","ENSMUSG00000027995","ENSMUS
G00000028028","ENSMUSG00000028082","ENSMUSG00000028223","ENSMUSG00000028309
","ENSMUSG00000028329","ENSMUSG00000028337","ENSMUSG00000028339","ENSMUSG00
000028343","ENSMUSG00000028410","ENSMUSG00000028581","ENSMUSG00000028597","
ENSMUSG00000028600","ENSMUSG00000028630","ENSMUSG00000028641","ENSMUSG00000
028646","ENSMUSG00000028673","ENSMUSG00000028756","ENSMUSG00000028766","ENS
MUSG00000028795","ENSMUSG00000028820","ENSMUSG00000028826","ENSMUSG00000028
859","ENSMUSG00000028862","ENSMUSG00000028878","ENSMUSG00000028885","ENSMUS
G00000028957","ENSMUSG00000028970","ENSMUSG00000029009","ENSMUSG00000029053
","ENSMUSG00000029090","ENSMUSG00000029119","ENSMUSG00000029177","ENSMUSG00
000029229","ENSMUSG00000029238","ENSMUSG00000029312","ENSMUSG00000029330","
ENSMUSG00000029468","ENSMUSG00000029471","ENSMUSG00000029570","ENSMUSG00000
029616","ENSMUSG00000029657","ENSMUSG00000029661","ENSMUSG00000029675","ENS
MUSG00000029687","ENSMUSG00000029762","ENSMUSG00000029778","ENSMUSG00000029
810","ENSMUSG00000029919","ENSMUSG00000029994","ENSMUSG00000030083","ENSMUS
G00000030087","ENSMUSG00000030096","ENSMUSG00000030103","ENSMUSG00000030172
","ENSMUSG00000030256","ENSMUSG00000030357","ENSMUSG00000030579","ENSMUSG00
000030678","ENSMUSG00000030681","ENSMUSG00000030701","ENSMUSG00000030711","
ENSMUSG00000030761","ENSMUSG00000030786","ENSMUSG00000030798","ENSMUSG00000
030921","ENSMUSG00000031004","ENSMUSG00000031016","ENSMUSG00000031024","ENS
MUSG00000031144","ENSMUSG00000031167","ENSMUSG00000031217","ENSMUSG00000031
221","ENSMUSG00000031232","ENSMUSG00000031239","ENSMUSG00000031274","ENSMUS
G00000031298","ENSMUSG00000031343","ENSMUSG00000031379","ENSMUSG00000031431
","ENSMUSG00000031442","ENSMUSG00000031467","ENSMUSG00000031479","ENSMUSG00

000031502","ENSMUSG00000031503","ENSMUSG00000031511","ENSMUSG00000031596","
ENSMUSG00000031740","ENSMUSG00000031762","ENSMUSG00000031765","ENSMUSG00000
031770","ENSMUSG00000031778","ENSMUSG00000031790","ENSMUSG00000031864","ENS
MUSG00000032000","ENSMUSG00000032010","ENSMUSG00000032086","ENSMUSG00000032
115","ENSMUSG00000032125","ENSMUSG00000032127","ENSMUSG00000032228","ENSMUS
G00000032348","ENSMUSG00000032349","ENSMUSG00000032363","ENSMUSG00000032369
","ENSMUSG00000032372","ENSMUSG00000032399","ENSMUSG00000032802","ENSMUSG00
000033032","ENSMUSG00000033082","ENSMUSG00000033159","ENSMUSG00000033191","
ENSMUSG00000033220","ENSMUSG00000033249","ENSMUSG00000033308","ENSMUSG00000
033318","ENSMUSG00000033460","ENSMUSG00000033499","ENSMUSG00000033581","ENS
MUSG00000033863","ENSMUSG00000033933","ENSMUSG00000034006","ENSMUSG00000034
007","ENSMUSG00000034116","ENSMUSG00000034154","ENSMUSG00000034157","ENSMUS
G00000034205","ENSMUSG00000034252","ENSMUSG00000034255","ENSMUSG00000034285
","ENSMUSG00000034341","ENSMUSG00000034349","ENSMUSG00000034403","ENSMUSG00
000034614","ENSMUSG00000034647","ENSMUSG00000034656","ENSMUSG00000034674","
ENSMUSG00000034707","ENSMUSG00000034795","ENSMUSG00000034832","ENSMUSG00000
034936","ENSMUSG00000034947","ENSMUSG00000034993","ENSMUSG00000034994","ENS
MUSG00000035004","ENSMUSG00000035064","ENSMUSG00000035206","ENSMUSG00000035
212","ENSMUSG00000035245","ENSMUSG00000035273","ENSMUSG00000035504","ENSMUS
G00000035513","ENSMUSG00000035772","ENSMUSG00000035824","ENSMUSG00000035828
","ENSMUSG00000035834","ENSMUSG00000035877","ENSMUSG00000035936","ENSMUSG00
000036040","ENSMUSG00000036273","ENSMUSG00000036275","ENSMUSG00000036402","
ENSMUSG00000036427","ENSMUSG00000036545","ENSMUSG00000036594","ENSMUSG00000
036615","ENSMUSG00000036667","ENSMUSG00000036862","ENSMUSG00000036887","ENS
MUSG00000036893","ENSMUSG00000036896","ENSMUSG00000036905","ENSMUSG00000036
934","ENSMUSG00000036944","ENSMUSG00000037003","ENSMUSG00000037058","ENSMUS
G00000037235","ENSMUSG00000037347","ENSMUSG00000037405","ENSMUSG00000037411
","ENSMUSG00000037455","ENSMUSG00000037461","ENSMUSG00000037475","ENSMUSG00
000037613","ENSMUSG00000037712","ENSMUSG00000037824","ENSMUSG00000037860","
ENSMUSG00000037892","ENSMUSG00000038007","ENSMUSG00000038143","ENSMUSG00000
038147","ENSMUSG00000038175","ENSMUSG00000038235","ENSMUSG00000038305","ENS
MUSG00000038393","ENSMUSG00000038521","ENSMUSG00000038642","ENSMUSG00000038
695","ENSMUSG00000038776","ENSMUSG00000039096","ENSMUSG00000039126","ENSMUS
G00000039159","ENSMUSG00000039176","ENSMUSG00000039254","ENSMUSG00000039328
","ENSMUSG00000039382","ENSMUSG00000039745","ENSMUSG00000039982","ENSMUSG00
000040093","ENSMUSG00000040097","ENSMUSG00000040170","ENSMUSG00000040174","
ENSMUSG00000040552","ENSMUSG00000040612","ENSMUSG00000040631","ENSMUSG00000
040713","ENSMUSG00000040723","ENSMUSG00000040751","ENSMUSG00000040820","ENS
MUSG00000041040","ENSMUSG00000041084","ENSMUSG00000041117","ENSMUSG00000041
355","ENSMUSG00000041417","ENSMUSG00000041515","ENSMUSG00000041560","ENSMUS
G00000041779","ENSMUSG00000041801","ENSMUSG00000041828","ENSMUSG00000041849
","ENSMUSG00000041926","ENSMUSG00000041936","ENSMUSG00000041992","ENSMUSG00
000042035","ENSMUSG00000042099","ENSMUSG00000042129","ENSMUSG00000042155","
ENSMUSG00000042251","ENSMUSG00000042286","ENSMUSG00000042426","ENSMUSG00000
042436","ENSMUSG00000042487","ENSMUSG00000042594","ENSMUSG00000042644","ENS
MUSG00000042677","ENSMUSG00000042688","ENSMUSG00000042712","ENSMUSG00000042
834","ENSMUSG00000043631","ENSMUSG00000044349","ENSMUSG00000044562","ENSMUS
G00000044583","ENSMUSG00000044949","ENSMUSG00000045193","ENSMUSG00000046230
","ENSMUSG00000046312","ENSMUSG00000046318","ENSMUSG00000046532","ENSMUSG00
000046688","ENSMUSG00000046743","ENSMUSG00000046805","ENSMUSG00000046994","
ENSMUSG00000047045","ENSMUSG00000047238","ENSMUSG00000047866","ENSMUSG00000
047945","ENSMUSG00000048126","ENSMUSG00000048249","ENSMUSG00000048330","ENS
MUSG00000048489","ENSMUSG00000048756","ENSMUSG00000049047","ENSMUSG00000049
076","ENSMUSG00000049804","ENSMUSG00000049878","ENSMUSG00000050103","ENSMUS
G00000050335","ENSMUSG00000050708","ENSMUSG00000050714","ENSMUSG00000051483
","ENSMUSG00000051506","ENSMUSG00000051579","ENSMUSG00000051864","ENSMUSG00
000052040","ENSMUSG00000052139","ENSMUSG00000052305","ENSMUSG00000052593","
ENSMUSG00000052760","ENSMUSG00000052812","ENSMUSG00000052915","ENSMUSG00000

```
053063","ENSMUSG00000053279","ENSMUSG00000053898","ENSMUSG00000054072","ENS
MUSG00000054404","ENSMUSG00000054509","ENSMUSG00000054612","ENSMUSG00000054
733","ENSMUSG00000054863","ENSMUSG00000055067","ENSMUSG00000055116","ENSMUS
G00000055541","ENSMUSG00000055866","ENSMUSG00000055980","ENSMUSG00000056228
","ENSMUSG00000056515","ENSMUSG00000056666","ENSMUSG00000056737","ENSMUSG00
000056749","ENSMUSG00000057137","ENSMUSG00000057193","ENSMUSG00000057406","
ENSMUSG00000057497","ENSMUSG00000057531","ENSMUSG00000057894","ENSMUSG00000
057897","ENSMUSG00000058135","ENSMUSG00000058586","ENSMUSG00000058587","ENS
MUSG00000058655","ENSMUSG00000058715","ENSMUSG00000058818","ENSMUSG00000059
013","ENSMUSG00000059182","ENSMUSG00000059498","ENSMUSG00000059714","ENSMUS
G00000059824","ENSMUSG00000059851","ENSMUSG00000059970","ENSMUSG00000060036
","ENSMUSG00000060373","ENSMUSG00000060586","ENSMUSG00000060671","ENSMUSG00
000060771","ENSMUSG00000060862","ENSMUSG00000060989","ENSMUSG00000061601","
ENSMUSG00000061740","ENSMUSG00000061838","ENSMUSG00000061950","ENSMUSG00000
062203","ENSMUSG00000063060","ENSMUSG00000063065","ENSMUSG00000063171","ENS
MUSG00000063275","ENSMUSG00000063415","ENSMUSG00000063576","ENSMUSG00000063
663","ENSMUSG00000064267","ENSMUSG00000064346","ENSMUSG00000066245","ENSMUS
G00000066640","ENSMUSG00000066687","ENSMUSG00000067274","ENSMUSG00000068290
","ENSMUSG00000068742","ENSMUSG00000068854","ENSMUSG00000069516","ENSMUSG00
000069833","ENSMUSG00000070031","ENSMUSG00000070436","ENSMUSG00000070544","
ENSMUSG00000071203","ENSMUSG00000071637","ENSMUSG00000071650","ENSMUSG00000
071714","ENSMUSG00000071715","ENSMUSG00000072949","ENSMUSG00000073409","ENS
MUSG00000073418","ENSMUSG00000073421","ENSMUSG00000073940","ENSMUSG00000074
264","ENSMUSG00000074748","ENSMUSG00000075284","ENSMUSG00000076435","ENSMUS
G00000076617","ENSMUSG00000078566","ENSMUSG00000078771","ENSMUSG00000078853
","ENSMUSG00000078922","ENSMUSG00000078945","ENSMUSG00000079037","ENSMUSG00
000079293","ENSMUSG00000079363","ENSMUSG00000079419","ENSMUSG00000079491","
ENSMUSG00000079499","ENSMUSG00000080316","ENSMUSG00000089715","ENSMUSG00000
090877","ENSMUSG00000092981","ENSMUSG00000095041","ENSMUSG00000096929","ENS
MUSG00000097415","ENSMUSG00000099875","ENSMUSG00000108218","ENSMUSG00000110
588","ENSMUSG00000111027","ENSMUSG00000114277")){
  tmp = attStats(AgeNC645CB[[i]])
  vec<-rep_len(i, length.out=11833)
  tmp$new_col <- vec
  SS<-rep_len(1, length.out=11833)
  tmp$SS <- SS
  attlist[[i]] = tmp
}

AgeNC645SS1CB = dplyr::bind_rows(attlist)
write.csv((AgeNC645SS1CB), "AgeNC645SS1CB", row.names = TRUE)
AgeNC645SS1CB_C = AgeNC645SS1CB[AgeNC645SS1CB$decision =="Confirmed",]
write.csv((AgeNC645SS1CB_C), "AgeNC645SS1CB_C", row.names = TRUE)
AgeNC645SS1CB_noR = AgeNC645SS1CB[AgeNC645SS1CB$decision !="Rejected",]
write.csv((AgeNC645SS1CB_noR), "AgeNC645SS1CB_noR", row.names = TRUE)


AgeNC645CB = list()
for (i in
c("ENSMUSG00000000056","ENSMUSG00000000154","ENSMUSG00000000184","ENSMUSG00
000000290","ENSMUSG00000000682","ENSMUSG00000000823","ENSMUSG00000001119","
ENSMUSG00000001143","ENSMUSG00000001229","ENSMUSG00000001506","ENSMUSG00000
001663","ENSMUSG00000001774","ENSMUSG00000001930","ENSMUSG00000002017","ENS
MUSG00000002289","ENSMUSG00000002763","ENSMUSG00000002900","ENSMUSG00000002
985","ENSMUSG00000002996","ENSMUSG00000003031","ENSMUSG00000003154","ENSMUS
G00000003283","ENSMUSG00000003348","ENSMUSG00000003458","ENSMUSG00000003541
","ENSMUSG00000003617","ENSMUSG00000003731","ENSMUSG00000003814","ENSMUSG00
000003849","ENSMUSG00000003949","ENSMUSG00000004105","ENSMUSG00000004460","
```

ENSMUSG00000004609","ENSMUSG00000004637","ENSMUSG00000005107","ENSMUSG00000
005397","ENSMUSG00000005417","ENSMUSG00000005447","ENSMUSG00000005686","ENS
MUSG00000005936","ENSMUSG00000006127","ENSMUSG00000006373","ENSMUSG00000006
386","ENSMUSG00000006611","ENSMUSG00000007617","ENSMUSG00000007777","ENSMUS
G00000007891","ENSMUSG00000008540","ENSMUSG00000008845","ENSMUSG00000009406
","ENSMUSG00000010554","ENSMUSG00000010651","ENSMUSG00000011256","ENSMUSG00
000013150","ENSMUSG00000013275","ENSMUSG00000014361","ENSMUSG00000014776","
ENSMUSG00000015189","ENSMUSG00000015340","ENSMUSG00000015468","ENSMUSG00000
015839","ENSMUSG00000015950","ENSMUSG00000016206","ENSMUSG00000016256","ENS
MUSG00000016946","ENSMUSG00000017132","ENSMUSG00000017707","ENSMUSG00000017
754","ENSMUSG00000017774","ENSMUSG00000018042","ENSMUSG00000018160","ENSMUS
G00000018217","ENSMUSG00000018415","ENSMUSG00000018427","ENSMUSG00000018501
","ENSMUSG00000018574","ENSMUSG00000018593","ENSMUSG00000018654","ENSMUSG00
000018666","ENSMUSG00000018774","ENSMUSG00000019055","ENSMUSG00000019843","
ENSMUSG00000019846","ENSMUSG00000019872","ENSMUSG00000019916","ENSMUSG00000
019923","ENSMUSG00000020009","ENSMUSG00000020034","ENSMUSG00000020038","ENS
MUSG00000020044","ENSMUSG00000020048","ENSMUSG00000020053","ENSMUSG00000020
111","ENSMUSG00000020120","ENSMUSG00000020123","ENSMUSG00000020143","ENSMUS
G00000020227","ENSMUSG00000020241","ENSMUSG00000020257","ENSMUSG00000020277
","ENSMUSG00000020288","ENSMUSG00000020459","ENSMUSG00000020467","ENSMUSG00
000020472","ENSMUSG00000020571","ENSMUSG00000020594","ENSMUSG00000020653","
ENSMUSG00000020661","ENSMUSG00000020674","ENSMUSG00000020810","ENSMUSG00000
020889","ENSMUSG00000020893","ENSMUSG00000020935","ENSMUSG00000020954","ENS
MUSG00000021003","ENSMUSG00000021025","ENSMUSG00000021037","ENSMUSG00000021
091","ENSMUSG00000021188","ENSMUSG00000021190","ENSMUSG00000021242","ENSMUS
G00000021262","ENSMUSG00000021306","ENSMUSG00000021508","ENSMUSG00000021559
","ENSMUSG00000021775","ENSMUSG00000021814","ENSMUSG00000021903","ENSMUSG00
000021939","ENSMUSG00000021998","ENSMUSG00000022035","ENSMUSG00000022091","
ENSMUSG00000022098","ENSMUSG00000022146","ENSMUSG00000022148","ENSMUSG00000
022186","ENSMUSG00000022197","ENSMUSG00000022270","ENSMUSG00000022372","ENS
MUSG00000022389","ENSMUSG00000022415","ENSMUSG00000022440","ENSMUSG00000022
472","ENSMUSG00000022488","ENSMUSG00000022623","ENSMUSG00000022656","ENSMUS
G00000022665","ENSMUSG00000022684","ENSMUSG00000022797","ENSMUSG00000022816
","ENSMUSG00000022831","ENSMUSG00000022848","ENSMUSG00000022912","ENSMUSG00
000022952","ENSMUSG00000022999","ENSMUSG00000023055","ENSMUSG00000023249","
ENSMUSG00000023328","ENSMUSG00000023367","ENSMUSG00000023952","ENSMUSG00000
023960","ENSMUSG00000024132","ENSMUSG00000024160","ENSMUSG00000024164","ENS
MUSG00000024180","ENSMUSG00000024190","ENSMUSG00000024222","ENSMUSG00000024
299","ENSMUSG00000024300","ENSMUSG00000024421","ENSMUSG00000024451","ENSMUS
G00000024501","ENSMUSG00000024538","ENSMUSG00000024576","ENSMUSG00000024610
","ENSMUSG00000024654","ENSMUSG00000024679","ENSMUSG00000024736","ENSMUSG00
000024810","ENSMUSG00000024885","ENSMUSG00000024900","ENSMUSG00000024965","
ENSMUSG00000024966","ENSMUSG00000024975","ENSMUSG00000025017","ENSMUSG00000
025037","ENSMUSG00000025133","ENSMUSG00000025158","ENSMUSG00000025176","ENS
MUSG00000025262","ENSMUSG00000025265","ENSMUSG00000025270","ENSMUSG00000025
358","ENSMUSG00000025511","ENSMUSG00000025512","ENSMUSG00000025584","ENSMUS
G00000025586","ENSMUSG00000025743","ENSMUSG00000025821","ENSMUSG00000025885
","ENSMUSG00000025888","ENSMUSG00000025980","ENSMUSG00000025981","ENSMUSG00
000025986","ENSMUSG00000026003","ENSMUSG00000026042","ENSMUSG00000026043","
ENSMUSG00000026074","ENSMUSG00000026077","ENSMUSG00000026082","ENSMUSG00000
026234","ENSMUSG00000026288","ENSMUSG00000026303","ENSMUSG00000026395","ENS
MUSG00000026478","ENSMUSG00000026480","ENSMUSG00000026548","ENSMUSG00000026
556","ENSMUSG00000026566","ENSMUSG00000026608","ENSMUSG00000026672","ENSMUS
G00000026728","ENSMUSG00000026739","ENSMUSG00000026786","ENSMUSG00000026837
","ENSMUSG00000026864","ENSMUSG00000026872","ENSMUSG00000026880","ENSMUSG00
000026956","ENSMUSG00000026979","ENSMUSG00000027204","ENSMUSG00000027248","
ENSMUSG00000027274","ENSMUSG00000027330","ENSMUSG00000027412","ENSMUSG00000
027435","ENSMUSG00000027635","ENSMUSG00000027669","ENSMUSG00000027843","ENS

MUSG00000027852","ENSMUSG00000027864","ENSMUSG00000027875","ENSMUSG00000027
933","ENSMUSG00000027947","ENSMUSG00000027962","ENSMUSG00000027995","ENSMUS
G00000028028","ENSMUSG00000028082","ENSMUSG00000028223","ENSMUSG00000028309
","ENSMUSG00000028329","ENSMUSG00000028337","ENSMUSG00000028339","ENSMUSG00
000028343","ENSMUSG00000028410","ENSMUSG00000028581","ENSMUSG00000028597","
ENSMUSG00000028600","ENSMUSG00000028630","ENSMUSG00000028641","ENSMUSG00000
028646","ENSMUSG00000028673","ENSMUSG00000028756","ENSMUSG00000028766","ENS
MUSG00000028795","ENSMUSG00000028820","ENSMUSG00000028826","ENSMUSG00000028
859","ENSMUSG00000028862","ENSMUSG00000028878","ENSMUSG00000028885","ENSMUS
G00000028957","ENSMUSG00000028970","ENSMUSG00000029009","ENSMUSG00000029053
","ENSMUSG00000029090","ENSMUSG00000029119","ENSMUSG00000029177","ENSMUSG00
000029229","ENSMUSG00000029238","ENSMUSG00000029312","ENSMUSG00000029330","
ENSMUSG00000029468","ENSMUSG00000029471","ENSMUSG00000029570","ENSMUSG00000
029616","ENSMUSG00000029657","ENSMUSG00000029661","ENSMUSG00000029675","ENS
MUSG00000029687","ENSMUSG00000029762","ENSMUSG00000029778","ENSMUSG00000029
810","ENSMUSG00000029919","ENSMUSG00000029994","ENSMUSG00000030083","ENSMUS
G00000030087","ENSMUSG00000030096","ENSMUSG00000030103","ENSMUSG00000030172
","ENSMUSG00000030256","ENSMUSG00000030357","ENSMUSG00000030579","ENSMUSG00
000030678","ENSMUSG00000030681","ENSMUSG00000030701","ENSMUSG00000030711","
ENSMUSG00000030761","ENSMUSG00000030786","ENSMUSG00000030798","ENSMUSG00000
030921","ENSMUSG00000031004","ENSMUSG00000031016","ENSMUSG00000031024","ENS
MUSG00000031144","ENSMUSG00000031167","ENSMUSG00000031217","ENSMUSG00000031
221","ENSMUSG00000031232","ENSMUSG00000031239","ENSMUSG00000031274","ENSMUS
G00000031298","ENSMUSG00000031343","ENSMUSG00000031379","ENSMUSG00000031431
","ENSMUSG00000031442","ENSMUSG00000031467","ENSMUSG00000031479","ENSMUSG00
000031502","ENSMUSG00000031503","ENSMUSG00000031511","ENSMUSG00000031596","
ENSMUSG00000031740","ENSMUSG00000031762","ENSMUSG00000031765","ENSMUSG00000
031770","ENSMUSG00000031778","ENSMUSG00000031790","ENSMUSG00000031864","ENS
MUSG00000032000","ENSMUSG00000032010","ENSMUSG00000032086","ENSMUSG00000032
115","ENSMUSG00000032125","ENSMUSG00000032127","ENSMUSG00000032228","ENSMUS
G00000032348","ENSMUSG00000032349","ENSMUSG00000032363","ENSMUSG00000032369
","ENSMUSG00000032372","ENSMUSG00000032399","ENSMUSG00000032802","ENSMUSG00
000033032","ENSMUSG00000033082","ENSMUSG00000033159","ENSMUSG00000033191","
ENSMUSG00000033220","ENSMUSG00000033249","ENSMUSG00000033308","ENSMUSG00000
033318","ENSMUSG00000033460","ENSMUSG00000033499","ENSMUSG00000033581","ENS
MUSG00000033863","ENSMUSG00000033933","ENSMUSG00000034006","ENSMUSG00000034
007","ENSMUSG00000034116","ENSMUSG00000034154","ENSMUSG00000034157","ENSMUS
G00000034205","ENSMUSG00000034252","ENSMUSG00000034255","ENSMUSG00000034285
","ENSMUSG00000034341","ENSMUSG00000034349","ENSMUSG00000034403","ENSMUSG00
000034614","ENSMUSG00000034647","ENSMUSG00000034656","ENSMUSG00000034674","
ENSMUSG00000034707","ENSMUSG00000034795","ENSMUSG00000034832","ENSMUSG00000
034936","ENSMUSG00000034947","ENSMUSG00000034993","ENSMUSG00000034994","ENS
MUSG00000035004","ENSMUSG00000035064","ENSMUSG00000035206","ENSMUSG00000035
212","ENSMUSG00000035245","ENSMUSG00000035273","ENSMUSG00000035504","ENSMUS
G00000035513","ENSMUSG00000035772","ENSMUSG00000035824","ENSMUSG00000035828
","ENSMUSG00000035834","ENSMUSG00000035877","ENSMUSG00000035936","ENSMUSG00
000036040","ENSMUSG00000036273","ENSMUSG00000036275","ENSMUSG00000036402","
ENSMUSG00000036427","ENSMUSG00000036545","ENSMUSG00000036594","ENSMUSG00000
036615","ENSMUSG00000036667","ENSMUSG00000036862","ENSMUSG00000036887","ENS
MUSG00000036893","ENSMUSG00000036896","ENSMUSG00000036905","ENSMUSG00000036
934","ENSMUSG00000036944","ENSMUSG00000037003","ENSMUSG00000037058","ENSMUS
G00000037235","ENSMUSG00000037347","ENSMUSG00000037405","ENSMUSG00000037411
","ENSMUSG00000037455","ENSMUSG00000037461","ENSMUSG00000037475","ENSMUSG00
000037613","ENSMUSG00000037712","ENSMUSG00000037824","ENSMUSG00000037860","
ENSMUSG00000037892","ENSMUSG00000038007","ENSMUSG00000038143","ENSMUSG00000
038147","ENSMUSG00000038175","ENSMUSG00000038235","ENSMUSG00000038305","ENS
MUSG00000038393","ENSMUSG00000038521","ENSMUSG00000038642","ENSMUSG00000038
695","ENSMUSG00000038776","ENSMUSG00000039096","ENSMUSG00000039126","ENSMUS

```
G00000039159","ENSMUSG00000039176","ENSMUSG00000039254","ENSMUSG00000039328
","ENSMUSG00000039382","ENSMUSG00000039745","ENSMUSG00000039982","ENSMUSG00
000040093","ENSMUSG00000040097","ENSMUSG00000040170","ENSMUSG00000040174","
ENSMUSG00000040552","ENSMUSG00000040612","ENSMUSG00000040631","ENSMUSG00000
040713","ENSMUSG00000040723","ENSMUSG00000040751","ENSMUSG00000040820","ENS
MUSG00000041040","ENSMUSG00000041084","ENSMUSG00000041117","ENSMUSG00000041
355","ENSMUSG00000041417","ENSMUSG00000041515","ENSMUSG00000041560","ENSMUS
G00000041779","ENSMUSG00000041801","ENSMUSG00000041828","ENSMUSG00000041849
","ENSMUSG00000041926","ENSMUSG00000041936","ENSMUSG00000041992","ENSMUSG00
000042035","ENSMUSG00000042099","ENSMUSG00000042129","ENSMUSG00000042155","
ENSMUSG00000042251","ENSMUSG00000042286","ENSMUSG00000042426","ENSMUSG00000
042436","ENSMUSG00000042487","ENSMUSG00000042594","ENSMUSG00000042644","ENS
MUSG00000042677","ENSMUSG00000042688","ENSMUSG00000042712","ENSMUSG00000042
834","ENSMUSG00000043631","ENSMUSG00000044349","ENSMUSG00000044562","ENSMUS
G00000044583","ENSMUSG00000044949","ENSMUSG00000045193","ENSMUSG00000046230
","ENSMUSG00000046312","ENSMUSG00000046318","ENSMUSG00000046532","ENSMUSG00
000046688","ENSMUSG00000046743","ENSMUSG00000046805","ENSMUSG00000046994","
ENSMUSG00000047045","ENSMUSG00000047238","ENSMUSG00000047866","ENSMUSG00000
047945","ENSMUSG00000048126","ENSMUSG00000048249","ENSMUSG00000048330","ENS
MUSG00000048489","ENSMUSG00000048756","ENSMUSG00000049047","ENSMUSG00000049
076","ENSMUSG00000049804","ENSMUSG00000049878","ENSMUSG00000050103","ENSMUS
G00000050335","ENSMUSG00000050708","ENSMUSG00000050714","ENSMUSG00000051483
","ENSMUSG00000051506","ENSMUSG00000051579","ENSMUSG00000051864","ENSMUSG00
000052040","ENSMUSG00000052139","ENSMUSG00000052305","ENSMUSG00000052593","
ENSMUSG00000052760","ENSMUSG00000052812","ENSMUSG00000052915","ENSMUSG00000
053063","ENSMUSG00000053279","ENSMUSG00000053898","ENSMUSG00000054072","ENS
MUSG00000054404","ENSMUSG00000054509","ENSMUSG00000054612","ENSMUSG00000054
733","ENSMUSG00000054863","ENSMUSG00000055067","ENSMUSG00000055116","ENSMUS
G00000055541","ENSMUSG00000055866","ENSMUSG00000055980","ENSMUSG00000056228
","ENSMUSG00000056515","ENSMUSG00000056666","ENSMUSG00000056737","ENSMUSG00
000056749","ENSMUSG00000057137","ENSMUSG00000057193","ENSMUSG00000057406","
ENSMUSG00000057497","ENSMUSG00000057531","ENSMUSG00000057894","ENSMUSG00000
057897","ENSMUSG00000058135","ENSMUSG00000058586","ENSMUSG00000058587","ENS
MUSG00000058655","ENSMUSG00000058715","ENSMUSG00000058818","ENSMUSG00000059
013","ENSMUSG00000059182","ENSMUSG00000059498","ENSMUSG00000059714","ENSMUS
G00000059824","ENSMUSG00000059851","ENSMUSG00000059970","ENSMUSG00000060036
","ENSMUSG00000060373","ENSMUSG00000060586","ENSMUSG00000060671","ENSMUSG00
000060771","ENSMUSG00000060862","ENSMUSG00000060989","ENSMUSG00000061601","
ENSMUSG00000061740","ENSMUSG00000061838","ENSMUSG00000061950","ENSMUSG00000
062203","ENSMUSG00000063060","ENSMUSG00000063065","ENSMUSG00000063171","ENS
MUSG00000063275","ENSMUSG00000063415","ENSMUSG00000063576","ENSMUSG00000063
663","ENSMUSG00000064267","ENSMUSG00000064346","ENSMUSG00000066245","ENSMUS
G00000066640","ENSMUSG00000066687","ENSMUSG00000067274","ENSMUSG00000068290
","ENSMUSG00000068742","ENSMUSG00000068854","ENSMUSG00000069516","ENSMUSG00
000069833","ENSMUSG00000070031","ENSMUSG00000070436","ENSMUSG00000070544","
ENSMUSG00000071203","ENSMUSG00000071637","ENSMUSG00000071650","ENSMUSG00000
071714","ENSMUSG00000071715","ENSMUSG00000072949","ENSMUSG00000073409","ENS
MUSG00000073418","ENSMUSG00000073421","ENSMUSG00000073940","ENSMUSG00000074
264","ENSMUSG00000074748","ENSMUSG00000075284","ENSMUSG00000076435","ENSMUS
G00000076617","ENSMUSG00000078566","ENSMUSG00000078771","ENSMUSG00000078853
","ENSMUSG00000078922","ENSMUSG00000078945","ENSMUSG00000079037","ENSMUSG00
000079293","ENSMUSG00000079363","ENSMUSG00000079419","ENSMUSG00000079491","
ENSMUSG00000079499","ENSMUSG00000080316","ENSMUSG00000089715","ENSMUSG00000
090877","ENSMUSG00000092981","ENSMUSG00000095041","ENSMUSG00000096929","ENS
MUSG00000097415","ENSMUSG00000099875","ENSMUSG00000108218","ENSMUSG00000110
588","ENSMUSG00000111027","ENSMUSG00000114277")){
  print(i)
  set.seed(2)
```

```
  data = N
  y=data.frame(data[,i])
  y=y[,1]
  x=data.frame(data[,colnames(data)!= i])
  output <- Boruta(y = y, x=x, doTrace = 2)
  AgeNC645CB[[i]] = output
}
Sys.time()

attlist = list()
for (i in
c("ENSMUSG00000000056","ENSMUSG00000000154","ENSMUSG00000000184","ENSMUSG00
000000290","ENSMUSG00000000682","ENSMUSG00000000823","ENSMUSG00000001119","
ENSMUSG00000001143","ENSMUSG00000001229","ENSMUSG00000001506","ENSMUSG00000
001663","ENSMUSG00000001774","ENSMUSG00000001930","ENSMUSG00000002017","ENS
MUSG00000002289","ENSMUSG00000002763","ENSMUSG00000002900","ENSMUSG00000002
985","ENSMUSG00000002996","ENSMUSG00000003031","ENSMUSG00000003154","ENSMUS
G00000003283","ENSMUSG00000003348","ENSMUSG00000003458","ENSMUSG00000003541
","ENSMUSG00000003617","ENSMUSG00000003731","ENSMUSG00000003814","ENSMUSG00
000003849","ENSMUSG00000003949","ENSMUSG00000004105","ENSMUSG00000004460","
ENSMUSG00000004609","ENSMUSG00000004637","ENSMUSG00000005107","ENSMUSG00000
005397","ENSMUSG00000005417","ENSMUSG00000005447","ENSMUSG00000005686","ENS
MUSG00000005936","ENSMUSG00000006127","ENSMUSG00000006373","ENSMUSG00000006
386","ENSMUSG00000006611","ENSMUSG00000007617","ENSMUSG00000007777","ENSMUS
G00000007891","ENSMUSG00000008540","ENSMUSG00000008845","ENSMUSG00000009406
","ENSMUSG00000010554","ENSMUSG00000010651","ENSMUSG00000011256","ENSMUSG00
000013150","ENSMUSG00000013275","ENSMUSG00000014361","ENSMUSG00000014776","
ENSMUSG00000015189","ENSMUSG00000015340","ENSMUSG00000015468","ENSMUSG00000
015839","ENSMUSG00000015950","ENSMUSG00000016206","ENSMUSG00000016256","ENS
MUSG00000016946","ENSMUSG00000017132","ENSMUSG00000017707","ENSMUSG00000017
754","ENSMUSG00000017774","ENSMUSG00000018042","ENSMUSG00000018160","ENSMUS
G00000018217","ENSMUSG00000018415","ENSMUSG00000018427","ENSMUSG00000018501
","ENSMUSG00000018574","ENSMUSG00000018593","ENSMUSG00000018654","ENSMUSG00
000018666","ENSMUSG00000018774","ENSMUSG00000019055","ENSMUSG00000019843","
ENSMUSG00000019846","ENSMUSG00000019872","ENSMUSG00000019916","ENSMUSG00000
019923","ENSMUSG00000020009","ENSMUSG00000020034","ENSMUSG00000020038","ENS
MUSG00000020044","ENSMUSG00000020048","ENSMUSG00000020053","ENSMUSG00000020
111","ENSMUSG00000020120","ENSMUSG00000020123","ENSMUSG00000020143","ENSMUS
G00000020227","ENSMUSG00000020241","ENSMUSG00000020257","ENSMUSG00000020277
","ENSMUSG00000020288","ENSMUSG00000020459","ENSMUSG00000020467","ENSMUSG00
000020472","ENSMUSG00000020571","ENSMUSG00000020594","ENSMUSG00000020653","
ENSMUSG00000020661","ENSMUSG00000020674","ENSMUSG00000020810","ENSMUSG00000
020889","ENSMUSG00000020893","ENSMUSG00000020935","ENSMUSG00000020954","ENS
MUSG00000021003","ENSMUSG00000021025","ENSMUSG00000021037","ENSMUSG00000021
091","ENSMUSG00000021188","ENSMUSG00000021190","ENSMUSG00000021242","ENSMUS
G00000021262","ENSMUSG00000021306","ENSMUSG00000021508","ENSMUSG00000021559
","ENSMUSG00000021775","ENSMUSG00000021814","ENSMUSG00000021903","ENSMUSG00
000021939","ENSMUSG00000021998","ENSMUSG00000022035","ENSMUSG00000022091","
ENSMUSG00000022098","ENSMUSG00000022146","ENSMUSG00000022148","ENSMUSG00000
022186","ENSMUSG00000022197","ENSMUSG00000022270","ENSMUSG00000022372","ENS
MUSG00000022389","ENSMUSG00000022415","ENSMUSG00000022440","ENSMUSG00000022
472","ENSMUSG00000022488","ENSMUSG00000022623","ENSMUSG00000022656","ENSMUS
G00000022665","ENSMUSG00000022684","ENSMUSG00000022797","ENSMUSG00000022816
","ENSMUSG00000022831","ENSMUSG00000022848","ENSMUSG00000022912","ENSMUSG00
000022952","ENSMUSG00000022999","ENSMUSG00000023055","ENSMUSG00000023249","
ENSMUSG00000023328","ENSMUSG00000023367","ENSMUSG00000023952","ENSMUSG00000
023960","ENSMUSG00000024132","ENSMUSG00000024160","ENSMUSG00000024164","ENS
MUSG00000024180","ENSMUSG00000024190","ENSMUSG00000024222","ENSMUSG00000024
```

299","ENSMUSG00000024300","ENSMUSG00000024421","ENSMUSG00000024451","ENSMUS
G00000024501","ENSMUSG00000024538","ENSMUSG00000024576","ENSMUSG00000024610
","ENSMUSG00000024654","ENSMUSG00000024679","ENSMUSG00000024736","ENSMUSG00
000024810","ENSMUSG00000024885","ENSMUSG00000024900","ENSMUSG00000024965","
ENSMUSG00000024966","ENSMUSG00000024975","ENSMUSG00000025017","ENSMUSG00000
025037","ENSMUSG00000025133","ENSMUSG00000025158","ENSMUSG00000025176","ENS
MUSG00000025262","ENSMUSG00000025265","ENSMUSG00000025270","ENSMUSG00000025
358","ENSMUSG00000025511","ENSMUSG00000025512","ENSMUSG00000025584","ENSMUS
G00000025586","ENSMUSG00000025743","ENSMUSG00000025821","ENSMUSG00000025885
","ENSMUSG00000025888","ENSMUSG00000025980","ENSMUSG00000025981","ENSMUSG00
000025986","ENSMUSG00000026003","ENSMUSG00000026042","ENSMUSG00000026043","
ENSMUSG00000026074","ENSMUSG00000026077","ENSMUSG00000026082","ENSMUSG00000
026234","ENSMUSG00000026288","ENSMUSG00000026303","ENSMUSG00000026395","ENS
MUSG00000026478","ENSMUSG00000026480","ENSMUSG00000026548","ENSMUSG00000026
556","ENSMUSG00000026566","ENSMUSG00000026608","ENSMUSG00000026672","ENSMUS
G00000026728","ENSMUSG00000026739","ENSMUSG00000026786","ENSMUSG00000026837
","ENSMUSG00000026864","ENSMUSG00000026872","ENSMUSG00000026880","ENSMUSG00
000026956","ENSMUSG00000026979","ENSMUSG00000027204","ENSMUSG00000027248","
ENSMUSG00000027274","ENSMUSG00000027330","ENSMUSG00000027412","ENSMUSG00000
027435","ENSMUSG00000027635","ENSMUSG00000027669","ENSMUSG00000027843","ENS
MUSG00000027852","ENSMUSG00000027864","ENSMUSG00000027875","ENSMUSG00000027
933","ENSMUSG00000027947","ENSMUSG00000027962","ENSMUSG00000027995","ENSMUS
G00000028028","ENSMUSG00000028082","ENSMUSG00000028223","ENSMUSG00000028309
","ENSMUSG00000028329","ENSMUSG00000028337","ENSMUSG00000028339","ENSMUSG00
000028343","ENSMUSG00000028410","ENSMUSG00000028581","ENSMUSG00000028597","
ENSMUSG00000028600","ENSMUSG00000028630","ENSMUSG00000028641","ENSMUSG00000
028646","ENSMUSG00000028673","ENSMUSG00000028756","ENSMUSG00000028766","ENS
MUSG00000028795","ENSMUSG00000028820","ENSMUSG00000028826","ENSMUSG00000028
859","ENSMUSG00000028862","ENSMUSG00000028878","ENSMUSG00000028885","ENSMUS
G00000028957","ENSMUSG00000028970","ENSMUSG00000029009","ENSMUSG00000029053
","ENSMUSG00000029090","ENSMUSG00000029119","ENSMUSG00000029177","ENSMUSG00
000029229","ENSMUSG00000029238","ENSMUSG00000029312","ENSMUSG00000029330","
ENSMUSG00000029468","ENSMUSG00000029471","ENSMUSG00000029570","ENSMUSG00000
029616","ENSMUSG00000029657","ENSMUSG00000029661","ENSMUSG00000029675","ENS
MUSG00000029687","ENSMUSG00000029762","ENSMUSG00000029778","ENSMUSG00000029
810","ENSMUSG00000029919","ENSMUSG00000029994","ENSMUSG00000030083","ENSMUS
G00000030087","ENSMUSG00000030096","ENSMUSG00000030103","ENSMUSG00000030172
","ENSMUSG00000030256","ENSMUSG00000030357","ENSMUSG00000030579","ENSMUSG00
000030678","ENSMUSG00000030681","ENSMUSG00000030701","ENSMUSG00000030711","
ENSMUSG00000030761","ENSMUSG00000030786","ENSMUSG00000030798","ENSMUSG00000
030921","ENSMUSG00000031004","ENSMUSG00000031016","ENSMUSG00000031024","ENS
MUSG00000031144","ENSMUSG00000031167","ENSMUSG00000031217","ENSMUSG00000031
221","ENSMUSG00000031232","ENSMUSG00000031239","ENSMUSG00000031274","ENSMUS
G00000031298","ENSMUSG00000031343","ENSMUSG00000031379","ENSMUSG00000031431
","ENSMUSG00000031442","ENSMUSG00000031467","ENSMUSG00000031479","ENSMUSG00
000031502","ENSMUSG00000031503","ENSMUSG00000031511","ENSMUSG00000031596","
ENSMUSG00000031740","ENSMUSG00000031762","ENSMUSG00000031765","ENSMUSG00000
031770","ENSMUSG00000031778","ENSMUSG00000031790","ENSMUSG00000031864","ENS
MUSG00000032000","ENSMUSG00000032010","ENSMUSG00000032086","ENSMUSG00000032
115","ENSMUSG00000032125","ENSMUSG00000032127","ENSMUSG00000032228","ENSMUS
G00000032348","ENSMUSG00000032349","ENSMUSG00000032363","ENSMUSG00000032369
","ENSMUSG00000032372","ENSMUSG00000032399","ENSMUSG00000032802","ENSMUSG00
000033032","ENSMUSG00000033082","ENSMUSG00000033159","ENSMUSG00000033191","
ENSMUSG00000033220","ENSMUSG00000033249","ENSMUSG00000033308","ENSMUSG00000
033318","ENSMUSG00000033460","ENSMUSG00000033499","ENSMUSG00000033581","ENS
MUSG00000033863","ENSMUSG00000033933","ENSMUSG00000034006","ENSMUSG00000034
007","ENSMUSG00000034116","ENSMUSG00000034154","ENSMUSG00000034157","ENSMUS
G00000034205","ENSMUSG00000034252","ENSMUSG00000034255","ENSMUSG00000034285

","ENSMUSG00000034341","ENSMUSG00000034349","ENSMUSG00000034403","ENSMUSG00000034614","ENSMUSG00000034647","ENSMUSG00000034656","ENSMUSG00000034674","ENSMUSG00000034707","ENSMUSG00000034795","ENSMUSG00000034832","ENSMUSG00000034936","ENSMUSG00000034947","ENSMUSG00000034993","ENSMUSG00000034994","ENSMUSG00000035004","ENSMUSG00000035064","ENSMUSG00000035206","ENSMUSG00000035212","ENSMUSG00000035245","ENSMUSG00000035273","ENSMUSG00000035504","ENSMUSG00000035513","ENSMUSG00000035772","ENSMUSG00000035824","ENSMUSG00000035828","ENSMUSG00000035834","ENSMUSG00000035877","ENSMUSG00000035936","ENSMUSG00000036040","ENSMUSG00000036273","ENSMUSG00000036275","ENSMUSG00000036402","ENSMUSG00000036427","ENSMUSG00000036545","ENSMUSG00000036594","ENSMUSG00000036615","ENSMUSG00000036667","ENSMUSG00000036862","ENSMUSG00000036887","ENSMUSG00000036893","ENSMUSG00000036896","ENSMUSG00000036905","ENSMUSG00000036934","ENSMUSG00000036944","ENSMUSG00000037003","ENSMUSG00000037058","ENSMUSG00000037235","ENSMUSG00000037347","ENSMUSG00000037405","ENSMUSG00000037411","ENSMUSG00000037455","ENSMUSG00000037461","ENSMUSG00000037475","ENSMUSG00000037613","ENSMUSG00000037712","ENSMUSG00000037824","ENSMUSG00000037860","ENSMUSG00000037892","ENSMUSG00000038007","ENSMUSG00000038143","ENSMUSG00000038147","ENSMUSG00000038175","ENSMUSG00000038235","ENSMUSG00000038305","ENSMUSG00000038393","ENSMUSG00000038521","ENSMUSG00000038642","ENSMUSG00000038695","ENSMUSG00000038776","ENSMUSG00000039096","ENSMUSG00000039126","ENSMUSG00000039159","ENSMUSG00000039176","ENSMUSG00000039254","ENSMUSG00000039328","ENSMUSG00000039382","ENSMUSG00000039745","ENSMUSG00000039982","ENSMUSG00000040093","ENSMUSG00000040097","ENSMUSG00000040170","ENSMUSG00000040174","ENSMUSG00000040552","ENSMUSG00000040612","ENSMUSG00000040631","ENSMUSG00000040713","ENSMUSG00000040723","ENSMUSG00000040751","ENSMUSG00000040820","ENSMUSG00000041040","ENSMUSG00000041084","ENSMUSG00000041117","ENSMUSG00000041355","ENSMUSG00000041417","ENSMUSG00000041515","ENSMUSG00000041560","ENSMUSG00000041779","ENSMUSG00000041801","ENSMUSG00000041828","ENSMUSG00000041849","ENSMUSG00000041926","ENSMUSG00000041936","ENSMUSG00000041992","ENSMUSG00000042035","ENSMUSG00000042099","ENSMUSG00000042129","ENSMUSG00000042155","ENSMUSG00000042251","ENSMUSG00000042286","ENSMUSG00000042426","ENSMUSG00000042436","ENSMUSG00000042487","ENSMUSG00000042594","ENSMUSG00000042644","ENSMUSG00000042677","ENSMUSG00000042688","ENSMUSG00000042712","ENSMUSG00000042834","ENSMUSG00000043631","ENSMUSG00000044349","ENSMUSG00000044562","ENSMUSG00000044583","ENSMUSG00000044949","ENSMUSG00000045193","ENSMUSG00000046230","ENSMUSG00000046312","ENSMUSG00000046318","ENSMUSG00000046532","ENSMUSG00000046688","ENSMUSG00000046743","ENSMUSG00000046805","ENSMUSG00000046994","ENSMUSG00000047045","ENSMUSG00000047238","ENSMUSG00000047866","ENSMUSG00000047945","ENSMUSG00000048126","ENSMUSG00000048249","ENSMUSG00000048330","ENSMUSG00000048489","ENSMUSG00000048756","ENSMUSG00000049047","ENSMUSG00000049076","ENSMUSG00000049804","ENSMUSG00000049878","ENSMUSG00000050103","ENSMUSG00000050335","ENSMUSG00000050708","ENSMUSG00000050714","ENSMUSG00000051483","ENSMUSG00000051506","ENSMUSG00000051579","ENSMUSG00000051864","ENSMUSG00000052040","ENSMUSG00000052139","ENSMUSG00000052305","ENSMUSG00000052593","ENSMUSG00000052760","ENSMUSG00000052812","ENSMUSG00000052915","ENSMUSG00000053063","ENSMUSG00000053279","ENSMUSG00000053898","ENSMUSG00000054072","ENSMUSG00000054404","ENSMUSG00000054509","ENSMUSG00000054612","ENSMUSG00000054733","ENSMUSG00000054863","ENSMUSG00000055067","ENSMUSG00000055116","ENSMUSG00000055541","ENSMUSG00000055866","ENSMUSG00000055980","ENSMUSG00000056228","ENSMUSG00000056515","ENSMUSG00000056666","ENSMUSG00000056737","ENSMUSG00000056749","ENSMUSG00000057137","ENSMUSG00000057193","ENSMUSG00000057406","ENSMUSG00000057497","ENSMUSG00000057531","ENSMUSG00000057894","ENSMUSG00000057897","ENSMUSG00000058135","ENSMUSG00000058586","ENSMUSG00000058587","ENSMUSG00000058655","ENSMUSG00000058715","ENSMUSG00000058818","ENSMUSG00000059013","ENSMUSG00000059182","ENSMUSG00000059498","ENSMUSG00000059714","ENSMUSG00000059824","ENSMUSG00000059851","ENSMUSG00000059970","ENSMUSG00000060036","ENSMUSG00000060373","ENSMUSG00000060586","ENSMUSG00000060671","ENSMUSG00000060771","ENSMUSG00000060862","ENSMUSG00000060989","ENSMUSG00000061601","

```
ENSMUSG00000061740","ENSMUSG00000061838","ENSMUSG00000061950","ENSMUSG00000
062203","ENSMUSG00000063060","ENSMUSG00000063065","ENSMUSG00000063171","ENS
MUSG00000063275","ENSMUSG00000063415","ENSMUSG00000063576","ENSMUSG00000063
663","ENSMUSG00000064267","ENSMUSG00000064346","ENSMUSG00000066245","ENSMUS
G00000066640","ENSMUSG00000066687","ENSMUSG00000067274","ENSMUSG00000068290
","ENSMUSG00000068742","ENSMUSG00000068854","ENSMUSG00000069516","ENSMUSG00
000069833","ENSMUSG00000070031","ENSMUSG00000070436","ENSMUSG00000070544","
ENSMUSG00000071203","ENSMUSG00000071637","ENSMUSG00000071650","ENSMUSG00000
071714","ENSMUSG00000071715","ENSMUSG00000072949","ENSMUSG00000073409","ENS
MUSG00000073418","ENSMUSG00000073421","ENSMUSG00000073940","ENSMUSG00000074
264","ENSMUSG00000074748","ENSMUSG00000075284","ENSMUSG00000076435","ENSMUS
G00000076617","ENSMUSG00000078566","ENSMUSG00000078771","ENSMUSG00000078853
","ENSMUSG00000078922","ENSMUSG00000078945","ENSMUSG00000079037","ENSMUSG00
000079293","ENSMUSG00000079363","ENSMUSG00000079419","ENSMUSG00000079491","
ENSMUSG00000079499","ENSMUSG00000080316","ENSMUSG00000089715","ENSMUSG00000
090877","ENSMUSG00000092981","ENSMUSG00000095041","ENSMUSG00000096929","ENS
MUSG00000097415","ENSMUSG00000099875","ENSMUSG00000108218","ENSMUSG00000110
588","ENSMUSG00000111027","ENSMUSG00000114277")){
  tmp = attStats(AgeNC645CB[[i]])
  vec<-rep_len(i, length.out=11833)
  tmp$new_col <- vec
  SS<-rep_len(2, length.out=11833)
  tmp$SS <- SS
  attlist[[i]] = tmp
}

AgeNC645SS2CB = dplyr::bind_rows(attlist)
write.csv((AgeNC645SS2CB), "AgeNC645SS2CB", row.names = TRUE)
AgeNC645SS2CB_C = AgeNC645SS2CB[AgeNC645SS2CB$decision =="Confirmed",]
write.csv((AgeNC645SS2CB_C), "AgeNC645SS2CB_C", row.names = TRUE)
AgeNC645SS2CB_noR = AgeNC645SS2CB[AgeNC645SS2CB$decision !="Rejected",]
write.csv((AgeNC645SS2CB_noR), "AgeNC645SS2CB_noR", row.names = TRUE)


AgeNC645CB = list()
for (i in
c("ENSMUSG00000000056","ENSMUSG00000000154","ENSMUSG00000000184","ENSMUSG00
000000290","ENSMUSG00000000682","ENSMUSG00000000823","ENSMUSG00000001119","
ENSMUSG00000001143","ENSMUSG00000001229","ENSMUSG00000001506","ENSMUSG00000
001663","ENSMUSG00000001774","ENSMUSG00000001930","ENSMUSG00000002017","ENS
MUSG00000002289","ENSMUSG00000002763","ENSMUSG00000002900","ENSMUSG00000002
985","ENSMUSG00000002996","ENSMUSG00000003031","ENSMUSG00000003154","ENSMUS
G00000003283","ENSMUSG00000003348","ENSMUSG00000003458","ENSMUSG00000003541
","ENSMUSG00000003617","ENSMUSG00000003731","ENSMUSG00000003814","ENSMUSG00
000003849","ENSMUSG00000003949","ENSMUSG00000004105","ENSMUSG00000004460","
ENSMUSG00000004609","ENSMUSG00000004637","ENSMUSG00000005107","ENSMUSG00000
005397","ENSMUSG00000005417","ENSMUSG00000005447","ENSMUSG00000005686","ENS
MUSG00000005936","ENSMUSG00000006127","ENSMUSG00000006373","ENSMUSG00000006
386","ENSMUSG00000006611","ENSMUSG00000007617","ENSMUSG00000007777","ENSMUS
G00000007891","ENSMUSG00000008540","ENSMUSG00000008845","ENSMUSG00000009406
","ENSMUSG00000010554","ENSMUSG00000010651","ENSMUSG00000011256","ENSMUSG00
000013150","ENSMUSG00000013275","ENSMUSG00000014361","ENSMUSG00000014776","
ENSMUSG00000015189","ENSMUSG00000015340","ENSMUSG00000015468","ENSMUSG00000
015839","ENSMUSG00000015950","ENSMUSG00000016206","ENSMUSG00000016256","ENS
MUSG00000016946","ENSMUSG00000017132","ENSMUSG00000017707","ENSMUSG00000017
754","ENSMUSG00000017774","ENSMUSG00000018042","ENSMUSG00000018160","ENSMUS
G00000018217","ENSMUSG00000018415","ENSMUSG00000018427","ENSMUSG00000018501
","ENSMUSG00000018574","ENSMUSG00000018593","ENSMUSG00000018654","ENSMUSG00
```

000018666","ENSMUSG00000018774","ENSMUSG00000019055","ENSMUSG00000019843","
ENSMUSG00000019846","ENSMUSG00000019872","ENSMUSG00000019916","ENSMUSG00000
019923","ENSMUSG00000020009","ENSMUSG00000020034","ENSMUSG00000020038","ENS
MUSG00000020044","ENSMUSG00000020048","ENSMUSG00000020053","ENSMUSG00000020
111","ENSMUSG00000020120","ENSMUSG00000020123","ENSMUSG00000020143","ENSMUS
G00000020227","ENSMUSG00000020241","ENSMUSG00000020257","ENSMUSG00000020277
","ENSMUSG00000020288","ENSMUSG00000020459","ENSMUSG00000020467","ENSMUSG00
000020472","ENSMUSG00000020571","ENSMUSG00000020594","ENSMUSG00000020653","
ENSMUSG00000020661","ENSMUSG00000020674","ENSMUSG00000020810","ENSMUSG00000
020889","ENSMUSG00000020893","ENSMUSG00000020935","ENSMUSG00000020954","ENS
MUSG00000021003","ENSMUSG00000021025","ENSMUSG00000021037","ENSMUSG00000021
091","ENSMUSG00000021188","ENSMUSG00000021190","ENSMUSG00000021242","ENSMUS
G00000021262","ENSMUSG00000021306","ENSMUSG00000021508","ENSMUSG00000021559
","ENSMUSG00000021775","ENSMUSG00000021814","ENSMUSG00000021903","ENSMUSG00
000021939","ENSMUSG00000021998","ENSMUSG00000022035","ENSMUSG00000022091","
ENSMUSG00000022098","ENSMUSG00000022146","ENSMUSG00000022148","ENSMUSG00000
022186","ENSMUSG00000022197","ENSMUSG00000022270","ENSMUSG00000022372","ENS
MUSG00000022389","ENSMUSG00000022415","ENSMUSG00000022440","ENSMUSG00000022
472","ENSMUSG00000022488","ENSMUSG00000022623","ENSMUSG00000022656","ENSMUS
G00000022665","ENSMUSG00000022684","ENSMUSG00000022797","ENSMUSG00000022816
","ENSMUSG00000022831","ENSMUSG00000022848","ENSMUSG00000022912","ENSMUSG00
000022952","ENSMUSG00000022999","ENSMUSG00000023055","ENSMUSG00000023249","
ENSMUSG00000023328","ENSMUSG00000023367","ENSMUSG00000023952","ENSMUSG00000
023960","ENSMUSG00000024132","ENSMUSG00000024160","ENSMUSG00000024164","ENS
MUSG00000024180","ENSMUSG00000024190","ENSMUSG00000024222","ENSMUSG00000024
299","ENSMUSG00000024300","ENSMUSG00000024421","ENSMUSG00000024451","ENSMUS
G00000024501","ENSMUSG00000024538","ENSMUSG00000024576","ENSMUSG00000024610
","ENSMUSG00000024654","ENSMUSG00000024679","ENSMUSG00000024736","ENSMUSG00
000024810","ENSMUSG00000024885","ENSMUSG00000024900","ENSMUSG00000024965","
ENSMUSG00000024966","ENSMUSG00000024975","ENSMUSG00000025017","ENSMUSG00000
025037","ENSMUSG00000025133","ENSMUSG00000025158","ENSMUSG00000025176","ENS
MUSG00000025262","ENSMUSG00000025265","ENSMUSG00000025270","ENSMUSG00000025
358","ENSMUSG00000025511","ENSMUSG00000025512","ENSMUSG00000025584","ENSMUS
G00000025586","ENSMUSG00000025743","ENSMUSG00000025821","ENSMUSG00000025885
","ENSMUSG00000025888","ENSMUSG00000025980","ENSMUSG00000025981","ENSMUSG00
000025986","ENSMUSG00000026003","ENSMUSG00000026042","ENSMUSG00000026043","
ENSMUSG00000026074","ENSMUSG00000026077","ENSMUSG00000026082","ENSMUSG00000
026234","ENSMUSG00000026288","ENSMUSG00000026303","ENSMUSG00000026395","ENS
MUSG00000026478","ENSMUSG00000026480","ENSMUSG00000026548","ENSMUSG00000026
556","ENSMUSG00000026566","ENSMUSG00000026608","ENSMUSG00000026672","ENSMUS
G00000026728","ENSMUSG00000026739","ENSMUSG00000026786","ENSMUSG00000026837
","ENSMUSG00000026864","ENSMUSG00000026872","ENSMUSG00000026880","ENSMUSG00
000026956","ENSMUSG00000026979","ENSMUSG00000027204","ENSMUSG00000027248","
ENSMUSG00000027274","ENSMUSG00000027330","ENSMUSG00000027412","ENSMUSG00000
027435","ENSMUSG00000027635","ENSMUSG00000027669","ENSMUSG00000027843","ENS
MUSG00000027852","ENSMUSG00000027864","ENSMUSG00000027875","ENSMUSG00000027
933","ENSMUSG00000027947","ENSMUSG00000027962","ENSMUSG00000027995","ENSMUS
G00000028028","ENSMUSG00000028082","ENSMUSG00000028223","ENSMUSG00000028309
","ENSMUSG00000028329","ENSMUSG00000028337","ENSMUSG00000028339","ENSMUSG00
000028343","ENSMUSG00000028410","ENSMUSG00000028581","ENSMUSG00000028597","
ENSMUSG00000028600","ENSMUSG00000028630","ENSMUSG00000028641","ENSMUSG00000
028646","ENSMUSG00000028673","ENSMUSG00000028756","ENSMUSG00000028766","ENS
MUSG00000028795","ENSMUSG00000028820","ENSMUSG00000028826","ENSMUSG00000028
859","ENSMUSG00000028862","ENSMUSG00000028878","ENSMUSG00000028885","ENSMUS
G00000028957","ENSMUSG00000028970","ENSMUSG00000029009","ENSMUSG00000029053
","ENSMUSG00000029090","ENSMUSG00000029119","ENSMUSG00000029177","ENSMUSG00
000029229","ENSMUSG00000029238","ENSMUSG00000029312","ENSMUSG00000029330","
ENSMUSG00000029468","ENSMUSG00000029471","ENSMUSG00000029570","ENSMUSG00000

029616","ENSMUSG00000029657","ENSMUSG00000029661","ENSMUSG00000029675","ENS
MUSG00000029687","ENSMUSG00000029762","ENSMUSG00000029778","ENSMUSG00000029
810","ENSMUSG00000029919","ENSMUSG00000029994","ENSMUSG00000030083","ENSMUS
G00000030087","ENSMUSG00000030096","ENSMUSG00000030103","ENSMUSG00000030172
","ENSMUSG00000030256","ENSMUSG00000030357","ENSMUSG00000030579","ENSMUSG00
000030678","ENSMUSG00000030681","ENSMUSG00000030701","ENSMUSG00000030711","
ENSMUSG00000030761","ENSMUSG00000030786","ENSMUSG00000030798","ENSMUSG00000
030921","ENSMUSG00000031004","ENSMUSG00000031016","ENSMUSG00000031024","ENS
MUSG00000031144","ENSMUSG00000031167","ENSMUSG00000031217","ENSMUSG00000031
221","ENSMUSG00000031232","ENSMUSG00000031239","ENSMUSG00000031274","ENSMUS
G00000031298","ENSMUSG00000031343","ENSMUSG00000031379","ENSMUSG00000031431
","ENSMUSG00000031442","ENSMUSG00000031467","ENSMUSG00000031479","ENSMUSG00
000031502","ENSMUSG00000031503","ENSMUSG00000031511","ENSMUSG00000031596","
ENSMUSG00000031740","ENSMUSG00000031762","ENSMUSG00000031765","ENSMUSG00000
031770","ENSMUSG00000031778","ENSMUSG00000031790","ENSMUSG00000031864","ENS
MUSG00000032000","ENSMUSG00000032010","ENSMUSG00000032086","ENSMUSG00000032
115","ENSMUSG00000032125","ENSMUSG00000032127","ENSMUSG00000032228","ENSMUS
G00000032348","ENSMUSG00000032349","ENSMUSG00000032363","ENSMUSG00000032369
","ENSMUSG00000032372","ENSMUSG00000032399","ENSMUSG00000032802","ENSMUSG00
000033032","ENSMUSG00000033082","ENSMUSG00000033159","ENSMUSG00000033191","
ENSMUSG00000033220","ENSMUSG00000033249","ENSMUSG00000033308","ENSMUSG00000
033318","ENSMUSG00000033460","ENSMUSG00000033499","ENSMUSG00000033581","ENS
MUSG00000033863","ENSMUSG00000033933","ENSMUSG00000034006","ENSMUSG00000034
007","ENSMUSG00000034116","ENSMUSG00000034154","ENSMUSG00000034157","ENSMUS
G00000034205","ENSMUSG00000034252","ENSMUSG00000034255","ENSMUSG00000034285
","ENSMUSG00000034341","ENSMUSG00000034349","ENSMUSG00000034403","ENSMUSG00
000034614","ENSMUSG00000034647","ENSMUSG00000034656","ENSMUSG00000034674","
ENSMUSG00000034707","ENSMUSG00000034795","ENSMUSG00000034832","ENSMUSG00000
034936","ENSMUSG00000034947","ENSMUSG00000034993","ENSMUSG00000034994","ENS
MUSG00000035004","ENSMUSG00000035064","ENSMUSG00000035206","ENSMUSG00000035
212","ENSMUSG00000035245","ENSMUSG00000035273","ENSMUSG00000035504","ENSMUS
G00000035513","ENSMUSG00000035772","ENSMUSG00000035824","ENSMUSG00000035828
","ENSMUSG00000035834","ENSMUSG00000035877","ENSMUSG00000035936","ENSMUSG00
000036040","ENSMUSG00000036273","ENSMUSG00000036275","ENSMUSG00000036402","
ENSMUSG00000036427","ENSMUSG00000036545","ENSMUSG00000036594","ENSMUSG00000
036615","ENSMUSG00000036667","ENSMUSG00000036862","ENSMUSG00000036887","ENS
MUSG00000036893","ENSMUSG00000036896","ENSMUSG00000036905","ENSMUSG00000036
934","ENSMUSG00000036944","ENSMUSG00000037003","ENSMUSG00000037058","ENSMUS
G00000037235","ENSMUSG00000037347","ENSMUSG00000037405","ENSMUSG00000037411
","ENSMUSG00000037455","ENSMUSG00000037461","ENSMUSG00000037475","ENSMUSG00
000037613","ENSMUSG00000037712","ENSMUSG00000037824","ENSMUSG00000037860","
ENSMUSG00000037892","ENSMUSG00000038007","ENSMUSG00000038143","ENSMUSG00000
038147","ENSMUSG00000038175","ENSMUSG00000038235","ENSMUSG00000038305","ENS
MUSG00000038393","ENSMUSG00000038521","ENSMUSG00000038642","ENSMUSG00000038
695","ENSMUSG00000038776","ENSMUSG00000039096","ENSMUSG00000039126","ENSMUS
G00000039159","ENSMUSG00000039176","ENSMUSG00000039254","ENSMUSG00000039328
","ENSMUSG00000039382","ENSMUSG00000039745","ENSMUSG00000039982","ENSMUSG00
000040093","ENSMUSG00000040097","ENSMUSG00000040170","ENSMUSG00000040174","
ENSMUSG00000040552","ENSMUSG00000040612","ENSMUSG00000040631","ENSMUSG00000
040713","ENSMUSG00000040723","ENSMUSG00000040751","ENSMUSG00000040820","ENS
MUSG00000041040","ENSMUSG00000041084","ENSMUSG00000041117","ENSMUSG00000041
355","ENSMUSG00000041417","ENSMUSG00000041515","ENSMUSG00000041560","ENSMUS
G00000041779","ENSMUSG00000041801","ENSMUSG00000041828","ENSMUSG00000041849
","ENSMUSG00000041926","ENSMUSG00000041936","ENSMUSG00000041992","ENSMUSG00
000042035","ENSMUSG00000042099","ENSMUSG00000042129","ENSMUSG00000042155","
ENSMUSG00000042251","ENSMUSG00000042286","ENSMUSG00000042426","ENSMUSG00000
042436","ENSMUSG00000042487","ENSMUSG00000042594","ENSMUSG00000042644","ENS
MUSG00000042677","ENSMUSG00000042688","ENSMUSG00000042712","ENSMUSG00000042

```
834","ENSMUSG00000043631","ENSMUSG00000044349","ENSMUSG00000044562","ENSMUS
G00000044583","ENSMUSG00000044949","ENSMUSG00000045193","ENSMUSG00000046230
","ENSMUSG00000046312","ENSMUSG00000046318","ENSMUSG00000046532","ENSMUSG00
000046688","ENSMUSG00000046743","ENSMUSG00000046805","ENSMUSG00000046994","
ENSMUSG00000047045","ENSMUSG00000047238","ENSMUSG00000047866","ENSMUSG00000
047945","ENSMUSG00000048126","ENSMUSG00000048249","ENSMUSG00000048330","ENS
MUSG00000048489","ENSMUSG00000048756","ENSMUSG00000049047","ENSMUSG00000049
076","ENSMUSG00000049804","ENSMUSG00000049878","ENSMUSG00000050103","ENSMUS
G00000050335","ENSMUSG00000050708","ENSMUSG00000050714","ENSMUSG00000051483
","ENSMUSG00000051506","ENSMUSG00000051579","ENSMUSG00000051864","ENSMUSG00
000052040","ENSMUSG00000052139","ENSMUSG00000052305","ENSMUSG00000052593","
ENSMUSG00000052760","ENSMUSG00000052812","ENSMUSG00000052915","ENSMUSG00000
053063","ENSMUSG00000053279","ENSMUSG00000053898","ENSMUSG00000054072","ENS
MUSG00000054404","ENSMUSG00000054509","ENSMUSG00000054612","ENSMUSG00000054
733","ENSMUSG00000054863","ENSMUSG00000055067","ENSMUSG00000055116","ENSMUS
G00000055541","ENSMUSG00000055866","ENSMUSG00000055980","ENSMUSG00000056228
","ENSMUSG00000056515","ENSMUSG00000056666","ENSMUSG00000056737","ENSMUSG00
000056749","ENSMUSG00000057137","ENSMUSG00000057193","ENSMUSG00000057406","
ENSMUSG00000057497","ENSMUSG00000057531","ENSMUSG00000057894","ENSMUSG00000
057897","ENSMUSG00000058135","ENSMUSG00000058586","ENSMUSG00000058587","ENS
MUSG00000058655","ENSMUSG00000058715","ENSMUSG00000058818","ENSMUSG00000059
013","ENSMUSG00000059182","ENSMUSG00000059498","ENSMUSG00000059714","ENSMUS
G00000059824","ENSMUSG00000059851","ENSMUSG00000059970","ENSMUSG00000060036
","ENSMUSG00000060373","ENSMUSG00000060586","ENSMUSG00000060671","ENSMUSG00
000060771","ENSMUSG00000060862","ENSMUSG00000060989","ENSMUSG00000061601","
ENSMUSG00000061740","ENSMUSG00000061838","ENSMUSG00000061950","ENSMUSG00000
062203","ENSMUSG00000063060","ENSMUSG00000063065","ENSMUSG00000063171","ENS
MUSG00000063275","ENSMUSG00000063415","ENSMUSG00000063576","ENSMUSG00000063
663","ENSMUSG00000064267","ENSMUSG00000064346","ENSMUSG00000066245","ENSMUS
G00000066640","ENSMUSG00000066687","ENSMUSG00000067274","ENSMUSG00000068290
","ENSMUSG00000068742","ENSMUSG00000068854","ENSMUSG00000069516","ENSMUSG00
000069833","ENSMUSG00000070031","ENSMUSG00000070436","ENSMUSG00000070544","
ENSMUSG00000071203","ENSMUSG00000071637","ENSMUSG00000071650","ENSMUSG00000
071714","ENSMUSG00000071715","ENSMUSG00000072949","ENSMUSG00000073409","ENS
MUSG00000073418","ENSMUSG00000073421","ENSMUSG00000073940","ENSMUSG00000074
264","ENSMUSG00000074748","ENSMUSG00000075284","ENSMUSG00000076435","ENSMUS
G00000076617","ENSMUSG00000078566","ENSMUSG00000078771","ENSMUSG00000078853
","ENSMUSG00000078922","ENSMUSG00000078945","ENSMUSG00000079037","ENSMUSG00
000079293","ENSMUSG00000079363","ENSMUSG00000079419","ENSMUSG00000079491","
ENSMUSG00000079499","ENSMUSG00000080316","ENSMUSG00000089715","ENSMUSG00000
090877","ENSMUSG00000092981","ENSMUSG00000095041","ENSMUSG00000096929","ENS
MUSG00000097415","ENSMUSG00000099875","ENSMUSG00000108218","ENSMUSG00000110
588","ENSMUSG00000111027","ENSMUSG00000114277")){
  print(i)
  set.seed(3)
  data = N
  y=data.frame(data[,i])
  y=y[,1]
  x=data.frame(data[,colnames(data)!= i])
  output <- Boruta(y = y, x=x, doTrace = 2)
  AgeNC645CB[[i]] = output
}
Sys.time()

attlist = list()
for (i in
c("ENSMUSG00000000056","ENSMUSG00000000154","ENSMUSG00000000184","ENSMUSG00
000000290","ENSMUSG00000000682","ENSMUSG00000000823","ENSMUSG00000001119","
```

ENSMUSG00000001143","ENSMUSG00000001229","ENSMUSG00000001506","ENSMUSG00000
001663","ENSMUSG00000001774","ENSMUSG00000001930","ENSMUSG00000002017","ENS
MUSG00000002289","ENSMUSG00000002763","ENSMUSG00000002900","ENSMUSG00000002
985","ENSMUSG00000002996","ENSMUSG00000003031","ENSMUSG00000003154","ENSMUS
G00000003283","ENSMUSG00000003348","ENSMUSG00000003458","ENSMUSG00000003541
","ENSMUSG00000003617","ENSMUSG00000003731","ENSMUSG00000003814","ENSMUSG00
000003849","ENSMUSG00000003949","ENSMUSG00000004105","ENSMUSG00000004460","
ENSMUSG00000004609","ENSMUSG00000004637","ENSMUSG00000005107","ENSMUSG00000
005397","ENSMUSG00000005417","ENSMUSG00000005447","ENSMUSG00000005686","ENS
MUSG00000005936","ENSMUSG00000006127","ENSMUSG00000006373","ENSMUSG00000006
386","ENSMUSG00000006611","ENSMUSG00000007617","ENSMUSG00000007777","ENSMUS
G00000007891","ENSMUSG00000008540","ENSMUSG00000008845","ENSMUSG00000009406
","ENSMUSG00000010554","ENSMUSG00000010651","ENSMUSG00000011256","ENSMUSG00
000013150","ENSMUSG00000013275","ENSMUSG00000014361","ENSMUSG00000014776","
ENSMUSG00000015189","ENSMUSG00000015340","ENSMUSG00000015468","ENSMUSG00000
015839","ENSMUSG00000015950","ENSMUSG00000016206","ENSMUSG00000016256","ENS
MUSG00000016946","ENSMUSG00000017132","ENSMUSG00000017707","ENSMUSG00000017
754","ENSMUSG00000017774","ENSMUSG00000018042","ENSMUSG00000018160","ENSMUS
G00000018217","ENSMUSG00000018415","ENSMUSG00000018427","ENSMUSG00000018501
","ENSMUSG00000018574","ENSMUSG00000018593","ENSMUSG00000018654","ENSMUSG00
000018666","ENSMUSG00000018774","ENSMUSG00000019055","ENSMUSG00000019843","
ENSMUSG00000019846","ENSMUSG00000019872","ENSMUSG00000019916","ENSMUSG00000
019923","ENSMUSG00000020009","ENSMUSG00000020034","ENSMUSG00000020038","ENS
MUSG00000020044","ENSMUSG00000020048","ENSMUSG00000020053","ENSMUSG00000020
111","ENSMUSG00000020120","ENSMUSG00000020123","ENSMUSG00000020143","ENSMUS
G00000020227","ENSMUSG00000020241","ENSMUSG00000020257","ENSMUSG00000020277
","ENSMUSG00000020288","ENSMUSG00000020459","ENSMUSG00000020467","ENSMUSG00
000020472","ENSMUSG00000020571","ENSMUSG00000020594","ENSMUSG00000020653","
ENSMUSG00000020661","ENSMUSG00000020674","ENSMUSG00000020810","ENSMUSG00000
020889","ENSMUSG00000020893","ENSMUSG00000020935","ENSMUSG00000020954","ENS
MUSG00000021003","ENSMUSG00000021025","ENSMUSG00000021037","ENSMUSG00000021
091","ENSMUSG00000021188","ENSMUSG00000021190","ENSMUSG00000021242","ENSMUS
G00000021262","ENSMUSG00000021306","ENSMUSG00000021508","ENSMUSG00000021559
","ENSMUSG00000021775","ENSMUSG00000021814","ENSMUSG00000021903","ENSMUSG00
000021939","ENSMUSG00000021998","ENSMUSG00000022035","ENSMUSG00000022091","
ENSMUSG00000022098","ENSMUSG00000022146","ENSMUSG00000022148","ENSMUSG00000
022186","ENSMUSG00000022197","ENSMUSG00000022270","ENSMUSG00000022372","ENS
MUSG00000022389","ENSMUSG00000022415","ENSMUSG00000022440","ENSMUSG00000022
472","ENSMUSG00000022488","ENSMUSG00000022623","ENSMUSG00000022656","ENSMUS
G00000022665","ENSMUSG00000022684","ENSMUSG00000022797","ENSMUSG00000022816
","ENSMUSG00000022831","ENSMUSG00000022848","ENSMUSG00000022912","ENSMUSG00
000022952","ENSMUSG00000022999","ENSMUSG00000023055","ENSMUSG00000023249","
ENSMUSG00000023328","ENSMUSG00000023367","ENSMUSG00000023952","ENSMUSG00000
023960","ENSMUSG00000024132","ENSMUSG00000024160","ENSMUSG00000024164","ENS
MUSG00000024180","ENSMUSG00000024190","ENSMUSG00000024222","ENSMUSG00000024
299","ENSMUSG00000024300","ENSMUSG00000024421","ENSMUSG00000024451","ENSMUS
G00000024501","ENSMUSG00000024538","ENSMUSG00000024576","ENSMUSG00000024610
","ENSMUSG00000024654","ENSMUSG00000024679","ENSMUSG00000024736","ENSMUSG00
000024810","ENSMUSG00000024885","ENSMUSG00000024900","ENSMUSG00000024965","
ENSMUSG00000024966","ENSMUSG00000024975","ENSMUSG00000025017","ENSMUSG00000
025037","ENSMUSG00000025133","ENSMUSG00000025158","ENSMUSG00000025176","ENS
MUSG00000025262","ENSMUSG00000025265","ENSMUSG00000025270","ENSMUSG00000025
358","ENSMUSG00000025511","ENSMUSG00000025512","ENSMUSG00000025584","ENSMUS
G00000025586","ENSMUSG00000025743","ENSMUSG00000025821","ENSMUSG00000025885
","ENSMUSG00000025888","ENSMUSG00000025980","ENSMUSG00000025981","ENSMUSG00
000025986","ENSMUSG00000026003","ENSMUSG00000026042","ENSMUSG00000026043","
ENSMUSG00000026074","ENSMUSG00000026077","ENSMUSG00000026082","ENSMUSG00000
026234","ENSMUSG00000026288","ENSMUSG00000026303","ENSMUSG00000026395","ENS

MUSG00000026478","ENSMUSG00000026480","ENSMUSG00000026548","ENSMUSG00000026
556","ENSMUSG00000026566","ENSMUSG00000026608","ENSMUSG00000026672","ENSMUS
G00000026728","ENSMUSG00000026739","ENSMUSG00000026786","ENSMUSG00000026837
","ENSMUSG00000026864","ENSMUSG00000026872","ENSMUSG00000026880","ENSMUSG00
000026956","ENSMUSG00000026979","ENSMUSG00000027204","ENSMUSG00000027248","
ENSMUSG00000027274","ENSMUSG00000027330","ENSMUSG00000027412","ENSMUSG00000
027435","ENSMUSG00000027635","ENSMUSG00000027669","ENSMUSG00000027843","ENS
MUSG00000027852","ENSMUSG00000027864","ENSMUSG00000027875","ENSMUSG00000027
933","ENSMUSG00000027947","ENSMUSG00000027962","ENSMUSG00000027995","ENSMUS
G00000028028","ENSMUSG00000028082","ENSMUSG00000028223","ENSMUSG00000028309
","ENSMUSG00000028329","ENSMUSG00000028337","ENSMUSG00000028339","ENSMUSG00
000028343","ENSMUSG00000028410","ENSMUSG00000028581","ENSMUSG00000028597","
ENSMUSG00000028600","ENSMUSG00000028630","ENSMUSG00000028641","ENSMUSG00000
028646","ENSMUSG00000028673","ENSMUSG00000028756","ENSMUSG00000028766","ENS
MUSG00000028795","ENSMUSG00000028820","ENSMUSG00000028826","ENSMUSG00000028
859","ENSMUSG00000028862","ENSMUSG00000028878","ENSMUSG00000028885","ENSMUS
G00000028957","ENSMUSG00000028970","ENSMUSG00000029009","ENSMUSG00000029053
","ENSMUSG00000029090","ENSMUSG00000029119","ENSMUSG00000029177","ENSMUSG00
000029229","ENSMUSG00000029238","ENSMUSG00000029312","ENSMUSG00000029330","
ENSMUSG00000029468","ENSMUSG00000029471","ENSMUSG00000029570","ENSMUSG00000
029616","ENSMUSG00000029657","ENSMUSG00000029661","ENSMUSG00000029675","ENS
MUSG00000029687","ENSMUSG00000029762","ENSMUSG00000029778","ENSMUSG00000029
810","ENSMUSG00000029919","ENSMUSG00000029994","ENSMUSG00000030083","ENSMUS
G00000030087","ENSMUSG00000030096","ENSMUSG00000030103","ENSMUSG00000030172
","ENSMUSG00000030256","ENSMUSG00000030357","ENSMUSG00000030579","ENSMUSG00
000030678","ENSMUSG00000030681","ENSMUSG00000030701","ENSMUSG00000030711","
ENSMUSG00000030761","ENSMUSG00000030786","ENSMUSG00000030798","ENSMUSG00000
030921","ENSMUSG00000031004","ENSMUSG00000031016","ENSMUSG00000031024","ENS
MUSG00000031144","ENSMUSG00000031167","ENSMUSG00000031217","ENSMUSG00000031
221","ENSMUSG00000031232","ENSMUSG00000031239","ENSMUSG00000031274","ENSMUS
G00000031298","ENSMUSG00000031343","ENSMUSG00000031379","ENSMUSG00000031431
","ENSMUSG00000031442","ENSMUSG00000031467","ENSMUSG00000031479","ENSMUSG00
000031502","ENSMUSG00000031503","ENSMUSG00000031511","ENSMUSG00000031596","
ENSMUSG00000031740","ENSMUSG00000031762","ENSMUSG00000031765","ENSMUSG00000
031770","ENSMUSG00000031778","ENSMUSG00000031790","ENSMUSG00000031864","ENS
MUSG00000032000","ENSMUSG00000032010","ENSMUSG00000032086","ENSMUSG00000032
115","ENSMUSG00000032125","ENSMUSG00000032127","ENSMUSG00000032228","ENSMUS
G00000032348","ENSMUSG00000032349","ENSMUSG00000032363","ENSMUSG00000032369
","ENSMUSG00000032372","ENSMUSG00000032399","ENSMUSG00000032802","ENSMUSG00
000033032","ENSMUSG00000033082","ENSMUSG00000033159","ENSMUSG00000033191","
ENSMUSG00000033220","ENSMUSG00000033249","ENSMUSG00000033308","ENSMUSG00000
033318","ENSMUSG00000033460","ENSMUSG00000033499","ENSMUSG00000033581","ENS
MUSG00000033863","ENSMUSG00000033933","ENSMUSG00000034006","ENSMUSG00000034
007","ENSMUSG00000034116","ENSMUSG00000034154","ENSMUSG00000034157","ENSMUS
G00000034205","ENSMUSG00000034252","ENSMUSG00000034255","ENSMUSG00000034285
","ENSMUSG00000034341","ENSMUSG00000034349","ENSMUSG00000034403","ENSMUSG00
000034614","ENSMUSG00000034647","ENSMUSG00000034656","ENSMUSG00000034674","
ENSMUSG00000034707","ENSMUSG00000034795","ENSMUSG00000034832","ENSMUSG00000
034936","ENSMUSG00000034947","ENSMUSG00000034993","ENSMUSG00000034994","ENS
MUSG00000035004","ENSMUSG00000035064","ENSMUSG00000035206","ENSMUSG00000035
212","ENSMUSG00000035245","ENSMUSG00000035273","ENSMUSG00000035504","ENSMUS
G00000035513","ENSMUSG00000035772","ENSMUSG00000035824","ENSMUSG00000035828
","ENSMUSG00000035834","ENSMUSG00000035877","ENSMUSG00000035936","ENSMUSG00
000036040","ENSMUSG00000036273","ENSMUSG00000036275","ENSMUSG00000036402","
ENSMUSG00000036427","ENSMUSG00000036545","ENSMUSG00000036594","ENSMUSG00000
036615","ENSMUSG00000036667","ENSMUSG00000036862","ENSMUSG00000036887","ENS
MUSG00000036893","ENSMUSG00000036896","ENSMUSG00000036905","ENSMUSG00000036
934","ENSMUSG00000036944","ENSMUSG00000037003","ENSMUSG00000037058","ENSMUS

G00000037235","ENSMUSG00000037347","ENSMUSG00000037405","ENSMUSG00000037411
","ENSMUSG00000037455","ENSMUSG00000037461","ENSMUSG00000037475","ENSMUSG00
000037613","ENSMUSG00000037712","ENSMUSG00000037824","ENSMUSG00000037860","
ENSMUSG00000037892","ENSMUSG00000038007","ENSMUSG00000038143","ENSMUSG00000
038147","ENSMUSG00000038175","ENSMUSG00000038235","ENSMUSG00000038305","ENS
MUSG00000038393","ENSMUSG00000038521","ENSMUSG00000038642","ENSMUSG00000038
695","ENSMUSG00000038776","ENSMUSG00000039096","ENSMUSG00000039126","ENSMUS
G00000039159","ENSMUSG00000039176","ENSMUSG00000039254","ENSMUSG00000039328
","ENSMUSG00000039382","ENSMUSG00000039745","ENSMUSG00000039982","ENSMUSG00
000040093","ENSMUSG00000040097","ENSMUSG00000040170","ENSMUSG00000040174","
ENSMUSG00000040552","ENSMUSG00000040612","ENSMUSG00000040631","ENSMUSG00000
040713","ENSMUSG00000040723","ENSMUSG00000040751","ENSMUSG00000040820","ENS
MUSG00000041040","ENSMUSG00000041084","ENSMUSG00000041117","ENSMUSG00000041
355","ENSMUSG00000041417","ENSMUSG00000041515","ENSMUSG00000041560","ENSMUS
G00000041779","ENSMUSG00000041801","ENSMUSG00000041828","ENSMUSG00000041849
","ENSMUSG00000041926","ENSMUSG00000041936","ENSMUSG00000041992","ENSMUSG00
000042035","ENSMUSG00000042099","ENSMUSG00000042129","ENSMUSG00000042155","
ENSMUSG00000042251","ENSMUSG00000042286","ENSMUSG00000042426","ENSMUSG00000
042436","ENSMUSG00000042487","ENSMUSG00000042594","ENSMUSG00000042644","ENS
MUSG00000042677","ENSMUSG00000042688","ENSMUSG00000042712","ENSMUSG00000042
834","ENSMUSG00000043631","ENSMUSG00000044349","ENSMUSG00000044562","ENSMUS
G00000044583","ENSMUSG00000044949","ENSMUSG00000045193","ENSMUSG00000046230
","ENSMUSG00000046312","ENSMUSG00000046318","ENSMUSG00000046532","ENSMUSG00
000046688","ENSMUSG00000046743","ENSMUSG00000046805","ENSMUSG00000046994","
ENSMUSG00000047045","ENSMUSG00000047238","ENSMUSG00000047866","ENSMUSG00000
047945","ENSMUSG00000048126","ENSMUSG00000048249","ENSMUSG00000048330","ENS
MUSG00000048489","ENSMUSG00000048756","ENSMUSG00000049047","ENSMUSG00000049
076","ENSMUSG00000049804","ENSMUSG00000049878","ENSMUSG00000050103","ENSMUS
G00000050335","ENSMUSG00000050708","ENSMUSG00000050714","ENSMUSG00000051483
","ENSMUSG00000051506","ENSMUSG00000051579","ENSMUSG00000051864","ENSMUSG00
000052040","ENSMUSG00000052139","ENSMUSG00000052305","ENSMUSG00000052593","
ENSMUSG00000052760","ENSMUSG00000052812","ENSMUSG00000052915","ENSMUSG00000
053063","ENSMUSG00000053279","ENSMUSG00000053898","ENSMUSG00000054072","ENS
MUSG00000054404","ENSMUSG00000054509","ENSMUSG00000054612","ENSMUSG00000054
733","ENSMUSG00000054863","ENSMUSG00000055067","ENSMUSG00000055116","ENSMUS
G00000055541","ENSMUSG00000055866","ENSMUSG00000055980","ENSMUSG00000056228
","ENSMUSG00000056515","ENSMUSG00000056666","ENSMUSG00000056737","ENSMUSG00
000056749","ENSMUSG00000057137","ENSMUSG00000057193","ENSMUSG00000057406","
ENSMUSG00000057497","ENSMUSG00000057531","ENSMUSG00000057894","ENSMUSG00000
057897","ENSMUSG00000058135","ENSMUSG00000058586","ENSMUSG00000058587","ENS
MUSG00000058655","ENSMUSG00000058715","ENSMUSG00000058818","ENSMUSG00000059
013","ENSMUSG00000059182","ENSMUSG00000059498","ENSMUSG00000059714","ENSMUS
G00000059824","ENSMUSG00000059851","ENSMUSG00000059970","ENSMUSG00000060036
","ENSMUSG00000060373","ENSMUSG00000060586","ENSMUSG00000060671","ENSMUSG00
000060771","ENSMUSG00000060862","ENSMUSG00000060989","ENSMUSG00000061601","
ENSMUSG00000061740","ENSMUSG00000061838","ENSMUSG00000061950","ENSMUSG00000
062203","ENSMUSG00000063060","ENSMUSG00000063065","ENSMUSG00000063171","ENS
MUSG00000063275","ENSMUSG00000063415","ENSMUSG00000063576","ENSMUSG00000063
663","ENSMUSG00000064267","ENSMUSG00000064346","ENSMUSG00000066245","ENSMUS
G00000066640","ENSMUSG00000066687","ENSMUSG00000067274","ENSMUSG00000068290
","ENSMUSG00000068742","ENSMUSG00000068854","ENSMUSG00000069516","ENSMUSG00
000069833","ENSMUSG00000070031","ENSMUSG00000070436","ENSMUSG00000070544","
ENSMUSG00000071203","ENSMUSG00000071637","ENSMUSG00000071650","ENSMUSG00000
071714","ENSMUSG00000071715","ENSMUSG00000072949","ENSMUSG00000073409","ENS
MUSG00000073418","ENSMUSG00000073421","ENSMUSG00000073940","ENSMUSG00000074
264","ENSMUSG00000074748","ENSMUSG00000075284","ENSMUSG00000076435","ENSMUS
G00000076617","ENSMUSG00000078566","ENSMUSG00000078771","ENSMUSG00000078853
","ENSMUSG00000078922","ENSMUSG00000078945","ENSMUSG00000079037","ENSMUSG00

```
000079293","ENSMUSG00000079363","ENSMUSG00000079419","ENSMUSG00000079491","
ENSMUSG00000079499","ENSMUSG00000080316","ENSMUSG00000089715","ENSMUSG00000
090877","ENSMUSG00000092981","ENSMUSG00000095041","ENSMUSG00000096929","ENS
MUSG00000097415","ENSMUSG00000099875","ENSMUSG00000108218","ENSMUSG00000110
588","ENSMUSG00000111027","ENSMUSG00000114277")){
  tmp = attStats(AgeNC645CB[[i]])
  vec<-rep_len(i, length.out=11833)
  tmp$new_col <- vec
  SS<-rep_len(3, length.out=11833)
  tmp$SS <- SS
  attlist[[i]] = tmp
}

AgeNC645SS3CB = dplyr::bind_rows(attlist)
write.csv((AgeNC645SS3CB), "AgeNC645SS3CB", row.names = TRUE)
AgeNC645SS3CB_C = AgeNC645SS3CB[AgeNC645SS3CB$decision =="Confirmed",]
write.csv((AgeNC645SS3CB_C), "AgeNC645SS3CB_C", row.names = TRUE)
AgeNC645SS3CB_noR = AgeNC645SS3CB[AgeNC645SS3CB$decision !="Rejected",]
write.csv((AgeNC645SS3CB_noR), "AgeNC645SS3CB_noR", row.names = TRUE)
```

Boruta all genes:

```
Sys.time()
library(readxl)
A <-read_excel("/home/aqcza/Tab. S2.xlsx")
A$Sex <-as.factor(A$Sex)
A$Strain <-as.factor(A$Strain)
A$Organ <-as.factor(A$Organ)

library(Boruta)
Sys.time()
BigCB11830SS1_5to300=list()
for (i in colnames(A[,5:300])){
  print(i)
  set.seed(1)
  data=A
  y=data.frame(data[,i])
  y=y[,1]
  x=data.frame(data[,colnames(data)!= i])
  output=Boruta(y = y, x=x, doTrace = 2)
  BigCB11830SS1_5to300[[i]]=output
}
Sys.time()

attlist_5to300=list()
for (i in colnames(A[,5:300])){
  tmp=attStats(BigCB11830SS1_5to300[[i]])
  vec=rep_len(i, length.out=11833)
  tmp$new_col=vec
  SS=rep_len(1, length.out=11833)
  tmp$SS=SS
  attlist_5to300[[i]]=tmp
}
Sys.time()

library(dplyr)
BigCB11830SS1_5to300_stacked=dplyr::bind_rows(attlist_5to300)
write.csv((BigCB11830SS1_5to300_stacked),
"/home/aqcza/save/test/Atestsmall1_5to300_stacked", row.names = TRUE)

BigCB11830SS1_5to300_stacked_C=BigCB11830SS1_5to300_stacked[BigCB11830SS1_5
to300_stacked$decision=="Confirmed",]
write.csv((BigCB11830SS1_5to300_stacked_C),
"/home/aqcza/save/test/Atestsmall1_5to300_stacked_C", row.names = TRUE)

BigCB11830SS1_5to300_stacked_noR=BigCB11830SS1_5to300_stacked[BigCB11830SS1
_5to300_stacked$decision!="Rejected",]
write.csv((BigCB11830SS1_5to300_stacked_noR),
"/home/aqcza/save/test/Atestsmall1_5to300_stacked_noR", row.names = TRUE)
```

Compute for all modules:

Start	Stop
5	300
301	600
601	900
901	1200
1201	1500
1501	1800
1801	2100
2101	2400
2401	2700
2701	3000
3001	3300
3301	3600
3601	3900
3901	4200
4201	4500
4501	4800
4801	5100
5101	5400
5401	5700
5701	6000
6001	6300
6301	6600
6601	6900
6901	7200
7201	7500
7501	7800
7801	8100
8101	8400
8401	8700
8701	9000
9001	9300
9301	9600
9601	9900
9901	10200
10201	10500
10501	10800
10801	11100
11101	11400
11401	11700
11701	11834

Compute for all seeds: set.seed(1), set.seed(2), set.seed(3)

Modules * seeds: 120 data packages
