# Supplementary material for: The circadian rhythm: A key variable in aging?
Source: Aging Cell. 2024 Jul 30;23(11):e14268. doi: 10.1111/acel.14268 (PMC11561671; doi:10.1111/acel.14268)
Supplement: Supplementary file 25 — Data S1–S6. [file ACEL-23-e14268-s017.zip › Data S2-S6/Data S5 new.pdf]

**For the Aging Network:**

```
library(data.table)
SS1C <- fread("confirmed features of 645 target variables, seed1")
SS2C <- fread("confirmed features of 645 target variables, seed2")
SS3C <- fread("confirmed features of 645 target variables, seed3")

SSall_C<-rbind(SS1C, SS2C, SS3C)

library(tidyr)
SSall_C <- SSall_C %>% separate(V1, c('Feature', 'Number'))

SSall_C$Concatenated <- paste(SSall_C$Feature, SSall_C$TargetVariable)

SSall_C_meanImpMean <-
aggregate(x= SSall_C$meanImp,
          by= list(SSall_C$Concatenated),
          FUN=mean)
SSall_C_normHitsMean <-
aggregate(x= SSall_C$normHits,
          by= list(SSall_C$Concatenated),
          FUN=mean)

DF<- SSall_C_meanImpMean$x * SSall_C_normHitsMean$x

SSall_C_DF <- cbind(SSall_C_meanImpMean, SSall_C_normHitsMean$x, DF)

SSall_C_DF <- SSall_C_DF %>% separate(Group.1, c('Feature', 'TV'))
```

For the Correlation Ratio Estimation:

```
Aging645=read_excel("645 target variables")

SSall_C_645<-merge(SSall_C_DF, Aging645, by="Feature")

SSall_C_645_SumFeature <-
aggregate(x= SSall_C_645$DF,
          by= list(SSall_C_645$Feature),
          FUN=sum)
SSall_C_645_SumTV <-
aggregate(SSall_C_645$DF,
          by= list(SSall_C_645$TV),
          FUN=sum)

final <-merge(SSall_C_645_SumFeature, SSall_C_645_SumTV, by="Group.1", all
= TRUE)

final_YdifX <- final$x.y / final$x.x

final_YdifX_complete <- cbind(final, final_YdifX)
```

**For the overall Target Variable Linkage Estimation:**

```
SSall_C_DF = SSall_C_DF[SSall_C_DF$Feature !="Age",]
SSall_C_DF = SSall_C_DF[SSall_C_DF$Feature !="Sex",]
SSall_C_DF = SSall_C_DF[SSall_C_DF$Feature !="Strain",]
SSall_C_DF = SSall_C_DF[SSall_C_DF$Feature !="Organ",]

SSall_C_DF_SumTV <-
aggregate(SSall_C_DF$DF,
          by= list(SSall_C_DF$TV),
          FUN=sum)
```
