# Supplementary material for: The circadian rhythm: A key variable in aging?
Source: Aging Cell. 2024 Jul 30;23(11):e14268. doi: 10.1111/acel.14268 (PMC11561671; doi:10.1111/acel.14268)
Supplement: Supplementary file 25 — Data S1–S6. [file ACEL-23-e14268-s017.zip › Data S2-S6/Data S6.pdf]

For the Accuracy Prediction:

```
install.packages("readxl")
library(readxl)
data_New1 <-read_excel("Table S2.xlsx")
```

numerical

```
set.seed(1)
ind50 <- sample(2, nrow(data_New1), replace = T, prob = c(0.5, 0.5))
train50 <- data_New1[ind50==1,]
test50 <- data_New1[ind50==2,]

library(mlbench)
library(Boruta)
library(caret)
library(randomForest)

set.seed(1)
rf50 <- randomForest(Age~., data = train50)
p50 <- predict(rf50, test50)
pr50=round(p50)
confusionMatrix(as.factor(pr50), as.factor(test50$Age))
```

```
confusionMatrix(as.factor(pr50), as.factor(test50$Age))
Confusion Matrix and Statistics

          Reference
Prediction  1  2  3  4  5
         1 32  0  0  0  0
         2 39 50  7  0  0
         3  1 15 51 28  5
         4  0  0  5 42 57
         5  0  0  0  3 16

Overall Statistics

               Accuracy : 0.5442
                 95% CI : (0.4904, 0.5971)
    No Information Rate : 0.2222
    P-Value [Acc > NIR] : < 2.2e-16

                  Kappa : 0.4343

 Mcnemar's Test P-Value : NA

Statistics by Class:

                     Class: 1 Class: 2 Class: 3 Class: 4 Class: 5
Sensitivity           0.44444   0.7692   0.8095   0.5753  0.20513
Specificity           1.00000   0.8392   0.8299   0.7770  0.98901
Pos Pred Value        1.00000   0.5208   0.5100   0.4038  0.84211
Neg Pred Value        0.87461   0.9412   0.9522   0.8745  0.81325
Prevalence            0.20513   0.1852   0.1795   0.2080  0.22222
Detection Rate        0.09117   0.1425   0.1453   0.1197  0.04558
Detection Prevalence  0.09117   0.2735   0.2849   0.2963  0.05413
Balanced Accuracy     0.72222   0.8042   0.8197   0.6762  0.59707
```

randomForest(may have a unique problem/error here.It seems to require line breaks from time to time, with many entries)

```
set.seed(1)
rfAgeN489 <- randomForest(Age ~ ENSMUSG00000048330 + ENSMUSG00000026043 +
ENSMUSG00000031502 + ENSMUSG00000070436 + ENSMUSG00000018593 +
ENSMUSG00000029661 + ENSMUSG00000042834 + ENSMUSG00000001506 +
ENSMUSG00000055116 + ENSMUSG00000038776 + ENSMUSG00000038007 +
ENSMUSG00000076617 + ENSMUSG00000031274 + ENSMUSG00000014776 +
ENSMUSG00000030256 + ENSMUSG00000073418 + ENSMUSG00000031503 +
ENSMUSG00000031167 + ENSMUSG00000029762 + ENSMUSG00000026566 +
ENSMUSG00000046805 + ENSMUSG00000030681 + ENSMUSG00000059824 +
ENSMUSG00000031239 + ENSMUSG00000033249 + ENSMUSG00000029675 +
ENSMUSG00000026077 + ENSMUSG00000018774 + ENSMUSG00000026042 +
ENSMUSG00000051579 + ENSMUSG00000039745 + ENSMUSG00000114277 +
ENSMUSG00000021775 + ENSMUSG00000028957 + ENSMUSG00000000682 +
ENSMUSG00000021814 + ENSMUSG00000022952 + ENSMUSG00000027864 +
ENSMUSG00000019055 + ENSMUSG00000042487 + ENSMUSG00000022389 +
ENSMUSG00000020571 + ENSMUSG00000020674 + ENSMUSG00000034116 +
ENSMUSG00000056737 + ENSMUSG00000034205 + ENSMUSG00000036545 +
ENSMUSG00000028970 + ENSMUSG00000054612 + ENSMUSG00000058818 +
ENSMUSG00000108218 + ENSMUSG00000040713 + ENSMUSG00000027995 +
ENSMUSG00000059182 + ENSMUSG00000030357 + ENSMUSG00000020120 +
ENSMUSG00000063275 + ENSMUSG00000037411 + ENSMUSG00000032010 +
ENSMUSG00000020661 + ENSMUSG00000021091 + ENSMUSG00000057531 +
ENSMUSG00000030701 + ENSMUSG00000066245 + ENSMUSG00000031762 +
ENSMUSG00000003814 + ENSMUSG00000027962 + ENSMUSG00000024501 +
ENSMUSG00000044949 + ENSMUSG00000016256 + ENSMUSG00000003949 +
ENSMUSG00000026837 + ENSMUSG00000074264 + ENSMUSG00000032372 +
ENSMUSG00000055866 + ENSMUSG00000055980 + ENSMUSG00000070031 +
ENSMUSG00000026478 + ENSMUSG00000068854 + ENSMUSG00000040631 +
ENSMUSG00000035824 + ENSMUSG00000043631 + ENSMUSG00000089715 +
ENSMUSG00000057897 + ENSMUSG00000029657 + ENSMUSG00000056749 +
ENSMUSG00000049047 + ENSMUSG00000059970 + ENSMUSG00000027274 +
ENSMUSG00000038147 + ENSMUSG00000029468 + ENSMUSG00000001229 +
ENSMUSG00000005397 + ENSMUSG00000020893 + ENSMUSG00000015340 +
ENSMUSG00000026234 + ENSMUSG00000003617 + ENSMUSG00000028343 +
ENSMUSG00000024810 + ENSMUSG00000030096 + ENSMUSG00000095041 +
ENSMUSG00000020467 + ENSMUSG00000006386 + ENSMUSG00000037461 +
ENSMUSG00000003849 + ENSMUSG00000064267 + ENSMUSG00000031343 +
ENSMUSG00000020935 + ENSMUSG00000034341 + ENSMUSG00000034832 +
ENSMUSG00000037003 + ENSMUSG00000026786 + ENSMUSG00000020810 +
ENSMUSG00000028339 + ENSMUSG00000063171 + ENSMUSG00000022148 +
ENSMUSG00000039126 + ENSMUSG00000028859 + ENSMUSG00000034993 +
ENSMUSG00000034707 + ENSMUSG00000029919 + ENSMUSG00000001143 +
ENSMUSG00000031790 + ENSMUSG00000059714 + ENSMUSG00000079293 +
ENSMUSG00000031511 + ENSMUSG00000017707 + ENSMUSG00000026880 +
ENSMUSG00000058655 + ENSMUSG00000017754 + ENSMUSG00000020227 +
ENSMUSG00000042155 + ENSMUSG00000029687 + ENSMUSG00000024299 +
ENSMUSG00000038305 + ENSMUSG00000052915 + ENSMUSG00000042644 +
ENSMUSG00000037712 + ENSMUSG00000026956 + ENSMUSG00000047238 +
ENSMUSG00000028756 + ENSMUSG00000024966 + ENSMUSG00000044583 +
ENSMUSG00000022440 + ENSMUSG00000020048 + ENSMUSG00000041801 +
ENSMUSG00000031740 + ENSMUSG00000076435 + ENSMUSG00000037405 +
ENSMUSG00000000290 + ENSMUSG00000026074 + ENSMUSG00000028028 +
ENSMUSG00000006611 + ENSMUSG00000035245 + ENSMUSG00000099875 +
ENSMUSG00000034285 + ENSMUSG00000032802 + ENSMUSG00000050708 +
```

ENSMUSG00000001119 + ENSMUSG00000068742 + ENSMUSG00000028826 +
ENSMUSG00000038642 + ENSMUSG00000025265 + ENSMUSG00000040820 +
ENSMUSG00000022831 + ENSMUSG00000030103 + ENSMUSG00000003283 +
ENSMUSG00000041355 + ENSMUSG00000035828 + ENSMUSG00000021037 +
ENSMUSG00000060373 + ENSMUSG00000034614 + ENSMUSG00000027852 +
ENSMUSG00000048489 + ENSMUSG00000005936 + ENSMUSG00000021903 +
ENSMUSG00000031379 + ENSMUSG00000025586 + ENSMUSG00000028862 +
ENSMUSG00000025017 + ENSMUSG00000021003 + ENSMUSG00000034795 +
ENSMUSG00000036887 + ENSMUSG00000056228 + ENSMUSG00000011256 +
ENSMUSG00000063576 + ENSMUSG00000018042 + ENSMUSG00000028885 +
ENSMUSG00000028597 + ENSMUSG00000060671 + ENSMUSG00000078945 +
ENSMUSG00000003154 + ENSMUSG00000004637 + ENSMUSG00000032125 +
ENSMUSG00000032086 + ENSMUSG00000022186 + ENSMUSG00000038143 +
ENSMUSG00000027669 + ENSMUSG00000025158 + ENSMUSG00000096929 +
ENSMUSG00000040751 + ENSMUSG00000024164 + ENSMUSG00000025743 +
ENSMUSG00000004460 + ENSMUSG00000020111 + ENSMUSG00000028410 +
ENSMUSG00000027204 + ENSMUSG00000029994 + ENSMUSG00000074748 +
ENSMUSG00000071650 + ENSMUSG00000030172 + ENSMUSG00000055541 +
ENSMUSG00000019923 + ENSMUSG00000023952 + ENSMUSG00000033318 +
ENSMUSG00000033220 + ENSMUSG00000021262 + ENSMUSG00000071715 +
ENSMUSG00000034647 + ENSMUSG00000042129 + ENSMUSG00000053898 +
ENSMUSG00000031016 + ENSMUSG00000024538 + ENSMUSG00000046532 +
ENSMUSG00000029471 + ENSMUSG00000050335 + ENSMUSG00000028766 +
ENSMUSG00000031479 + ENSMUSG00000026739 + ENSMUSG00000030678 +
ENSMUSG00000025176 + ENSMUSG00000028581 + ENSMUSG00000029616 +
ENSMUSG00000022816 + ENSMUSG00000026979 + ENSMUSG00000039328 +
ENSMUSG00000052760 + ENSMUSG00000022415 + ENSMUSG00000030798 +
ENSMUSG00000038695 + ENSMUSG00000039982 + ENSMUSG00000028878 +
ENSMUSG00000024885 + ENSMUSG00000004609 + ENSMUSG00000020143 +
ENSMUSG00000023249 + ENSMUSG00000046318 + ENSMUSG00000007891 +
ENSMUSG00000040170 + ENSMUSG00000037860 + ENSMUSG00000009406 +
ENSMUSG00000022488 + ENSMUSG00000031778 + ENSMUSG00000026608 +
ENSMUSG00000024900 + ENSMUSG00000025511 + ENSMUSG00000015950 +
ENSMUSG00000021559 + ENSMUSG00000079419 + ENSMUSG00000071714 +
ENSMUSG00000097415 + ENSMUSG00000053063 + ENSMUSG00000041117 +
ENSMUSG00000022372 + ENSMUSG00000040552 + ENSMUSG00000027435 +
ENSMUSG00000036896 + ENSMUSG00000041040 + ENSMUSG00000052593 +
ENSMUSG00000034674 + ENSMUSG00000021998 + ENSMUSG00000053279 +
ENSMUSG00000051864 + ENSMUSG00000033581 + ENSMUSG00000054509 +
ENSMUSG00000024679 + ENSMUSG00000032000 + ENSMUSG00000032369 +
ENSMUSG00000024222 + ENSMUSG00000036594 + ENSMUSG00000016946 +
ENSMUSG00000041084 + ENSMUSG00000054863 + ENSMUSG00000033032 +
ENSMUSG00000036905 + ENSMUSG00000026548 + ENSMUSG00000039176 +
ENSMUSG00000025821 + ENSMUSG00000030087 + ENSMUSG00000036934 +
ENSMUSG00000031144 + ENSMUSG00000018415 + ENSMUSG00000047866 +
ENSMUSG00000020889 + ENSMUSG00000022035 + ENSMUSG00000110588 +
ENSMUSG00000035206 + ENSMUSG00000040612 + ENSMUSG00000033460 +
ENSMUSG00000025584 + ENSMUSG00000034154 + ENSMUSG00000079037 +
ENSMUSG00000041849 + ENSMUSG00000026303 + ENSMUSG00000073409 +
ENSMUSG00000026003 + ENSMUSG00000002763 + ENSMUSG00000033863 +
ENSMUSG00000000154 + ENSMUSG00000020241 + ENSMUSG00000066640 +
ENSMUSG00000056666 + ENSMUSG00000035064 + ENSMUSG00000052139 +
ENSMUSG00000059851 + ENSMUSG00000040097 + ENSMUSG00000031770 +
ENSMUSG00000029053 + ENSMUSG00000035513 + ENSMUSG00000036667 +
ENSMUSG00000004105 + ENSMUSG00000036402 + ENSMUSG00000024300 +
ENSMUSG00000031765 + ENSMUSG00000027933 + ENSMUSG00000026082 +
ENSMUSG00000033933 + ENSMUSG00000018427 + ENSMUSG00000030786 +
ENSMUSG00000041779 + ENSMUSG00000071203 + ENSMUSG00000022091 +

```
ENSMUSG00000027843 + ENSMUSG00000031221 + ENSMUSG00000032348 +
ENSMUSG00000018654 + ENSMUSG00000001930 + ENSMUSG00000020044 +
ENSMUSG00000036427 + ENSMUSG00000024736 + ENSMUSG00000063065 +
ENSMUSG00000025888 + ENSMUSG00000071637 + ENSMUSG00000003541 +
ENSMUSG00000022656 + ENSMUSG00000042688 + ENSMUSG00000064346 +
ENSMUSG00000058715 + ENSMUSG00000032127 + ENSMUSG00000028630 +
ENSMUSG00000003031 + ENSMUSG00000041515 + ENSMUSG00000026395 +
ENSMUSG00000033191 + ENSMUSG00000027947 + ENSMUSG00000090877 +
ENSMUSG00000022146 + ENSMUSG00000022848 + ENSMUSG00000078566 +
ENSMUSG00000028641 + ENSMUSG00000005107 + ENSMUSG00000025980 +
ENSMUSG00000027248 + ENSMUSG00000020009 + ENSMUSG00000069516 +
ENSMUSG00000059013 + ENSMUSG00000033308 + ENSMUSG00000075284 +
ENSMUSG00000024180 + ENSMUSG00000015468 + ENSMUSG00000041936 +
ENSMUSG00000060586 + ENSMUSG00000036944 + ENSMUSG00000037613 +
ENSMUSG00000055067 + ENSMUSG00000026864 + ENSMUSG00000058586 +
ENSMUSG00000037892 + ENSMUSG00000001663 + ENSMUSG00000032115 +
ENSMUSG00000030921 + ENSMUSG00000031024 + ENSMUSG00000031467 +
ENSMUSG00000007777 + ENSMUSG00000033082 + ENSMUSG00000038521 +
ENSMUSG00000016206 + ENSMUSG00000026672 + ENSMUSG00000002900 +
ENSMUSG00000060036 + ENSMUSG00000040174 + ENSMUSG00000045193 +
ENSMUSG00000025512 + ENSMUSG00000015839 + ENSMUSG00000054733 +
ENSMUSG00000029177 + ENSMUSG00000005686 + ENSMUSG00000035004 +
ENSMUSG00000021508 + ENSMUSG00000020277 + ENSMUSG00000031298 +
ENSMUSG00000056515 + ENSMUSG00000003731 + ENSMUSG00000042712 +
ENSMUSG00000022665 + ENSMUSG00000051506 + ENSMUSG00000042677 +
ENSMUSG00000029778 + ENSMUSG00000034994 + ENSMUSG00000079491 +
ENSMUSG00000020288 + ENSMUSG00000037455 + ENSMUSG00000034349 +
ENSMUSG00000021025 + ENSMUSG00000018666 + ENSMUSG00000035273 +
ENSMUSG00000020257 + ENSMUSG00000019846 + ENSMUSG00000037824 +
ENSMUSG00000036893 + ENSMUSG00000078771 + ENSMUSG00000030579 +
ENSMUSG00000036040 + ENSMUSG00000041926 + ENSMUSG00000022270 +
ENSMUSG00000073421 + ENSMUSG00000080316 + ENSMUSG00000023960 +
ENSMUSG00000050103 + ENSMUSG00000057406 + ENSMUSG00000030711 +
ENSMUSG00000005447 + ENSMUSG00000035504 + ENSMUSG00000029312 +
ENSMUSG00000044349 + ENSMUSG00000029009 + ENSMUSG00000034947 +
ENSMUSG00000046688 + ENSMUSG00000025037 + ENSMUSG00000042099 +
ENSMUSG00000028329 + ENSMUSG00000029330 + ENSMUSG00000039254 +
ENSMUSG00000041560 + ENSMUSG00000020034 + ENSMUSG00000021939 +
ENSMUSG00000049804 + ENSMUSG00000047045 + ENSMUSG00000069833 +
ENSMUSG00000040093 + ENSMUSG00000066687 + ENSMUSG00000000823 +
ENSMUSG00000028082 + ENSMUSG00000061740 + ENSMUSG00000028795 +
ENSMUSG00000030761 + ENSMUSG00000060989 + ENSMUSG00000019843 +
ENSMUSG00000049076 + ENSMUSG00000028646 + ENSMUSG00000023367 +
ENSMUSG00000022472 + ENSMUSG00000035834 + ENSMUSG00000036862 +
ENSMUSG00000034936 + ENSMUSG00000034656 + ENSMUSG00000039096 +
ENSMUSG00000036615 + ENSMUSG00000037347 + ENSMUSG00000079363 +
ENSMUSG00000006127 + ENSMUSG00000032399 + ENSMUSG00000022797 +
ENSMUSG00000061838 + ENSMUSG00000028673 + ENSMUSG00000025133 +
ENSMUSG00000024190 + ENSMUSG00000031232 + ENSMUSG00000019872 +
ENSMUSG00000019916 + ENSMUSG00000078922 + ENSMUSG00000013150 +
ENSMUSG00000058587 + ENSMUSG00000036275 + ENSMUSG00000025885 +
ENSMUSG00000042035 + ENSMUSG00000014361 + ENSMUSG00000026556 +
ENSMUSG00000025986,
data = train50)
pAgeN489 <- predict(rfAgeN489, test50)
pAgeN489r=round(pAgeN489)
confusionMatrix(as.factor(pAgeN489r), as.factor(test50$Age))
```

```
confusionMatrix(as.factor(pAgeN489r), as.factor(test50$Age))
Confusion Matrix and Statistics

          Reference
Prediction  1  2  3  4  5
         1 45  0  0  0  0
         2 26 59  1  0  0
         3  1  6 59  5  2
         4  0  0  3 64  8
         5  0  0  0  4 68

Overall Statistics

               Accuracy : 0.8405
                 95% CI : (0.7979, 0.8772)
    No Information Rate : 0.2222
    P-Value [Acc > NIR] : < 2.2e-16

                  Kappa : 0.8008

 Mcnemar's Test P-Value : NA

Statistics by Class:

                     Class: 1 Class: 2 Class: 3 Class: 4 Class: 5
Sensitivity            0.6250   0.9077   0.9365   0.8767   0.8718
Specificity            1.0000   0.9056   0.9514   0.9604   0.9853
Pos Pred Value         1.0000   0.6860   0.8082   0.8533   0.9444
Neg Pred Value         0.9118   0.9774   0.9856   0.9674   0.9642
Prevalence             0.2051   0.1852   0.1795   0.2080   0.2222
Detection Rate         0.1282   0.1681   0.1681   0.1823   0.1937
Detection Prevalence   0.1282   0.2450   0.2080   0.2137   0.2051
Balanced Accuracy      0.8125   0.9066   0.9439   0.9186   0.9286
```

categorical

```
data_New1$Age <-as.factor(data_New1$Age)

set.seed(1)
ind50 <- sample(2, nrow(data_New1), replace = T, prob = c(0.5, 0.5))
train50 <- data_New1[ind50==1,]
test50 <- data_New1[ind50==2,]

set.seed(1)
rf50 <- randomForest(Age~., data = train50)
p50 <- predict(rf50, test50)
confusionMatrix(as.factor(p50), as.factor(test50$Age))
```

```
confusionMatrix(as.factor(p50), as.factor(test50$Age))
Confusion Matrix and Statistics

          Reference
Prediction  1  2  3  4  5
         1 57 13  2  1  0
         2  8 46 18  7  1
         3  2 10 38 13  1
         4  0  2 11 40 15
         5  0  0  1  9 65

Overall Statistics

               Accuracy : 0.6833
                 95% CI : (0.6325, 0.7311)
    No Information Rate : 0.2278
    P-Value [Acc > NIR] : < 2.2e-16

                  Kappa : 0.604

 Mcnemar's Test P-Value : NA

Statistics by Class:

                     Class: 1 Class: 2 Class: 3 Class: 4
Sensitivity            0.8507   0.6479   0.5429   0.5714
Specificity            0.9454   0.8824   0.9103   0.9034
Pos Pred Value         0.7808   0.5750   0.5938   0.5882
Neg Pred Value         0.9652   0.9107   0.8919   0.8973
Prevalence             0.1861   0.1972   0.1944   0.1944
Detection Rate         0.1583   0.1278   0.1056   0.1111
Detection Prevalence   0.2028   0.2222   0.1778   0.1889
Balanced Accuracy      0.8981   0.7651   0.7266   0.7374
                     Class: 5
Sensitivity            0.7927
Specificity            0.9640
Pos Pred Value         0.8667
Neg Pred Value         0.9404
Prevalence             0.2278
Detection Rate         0.1806
Detection Prevalence   0.2083
Balanced Accuracy      0.8784
```

randomForest(may have a unique problem/error here.It seems to require line breaks from time to time, with many entries)

```
set.seed(1)
rfAgeC571 <- randomForest(Age ~ ENSMUSG00000048330 + ENSMUSG00000018593 +
ENSMUSG00000026043 + ENSMUSG00000029661 + ENSMUSG00000055116 +
ENSMUSG00000031502 + ENSMUSG00000038007 + ENSMUSG00000059824 +
ENSMUSG00000001506 + ENSMUSG00000070436 + ENSMUSG00000038776 +
ENSMUSG00000030256 + ENSMUSG00000031239 + ENSMUSG00000031274 +
ENSMUSG00000026077 + ENSMUSG00000022389 + ENSMUSG00000073418 +
ENSMUSG00000028957 + ENSMUSG00000046805 + ENSMUSG00000021775 +
ENSMUSG00000042834 + ENSMUSG00000042487 + ENSMUSG00000029762 +
ENSMUSG00000032010 + ENSMUSG00000026566 + ENSMUSG00000055866 +
ENSMUSG00000031167 + ENSMUSG00000029675 + ENSMUSG00000031503 +
ENSMUSG00000031762 + ENSMUSG00000076617 + ENSMUSG00000018774 +
ENSMUSG00000003949 + ENSMUSG00000033249 + ENSMUSG00000030681 +
ENSMUSG00000026042 + ENSMUSG00000022952 + ENSMUSG00000039745 +
ENSMUSG00000014776 + ENSMUSG00000036545 + ENSMUSG00000030701 +
ENSMUSG00000037411 + ENSMUSG00000037003 + ENSMUSG00000006386 +
ENSMUSG00000034205 + ENSMUSG00000020893 + ENSMUSG00000056737 +
ENSMUSG00000000682 + ENSMUSG00000019055 + ENSMUSG00000114277 +
ENSMUSG00000028862 + ENSMUSG00000108218 + ENSMUSG00000051579 +
ENSMUSG00000031016 + ENSMUSG00000074264 + ENSMUSG00000027962 +
ENSMUSG00000020120 + ENSMUSG00000017707 + ENSMUSG00000026837 +
ENSMUSG00000001119 + ENSMUSG00000028826 + ENSMUSG00000030357 +
ENSMUSG00000027864 + ENSMUSG00000022440 + ENSMUSG00000016256 +
ENSMUSG00000053898 + ENSMUSG00000063275 + ENSMUSG00000059182 +
ENSMUSG00000021091 + ENSMUSG00000040713 + ENSMUSG00000058818 +
ENSMUSG00000015340 + ENSMUSG00000054612 + ENSMUSG00000020674 +
ENSMUSG00000026003 + ENSMUSG00000033863 + ENSMUSG00000038147 +
ENSMUSG00000034116 + ENSMUSG00000029687 + ENSMUSG00000020467 +
ENSMUSG00000056749 + ENSMUSG00000026478 + ENSMUSG00000028970 +
ENSMUSG00000026880 + ENSMUSG00000035828 + ENSMUSG00000068742 +
ENSMUSG00000035824 + ENSMUSG00000003849 + ENSMUSG00000026234 +
ENSMUSG00000020661 + ENSMUSG00000055980 + ENSMUSG00000031740 +
ENSMUSG00000030096 + ENSMUSG00000058655 + ENSMUSG00000030103 +
ENSMUSG00000089715 + ENSMUSG00000027995 + ENSMUSG00000021814 +
ENSMUSG00000031479 + ENSMUSG00000020889 + ENSMUSG00000066245 +
ENSMUSG00000096929 + ENSMUSG00000028878 + ENSMUSG00000057897 +
ENSMUSG00000034832 + ENSMUSG00000028756 + ENSMUSG00000033032 +
ENSMUSG00000020571 + ENSMUSG00000027274 + ENSMUSG00000064267 +
ENSMUSG00000003617 + ENSMUSG00000034993 + ENSMUSG00000023952 +
ENSMUSG00000005397 + ENSMUSG00000034285 + ENSMUSG00000029657 +
ENSMUSG00000034707 + ENSMUSG00000034614 + ENSMUSG00000020935 +
ENSMUSG00000025158 + ENSMUSG00000057531 + ENSMUSG00000015468 +
ENSMUSG00000035064 + ENSMUSG00000031765 + ENSMUSG00000032372 +
ENSMUSG00000038143 + ENSMUSG00000029468 + ENSMUSG00000031770 +
ENSMUSG00000020810 + ENSMUSG00000025511 + ENSMUSG00000028339 +
ENSMUSG00000024966 + ENSMUSG00000024900 + ENSMUSG00000023328 +
ENSMUSG00000000290 + ENSMUSG00000042644 + ENSMUSG00000020241 +
ENSMUSG00000046532 + ENSMUSG00000079363 + ENSMUSG00000003814 +
ENSMUSG00000034341 + ENSMUSG00000044583 + ENSMUSG00000035245 +
ENSMUSG00000001229 + ENSMUSG00000078922 + ENSMUSG00000022091 +
ENSMUSG00000041828 + ENSMUSG00000110588 + ENSMUSG00000004609 +
ENSMUSG00000028028 + ENSMUSG00000025743 + ENSMUSG00000068854 +
ENSMUSG00000022148 + ENSMUSG00000027852 + ENSMUSG00000028859 +
ENSMUSG00000041801 + ENSMUSG00000034647 + ENSMUSG00000040631 +
```

ENSMUSG00000036040 + ENSMUSG00000097415 + ENSMUSG00000037461 +
ENSMUSG00000003283 + ENSMUSG00000031343 + ENSMUSG00000057193 +
ENSMUSG00000029330 + ENSMUSG00000068290 + ENSMUSG00000079293 +
ENSMUSG00000029919 + ENSMUSG00000021903 + ENSMUSG00000071650 +
ENSMUSG00000038642 + ENSMUSG00000022831 + ENSMUSG00000041355 +
ENSMUSG00000039126 + ENSMUSG00000071637 + ENSMUSG00000070031 +
ENSMUSG00000026956 + ENSMUSG00000078945 + ENSMUSG00000049047 +
ENSMUSG00000054733 + ENSMUSG00000026786 + ENSMUSG00000028646 +
ENSMUSG00000028410 + ENSMUSG00000002985 + ENSMUSG00000046318 +
ENSMUSG00000040751 + ENSMUSG00000024222 + ENSMUSG00000026872 +
ENSMUSG00000059970 + ENSMUSG00000099875 + ENSMUSG00000031864 +
ENSMUSG00000059013 + ENSMUSG00000024299 + ENSMUSG00000027947 +
ENSMUSG00000032802 + ENSMUSG00000024810 + ENSMUSG00000044949 +
ENSMUSG00000063576 + ENSMUSG00000095041 + ENSMUSG00000024538 +
ENSMUSG00000024501 + ENSMUSG00000004105 + ENSMUSG00000028597 +
ENSMUSG00000056666 + ENSMUSG00000033220 + ENSMUSG00000090877 +
ENSMUSG00000029238 + ENSMUSG00000036594 + ENSMUSG00000056515 +
ENSMUSG00000027843 + ENSMUSG00000031221 + ENSMUSG00000034006 +
ENSMUSG00000060671 + ENSMUSG00000025017 + ENSMUSG00000004637 +
ENSMUSG00000050335 + ENSMUSG00000017754 + ENSMUSG00000066687 +
ENSMUSG00000076435 + ENSMUSG00000079419 + ENSMUSG00000032125 +
ENSMUSG00000026082 + ENSMUSG00000032369 + ENSMUSG00000040820 +
ENSMUSG00000020123 + ENSMUSG00000025986 + ENSMUSG00000059714 +
ENSMUSG00000001143 + ENSMUSG00000020009 + ENSMUSG00000064346 +
ENSMUSG00000032086 + ENSMUSG00000060989 + ENSMUSG00000040552 +
ENSMUSG00000056228 + ENSMUSG00000028581 + ENSMUSG00000025586 +
ENSMUSG00000029053 + ENSMUSG00000031232 + ENSMUSG00000021559 +
ENSMUSG00000057406 + ENSMUSG00000022186 + ENSMUSG00000052915 +
ENSMUSG00000058587 + ENSMUSG00000051864 + ENSMUSG00000037405 +
ENSMUSG00000028885 + ENSMUSG00000030087 + ENSMUSG00000033318 +
ENSMUSG00000048489 + ENSMUSG00000022488 + ENSMUSG00000020044 +
ENSMUSG00000003731 + ENSMUSG00000053063 + ENSMUSG00000034255 +
ENSMUSG00000037860 + ENSMUSG00000025265 + ENSMUSG00000021262 +
ENSMUSG00000020038 + ENSMUSG00000050708 + ENSMUSG00000035936 +
ENSMUSG00000022372 + ENSMUSG00000015950 + ENSMUSG00000025981 +
ENSMUSG00000053279 + ENSMUSG00000024885 + ENSMUSG00000055541 +
ENSMUSG00000022816 + ENSMUSG00000073940 + ENSMUSG00000020048 +
ENSMUSG00000041084 + ENSMUSG00000060373 + ENSMUSG00000018427 +
ENSMUSG00000043631 + ENSMUSG00000041040 + ENSMUSG00000075284 +
ENSMUSG00000021939 + ENSMUSG00000020227 + ENSMUSG00000026074 +
ENSMUSG00000024164 + ENSMUSG00000034795 + ENSMUSG00000035834 +
ENSMUSG00000028630 + ENSMUSG00000031431 + ENSMUSG00000020594 +
ENSMUSG00000003541 + ENSMUSG00000028343 + ENSMUSG00000010651 +
ENSMUSG00000027330 + ENSMUSG00000021003 + ENSMUSG00000036887 +
ENSMUSG00000020053 + ENSMUSG00000040093 + ENSMUSG00000042099 +
ENSMUSG00000021025 + ENSMUSG00000026608 + ENSMUSG00000031511 +
ENSMUSG00000018654 + ENSMUSG00000069516 + ENSMUSG00000030798 +
ENSMUSG00000019923 + ENSMUSG00000042155 + ENSMUSG00000002763 +
ENSMUSG00000021037 + ENSMUSG00000040170 + ENSMUSG00000045193 +
ENSMUSG00000039982 + ENSMUSG00000026739 + ENSMUSG00000058135 +
ENSMUSG00000003458 + ENSMUSG00000007891 + ENSMUSG00000027204 +
ENSMUSG00000034154 + ENSMUSG00000049076 + ENSMUSG00000032000 +
ENSMUSG00000024679 + ENSMUSG00000021188 + ENSMUSG00000078566 +
ENSMUSG00000052593 + ENSMUSG00000052760 + ENSMUSG00000030172 +
ENSMUSG00000024132 + ENSMUSG00000010554 + ENSMUSG00000037712 +
ENSMUSG00000024610 + ENSMUSG00000060586 + ENSMUSG00000035004 +
ENSMUSG00000054863 + ENSMUSG00000022912 + ENSMUSG00000020034 +
ENSMUSG00000024300 + ENSMUSG00000074748 + ENSMUSG00000039382 +

ENSMUSG00000063171 + ENSMUSG00000028309 + ENSMUSG00000111027 +
ENSMUSG00000020257 + ENSMUSG00000032228 + ENSMUSG00000057137 +
ENSMUSG00000037613 + ENSMUSG00000042426 + ENSMUSG00000071715 +
ENSMUSG00000020653 + ENSMUSG00000034349 + ENSMUSG00000005447 +
ENSMUSG00000029570 + ENSMUSG00000041992 + ENSMUSG00000071714 +
ENSMUSG00000022415 + ENSMUSG00000033581 + ENSMUSG00000018160 +
ENSMUSG00000042129 + ENSMUSG00000047045 + ENSMUSG00000026979 +
ENSMUSG00000031790 + ENSMUSG00000027933 + ENSMUSG00000021998 +
ENSMUSG00000007777 + ENSMUSG00000047866 + ENSMUSG00000038235 +
ENSMUSG00000039096 + ENSMUSG00000060862 + ENSMUSG00000017132 +
ENSMUSG00000033191 + ENSMUSG00000006127 + ENSMUSG00000039176 +
ENSMUSG00000036273 + ENSMUSG00000050714 + ENSMUSG00000005936 +
ENSMUSG00000018217 + ENSMUSG00000020954 + ENSMUSG00000020472 +
ENSMUSG00000026395 + ENSMUSG00000025888 + ENSMUSG00000032348 +
ENSMUSG00000029994 + ENSMUSG00000040723 + ENSMUSG00000057894 +
ENSMUSG00000034947 + ENSMUSG00000003031 + ENSMUSG00000035504 +
ENSMUSG00000063060 + ENSMUSG00000005417 + ENSMUSG00000038175 +
ENSMUSG00000059498 + ENSMUSG00000060036 + ENSMUSG00000030579 +
ENSMUSG00000030761 + ENSMUSG00000042436 + ENSMUSG00000031379 +
ENSMUSG00000034007 + ENSMUSG00000072949 + ENSMUSG00000023960 +
ENSMUSG00000003348 + ENSMUSG00000067274 + ENSMUSG00000025133 +
ENSMUSG00000018501 + ENSMUSG00000041560 + ENSMUSG00000015189 +
ENSMUSG00000042251 + ENSMUSG00000000056 + ENSMUSG00000022848 +
ENSMUSG00000028223 + ENSMUSG00000080316 + ENSMUSG00000027875 +
ENSMUSG00000022098 + ENSMUSG00000029090 + ENSMUSG00000015839 +
ENSMUSG00000063415 + ENSMUSG00000022665 + ENSMUSG00000018415 +
ENSMUSG00000070544 + ENSMUSG00000039254 + ENSMUSG00000042594 +
ENSMUSG00000021508 + ENSMUSG00000000184 + ENSMUSG00000025821 +
ENSMUSG00000009406 + ENSMUSG00000037892 + ENSMUSG00000049804 +
ENSMUSG00000036944 + ENSMUSG00000025584 + ENSMUSG00000052305 +
ENSMUSG00000006611 + ENSMUSG00000058715 + ENSMUSG00000073421 +
ENSMUSG00000020143 + ENSMUSG00000061950 + ENSMUSG00000013150 +
ENSMUSG00000028641 + ENSMUSG00000023367 + ENSMUSG00000028795 +
ENSMUSG00000041117 + ENSMUSG00000073409 + ENSMUSG00000049878 +
ENSMUSG00000016946 + ENSMUSG00000033460 + ENSMUSG00000028082 +
ENSMUSG00000017774 + ENSMUSG00000024421 + ENSMUSG00000006373 +
ENSMUSG00000035877 + ENSMUSG00000004460 + ENSMUSG00000028337 +
ENSMUSG00000003154 + ENSMUSG00000046994 + ENSMUSG00000018042 +
ENSMUSG00000035513 + ENSMUSG00000031778 + ENSMUSG00000026480 +
ENSMUSG00000041515 + ENSMUSG00000022197 + ENSMUSG00000030083 +
ENSMUSG00000092981 + ENSMUSG00000020111 + ENSMUSG00000035212 +
ENSMUSG00000054404 + ENSMUSG00000002996 + ENSMUSG00000028329 +
ENSMUSG00000020459 + ENSMUSG00000024190 + ENSMUSG00000024975 +
ENSMUSG00000028600 + ENSMUSG00000024576 + ENSMUSG00000038305 +
ENSMUSG00000037235 + ENSMUSG00000011256 + ENSMUSG00000027412 +
ENSMUSG00000024965 + ENSMUSG00000063663 + ENSMUSG00000008540 +
ENSMUSG00000030921 + ENSMUSG00000046743 + ENSMUSG00000018574 +
ENSMUSG00000057497 + ENSMUSG00000029119 + ENSMUSG00000032349 +
ENSMUSG00000001930 + ENSMUSG00000002017 + ENSMUSG00000027669 +
ENSMUSG00000031596 + ENSMUSG00000013275 + ENSMUSG00000038393 +
ENSMUSG00000026303 + ENSMUSG00000044562 + ENSMUSG00000055067 +
ENSMUSG00000032127 + ENSMUSG00000040612 + ENSMUSG00000034403 +
ENSMUSG00000034157 + ENSMUSG00000029229 + ENSMUSG00000034252 +
ENSMUSG00000026288 + ENSMUSG00000026548 + ENSMUSG00000047945 +
ENSMUSG00000039328 + ENSMUSG00000026728 + ENSMUSG00000025270 +
ENSMUSG00000037475 + ENSMUSG00000033499 + ENSMUSG00000022270 +
ENSMUSG00000030711 + ENSMUSG00000032399 + ENSMUSG00000029810 +
ENSMUSG00000046230 + ENSMUSG00000042677 + ENSMUSG00000048126 +

```
ENSMUSG00000001774 + ENSMUSG00000021306 + ENSMUSG00000051483 +
ENSMUSG00000008845 + ENSMUSG00000071203 + ENSMUSG00000021242 +
ENSMUSG00000024654 + ENSMUSG00000078853 + ENSMUSG00000033082 +
ENSMUSG00000054072 + ENSMUSG00000007617 + ENSMUSG00000023055 +
ENSMUSG00000036896 + ENSMUSG00000048756 + ENSMUSG00000022146 +
ENSMUSG00000061601 + ENSMUSG00000034674 + ENSMUSG00000054509 +
ENSMUSG00000019916 + ENSMUSG00000044349 + ENSMUSG00000021190 +
ENSMUSG00000024451 + ENSMUSG00000036402 + ENSMUSG00000022035 +
ENSMUSG00000014361 + ENSMUSG00000032363 + ENSMUSG00000022623 +
ENSMUSG00000079499 + ENSMUSG00000060771 + ENSMUSG00000037058 +
ENSMUSG00000027635 + ENSMUSG00000039159 + ENSMUSG00000033159 +
ENSMUSG00000036667 + ENSMUSG00000069833 + ENSMUSG00000062203 +
ENSMUSG00000028820 + ENSMUSG00000036275 + ENSMUSG00000024160 +
ENSMUSG00000019872 + ENSMUSG00000025358 + ENSMUSG00000002289 +
ENSMUSG00000041779 + ENSMUSG00000035206 + ENSMUSG00000031442 +
ENSMUSG00000036934 + ENSMUSG00000030678 + ENSMUSG00000022684 +
ENSMUSG00000052812 + ENSMUSG00000031217 + ENSMUSG00000029471 +
ENSMUSG00000041936 + ENSMUSG00000052040 + ENSMUSG00000041417 +
ENSMUSG00000036862 + ENSMUSG00000046312 + ENSMUSG00000048249 +
ENSMUSG00000038695 + ENSMUSG00000034936 + ENSMUSG00000022999 +
ENSMUSG00000059851 + ENSMUSG00000025262 + ENSMUSG00000042286 +
ENSMUSG00000035772 + ENSMUSG00000031004
, data = train50)
pAgeC571 <- predict(rfAgeC571, test50)
confusionMatrix(as.factor(pAgeC571), as.factor(test50$Age))
```

```
confusionMatrix(as.factor(pAgeC571), as.factor(test50$Age))
Confusion Matrix and Statistics

          Reference
Prediction  1  2  3  4  5
         1 62  7  0  0  0
         2  4 60 10  3  0
         3  1  4 50  9  2
         4  0  0  8 50 18
         5  0  0  2  8 62

Overall Statistics

               Accuracy : 0.7889
                 95% CI : (0.743, 0.8299)
    No Information Rate : 0.2278
    P-Value [Acc > NIR] : < 2.2e-16

                  Kappa : 0.7361

 Mcnemar's Test P-Value : NA

Statistics by Class:

                     Class: 1 Class: 2 Class: 3 Class: 4
Sensitivity            0.9254   0.8451   0.7143   0.7143
Specificity            0.9761   0.9412   0.9448   0.9103
Pos Pred Value         0.8986   0.7792   0.7576   0.6579
Neg Pred Value         0.9828   0.9611   0.9320   0.9296
Prevalence             0.1861   0.1972   0.1944   0.1944
Detection Rate         0.1722   0.1667   0.1389   0.1389
Detection Prevalence   0.1917   0.2139   0.1833   0.2111
Balanced Accuracy      0.9507   0.8931   0.8296   0.8123
                     Class: 5
Sensitivity            0.7561
```

```
Specificity            0.9640
Pos Pred Value         0.8611
Neg Pred Value         0.9306
Prevalence             0.2278
Detection Rate         0.1722
Detection Prevalence   0.2000
Balanced Accuracy      0.8601
```
